# The effects of genital myiasis on the diversity of the vaginal microbiota in female Bactrian camels

**DOI:** 10.1186/s12917-022-03189-5

**Published:** 2022-03-05

**Authors:** Likang Zhi, Dongdong Ai, Ming Yong, Huar Bao, Baoxiang Han, Bo Sun, Ya Tu, Demtu Er

**Affiliations:** 1 College of Veterinary Medicine, Key Laboratory of Clinical Diagnosis and Treatment Technology in Animal Disease, Ministry of Agriculture and Rural Affairs, Inner Mongolia Agricultural University, Hohhot, 010018 P.R. China; 2 Veterinary Administration Bureau of Bayannur City, Linhe District, Bayannur City, 015000 Inner Mongolia Autonomous Region P.R. China; 3 Alxa League Institute of Animal Health Supervision, Bayanhot, 750306 Inner Mongolia Autonomous Region P.R. China; 4 Detachment of Alxa League Agriculture and Animal Husbandry Comprehensive Administrative Law Enforcement, Bayanhot, 750306 Inner Mongolia Autonomous Region P.R. China

**Keywords:** Bacteriome, Bactrian camels, Genital myiasis, Vaginal flora diversity, 16S rRNA

## Abstract

**Background:**

Genital myasis is one of the most important diseases that affects the reproductive organs of Bactrian camels in which can cause serious mechanical damage to the vaginal tissue. The accumulation of bacteria in the vagina of female camels can affect their health and reproductive ability. The effect of this damage is commonly manifested in the vaginal flora and vaginal mucosal immune system. Therefore, this investigation is a study of the diversity of the vaginal flora and the differences between healthy Bactrian camels and those suffering from genital myiasis.

**Results:**

Vaginal microbiota samples were collected from two groups of female Bactrian camels of the same age. An Illumina MiSeq was used to sequence the 16S rRNA V3-V4 hypervariable sequence in the samples. The results showed that the vaginal microflora of the infected camels had a significantly greater operational taxonomic unit (OTU) value. According to the assessment of the alpha diversity index and the vaginal pH, the diversity index of the infected camel flora was higher than that of the normal camel flora, and the vaginal pH was lower than that of the normal camels (*p* < 0.01). There were no significant differences between the two groups in the abundance of dominant genera in the Bactrian camel vagina (*P* > 0.05), indicating that the certain stability is maintained.

**Conclusions:**

Overall, this comparison revealed the differences and similarities between the vaginal microbiota of Bactrian camels in various health statues. In addition, these data provide a reference point for understanding the types of bacteria that cause genital myiasis affecting the healthy development of Bactrian camels.

**Supplementary Information:**

The online version contains supplementary material available at 10.1186/s12917-022-03189-5.

## Background

The Bactrian camel is one of the unique domestic animals in China. It mainly lives in the hot and arid regions of the Gobi and desert area in northwestern China. It is known as the “boat of the desert” [1]. For a long time, the development of the Bactrian camel breeding industry has been hampered by genital myiasis, which has been linked to serious economic losses to local herders.

Genital myiasis of Bactrian camels is a serious parasitic disease. Larvae of *Wohlfahrtia magnifica* [2] parasitize the perineal and vaginal mucosal and cutaneous regions of Bactrian camels where cause traumatic lesions [3]. Genital myiasis has a distinct seasonality and occurs in May–September of each summer and autumn [4, 5]. Clinical symptoms in diseased camels are the result of severe mechanical damage to the affected tissue and mucosal sites, causing harmful effects, such as local inflammation, anxiety and anorexia [5–7]. Through long-term experimental observation, we found that the diseased camel’s vaginal wound is exposed to the external environment but is rarely infected or purulent. When the larvae of *Wohlfahrtia magnifica* are detached from the host, 94.5% of the diseased camel wounds spontaneously recover [2]. In addition, other important elements affect the vaginal microenvironment.

The vaginal mucosa in healthy animals is colonized by an equilibrated and dynamic population of aerobic, facultative anaerobic and obligate anaerobic microbes [8]. The vaginal flora constitutes a natural barrier formed on the surface of the vaginal mucosa, but some factors can disturb the balance in its composition [9]. Disruption of the vaginal microbiota equilibrium promotes infectious clinical syndromes with diverse symptoms, such as vaginal discharge, irritation, pruritus, and vulvar burning [10]. The formation of a reciprocal symbiotic relationship between the vaginal flora and the host is an important factor in maintaining the stability of the vaginal microenvironment. It is also a relevant component of the multifaceted resistance of female mammals to pathogen invasion. This has a major impact on the health and disease of the host organism. The vaginal microbiota has importance in preserving vaginal health and defending the host against disease [11]. Thus, the vaginal microbiome can indicate the health or disease status of the female camel and whether there are changes according to the treatment of any existing vaginal-related diseases [12–16].

At present, research on vaginal microbiology is mainly focused on humans. There are minimal data on the vaginal microflora in livestock species as well as its potential role in animal vaginal mucosal immunology. Research on the vaginal microbiology of Bactrian camels has not yet been conducted. Therefore, in this study, we completed the first high-throughput sequencing analysis of the vaginal flora of Bactrian camels. By comparing the diversity of the vaginal microbiota and differences between the diseased and healthy group in the same herd, the effects of environmental and nutritional factors on the vaginal bacterial community were eliminated, and an analysis was performed to determine the immune-related differences in microbiome composition.

Through this study, a new understanding of the vaginal mucosal immune mechanism of the Alxa Bactrian camel and its mechanism and resistance to vaginal myiasis caused by the larvae of *Wohlfahrtia magnifica* was obtained, which lays the foundation for future research and provides a new idea for the prevention and treatment of vaginal myiasis in the Alxa Bactrian camel.

## Results

### Vaginal pH

The results showed that the vaginal pH of the healthy group of Bactrian camels ranged from 7.47 to 8.23, with an average of 7.85 ± 0.13. The vaginal pH of the diseased group was significantly lower (*P* < 0.01) in the range of 7.18–7.61 with an average of 7.41 ± 0.11.

### Sequencing information

After optimization of quality control and chimaera removal, a total of 1,644,139 reads were obtained for all 23 samples, resulting in an average of 71,484 reads per sample (Table 1). Samples from the diseased group were taken and yielded a total of 744,455 reads, with an average of 77,446 ± 11,214 reads per sample. The healthy group samples yielded a total of 899,684 reads with an average of 69,206 ± 11,047 reads per sample. The results showed that there was no statistically significant difference in the number of optimized sequences between the two groups of samples (*P* > 0.05).

**Table 1:** Sequence and alpha diversity statistics of the 16S rRNA gene sequences for bacterial populations in the vagina of diseased and healthy camels

Group	Sample No	Average of sequence No	Simpson index	Chao1	ACE	Shannon index
**Diseased**	10	77,446 ± 11,214	0.94 ± 0.04	495.04 ± 230.85	497.33 ± 228.58	5.55 ± 1.05
**Healthy**	13	69,206 ± 11,047	0.94 ± 0.03	361.65 ± 147.45	364.26 ± 148.16	5.07 ± 0.59
*P*	——	0.28	0.98	0.11	0.11	0.17

### Alpha and beta diversity analyses

**Fig. 1:**
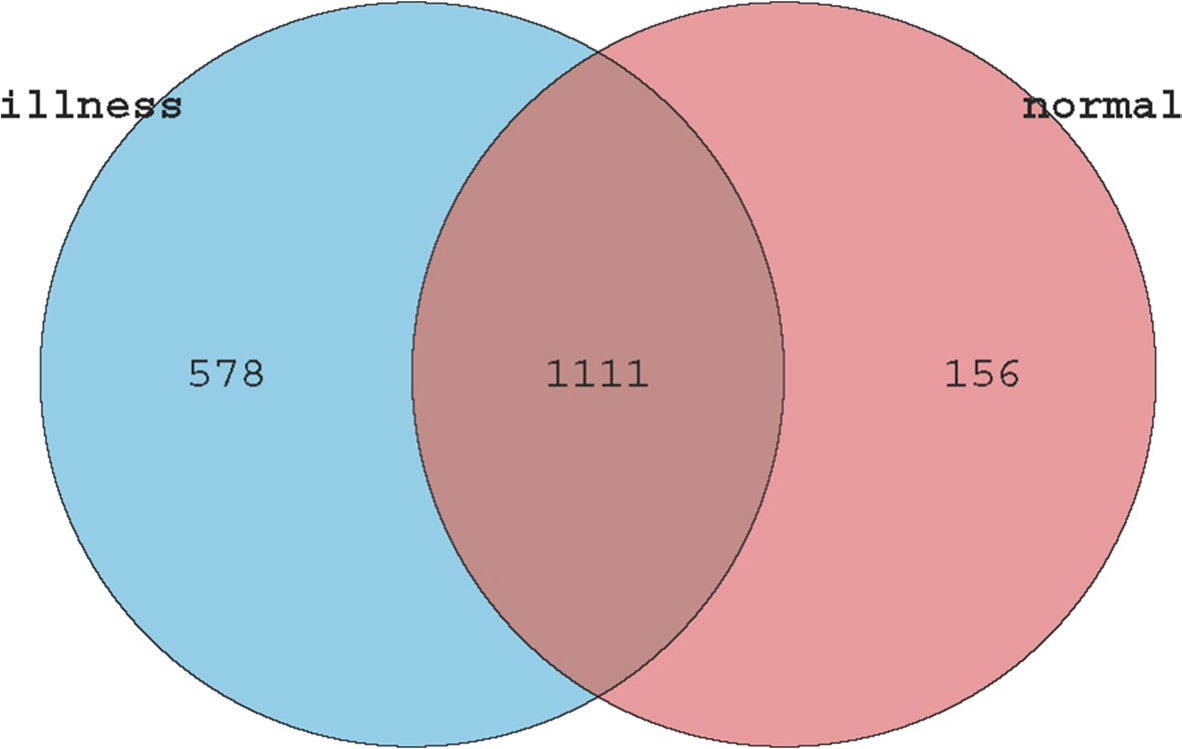
Total OTU Venn diagram

The sequences obtained above were subjected to merging and operational taxonomic unit (OTU) division based on 97% sequence similarity. Additionally, the OTUs with abundance values lower than 0.001% of the total sample sequencing amount were removed [17]. A total of 1845 OTUs were detected, with an average of 1689 in each sample. Moreover, 1267 OTUs were detected in the diseased group. In addition, the normal vaginal flora for each was maintained with 1111 OTUs shared between various vaginal environments (Fig. 1).

Alpha diversity was measured and observed using OTU, Chao1, ACE, Simpson (the Simpson diversity index is derived based on the assumption that two individuals are randomly selected in an infinite community and based on the probability that they belong to the same species) and Shannon diversity indices (the Shannon diversity index is a way to measure the diversity of species in a community). The conclusive analysis results are presented in Table 1. No significant differences existed in alpha diversity between the healthy samples of female Bactrian camels and those of ill camels, according to the vaginal bacteria observed by the OTU, Chao1, ACE, Simpson and Shannon’s diversity indices (*p* > 0.05). However, the vagina of diseased female Bactrian camels had a significantly greater number of OTUs than the normal Bactrian camel vagina, with increased richness as measured by Chao1 and ACE and greater diversity as measured by Shannon’s diversity index and the Simpson index, all of which are presented in Table 1.

**Fig. 2:**
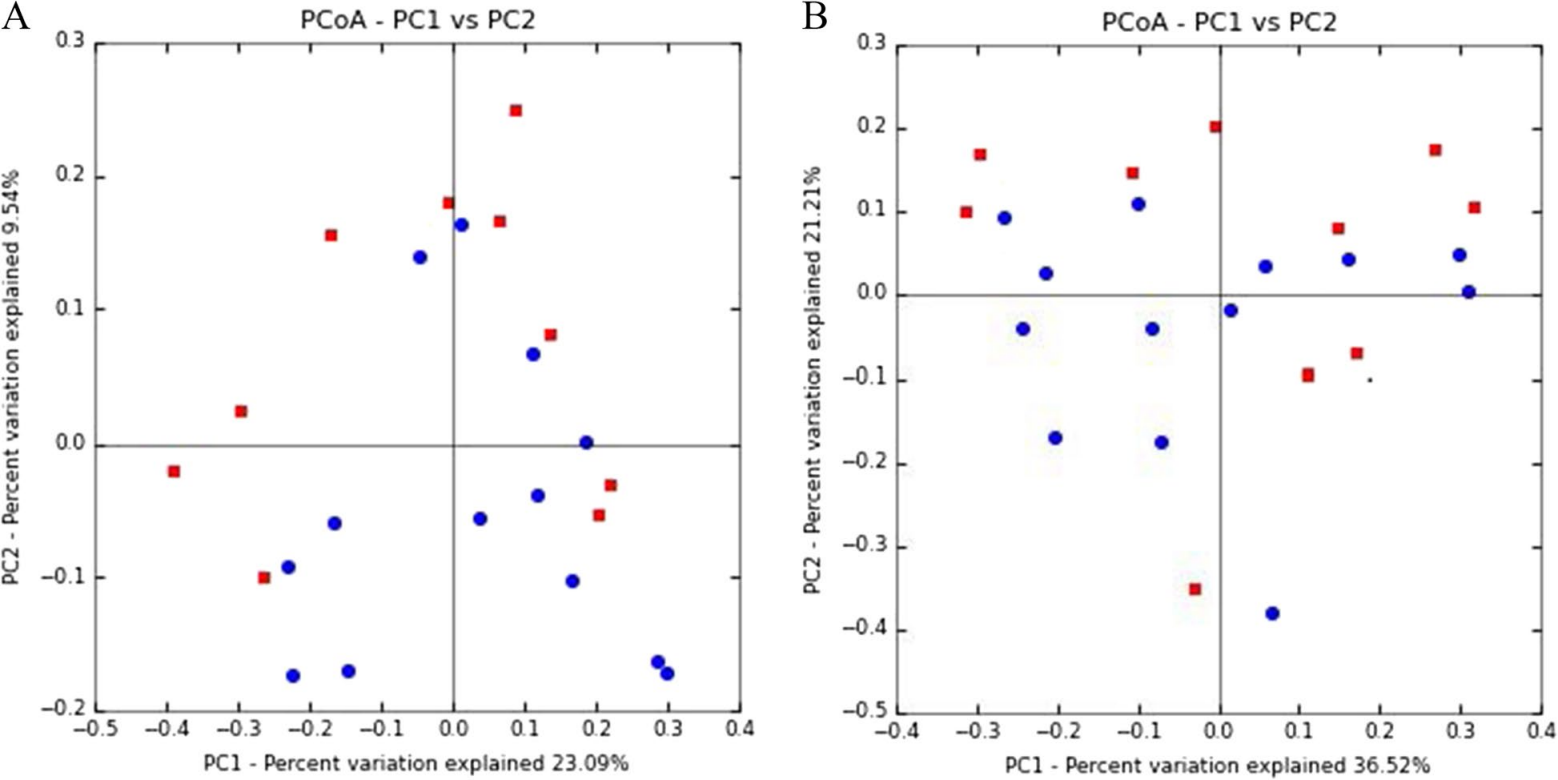
Principal coordinate analysis of vaginal samples from diseased female camels and healthy female camels using UniFrac unweighted (**A**) and weighted (**B**) metrics. Vaginal samples from diseased female camels (*n* = 10) are represented by red squares, and vaginal samples from healthy camels (*n* = 13) are represented by blue circles

Beta-diversity was also analyzed to examine differences in microbial communities between samples. Using an OTU-centric approach PCoA matrices were employed using weighted and unweighted UniFrac distance matrices to compare the phylogenetic divergence among the OTU between samples from ill camels and healthy camel vaginal samples (Fig. 2). The results showed that the subsets of healthy camel vaginal samples were more closely clustered in the weighted and unweighted UniFrac distance matrix. In addition, (Analysis of similarities) ANOSIM showed that there was a significant difference between the vaginal samples of diseased camels and healthy camels (*P* = 0.033). Statistical analysis showed that the difference between the groups was significantly greater than that within groups (*R* = 0.1483), and the grouping effect was evidently good.

### Taxonomic composition analysis

According to the results of OTU classification and classification status identification, the dominant vaginal flora and average relative abundance of Bactrian camels in the healthy group and the diseased group were, respectively, identified at the phylum level: Firmicutes (39.33% ± 16.228.34% ± 19.39); Proteobacteria (28.48% ± 15.0635.23% ± 14.18); Fusobacteria (16.12% ± 11.3216.86% ± 13.55); Bacteroidetes (6.04% ± 3.58.95% ± 3.6); and Actinobacteria (7.5% ± 3.07% ± 6.17% ± 4.21). The average relative abundance of unallocated taxa was (1.45% ± 2.81% ± 1.72% ± 2.81). The relative abundance of the dominant vaginal flora of the Bactrian camels was not significantly different between the healthy group and the diseased group (*P* > 0.05).

At the genus level, the dominant bacteria and their average relative abundance identified from the vaginas of Bactrian camels in the normal group and the group of ill female camels were Campylobacter (9.58% ± 7.03% ± 9.9% ± 10.05); Ochrobactrum (5.76% ± 6.037.56% ± 5.99); Fusobacterium (6.42% ± 5.596.6% ± 5.84); GW-34 (5.96% ± 7.535.58% ± 12.15); Porphyromonas (4.08% ± 3.574.96% ± 3.98); Facklamia (6.08% ± 4.582.33% ± 1.15); Sediminibacterium (1.45% ± 1.65% ± 2.48% ± 2.56); Helcococcus (1.25% ± 2.32.38% ± 4.05); Peptoniphilus (1.04% ± 1.052.11% ± 1.85); 1–68 (1.59% ± 2.220.93% ± 1.56); Clostridium (1.22% ± 1.071.23% ± 1.3); Acinetobacter (1.18% ± 1.281% ± 0.78); Fusibacter (1.29% ± 1.680.44% ± 0.62); Sphingomonas (0.68% ± 0.631.09% ± 0.86); and ph2 (0.9% ± 0.830.53% ± 0.98). The relative abundance of the dominant vaginal flora of the Bactrian camels was not significantly different between the healthy and diseased group (*P* > 0.05).

The genera of vaginal microorganisms that could not be identified or placed in defined categories in either the healthy or diseased Bactrian camels and their average relative abundances were as follows: family Leptotrichiaceae (9.58% ± 7.03% ± 9.9% ± 10.05), family Aerococcaceae (7.78% ± 5.182.71% ± 4.2), family Carnobacteriaceae 6.81% ± 7.080.3% ± 0.62), family Xanthomonadaceae (1.13% ± 1.092.97% ± 2.96), family Pseudomonadaceae (1.9% ± 2.520.9% ± 0.75), family Tissierellaceae (1.39% ± 1.491.12% ± 1.75), family Ruminococcaceae (0.35% ± 0.391.69% ± 3.14), family Enterobacteriaceae (0.47% ± 0.781.39% ± 2.04), and family Comamonadaceae (0.35% ± 0.391.02% ± 0.78).

**Fig. 3:**
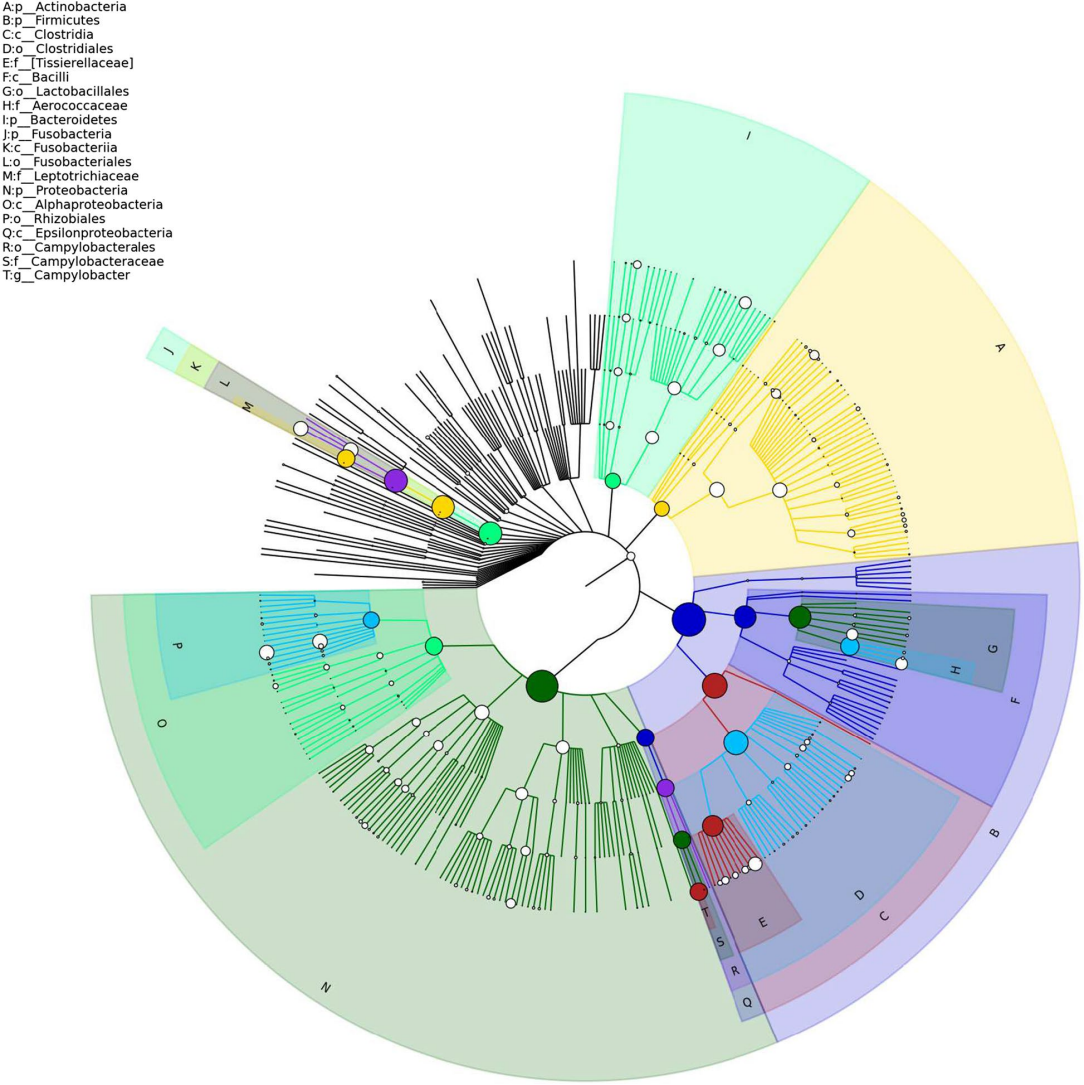
Sample overall classification-level tree diagram based on GraPhlAn Note: The classification level tree shows the hierarchical relationship of all classification units (represented by nodes) from the taxon to the genus (from the inner circle to the outer circle) in the sample population. The node size corresponds to the average relative abundance of the classification unit. The top 20 units of relative abundance are also identified by letters in the figure (from taxon to genus in order from outer layer to inner layer), and the shading colour on the letter is the same as the corresponding node colour

**Fig. 4:**
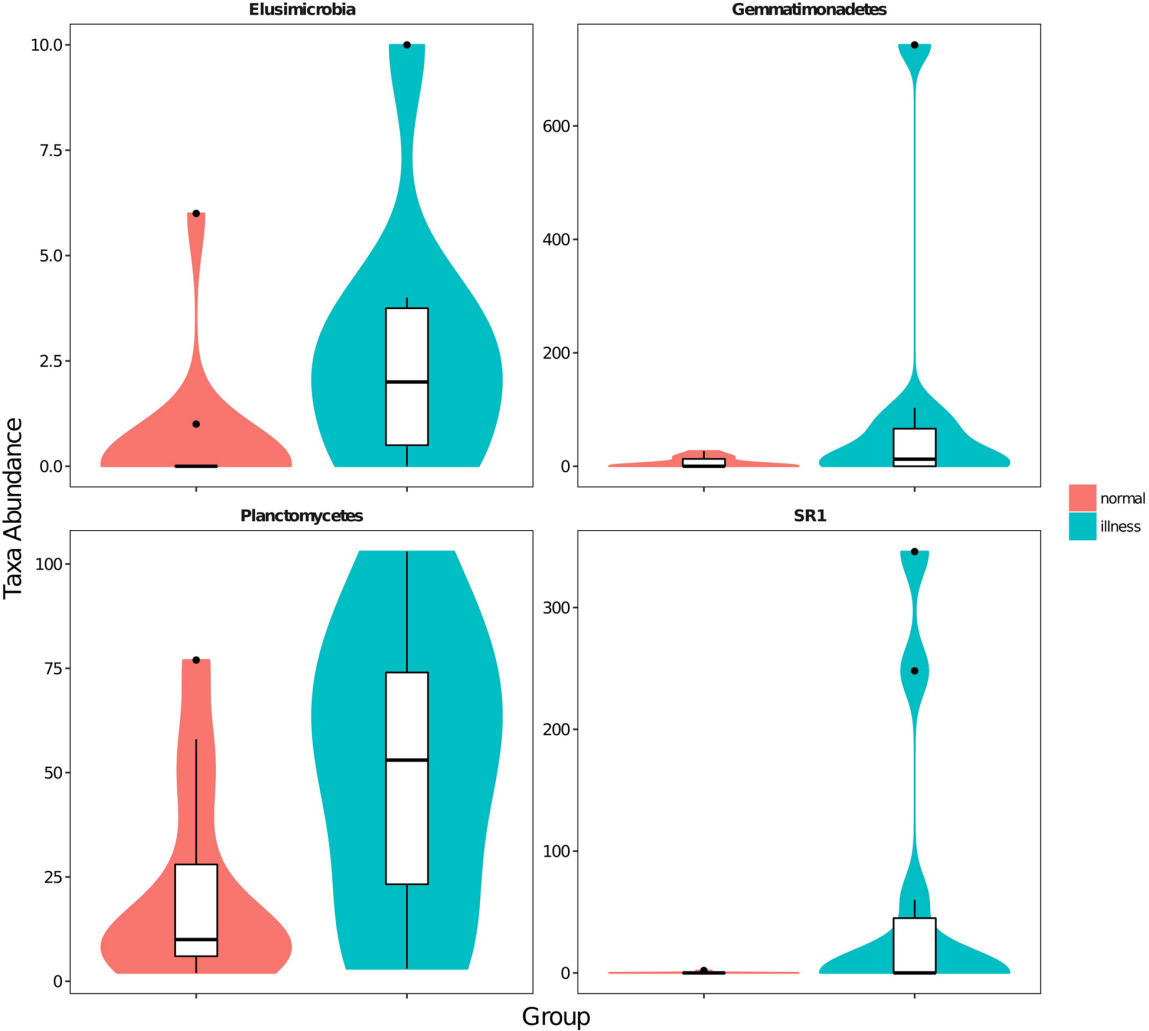
Abundance distribution of phylum-level taxa, with significant differences between sample groups

**Fig. 5:**
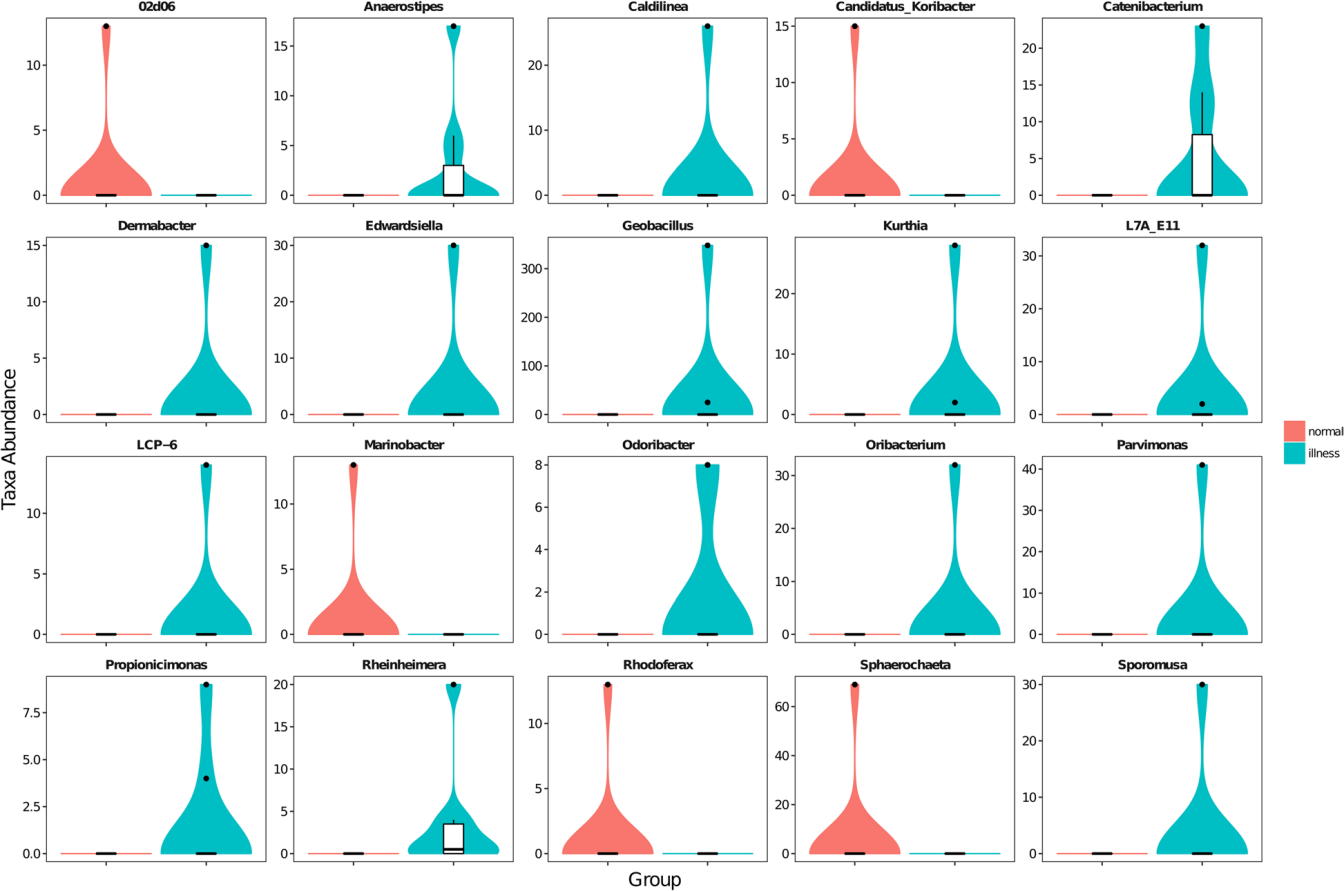
Abundance distribution of the top 20 taxa with significant differences at the genus level. Note: The abscissa in the figure is the taxonomic unit, which shows a significant difference, and the ordinate is the sequence quantity of each taxon in each sample group. The border of the figure represents the interquartile range (IQR), the horizontal line represents the median value, and the upper and lower extensions represent the 1.5-times IQR range, except the upper and lower quartiles. Additionally, the symbol "•" indicates an extreme value exceeding the range

The visualization tool GraPhlAn [18] was used to build a hierarchical tree of the composition of the sample population at each classification level (Fig. 3).Using mothur software, the statistical algorithm Metastats (http://metastats.cbcb.umd.edu/) [19] was used. We were able to determine the overall classification level of all classification units in the sample population. The difference of sequence quantity (i.e., absolute abundance) between each taxon at the phylum and genus levels was analyzed and compared (pairwise). We found that there were 4 classification units with significant differences in taxon levels (Fig. 4), namely, SR1 (*p* = 0.030 *q* = 0.120), Planctomycetes (*p* = 0.030 *q* = 0.120), Gemmatimonadetes (*p* = 0.041 *q* = 0.120), and Elusimicrobia (*p* = 0.048 *q* = 0.120). There were 51 taxonomies with significant differences in levels (Fig. 5), mainly Anaerostipes (*p* = 0.001 *q* = 0.005), Caldilinea (*p* = 0.001 *q* = 0.005), Edwardsiella (*p* = 0.001 *q* = 0.005), Lactobacillus (*p* = 0.027 *q* = 0.064), etc.

## Discussion

The implications and analysis of this study has provided further information the vaginal microecosystem of Bactrian camels. Relevant studies have proven that the combination of the microflora related to the human body can affect human immunity and provide the first line of defence against opportunistic pathogen colonization [20]. The importance of microbial metabolism to the host immune system can be revealed by characterizing the composition and function of individual microbial species and complex microbial communities [21]. This study is based on a basic research analysis. By comparing the differences in the structure and diversity of healthy camels and diseased camels, we were able to analyze the role of the vaginal microecosystem of Bactrian camels in their immunity and recovery stages after vaginal myiasis infection. Additional understanding of these stages may provide a new approach for the prevention and treatment of genital myiasis of Bactrian camels, which will result in positive advances for the clinical treatment of genital myiasis.

In this study, the bacterial phyla with the highest abundance identified in the two groups of Bactrian camel vaginal samples were Firmicutes, Proteobacteria, Fusobacterium and Bacteroides. These phyla are representative of the most common phyla found in many environments, especially in host-microbiome relationships. Previous studies have shown that the proportions and relative abundances of these taxa are related to changes in host physiology [22]. Therefore, when we performed ANOSIM on the samples, we found that even if there were differences among several individuals in the same group, the difference was obviously smaller than the registered between the groups.

Due to its residence in a natural channel, the vaginal flora is susceptible to environmental microbes. The increase in the diversity and richness of the bacterial community in the vagina of the diseased camels can be explained by the fact that the vulva is affected by fly maggots, which causes swelling and deformation such that the channel cannot be completely closed, allowing a large number of external bacteria to enter the vagina. However, the taxonomic composition analysis of Bactrian camels showed that there were no significant differences in the overall structure of the vaginal flora, indicating that the vaginal microecology of Bactrian camels had certain stability. In addition, immunomodulatory symbionts induce specific self-targeted responses that indirectly regulate immune responses to surrounding microorganisms [23]. Thus, the key role of microbial flora in maintaining homeostasis in the vaginal environment has been demonstrated [24], and the vaginal-associated microbiota may significantly affect the vaginal mucosal regulation of Bactrian camels. For example, the flora on the vaginal mucosa reduces the colonization of pathogenic bacteria by competing for living space and nutrients together with the production of short-chain fatty acids, bacteriocins, and reactive oxygen species to inhibit or kill pathogenic bacteria [25]. When the larvae of *Wohlfahrtia magnifica* invade the vagina of Bactrian camels, the external environment of microorganisms enters the vagina of the diseased camel, and vaginal pathogenic bacteria stimulate the mucosal immune system. For example, inflammation strengthens the clearance of pathogenic bacteria and reduces the possibility of pyogenic disease. Therefore, the vaginal mucosal immune system is able to identify beneficial microorganisms and harmful microorganisms, and pathogens are eliminated by the body's clearance immune response, while commensal bacteria remain safe [26].

The chemical nature of the vaginal mucosal niche drives the composition of symbiotic microorganisms with unknown microbial roles and host factors that lead to differences in its microecological composition and strain levels [27]. The acid-producing genera of Bactrian vaginal flora in the illness group were significantly enriched, such as Lactobacillus, Edwardsiella, Oribacterium, Parvimonas, Propionicimonas, and Sporomusa. This result is consistent with the low pH found, suggesting that maintaining a low vaginal pH prevents the colonization of pathogenic microorganisms and has a positive impact on the body resistance to pathogen invasion [28–33]. Studies have shown that the production of lactic acid and other antimicrobial metabolites by the vaginal microflora prevents endogenous opportunistic bacterial proliferation and immunomodulation [34, 35]. Lactobacillus is an important probiotic in the reproductive tract of female animals and can convert lactose and other sugars into lactic acid, which can prevent infection and reduce the risk of inflammation [36–38]. Lactobacillus also plays a role in accelerating the healing of tissue wounds [39].

## Conclusions

Through high-throughput sequencing of the vaginal flora of Bactrian camels, the diversity of the vaginal microbiota was revealed, and it has been demonstrated that genital myasis affects its composition. This study lay the basis for future research and proposed a new idea for the prevention and treatment of camel vaginal myiasis.

## Methods

### Experimental design and sampling

All female Bactrian camels involved in this study were part of a Bactrian camel herd registered with the College of Veterinary Medicine Inner Mongolia Agricultural University. All experimental procedures were approved by the Animal Protection and Use Committee of Inner Mongolia Agricultural University and strictly followed animal welfare and ethical guidelines [40].

According to the farming standards, all 23 Bactrian camels, including 10 suffering from genital myiasis and 13 healthy camels, were mature female, 8-years old animals. In addition, the camels studied were free-ranged and had no supplementary feeding except drinking water; additional findings were as follows: there was no history of vaginal drug release within one year; no oestrus or pregnancy for one month; and no antibiotics or antifungal drugs were used systematically within one month.

Routine sterile operations were used before each sampling and strictly followed. In addition, the procedural steps strictly ensured an aseptic opening of the female camel’s vagina and swabbing 5 times along the vaginal wall to collect the vaginal secretions. Then, the swabs were quickly placed in sterile 5 ml cryotubes. Finally, the samples were labelled and quickly stored in liquid nitrogen or at -80 °C and used to extract the 16S rRNA gene. Shortly afterwards, the pH of each sample was measured using an UltraBasic pH metre (Denver Instruments, Arvada, CO, United States).

### Bacterial DNA isolation

The thawed sample was centrifuged at 10,000 rpm for 10 min to collect bacterial cells, and the supernatant was discarded. The total DNA of the sample was extracted using a vaginal swab genomic DNA kit (Qiagen QIAamp DNA Mini Kit), and the specific steps were performed according to the instructions. The DNA was extracted and stored at -20 °C. The extracted DNA quality was evaluated by 0.8% agarose gel electrophoresis, and the DNA was quantified with an ultraviolet spectrophotometer.

### Sequencing of 16S rRNA

In combination with the fluorescence quantification results, each sample was mixed in a corresponding ratio according to the sequencing amount requirement of each sample. The processed samples were sent to Beijing WEISHENGTAI Co., Ltd. for paired-end 2 × 300 bp sequencing with the Illumina HiSeq 2000 platform.

### Statistical analysis

Basic statistical analysis was performed using SPSS statistics 20.0 statistical analysis software. Two pairs of comparisons of the measured data were performed, in accordance with the normal distribution. Two independent samples tests were performed, and *P* < 0.05 was therefore considered statistically significant.

Beta diversity was analyzed to examine differences in microbial communities between samples. Using an OTU-centric approach, (Principal Co-ordinates Analysis)PCoA matrices were employed using weighted and unweighted UniFrac distance matrices to compare the phylogenetic divergence among the OTUs between diseased camel samples and healthy camel vaginal samples.The visualization tool GraPhlAn [18] was used to build a hierarchical tree of the composition of the sample population at each classification level. Each classification unit was distinguished by different colours, and their distribution in abundance was also reflected by the node size.

Using mothur software, the statistical algorithm Metastats (http://metastats.cbcb.umd.edu/)was used. We were able to determine the overall classification level of all classification units in the sample population. The difference of sequence quantity (i.e., absolute abundance) between each taxon at the phylum and genus levels was analyzed and compared (pairwise).

## Supplementary Information

**Additional file 1.**

**Additional file 2.**

**Additional file 3.**

**Additional file 4.**

**Additional file 5.**

**Additional file 6.**

**Additional file 7.**

## Data Availability

We have submitted raw data through supplementary materials.

## Abbreviations

OUT: Operational Taxonomic Unit
ANOSIM: Analysis of similarities
PCoA: Principal Co-ordinates Analysis
