# Supplementary material for: The effects of genital myiasis on the diversity of the vaginal microbiota in female Bactrian camels
Source: BMC Vet Res. 2022 Mar 5;18:87. doi: 10.1186/s12917-022-03189-5 (PMC8897907; doi:10.1186/s12917-022-03189-5)
Supplement: Supplementary file 2 — Additional file 2. [file 12917_2022_3189_MOESM2_ESM.docx]

# 1、项目信息

**样本PCR扩增报告**

| 订单编号 | SP17090510; | 合同编号 | ME201707251001 |
| --- | --- | --- | --- |
| 项目名称 | 内蒙古农业大学-23个样品细菌多样性分析; | 客户单位 | 内蒙古农业大学; |
| 客户姓名 | 支立康; | 销售代表 | 黄腾杰 |
| 正式扩增人 | 高建礼; | 报告审核员 | 叶诞一 |
| 收样日期 | 2017-09-05; | 扩增完成日期 | 2017-09-09; |

# 2、扩增仪器及试剂

| **仪器/耗材** | **作用** | **品牌** | **型号** |
| --- | --- | --- | --- |
| PCR扩增仪 | 目标片段扩增 | ABI | 2720 |
| 酶标仪 | 荧光定量设备 | BioTek | FLX800T |
| 电泳仪 | 琼脂糖电泳设备 | 北京六一 | DYY-6C |
| 凝胶成像系统 | 凝胶成像设备 | 北京百晶 | BG-gdsAUTO(130) |
| Q5® High-Fidelity DNA Polymerase | 扩增聚合酶 | NEB | M0491L |
| Quant-iT PicoGreen dsDNA Assay Kit | 荧光定量染料 | Invitrogen | P7589 |
| Agarose | 琼脂糖凝胶试剂 | Invitrogen | 75510-019 |
| Marker | 核酸分子量大小的衡量标准 | Takara | DL2000 |
| TAE | 琼脂糖凝胶电泳缓冲液 | Invitrogen | AM9870 |

**3、样品扩增结果**

- 1. **引物信息**

| **引物名称** | **前引物序列** | **后引物序列** |
| --- | --- | --- |
| 338F/806R | ACTCCTACGGGAGGCAGCA | GGACTACHVGGGTWTCTAAT |

- 1. **扩增结果**

| **样品编号** | **样品名称** | **引物名称** | **测序平台** | **前引物编号** | **前引物barcode** | **扩增是否成功** | **扩增片段大小bp** | **回收浓度**  **（ng/ul）** | **扩增备注** |
| --- | --- | --- | --- | --- | --- | --- | --- | --- | --- |
| D7g6109 | A08 | 338F/806R | miseq | F15 | CACAAGT | 是 | 500 | 1.63 |  |
| D7g6102 | A01 | 338F/806R | miseq | F8 | TGACTGA | 是 | 500 | 8.28 |  |
| D7g6103 | A02 | 338F/806R | miseq | F9 | TGAGACT | 是 | 500 | 0.54 |  |
| D7g6104 | A03 | 338F/806R | miseq | F10 | TGTCAGT | 是 | 500 | 0.58 |  |
| D7g6105 | A04 | 338F/806R | miseq | F11 | TGTGTCA | 是 | 500 | 4.87 |  |
| D7g6106 | A05 | 338F/806R | miseq | F12 | CAACTGT | 是 | 500 | 0.55 |  |
| D7g6107 | A06 | 338F/806R | miseq | F13 | CATCAGA | 是 | 500 | 1.17 |  |
| D7g6108 | A07 | 338F/806R | miseq | F14 | CATGTCT | 是 | 500 | 1.57 |  |
| D7g6110 | A09 | 338F/806R | miseq | F16 | CACACTG | 是 | 500 | 0.56 |  |
| D7g6111 | A10 | 338F/806R | miseq | F17 | CACTTGA | 是 | 500 | 2.37 |  |
| D7g6112 | A11 | 338F/806R | miseq | F18 | CACTGAT | 是 | 500 | 0.76 |  |
| D7g6113 | A12 | 338F/806R | miseq | F19 | CAGATCA | 是 | 500 | 1.03 |  |
| D7g6114 | A13 | 338F/806R | miseq | F20 | CAGACAT | 是 | 500 | 2.24 |  |
| D7g6115 | B01 | 338F/806R | miseq | F21 | CAGAGTC | 是 | 500 | 0.53 |  |
| D7g6116 | B02 | 338F/806R | miseq | F22 | CAGTACT | 是 | 500 | 0.45 |  |

| D7g6117 | B03 | 338F/806R | miseq | F23 | CAGTCTA | 是 | 500 | 0.74 |  |
| --- | --- | --- | --- | --- | --- | --- | --- | --- | --- |
| D7g6118 | B04 | 338F/806R | miseq | F24 | CTAGACA | 是 | 500 | 0.69 |  |
| D7g6119 | B05 | 338F/806R | miseq | F25 | CTTGAGT | 是 | 500 | 1.04 |  |
| D7g6120 | B06 | 338F/806R | miseq | F26 | CTCACGA | 是 | 500 | 0.59 |  |
| D7g6121 | B07 | 338F/806R | miseq | F27 | CTCAGAC | 是 | 500 | 4.07 |  |
| D7g6122 | B08 | 338F/806R | miseq | F28 | CTCTCAG | 是 | 500 | 0.98 |  |
| D7g6123 | B09 | 338F/806R | miseq | F29 | CTCTGTA | 是 | 500 | 0.97 |  |
| D7g6124 | B10 | 338F/806R | miseq | F30 | CTGATGT | 是 | 500 | 2.78 |  |

## **扩增备注**：A: 完全无条带；B: 非常弥散的片段；C: 非特异性条带，无目标片段； **回收浓度说明：**回收浓度不低于0.5ng/ul即满足建库要求

**4、结果说明**

1. 样品统一稀释到20ng/ul，不足的直接使用原液，同时会根据样品的实际扩增情况对样本进行一定的稀释调整。
2. 扩增体系(25μL):5×reaction buffer 5μL, 5×GC buffer 5μL, dNTP（2.5mM） 2μL, Forwardprimer（10uM）1μL, Reverseprimer（10uM）1μL, DNA Template 2μL,ddH2O 8.75μL, Q5 DNA Polymerase 0.25μL(注：部分扩增比较困难的样品，实际扩增体系会进行部分微调)。
3. 扩增参数：Initial denaturation 98℃ 2min，Denaturation 98℃ 15s，Annealing 55℃ 30s，Extension 72℃ 30s，Final extension 72℃ 5min， 10℃ Hold.25-30Cycles(注：退火温度会进行调整以确定最佳温度，循环数会根据样品本身进行微调，以确保使用最少的循环扩增出合格的目标条带)。
4. 对于扩增不成功的样品，由于已经尝试多次扩增，建议客户提供备用的样品或是重新取样。
