# Supplementary material for: The effects of genital myiasis on the diversity of the vaginal microbiota in female Bactrian camels
Source: BMC Vet Res. 2022 Mar 5;18:87. doi: 10.1186/s12917-022-03189-5 (PMC8897907; doi:10.1186/s12917-022-03189-5)
Supplement: Supplementary file 3 — Additional file 3. [file 12917_2022_3189_MOESM3_ESM.docx]

**ARRIVE Guidelines**

During the whole experiment, we contact with animals during the taking samples,only. The sampling process does not cause any damage to the animals.

1.Study design

The sampling season of this experiment was summer, in order to avoid the impact of wind, sand and rain, the experiment was carried out indoors. When sampling, make sure the camel is properly secured and the person collecting the sample wears a mask.

Routine sterile operations were used before each sampling and strictly followed. In addition, the procedural steps to strictly ensure an aseptic open the female camel’s vaginal and rolled 5 times, along the vaginal wall to wipe the vaginal secretions. Then they were quickly placed in a sterile 5ml cryotube. Lastly, the sample was labeled and quickly stored in a liquid nitrogen or in a -80℃ refrigerator and used to extract the 16S rRNA gene. Shortly afterwards, the pH of each sample was measured using an UltraBasic pH meter (Denver Instruments Arvada CO United States).

2.Experimental procedures

(1) Bacterial DNA Isolation

The routine procedure is followed in this laboratory.

(2) Sequencing of 16S rRNA

In combination with the fluorescence quantification results, each sample was mixed in a corresponding ratio according to the sequencing amount requirement of each sample. The processed samples were sent to Beijing WEISHENGTAI Co. Ltd. for double-ended 2×300 bp sequencing (Paired-end) through the Illumina HiSeq 2000 platform.

(3) Sequence Read Processing and Statistical Analysis

Basic statistical analysis was performed using SPSS Statistics 20.0 statistical analysis software.

3. Experimental animals

The experimental animals were 13 healthy female Bactrian Camels and 10 sick female Bactrian Camels.
