# Supplementary material for: The effects of genital myiasis on the diversity of the vaginal microbiota in female Bactrian camels
Source: BMC Vet Res. 2022 Mar 5;18:87. doi: 10.1186/s12917-022-03189-5 (PMC8897907; doi:10.1186/s12917-022-03189-5)

# 1、项目信息

**菌群DNA样品检测报告**

| 订单编号 | SP17090510 | 合同编号 | ME201707251001 |
| --- | --- | --- | --- |
| 项目名称 | 内蒙古农业大学-23个样品细菌多样性 | 客户单位 | 内蒙古农业大学 |
| 客户姓名 | 支立康 | 销售代表 | 黄腾杰 |
| 质检员 | 郑林林 | 报告审核员 | 叶诞一 |
| 收样日期 | 2017-09-05 | 检测完成日期 | 2017-09-07 |

# 2、检测仪器及试剂

| **仪器/耗材** | **作用** | **品牌** | **型号** |
| --- | --- | --- | --- |
| Nanodrop | 紫外定量设备 | Thermo Scientific | NC2000 |
| 电泳仪 | 琼脂糖电泳设备 | 北京六一 | DYY-6C |
| 凝胶成像系统 | 凝胶成像设备 | 北京百晶 | BG-gdsAUTO(130) |
| Agarose | 琼脂糖凝胶试剂 | Invitrogen | 75510-019 |
| Marker | 核酸分子量大小的衡量标准 | Takara | DL15000或DL2000 |
| TAE | 琼脂糖凝胶电泳缓冲液 | Invitrogen | AM9870 |

## 3、样品检测结果

- 1. **紫外分光光度检测**

| **泳道** | **样品编号** | **样品名称** | **样品类型** | **处理类型** | **紫外浓度(ng/μl)** | **260/280值** | **260/230值** | **体积(μl)** | **总量(μg)** | **质检备注** |
| --- | --- | --- | --- | --- | --- | --- | --- | --- | --- | --- |
| 1 | D7g6102 | A01 | 原样 | DNA | 1.50 | 1.09 | 0.11 | 50.00 | 0.08 |  |
| 2 | D7g6103 | A02 | 原样 | DNA | 2.00 | 1.48 | 0.14 | 50.00 | 0.10 |  |
| 3 | D7g6104 | A03 | 原样 | DNA | 1.50 | 1.82 | 0.10 | 50.00 | 0.08 |  |
| 4 | D7g6105 | A04 | 原样 | DNA | 1.00 | 1.25 | 0.09 | 50.00 | 0.05 |  |
| 5 | D7g6106 | A05 | 原样 | DNA | 1.50 | 1.11 | 0.11 | 50.00 | 0.08 |  |
| 6 | D7g6107 | A06 | 原样 | DNA | 1.40 | 1.09 | 0.15 | 50.00 | 0.07 |  |
| 7 | D7g6108 | A07 | 原样 | DNA | 1.30 | 1.36 | 0.12 | 50.00 | 0.07 |  |
| 8 | D7g6109 | A08 | 原样 | DNA | 1.10 | 1.08 | 0.08 | 50.00 | 0.06 |  |
| 9 | D7g6110 | A09 | 原样 | DNA | 1.30 | 1.50 | 0.11 | 50.00 | 0.07 |  |
| 10 | D7g6111 | A10 | 原样 | DNA | 2.30 | 1.06 | 0.17 | 50.00 | 0.12 |  |
| 11 | D7g6112 | A11 | 原样 | DNA | 1.10 | 1.87 | 0.11 | 50.00 | 0.06 |  |
| 12 | D7g6113 | A12 | 原样 | DNA | 0.90 | 1.62 | 0.09 | 50.00 | 0.05 |  |
| 13 | D7g6114 | A13 | 原样 | DNA | 1.90 | 2.16 | 0.14 | 50.00 | 0.10 |  |
| 14 | D7g6115 | B01 | 原样 | DNA | 1.00 | 1.71 | 0.08 | 50.00 | 0.05 |  |
| 15 | D7g6116 | B02 | 原样 | DNA | 1.50 | 1.09 | 0.11 | 50.00 | 0.08 |  |
| 16 | D7g6117 | B03 | 原样 | DNA | 1.40 | 1.20 | 0.13 | 50.00 | 0.07 |  |
| 17 | D7g6118 | B04 | 原样 | DNA | 1.40 | 1.89 | 0.12 | 50.00 | 0.07 |  |
| 18 | D7g6119 | B05 | 原样 | DNA | 0.90 | 1.81 | 0.10 | 50.00 | 0.05 |  |

| 19 | D7g6120 | B06 | 原样 | DNA | 1.60 | 1.61 | 0.16 | 50.00 | 0.08 |  |
| --- | --- | --- | --- | --- | --- | --- | --- | --- | --- | --- |
| 20 | D7g6121 | B07 | 原样 | DNA | 2.40 | 1.04 | 0.26 | 50.00 | 0.12 |  |
| 21 | D7g6122 | B08 | 原样 | DNA | 1.70 | 1.14 | 0.16 | 50.00 | 0.09 |  |
| 22 | D7g6123 | B09 | 原样 | DNA | 0.80 | 1.06 | 0.10 | 50.00 | 0.04 |  |
| 23 | D7g6124 | B10 | 原样 | DNA | 1.40 | 1.61 | 0.15 | 50.00 | 0.07 |  |

**质检备注：**

a:样品有降解；b:样品有多条带；c：DNA或RNA污染；d：样品粘稠或浑浊；e：样品体积不足；f：样品纯度不够；g：样品浓度不够；

**结果说明备注：**

由于菌群组成谱项目本身的特殊性，上述检测结果不能作为一个定性的判断标准。最终结果以PCR鉴定为准，该检测结果仅作为一个后续PCR扩增的参考。如PCR扩增能够得到有效的目标条带，那么该样品即为合格（即完全符合后续的测序要求），反之则视为不合格样品。

- 1. **样品检测图**

| **Marker上样量** | **样品上样量** | **电泳时间** | **琼脂糖浓度** | **电压** | **电流** |
| --- | --- | --- | --- | --- | --- |
| 5μl | 5μl | 20min | 1.20% | 120V | 恒压，电流约80mA |


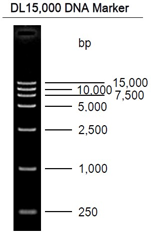

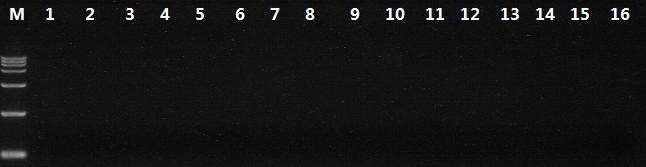

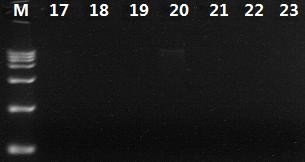

Supplement: Supplementary file 4 — Additional file 4. [file 12917_2022_3189_MOESM4_ESM.docx]
