# Supplementary material for: The effects of genital myiasis on the diversity of the vaginal microbiota in female Bactrian camels
Source: BMC Vet Res. 2022 Mar 5;18:87. doi: 10.1186/s12917-022-03189-5 (PMC8897907; doi:10.1186/s12917-022-03189-5)
Supplement: Supplementary file 5 — Additional file 5. [file 12917_2022_3189_MOESM5_ESM.zip › MPL201709200_16s_yy/Treat1/B02_arare/alpha_rarefaction_plots/rarefaction_plots.html]

 
 
 
   
   Rarefaction Curves 
 
td.data{font-size:10px;border-spacing:0px 10px;text-align:center;}
td.headers{font-size:12px;font-weight:bold;text-align:center;}
table{border-spacing:0px;}
.removed{display:none;}
.expands{cursor:pointer; cursor:hand;}
.child1 td:first-child{padding-left: 3px;}
 
 

function show_hide_category(checkobject){
    var imagetype=document.getElementById('imagetype').value;
    img=document.getElementById(checkobject.name.replace('_raw'+imagetype,'_ave'+imagetype))
    if (checkobject.checked==false){
        img.style.display='none';
    }else{
        img.style.display='';
    }
}

function reset_tree(){
    var category=document.getElementById('category').value;
    var metric=document.getElementById('metric').value;
    var old_all_categories=document.getElementById('all_categories');
    var imagetype=document.getElementById('imagetype').value;
    cat_list=old_all_categories.value.split('$#!')
    if (metric!='' && category != ''){
    for (var i=1, il=cat_list.length; i 

 
 
 
 
 
 
 
 
  
  Select a Metric:  
 
 
   
 chao1 
 observed_species 
 shannon 
 
 
    Select a Category:  
 
 
   
 BarcodeSequence 
 Description 
 LinkerPrimerSequence 
 SampleID 
 Treat1 
 
 
 
 
 
  

 
      Show Categories: 
     
           
         All 
         None 
         Invert 
     
      
 Legend   
  ▶     ■    AAAAA   
  ∟    ◆   A01   
  ▶     ■    AAAAC   
  ∟    ◆   A03   
  ▶     ■    AAAAG   
  ∟    ◆   A04   
  ▶     ■    AAAAT   
  ∟    ◆   A02   
  ▶     ■    AAACA   
  ∟    ◆   A09   
  ▶     ■    AAACC   
  ∟    ◆   A11   
  ▶     ■    AAACG   
  ∟    ◆   A12   
  ▶     ■    AAACT   
  ∟    ◆   A10   
  ▶     ■    AAAGA   
  ∟    ◆   A13   
  ▶     ■    AAAGC   
  ∟    ◆   B02   
  ▶     ■    AAAGG   
  ∟    ◆   B03   
  ▶     ■    AAAGT   
  ∟    ◆   B01   
  ▶     ■    AAATA   
  ∟    ◆   A05   
  ▶     ■    AAATC   
  ∟    ◆   A07   
  ▶     ■    AAATG   
  ∟    ◆   A08   
  ▶     ■    AAATT   
  ∟    ◆   A06   
  ▶     ■    AATAA   
  ∟    ◆   B04   
  ▶     ■    AATAC   
  ∟    ◆   B06   
  ▶     ■    AATAG   
  ∟    ◆   B07   
  ▶     ■    AATAT   
  ∟    ◆   B05   
  ▶     ■    AATTA   
  ∟    ◆   B08   
  ▶     ■    AATTC   
  ∟    ◆   B10   
  ▶     ■    AATTT   
  ∟    ◆   B09   
  ▶     ■    A01   
  ∟    ◆   A01   
  ▶     ■    A02   
  ∟    ◆   A02   
  ▶     ■    A03   
  ∟    ◆   A03   
  ▶     ■    A04   
  ∟    ◆   A04   
  ▶     ■    A05   
  ∟    ◆   A05   
  ▶     ■    A06   
  ∟    ◆   A06   
  ▶     ■    A07   
  ∟    ◆   A07   
  ▶     ■    A08   
  ∟    ◆   A08   
  ▶     ■    A09   
  ∟    ◆   A09   
  ▶     ■    A10   
  ∟    ◆   A10   
  ▶     ■    A11   
  ∟    ◆   A11   
  ▶     ■    A12   
  ∟    ◆   A12   
  ▶     ■    A13   
  ∟    ◆   A13   
  ▶     ■    B01   
  ∟    ◆   B01   
  ▶     ■    B02   
  ∟    ◆   B02   
  ▶     ■    B03   
  ∟    ◆   B03   
  ▶     ■    B04   
  ∟    ◆   B04   
  ▶     ■    B05   
  ∟    ◆   B05   
  ▶     ■    B06   
  ∟    ◆   B06   
  ▶     ■    B07   
  ∟    ◆   B07   
  ▶     ■    B08   
  ∟    ◆   B08   
  ▶     ■    B09   
  ∟    ◆   B09   
  ▶     ■    B10   
  ∟    ◆   B10   
  ▶     ■    ACTCCTACGGGAGGCAGCA   
  ∟    ◆   A01   
  ∟    ◆   A02   
  ∟    ◆   A03   
  ∟    ◆   A04   
  ∟    ◆   A05   
  ∟    ◆   A06   
  ∟    ◆   A07   
  ∟    ◆   A08   
  ∟    ◆   A09   
  ∟    ◆   A10   
  ∟    ◆   A11   
  ∟    ◆   A12   
  ∟    ◆   A13   
  ∟    ◆   B01   
  ∟    ◆   B02   
  ∟    ◆   B03   
  ∟    ◆   B04   
  ∟    ◆   B05   
  ∟    ◆   B06   
  ∟    ◆   B07   
  ∟    ◆   B08   
  ∟    ◆   B09   
  ∟    ◆   B10   
  ▶     ■    A01   
  ∟    ◆   A01   
  ▶     ■    A02   
  ∟    ◆   A02   
  ▶     ■    A03   
  ∟    ◆   A03   
  ▶     ■    A04   
  ∟    ◆   A04   
  ▶     ■    A05   
  ∟    ◆   A05   
  ▶     ■    A06   
  ∟    ◆   A06   
  ▶     ■    A07   
  ∟    ◆   A07   
  ▶     ■    A08   
  ∟    ◆   A08   
  ▶     ■    A09   
  ∟    ◆   A09   
  ▶     ■    A10   
  ∟    ◆   A10   
  ▶     ■    A11   
  ∟    ◆   A11   
  ▶     ■    A12   
  ∟    ◆   A12   
  ▶     ■    A13   
  ∟    ◆   A13   
  ▶     ■    B01   
  ∟    ◆   B01   
  ▶     ■    B02   
  ∟    ◆   B02   
  ▶     ■    B03   
  ∟    ◆   B03   
  ▶     ■    B04   
  ∟    ◆   B04   
  ▶     ■    B05   
  ∟    ◆   B05   
  ▶     ■    B06   
  ∟    ◆   B06   
  ▶     ■    B07   
  ∟    ◆   B07   
  ▶     ■    B08   
  ∟    ◆   B08   
  ▶     ■    B09   
  ∟    ◆   B09   
  ▶     ■    B10   
  ∟    ◆   B10   
  ▶     ■    illness   
  ∟    ◆   B01   
  ∟    ◆   B02   
  ∟    ◆   B03   
  ∟    ◆   B04   
  ∟    ◆   B05   
  ∟    ◆   B06   
  ∟    ◆   B07   
  ∟    ◆   B08   
  ∟    ◆   B09   
  ∟    ◆   B10   
  ▶     ■    normal   
  ∟    ◆   A01   
  ∟    ◆   A02   
  ∟    ◆   A03   
  ∟    ◆   A04   
  ∟    ◆   A05   
  ∟    ◆   A06   
  ∟    ◆   A07   
  ∟    ◆   A08   
  ∟    ◆   A09   
  ∟    ◆   A10   
  ∟    ◆   A11   
  ∟    ◆   A12   
  ∟    ◆   A13   
  ▶     ■    AAAAA   
  ∟    ◆   A01   
  ▶     ■    AAAAC   
  ∟    ◆   A03   
  ▶     ■    AAAAG   
  ∟    ◆   A04   
  ▶     ■    AAAAT   
  ∟    ◆   A02   
  ▶     ■    AAACA   
  ∟    ◆   A09   
  ▶     ■    AAACC   
  ∟    ◆   A11   
  ▶     ■    AAACG   
  ∟    ◆   A12   
  ▶     ■    AAACT   
  ∟    ◆   A10   
  ▶     ■    AAAGA   
  ∟    ◆   A13   
  ▶     ■    AAAGC   
  ∟    ◆   B02   
  ▶     ■    AAAGG   
  ∟    ◆   B03   
  ▶     ■    AAAGT   
  ∟    ◆   B01   
  ▶     ■    AAATA   
  ∟    ◆   A05   
  ▶     ■    AAATC   
  ∟    ◆   A07   
  ▶     ■    AAATG   
  ∟    ◆   A08   
  ▶     ■    AAATT   
  ∟    ◆   A06   
  ▶     ■    AATAA   
  ∟    ◆   B04   
  ▶     ■    AATAC   
  ∟    ◆   B06   
  ▶     ■    AATAG   
  ∟    ◆   B07   
  ▶     ■    AATAT   
  ∟    ◆   B05   
  ▶     ■    AATTA   
  ∟    ◆   B08   
  ▶     ■    AATTC   
  ∟    ◆   B10   
  ▶     ■    AATTT   
  ∟    ◆   B09   
  ▶     ■    A01   
  ∟    ◆   A01   
  ▶     ■    A02   
  ∟    ◆   A02   
  ▶     ■    A03   
  ∟    ◆   A03   
  ▶     ■    A04   
  ∟    ◆   A04   
  ▶     ■    A05   
  ∟    ◆   A05   
  ▶     ■    A06   
  ∟    ◆   A06   
  ▶     ■    A07   
  ∟    ◆   A07   
  ▶     ■    A08   
  ∟    ◆   A08   
  ▶     ■    A09   
  ∟    ◆   A09   
  ▶     ■    A10   
  ∟    ◆   A10   
  ▶     ■    A11   
  ∟    ◆   A11   
  ▶     ■    A12   
  ∟    ◆   A12   
  ▶     ■    A13   
  ∟    ◆   A13   
  ▶     ■    B01   
  ∟    ◆   B01   
  ▶     ■    B02   
  ∟    ◆   B02   
  ▶     ■    B03   
  ∟    ◆   B03   
  ▶     ■    B04   
  ∟    ◆   B04   
  ▶     ■    B05   
  ∟    ◆   B05   
  ▶     ■    B06   
  ∟    ◆   B06   
  ▶     ■    B07   
  ∟    ◆   B07   
  ▶     ■    B08   
  ∟    ◆   B08   
  ▶     ■    B09   
  ∟    ◆   B09   
  ▶     ■    B10   
  ∟    ◆   B10   
  ▶     ■    ACTCCTACGGGAGGCAGCA   
  ∟    ◆   A01   
  ∟    ◆   A02   
  ∟    ◆   A03   
  ∟    ◆   A04   
  ∟    ◆   A05   
  ∟    ◆   A06   
  ∟    ◆   A07   
  ∟    ◆   A08   
  ∟    ◆   A09   
  ∟    ◆   A10   
  ∟    ◆   A11   
  ∟    ◆   A12   
  ∟    ◆   A13   
  ∟    ◆   B01   
  ∟    ◆   B02   
  ∟    ◆   B03   
  ∟    ◆   B04   
  ∟    ◆   B05   
  ∟    ◆   B06   
  ∟    ◆   B07   
  ∟    ◆   B08   
  ∟    ◆   B09   
  ∟    ◆   B10   
  ▶     ■    A01   
  ∟    ◆   A01   
  ▶     ■    A02   
  ∟    ◆   A02   
  ▶     ■    A03   
  ∟    ◆   A03   
  ▶     ■    A04   
  ∟    ◆   A04   
  ▶     ■    A05   
  ∟    ◆   A05   
  ▶     ■    A06   
  ∟    ◆   A06   
  ▶     ■    A07   
  ∟    ◆   A07   
  ▶     ■    A08   
  ∟    ◆   A08   
  ▶     ■    A09   
  ∟    ◆   A09   
  ▶     ■    A10   
  ∟    ◆   A10   
  ▶     ■    A11   
  ∟    ◆   A11   
  ▶     ■    A12   
  ∟    ◆   A12   
  ▶     ■    A13   
  ∟    ◆   A13   
  ▶     ■    B01   
  ∟    ◆   B01   
  ▶     ■    B02   
  ∟    ◆   B02   
  ▶     ■    B03   
  ∟    ◆   B03   
  ▶     ■    B04   
  ∟    ◆   B04   
  ▶     ■    B05   
  ∟    ◆   B05   
  ▶     ■    B06   
  ∟    ◆   B06   
  ▶     ■    B07   
  ∟    ◆   B07   
  ▶     ■    B08   
  ∟    ◆   B08   
  ▶     ■    B09   
  ∟    ◆   B09   
  ▶     ■    B10   
  ∟    ◆   B10   
  ▶     ■    illness   
  ∟    ◆   B01   
  ∟    ◆   B02   
  ∟    ◆   B03   
  ∟    ◆   B04   
  ∟    ◆   B05   
  ∟    ◆   B06   
  ∟    ◆   B07   
  ∟    ◆   B08   
  ∟    ◆   B09   
  ∟    ◆   B10   
  ▶     ■    normal   
  ∟    ◆   A01   
  ∟    ◆   A02   
  ∟    ◆   A03   
  ∟    ◆   A04   
  ∟    ◆   A05   
  ∟    ◆   A06   
  ∟    ◆   A07   
  ∟    ◆   A08   
  ∟    ◆   A09   
  ∟    ◆   A10   
  ∟    ◆   A11   
  ∟    ◆   A12   
  ∟    ◆   A13   
  ▶     ■    AAAAA   
  ∟    ◆   A01   
  ▶     ■    AAAAC   
  ∟    ◆   A03   
  ▶     ■    AAAAG   
  ∟    ◆   A04   
  ▶     ■    AAAAT   
  ∟    ◆   A02   
  ▶     ■    AAACA   
  ∟    ◆   A09   
  ▶     ■    AAACC   
  ∟    ◆   A11   
  ▶     ■    AAACG   
  ∟    ◆   A12   
  ▶     ■    AAACT   
  ∟    ◆   A10   
  ▶     ■    AAAGA   
  ∟    ◆   A13   
  ▶     ■    AAAGC   
  ∟    ◆   B02   
  ▶     ■    AAAGG   
  ∟    ◆   B03   
  ▶     ■    AAAGT   
  ∟    ◆   B01   
  ▶     ■    AAATA   
  ∟    ◆   A05   
  ▶     ■    AAATC   
  ∟    ◆   A07   
  ▶     ■    AAATG   
  ∟    ◆   A08   
  ▶     ■    AAATT   
  ∟    ◆   A06   
  ▶     ■    AATAA   
  ∟    ◆   B04   
  ▶     ■    AATAC   
  ∟    ◆   B06   
  ▶     ■    AATAG   
  ∟    ◆   B07   
  ▶     ■    AATAT   
  ∟    ◆   B05   
  ▶     ■    AATTA   
  ∟    ◆   B08   
  ▶     ■    AATTC   
  ∟    ◆   B10   
  ▶     ■    AATTT   
  ∟    ◆   B09   
  ▶     ■    A01   
  ∟    ◆   A01   
  ▶     ■    A02   
  ∟    ◆   A02   
  ▶     ■    A03   
  ∟    ◆   A03   
  ▶     ■    A04   
  ∟    ◆   A04   
  ▶     ■    A05   
  ∟    ◆   A05   
  ▶     ■    A06   
  ∟    ◆   A06   
  ▶     ■    A07   
  ∟    ◆   A07   
  ▶     ■    A08   
  ∟    ◆   A08   
  ▶     ■    A09   
  ∟    ◆   A09   
  ▶     ■    A10   
  ∟    ◆   A10   
  ▶     ■    A11   
  ∟    ◆   A11   
  ▶     ■    A12   
  ∟    ◆   A12   
  ▶     ■    A13   
  ∟    ◆   A13   
  ▶     ■    B01   
  ∟    ◆   B01   
  ▶     ■    B02   
  ∟    ◆   B02   
  ▶     ■    B03   
  ∟    ◆   B03   
  ▶     ■    B04   
  ∟    ◆   B04   
  ▶     ■    B05   
  ∟    ◆   B05   
  ▶     ■    B06   
  ∟    ◆   B06   
  ▶     ■    B07   
  ∟    ◆   B07   
  ▶     ■    B08   
  ∟    ◆   B08   
  ▶     ■    B09   
  ∟    ◆   B09   
  ▶     ■    B10   
  ∟    ◆   B10   
  ▶     ■    ACTCCTACGGGAGGCAGCA   
  ∟    ◆   A01   
  ∟    ◆   A02   
  ∟    ◆   A03   
  ∟    ◆   A04   
  ∟    ◆   A05   
  ∟    ◆   A06   
  ∟    ◆   A07   
  ∟    ◆   A08   
  ∟    ◆   A09   
  ∟    ◆   A10   
  ∟    ◆   A11   
  ∟    ◆   A12   
  ∟    ◆   A13   
  ∟    ◆   B01   
  ∟    ◆   B02   
  ∟    ◆   B03   
  ∟    ◆   B04   
  ∟    ◆   B05   
  ∟    ◆   B06   
  ∟    ◆   B07   
  ∟    ◆   B08   
  ∟    ◆   B09   
  ∟    ◆   B10   
  ▶     ■    A01   
  ∟    ◆   A01   
  ▶     ■    A02   
  ∟    ◆   A02   
  ▶     ■    A03   
  ∟    ◆   A03   
  ▶     ■    A04   
  ∟    ◆   A04   
  ▶     ■    A05   
  ∟    ◆   A05   
  ▶     ■    A06   
  ∟    ◆   A06   
  ▶     ■    A07   
  ∟    ◆   A07   
  ▶     ■    A08   
  ∟    ◆   A08   
  ▶     ■    A09   
  ∟    ◆   A09   
  ▶     ■    A10   
  ∟    ◆   A10   
  ▶     ■    A11   
  ∟    ◆   A11   
  ▶     ■    A12   
  ∟    ◆   A12   
  ▶     ■    A13   
  ∟    ◆   A13   
  ▶     ■    B01   
  ∟    ◆   B01   
  ▶     ■    B02   
  ∟    ◆   B02   
  ▶     ■    B03   
  ∟    ◆   B03   
  ▶     ■    B04   
  ∟    ◆   B04   
  ▶     ■    B05   
  ∟    ◆   B05   
  ▶     ■    B06   
  ∟    ◆   B06   
  ▶     ■    B07   
  ∟    ◆   B07   
  ▶     ■    B08   
  ∟    ◆   B08   
  ▶     ■    B09   
  ∟    ◆   B09   
  ▶     ■    B10   
  ∟    ◆   B10   
  ▶     ■    illness   
  ∟    ◆   B01   
  ∟    ◆   B02   
  ∟    ◆   B03   
  ∟    ◆   B04   
  ∟    ◆   B05   
  ∟    ◆   B06   
  ∟    ◆   B07   
  ∟    ◆   B08   
  ∟    ◆   B09   
  ∟    ◆   B10   
  ▶     ■    normal   
  ∟    ◆   A01   
  ∟    ◆   A02   
  ∟    ◆   A03   
  ∟    ◆   A04   
  ∟    ◆   A05   
  ∟    ◆   A06   
  ∟    ◆   A07   
  ∟    ◆   A08   
  ∟    ◆   A09   
  ∟    ◆   A10   
  ∟    ◆   A11   
  ∟    ◆   A12   
  ∟    ◆   A13   
   
 
 
 If the lines for some categories do not extend all the way to the right end of the x-axis, that means that at least one of the samples in that category does not have that many samples. 
 
  
 
  Treat1  Seqs/Sample 
 chao1 Ave.  chao1 Err. 
 observed_species Ave.  observed_species Err. 
 shannon Ave.  shannon Err. 
 
 
 illness  10.0 
     21.955       6.162 
      7.935       0.960 
      2.852       0.247 
 
 illness  1553.0 
    311.972     158.141 
    188.480      89.663 
      5.374       0.920 
 
 illness  3096.0 
    377.564     179.400 
    256.065     125.819 
      5.455       0.958 
 
 illness  4639.0 
    405.466     185.364 
    299.220     146.645 
      5.489       0.972 
 
 illness  6182.0 
    426.392     193.667 
    327.880     161.229 
      5.500       0.979 
 
 illness  7725.0 
    440.960     198.644 
    350.510     170.653 
      5.513       0.987 
 
 illness  9268.0 
    450.041     200.403 
    369.515     177.518 
      5.520       0.984 
 
 illness  10811.0 
    458.500     200.126 
    383.115     181.787 
      5.527       0.987 
 
 illness  12354.0 
    464.641     204.688 
    394.605     186.635 
      5.531       0.991 
 
 illness  13897.0 
    468.589     205.558 
    405.170     189.585 
      5.532       0.990 
 
 illness  15440.0 
    475.920     204.218 
    414.125     191.418 
      5.536       0.992 
 
 illness  16983.0 
    477.745     206.674 
    421.395     192.869 
      5.538       0.993 
 
 illness  18526.0 
    480.842     210.236 
    427.925     195.982 
      5.542       0.994 
 
 illness  20069.0 
    484.611     209.711 
    433.960     196.723 
      5.540       0.994 
 
 illness  21612.0 
    486.149     211.334 
    439.075     198.434 
      5.545       0.995 
 
 illness  23155.0 
    488.099     211.535 
    443.835     199.780 
      5.545       0.994 
 
 illness  24698.0 
    492.189     213.868 
    448.800     201.062 
      5.548       0.994 
 
 illness  26241.0 
    491.118     213.052 
    452.360     202.430 
      5.547       0.995 
 
 illness  27784.0 
    491.526     212.534 
    455.330     202.668 
      5.548       0.997 
 
 illness  29327.0 
    493.751     215.319 
    459.175     204.176 
      5.549       0.996 
 
 illness  30870.0 
    492.872     215.706 
    461.860     204.793 
      5.550       0.997 
 
 illness  32413.0 
    493.764     214.796 
    464.135     205.058 
      5.549       0.997 
 
 illness  33956.0 
    494.552     217.162 
    466.970     206.203 
      5.551       0.996 
 
 illness  35499.0 
    494.328     216.107 
    468.680     206.721 
      5.550       0.997 
 
 illness  37042.0 
    494.612     216.603 
    471.070     207.502 
      5.552       0.997 
 
 illness  38585.0 
    494.105     216.872 
    472.485     208.090 
      5.552       0.997 
 
 illness  40128.0 
    494.915     217.397 
    474.450     208.474 
      5.552       0.997 
 
 illness  41671.0 
    495.029     217.798 
    476.100     209.295 
      5.552       0.997 
 
 illness  43214.0 
    495.242     218.466 
    477.570     209.675 
      5.553       0.998 
 
 illness  44757.0 
    495.078     218.766 
    478.725     210.261 
      5.553       0.998 
 
 illness  46300.0 
        nan         nan 
        nan         nan 
        nan         nan 
 
 normal  10.0 
     19.108       4.925 
      7.827       0.635 
      2.843       0.156 
 
 normal  1553.0 
    216.236      84.056 
    124.115      44.020 
      4.951       0.523 
 
 normal  3096.0 
    265.631     106.370 
    166.685      63.556 
      4.996       0.543 
 
 normal  4639.0 
    295.214     113.057 
    196.885      75.144 
      5.021       0.545 
 
 normal  6182.0 
    310.384     119.215 
    218.265      84.839 
      5.030       0.556 
 
 normal  7725.0 
    325.519     124.287 
    236.735      91.963 
      5.034       0.555 
 
 normal  9268.0 
    334.121     124.640 
    251.638      97.044 
      5.041       0.554 
 
 normal  10811.0 
    341.800     128.973 
    263.627     101.711 
      5.048       0.559 
 
 normal  12354.0 
    347.843     128.075 
    274.688     106.012 
      5.050       0.557 
 
 normal  13897.0 
    351.190     131.787 
    283.754     109.231 
      5.051       0.558 
 
 normal  15440.0 
    353.193     132.950 
    292.296     112.691 
      5.052       0.559 
 
 normal  16983.0 
    356.987     134.096 
    300.035     114.642 
      5.055       0.561 
 
 normal  18526.0 
    358.053     135.913 
    305.158     116.935 
      5.054       0.559 
 
 normal  20069.0 
    361.059     136.549 
    311.619     118.787 
      5.058       0.560 
 
 normal  21612.0 
    362.823     138.631 
    316.935     121.241 
      5.059       0.562 
 
 normal  23155.0 
    362.640     137.051 
    321.042     121.842 
      5.060       0.563 
 
 normal  24698.0 
    362.547     137.378 
    325.346     124.187 
      5.060       0.562 
 
 normal  26241.0 
    363.629     139.993 
    329.142     125.380 
      5.060       0.561 
 
 normal  27784.0 
    364.541     139.552 
    332.862     126.843 
      5.062       0.562 
 
 normal  29327.0 
    365.686     140.406 
    336.131     128.065 
      5.062       0.562 
 
 normal  30870.0 
    363.633     139.666 
    338.031     128.925 
      5.063       0.563 
 
 normal  32413.0 
    362.842     140.155 
    340.585     129.616 
      5.063       0.563 
 
 normal  33956.0 
    363.437     140.424 
    343.088     130.571 
      5.063       0.563 
 
 normal  35499.0 
    362.906     140.502 
    344.981     131.340 
      5.064       0.563 
 
 normal  37042.0 
    362.848     140.858 
    346.735     132.182 
      5.064       0.563 
 
 normal  38585.0 
    361.720     140.519 
    348.104     132.830 
      5.065       0.563 
 
 normal  40128.0 
    361.852     141.036 
    349.442     133.326 
      5.065       0.563 
 
 normal  41671.0 
    361.907     141.086 
    350.808     133.949 
      5.065       0.563 
 
 normal  43214.0 
    361.864     141.090 
    351.969     134.429 
      5.065       0.563 
 
 normal  44757.0 
    361.541     141.439 
    352.865     135.019 
      5.066       0.563 
 
 normal  46300.0 
        nan         nan 
        nan         nan 
        nan         nan 
 
  LinkerPrimerSequence  Seqs/Sample 
 chao1 Ave.  chao1 Err. 
 observed_species Ave.  observed_species Err. 
 shannon Ave.  shannon Err. 
 
 
 ACTCCTACGGGAGGCAGCA  10.0 
     20.346       5.675 
      7.874       0.795 
      2.847       0.201 
 
 ACTCCTACGGGAGGCAGCA  1553.0 
    257.860     130.840 
    152.100      74.891 
      5.135       0.753 
 
 ACTCCTACGGGAGGCAGCA  3096.0 
    314.298     153.191 
    205.546     105.495 
      5.195       0.786 
 
 ACTCCTACGGGAGGCAGCA  4639.0 
    343.149     158.590 
    241.378     122.943 
      5.225       0.795 
 
 ACTCCTACGGGAGGCAGCA  6182.0 
    360.822     166.276 
    265.924     135.362 
      5.234       0.804 
 
 ACTCCTACGGGAGGCAGCA  7725.0 
    375.710     170.770 
    286.202     143.608 
      5.242       0.809 
 
 ACTCCTACGGGAGGCAGCA  9268.0 
    384.521     171.884 
    302.889     149.795 
      5.249       0.807 
 
 ACTCCTACGGGAGGCAGCA  10811.0 
    392.539     173.672 
    315.578     154.026 
      5.256       0.811 
 
 ACTCCTACGGGAGGCAGCA  12354.0 
    398.625     175.613 
    326.826     158.211 
      5.259       0.812 
 
 ACTCCTACGGGAGGCAGCA  13897.0 
    402.233     177.694 
    336.543     161.226 
      5.260       0.812 
 
 ACTCCTACGGGAGGCAGCA  15440.0 
    406.553     178.395 
    345.265     163.573 
      5.263       0.814 
 
 ACTCCTACGGGAGGCAGCA  16983.0 
    409.490     179.774 
    352.800     164.988 
      5.265       0.815 
 
 ACTCCTACGGGAGGCAGCA  18526.0 
    411.440     182.656 
    358.535     167.726 
      5.266       0.815 
 
 ACTCCTACGGGAGGCAGCA  20069.0 
    414.777     182.788 
    364.811     168.759 
      5.268       0.815 
 
 ACTCCTACGGGAGGCAGCA  21612.0 
    416.443     184.441 
    370.039     170.571 
      5.270       0.817 
 
 ACTCCTACGGGAGGCAGCA  23155.0 
    417.187     184.227 
    374.430     171.608 
      5.271       0.816 
 
 ACTCCTACGGGAGGCAGCA  24698.0 
    418.913     186.237 
    379.022     173.318 
      5.272       0.817 
 
 ACTCCTACGGGAGGCAGCA  26241.0 
    419.059     186.565 
    382.715     174.450 
      5.272       0.817 
 
 ACTCCTACGGGAGGCAGCA  27784.0 
    419.752     186.037 
    386.109     175.038 
      5.273       0.818 
 
 ACTCCTACGGGAGGCAGCA  29327.0 
    421.367     187.964 
    389.628     176.396 
      5.274       0.818 
 
 ACTCCTACGGGAGGCAGCA  30870.0 
    419.824     188.043 
    391.870     177.195 
      5.274       0.818 
 
 ACTCCTACGGGAGGCAGCA  32413.0 
    419.765     188.082 
    394.302     177.565 
      5.274       0.818 
 
 ACTCCTACGGGAGGCAGCA  33956.0 
    420.444     189.405 
    396.950     178.590 
      5.275       0.818 
 
 ACTCCTACGGGAGGCAGCA  35499.0 
    420.046     188.964 
    398.763     179.138 
      5.275       0.818 
 
 ACTCCTACGGGAGGCAGCA  37042.0 
    420.137     189.419 
    400.793     179.986 
      5.276       0.818 
 
 ACTCCTACGGGAGGCAGCA  38585.0 
    419.279     189.518 
    402.183     180.558 
      5.276       0.819 
 
 ACTCCTACGGGAGGCAGCA  40128.0 
    419.706     190.111 
    403.793     181.063 
      5.277       0.819 
 
 ACTCCTACGGGAGGCAGCA  41671.0 
    419.786     190.342 
    405.283     181.781 
      5.277       0.819 
 
 ACTCCTACGGGAGGCAGCA  43214.0 
    419.854     190.721 
    406.578     182.224 
      5.277       0.819 
 
 ACTCCTACGGGAGGCAGCA  44757.0 
    419.600     191.043 
    407.587     182.807 
      5.277       0.819 
 
 ACTCCTACGGGAGGCAGCA  46300.0 
        nan         nan 
        nan         nan 
        nan         nan 
 
  BarcodeSequence  Seqs/Sample 
 chao1 Ave.  chao1 Err. 
 observed_species Ave.  observed_species Err. 
 shannon Ave.  shannon Err. 
 
 
 AAAAA  10.0 
     13.460         nan 
      7.200         nan 
      2.710         nan 
 
 AAAAA  1553.0 
    143.566         nan 
     83.500         nan 
      4.309         nan 
 
 AAAAA  3096.0 
    183.101         nan 
    111.200         nan 
      4.320         nan 
 
 AAAAA  4639.0 
    219.139         nan 
    134.050         nan 
      4.350         nan 
 
 AAAAA  6182.0 
    231.738         nan 
    151.150         nan 
      4.359         nan 
 
 AAAAA  7725.0 
    241.876         nan 
    161.150         nan 
      4.351         nan 
 
 AAAAA  9268.0 
    246.748         nan 
    174.500         nan 
      4.360         nan 
 
 AAAAA  10811.0 
    256.697         nan 
    184.550         nan 
      4.366         nan 
 
 AAAAA  12354.0 
    269.593         nan 
    194.600         nan 
      4.369         nan 
 
 AAAAA  13897.0 
    266.605         nan 
    199.500         nan 
      4.364         nan 
 
 AAAAA  15440.0 
    266.184         nan 
    205.250         nan 
      4.366         nan 
 
 AAAAA  16983.0 
    273.905         nan 
    215.750         nan 
      4.372         nan 
 
 AAAAA  18526.0 
    265.761         nan 
    218.600         nan 
      4.376         nan 
 
 AAAAA  20069.0 
    271.658         nan 
    224.900         nan 
      4.375         nan 
 
 AAAAA  21612.0 
    267.841         nan 
    228.900         nan 
      4.376         nan 
 
 AAAAA  23155.0 
    271.397         nan 
    232.900         nan 
      4.375         nan 
 
 AAAAA  24698.0 
    272.454         nan 
    236.100         nan 
      4.374         nan 
 
 AAAAA  26241.0 
    275.571         nan 
    240.750         nan 
      4.376         nan 
 
 AAAAA  27784.0 
    275.992         nan 
    245.050         nan 
      4.379         nan 
 
 AAAAA  29327.0 
    274.484         nan 
    246.700         nan 
      4.379         nan 
 
 AAAAA  30870.0 
    269.618         nan 
    247.150         nan 
      4.377         nan 
 
 AAAAA  32413.0 
    266.919         nan 
    250.500         nan 
      4.377         nan 
 
 AAAAA  33956.0 
    267.089         nan 
    252.350         nan 
      4.378         nan 
 
 AAAAA  35499.0 
    265.820         nan 
    254.000         nan 
      4.378         nan 
 
 AAAAA  37042.0 
    265.929         nan 
    255.800         nan 
      4.380         nan 
 
 AAAAA  38585.0 
    264.324         nan 
    256.750         nan 
      4.378         nan 
 
 AAAAA  40128.0 
    263.007         nan 
    258.000         nan 
      4.380         nan 
 
 AAAAA  41671.0 
    263.381         nan 
    259.250         nan 
      4.380         nan 
 
 AAAAA  43214.0 
    263.345         nan 
    260.250         nan 
      4.380         nan 
 
 AAAAA  44757.0 
    262.140         nan 
    260.650         nan 
      4.381         nan 
 
 AAAAA  46300.0 
        nan         nan 
        nan         nan 
        nan         nan 
 
 AAAAC  10.0 
     23.225         nan 
      8.600         nan 
      3.028         nan 
 
 AAAAC  1553.0 
    354.040         nan 
    208.000         nan 
      5.726         nan 
 
 AAAAC  3096.0 
    404.240         nan 
    281.600         nan 
      5.787         nan 
 
 AAAAC  4639.0 
    415.803         nan 
    324.300         nan 
      5.841         nan 
 
 AAAAC  6182.0 
    426.677         nan 
    351.700         nan 
      5.865         nan 
 
 AAAAC  7725.0 
    439.880         nan 
    373.000         nan 
      5.854         nan 
 
 AAAAC  9268.0 
    452.576         nan 
    389.000         nan 
      5.862         nan 
 
 AAAAC  10811.0 
    454.955         nan 
    401.000         nan 
      5.882         nan 
 
 AAAAC  12354.0 
    462.824         nan 
    413.200         nan 
      5.872         nan 
 
 AAAAC  13897.0 
    471.267         nan 
    421.750         nan 
      5.870         nan 
 
 AAAAC  15440.0 
    468.377         nan 
    427.650         nan 
      5.875         nan 
 
 AAAAC  16983.0 
    464.227         nan 
    433.250         nan 
      5.888         nan 
 
 AAAAC  18526.0 
    470.228         nan 
    438.550         nan 
      5.881         nan 
 
 AAAAC  20069.0 
    477.009         nan 
    445.350         nan 
      5.889         nan 
 
 AAAAC  21612.0 
    478.131         nan 
    448.750         nan 
      5.891         nan 
 
 AAAAC  23155.0 
    472.750         nan 
    450.050         nan 
      5.893         nan 
 
 AAAAC  24698.0 
    477.940         nan 
    454.150         nan 
      5.890         nan 
 
 AAAAC  26241.0 
    479.128         nan 
    457.550         nan 
      5.888         nan 
 
 AAAAC  27784.0 
    476.269         nan 
    459.250         nan 
      5.895         nan 
 
 AAAAC  29327.0 
    483.202         nan 
    462.150         nan 
      5.897         nan 
 
 AAAAC  30870.0 
    479.549         nan 
    462.650         nan 
      5.898         nan 
 
 AAAAC  32413.0 
    478.138         nan 
    463.950         nan 
      5.896         nan 
 
 AAAAC  33956.0 
    478.809         nan 
    465.450         nan 
      5.897         nan 
 
 AAAAC  35499.0 
    480.029         nan 
    467.700         nan 
      5.895         nan 
 
 AAAAC  37042.0 
    480.141         nan 
    468.500         nan 
      5.896         nan 
 
 AAAAC  38585.0 
    481.266         nan 
    469.850         nan 
      5.898         nan 
 
 AAAAC  40128.0 
    481.598         nan 
    470.800         nan 
      5.900         nan 
 
 AAAAC  41671.0 
    481.240         nan 
    471.650         nan 
      5.898         nan 
 
 AAAAC  43214.0 
    481.833         nan 
    472.150         nan 
      5.898         nan 
 
 AAAAC  44757.0 
    481.720         nan 
    473.150         nan 
      5.898         nan 
 
 AAAAC  46300.0 
    482.762         nan 
    474.000         nan 
      5.899         nan 
 
 AAAAG  10.0 
     22.275         nan 
      8.000         nan 
      2.873         nan 
 
 AAAAG  1553.0 
    261.024         nan 
    162.900         nan 
      5.250         nan 
 
 AAAAG  3096.0 
    287.134         nan 
    213.350         nan 
      5.311         nan 
 
 AAAAG  4639.0 
    315.337         nan 
    244.000         nan 
      5.343         nan 
 
 AAAAG  6182.0 
    317.813         nan 
    262.850         nan 
      5.348         nan 
 
 AAAAG  7725.0 
    341.689         nan 
    284.450         nan 
      5.353         nan 
 
 AAAAG  9268.0 
    340.222         nan 
    293.050         nan 
      5.361         nan 
 
 AAAAG  10811.0 
    355.262         nan 
    305.100         nan 
      5.377         nan 
 
 AAAAG  12354.0 
    358.778         nan 
    314.700         nan 
      5.378         nan 
 
 AAAAG  13897.0 
    362.498         nan 
    321.100         nan 
      5.379         nan 
 
 AAAAG  15440.0 
    365.593         nan 
    327.250         nan 
      5.371         nan 
 
 AAAAG  16983.0 
    365.968         nan 
    332.000         nan 
      5.380         nan 
 
 AAAAG  18526.0 
    367.835         nan 
    335.400         nan 
      5.382         nan 
 
 AAAAG  20069.0 
    371.082         nan 
    339.800         nan 
      5.383         nan 
 
 AAAAG  21612.0 
    374.429         nan 
    344.950         nan 
      5.387         nan 
 
 AAAAG  23155.0 
    374.900         nan 
    347.350         nan 
      5.384         nan 
 
 AAAAG  24698.0 
    375.540         nan 
    350.100         nan 
      5.388         nan 
 
 AAAAG  26241.0 
    374.762         nan 
    352.450         nan 
      5.388         nan 
 
 AAAAG  27784.0 
    377.844         nan 
    356.150         nan 
      5.391         nan 
 
 AAAAG  29327.0 
    377.822         nan 
    358.650         nan 
      5.386         nan 
 
 AAAAG  30870.0 
    378.494         nan 
    359.050         nan 
      5.387         nan 
 
 AAAAG  32413.0 
    374.710         nan 
    361.050         nan 
      5.388         nan 
 
 AAAAG  33956.0 
    374.578         nan 
    362.250         nan 
      5.388         nan 
 
 AAAAG  35499.0 
    375.072         nan 
    363.500         nan 
      5.388         nan 
 
 AAAAG  37042.0 
    375.091         nan 
    364.000         nan 
      5.387         nan 
 
 AAAAG  38585.0 
    373.100         nan 
    365.500         nan 
      5.390         nan 
 
 AAAAG  40128.0 
    372.383         nan 
    366.050         nan 
      5.388         nan 
 
 AAAAG  41671.0 
    371.961         nan 
    367.450         nan 
      5.390         nan 
 
 AAAAG  43214.0 
    371.554         nan 
    367.800         nan 
      5.389         nan 
 
 AAAAG  44757.0 
    370.929         nan 
    368.500         nan 
      5.390         nan 
 
 AAAAG  46300.0 
    370.712         nan 
    369.000         nan 
      5.390         nan 
 
 AAAAT  10.0 
     16.800         nan 
      7.500         nan 
      2.768         nan 
 
 AAAAT  1553.0 
     98.090         nan 
     66.100         nan 
      4.538         nan 
 
 AAAAT  3096.0 
    103.836         nan 
     79.100         nan 
      4.560         nan 
 
 AAAAT  4639.0 
    126.607         nan 
     90.650         nan 
      4.577         nan 
 
 AAAAT  6182.0 
    135.829         nan 
     97.250         nan 
      4.572         nan 
 
 AAAAT  7725.0 
    138.571         nan 
    104.100         nan 
      4.575         nan 
 
 AAAAT  9268.0 
    153.311         nan 
    109.550         nan 
      4.588         nan 
 
 AAAAT  10811.0 
    147.420         nan 
    113.000         nan 
      4.581         nan 
 
 AAAAT  12354.0 
    152.059         nan 
    119.250         nan 
      4.583         nan 
 
 AAAAT  13897.0 
    150.117         nan 
    119.950         nan 
      4.582         nan 
 
 AAAAT  15440.0 
    151.888         nan 
    123.850         nan 
      4.578         nan 
 
 AAAAT  16983.0 
    153.484         nan 
    127.850         nan 
      4.583         nan 
 
 AAAAT  18526.0 
    152.399         nan 
    129.800         nan 
      4.582         nan 
 
 AAAAT  20069.0 
    155.152         nan 
    134.350         nan 
      4.589         nan 
 
 AAAAT  21612.0 
    152.857         nan 
    135.350         nan 
      4.588         nan 
 
 AAAAT  23155.0 
    154.124         nan 
    137.100         nan 
      4.588         nan 
 
 AAAAT  24698.0 
    151.876         nan 
    137.350         nan 
      4.584         nan 
 
 AAAAT  26241.0 
    151.355         nan 
    140.300         nan 
      4.586         nan 
 
 AAAAT  27784.0 
    152.822         nan 
    142.500         nan 
      4.589         nan 
 
 AAAAT  29327.0 
    151.477         nan 
    142.900         nan 
      4.589         nan 
 
 AAAAT  30870.0 
    152.283         nan 
    143.950         nan 
      4.591         nan 
 
 AAAAT  32413.0 
    150.937         nan 
    144.550         nan 
      4.589         nan 
 
 AAAAT  33956.0 
    151.101         nan 
    146.400         nan 
      4.588         nan 
 
 AAAAT  35499.0 
    151.072         nan 
    147.300         nan 
      4.589         nan 
 
 AAAAT  37042.0 
    150.620         nan 
    147.550         nan 
      4.588         nan 
 
 AAAAT  38585.0 
    149.167         nan 
    147.850         nan 
      4.588         nan 
 
 AAAAT  40128.0 
    149.790         nan 
    148.350         nan 
      4.588         nan 
 
 AAAAT  41671.0 
    149.351         nan 
    148.650         nan 
      4.588         nan 
 
 AAAAT  43214.0 
    149.050         nan 
    148.750         nan 
      4.588         nan 
 
 AAAAT  44757.0 
    149.065         nan 
    149.000         nan 
      4.589         nan 
 
 AAAAT  46300.0 
        nan         nan 
        nan         nan 
        nan         nan 
 
 AAACA  10.0 
     14.375         nan 
      7.350         nan 
      2.729         nan 
 
 AAACA  1553.0 
    178.007         nan 
    111.400         nan 
      4.803         nan 
 
 AAACA  3096.0 
    211.053         nan 
    145.250         nan 
      4.861         nan 
 
 AAACA  4639.0 
    234.992         nan 
    169.050         nan 
      4.875         nan 
 
 AAACA  6182.0 
    247.141         nan 
    188.700         nan 
      4.889         nan 
 
 AAACA  7725.0 
    247.287         nan 
    197.900         nan 
      4.881         nan 
 
 AAACA  9268.0 
    263.070         nan 
    211.500         nan 
      4.899         nan 
 
 AAACA  10811.0 
    270.399         nan 
    221.550         nan 
      4.911         nan 
 
 AAACA  12354.0 
    269.470         nan 
    226.850         nan 
      4.904         nan 
 
 AAACA  13897.0 
    269.818         nan 
    231.350         nan 
      4.901         nan 
 
 AAACA  15440.0 
    272.411         nan 
    238.400         nan 
      4.913         nan 
 
 AAACA  16983.0 
    274.316         nan 
    243.900         nan 
      4.906         nan 
 
 AAACA  18526.0 
    273.275         nan 
    247.000         nan 
      4.909         nan 
 
 AAACA  20069.0 
    277.406         nan 
    251.200         nan 
      4.912         nan 
 
 AAACA  21612.0 
    274.667         nan 
    253.300         nan 
      4.914         nan 
 
 AAACA  23155.0 
    273.951         nan 
    256.450         nan 
      4.917         nan 
 
 AAACA  24698.0 
    277.225         nan 
    259.650         nan 
      4.910         nan 
 
 AAACA  26241.0 
    275.256         nan 
    260.700         nan 
      4.909         nan 
 
 AAACA  27784.0 
    273.190         nan 
    263.200         nan 
      4.913         nan 
 
 AAACA  29327.0 
    273.793         nan 
    264.400         nan 
      4.916         nan 
 
 AAACA  30870.0 
    272.180         nan 
    265.350         nan 
      4.916         nan 
 
 AAACA  32413.0 
    272.900         nan 
    266.700         nan 
      4.915         nan 
 
 AAACA  33956.0 
    273.000         nan 
    268.000         nan 
      4.915         nan 
 
 AAACA  35499.0 
    272.846         nan 
    268.600         nan 
      4.915         nan 
 
 AAACA  37042.0 
    272.274         nan 
    269.400         nan 
      4.915         nan 
 
 AAACA  38585.0 
    271.689         nan 
    269.600         nan 
      4.916         nan 
 
 AAACA  40128.0 
    271.463         nan 
    270.450         nan 
      4.917         nan 
 
 AAACA  41671.0 
    271.322         nan 
    270.700         nan 
      4.917         nan 
 
 AAACA  43214.0 
    271.224         nan 
    270.900         nan 
      4.916         nan 
 
 AAACA  44757.0 
    271.056         nan 
    271.000         nan 
      4.917         nan 
 
 AAACA  46300.0 
    271.000         nan 
    271.000         nan 
      4.917         nan 
 
 AAACC  10.0 
     26.075         nan 
      8.700         nan 
      3.048         nan 
 
 AAACC  1553.0 
    337.551         nan 
    187.850         nan 
      5.741         nan 
 
 AAACC  3096.0 
    424.173         nan 
    261.600         nan 
      5.825         nan 
 
 AAACC  4639.0 
    445.386         nan 
    308.750         nan 
      5.836         nan 
 
 AAACC  6182.0 
    483.799         nan 
    350.300         nan 
      5.866         nan 
 
 AAACC  7725.0 
    495.701         nan 
    380.800         nan 
      5.880         nan 
 
 AAACC  9268.0 
    506.781         nan 
    400.100         nan 
      5.884         nan 
 
 AAACC  10811.0 
    519.652         nan 
    417.950         nan 
      5.889         nan 
 
 AAACC  12354.0 
    522.918         nan 
    437.200         nan 
      5.897         nan 
 
 AAACC  13897.0 
    530.804         nan 
    448.250         nan 
      5.893         nan 
 
 AAACC  15440.0 
    542.258         nan 
    465.300         nan 
      5.905         nan 
 
 AAACC  16983.0 
    541.336         nan 
    472.600         nan 
      5.902         nan 
 
 AAACC  18526.0 
    543.081         nan 
    480.750         nan 
      5.902         nan 
 
 AAACC  20069.0 
    545.462         nan 
    485.950         nan 
      5.907         nan 
 
 AAACC  21612.0 
    545.599         nan 
    493.950         nan 
      5.913         nan 
 
 AAACC  23155.0 
    543.960         nan 
    498.700         nan 
      5.912         nan 
 
 AAACC  24698.0 
    552.222         nan 
    507.800         nan 
      5.916         nan 
 
 AAACC  26241.0 
    551.697         nan 
    510.150         nan 
      5.912         nan 
 
 AAACC  27784.0 
    555.845         nan 
    517.450         nan 
      5.914         nan 
 
 AAACC  29327.0 
    558.501         nan 
    521.000         nan 
      5.918         nan 
 
 AAACC  30870.0 
    552.065         nan 
    521.950         nan 
      5.917         nan 
 
 AAACC  32413.0 
    556.008         nan 
    525.800         nan 
      5.918         nan 
 
 AAACC  33956.0 
    554.933         nan 
    528.600         nan 
      5.920         nan 
 
 AAACC  35499.0 
    554.504         nan 
    530.350         nan 
      5.920         nan 
 
 AAACC  37042.0 
    557.903         nan 
    534.600         nan 
      5.922         nan 
 
 AAACC  38585.0 
    554.788         nan 
    534.800         nan 
      5.921         nan 
 
 AAACC  40128.0 
    559.701         nan 
    538.350         nan 
      5.922         nan 
 
 AAACC  41671.0 
    558.218         nan 
    539.500         nan 
      5.923         nan 
 
 AAACC  43214.0 
    559.722         nan 
    540.750         nan 
      5.921         nan 
 
 AAACC  44757.0 
    560.901         nan 
    542.500         nan 
      5.922         nan 
 
 AAACC  46300.0 
    562.278         nan 
    544.000         nan 
      5.922         nan 
 
 AAACG  10.0 
     27.400         nan 
      8.550         nan 
      3.003         nan 
 
 AAACG  1553.0 
    289.482         nan 
    150.600         nan 
      5.293         nan 
 
 AAACG  3096.0 
    385.600         nan 
    212.200         nan 
      5.350         nan 
 
 AAACG  4639.0 
    441.285         nan 
    261.300         nan 
      5.380         nan 
 
 AAACG  6182.0 
    480.039         nan 
    300.400         nan 
      5.414         nan 
 
 AAACG  7725.0 
    478.046         nan 
    328.850         nan 
      5.414         nan 
 
 AAACG  9268.0 
    493.466         nan 
    355.450         nan 
      5.421         nan 
 
 AAACG  10811.0 
    519.243         nan 
    379.350         nan 
      5.430         nan 
 
 AAACG  12354.0 
    519.861         nan 
    395.250         nan 
      5.430         nan 
 
 AAACG  13897.0 
    527.763         nan 
    412.450         nan 
      5.442         nan 
 
 AAACG  15440.0 
    536.400         nan 
    427.200         nan 
      5.436         nan 
 
 AAACG  16983.0 
    546.130         nan 
    441.100         nan 
      5.445         nan 
 
 AAACG  18526.0 
    541.036         nan 
    447.600         nan 
      5.441         nan 
 
 AAACG  20069.0 
    548.679         nan 
    459.950         nan 
      5.443         nan 
 
 AAACG  21612.0 
    556.770         nan 
    470.900         nan 
      5.444         nan 
 
 AAACG  23155.0 
    557.073         nan 
    479.000         nan 
      5.453         nan 
 
 AAACG  24698.0 
    553.125         nan 
    484.100         nan 
      5.448         nan 
 
 AAACG  26241.0 
    556.776         nan 
    491.250         nan 
      5.446         nan 
 
 AAACG  27784.0 
    560.951         nan 
    498.200         nan 
      5.448         nan 
 
 AAACG  29327.0 
    561.770         nan 
    505.800         nan 
      5.450         nan 
 
 AAACG  30870.0 
    559.708         nan 
    509.400         nan 
      5.454         nan 
 
 AAACG  32413.0 
    559.658         nan 
    513.700         nan 
      5.452         nan 
 
 AAACG  33956.0 
    565.976         nan 
    519.350         nan 
      5.451         nan 
 
 AAACG  35499.0 
    560.043         nan 
    522.500         nan 
      5.453         nan 
 
 AAACG  37042.0 
    561.724         nan 
    525.750         nan 
      5.455         nan 
 
 AAACG  38585.0 
    559.869         nan 
    529.250         nan 
      5.455         nan 
 
 AAACG  40128.0 
    560.906         nan 
    531.050         nan 
      5.455         nan 
 
 AAACG  41671.0 
    563.545         nan 
    534.650         nan 
      5.455         nan 
 
 AAACG  43214.0 
    561.163         nan 
    536.750         nan 
      5.455         nan 
 
 AAACG  44757.0 
    560.077         nan 
    538.700         nan 
      5.456         nan 
 
 AAACG  46300.0 
        nan         nan 
        nan         nan 
        nan         nan 
 
 AAACT  10.0 
     22.200         nan 
      8.150         nan 
      2.919         nan 
 
 AAACT  1553.0 
    242.391         nan 
    117.750         nan 
      4.649         nan 
 
 AAACT  3096.0 
    313.905         nan 
    165.750         nan 
      4.699         nan 
 
 AAACT  4639.0 
    353.874         nan 
    199.900         nan 
      4.736         nan 
 
 AAACT  6182.0 
    369.505         nan 
    223.300         nan 
      4.737         nan 
 
 AAACT  7725.0 
    411.911         nan 
    250.600         nan 
      4.747         nan 
 
 AAACT  9268.0 
    418.491         nan 
    271.650         nan 
      4.764         nan 
 
 AAACT  10811.0 
    433.524         nan 
    289.050         nan 
      4.757         nan 
 
 AAACT  12354.0 
    437.387         nan 
    305.350         nan 
      4.768         nan 
 
 AAACT  13897.0 
    453.960         nan 
    320.150         nan 
      4.772         nan 
 
 AAACT  15440.0 
    451.358         nan 
    333.800         nan 
      4.775         nan 
 
 AAACT  16983.0 
    464.049         nan 
    345.550         nan 
      4.772         nan 
 
 AAACT  18526.0 
    481.031         nan 
    357.200         nan 
      4.774         nan 
 
 AAACT  20069.0 
    468.901         nan 
    363.100         nan 
      4.772         nan 
 
 AAACT  21612.0 
    482.856         nan 
    374.700         nan 
      4.776         nan 
 
 AAACT  23155.0 
    472.893         nan 
    380.600         nan 
      4.779         nan 
 
 AAACT  24698.0 
    474.441         nan 
    389.650         nan 
      4.780         nan 
 
 AAACT  26241.0 
    486.727         nan 
    397.500         nan 
      4.782         nan 
 
 AAACT  27784.0 
    483.101         nan 
    404.050         nan 
      4.782         nan 
 
 AAACT  29327.0 
    485.436         nan 
    409.650         nan 
      4.781         nan 
 
 AAACT  30870.0 
    486.520         nan 
    415.550         nan 
      4.779         nan 
 
 AAACT  32413.0 
    483.505         nan 
    420.300         nan 
      4.784         nan 
 
 AAACT  33956.0 
    483.169         nan 
    423.950         nan 
      4.787         nan 
 
 AAACT  35499.0 
    488.214         nan 
    429.950         nan 
      4.785         nan 
 
 AAACT  37042.0 
    486.604         nan 
    433.300         nan 
      4.786         nan 
 
 AAACT  38585.0 
    486.783         nan 
    437.850         nan 
      4.786         nan 
 
 AAACT  40128.0 
    487.146         nan 
    440.300         nan 
      4.786         nan 
 
 AAACT  41671.0 
    487.725         nan 
    443.500         nan 
      4.786         nan 
 
 AAACT  43214.0 
    488.502         nan 
    447.500         nan 
      4.786         nan 
 
 AAACT  44757.0 
    491.004         nan 
    450.400         nan 
      4.787         nan 
 
 AAACT  46300.0 
        nan         nan 
        nan         nan 
        nan         nan 
 
 AAAGA  10.0 
     15.175         nan 
      7.450         nan 
      2.764         nan 
 
 AAAGA  1553.0 
    119.208         nan 
     83.200         nan 
      4.842         nan 
 
 AAAGA  3096.0 
    149.651         nan 
    100.950         nan 
      4.849         nan 
 
 AAAGA  4639.0 
    177.379         nan 
    118.600         nan 
      4.881         nan 
 
 AAAGA  6182.0 
    178.103         nan 
    126.650         nan 
      4.864         nan 
 
 AAAGA  7725.0 
    193.800         nan 
    139.200         nan 
      4.875         nan 
 
 AAAGA  9268.0 
    201.914         nan 
    147.650         nan 
      4.877         nan 
 
 AAAGA  10811.0 
    204.416         nan 
    153.350         nan 
      4.881         nan 
 
 AAAGA  12354.0 
    224.370         nan 
    159.100         nan 
      4.883         nan 
 
 AAAGA  13897.0 
    222.277         nan 
    166.350         nan 
      4.883         nan 
 
 AAAGA  15440.0 
    223.952         nan 
    173.150         nan 
      4.887         nan 
 
 AAAGA  16983.0 
    219.613         nan 
    176.750         nan 
      4.883         nan 
 
 AAAGA  18526.0 
    221.748         nan 
    181.350         nan 
      4.887         nan 
 
 AAAGA  20069.0 
    227.671         nan 
    184.700         nan 
      4.888         nan 
 
 AAAGA  21612.0 
    230.448         nan 
    189.050         nan 
      4.885         nan 
 
 AAAGA  23155.0 
    234.783         nan 
    194.500         nan 
      4.889         nan 
 
 AAAGA  24698.0 
    227.339         nan 
    195.400         nan 
      4.892         nan 
 
 AAAGA  26241.0 
    226.276         nan 
    197.300         nan 
      4.887         nan 
 
 AAAGA  27784.0 
    228.496         nan 
    199.200         nan 
      4.888         nan 
 
 AAAGA  29327.0 
    232.204         nan 
    202.400         nan 
      4.887         nan 
 
 AAAGA  30870.0 
    231.194         nan 
    204.350         nan 
      4.889         nan 
 
 AAAGA  32413.0 
    230.697         nan 
    207.050         nan 
      4.889         nan 
 
 AAAGA  33956.0 
    231.539         nan 
    208.450         nan 
      4.887         nan 
 
 AAAGA  35499.0 
    229.884         nan 
    210.100         nan 
      4.891         nan 
 
 AAAGA  37042.0 
    230.315         nan 
    212.300         nan 
      4.890         nan 
 
 AAAGA  38585.0 
    231.225         nan 
    212.900         nan 
      4.890         nan 
 
 AAAGA  40128.0 
    230.419         nan 
    214.600         nan 
      4.892         nan 
 
 AAAGA  41671.0 
    232.358         nan 
    215.500         nan 
      4.889         nan 
 
 AAAGA  43214.0 
    233.125         nan 
    217.450         nan 
      4.891         nan 
 
 AAAGA  44757.0 
    230.639         nan 
    217.750         nan 
      4.890         nan 
 
 AAAGA  46300.0 
        nan         nan 
        nan         nan 
        nan         nan 
 
 AAAGC  10.0 
     19.760         nan 
      7.700         nan 
      2.799         nan 
 
 AAAGC  1553.0 
    258.999         nan 
    169.750         nan 
      5.166         nan 
 
 AAAGC  3096.0 
    290.576         nan 
    221.550         nan 
      5.224         nan 
 
 AAAGC  4639.0 
    308.618         nan 
    254.350         nan 
      5.290         nan 
 
 AAAGC  6182.0 
    314.501         nan 
    271.800         nan 
      5.278         nan 
 
 AAAGC  7725.0 
    323.094         nan 
    285.800         nan 
      5.289         nan 
 
 AAAGC  9268.0 
    329.045         nan 
    296.850         nan 
      5.290         nan 
 
 AAAGC  10811.0 
    335.897         nan 
    305.950         nan 
      5.297         nan 
 
 AAAGC  12354.0 
    336.000         nan 
    311.300         nan 
      5.305         nan 
 
 AAAGC  13897.0 
    333.997         nan 
    314.400         nan 
      5.299         nan 
 
 AAAGC  15440.0 
    337.622         nan 
    318.250         nan 
      5.297         nan 
 
 AAAGC  16983.0 
    338.451         nan 
    322.900         nan 
      5.301         nan 
 
 AAAGC  18526.0 
    337.802         nan 
    324.200         nan 
      5.311         nan 
 
 AAAGC  20069.0 
    338.792         nan 
    327.550         nan 
      5.306         nan 
 
 AAAGC  21612.0 
    343.051         nan 
    330.900         nan 
      5.306         nan 
 
 AAAGC  23155.0 
    340.797         nan 
    330.850         nan 
      5.310         nan 
 
 AAAGC  24698.0 
    340.784         nan 
    332.750         nan 
      5.310         nan 
 
 AAAGC  26241.0 
    341.632         nan 
    334.050         nan 
      5.308         nan 
 
 AAAGC  27784.0 
    340.574         nan 
    335.750         nan 
      5.311         nan 
 
 AAAGC  29327.0 
    339.114         nan 
    335.200         nan 
      5.312         nan 
 
 AAAGC  30870.0 
    339.532         nan 
    336.450         nan 
      5.312         nan 
 
 AAAGC  32413.0 
    340.679         nan 
    337.400         nan 
      5.308         nan 
 
 AAAGC  33956.0 
    338.699         nan 
    337.000         nan 
      5.311         nan 
 
 AAAGC  35499.0 
    339.387         nan 
    338.100         nan 
      5.312         nan 
 
 AAAGC  37042.0 
    339.660         nan 
    338.550         nan 
      5.314         nan 
 
 AAAGC  38585.0 
    338.814         nan 
    338.250         nan 
      5.313         nan 
 
 AAAGC  40128.0 
    339.152         nan 
    338.800         nan 
      5.315         nan 
 
 AAAGC  41671.0 
    338.949         nan 
    338.800         nan 
      5.313         nan 
 
 AAAGC  43214.0 
    339.081         nan 
    338.950         nan 
      5.314         nan 
 
 AAAGC  44757.0 
    338.968         nan 
    338.950         nan 
      5.314         nan 
 
 AAAGC  46300.0 
    339.000         nan 
    339.000         nan 
      5.313         nan 
 
 AAAGG  10.0 
     26.400         nan 
      8.850         nan 
      3.084         nan 
 
 AAAGG  1553.0 
    420.476         nan 
    287.150         nan 
      6.356         nan 
 
 AAAGG  3096.0 
    483.050         nan 
    379.200         nan 
      6.494         nan 
 
 AAAGG  4639.0 
    522.172         nan 
    428.550         nan 
      6.542         nan 
 
 AAAGG  6182.0 
    535.578         nan 
    458.350         nan 
      6.556         nan 
 
 AAAGG  7725.0 
    551.792         nan 
    482.150         nan 
      6.586         nan 
 
 AAAGG  9268.0 
    558.203         nan 
    496.900         nan 
      6.579         nan 
 
 AAAGG  10811.0 
    564.182         nan 
    513.800         nan 
      6.597         nan 
 
 AAAGG  12354.0 
    578.665         nan 
    522.350         nan 
      6.592         nan 
 
 AAAGG  13897.0 
    573.795         nan 
    529.500         nan 
      6.599         nan 
 
 AAAGG  15440.0 
    587.887         nan 
    539.500         nan 
      6.609         nan 
 
 AAAGG  16983.0 
    591.866         nan 
    545.800         nan 
      6.608         nan 
 
 AAAGG  18526.0 
    596.707         nan 
    552.300         nan 
      6.609         nan 
 
 AAAGG  20069.0 
    600.125         nan 
    556.100         nan 
      6.608         nan 
 
 AAAGG  21612.0 
    607.703         nan 
    561.650         nan 
      6.615         nan 
 
 AAAGG  23155.0 
    608.871         nan 
    565.650         nan 
      6.613         nan 
 
 AAAGG  24698.0 
    615.347         nan 
    570.300         nan 
      6.615         nan 
 
 AAAGG  26241.0 
    613.935         nan 
    573.350         nan 
      6.619         nan 
 
 AAAGG  27784.0 
    623.669         nan 
    577.300         nan 
      6.620         nan 
 
 AAAGG  29327.0 
    617.963         nan 
    577.700         nan 
      6.617         nan 
 
 AAAGG  30870.0 
    626.295         nan 
    583.550         nan 
      6.621         nan 
 
 AAAGG  32413.0 
    628.586         nan 
    585.800         nan 
      6.621         nan 
 
 AAAGG  33956.0 
    624.772         nan 
    587.700         nan 
      6.622         nan 
 
 AAAGG  35499.0 
    627.539         nan 
    589.200         nan 
      6.621         nan 
 
 AAAGG  37042.0 
    628.241         nan 
    591.950         nan 
      6.623         nan 
 
 AAAGG  38585.0 
    628.215         nan 
    593.950         nan 
      6.623         nan 
 
 AAAGG  40128.0 
    628.147         nan 
    595.400         nan 
      6.621         nan 
 
 AAAGG  41671.0 
    633.045         nan 
    598.250         nan 
      6.622         nan 
 
 AAAGG  43214.0 
    632.983         nan 
    600.050         nan 
      6.624         nan 
 
 AAAGG  44757.0 
    635.330         nan 
    601.750         nan 
      6.625         nan 
 
 AAAGG  46300.0 
    634.794         nan 
    603.000         nan 
      6.625         nan 
 
 AAAGT  10.0 
     25.650         nan 
      8.700         nan 
      3.054         nan 
 
 AAAGT  1553.0 
    323.150         nan 
    197.050         nan 
      5.997         nan 
 
 AAAGT  3096.0 
    376.215         nan 
    265.250         nan 
      6.078         nan 
 
 AAAGT  4639.0 
    397.841         nan 
    309.100         nan 
      6.127         nan 
 
 AAAGT  6182.0 
    404.897         nan 
    332.300         nan 
      6.137         nan 
 
 AAAGT  7725.0 
    423.863         nan 
    355.700         nan 
      6.143         nan 
 
 AAAGT  9268.0 
    432.382         nan 
    369.000         nan 
      6.148         nan 
 
 AAAGT  10811.0 
    440.977         nan 
    384.600         nan 
      6.165         nan 
 
 AAAGT  12354.0 
    436.616         nan 
    391.400         nan 
      6.170         nan 
 
 AAAGT  13897.0 
    443.201         nan 
    400.900         nan 
      6.172         nan 
 
 AAAGT  15440.0 
    443.941         nan 
    408.550         nan 
      6.170         nan 
 
 AAAGT  16983.0 
    446.594         nan 
    415.300         nan 
      6.175         nan 
 
 AAAGT  18526.0 
    447.682         nan 
    417.500         nan 
      6.175         nan 
 
 AAAGT  20069.0 
    449.022         nan 
    421.600         nan 
      6.174         nan 
 
 AAAGT  21612.0 
    446.604         nan 
    425.550         nan 
      6.182         nan 
 
 AAAGT  23155.0 
    453.224         nan 
    430.150         nan 
      6.176         nan 
 
 AAAGT  24698.0 
    452.140         nan 
    432.450         nan 
      6.179         nan 
 
 AAAGT  26241.0 
    448.967         nan 
    434.150         nan 
      6.182         nan 
 
 AAAGT  27784.0 
    449.349         nan 
    437.350         nan 
      6.183         nan 
 
 AAAGT  29327.0 
    449.162         nan 
    437.550         nan 
      6.184         nan 
 
 AAAGT  30870.0 
    446.542         nan 
    439.600         nan 
      6.182         nan 
 
 AAAGT  32413.0 
    447.231         nan 
    441.150         nan 
      6.182         nan 
 
 AAAGT  33956.0 
    448.170         nan 
    442.550         nan 
      6.184         nan 
 
 AAAGT  35499.0 
    447.152         nan 
    443.400         nan 
      6.184         nan 
 
 AAAGT  37042.0 
    446.676         nan 
    444.300         nan 
      6.184         nan 
 
 AAAGT  38585.0 
    446.536         nan 
    444.900         nan 
      6.186         nan 
 
 AAAGT  40128.0 
    446.865         nan 
    445.550         nan 
      6.185         nan 
 
 AAAGT  41671.0 
    446.448         nan 
    445.600         nan 
      6.184         nan 
 
 AAAGT  43214.0 
    446.293         nan 
    446.000         nan 
      6.186         nan 
 
 AAAGT  44757.0 
    446.094         nan 
    446.000         nan 
      6.185         nan 
 
 AAAGT  46300.0 
    446.000         nan 
    446.000         nan 
      6.186         nan 
 
 AAATA  10.0 
     10.067         nan 
      6.350         nan 
      2.453         nan 
 
 AAATA  1553.0 
    106.510         nan 
     58.900         nan 
      3.809         nan 
 
 AAATA  3096.0 
    118.318         nan 
     75.150         nan 
      3.813         nan 
 
 AAATA  4639.0 
    127.828         nan 
     88.100         nan 
      3.833         nan 
 
 AAATA  6182.0 
    133.552         nan 
     94.350         nan 
      3.820         nan 
 
 AAATA  7725.0 
    146.678         nan 
    104.950         nan 
      3.833         nan 
 
 AAATA  9268.0 
    146.871         nan 
    111.350         nan 
      3.835         nan 
 
 AAATA  10811.0 
    152.116         nan 
    117.750         nan 
      3.837         nan 
 
 AAATA  12354.0 
    153.553         nan 
    120.150         nan 
      3.841         nan 
 
 AAATA  13897.0 
    156.110         nan 
    127.650         nan 
      3.839         nan 
 
 AAATA  15440.0 
    158.781         nan 
    130.250         nan 
      3.838         nan 
 
 AAATA  16983.0 
    159.438         nan 
    133.550         nan 
      3.842         nan 
 
 AAATA  18526.0 
    159.567         nan 
    136.100         nan 
      3.839         nan 
 
 AAATA  20069.0 
    157.719         nan 
    139.200         nan 
      3.844         nan 
 
 AAATA  21612.0 
    157.939         nan 
    140.800         nan 
      3.841         nan 
 
 AAATA  23155.0 
    158.607         nan 
    143.400         nan 
      3.838         nan 
 
 AAATA  24698.0 
    162.023         nan 
    145.150         nan 
      3.844         nan 
 
 AAATA  26241.0 
    157.573         nan 
    146.950         nan 
      3.847         nan 
 
 AAATA  27784.0 
    158.990         nan 
    148.050         nan 
      3.845         nan 
 
 AAATA  29327.0 
    159.099         nan 
    149.700         nan 
      3.844         nan 
 
 AAATA  30870.0 
    158.129         nan 
    150.050         nan 
      3.844         nan 
 
 AAATA  32413.0 
    156.594         nan 
    151.300         nan 
      3.844         nan 
 
 AAATA  33956.0 
    157.779         nan 
    152.800         nan 
      3.845         nan 
 
 AAATA  35499.0 
    156.781         nan 
    153.350         nan 
      3.846         nan 
 
 AAATA  37042.0 
    155.625         nan 
    153.400         nan 
      3.845         nan 
 
 AAATA  38585.0 
    155.604         nan 
    153.950         nan 
      3.846         nan 
 
 AAATA  40128.0 
    155.410         nan 
    154.350         nan 
      3.846         nan 
 
 AAATA  41671.0 
    155.375         nan 
    154.800         nan 
      3.846         nan 
 
 AAATA  43214.0 
    155.254         nan 
    154.950         nan 
      3.846         nan 
 
 AAATA  44757.0 
    155.108         nan 
    155.000         nan 
      3.846         nan 
 
 AAATA  46300.0 
    155.000         nan 
    155.000         nan 
      3.846         nan 
 
 AAATC  10.0 
     18.250         nan 
      8.050         nan 
      2.911         nan 
 
 AAATC  1553.0 
    171.159         nan 
    106.150         nan 
      5.147         nan 
 
 AAATC  3096.0 
    222.244         nan 
    138.800         nan 
      5.205         nan 
 
 AAATC  4639.0 
    240.379         nan 
    160.250         nan 
      5.212         nan 
 
 AAATC  6182.0 
    276.849         nan 
    177.100         nan 
      5.217         nan 
 
 AAATC  7725.0 
    279.056         nan 
    189.900         nan 
      5.219         nan 
 
 AAATC  9268.0 
    297.130         nan 
    203.150         nan 
      5.217         nan 
 
 AAATC  10811.0 
    298.418         nan 
    211.550         nan 
      5.223         nan 
 
 AAATC  12354.0 
    301.960         nan 
    223.550         nan 
      5.230         nan 
 
 AAATC  13897.0 
    296.278         nan 
    231.150         nan 
      5.236         nan 
 
 AAATC  15440.0 
    301.236         nan 
    240.200         nan 
      5.230         nan 
 
 AAATC  16983.0 
    313.601         nan 
    249.300         nan 
      5.232         nan 
 
 AAATC  18526.0 
    311.381         nan 
    250.100         nan 
      5.230         nan 
 
 AAATC  20069.0 
    313.954         nan 
    258.850         nan 
      5.237         nan 
 
 AAATC  21612.0 
    311.018         nan 
    263.300         nan 
      5.234         nan 
 
 AAATC  23155.0 
    315.783         nan 
    270.150         nan 
      5.239         nan 
 
 AAATC  24698.0 
    312.240         nan 
    273.000         nan 
      5.237         nan 
 
 AAATC  26241.0 
    310.858         nan 
    276.700         nan 
      5.239         nan 
 
 AAATC  27784.0 
    315.036         nan 
    279.650         nan 
      5.238         nan 
 
 AAATC  29327.0 
    313.750         nan 
    284.000         nan 
      5.239         nan 
 
 AAATC  30870.0 
    310.224         nan 
    286.800         nan 
      5.237         nan 
 
 AAATC  32413.0 
    310.285         nan 
    289.500         nan 
      5.240         nan 
 
 AAATC  33956.0 
    311.160         nan 
    292.200         nan 
      5.236         nan 
 
 AAATC  35499.0 
    307.911         nan 
    293.350         nan 
      5.241         nan 
 
 AAATC  37042.0 
    308.393         nan 
    295.700         nan 
      5.241         nan 
 
 AAATC  38585.0 
    304.972         nan 
    296.450         nan 
      5.242         nan 
 
 AAATC  40128.0 
    305.507         nan 
    297.850         nan 
      5.241         nan 
 
 AAATC  41671.0 
    305.137         nan 
    299.400         nan 
      5.243         nan 
 
 AAATC  43214.0 
    304.780         nan 
    300.900         nan 
      5.242         nan 
 
 AAATC  44757.0 
    304.382         nan 
    301.650         nan 
      5.241         nan 
 
 AAATC  46300.0 
    303.364         nan 
    302.000         nan 
      5.242         nan 
 
 AAATG  10.0 
     20.875         nan 
      8.150         nan 
      2.929         nan 
 
 AAATG  1553.0 
    293.045         nan 
    138.950         nan 
      5.196         nan 
 
 AAATG  3096.0 
    377.470         nan 
    200.500         nan 
      5.244         nan 
 
 AAATG  4639.0 
    440.541         nan 
    249.450         nan 
      5.280         nan 
 
 AAATG  6182.0 
    445.577         nan 
    280.600         nan 
      5.297         nan 
 
 AAATG  7725.0 
    493.514         nan 
    311.350         nan 
      5.299         nan 
 
 AAATG  9268.0 
    496.078         nan 
    340.400         nan 
      5.308         nan 
 
 AAATG  10811.0 
    499.195         nan 
    358.850         nan 
      5.326         nan 
 
 AAATG  12354.0 
    508.754         nan 
    377.750         nan 
      5.322         nan 
 
 AAATG  13897.0 
    510.334         nan 
    393.200         nan 
      5.325         nan 
 
 AAATG  15440.0 
    512.669         nan 
    409.050         nan 
      5.333         nan 
 
 AAATG  16983.0 
    517.998         nan 
    422.100         nan 
      5.333         nan 
 
 AAATG  18526.0 
    520.362         nan 
    434.100         nan 
      5.334         nan 
 
 AAATG  20069.0 
    531.591         nan 
    447.550         nan 
      5.338         nan 
 
 AAATG  21612.0 
    529.702         nan 
    456.650         nan 
      5.338         nan 
 
 AAATG  23155.0 
    533.185         nan 
    461.250         nan 
      5.338         nan 
 
 AAATG  24698.0 
    524.230         nan 
    470.050         nan 
      5.342         nan 
 
 AAATG  26241.0 
    532.521         nan 
    477.900         nan 
      5.343         nan 
 
 AAATG  27784.0 
    528.560         nan 
    482.900         nan 
      5.343         nan 
 
 AAATG  29327.0 
    528.124         nan 
    486.500         nan 
      5.343         nan 
 
 AAATG  30870.0 
    525.610         nan 
    492.500         nan 
      5.344         nan 
 
 AAATG  32413.0 
    524.832         nan 
    495.050         nan 
      5.344         nan 
 
 AAATG  33956.0 
    523.966         nan 
    500.500         nan 
      5.348         nan 
 
 AAATG  35499.0 
    523.395         nan 
    503.000         nan 
      5.346         nan 
 
 AAATG  37042.0 
    520.582         nan 
    505.400         nan 
      5.348         nan 
 
 AAATG  38585.0 
    518.193         nan 
    507.000         nan 
      5.349         nan 
 
 AAATG  40128.0 
    515.406         nan 
    508.150         nan 
      5.350         nan 
 
 AAATG  41671.0 
    515.222         nan 
    509.900         nan 
      5.349         nan 
 
 AAATG  43214.0 
    515.186         nan 
    511.600         nan 
      5.349         nan 
 
 AAATG  44757.0 
    513.934         nan 
    512.350         nan 
      5.349         nan 
 
 AAATG  46300.0 
        nan         nan 
        nan         nan 
        nan         nan 
 
 AAATT  10.0 
     18.225         nan 
      7.700         nan 
      2.822         nan 
 
 AAATT  1553.0 
    216.990         nan 
    138.200         nan 
      5.063         nan 
 
 AAATT  3096.0 
    272.482         nan 
    181.450         nan 
      5.120         nan 
 
 AAATT  4639.0 
    299.225         nan 
    211.100         nan 
      5.130         nan 
 
 AAATT  6182.0 
    308.372         nan 
    233.100         nan 
      5.140         nan 
 
 AAATT  7725.0 
    323.732         nan 
    251.300         nan 
      5.156         nan 
 
 AAATT  9268.0 
    326.919         nan 
    263.950         nan 
      5.160         nan 
 
 AAATT  10811.0 
    332.098         nan 
    274.100         nan 
      5.162         nan 
 
 AAATT  12354.0 
    340.432         nan 
    284.000         nan 
      5.169         nan 
 
 AAATT  13897.0 
    347.642         nan 
    295.950         nan 
      5.171         nan 
 
 AAATT  15440.0 
    340.408         nan 
    298.500         nan 
      5.172         nan 
 
 AAATT  16983.0 
    346.765         nan 
    306.750         nan 
      5.176         nan 
 
 AAATT  18526.0 
    346.982         nan 
    310.500         nan 
      5.172         nan 
 
 AAATT  20069.0 
    347.487         nan 
    316.150         nan 
      5.181         nan 
 
 AAATT  21612.0 
    354.439         nan 
    319.550         nan 
      5.180         nan 
 
 AAATT  23155.0 
    350.907         nan 
    322.100         nan 
      5.177         nan 
 
 AAATT  24698.0 
    352.456         nan 
    327.000         nan 
      5.175         nan 
 
 AAATT  26241.0 
    348.673         nan 
    329.350         nan 
      5.181         nan 
 
 AAATT  27784.0 
    351.936         nan 
    331.550         nan 
      5.177         nan 
 
 AAATT  29327.0 
    354.258         nan 
    335.850         nan 
      5.180         nan 
 
 AAATT  30870.0 
    351.654         nan 
    335.650         nan 
      5.180         nan 
 
 AAATT  32413.0 
    351.768         nan 
    338.150         nan 
      5.180         nan 
 
 AAATT  33956.0 
    351.582         nan 
    339.850         nan 
      5.181         nan 
 
 AAATT  35499.0 
    352.211         nan 
    341.050         nan 
      5.184         nan 
 
 AAATT  37042.0 
    351.828         nan 
    341.850         nan 
      5.183         nan 
 
 AAATT  38585.0 
    351.376         nan 
    343.600         nan 
      5.184         nan 
 
 AAATT  40128.0 
    351.341         nan 
    344.450         nan 
      5.184         nan 
 
 AAATT  41671.0 
    349.960         nan 
    345.550         nan 
      5.183         nan 
 
 AAATT  43214.0 
    349.491         nan 
    345.850         nan 
      5.186         nan 
 
 AAATT  44757.0 
    349.074         nan 
    346.600         nan 
      5.185         nan 
 
 AAATT  46300.0 
    348.665         nan 
    346.950         nan 
      5.184         nan 
 
 AATAA  10.0 
     21.325         nan 
      7.950         nan 
      2.866         nan 
 
 AATAA  1553.0 
    236.280         nan 
    148.700         nan 
      5.079         nan 
 
 AATAA  3096.0 
    306.666         nan 
    196.000         nan 
      5.134         nan 
 
 AATAA  4639.0 
    322.072         nan 
    226.950         nan 
      5.154         nan 
 
 AATAA  6182.0 
    343.894         nan 
    254.250         nan 
      5.166         nan 
 
 AATAA  7725.0 
    359.265         nan 
    272.150         nan 
      5.164         nan 
 
 AATAA  9268.0 
    363.720         nan 
    288.150         nan 
      5.186         nan 
 
 AATAA  10811.0 
    373.656         nan 
    301.650         nan 
      5.183         nan 
 
 AATAA  12354.0 
    374.147         nan 
    309.650         nan 
      5.182         nan 
 
 AATAA  13897.0 
    380.587         nan 
    321.100         nan 
      5.191         nan 
 
 AATAA  15440.0 
    390.064         nan 
    329.800         nan 
      5.193         nan 
 
 AATAA  16983.0 
    388.978         nan 
    336.550         nan 
      5.190         nan 
 
 AATAA  18526.0 
    388.941         nan 
    341.450         nan 
      5.196         nan 
 
 AATAA  20069.0 
    391.786         nan 
    345.200         nan 
      5.194         nan 
 
 AATAA  21612.0 
    392.218         nan 
    351.250         nan 
      5.197         nan 
 
 AATAA  23155.0 
    400.442         nan 
    357.750         nan 
      5.196         nan 
 
 AATAA  24698.0 
    398.091         nan 
    361.300         nan 
      5.199         nan 
 
 AATAA  26241.0 
    400.787         nan 
    364.100         nan 
      5.200         nan 
 
 AATAA  27784.0 
    405.560         nan 
    367.850         nan 
      5.199         nan 
 
 AATAA  29327.0 
    407.521         nan 
    371.900         nan 
      5.200         nan 
 
 AATAA  30870.0 
    404.192         nan 
    374.950         nan 
      5.200         nan 
 
 AATAA  32413.0 
    399.209         nan 
    375.450         nan 
      5.202         nan 
 
 AATAA  33956.0 
    402.404         nan 
    378.900         nan 
      5.202         nan 
 
 AATAA  35499.0 
    403.400         nan 
    380.750         nan 
      5.203         nan 
 
 AATAA  37042.0 
    403.689         nan 
    381.350         nan 
      5.202         nan 
 
 AATAA  38585.0 
    403.622         nan 
    384.000         nan 
      5.203         nan 
 
 AATAA  40128.0 
    403.239         nan 
    386.150         nan 
      5.206         nan 
 
 AATAA  41671.0 
    404.471         nan 
    388.000         nan 
      5.204         nan 
 
 AATAA  43214.0 
    402.986         nan 
    388.150         nan 
      5.202         nan 
 
 AATAA  44757.0 
    402.631         nan 
    389.950         nan 
      5.205         nan 
 
 AATAA  46300.0 
    402.601         nan 
    390.950         nan 
      5.205         nan 
 
 AATAC  10.0 
     11.542         nan 
      6.500         nan 
      2.484         nan 
 
 AATAC  1553.0 
    129.009         nan 
     83.700         nan 
      4.175         nan 
 
 AATAC  3096.0 
    159.724         nan 
    112.900         nan 
      4.235         nan 
 
 AATAC  4639.0 
    179.725         nan 
    131.400         nan 
      4.245         nan 
 
 AATAC  6182.0 
    179.607         nan 
    140.700         nan 
      4.244         nan 
 
 AATAC  7725.0 
    187.863         nan 
    152.700         nan 
      4.256         nan 
 
 AATAC  9268.0 
    195.133         nan 
    160.900         nan 
      4.257         nan 
 
 AATAC  10811.0 
    198.144         nan 
    167.150         nan 
      4.257         nan 
 
 AATAC  12354.0 
    193.595         nan 
    170.850         nan 
      4.265         nan 
 
 AATAC  13897.0 
    202.830         nan 
    175.000         nan 
      4.257         nan 
 
 AATAC  15440.0 
    203.849         nan 
    181.000         nan 
      4.267         nan 
 
 AATAC  16983.0 
    204.526         nan 
    183.800         nan 
      4.264         nan 
 
 AATAC  18526.0 
    202.017         nan 
    184.900         nan 
      4.268         nan 
 
 AATAC  20069.0 
    206.297         nan 
    188.950         nan 
      4.266         nan 
 
 AATAC  21612.0 
    204.542         nan 
    189.400         nan 
      4.266         nan 
 
 AATAC  23155.0 
    207.238         nan 
    192.050         nan 
      4.271         nan 
 
 AATAC  24698.0 
    206.909         nan 
    194.050         nan 
      4.272         nan 
 
 AATAC  26241.0 
    204.757         nan 
    194.300         nan 
      4.272         nan 
 
 AATAC  27784.0 
    205.419         nan 
    195.150         nan 
      4.266         nan 
 
 AATAC  29327.0 
    204.682         nan 
    197.150         nan 
      4.270         nan 
 
 AATAC  30870.0 
    206.278         nan 
    198.250         nan 
      4.269         nan 
 
 AATAC  32413.0 
    206.696         nan 
    199.950         nan 
      4.272         nan 
 
 AATAC  33956.0 
    205.786         nan 
    200.150         nan 
      4.272         nan 
 
 AATAC  35499.0 
    206.176         nan 
    200.350         nan 
      4.271         nan 
 
 AATAC  37042.0 
    206.421         nan 
    201.850         nan 
      4.273         nan 
 
 AATAC  38585.0 
    206.118         nan 
    201.900         nan 
      4.272         nan 
 
 AATAC  40128.0 
    206.675         nan 
    202.450         nan 
      4.271         nan 
 
 AATAC  41671.0 
    206.204         nan 
    202.800         nan 
      4.273         nan 
 
 AATAC  43214.0 
    206.646         nan 
    203.400         nan 
      4.273         nan 
 
 AATAC  44757.0 
    206.494         nan 
    203.450         nan 
      4.272         nan 
 
 AATAC  46300.0 
    206.393         nan 
    204.000         nan 
      4.272         nan 
 
 AATAG  10.0 
     19.585         nan 
      7.500         nan 
      2.754         nan 
 
 AATAG  1553.0 
    365.426         nan 
    194.350         nan 
      5.196         nan 
 
 AATAG  3096.0 
    478.774         nan 
    276.200         nan 
      5.288         nan 
 
 AATAG  4639.0 
    526.519         nan 
    333.250         nan 
      5.317         nan 
 
 AATAG  6182.0 
    554.288         nan 
    372.950         nan 
      5.330         nan 
 
 AATAG  7725.0 
    588.041         nan 
    409.950         nan 
      5.362         nan 
 
 AATAG  9268.0 
    595.200         nan 
    444.300         nan 
      5.371         nan 
 
 AATAG  10811.0 
    603.369         nan 
    461.900         nan 
      5.367         nan 
 
 AATAG  12354.0 
    624.162         nan 
    483.000         nan 
      5.375         nan 
 
 AATAG  13897.0 
    639.121         nan 
    505.300         nan 
      5.372         nan 
 
 AATAG  15440.0 
    642.792         nan 
    518.000         nan 
      5.383         nan 
 
 AATAG  16983.0 
    647.245         nan 
    533.600         nan 
      5.390         nan 
 
 AATAG  18526.0 
    657.001         nan 
    548.200         nan 
      5.391         nan 
 
 AATAG  20069.0 
    658.580         nan 
    557.400         nan 
      5.390         nan 
 
 AATAG  21612.0 
    663.647         nan 
    568.800         nan 
      5.391         nan 
 
 AATAG  23155.0 
    661.659         nan 
    576.000         nan 
      5.397         nan 
 
 AATAG  24698.0 
    670.033         nan 
    586.550         nan 
      5.402         nan 
 
 AATAG  26241.0 
    664.923         nan 
    592.850         nan 
      5.396         nan 
 
 AATAG  27784.0 
    666.547         nan 
    598.850         nan 
      5.401         nan 
 
 AATAG  29327.0 
    672.648         nan 
    608.250         nan 
      5.401         nan 
 
 AATAG  30870.0 
    671.942         nan 
    612.900         nan 
      5.405         nan 
 
 AATAG  32413.0 
    678.599         nan 
    617.400         nan 
      5.403         nan 
 
 AATAG  33956.0 
    678.934         nan 
    622.800         nan 
      5.405         nan 
 
 AATAG  35499.0 
    674.104         nan 
    626.350         nan 
      5.404         nan 
 
 AATAG  37042.0 
    677.612         nan 
    632.000         nan 
      5.406         nan 
 
 AATAG  38585.0 
    675.150         nan 
    634.800         nan 
      5.404         nan 
 
 AATAG  40128.0 
    678.134         nan 
    637.850         nan 
      5.407         nan 
 
 AATAG  41671.0 
    675.284         nan 
    640.700         nan 
      5.407         nan 
 
 AATAG  43214.0 
    677.970         nan 
    644.600         nan 
      5.407         nan 
 
 AATAG  44757.0 
    674.857         nan 
    646.600         nan 
      5.406         nan 
 
 AATAG  46300.0 
        nan         nan 
        nan         nan 
        nan         nan 
 
 AATAT  10.0 
     26.950         nan 
      8.500         nan 
      2.989         nan 
 
 AATAT  1553.0 
    203.493         nan 
    136.500         nan 
      5.206         nan 
 
 AATAT  3096.0 
    251.866         nan 
    179.700         nan 
      5.262         nan 
 
 AATAT  4639.0 
    276.439         nan 
    209.850         nan 
      5.293         nan 
 
 AATAT  6182.0 
    294.487         nan 
    228.550         nan 
      5.300         nan 
 
 AATAT  7725.0 
    301.932         nan 
    242.800         nan 
      5.311         nan 
 
 AATAT  9268.0 
    299.781         nan 
    254.250         nan 
      5.307         nan 
 
 AATAT  10811.0 
    305.138         nan 
    263.400         nan 
      5.320         nan 
 
 AATAT  12354.0 
    311.323         nan 
    272.050         nan 
      5.320         nan 
 
 AATAT  13897.0 
    311.376         nan 
    278.400         nan 
      5.318         nan 
 
 AATAT  15440.0 
    320.308         nan 
    284.700         nan 
      5.318         nan 
 
 AATAT  16983.0 
    319.049         nan 
    288.650         nan 
      5.327         nan 
 
 AATAT  18526.0 
    324.613         nan 
    294.250         nan 
      5.325         nan 
 
 AATAT  20069.0 
    328.099         nan 
    298.550         nan 
      5.326         nan 
 
 AATAT  21612.0 
    323.844         nan 
    300.250         nan 
      5.326         nan 
 
 AATAT  23155.0 
    325.860         nan 
    302.000         nan 
      5.330         nan 
 
 AATAT  24698.0 
    328.656         nan 
    305.250         nan 
      5.334         nan 
 
 AATAT  26241.0 
    332.058         nan 
    307.650         nan 
      5.327         nan 
 
 AATAT  27784.0 
    328.185         nan 
    309.500         nan 
      5.328         nan 
 
 AATAT  29327.0 
    329.608         nan 
    311.050         nan 
      5.334         nan 
 
 AATAT  30870.0 
    330.188         nan 
    312.600         nan 
      5.335         nan 
 
 AATAT  32413.0 
    334.962         nan 
    314.550         nan 
      5.331         nan 
 
 AATAT  33956.0 
    332.838         nan 
    315.800         nan 
      5.334         nan 
 
 AATAT  35499.0 
    332.661         nan 
    316.850         nan 
      5.332         nan 
 
 AATAT  37042.0 
    333.321         nan 
    318.400         nan 
      5.333         nan 
 
 AATAT  38585.0 
    332.356         nan 
    318.700         nan 
      5.334         nan 
 
 AATAT  40128.0 
    333.807         nan 
    320.300         nan 
      5.333         nan 
 
 AATAT  41671.0 
    333.821         nan 
    321.000         nan 
      5.333         nan 
 
 AATAT  43214.0 
    334.785         nan 
    322.900         nan 
      5.334         nan 
 
 AATAT  44757.0 
    334.301         nan 
    323.350         nan 
      5.333         nan 
 
 AATAT  46300.0 
    334.166         nan 
    324.000         nan 
      5.334         nan 
 
 AATTA  10.0 
     31.175         nan 
      9.050         nan 
      3.128         nan 
 
 AATTA  1553.0 
    710.944         nan 
    401.450         nan 
      7.061         nan 
 
 AATTA  3096.0 
    815.254         nan 
    561.900         nan 
      7.230         nan 
 
 AATTA  4639.0 
    848.638         nan 
    660.450         nan 
      7.290         nan 
 
 AATTA  6182.0 
    875.559         nan 
    727.350         nan 
      7.322         nan 
 
 AATTA  7725.0 
    900.633         nan 
    772.850         nan 
      7.347         nan 
 
 AATTA  9268.0 
    907.823         nan 
    807.200         nan 
      7.354         nan 
 
 AATTA  10811.0 
    914.207         nan 
    829.200         nan 
      7.364         nan 
 
 AATTA  12354.0 
    920.602         nan 
    851.150         nan 
      7.390         nan 
 
 AATTA  13897.0 
    928.232         nan 
    866.250         nan 
      7.381         nan 
 
 AATTA  15440.0 
    926.439         nan 
    877.300         nan 
      7.392         nan 
 
 AATTA  16983.0 
    928.087         nan 
    883.000         nan 
      7.397         nan 
 
 AATTA  18526.0 
    937.147         nan 
    894.000         nan 
      7.404         nan 
 
 AATTA  20069.0 
    938.783         nan 
    900.200         nan 
      7.401         nan 
 
 AATTA  21612.0 
    942.375         nan 
    906.400         nan 
      7.411         nan 
 
 AATTA  23155.0 
    944.768         nan 
    911.700         nan 
      7.409         nan 
 
 AATTA  24698.0 
    948.343         nan 
    915.850         nan 
      7.412         nan 
 
 AATTA  26241.0 
    950.034         nan 
    921.350         nan 
      7.413         nan 
 
 AATTA  27784.0 
    946.051         nan 
    921.500         nan 
      7.415         nan 
 
 AATTA  29327.0 
    955.286         nan 
    927.300         nan 
      7.418         nan 
 
 AATTA  30870.0 
    953.584         nan 
    928.750         nan 
      7.417         nan 
 
 AATTA  32413.0 
    950.501         nan 
    930.150         nan 
      7.420         nan 
 
 AATTA  33956.0 
    958.533         nan 
    933.100         nan 
      7.420         nan 
 
 AATTA  35499.0 
    955.663         nan 
    934.550         nan 
      7.419         nan 
 
 AATTA  37042.0 
    959.841         nan 
    937.450         nan 
      7.421         nan 
 
 AATTA  38585.0 
    959.969         nan 
    938.600         nan 
      7.421         nan 
 
 AATTA  40128.0 
    963.149         nan 
    940.350         nan 
      7.424         nan 
 
 AATTA  41671.0 
    964.576         nan 
    942.300         nan 
      7.423         nan 
 
 AATTA  43214.0 
    966.445         nan 
    942.800         nan 
      7.425         nan 
 
 AATTA  44757.0 
    968.959         nan 
    944.600         nan 
      7.424         nan 
 
 AATTA  46300.0 
    970.667         nan 
    946.000         nan 
      7.425         nan 
 
 AATTC  10.0 
     11.717         nan 
      6.050         nan 
      2.355         nan 
 
 AATTC  1553.0 
    162.420         nan 
     91.600         nan 
      3.786         nan 
 
 AATTC  3096.0 
    199.462         nan 
    122.350         nan 
      3.802         nan 
 
 AATTC  4639.0 
    223.711         nan 
    147.150         nan 
      3.813         nan 
 
 AATTC  6182.0 
    234.353         nan 
    161.100         nan 
      3.822         nan 
 
 AATTC  7725.0 
    246.529         nan 
    174.800         nan 
      3.814         nan 
 
 AATTC  9268.0 
    257.838         nan 
    188.800         nan 
      3.831         nan 
 
 AATTC  10811.0 
    275.462         nan 
    198.350         nan 
      3.839         nan 
 
 AATTC  12354.0 
    280.012         nan 
    206.450         nan 
      3.838         nan 
 
 AATTC  13897.0 
    282.757         nan 
    216.850         nan 
      3.846         nan 
 
 AATTC  15440.0 
    300.006         nan 
    224.700         nan 
      3.843         nan 
 
 AATTC  16983.0 
    292.657         nan 
    229.200         nan 
      3.845         nan 
 
 AATTC  18526.0 
    290.710         nan 
    233.250         nan 
      3.846         nan 
 
 AATTC  20069.0 
    299.672         nan 
    241.500         nan 
      3.844         nan 
 
 AATTC  21612.0 
    302.795         nan 
    246.150         nan 
      3.853         nan 
 
 AATTC  23155.0 
    299.232         nan 
    249.400         nan 
      3.849         nan 
 
 AATTC  24698.0 
    306.505         nan 
    253.950         nan 
      3.854         nan 
 
 AATTC  26241.0 
    306.703         nan 
    258.600         nan 
      3.852         nan 
 
 AATTC  27784.0 
    307.653         nan 
    260.700         nan 
      3.852         nan 
 
 AATTC  29327.0 
    309.012         nan 
    266.000         nan 
      3.853         nan 
 
 AATTC  30870.0 
    301.291         nan 
    267.450         nan 
      3.852         nan 
 
 AATTC  32413.0 
    305.888         nan 
    270.650         nan 
      3.849         nan 
 
 AATTC  33956.0 
    304.022         nan 
    273.550         nan 
      3.856         nan 
 
 AATTC  35499.0 
    305.442         nan 
    274.950         nan 
      3.848         nan 
 
 AATTC  37042.0 
    306.511         nan 
    278.400         nan 
      3.852         nan 
 
 AATTC  38585.0 
    304.608         nan 
    279.400         nan 
      3.852         nan 
 
 AATTC  40128.0 
    305.814         nan 
    282.450         nan 
      3.854         nan 
 
 AATTC  41671.0 
    304.761         nan 
    283.600         nan 
      3.853         nan 
 
 AATTC  43214.0 
    303.013         nan 
    285.050         nan 
      3.854         nan 
 
 AATTC  44757.0 
    303.521         nan 
    286.250         nan 
      3.855         nan 
 
 AATTC  46300.0 
    303.945         nan 
    287.950         nan 
      3.854         nan 
 
 AATTT  10.0 
     25.450         nan 
      8.550         nan 
      3.007         nan 
 
 AATTT  1553.0 
    309.520         nan 
    174.550         nan 
      5.720         nan 
 
 AATTT  3096.0 
    414.052         nan 
    245.600         nan 
      5.801         nan 
 
 AATTT  4639.0 
    448.920         nan 
    291.150         nan 
      5.822         nan 
 
 AATTT  6182.0 
    526.753         nan 
    331.450         nan 
      5.846         nan 
 
 AATTT  7725.0 
    526.586         nan 
    356.200         nan 
      5.855         nan 
 
 AATTT  9268.0 
    561.287         nan 
    388.800         nan 
      5.873         nan 
 
 AATTT  10811.0 
    573.968         nan 
    405.150         nan 
      5.880         nan 
 
 AATTT  12354.0 
    591.287         nan 
    427.850         nan 
      5.875         nan 
 
 AATTT  13897.0 
    589.996         nan 
    444.000         nan 
      5.886         nan 
 
 AATTT  15440.0 
    606.293         nan 
    459.450         nan 
      5.889         nan 
 
 AATTT  16983.0 
    619.998         nan 
    475.150         nan 
      5.888         nan 
 
 AATTT  18526.0 
    625.802         nan 
    489.200         nan 
      5.892         nan 
 
 AATTT  20069.0 
    634.954         nan 
    502.550         nan 
      5.894         nan 
 
 AATTT  21612.0 
    634.708         nan 
    510.400         nan 
      5.897         nan 
 
 AATTT  23155.0 
    638.900         nan 
    522.800         nan 
      5.899         nan 
 
 AATTT  24698.0 
    655.083         nan 
    535.550         nan 
      5.902         nan 
 
 AATTT  26241.0 
    647.386         nan 
    543.200         nan 
      5.900         nan 
 
 AATTT  27784.0 
    642.254         nan 
    549.350         nan 
      5.904         nan 
 
 AATTT  29327.0 
    652.513         nan 
    559.650         nan 
      5.901         nan 
 
 AATTT  30870.0 
    648.872         nan 
    564.100         nan 
      5.903         nan 
 
 AATTT  32413.0 
    645.293         nan 
    568.850         nan 
      5.905         nan 
 
 AATTT  33956.0 
    651.364         nan 
    578.150         nan 
      5.907         nan 
 
 AATTT  35499.0 
    651.752         nan 
    582.300         nan 
      5.907         nan 
 
 AATTT  37042.0 
    644.143         nan 
    586.450         nan 
      5.909         nan 
 
 AATTT  38585.0 
    645.663         nan 
    590.350         nan 
      5.907         nan 
 
 AATTT  40128.0 
    644.170         nan 
    595.200         nan 
      5.908         nan 
 
 AATTT  41671.0 
    642.726         nan 
    599.950         nan 
      5.910         nan 
 
 AATTT  43214.0 
    642.215         nan 
    603.800         nan 
      5.910         nan 
 
 AATTT  44757.0 
    639.622         nan 
    606.350         nan 
      5.910         nan 
 
 AATTT  46300.0 
        nan         nan 
        nan         nan 
        nan         nan 
 
  Description  Seqs/Sample 
 chao1 Ave.  chao1 Err. 
 observed_species Ave.  observed_species Err. 
 shannon Ave.  shannon Err. 
 
 
 A01  10.0 
     13.460         nan 
      7.200         nan 
      2.710         nan 
 
 A01  1553.0 
    143.566         nan 
     83.500         nan 
      4.309         nan 
 
 A01  3096.0 
    183.101         nan 
    111.200         nan 
      4.320         nan 
 
 A01  4639.0 
    219.139         nan 
    134.050         nan 
      4.350         nan 
 
 A01  6182.0 
    231.738         nan 
    151.150         nan 
      4.359         nan 
 
 A01  7725.0 
    241.876         nan 
    161.150         nan 
      4.351         nan 
 
 A01  9268.0 
    246.748         nan 
    174.500         nan 
      4.360         nan 
 
 A01  10811.0 
    256.697         nan 
    184.550         nan 
      4.366         nan 
 
 A01  12354.0 
    269.593         nan 
    194.600         nan 
      4.369         nan 
 
 A01  13897.0 
    266.605         nan 
    199.500         nan 
      4.364         nan 
 
 A01  15440.0 
    266.184         nan 
    205.250         nan 
      4.366         nan 
 
 A01  16983.0 
    273.905         nan 
    215.750         nan 
      4.372         nan 
 
 A01  18526.0 
    265.761         nan 
    218.600         nan 
      4.376         nan 
 
 A01  20069.0 
    271.658         nan 
    224.900         nan 
      4.375         nan 
 
 A01  21612.0 
    267.841         nan 
    228.900         nan 
      4.376         nan 
 
 A01  23155.0 
    271.397         nan 
    232.900         nan 
      4.375         nan 
 
 A01  24698.0 
    272.454         nan 
    236.100         nan 
      4.374         nan 
 
 A01  26241.0 
    275.571         nan 
    240.750         nan 
      4.376         nan 
 
 A01  27784.0 
    275.992         nan 
    245.050         nan 
      4.379         nan 
 
 A01  29327.0 
    274.484         nan 
    246.700         nan 
      4.379         nan 
 
 A01  30870.0 
    269.618         nan 
    247.150         nan 
      4.377         nan 
 
 A01  32413.0 
    266.919         nan 
    250.500         nan 
      4.377         nan 
 
 A01  33956.0 
    267.089         nan 
    252.350         nan 
      4.378         nan 
 
 A01  35499.0 
    265.820         nan 
    254.000         nan 
      4.378         nan 
 
 A01  37042.0 
    265.929         nan 
    255.800         nan 
      4.380         nan 
 
 A01  38585.0 
    264.324         nan 
    256.750         nan 
      4.378         nan 
 
 A01  40128.0 
    263.007         nan 
    258.000         nan 
      4.380         nan 
 
 A01  41671.0 
    263.381         nan 
    259.250         nan 
      4.380         nan 
 
 A01  43214.0 
    263.345         nan 
    260.250         nan 
      4.380         nan 
 
 A01  44757.0 
    262.140         nan 
    260.650         nan 
      4.381         nan 
 
 A01  46300.0 
        nan         nan 
        nan         nan 
        nan         nan 
 
 A02  10.0 
     16.800         nan 
      7.500         nan 
      2.768         nan 
 
 A02  1553.0 
     98.090         nan 
     66.100         nan 
      4.538         nan 
 
 A02  3096.0 
    103.836         nan 
     79.100         nan 
      4.560         nan 
 
 A02  4639.0 
    126.607         nan 
     90.650         nan 
      4.577         nan 
 
 A02  6182.0 
    135.829         nan 
     97.250         nan 
      4.572         nan 
 
 A02  7725.0 
    138.571         nan 
    104.100         nan 
      4.575         nan 
 
 A02  9268.0 
    153.311         nan 
    109.550         nan 
      4.588         nan 
 
 A02  10811.0 
    147.420         nan 
    113.000         nan 
      4.581         nan 
 
 A02  12354.0 
    152.059         nan 
    119.250         nan 
      4.583         nan 
 
 A02  13897.0 
    150.117         nan 
    119.950         nan 
      4.582         nan 
 
 A02  15440.0 
    151.888         nan 
    123.850         nan 
      4.578         nan 
 
 A02  16983.0 
    153.484         nan 
    127.850         nan 
      4.583         nan 
 
 A02  18526.0 
    152.399         nan 
    129.800         nan 
      4.582         nan 
 
 A02  20069.0 
    155.152         nan 
    134.350         nan 
      4.589         nan 
 
 A02  21612.0 
    152.857         nan 
    135.350         nan 
      4.588         nan 
 
 A02  23155.0 
    154.124         nan 
    137.100         nan 
      4.588         nan 
 
 A02  24698.0 
    151.876         nan 
    137.350         nan 
      4.584         nan 
 
 A02  26241.0 
    151.355         nan 
    140.300         nan 
      4.586         nan 
 
 A02  27784.0 
    152.822         nan 
    142.500         nan 
      4.589         nan 
 
 A02  29327.0 
    151.477         nan 
    142.900         nan 
      4.589         nan 
 
 A02  30870.0 
    152.283         nan 
    143.950         nan 
      4.591         nan 
 
 A02  32413.0 
    150.937         nan 
    144.550         nan 
      4.589         nan 
 
 A02  33956.0 
    151.101         nan 
    146.400         nan 
      4.588         nan 
 
 A02  35499.0 
    151.072         nan 
    147.300         nan 
      4.589         nan 
 
 A02  37042.0 
    150.620         nan 
    147.550         nan 
      4.588         nan 
 
 A02  38585.0 
    149.167         nan 
    147.850         nan 
      4.588         nan 
 
 A02  40128.0 
    149.790         nan 
    148.350         nan 
      4.588         nan 
 
 A02  41671.0 
    149.351         nan 
    148.650         nan 
      4.588         nan 
 
 A02  43214.0 
    149.050         nan 
    148.750         nan 
      4.588         nan 
 
 A02  44757.0 
    149.065         nan 
    149.000         nan 
      4.589         nan 
 
 A02  46300.0 
        nan         nan 
        nan         nan 
        nan         nan 
 
 A03  10.0 
     23.225         nan 
      8.600         nan 
      3.028         nan 
 
 A03  1553.0 
    354.040         nan 
    208.000         nan 
      5.726         nan 
 
 A03  3096.0 
    404.240         nan 
    281.600         nan 
      5.787         nan 
 
 A03  4639.0 
    415.803         nan 
    324.300         nan 
      5.841         nan 
 
 A03  6182.0 
    426.677         nan 
    351.700         nan 
      5.865         nan 
 
 A03  7725.0 
    439.880         nan 
    373.000         nan 
      5.854         nan 
 
 A03  9268.0 
    452.576         nan 
    389.000         nan 
      5.862         nan 
 
 A03  10811.0 
    454.955         nan 
    401.000         nan 
      5.882         nan 
 
 A03  12354.0 
    462.824         nan 
    413.200         nan 
      5.872         nan 
 
 A03  13897.0 
    471.267         nan 
    421.750         nan 
      5.870         nan 
 
 A03  15440.0 
    468.377         nan 
    427.650         nan 
      5.875         nan 
 
 A03  16983.0 
    464.227         nan 
    433.250         nan 
      5.888         nan 
 
 A03  18526.0 
    470.228         nan 
    438.550         nan 
      5.881         nan 
 
 A03  20069.0 
    477.009         nan 
    445.350         nan 
      5.889         nan 
 
 A03  21612.0 
    478.131         nan 
    448.750         nan 
      5.891         nan 
 
 A03  23155.0 
    472.750         nan 
    450.050         nan 
      5.893         nan 
 
 A03  24698.0 
    477.940         nan 
    454.150         nan 
      5.890         nan 
 
 A03  26241.0 
    479.128         nan 
    457.550         nan 
      5.888         nan 
 
 A03  27784.0 
    476.269         nan 
    459.250         nan 
      5.895         nan 
 
 A03  29327.0 
    483.202         nan 
    462.150         nan 
      5.897         nan 
 
 A03  30870.0 
    479.549         nan 
    462.650         nan 
      5.898         nan 
 
 A03  32413.0 
    478.138         nan 
    463.950         nan 
      5.896         nan 
 
 A03  33956.0 
    478.809         nan 
    465.450         nan 
      5.897         nan 
 
 A03  35499.0 
    480.029         nan 
    467.700         nan 
      5.895         nan 
 
 A03  37042.0 
    480.141         nan 
    468.500         nan 
      5.896         nan 
 
 A03  38585.0 
    481.266         nan 
    469.850         nan 
      5.898         nan 
 
 A03  40128.0 
    481.598         nan 
    470.800         nan 
      5.900         nan 
 
 A03  41671.0 
    481.240         nan 
    471.650         nan 
      5.898         nan 
 
 A03  43214.0 
    481.833         nan 
    472.150         nan 
      5.898         nan 
 
 A03  44757.0 
    481.720         nan 
    473.150         nan 
      5.898         nan 
 
 A03  46300.0 
    482.762         nan 
    474.000         nan 
      5.899         nan 
 
 A04  10.0 
     22.275         nan 
      8.000         nan 
      2.873         nan 
 
 A04  1553.0 
    261.024         nan 
    162.900         nan 
      5.250         nan 
 
 A04  3096.0 
    287.134         nan 
    213.350         nan 
      5.311         nan 
 
 A04  4639.0 
    315.337         nan 
    244.000         nan 
      5.343         nan 
 
 A04  6182.0 
    317.813         nan 
    262.850         nan 
      5.348         nan 
 
 A04  7725.0 
    341.689         nan 
    284.450         nan 
      5.353         nan 
 
 A04  9268.0 
    340.222         nan 
    293.050         nan 
      5.361         nan 
 
 A04  10811.0 
    355.262         nan 
    305.100         nan 
      5.377         nan 
 
 A04  12354.0 
    358.778         nan 
    314.700         nan 
      5.378         nan 
 
 A04  13897.0 
    362.498         nan 
    321.100         nan 
      5.379         nan 
 
 A04  15440.0 
    365.593         nan 
    327.250         nan 
      5.371         nan 
 
 A04  16983.0 
    365.968         nan 
    332.000         nan 
      5.380         nan 
 
 A04  18526.0 
    367.835         nan 
    335.400         nan 
      5.382         nan 
 
 A04  20069.0 
    371.082         nan 
    339.800         nan 
      5.383         nan 
 
 A04  21612.0 
    374.429         nan 
    344.950         nan 
      5.387         nan 
 
 A04  23155.0 
    374.900         nan 
    347.350         nan 
      5.384         nan 
 
 A04  24698.0 
    375.540         nan 
    350.100         nan 
      5.388         nan 
 
 A04  26241.0 
    374.762         nan 
    352.450         nan 
      5.388         nan 
 
 A04  27784.0 
    377.844         nan 
    356.150         nan 
      5.391         nan 
 
 A04  29327.0 
    377.822         nan 
    358.650         nan 
      5.386         nan 
 
 A04  30870.0 
    378.494         nan 
    359.050         nan 
      5.387         nan 
 
 A04  32413.0 
    374.710         nan 
    361.050         nan 
      5.388         nan 
 
 A04  33956.0 
    374.578         nan 
    362.250         nan 
      5.388         nan 
 
 A04  35499.0 
    375.072         nan 
    363.500         nan 
      5.388         nan 
 
 A04  37042.0 
    375.091         nan 
    364.000         nan 
      5.387         nan 
 
 A04  38585.0 
    373.100         nan 
    365.500         nan 
      5.390         nan 
 
 A04  40128.0 
    372.383         nan 
    366.050         nan 
      5.388         nan 
 
 A04  41671.0 
    371.961         nan 
    367.450         nan 
      5.390         nan 
 
 A04  43214.0 
    371.554         nan 
    367.800         nan 
      5.389         nan 
 
 A04  44757.0 
    370.929         nan 
    368.500         nan 
      5.390         nan 
 
 A04  46300.0 
    370.712         nan 
    369.000         nan 
      5.390         nan 
 
 A05  10.0 
     10.067         nan 
      6.350         nan 
      2.453         nan 
 
 A05  1553.0 
    106.510         nan 
     58.900         nan 
      3.809         nan 
 
 A05  3096.0 
    118.318         nan 
     75.150         nan 
      3.813         nan 
 
 A05  4639.0 
    127.828         nan 
     88.100         nan 
      3.833         nan 
 
 A05  6182.0 
    133.552         nan 
     94.350         nan 
      3.820         nan 
 
 A05  7725.0 
    146.678         nan 
    104.950         nan 
      3.833         nan 
 
 A05  9268.0 
    146.871         nan 
    111.350         nan 
      3.835         nan 
 
 A05  10811.0 
    152.116         nan 
    117.750         nan 
      3.837         nan 
 
 A05  12354.0 
    153.553         nan 
    120.150         nan 
      3.841         nan 
 
 A05  13897.0 
    156.110         nan 
    127.650         nan 
      3.839         nan 
 
 A05  15440.0 
    158.781         nan 
    130.250         nan 
      3.838         nan 
 
 A05  16983.0 
    159.438         nan 
    133.550         nan 
      3.842         nan 
 
 A05  18526.0 
    159.567         nan 
    136.100         nan 
      3.839         nan 
 
 A05  20069.0 
    157.719         nan 
    139.200         nan 
      3.844         nan 
 
 A05  21612.0 
    157.939         nan 
    140.800         nan 
      3.841         nan 
 
 A05  23155.0 
    158.607         nan 
    143.400         nan 
      3.838         nan 
 
 A05  24698.0 
    162.023         nan 
    145.150         nan 
      3.844         nan 
 
 A05  26241.0 
    157.573         nan 
    146.950         nan 
      3.847         nan 
 
 A05  27784.0 
    158.990         nan 
    148.050         nan 
      3.845         nan 
 
 A05  29327.0 
    159.099         nan 
    149.700         nan 
      3.844         nan 
 
 A05  30870.0 
    158.129         nan 
    150.050         nan 
      3.844         nan 
 
 A05  32413.0 
    156.594         nan 
    151.300         nan 
      3.844         nan 
 
 A05  33956.0 
    157.779         nan 
    152.800         nan 
      3.845         nan 
 
 A05  35499.0 
    156.781         nan 
    153.350         nan 
      3.846         nan 
 
 A05  37042.0 
    155.625         nan 
    153.400         nan 
      3.845         nan 
 
 A05  38585.0 
    155.604         nan 
    153.950         nan 
      3.846         nan 
 
 A05  40128.0 
    155.410         nan 
    154.350         nan 
      3.846         nan 
 
 A05  41671.0 
    155.375         nan 
    154.800         nan 
      3.846         nan 
 
 A05  43214.0 
    155.254         nan 
    154.950         nan 
      3.846         nan 
 
 A05  44757.0 
    155.108         nan 
    155.000         nan 
      3.846         nan 
 
 A05  46300.0 
    155.000         nan 
    155.000         nan 
      3.846         nan 
 
 A06  10.0 
     18.225         nan 
      7.700         nan 
      2.822         nan 
 
 A06  1553.0 
    216.990         nan 
    138.200         nan 
      5.063         nan 
 
 A06  3096.0 
    272.482         nan 
    181.450         nan 
      5.120         nan 
 
 A06  4639.0 
    299.225         nan 
    211.100         nan 
      5.130         nan 
 
 A06  6182.0 
    308.372         nan 
    233.100         nan 
      5.140         nan 
 
 A06  7725.0 
    323.732         nan 
    251.300         nan 
      5.156         nan 
 
 A06  9268.0 
    326.919         nan 
    263.950         nan 
      5.160         nan 
 
 A06  10811.0 
    332.098         nan 
    274.100         nan 
      5.162         nan 
 
 A06  12354.0 
    340.432         nan 
    284.000         nan 
      5.169         nan 
 
 A06  13897.0 
    347.642         nan 
    295.950         nan 
      5.171         nan 
 
 A06  15440.0 
    340.408         nan 
    298.500         nan 
      5.172         nan 
 
 A06  16983.0 
    346.765         nan 
    306.750         nan 
      5.176         nan 
 
 A06  18526.0 
    346.982         nan 
    310.500         nan 
      5.172         nan 
 
 A06  20069.0 
    347.487         nan 
    316.150         nan 
      5.181         nan 
 
 A06  21612.0 
    354.439         nan 
    319.550         nan 
      5.180         nan 
 
 A06  23155.0 
    350.907         nan 
    322.100         nan 
      5.177         nan 
 
 A06  24698.0 
    352.456         nan 
    327.000         nan 
      5.175         nan 
 
 A06  26241.0 
    348.673         nan 
    329.350         nan 
      5.181         nan 
 
 A06  27784.0 
    351.936         nan 
    331.550         nan 
      5.177         nan 
 
 A06  29327.0 
    354.258         nan 
    335.850         nan 
      5.180         nan 
 
 A06  30870.0 
    351.654         nan 
    335.650         nan 
      5.180         nan 
 
 A06  32413.0 
    351.768         nan 
    338.150         nan 
      5.180         nan 
 
 A06  33956.0 
    351.582         nan 
    339.850         nan 
      5.181         nan 
 
 A06  35499.0 
    352.211         nan 
    341.050         nan 
      5.184         nan 
 
 A06  37042.0 
    351.828         nan 
    341.850         nan 
      5.183         nan 
 
 A06  38585.0 
    351.376         nan 
    343.600         nan 
      5.184         nan 
 
 A06  40128.0 
    351.341         nan 
    344.450         nan 
      5.184         nan 
 
 A06  41671.0 
    349.960         nan 
    345.550         nan 
      5.183         nan 
 
 A06  43214.0 
    349.491         nan 
    345.850         nan 
      5.186         nan 
 
 A06  44757.0 
    349.074         nan 
    346.600         nan 
      5.185         nan 
 
 A06  46300.0 
    348.665         nan 
    346.950         nan 
      5.184         nan 
 
 A07  10.0 
     18.250         nan 
      8.050         nan 
      2.911         nan 
 
 A07  1553.0 
    171.159         nan 
    106.150         nan 
      5.147         nan 
 
 A07  3096.0 
    222.244         nan 
    138.800         nan 
      5.205         nan 
 
 A07  4639.0 
    240.379         nan 
    160.250         nan 
      5.212         nan 
 
 A07  6182.0 
    276.849         nan 
    177.100         nan 
      5.217         nan 
 
 A07  7725.0 
    279.056         nan 
    189.900         nan 
      5.219         nan 
 
 A07  9268.0 
    297.130         nan 
    203.150         nan 
      5.217         nan 
 
 A07  10811.0 
    298.418         nan 
    211.550         nan 
      5.223         nan 
 
 A07  12354.0 
    301.960         nan 
    223.550         nan 
      5.230         nan 
 
 A07  13897.0 
    296.278         nan 
    231.150         nan 
      5.236         nan 
 
 A07  15440.0 
    301.236         nan 
    240.200         nan 
      5.230         nan 
 
 A07  16983.0 
    313.601         nan 
    249.300         nan 
      5.232         nan 
 
 A07  18526.0 
    311.381         nan 
    250.100         nan 
      5.230         nan 
 
 A07  20069.0 
    313.954         nan 
    258.850         nan 
      5.237         nan 
 
 A07  21612.0 
    311.018         nan 
    263.300         nan 
      5.234         nan 
 
 A07  23155.0 
    315.783         nan 
    270.150         nan 
      5.239         nan 
 
 A07  24698.0 
    312.240         nan 
    273.000         nan 
      5.237         nan 
 
 A07  26241.0 
    310.858         nan 
    276.700         nan 
      5.239         nan 
 
 A07  27784.0 
    315.036         nan 
    279.650         nan 
      5.238         nan 
 
 A07  29327.0 
    313.750         nan 
    284.000         nan 
      5.239         nan 
 
 A07  30870.0 
    310.224         nan 
    286.800         nan 
      5.237         nan 
 
 A07  32413.0 
    310.285         nan 
    289.500         nan 
      5.240         nan 
 
 A07  33956.0 
    311.160         nan 
    292.200         nan 
      5.236         nan 
 
 A07  35499.0 
    307.911         nan 
    293.350         nan 
      5.241         nan 
 
 A07  37042.0 
    308.393         nan 
    295.700         nan 
      5.241         nan 
 
 A07  38585.0 
    304.972         nan 
    296.450         nan 
      5.242         nan 
 
 A07  40128.0 
    305.507         nan 
    297.850         nan 
      5.241         nan 
 
 A07  41671.0 
    305.137         nan 
    299.400         nan 
      5.243         nan 
 
 A07  43214.0 
    304.780         nan 
    300.900         nan 
      5.242         nan 
 
 A07  44757.0 
    304.382         nan 
    301.650         nan 
      5.241         nan 
 
 A07  46300.0 
    303.364         nan 
    302.000         nan 
      5.242         nan 
 
 A08  10.0 
     20.875         nan 
      8.150         nan 
      2.929         nan 
 
 A08  1553.0 
    293.045         nan 
    138.950         nan 
      5.196         nan 
 
 A08  3096.0 
    377.470         nan 
    200.500         nan 
      5.244         nan 
 
 A08  4639.0 
    440.541         nan 
    249.450         nan 
      5.280         nan 
 
 A08  6182.0 
    445.577         nan 
    280.600         nan 
      5.297         nan 
 
 A08  7725.0 
    493.514         nan 
    311.350         nan 
      5.299         nan 
 
 A08  9268.0 
    496.078         nan 
    340.400         nan 
      5.308         nan 
 
 A08  10811.0 
    499.195         nan 
    358.850         nan 
      5.326         nan 
 
 A08  12354.0 
    508.754         nan 
    377.750         nan 
      5.322         nan 
 
 A08  13897.0 
    510.334         nan 
    393.200         nan 
      5.325         nan 
 
 A08  15440.0 
    512.669         nan 
    409.050         nan 
      5.333         nan 
 
 A08  16983.0 
    517.998         nan 
    422.100         nan 
      5.333         nan 
 
 A08  18526.0 
    520.362         nan 
    434.100         nan 
      5.334         nan 
 
 A08  20069.0 
    531.591         nan 
    447.550         nan 
      5.338         nan 
 
 A08  21612.0 
    529.702         nan 
    456.650         nan 
      5.338         nan 
 
 A08  23155.0 
    533.185         nan 
    461.250         nan 
      5.338         nan 
 
 A08  24698.0 
    524.230         nan 
    470.050         nan 
      5.342         nan 
 
 A08  26241.0 
    532.521         nan 
    477.900         nan 
      5.343         nan 
 
 A08  27784.0 
    528.560         nan 
    482.900         nan 
      5.343         nan 
 
 A08  29327.0 
    528.124         nan 
    486.500         nan 
      5.343         nan 
 
 A08  30870.0 
    525.610         nan 
    492.500         nan 
      5.344         nan 
 
 A08  32413.0 
    524.832         nan 
    495.050         nan 
      5.344         nan 
 
 A08  33956.0 
    523.966         nan 
    500.500         nan 
      5.348         nan 
 
 A08  35499.0 
    523.395         nan 
    503.000         nan 
      5.346         nan 
 
 A08  37042.0 
    520.582         nan 
    505.400         nan 
      5.348         nan 
 
 A08  38585.0 
    518.193         nan 
    507.000         nan 
      5.349         nan 
 
 A08  40128.0 
    515.406         nan 
    508.150         nan 
      5.350         nan 
 
 A08  41671.0 
    515.222         nan 
    509.900         nan 
      5.349         nan 
 
 A08  43214.0 
    515.186         nan 
    511.600         nan 
      5.349         nan 
 
 A08  44757.0 
    513.934         nan 
    512.350         nan 
      5.349         nan 
 
 A08  46300.0 
        nan         nan 
        nan         nan 
        nan         nan 
 
 A09  10.0 
     14.375         nan 
      7.350         nan 
      2.729         nan 
 
 A09  1553.0 
    178.007         nan 
    111.400         nan 
      4.803         nan 
 
 A09  3096.0 
    211.053         nan 
    145.250         nan 
      4.861         nan 
 
 A09  4639.0 
    234.992         nan 
    169.050         nan 
      4.875         nan 
 
 A09  6182.0 
    247.141         nan 
    188.700         nan 
      4.889         nan 
 
 A09  7725.0 
    247.287         nan 
    197.900         nan 
      4.881         nan 
 
 A09  9268.0 
    263.070         nan 
    211.500         nan 
      4.899         nan 
 
 A09  10811.0 
    270.399         nan 
    221.550         nan 
      4.911         nan 
 
 A09  12354.0 
    269.470         nan 
    226.850         nan 
      4.904         nan 
 
 A09  13897.0 
    269.818         nan 
    231.350         nan 
      4.901         nan 
 
 A09  15440.0 
    272.411         nan 
    238.400         nan 
      4.913         nan 
 
 A09  16983.0 
    274.316         nan 
    243.900         nan 
      4.906         nan 
 
 A09  18526.0 
    273.275         nan 
    247.000         nan 
      4.909         nan 
 
 A09  20069.0 
    277.406         nan 
    251.200         nan 
      4.912         nan 
 
 A09  21612.0 
    274.667         nan 
    253.300         nan 
      4.914         nan 
 
 A09  23155.0 
    273.951         nan 
    256.450         nan 
      4.917         nan 
 
 A09  24698.0 
    277.225         nan 
    259.650         nan 
      4.910         nan 
 
 A09  26241.0 
    275.256         nan 
    260.700         nan 
      4.909         nan 
 
 A09  27784.0 
    273.190         nan 
    263.200         nan 
      4.913         nan 
 
 A09  29327.0 
    273.793         nan 
    264.400         nan 
      4.916         nan 
 
 A09  30870.0 
    272.180         nan 
    265.350         nan 
      4.916         nan 
 
 A09  32413.0 
    272.900         nan 
    266.700         nan 
      4.915         nan 
 
 A09  33956.0 
    273.000         nan 
    268.000         nan 
      4.915         nan 
 
 A09  35499.0 
    272.846         nan 
    268.600         nan 
      4.915         nan 
 
 A09  37042.0 
    272.274         nan 
    269.400         nan 
      4.915         nan 
 
 A09  38585.0 
    271.689         nan 
    269.600         nan 
      4.916         nan 
 
 A09  40128.0 
    271.463         nan 
    270.450         nan 
      4.917         nan 
 
 A09  41671.0 
    271.322         nan 
    270.700         nan 
      4.917         nan 
 
 A09  43214.0 
    271.224         nan 
    270.900         nan 
      4.916         nan 
 
 A09  44757.0 
    271.056         nan 
    271.000         nan 
      4.917         nan 
 
 A09  46300.0 
    271.000         nan 
    271.000         nan 
      4.917         nan 
 
 A10  10.0 
     22.200         nan 
      8.150         nan 
      2.919         nan 
 
 A10  1553.0 
    242.391         nan 
    117.750         nan 
      4.649         nan 
 
 A10  3096.0 
    313.905         nan 
    165.750         nan 
      4.699         nan 
 
 A10  4639.0 
    353.874         nan 
    199.900         nan 
      4.736         nan 
 
 A10  6182.0 
    369.505         nan 
    223.300         nan 
      4.737         nan 
 
 A10  7725.0 
    411.911         nan 
    250.600         nan 
      4.747         nan 
 
 A10  9268.0 
    418.491         nan 
    271.650         nan 
      4.764         nan 
 
 A10  10811.0 
    433.524         nan 
    289.050         nan 
      4.757         nan 
 
 A10  12354.0 
    437.387         nan 
    305.350         nan 
      4.768         nan 
 
 A10  13897.0 
    453.960         nan 
    320.150         nan 
      4.772         nan 
 
 A10  15440.0 
    451.358         nan 
    333.800         nan 
      4.775         nan 
 
 A10  16983.0 
    464.049         nan 
    345.550         nan 
      4.772         nan 
 
 A10  18526.0 
    481.031         nan 
    357.200         nan 
      4.774         nan 
 
 A10  20069.0 
    468.901         nan 
    363.100         nan 
      4.772         nan 
 
 A10  21612.0 
    482.856         nan 
    374.700         nan 
      4.776         nan 
 
 A10  23155.0 
    472.893         nan 
    380.600         nan 
      4.779         nan 
 
 A10  24698.0 
    474.441         nan 
    389.650         nan 
      4.780         nan 
 
 A10  26241.0 
    486.727         nan 
    397.500         nan 
      4.782         nan 
 
 A10  27784.0 
    483.101         nan 
    404.050         nan 
      4.782         nan 
 
 A10  29327.0 
    485.436         nan 
    409.650         nan 
      4.781         nan 
 
 A10  30870.0 
    486.520         nan 
    415.550         nan 
      4.779         nan 
 
 A10  32413.0 
    483.505         nan 
    420.300         nan 
      4.784         nan 
 
 A10  33956.0 
    483.169         nan 
    423.950         nan 
      4.787         nan 
 
 A10  35499.0 
    488.214         nan 
    429.950         nan 
      4.785         nan 
 
 A10  37042.0 
    486.604         nan 
    433.300         nan 
      4.786         nan 
 
 A10  38585.0 
    486.783         nan 
    437.850         nan 
      4.786         nan 
 
 A10  40128.0 
    487.146         nan 
    440.300         nan 
      4.786         nan 
 
 A10  41671.0 
    487.725         nan 
    443.500         nan 
      4.786         nan 
 
 A10  43214.0 
    488.502         nan 
    447.500         nan 
      4.786         nan 
 
 A10  44757.0 
    491.004         nan 
    450.400         nan 
      4.787         nan 
 
 A10  46300.0 
        nan         nan 
        nan         nan 
        nan         nan 
 
 A11  10.0 
     26.075         nan 
      8.700         nan 
      3.048         nan 
 
 A11  1553.0 
    337.551         nan 
    187.850         nan 
      5.741         nan 
 
 A11  3096.0 
    424.173         nan 
    261.600         nan 
      5.825         nan 
 
 A11  4639.0 
    445.386         nan 
    308.750         nan 
      5.836         nan 
 
 A11  6182.0 
    483.799         nan 
    350.300         nan 
      5.866         nan 
 
 A11  7725.0 
    495.701         nan 
    380.800         nan 
      5.880         nan 
 
 A11  9268.0 
    506.781         nan 
    400.100         nan 
      5.884         nan 
 
 A11  10811.0 
    519.652         nan 
    417.950         nan 
      5.889         nan 
 
 A11  12354.0 
    522.918         nan 
    437.200         nan 
      5.897         nan 
 
 A11  13897.0 
    530.804         nan 
    448.250         nan 
      5.893         nan 
 
 A11  15440.0 
    542.258         nan 
    465.300         nan 
      5.905         nan 
 
 A11  16983.0 
    541.336         nan 
    472.600         nan 
      5.902         nan 
 
 A11  18526.0 
    543.081         nan 
    480.750         nan 
      5.902         nan 
 
 A11  20069.0 
    545.462         nan 
    485.950         nan 
      5.907         nan 
 
 A11  21612.0 
    545.599         nan 
    493.950         nan 
      5.913         nan 
 
 A11  23155.0 
    543.960         nan 
    498.700         nan 
      5.912         nan 
 
 A11  24698.0 
    552.222         nan 
    507.800         nan 
      5.916         nan 
 
 A11  26241.0 
    551.697         nan 
    510.150         nan 
      5.912         nan 
 
 A11  27784.0 
    555.845         nan 
    517.450         nan 
      5.914         nan 
 
 A11  29327.0 
    558.501         nan 
    521.000         nan 
      5.918         nan 
 
 A11  30870.0 
    552.065         nan 
    521.950         nan 
      5.917         nan 
 
 A11  32413.0 
    556.008         nan 
    525.800         nan 
      5.918         nan 
 
 A11  33956.0 
    554.933         nan 
    528.600         nan 
      5.920         nan 
 
 A11  35499.0 
    554.504         nan 
    530.350         nan 
      5.920         nan 
 
 A11  37042.0 
    557.903         nan 
    534.600         nan 
      5.922         nan 
 
 A11  38585.0 
    554.788         nan 
    534.800         nan 
      5.921         nan 
 
 A11  40128.0 
    559.701         nan 
    538.350         nan 
      5.922         nan 
 
 A11  41671.0 
    558.218         nan 
    539.500         nan 
      5.923         nan 
 
 A11  43214.0 
    559.722         nan 
    540.750         nan 
      5.921         nan 
 
 A11  44757.0 
    560.901         nan 
    542.500         nan 
      5.922         nan 
 
 A11  46300.0 
    562.278         nan 
    544.000         nan 
      5.922         nan 
 
 A12  10.0 
     27.400         nan 
      8.550         nan 
      3.003         nan 
 
 A12  1553.0 
    289.482         nan 
    150.600         nan 
      5.293         nan 
 
 A12  3096.0 
    385.600         nan 
    212.200         nan 
      5.350         nan 
 
 A12  4639.0 
    441.285         nan 
    261.300         nan 
      5.380         nan 
 
 A12  6182.0 
    480.039         nan 
    300.400         nan 
      5.414         nan 
 
 A12  7725.0 
    478.046         nan 
    328.850         nan 
      5.414         nan 
 
 A12  9268.0 
    493.466         nan 
    355.450         nan 
      5.421         nan 
 
 A12  10811.0 
    519.243         nan 
    379.350         nan 
      5.430         nan 
 
 A12  12354.0 
    519.861         nan 
    395.250         nan 
      5.430         nan 
 
 A12  13897.0 
    527.763         nan 
    412.450         nan 
      5.442         nan 
 
 A12  15440.0 
    536.400         nan 
    427.200         nan 
      5.436         nan 
 
 A12  16983.0 
    546.130         nan 
    441.100         nan 
      5.445         nan 
 
 A12  18526.0 
    541.036         nan 
    447.600         nan 
      5.441         nan 
 
 A12  20069.0 
    548.679         nan 
    459.950         nan 
      5.443         nan 
 
 A12  21612.0 
    556.770         nan 
    470.900         nan 
      5.444         nan 
 
 A12  23155.0 
    557.073         nan 
    479.000         nan 
      5.453         nan 
 
 A12  24698.0 
    553.125         nan 
    484.100         nan 
      5.448         nan 
 
 A12  26241.0 
    556.776         nan 
    491.250         nan 
      5.446         nan 
 
 A12  27784.0 
    560.951         nan 
    498.200         nan 
      5.448         nan 
 
 A12  29327.0 
    561.770         nan 
    505.800         nan 
      5.450         nan 
 
 A12  30870.0 
    559.708         nan 
    509.400         nan 
      5.454         nan 
 
 A12  32413.0 
    559.658         nan 
    513.700         nan 
      5.452         nan 
 
 A12  33956.0 
    565.976         nan 
    519.350         nan 
      5.451         nan 
 
 A12  35499.0 
    560.043         nan 
    522.500         nan 
      5.453         nan 
 
 A12  37042.0 
    561.724         nan 
    525.750         nan 
      5.455         nan 
 
 A12  38585.0 
    559.869         nan 
    529.250         nan 
      5.455         nan 
 
 A12  40128.0 
    560.906         nan 
    531.050         nan 
      5.455         nan 
 
 A12  41671.0 
    563.545         nan 
    534.650         nan 
      5.455         nan 
 
 A12  43214.0 
    561.163         nan 
    536.750         nan 
      5.455         nan 
 
 A12  44757.0 
    560.077         nan 
    538.700         nan 
      5.456         nan 
 
 A12  46300.0 
        nan         nan 
        nan         nan 
        nan         nan 
 
 A13  10.0 
     15.175         nan 
      7.450         nan 
      2.764         nan 
 
 A13  1553.0 
    119.208         nan 
     83.200         nan 
      4.842         nan 
 
 A13  3096.0 
    149.651         nan 
    100.950         nan 
      4.849         nan 
 
 A13  4639.0 
    177.379         nan 
    118.600         nan 
      4.881         nan 
 
 A13  6182.0 
    178.103         nan 
    126.650         nan 
      4.864         nan 
 
 A13  7725.0 
    193.800         nan 
    139.200         nan 
      4.875         nan 
 
 A13  9268.0 
    201.914         nan 
    147.650         nan 
      4.877         nan 
 
 A13  10811.0 
    204.416         nan 
    153.350         nan 
      4.881         nan 
 
 A13  12354.0 
    224.370         nan 
    159.100         nan 
      4.883         nan 
 
 A13  13897.0 
    222.277         nan 
    166.350         nan 
      4.883         nan 
 
 A13  15440.0 
    223.952         nan 
    173.150         nan 
      4.887         nan 
 
 A13  16983.0 
    219.613         nan 
    176.750         nan 
      4.883         nan 
 
 A13  18526.0 
    221.748         nan 
    181.350         nan 
      4.887         nan 
 
 A13  20069.0 
    227.671         nan 
    184.700         nan 
      4.888         nan 
 
 A13  21612.0 
    230.448         nan 
    189.050         nan 
      4.885         nan 
 
 A13  23155.0 
    234.783         nan 
    194.500         nan 
      4.889         nan 
 
 A13  24698.0 
    227.339         nan 
    195.400         nan 
      4.892         nan 
 
 A13  26241.0 
    226.276         nan 
    197.300         nan 
      4.887         nan 
 
 A13  27784.0 
    228.496         nan 
    199.200         nan 
      4.888         nan 
 
 A13  29327.0 
    232.204         nan 
    202.400         nan 
      4.887         nan 
 
 A13  30870.0 
    231.194         nan 
    204.350         nan 
      4.889         nan 
 
 A13  32413.0 
    230.697         nan 
    207.050         nan 
      4.889         nan 
 
 A13  33956.0 
    231.539         nan 
    208.450         nan 
      4.887         nan 
 
 A13  35499.0 
    229.884         nan 
    210.100         nan 
      4.891         nan 
 
 A13  37042.0 
    230.315         nan 
    212.300         nan 
      4.890         nan 
 
 A13  38585.0 
    231.225         nan 
    212.900         nan 
      4.890         nan 
 
 A13  40128.0 
    230.419         nan 
    214.600         nan 
      4.892         nan 
 
 A13  41671.0 
    232.358         nan 
    215.500         nan 
      4.889         nan 
 
 A13  43214.0 
    233.125         nan 
    217.450         nan 
      4.891         nan 
 
 A13  44757.0 
    230.639         nan 
    217.750         nan 
      4.890         nan 
 
 A13  46300.0 
        nan         nan 
        nan         nan 
        nan         nan 
 
 B01  10.0 
     25.650         nan 
      8.700         nan 
      3.054         nan 
 
 B01  1553.0 
    323.150         nan 
    197.050         nan 
      5.997         nan 
 
 B01  3096.0 
    376.215         nan 
    265.250         nan 
      6.078         nan 
 
 B01  4639.0 
    397.841         nan 
    309.100         nan 
      6.127         nan 
 
 B01  6182.0 
    404.897         nan 
    332.300         nan 
      6.137         nan 
 
 B01  7725.0 
    423.863         nan 
    355.700         nan 
      6.143         nan 
 
 B01  9268.0 
    432.382         nan 
    369.000         nan 
      6.148         nan 
 
 B01  10811.0 
    440.977         nan 
    384.600         nan 
      6.165         nan 
 
 B01  12354.0 
    436.616         nan 
    391.400         nan 
      6.170         nan 
 
 B01  13897.0 
    443.201         nan 
    400.900         nan 
      6.172         nan 
 
 B01  15440.0 
    443.941         nan 
    408.550         nan 
      6.170         nan 
 
 B01  16983.0 
    446.594         nan 
    415.300         nan 
      6.175         nan 
 
 B01  18526.0 
    447.682         nan 
    417.500         nan 
      6.175         nan 
 
 B01  20069.0 
    449.022         nan 
    421.600         nan 
      6.174         nan 
 
 B01  21612.0 
    446.604         nan 
    425.550         nan 
      6.182         nan 
 
 B01  23155.0 
    453.224         nan 
    430.150         nan 
      6.176         nan 
 
 B01  24698.0 
    452.140         nan 
    432.450         nan 
      6.179         nan 
 
 B01  26241.0 
    448.967         nan 
    434.150         nan 
      6.182         nan 
 
 B01  27784.0 
    449.349         nan 
    437.350         nan 
      6.183         nan 
 
 B01  29327.0 
    449.162         nan 
    437.550         nan 
      6.184         nan 
 
 B01  30870.0 
    446.542         nan 
    439.600         nan 
      6.182         nan 
 
 B01  32413.0 
    447.231         nan 
    441.150         nan 
      6.182         nan 
 
 B01  33956.0 
    448.170         nan 
    442.550         nan 
      6.184         nan 
 
 B01  35499.0 
    447.152         nan 
    443.400         nan 
      6.184         nan 
 
 B01  37042.0 
    446.676         nan 
    444.300         nan 
      6.184         nan 
 
 B01  38585.0 
    446.536         nan 
    444.900         nan 
      6.186         nan 
 
 B01  40128.0 
    446.865         nan 
    445.550         nan 
      6.185         nan 
 
 B01  41671.0 
    446.448         nan 
    445.600         nan 
      6.184         nan 
 
 B01  43214.0 
    446.293         nan 
    446.000         nan 
      6.186         nan 
 
 B01  44757.0 
    446.094         nan 
    446.000         nan 
      6.185         nan 
 
 B01  46300.0 
    446.000         nan 
    446.000         nan 
      6.186         nan 
 
 B02  10.0 
     19.760         nan 
      7.700         nan 
      2.799         nan 
 
 B02  1553.0 
    258.999         nan 
    169.750         nan 
      5.166         nan 
 
 B02  3096.0 
    290.576         nan 
    221.550         nan 
      5.224         nan 
 
 B02  4639.0 
    308.618         nan 
    254.350         nan 
      5.290         nan 
 
 B02  6182.0 
    314.501         nan 
    271.800         nan 
      5.278         nan 
 
 B02  7725.0 
    323.094         nan 
    285.800         nan 
      5.289         nan 
 
 B02  9268.0 
    329.045         nan 
    296.850         nan 
      5.290         nan 
 
 B02  10811.0 
    335.897         nan 
    305.950         nan 
      5.297         nan 
 
 B02  12354.0 
    336.000         nan 
    311.300         nan 
      5.305         nan 
 
 B02  13897.0 
    333.997         nan 
    314.400         nan 
      5.299         nan 
 
 B02  15440.0 
    337.622         nan 
    318.250         nan 
      5.297         nan 
 
 B02  16983.0 
    338.451         nan 
    322.900         nan 
      5.301         nan 
 
 B02  18526.0 
    337.802         nan 
    324.200         nan 
      5.311         nan 
 
 B02  20069.0 
    338.792         nan 
    327.550         nan 
      5.306         nan 
 
 B02  21612.0 
    343.051         nan 
    330.900         nan 
      5.306         nan 
 
 B02  23155.0 
    340.797         nan 
    330.850         nan 
      5.310         nan 
 
 B02  24698.0 
    340.784         nan 
    332.750         nan 
      5.310         nan 
 
 B02  26241.0 
    341.632         nan 
    334.050         nan 
      5.308         nan 
 
 B02  27784.0 
    340.574         nan 
    335.750         nan 
      5.311         nan 
 
 B02  29327.0 
    339.114         nan 
    335.200         nan 
      5.312         nan 
 
 B02  30870.0 
    339.532         nan 
    336.450         nan 
      5.312         nan 
 
 B02  32413.0 
    340.679         nan 
    337.400         nan 
      5.308         nan 
 
 B02  33956.0 
    338.699         nan 
    337.000         nan 
      5.311         nan 
 
 B02  35499.0 
    339.387         nan 
    338.100         nan 
      5.312         nan 
 
 B02  37042.0 
    339.660         nan 
    338.550         nan 
      5.314         nan 
 
 B02  38585.0 
    338.814         nan 
    338.250         nan 
      5.313         nan 
 
 B02  40128.0 
    339.152         nan 
    338.800         nan 
      5.315         nan 
 
 B02  41671.0 
    338.949         nan 
    338.800         nan 
      5.313         nan 
 
 B02  43214.0 
    339.081         nan 
    338.950         nan 
      5.314         nan 
 
 B02  44757.0 
    338.968         nan 
    338.950         nan 
      5.314         nan 
 
 B02  46300.0 
    339.000         nan 
    339.000         nan 
      5.313         nan 
 
 B03  10.0 
     26.400         nan 
      8.850         nan 
      3.084         nan 
 
 B03  1553.0 
    420.476         nan 
    287.150         nan 
      6.356         nan 
 
 B03  3096.0 
    483.050         nan 
    379.200         nan 
      6.494         nan 
 
 B03  4639.0 
    522.172         nan 
    428.550         nan 
      6.542         nan 
 
 B03  6182.0 
    535.578         nan 
    458.350         nan 
      6.556         nan 
 
 B03  7725.0 
    551.792         nan 
    482.150         nan 
      6.586         nan 
 
 B03  9268.0 
    558.203         nan 
    496.900         nan 
      6.579         nan 
 
 B03  10811.0 
    564.182         nan 
    513.800         nan 
      6.597         nan 
 
 B03  12354.0 
    578.665         nan 
    522.350         nan 
      6.592         nan 
 
 B03  13897.0 
    573.795         nan 
    529.500         nan 
      6.599         nan 
 
 B03  15440.0 
    587.887         nan 
    539.500         nan 
      6.609         nan 
 
 B03  16983.0 
    591.866         nan 
    545.800         nan 
      6.608         nan 
 
 B03  18526.0 
    596.707         nan 
    552.300         nan 
      6.609         nan 
 
 B03  20069.0 
    600.125         nan 
    556.100         nan 
      6.608         nan 
 
 B03  21612.0 
    607.703         nan 
    561.650         nan 
      6.615         nan 
 
 B03  23155.0 
    608.871         nan 
    565.650         nan 
      6.613         nan 
 
 B03  24698.0 
    615.347         nan 
    570.300         nan 
      6.615         nan 
 
 B03  26241.0 
    613.935         nan 
    573.350         nan 
      6.619         nan 
 
 B03  27784.0 
    623.669         nan 
    577.300         nan 
      6.620         nan 
 
 B03  29327.0 
    617.963         nan 
    577.700         nan 
      6.617         nan 
 
 B03  30870.0 
    626.295         nan 
    583.550         nan 
      6.621         nan 
 
 B03  32413.0 
    628.586         nan 
    585.800         nan 
      6.621         nan 
 
 B03  33956.0 
    624.772         nan 
    587.700         nan 
      6.622         nan 
 
 B03  35499.0 
    627.539         nan 
    589.200         nan 
      6.621         nan 
 
 B03  37042.0 
    628.241         nan 
    591.950         nan 
      6.623         nan 
 
 B03  38585.0 
    628.215         nan 
    593.950         nan 
      6.623         nan 
 
 B03  40128.0 
    628.147         nan 
    595.400         nan 
      6.621         nan 
 
 B03  41671.0 
    633.045         nan 
    598.250         nan 
      6.622         nan 
 
 B03  43214.0 
    632.983         nan 
    600.050         nan 
      6.624         nan 
 
 B03  44757.0 
    635.330         nan 
    601.750         nan 
      6.625         nan 
 
 B03  46300.0 
    634.794         nan 
    603.000         nan 
      6.625         nan 
 
 B04  10.0 
     21.325         nan 
      7.950         nan 
      2.866         nan 
 
 B04  1553.0 
    236.280         nan 
    148.700         nan 
      5.079         nan 
 
 B04  3096.0 
    306.666         nan 
    196.000         nan 
      5.134         nan 
 
 B04  4639.0 
    322.072         nan 
    226.950         nan 
      5.154         nan 
 
 B04  6182.0 
    343.894         nan 
    254.250         nan 
      5.166         nan 
 
 B04  7725.0 
    359.265         nan 
    272.150         nan 
      5.164         nan 
 
 B04  9268.0 
    363.720         nan 
    288.150         nan 
      5.186         nan 
 
 B04  10811.0 
    373.656         nan 
    301.650         nan 
      5.183         nan 
 
 B04  12354.0 
    374.147         nan 
    309.650         nan 
      5.182         nan 
 
 B04  13897.0 
    380.587         nan 
    321.100         nan 
      5.191         nan 
 
 B04  15440.0 
    390.064         nan 
    329.800         nan 
      5.193         nan 
 
 B04  16983.0 
    388.978         nan 
    336.550         nan 
      5.190         nan 
 
 B04  18526.0 
    388.941         nan 
    341.450         nan 
      5.196         nan 
 
 B04  20069.0 
    391.786         nan 
    345.200         nan 
      5.194         nan 
 
 B04  21612.0 
    392.218         nan 
    351.250         nan 
      5.197         nan 
 
 B04  23155.0 
    400.442         nan 
    357.750         nan 
      5.196         nan 
 
 B04  24698.0 
    398.091         nan 
    361.300         nan 
      5.199         nan 
 
 B04  26241.0 
    400.787         nan 
    364.100         nan 
      5.200         nan 
 
 B04  27784.0 
    405.560         nan 
    367.850         nan 
      5.199         nan 
 
 B04  29327.0 
    407.521         nan 
    371.900         nan 
      5.200         nan 
 
 B04  30870.0 
    404.192         nan 
    374.950         nan 
      5.200         nan 
 
 B04  32413.0 
    399.209         nan 
    375.450         nan 
      5.202         nan 
 
 B04  33956.0 
    402.404         nan 
    378.900         nan 
      5.202         nan 
 
 B04  35499.0 
    403.400         nan 
    380.750         nan 
      5.203         nan 
 
 B04  37042.0 
    403.689         nan 
    381.350         nan 
      5.202         nan 
 
 B04  38585.0 
    403.622         nan 
    384.000         nan 
      5.203         nan 
 
 B04  40128.0 
    403.239         nan 
    386.150         nan 
      5.206         nan 
 
 B04  41671.0 
    404.471         nan 
    388.000         nan 
      5.204         nan 
 
 B04  43214.0 
    402.986         nan 
    388.150         nan 
      5.202         nan 
 
 B04  44757.0 
    402.631         nan 
    389.950         nan 
      5.205         nan 
 
 B04  46300.0 
    402.601         nan 
    390.950         nan 
      5.205         nan 
 
 B05  10.0 
     26.950         nan 
      8.500         nan 
      2.989         nan 
 
 B05  1553.0 
    203.493         nan 
    136.500         nan 
      5.206         nan 
 
 B05  3096.0 
    251.866         nan 
    179.700         nan 
      5.262         nan 
 
 B05  4639.0 
    276.439         nan 
    209.850         nan 
      5.293         nan 
 
 B05  6182.0 
    294.487         nan 
    228.550         nan 
      5.300         nan 
 
 B05  7725.0 
    301.932         nan 
    242.800         nan 
      5.311         nan 
 
 B05  9268.0 
    299.781         nan 
    254.250         nan 
      5.307         nan 
 
 B05  10811.0 
    305.138         nan 
    263.400         nan 
      5.320         nan 
 
 B05  12354.0 
    311.323         nan 
    272.050         nan 
      5.320         nan 
 
 B05  13897.0 
    311.376         nan 
    278.400         nan 
      5.318         nan 
 
 B05  15440.0 
    320.308         nan 
    284.700         nan 
      5.318         nan 
 
 B05  16983.0 
    319.049         nan 
    288.650         nan 
      5.327         nan 
 
 B05  18526.0 
    324.613         nan 
    294.250         nan 
      5.325         nan 
 
 B05  20069.0 
    328.099         nan 
    298.550         nan 
      5.326         nan 
 
 B05  21612.0 
    323.844         nan 
    300.250         nan 
      5.326         nan 
 
 B05  23155.0 
    325.860         nan 
    302.000         nan 
      5.330         nan 
 
 B05  24698.0 
    328.656         nan 
    305.250         nan 
      5.334         nan 
 
 B05  26241.0 
    332.058         nan 
    307.650         nan 
      5.327         nan 
 
 B05  27784.0 
    328.185         nan 
    309.500         nan 
      5.328         nan 
 
 B05  29327.0 
    329.608         nan 
    311.050         nan 
      5.334         nan 
 
 B05  30870.0 
    330.188         nan 
    312.600         nan 
      5.335         nan 
 
 B05  32413.0 
    334.962         nan 
    314.550         nan 
      5.331         nan 
 
 B05  33956.0 
    332.838         nan 
    315.800         nan 
      5.334         nan 
 
 B05  35499.0 
    332.661         nan 
    316.850         nan 
      5.332         nan 
 
 B05  37042.0 
    333.321         nan 
    318.400         nan 
      5.333         nan 
 
 B05  38585.0 
    332.356         nan 
    318.700         nan 
      5.334         nan 
 
 B05  40128.0 
    333.807         nan 
    320.300         nan 
      5.333         nan 
 
 B05  41671.0 
    333.821         nan 
    321.000         nan 
      5.333         nan 
 
 B05  43214.0 
    334.785         nan 
    322.900         nan 
      5.334         nan 
 
 B05  44757.0 
    334.301         nan 
    323.350         nan 
      5.333         nan 
 
 B05  46300.0 
    334.166         nan 
    324.000         nan 
      5.334         nan 
 
 B06  10.0 
     11.542         nan 
      6.500         nan 
      2.484         nan 
 
 B06  1553.0 
    129.009         nan 
     83.700         nan 
      4.175         nan 
 
 B06  3096.0 
    159.724         nan 
    112.900         nan 
      4.235         nan 
 
 B06  4639.0 
    179.725         nan 
    131.400         nan 
      4.245         nan 
 
 B06  6182.0 
    179.607         nan 
    140.700         nan 
      4.244         nan 
 
 B06  7725.0 
    187.863         nan 
    152.700         nan 
      4.256         nan 
 
 B06  9268.0 
    195.133         nan 
    160.900         nan 
      4.257         nan 
 
 B06  10811.0 
    198.144         nan 
    167.150         nan 
      4.257         nan 
 
 B06  12354.0 
    193.595         nan 
    170.850         nan 
      4.265         nan 
 
 B06  13897.0 
    202.830         nan 
    175.000         nan 
      4.257         nan 
 
 B06  15440.0 
    203.849         nan 
    181.000         nan 
      4.267         nan 
 
 B06  16983.0 
    204.526         nan 
    183.800         nan 
      4.264         nan 
 
 B06  18526.0 
    202.017         nan 
    184.900         nan 
      4.268         nan 
 
 B06  20069.0 
    206.297         nan 
    188.950         nan 
      4.266         nan 
 
 B06  21612.0 
    204.542         nan 
    189.400         nan 
      4.266         nan 
 
 B06  23155.0 
    207.238         nan 
    192.050         nan 
      4.271         nan 
 
 B06  24698.0 
    206.909         nan 
    194.050         nan 
      4.272         nan 
 
 B06  26241.0 
    204.757         nan 
    194.300         nan 
      4.272         nan 
 
 B06  27784.0 
    205.419         nan 
    195.150         nan 
      4.266         nan 
 
 B06  29327.0 
    204.682         nan 
    197.150         nan 
      4.270         nan 
 
 B06  30870.0 
    206.278         nan 
    198.250         nan 
      4.269         nan 
 
 B06  32413.0 
    206.696         nan 
    199.950         nan 
      4.272         nan 
 
 B06  33956.0 
    205.786         nan 
    200.150         nan 
      4.272         nan 
 
 B06  35499.0 
    206.176         nan 
    200.350         nan 
      4.271         nan 
 
 B06  37042.0 
    206.421         nan 
    201.850         nan 
      4.273         nan 
 
 B06  38585.0 
    206.118         nan 
    201.900         nan 
      4.272         nan 
 
 B06  40128.0 
    206.675         nan 
    202.450         nan 
      4.271         nan 
 
 B06  41671.0 
    206.204         nan 
    202.800         nan 
      4.273         nan 
 
 B06  43214.0 
    206.646         nan 
    203.400         nan 
      4.273         nan 
 
 B06  44757.0 
    206.494         nan 
    203.450         nan 
      4.272         nan 
 
 B06  46300.0 
    206.393         nan 
    204.000         nan 
      4.272         nan 
 
 B07  10.0 
     19.585         nan 
      7.500         nan 
      2.754         nan 
 
 B07  1553.0 
    365.426         nan 
    194.350         nan 
      5.196         nan 
 
 B07  3096.0 
    478.774         nan 
    276.200         nan 
      5.288         nan 
 
 B07  4639.0 
    526.519         nan 
    333.250         nan 
      5.317         nan 
 
 B07  6182.0 
    554.288         nan 
    372.950         nan 
      5.330         nan 
 
 B07  7725.0 
    588.041         nan 
    409.950         nan 
      5.362         nan 
 
 B07  9268.0 
    595.200         nan 
    444.300         nan 
      5.371         nan 
 
 B07  10811.0 
    603.369         nan 
    461.900         nan 
      5.367         nan 
 
 B07  12354.0 
    624.162         nan 
    483.000         nan 
      5.375         nan 
 
 B07  13897.0 
    639.121         nan 
    505.300         nan 
      5.372         nan 
 
 B07  15440.0 
    642.792         nan 
    518.000         nan 
      5.383         nan 
 
 B07  16983.0 
    647.245         nan 
    533.600         nan 
      5.390         nan 
 
 B07  18526.0 
    657.001         nan 
    548.200         nan 
      5.391         nan 
 
 B07  20069.0 
    658.580         nan 
    557.400         nan 
      5.390         nan 
 
 B07  21612.0 
    663.647         nan 
    568.800         nan 
      5.391         nan 
 
 B07  23155.0 
    661.659         nan 
    576.000         nan 
      5.397         nan 
 
 B07  24698.0 
    670.033         nan 
    586.550         nan 
      5.402         nan 
 
 B07  26241.0 
    664.923         nan 
    592.850         nan 
      5.396         nan 
 
 B07  27784.0 
    666.547         nan 
    598.850         nan 
      5.401         nan 
 
 B07  29327.0 
    672.648         nan 
    608.250         nan 
      5.401         nan 
 
 B07  30870.0 
    671.942         nan 
    612.900         nan 
      5.405         nan 
 
 B07  32413.0 
    678.599         nan 
    617.400         nan 
      5.403         nan 
 
 B07  33956.0 
    678.934         nan 
    622.800         nan 
      5.405         nan 
 
 B07  35499.0 
    674.104         nan 
    626.350         nan 
      5.404         nan 
 
 B07  37042.0 
    677.612         nan 
    632.000         nan 
      5.406         nan 
 
 B07  38585.0 
    675.150         nan 
    634.800         nan 
      5.404         nan 
 
 B07  40128.0 
    678.134         nan 
    637.850         nan 
      5.407         nan 
 
 B07  41671.0 
    675.284         nan 
    640.700         nan 
      5.407         nan 
 
 B07  43214.0 
    677.970         nan 
    644.600         nan 
      5.407         nan 
 
 B07  44757.0 
    674.857         nan 
    646.600         nan 
      5.406         nan 
 
 B07  46300.0 
        nan         nan 
        nan         nan 
        nan         nan 
 
 B08  10.0 
     31.175         nan 
      9.050         nan 
      3.128         nan 
 
 B08  1553.0 
    710.944         nan 
    401.450         nan 
      7.061         nan 
 
 B08  3096.0 
    815.254         nan 
    561.900         nan 
      7.230         nan 
 
 B08  4639.0 
    848.638         nan 
    660.450         nan 
      7.290         nan 
 
 B08  6182.0 
    875.559         nan 
    727.350         nan 
      7.322         nan 
 
 B08  7725.0 
    900.633         nan 
    772.850         nan 
      7.347         nan 
 
 B08  9268.0 
    907.823         nan 
    807.200         nan 
      7.354         nan 
 
 B08  10811.0 
    914.207         nan 
    829.200         nan 
      7.364         nan 
 
 B08  12354.0 
    920.602         nan 
    851.150         nan 
      7.390         nan 
 
 B08  13897.0 
    928.232         nan 
    866.250         nan 
      7.381         nan 
 
 B08  15440.0 
    926.439         nan 
    877.300         nan 
      7.392         nan 
 
 B08  16983.0 
    928.087         nan 
    883.000         nan 
      7.397         nan 
 
 B08  18526.0 
    937.147         nan 
    894.000         nan 
      7.404         nan 
 
 B08  20069.0 
    938.783         nan 
    900.200         nan 
      7.401         nan 
 
 B08  21612.0 
    942.375         nan 
    906.400         nan 
      7.411         nan 
 
 B08  23155.0 
    944.768         nan 
    911.700         nan 
      7.409         nan 
 
 B08  24698.0 
    948.343         nan 
    915.850         nan 
      7.412         nan 
 
 B08  26241.0 
    950.034         nan 
    921.350         nan 
      7.413         nan 
 
 B08  27784.0 
    946.051         nan 
    921.500         nan 
      7.415         nan 
 
 B08  29327.0 
    955.286         nan 
    927.300         nan 
      7.418         nan 
 
 B08  30870.0 
    953.584         nan 
    928.750         nan 
      7.417         nan 
 
 B08  32413.0 
    950.501         nan 
    930.150         nan 
      7.420         nan 
 
 B08  33956.0 
    958.533         nan 
    933.100         nan 
      7.420         nan 
 
 B08  35499.0 
    955.663         nan 
    934.550         nan 
      7.419         nan 
 
 B08  37042.0 
    959.841         nan 
    937.450         nan 
      7.421         nan 
 
 B08  38585.0 
    959.969         nan 
    938.600         nan 
      7.421         nan 
 
 B08  40128.0 
    963.149         nan 
    940.350         nan 
      7.424         nan 
 
 B08  41671.0 
    964.576         nan 
    942.300         nan 
      7.423         nan 
 
 B08  43214.0 
    966.445         nan 
    942.800         nan 
      7.425         nan 
 
 B08  44757.0 
    968.959         nan 
    944.600         nan 
      7.424         nan 
 
 B08  46300.0 
    970.667         nan 
    946.000         nan 
      7.425         nan 
 
 B09  10.0 
     25.450         nan 
      8.550         nan 
      3.007         nan 
 
 B09  1553.0 
    309.520         nan 
    174.550         nan 
      5.720         nan 
 
 B09  3096.0 
    414.052         nan 
    245.600         nan 
      5.801         nan 
 
 B09  4639.0 
    448.920         nan 
    291.150         nan 
      5.822         nan 
 
 B09  6182.0 
    526.753         nan 
    331.450         nan 
      5.846         nan 
 
 B09  7725.0 
    526.586         nan 
    356.200         nan 
      5.855         nan 
 
 B09  9268.0 
    561.287         nan 
    388.800         nan 
      5.873         nan 
 
 B09  10811.0 
    573.968         nan 
    405.150         nan 
      5.880         nan 
 
 B09  12354.0 
    591.287         nan 
    427.850         nan 
      5.875         nan 
 
 B09  13897.0 
    589.996         nan 
    444.000         nan 
      5.886         nan 
 
 B09  15440.0 
    606.293         nan 
    459.450         nan 
      5.889         nan 
 
 B09  16983.0 
    619.998         nan 
    475.150         nan 
      5.888         nan 
 
 B09  18526.0 
    625.802         nan 
    489.200         nan 
      5.892         nan 
 
 B09  20069.0 
    634.954         nan 
    502.550         nan 
      5.894         nan 
 
 B09  21612.0 
    634.708         nan 
    510.400         nan 
      5.897         nan 
 
 B09  23155.0 
    638.900         nan 
    522.800         nan 
      5.899         nan 
 
 B09  24698.0 
    655.083         nan 
    535.550         nan 
      5.902         nan 
 
 B09  26241.0 
    647.386         nan 
    543.200         nan 
      5.900         nan 
 
 B09  27784.0 
    642.254         nan 
    549.350         nan 
      5.904         nan 
 
 B09  29327.0 
    652.513         nan 
    559.650         nan 
      5.901         nan 
 
 B09  30870.0 
    648.872         nan 
    564.100         nan 
      5.903         nan 
 
 B09  32413.0 
    645.293         nan 
    568.850         nan 
      5.905         nan 
 
 B09  33956.0 
    651.364         nan 
    578.150         nan 
      5.907         nan 
 
 B09  35499.0 
    651.752         nan 
    582.300         nan 
      5.907         nan 
 
 B09  37042.0 
    644.143         nan 
    586.450         nan 
      5.909         nan 
 
 B09  38585.0 
    645.663         nan 
    590.350         nan 
      5.907         nan 
 
 B09  40128.0 
    644.170         nan 
    595.200         nan 
      5.908         nan 
 
 B09  41671.0 
    642.726         nan 
    599.950         nan 
      5.910         nan 
 
 B09  43214.0 
    642.215         nan 
    603.800         nan 
      5.910         nan 
 
 B09  44757.0 
    639.622         nan 
    606.350         nan 
      5.910         nan 
 
 B09  46300.0 
        nan         nan 
        nan         nan 
        nan         nan 
 
 B10  10.0 
     11.717         nan 
      6.050         nan 
      2.355         nan 
 
 B10  1553.0 
    162.420         nan 
     91.600         nan 
      3.786         nan 
 
 B10  3096.0 
    199.462         nan 
    122.350         nan 
      3.802         nan 
 
 B10  4639.0 
    223.711         nan 
    147.150         nan 
      3.813         nan 
 
 B10  6182.0 
    234.353         nan 
    161.100         nan 
      3.822         nan 
 
 B10  7725.0 
    246.529         nan 
    174.800         nan 
      3.814         nan 
 
 B10  9268.0 
    257.838         nan 
    188.800         nan 
      3.831         nan 
 
 B10  10811.0 
    275.462         nan 
    198.350         nan 
      3.839         nan 
 
 B10  12354.0 
    280.012         nan 
    206.450         nan 
      3.838         nan 
 
 B10  13897.0 
    282.757         nan 
    216.850         nan 
      3.846         nan 
 
 B10  15440.0 
    300.006         nan 
    224.700         nan 
      3.843         nan 
 
 B10  16983.0 
    292.657         nan 
    229.200         nan 
      3.845         nan 
 
 B10  18526.0 
    290.710         nan 
    233.250         nan 
      3.846         nan 
 
 B10  20069.0 
    299.672         nan 
    241.500         nan 
      3.844         nan 
 
 B10  21612.0 
    302.795         nan 
    246.150         nan 
      3.853         nan 
 
 B10  23155.0 
    299.232         nan 
    249.400         nan 
      3.849         nan 
 
 B10  24698.0 
    306.505         nan 
    253.950         nan 
      3.854         nan 
 
 B10  26241.0 
    306.703         nan 
    258.600         nan 
      3.852         nan 
 
 B10  27784.0 
    307.653         nan 
    260.700         nan 
      3.852         nan 
 
 B10  29327.0 
    309.012         nan 
    266.000         nan 
      3.853         nan 
 
 B10  30870.0 
    301.291         nan 
    267.450         nan 
      3.852         nan 
 
 B10  32413.0 
    305.888         nan 
    270.650         nan 
      3.849         nan 
 
 B10  33956.0 
    304.022         nan 
    273.550         nan 
      3.856         nan 
 
 B10  35499.0 
    305.442         nan 
    274.950         nan 
      3.848         nan 
 
 B10  37042.0 
    306.511         nan 
    278.400         nan 
      3.852         nan 
 
 B10  38585.0 
    304.608         nan 
    279.400         nan 
      3.852         nan 
 
 B10  40128.0 
    305.814         nan 
    282.450         nan 
      3.854         nan 
 
 B10  41671.0 
    304.761         nan 
    283.600         nan 
      3.853         nan 
 
 B10  43214.0 
    303.013         nan 
    285.050         nan 
      3.854         nan 
 
 B10  44757.0 
    303.521         nan 
    286.250         nan 
      3.855         nan 
 
 B10  46300.0 
    303.945         nan 
    287.950         nan 
      3.854         nan 
 
  SampleID  Seqs/Sample 
 chao1 Ave.  chao1 Err. 
 observed_species Ave.  observed_species Err. 
 shannon Ave.  shannon Err. 
 
 
 A01  10.0 
     13.460         nan 
      7.200         nan 
      2.710         nan 
 
 A01  1553.0 
    143.566         nan 
     83.500         nan 
      4.309         nan 
 
 A01  3096.0 
    183.101         nan 
    111.200         nan 
      4.320         nan 
 
 A01  4639.0 
    219.139         nan 
    134.050         nan 
      4.350         nan 
 
 A01  6182.0 
    231.738         nan 
    151.150         nan 
      4.359         nan 
 
 A01  7725.0 
    241.876         nan 
    161.150         nan 
      4.351         nan 
 
 A01  9268.0 
    246.748         nan 
    174.500         nan 
      4.360         nan 
 
 A01  10811.0 
    256.697         nan 
    184.550         nan 
      4.366         nan 
 
 A01  12354.0 
    269.593         nan 
    194.600         nan 
      4.369         nan 
 
 A01  13897.0 
    266.605         nan 
    199.500         nan 
      4.364         nan 
 
 A01  15440.0 
    266.184         nan 
    205.250         nan 
      4.366         nan 
 
 A01  16983.0 
    273.905         nan 
    215.750         nan 
      4.372         nan 
 
 A01  18526.0 
    265.761         nan 
    218.600         nan 
      4.376         nan 
 
 A01  20069.0 
    271.658         nan 
    224.900         nan 
      4.375         nan 
 
 A01  21612.0 
    267.841         nan 
    228.900         nan 
      4.376         nan 
 
 A01  23155.0 
    271.397         nan 
    232.900         nan 
      4.375         nan 
 
 A01  24698.0 
    272.454         nan 
    236.100         nan 
      4.374         nan 
 
 A01  26241.0 
    275.571         nan 
    240.750         nan 
      4.376         nan 
 
 A01  27784.0 
    275.992         nan 
    245.050         nan 
      4.379         nan 
 
 A01  29327.0 
    274.484         nan 
    246.700         nan 
      4.379         nan 
 
 A01  30870.0 
    269.618         nan 
    247.150         nan 
      4.377         nan 
 
 A01  32413.0 
    266.919         nan 
    250.500         nan 
      4.377         nan 
 
 A01  33956.0 
    267.089         nan 
    252.350         nan 
      4.378         nan 
 
 A01  35499.0 
    265.820         nan 
    254.000         nan 
      4.378         nan 
 
 A01  37042.0 
    265.929         nan 
    255.800         nan 
      4.380         nan 
 
 A01  38585.0 
    264.324         nan 
    256.750         nan 
      4.378         nan 
 
 A01  40128.0 
    263.007         nan 
    258.000         nan 
      4.380         nan 
 
 A01  41671.0 
    263.381         nan 
    259.250         nan 
      4.380         nan 
 
 A01  43214.0 
    263.345         nan 
    260.250         nan 
      4.380         nan 
 
 A01  44757.0 
    262.140         nan 
    260.650         nan 
      4.381         nan 
 
 A01  46300.0 
        nan         nan 
        nan         nan 
        nan         nan 
 
 A02  10.0 
     16.800         nan 
      7.500         nan 
      2.768         nan 
 
 A02  1553.0 
     98.090         nan 
     66.100         nan 
      4.538         nan 
 
 A02  3096.0 
    103.836         nan 
     79.100         nan 
      4.560         nan 
 
 A02  4639.0 
    126.607         nan 
     90.650         nan 
      4.577         nan 
 
 A02  6182.0 
    135.829         nan 
     97.250         nan 
      4.572         nan 
 
 A02  7725.0 
    138.571         nan 
    104.100         nan 
      4.575         nan 
 
 A02  9268.0 
    153.311         nan 
    109.550         nan 
      4.588         nan 
 
 A02  10811.0 
    147.420         nan 
    113.000         nan 
      4.581         nan 
 
 A02  12354.0 
    152.059         nan 
    119.250         nan 
      4.583         nan 
 
 A02  13897.0 
    150.117         nan 
    119.950         nan 
      4.582         nan 
 
 A02  15440.0 
    151.888         nan 
    123.850         nan 
      4.578         nan 
 
 A02  16983.0 
    153.484         nan 
    127.850         nan 
      4.583         nan 
 
 A02  18526.0 
    152.399         nan 
    129.800         nan 
      4.582         nan 
 
 A02  20069.0 
    155.152         nan 
    134.350         nan 
      4.589         nan 
 
 A02  21612.0 
    152.857         nan 
    135.350         nan 
      4.588         nan 
 
 A02  23155.0 
    154.124         nan 
    137.100         nan 
      4.588         nan 
 
 A02  24698.0 
    151.876         nan 
    137.350         nan 
      4.584         nan 
 
 A02  26241.0 
    151.355         nan 
    140.300         nan 
      4.586         nan 
 
 A02  27784.0 
    152.822         nan 
    142.500         nan 
      4.589         nan 
 
 A02  29327.0 
    151.477         nan 
    142.900         nan 
      4.589         nan 
 
 A02  30870.0 
    152.283         nan 
    143.950         nan 
      4.591         nan 
 
 A02  32413.0 
    150.937         nan 
    144.550         nan 
      4.589         nan 
 
 A02  33956.0 
    151.101         nan 
    146.400         nan 
      4.588         nan 
 
 A02  35499.0 
    151.072         nan 
    147.300         nan 
      4.589         nan 
 
 A02  37042.0 
    150.620         nan 
    147.550         nan 
      4.588         nan 
 
 A02  38585.0 
    149.167         nan 
    147.850         nan 
      4.588         nan 
 
 A02  40128.0 
    149.790         nan 
    148.350         nan 
      4.588         nan 
 
 A02  41671.0 
    149.351         nan 
    148.650         nan 
      4.588         nan 
 
 A02  43214.0 
    149.050         nan 
    148.750         nan 
      4.588         nan 
 
 A02  44757.0 
    149.065         nan 
    149.000         nan 
      4.589         nan 
 
 A02  46300.0 
        nan         nan 
        nan         nan 
        nan         nan 
 
 A03  10.0 
     23.225         nan 
      8.600         nan 
      3.028         nan 
 
 A03  1553.0 
    354.040         nan 
    208.000         nan 
      5.726         nan 
 
 A03  3096.0 
    404.240         nan 
    281.600         nan 
      5.787         nan 
 
 A03  4639.0 
    415.803         nan 
    324.300         nan 
      5.841         nan 
 
 A03  6182.0 
    426.677         nan 
    351.700         nan 
      5.865         nan 
 
 A03  7725.0 
    439.880         nan 
    373.000         nan 
      5.854         nan 
 
 A03  9268.0 
    452.576         nan 
    389.000         nan 
      5.862         nan 
 
 A03  10811.0 
    454.955         nan 
    401.000         nan 
      5.882         nan 
 
 A03  12354.0 
    462.824         nan 
    413.200         nan 
      5.872         nan 
 
 A03  13897.0 
    471.267         nan 
    421.750         nan 
      5.870         nan 
 
 A03  15440.0 
    468.377         nan 
    427.650         nan 
      5.875         nan 
 
 A03  16983.0 
    464.227         nan 
    433.250         nan 
      5.888         nan 
 
 A03  18526.0 
    470.228         nan 
    438.550         nan 
      5.881         nan 
 
 A03  20069.0 
    477.009         nan 
    445.350         nan 
      5.889         nan 
 
 A03  21612.0 
    478.131         nan 
    448.750         nan 
      5.891         nan 
 
 A03  23155.0 
    472.750         nan 
    450.050         nan 
      5.893         nan 
 
 A03  24698.0 
    477.940         nan 
    454.150         nan 
      5.890         nan 
 
 A03  26241.0 
    479.128         nan 
    457.550         nan 
      5.888         nan 
 
 A03  27784.0 
    476.269         nan 
    459.250         nan 
      5.895         nan 
 
 A03  29327.0 
    483.202         nan 
    462.150         nan 
      5.897         nan 
 
 A03  30870.0 
    479.549         nan 
    462.650         nan 
      5.898         nan 
 
 A03  32413.0 
    478.138         nan 
    463.950         nan 
      5.896         nan 
 
 A03  33956.0 
    478.809         nan 
    465.450         nan 
      5.897         nan 
 
 A03  35499.0 
    480.029         nan 
    467.700         nan 
      5.895         nan 
 
 A03  37042.0 
    480.141         nan 
    468.500         nan 
      5.896         nan 
 
 A03  38585.0 
    481.266         nan 
    469.850         nan 
      5.898         nan 
 
 A03  40128.0 
    481.598         nan 
    470.800         nan 
      5.900         nan 
 
 A03  41671.0 
    481.240         nan 
    471.650         nan 
      5.898         nan 
 
 A03  43214.0 
    481.833         nan 
    472.150         nan 
      5.898         nan 
 
 A03  44757.0 
    481.720         nan 
    473.150         nan 
      5.898         nan 
 
 A03  46300.0 
    482.762         nan 
    474.000         nan 
      5.899         nan 
 
 A04  10.0 
     22.275         nan 
      8.000         nan 
      2.873         nan 
 
 A04  1553.0 
    261.024         nan 
    162.900         nan 
      5.250         nan 
 
 A04  3096.0 
    287.134         nan 
    213.350         nan 
      5.311         nan 
 
 A04  4639.0 
    315.337         nan 
    244.000         nan 
      5.343         nan 
 
 A04  6182.0 
    317.813         nan 
    262.850         nan 
      5.348         nan 
 
 A04  7725.0 
    341.689         nan 
    284.450         nan 
      5.353         nan 
 
 A04  9268.0 
    340.222         nan 
    293.050         nan 
      5.361         nan 
 
 A04  10811.0 
    355.262         nan 
    305.100         nan 
      5.377         nan 
 
 A04  12354.0 
    358.778         nan 
    314.700         nan 
      5.378         nan 
 
 A04  13897.0 
    362.498         nan 
    321.100         nan 
      5.379         nan 
 
 A04  15440.0 
    365.593         nan 
    327.250         nan 
      5.371         nan 
 
 A04  16983.0 
    365.968         nan 
    332.000         nan 
      5.380         nan 
 
 A04  18526.0 
    367.835         nan 
    335.400         nan 
      5.382         nan 
 
 A04  20069.0 
    371.082         nan 
    339.800         nan 
      5.383         nan 
 
 A04  21612.0 
    374.429         nan 
    344.950         nan 
      5.387         nan 
 
 A04  23155.0 
    374.900         nan 
    347.350         nan 
      5.384         nan 
 
 A04  24698.0 
    375.540         nan 
    350.100         nan 
      5.388         nan 
 
 A04  26241.0 
    374.762         nan 
    352.450         nan 
      5.388         nan 
 
 A04  27784.0 
    377.844         nan 
    356.150         nan 
      5.391         nan 
 
 A04  29327.0 
    377.822         nan 
    358.650         nan 
      5.386         nan 
 
 A04  30870.0 
    378.494         nan 
    359.050         nan 
      5.387         nan 
 
 A04  32413.0 
    374.710         nan 
    361.050         nan 
      5.388         nan 
 
 A04  33956.0 
    374.578         nan 
    362.250         nan 
      5.388         nan 
 
 A04  35499.0 
    375.072         nan 
    363.500         nan 
      5.388         nan 
 
 A04  37042.0 
    375.091         nan 
    364.000         nan 
      5.387         nan 
 
 A04  38585.0 
    373.100         nan 
    365.500         nan 
      5.390         nan 
 
 A04  40128.0 
    372.383         nan 
    366.050         nan 
      5.388         nan 
 
 A04  41671.0 
    371.961         nan 
    367.450         nan 
      5.390         nan 
 
 A04  43214.0 
    371.554         nan 
    367.800         nan 
      5.389         nan 
 
 A04  44757.0 
    370.929         nan 
    368.500         nan 
      5.390         nan 
 
 A04  46300.0 
    370.712         nan 
    369.000         nan 
      5.390         nan 
 
 A05  10.0 
     10.067         nan 
      6.350         nan 
      2.453         nan 
 
 A05  1553.0 
    106.510         nan 
     58.900         nan 
      3.809         nan 
 
 A05  3096.0 
    118.318         nan 
     75.150         nan 
      3.813         nan 
 
 A05  4639.0 
    127.828         nan 
     88.100         nan 
      3.833         nan 
 
 A05  6182.0 
    133.552         nan 
     94.350         nan 
      3.820         nan 
 
 A05  7725.0 
    146.678         nan 
    104.950         nan 
      3.833         nan 
 
 A05  9268.0 
    146.871         nan 
    111.350         nan 
      3.835         nan 
 
 A05  10811.0 
    152.116         nan 
    117.750         nan 
      3.837         nan 
 
 A05  12354.0 
    153.553         nan 
    120.150         nan 
      3.841         nan 
 
 A05  13897.0 
    156.110         nan 
    127.650         nan 
      3.839         nan 
 
 A05  15440.0 
    158.781         nan 
    130.250         nan 
      3.838         nan 
 
 A05  16983.0 
    159.438         nan 
    133.550         nan 
      3.842         nan 
 
 A05  18526.0 
    159.567         nan 
    136.100         nan 
      3.839         nan 
 
 A05  20069.0 
    157.719         nan 
    139.200         nan 
      3.844         nan 
 
 A05  21612.0 
    157.939         nan 
    140.800         nan 
      3.841         nan 
 
 A05  23155.0 
    158.607         nan 
    143.400         nan 
      3.838         nan 
 
 A05  24698.0 
    162.023         nan 
    145.150         nan 
      3.844         nan 
 
 A05  26241.0 
    157.573         nan 
    146.950         nan 
      3.847         nan 
 
 A05  27784.0 
    158.990         nan 
    148.050         nan 
      3.845         nan 
 
 A05  29327.0 
    159.099         nan 
    149.700         nan 
      3.844         nan 
 
 A05  30870.0 
    158.129         nan 
    150.050         nan 
      3.844         nan 
 
 A05  32413.0 
    156.594         nan 
    151.300         nan 
      3.844         nan 
 
 A05  33956.0 
    157.779         nan 
    152.800         nan 
      3.845         nan 
 
 A05  35499.0 
    156.781         nan 
    153.350         nan 
      3.846         nan 
 
 A05  37042.0 
    155.625         nan 
    153.400         nan 
      3.845         nan 
 
 A05  38585.0 
    155.604         nan 
    153.950         nan 
      3.846         nan 
 
 A05  40128.0 
    155.410         nan 
    154.350         nan 
      3.846         nan 
 
 A05  41671.0 
    155.375         nan 
    154.800         nan 
      3.846         nan 
 
 A05  43214.0 
    155.254         nan 
    154.950         nan 
      3.846         nan 
 
 A05  44757.0 
    155.108         nan 
    155.000         nan 
      3.846         nan 
 
 A05  46300.0 
    155.000         nan 
    155.000         nan 
      3.846         nan 
 
 A06  10.0 
     18.225         nan 
      7.700         nan 
      2.822         nan 
 
 A06  1553.0 
    216.990         nan 
    138.200         nan 
      5.063         nan 
 
 A06  3096.0 
    272.482         nan 
    181.450         nan 
      5.120         nan 
 
 A06  4639.0 
    299.225         nan 
    211.100         nan 
      5.130         nan 
 
 A06  6182.0 
    308.372         nan 
    233.100         nan 
      5.140         nan 
 
 A06  7725.0 
    323.732         nan 
    251.300         nan 
      5.156         nan 
 
 A06  9268.0 
    326.919         nan 
    263.950         nan 
      5.160         nan 
 
 A06  10811.0 
    332.098         nan 
    274.100         nan 
      5.162         nan 
 
 A06  12354.0 
    340.432         nan 
    284.000         nan 
      5.169         nan 
 
 A06  13897.0 
    347.642         nan 
    295.950         nan 
      5.171         nan 
 
 A06  15440.0 
    340.408         nan 
    298.500         nan 
      5.172         nan 
 
 A06  16983.0 
    346.765         nan 
    306.750         nan 
      5.176         nan 
 
 A06  18526.0 
    346.982         nan 
    310.500         nan 
      5.172         nan 
 
 A06  20069.0 
    347.487         nan 
    316.150         nan 
      5.181         nan 
 
 A06  21612.0 
    354.439         nan 
    319.550         nan 
      5.180         nan 
 
 A06  23155.0 
    350.907         nan 
    322.100         nan 
      5.177         nan 
 
 A06  24698.0 
    352.456         nan 
    327.000         nan 
      5.175         nan 
 
 A06  26241.0 
    348.673         nan 
    329.350         nan 
      5.181         nan 
 
 A06  27784.0 
    351.936         nan 
    331.550         nan 
      5.177         nan 
 
 A06  29327.0 
    354.258         nan 
    335.850         nan 
      5.180         nan 
 
 A06  30870.0 
    351.654         nan 
    335.650         nan 
      5.180         nan 
 
 A06  32413.0 
    351.768         nan 
    338.150         nan 
      5.180         nan 
 
 A06  33956.0 
    351.582         nan 
    339.850         nan 
      5.181         nan 
 
 A06  35499.0 
    352.211         nan 
    341.050         nan 
      5.184         nan 
 
 A06  37042.0 
    351.828         nan 
    341.850         nan 
      5.183         nan 
 
 A06  38585.0 
    351.376         nan 
    343.600         nan 
      5.184         nan 
 
 A06  40128.0 
    351.341         nan 
    344.450         nan 
      5.184         nan 
 
 A06  41671.0 
    349.960         nan 
    345.550         nan 
      5.183         nan 
 
 A06  43214.0 
    349.491         nan 
    345.850         nan 
      5.186         nan 
 
 A06  44757.0 
    349.074         nan 
    346.600         nan 
      5.185         nan 
 
 A06  46300.0 
    348.665         nan 
    346.950         nan 
      5.184         nan 
 
 A07  10.0 
     18.250         nan 
      8.050         nan 
      2.911         nan 
 
 A07  1553.0 
    171.159         nan 
    106.150         nan 
      5.147         nan 
 
 A07  3096.0 
    222.244         nan 
    138.800         nan 
      5.205         nan 
 
 A07  4639.0 
    240.379         nan 
    160.250         nan 
      5.212         nan 
 
 A07  6182.0 
    276.849         nan 
    177.100         nan 
      5.217         nan 
 
 A07  7725.0 
    279.056         nan 
    189.900         nan 
      5.219         nan 
 
 A07  9268.0 
    297.130         nan 
    203.150         nan 
      5.217         nan 
 
 A07  10811.0 
    298.418         nan 
    211.550         nan 
      5.223         nan 
 
 A07  12354.0 
    301.960         nan 
    223.550         nan 
      5.230         nan 
 
 A07  13897.0 
    296.278         nan 
    231.150         nan 
      5.236         nan 
 
 A07  15440.0 
    301.236         nan 
    240.200         nan 
      5.230         nan 
 
 A07  16983.0 
    313.601         nan 
    249.300         nan 
      5.232         nan 
 
 A07  18526.0 
    311.381         nan 
    250.100         nan 
      5.230         nan 
 
 A07  20069.0 
    313.954         nan 
    258.850         nan 
      5.237         nan 
 
 A07  21612.0 
    311.018         nan 
    263.300         nan 
      5.234         nan 
 
 A07  23155.0 
    315.783         nan 
    270.150         nan 
      5.239         nan 
 
 A07  24698.0 
    312.240         nan 
    273.000         nan 
      5.237         nan 
 
 A07  26241.0 
    310.858         nan 
    276.700         nan 
      5.239         nan 
 
 A07  27784.0 
    315.036         nan 
    279.650         nan 
      5.238         nan 
 
 A07  29327.0 
    313.750         nan 
    284.000         nan 
      5.239         nan 
 
 A07  30870.0 
    310.224         nan 
    286.800         nan 
      5.237         nan 
 
 A07  32413.0 
    310.285         nan 
    289.500         nan 
      5.240         nan 
 
 A07  33956.0 
    311.160         nan 
    292.200         nan 
      5.236         nan 
 
 A07  35499.0 
    307.911         nan 
    293.350         nan 
      5.241         nan 
 
 A07  37042.0 
    308.393         nan 
    295.700         nan 
      5.241         nan 
 
 A07  38585.0 
    304.972         nan 
    296.450         nan 
      5.242         nan 
 
 A07  40128.0 
    305.507         nan 
    297.850         nan 
      5.241         nan 
 
 A07  41671.0 
    305.137         nan 
    299.400         nan 
      5.243         nan 
 
 A07  43214.0 
    304.780         nan 
    300.900         nan 
      5.242         nan 
 
 A07  44757.0 
    304.382         nan 
    301.650         nan 
      5.241         nan 
 
 A07  46300.0 
    303.364         nan 
    302.000         nan 
      5.242         nan 
 
 A08  10.0 
     20.875         nan 
      8.150         nan 
      2.929         nan 
 
 A08  1553.0 
    293.045         nan 
    138.950         nan 
      5.196         nan 
 
 A08  3096.0 
    377.470         nan 
    200.500         nan 
      5.244         nan 
 
 A08  4639.0 
    440.541         nan 
    249.450         nan 
      5.280         nan 
 
 A08  6182.0 
    445.577         nan 
    280.600         nan 
      5.297         nan 
 
 A08  7725.0 
    493.514         nan 
    311.350         nan 
      5.299         nan 
 
 A08  9268.0 
    496.078         nan 
    340.400         nan 
      5.308         nan 
 
 A08  10811.0 
    499.195         nan 
    358.850         nan 
      5.326         nan 
 
 A08  12354.0 
    508.754         nan 
    377.750         nan 
      5.322         nan 
 
 A08  13897.0 
    510.334         nan 
    393.200         nan 
      5.325         nan 
 
 A08  15440.0 
    512.669         nan 
    409.050         nan 
      5.333         nan 
 
 A08  16983.0 
    517.998         nan 
    422.100         nan 
      5.333         nan 
 
 A08  18526.0 
    520.362         nan 
    434.100         nan 
      5.334         nan 
 
 A08  20069.0 
    531.591         nan 
    447.550         nan 
      5.338         nan 
 
 A08  21612.0 
    529.702         nan 
    456.650         nan 
      5.338         nan 
 
 A08  23155.0 
    533.185         nan 
    461.250         nan 
      5.338         nan 
 
 A08  24698.0 
    524.230         nan 
    470.050         nan 
      5.342         nan 
 
 A08  26241.0 
    532.521         nan 
    477.900         nan 
      5.343         nan 
 
 A08  27784.0 
    528.560         nan 
    482.900         nan 
      5.343         nan 
 
 A08  29327.0 
    528.124         nan 
    486.500         nan 
      5.343         nan 
 
 A08  30870.0 
    525.610         nan 
    492.500         nan 
      5.344         nan 
 
 A08  32413.0 
    524.832         nan 
    495.050         nan 
      5.344         nan 
 
 A08  33956.0 
    523.966         nan 
    500.500         nan 
      5.348         nan 
 
 A08  35499.0 
    523.395         nan 
    503.000         nan 
      5.346         nan 
 
 A08  37042.0 
    520.582         nan 
    505.400         nan 
      5.348         nan 
 
 A08  38585.0 
    518.193         nan 
    507.000         nan 
      5.349         nan 
 
 A08  40128.0 
    515.406         nan 
    508.150         nan 
      5.350         nan 
 
 A08  41671.0 
    515.222         nan 
    509.900         nan 
      5.349         nan 
 
 A08  43214.0 
    515.186         nan 
    511.600         nan 
      5.349         nan 
 
 A08  44757.0 
    513.934         nan 
    512.350         nan 
      5.349         nan 
 
 A08  46300.0 
        nan         nan 
        nan         nan 
        nan         nan 
 
 A09  10.0 
     14.375         nan 
      7.350         nan 
      2.729         nan 
 
 A09  1553.0 
    178.007         nan 
    111.400         nan 
      4.803         nan 
 
 A09  3096.0 
    211.053         nan 
    145.250         nan 
      4.861         nan 
 
 A09  4639.0 
    234.992         nan 
    169.050         nan 
      4.875         nan 
 
 A09  6182.0 
    247.141         nan 
    188.700         nan 
      4.889         nan 
 
 A09  7725.0 
    247.287         nan 
    197.900         nan 
      4.881         nan 
 
 A09  9268.0 
    263.070         nan 
    211.500         nan 
      4.899         nan 
 
 A09  10811.0 
    270.399         nan 
    221.550         nan 
      4.911         nan 
 
 A09  12354.0 
    269.470         nan 
    226.850         nan 
      4.904         nan 
 
 A09  13897.0 
    269.818         nan 
    231.350         nan 
      4.901         nan 
 
 A09  15440.0 
    272.411         nan 
    238.400         nan 
      4.913         nan 
 
 A09  16983.0 
    274.316         nan 
    243.900         nan 
      4.906         nan 
 
 A09  18526.0 
    273.275         nan 
    247.000         nan 
      4.909         nan 
 
 A09  20069.0 
    277.406         nan 
    251.200         nan 
      4.912         nan 
 
 A09  21612.0 
    274.667         nan 
    253.300         nan 
      4.914         nan 
 
 A09  23155.0 
    273.951         nan 
    256.450         nan 
      4.917         nan 
 
 A09  24698.0 
    277.225         nan 
    259.650         nan 
      4.910         nan 
 
 A09  26241.0 
    275.256         nan 
    260.700         nan 
      4.909         nan 
 
 A09  27784.0 
    273.190         nan 
    263.200         nan 
      4.913         nan 
 
 A09  29327.0 
    273.793         nan 
    264.400         nan 
      4.916         nan 
 
 A09  30870.0 
    272.180         nan 
    265.350         nan 
      4.916         nan 
 
 A09  32413.0 
    272.900         nan 
    266.700         nan 
      4.915         nan 
 
 A09  33956.0 
    273.000         nan 
    268.000         nan 
      4.915         nan 
 
 A09  35499.0 
    272.846         nan 
    268.600         nan 
      4.915         nan 
 
 A09  37042.0 
    272.274         nan 
    269.400         nan 
      4.915         nan 
 
 A09  38585.0 
    271.689         nan 
    269.600         nan 
      4.916         nan 
 
 A09  40128.0 
    271.463         nan 
    270.450         nan 
      4.917         nan 
 
 A09  41671.0 
    271.322         nan 
    270.700         nan 
      4.917         nan 
 
 A09  43214.0 
    271.224         nan 
    270.900         nan 
      4.916         nan 
 
 A09  44757.0 
    271.056         nan 
    271.000         nan 
      4.917         nan 
 
 A09  46300.0 
    271.000         nan 
    271.000         nan 
      4.917         nan 
 
 A10  10.0 
     22.200         nan 
      8.150         nan 
      2.919         nan 
 
 A10  1553.0 
    242.391         nan 
    117.750         nan 
      4.649         nan 
 
 A10  3096.0 
    313.905         nan 
    165.750         nan 
      4.699         nan 
 
 A10  4639.0 
    353.874         nan 
    199.900         nan 
      4.736         nan 
 
 A10  6182.0 
    369.505         nan 
    223.300         nan 
      4.737         nan 
 
 A10  7725.0 
    411.911         nan 
    250.600         nan 
      4.747         nan 
 
 A10  9268.0 
    418.491         nan 
    271.650         nan 
      4.764         nan 
 
 A10  10811.0 
    433.524         nan 
    289.050         nan 
      4.757         nan 
 
 A10  12354.0 
    437.387         nan 
    305.350         nan 
      4.768         nan 
 
 A10  13897.0 
    453.960         nan 
    320.150         nan 
      4.772         nan 
 
 A10  15440.0 
    451.358         nan 
    333.800         nan 
      4.775         nan 
 
 A10  16983.0 
    464.049         nan 
    345.550         nan 
      4.772         nan 
 
 A10  18526.0 
    481.031         nan 
    357.200         nan 
      4.774         nan 
 
 A10  20069.0 
    468.901         nan 
    363.100         nan 
      4.772         nan 
 
 A10  21612.0 
    482.856         nan 
    374.700         nan 
      4.776         nan 
 
 A10  23155.0 
    472.893         nan 
    380.600         nan 
      4.779         nan 
 
 A10  24698.0 
    474.441         nan 
    389.650         nan 
      4.780         nan 
 
 A10  26241.0 
    486.727         nan 
    397.500         nan 
      4.782         nan 
 
 A10  27784.0 
    483.101         nan 
    404.050         nan 
      4.782         nan 
 
 A10  29327.0 
    485.436         nan 
    409.650         nan 
      4.781         nan 
 
 A10  30870.0 
    486.520         nan 
    415.550         nan 
      4.779         nan 
 
 A10  32413.0 
    483.505         nan 
    420.300         nan 
      4.784         nan 
 
 A10  33956.0 
    483.169         nan 
    423.950         nan 
      4.787         nan 
 
 A10  35499.0 
    488.214         nan 
    429.950         nan 
      4.785         nan 
 
 A10  37042.0 
    486.604         nan 
    433.300         nan 
      4.786         nan 
 
 A10  38585.0 
    486.783         nan 
    437.850         nan 
      4.786         nan 
 
 A10  40128.0 
    487.146         nan 
    440.300         nan 
      4.786         nan 
 
 A10  41671.0 
    487.725         nan 
    443.500         nan 
      4.786         nan 
 
 A10  43214.0 
    488.502         nan 
    447.500         nan 
      4.786         nan 
 
 A10  44757.0 
    491.004         nan 
    450.400         nan 
      4.787         nan 
 
 A10  46300.0 
        nan         nan 
        nan         nan 
        nan         nan 
 
 A11  10.0 
     26.075         nan 
      8.700         nan 
      3.048         nan 
 
 A11  1553.0 
    337.551         nan 
    187.850         nan 
      5.741         nan 
 
 A11  3096.0 
    424.173         nan 
    261.600         nan 
      5.825         nan 
 
 A11  4639.0 
    445.386         nan 
    308.750         nan 
      5.836         nan 
 
 A11  6182.0 
    483.799         nan 
    350.300         nan 
      5.866         nan 
 
 A11  7725.0 
    495.701         nan 
    380.800         nan 
      5.880         nan 
 
 A11  9268.0 
    506.781         nan 
    400.100         nan 
      5.884         nan 
 
 A11  10811.0 
    519.652         nan 
    417.950         nan 
      5.889         nan 
 
 A11  12354.0 
    522.918         nan 
    437.200         nan 
      5.897         nan 
 
 A11  13897.0 
    530.804         nan 
    448.250         nan 
      5.893         nan 
 
 A11  15440.0 
    542.258         nan 
    465.300         nan 
      5.905         nan 
 
 A11  16983.0 
    541.336         nan 
    472.600         nan 
      5.902         nan 
 
 A11  18526.0 
    543.081         nan 
    480.750         nan 
      5.902         nan 
 
 A11  20069.0 
    545.462         nan 
    485.950         nan 
      5.907         nan 
 
 A11  21612.0 
    545.599         nan 
    493.950         nan 
      5.913         nan 
 
 A11  23155.0 
    543.960         nan 
    498.700         nan 
      5.912         nan 
 
 A11  24698.0 
    552.222         nan 
    507.800         nan 
      5.916         nan 
 
 A11  26241.0 
    551.697         nan 
    510.150         nan 
      5.912         nan 
 
 A11  27784.0 
    555.845         nan 
    517.450         nan 
      5.914         nan 
 
 A11  29327.0 
    558.501         nan 
    521.000         nan 
      5.918         nan 
 
 A11  30870.0 
    552.065         nan 
    521.950         nan 
      5.917         nan 
 
 A11  32413.0 
    556.008         nan 
    525.800         nan 
      5.918         nan 
 
 A11  33956.0 
    554.933         nan 
    528.600         nan 
      5.920         nan 
 
 A11  35499.0 
    554.504         nan 
    530.350         nan 
      5.920         nan 
 
 A11  37042.0 
    557.903         nan 
    534.600         nan 
      5.922         nan 
 
 A11  38585.0 
    554.788         nan 
    534.800         nan 
      5.921         nan 
 
 A11  40128.0 
    559.701         nan 
    538.350         nan 
      5.922         nan 
 
 A11  41671.0 
    558.218         nan 
    539.500         nan 
      5.923         nan 
 
 A11  43214.0 
    559.722         nan 
    540.750         nan 
      5.921         nan 
 
 A11  44757.0 
    560.901         nan 
    542.500         nan 
      5.922         nan 
 
 A11  46300.0 
    562.278         nan 
    544.000         nan 
      5.922         nan 
 
 A12  10.0 
     27.400         nan 
      8.550         nan 
      3.003         nan 
 
 A12  1553.0 
    289.482         nan 
    150.600         nan 
      5.293         nan 
 
 A12  3096.0 
    385.600         nan 
    212.200         nan 
      5.350         nan 
 
 A12  4639.0 
    441.285         nan 
    261.300         nan 
      5.380         nan 
 
 A12  6182.0 
    480.039         nan 
    300.400         nan 
      5.414         nan 
 
 A12  7725.0 
    478.046         nan 
    328.850         nan 
      5.414         nan 
 
 A12  9268.0 
    493.466         nan 
    355.450         nan 
      5.421         nan 
 
 A12  10811.0 
    519.243         nan 
    379.350         nan 
      5.430         nan 
 
 A12  12354.0 
    519.861         nan 
    395.250         nan 
      5.430         nan 
 
 A12  13897.0 
    527.763         nan 
    412.450         nan 
      5.442         nan 
 
 A12  15440.0 
    536.400         nan 
    427.200         nan 
      5.436         nan 
 
 A12  16983.0 
    546.130         nan 
    441.100         nan 
      5.445         nan 
 
 A12  18526.0 
    541.036         nan 
    447.600         nan 
      5.441         nan 
 
 A12  20069.0 
    548.679         nan 
    459.950         nan 
      5.443         nan 
 
 A12  21612.0 
    556.770         nan 
    470.900         nan 
      5.444         nan 
 
 A12  23155.0 
    557.073         nan 
    479.000         nan 
      5.453         nan 
 
 A12  24698.0 
    553.125         nan 
    484.100         nan 
      5.448         nan 
 
 A12  26241.0 
    556.776         nan 
    491.250         nan 
      5.446         nan 
 
 A12  27784.0 
    560.951         nan 
    498.200         nan 
      5.448         nan 
 
 A12  29327.0 
    561.770         nan 
    505.800         nan 
      5.450         nan 
 
 A12  30870.0 
    559.708         nan 
    509.400         nan 
      5.454         nan 
 
 A12  32413.0 
    559.658         nan 
    513.700         nan 
      5.452         nan 
 
 A12  33956.0 
    565.976         nan 
    519.350         nan 
      5.451         nan 
 
 A12  35499.0 
    560.043         nan 
    522.500         nan 
      5.453         nan 
 
 A12  37042.0 
    561.724         nan 
    525.750         nan 
      5.455         nan 
 
 A12  38585.0 
    559.869         nan 
    529.250         nan 
      5.455         nan 
 
 A12  40128.0 
    560.906         nan 
    531.050         nan 
      5.455         nan 
 
 A12  41671.0 
    563.545         nan 
    534.650         nan 
      5.455         nan 
 
 A12  43214.0 
    561.163         nan 
    536.750         nan 
      5.455         nan 
 
 A12  44757.0 
    560.077         nan 
    538.700         nan 
      5.456         nan 
 
 A12  46300.0 
        nan         nan 
        nan         nan 
        nan         nan 
 
 A13  10.0 
     15.175         nan 
      7.450         nan 
      2.764         nan 
 
 A13  1553.0 
    119.208         nan 
     83.200         nan 
      4.842         nan 
 
 A13  3096.0 
    149.651         nan 
    100.950         nan 
      4.849         nan 
 
 A13  4639.0 
    177.379         nan 
    118.600         nan 
      4.881         nan 
 
 A13  6182.0 
    178.103         nan 
    126.650         nan 
      4.864         nan 
 
 A13  7725.0 
    193.800         nan 
    139.200         nan 
      4.875         nan 
 
 A13  9268.0 
    201.914         nan 
    147.650         nan 
      4.877         nan 
 
 A13  10811.0 
    204.416         nan 
    153.350         nan 
      4.881         nan 
 
 A13  12354.0 
    224.370         nan 
    159.100         nan 
      4.883         nan 
 
 A13  13897.0 
    222.277         nan 
    166.350         nan 
      4.883         nan 
 
 A13  15440.0 
    223.952         nan 
    173.150         nan 
      4.887         nan 
 
 A13  16983.0 
    219.613         nan 
    176.750         nan 
      4.883         nan 
 
 A13  18526.0 
    221.748         nan 
    181.350         nan 
      4.887         nan 
 
 A13  20069.0 
    227.671         nan 
    184.700         nan 
      4.888         nan 
 
 A13  21612.0 
    230.448         nan 
    189.050         nan 
      4.885         nan 
 
 A13  23155.0 
    234.783         nan 
    194.500         nan 
      4.889         nan 
 
 A13  24698.0 
    227.339         nan 
    195.400         nan 
      4.892         nan 
 
 A13  26241.0 
    226.276         nan 
    197.300         nan 
      4.887         nan 
 
 A13  27784.0 
    228.496         nan 
    199.200         nan 
      4.888         nan 
 
 A13  29327.0 
    232.204         nan 
    202.400         nan 
      4.887         nan 
 
 A13  30870.0 
    231.194         nan 
    204.350         nan 
      4.889         nan 
 
 A13  32413.0 
    230.697         nan 
    207.050         nan 
      4.889         nan 
 
 A13  33956.0 
    231.539         nan 
    208.450         nan 
      4.887         nan 
 
 A13  35499.0 
    229.884         nan 
    210.100         nan 
      4.891         nan 
 
 A13  37042.0 
    230.315         nan 
    212.300         nan 
      4.890         nan 
 
 A13  38585.0 
    231.225         nan 
    212.900         nan 
      4.890         nan 
 
 A13  40128.0 
    230.419         nan 
    214.600         nan 
      4.892         nan 
 
 A13  41671.0 
    232.358         nan 
    215.500         nan 
      4.889         nan 
 
 A13  43214.0 
    233.125         nan 
    217.450         nan 
      4.891         nan 
 
 A13  44757.0 
    230.639         nan 
    217.750         nan 
      4.890         nan 
 
 A13  46300.0 
        nan         nan 
        nan         nan 
        nan         nan 
 
 B01  10.0 
     25.650         nan 
      8.700         nan 
      3.054         nan 
 
 B01  1553.0 
    323.150         nan 
    197.050         nan 
      5.997         nan 
 
 B01  3096.0 
    376.215         nan 
    265.250         nan 
      6.078         nan 
 
 B01  4639.0 
    397.841         nan 
    309.100         nan 
      6.127         nan 
 
 B01  6182.0 
    404.897         nan 
    332.300         nan 
      6.137         nan 
 
 B01  7725.0 
    423.863         nan 
    355.700         nan 
      6.143         nan 
 
 B01  9268.0 
    432.382         nan 
    369.000         nan 
      6.148         nan 
 
 B01  10811.0 
    440.977         nan 
    384.600         nan 
      6.165         nan 
 
 B01  12354.0 
    436.616         nan 
    391.400         nan 
      6.170         nan 
 
 B01  13897.0 
    443.201         nan 
    400.900         nan 
      6.172         nan 
 
 B01  15440.0 
    443.941         nan 
    408.550         nan 
      6.170         nan 
 
 B01  16983.0 
    446.594         nan 
    415.300         nan 
      6.175         nan 
 
 B01  18526.0 
    447.682         nan 
    417.500         nan 
      6.175         nan 
 
 B01  20069.0 
    449.022         nan 
    421.600         nan 
      6.174         nan 
 
 B01  21612.0 
    446.604         nan 
    425.550         nan 
      6.182         nan 
 
 B01  23155.0 
    453.224         nan 
    430.150         nan 
      6.176         nan 
 
 B01  24698.0 
    452.140         nan 
    432.450         nan 
      6.179         nan 
 
 B01  26241.0 
    448.967         nan 
    434.150         nan 
      6.182         nan 
 
 B01  27784.0 
    449.349         nan 
    437.350         nan 
      6.183         nan 
 
 B01  29327.0 
    449.162         nan 
    437.550         nan 
      6.184         nan 
 
 B01  30870.0 
    446.542         nan 
    439.600         nan 
      6.182         nan 
 
 B01  32413.0 
    447.231         nan 
    441.150         nan 
      6.182         nan 
 
 B01  33956.0 
    448.170         nan 
    442.550         nan 
      6.184         nan 
 
 B01  35499.0 
    447.152         nan 
    443.400         nan 
      6.184         nan 
 
 B01  37042.0 
    446.676         nan 
    444.300         nan 
      6.184         nan 
 
 B01  38585.0 
    446.536         nan 
    444.900         nan 
      6.186         nan 
 
 B01  40128.0 
    446.865         nan 
    445.550         nan 
      6.185         nan 
 
 B01  41671.0 
    446.448         nan 
    445.600         nan 
      6.184         nan 
 
 B01  43214.0 
    446.293         nan 
    446.000         nan 
      6.186         nan 
 
 B01  44757.0 
    446.094         nan 
    446.000         nan 
      6.185         nan 
 
 B01  46300.0 
    446.000         nan 
    446.000         nan 
      6.186         nan 
 
 B02  10.0 
     19.760         nan 
      7.700         nan 
      2.799         nan 
 
 B02  1553.0 
    258.999         nan 
    169.750         nan 
      5.166         nan 
 
 B02  3096.0 
    290.576         nan 
    221.550         nan 
      5.224         nan 
 
 B02  4639.0 
    308.618         nan 
    254.350         nan 
      5.290         nan 
 
 B02  6182.0 
    314.501         nan 
    271.800         nan 
      5.278         nan 
 
 B02  7725.0 
    323.094         nan 
    285.800         nan 
      5.289         nan 
 
 B02  9268.0 
    329.045         nan 
    296.850         nan 
      5.290         nan 
 
 B02  10811.0 
    335.897         nan 
    305.950         nan 
      5.297         nan 
 
 B02  12354.0 
    336.000         nan 
    311.300         nan 
      5.305         nan 
 
 B02  13897.0 
    333.997         nan 
    314.400         nan 
      5.299         nan 
 
 B02  15440.0 
    337.622         nan 
    318.250         nan 
      5.297         nan 
 
 B02  16983.0 
    338.451         nan 
    322.900         nan 
      5.301         nan 
 
 B02  18526.0 
    337.802         nan 
    324.200         nan 
      5.311         nan 
 
 B02  20069.0 
    338.792         nan 
    327.550         nan 
      5.306         nan 
 
 B02  21612.0 
    343.051         nan 
    330.900         nan 
      5.306         nan 
 
 B02  23155.0 
    340.797         nan 
    330.850         nan 
      5.310         nan 
 
 B02  24698.0 
    340.784         nan 
    332.750         nan 
      5.310         nan 
 
 B02  26241.0 
    341.632         nan 
    334.050         nan 
      5.308         nan 
 
 B02  27784.0 
    340.574         nan 
    335.750         nan 
      5.311         nan 
 
 B02  29327.0 
    339.114         nan 
    335.200         nan 
      5.312         nan 
 
 B02  30870.0 
    339.532         nan 
    336.450         nan 
      5.312         nan 
 
 B02  32413.0 
    340.679         nan 
    337.400         nan 
      5.308         nan 
 
 B02  33956.0 
    338.699         nan 
    337.000         nan 
      5.311         nan 
 
 B02  35499.0 
    339.387         nan 
    338.100         nan 
      5.312         nan 
 
 B02  37042.0 
    339.660         nan 
    338.550         nan 
      5.314         nan 
 
 B02  38585.0 
    338.814         nan 
    338.250         nan 
      5.313         nan 
 
 B02  40128.0 
    339.152         nan 
    338.800         nan 
      5.315         nan 
 
 B02  41671.0 
    338.949         nan 
    338.800         nan 
      5.313         nan 
 
 B02  43214.0 
    339.081         nan 
    338.950         nan 
      5.314         nan 
 
 B02  44757.0 
    338.968         nan 
    338.950         nan 
      5.314         nan 
 
 B02  46300.0 
    339.000         nan 
    339.000         nan 
      5.313         nan 
 
 B03  10.0 
     26.400         nan 
      8.850         nan 
      3.084         nan 
 
 B03  1553.0 
    420.476         nan 
    287.150         nan 
      6.356         nan 
 
 B03  3096.0 
    483.050         nan 
    379.200         nan 
      6.494         nan 
 
 B03  4639.0 
    522.172         nan 
    428.550         nan 
      6.542         nan 
 
 B03  6182.0 
    535.578         nan 
    458.350         nan 
      6.556         nan 
 
 B03  7725.0 
    551.792         nan 
    482.150         nan 
      6.586         nan 
 
 B03  9268.0 
    558.203         nan 
    496.900         nan 
      6.579         nan 
 
 B03  10811.0 
    564.182         nan 
    513.800         nan 
      6.597         nan 
 
 B03  12354.0 
    578.665         nan 
    522.350         nan 
      6.592         nan 
 
 B03  13897.0 
    573.795         nan 
    529.500         nan 
      6.599         nan 
 
 B03  15440.0 
    587.887         nan 
    539.500         nan 
      6.609         nan 
 
 B03  16983.0 
    591.866         nan 
    545.800         nan 
      6.608         nan 
 
 B03  18526.0 
    596.707         nan 
    552.300         nan 
      6.609         nan 
 
 B03  20069.0 
    600.125         nan 
    556.100         nan 
      6.608         nan 
 
 B03  21612.0 
    607.703         nan 
    561.650         nan 
      6.615         nan 
 
 B03  23155.0 
    608.871         nan 
    565.650         nan 
      6.613         nan 
 
 B03  24698.0 
    615.347         nan 
    570.300         nan 
      6.615         nan 
 
 B03  26241.0 
    613.935         nan 
    573.350         nan 
      6.619         nan 
 
 B03  27784.0 
    623.669         nan 
    577.300         nan 
      6.620         nan 
 
 B03  29327.0 
    617.963         nan 
    577.700         nan 
      6.617         nan 
 
 B03  30870.0 
    626.295         nan 
    583.550         nan 
      6.621         nan 
 
 B03  32413.0 
    628.586         nan 
    585.800         nan 
      6.621         nan 
 
 B03  33956.0 
    624.772         nan 
    587.700         nan 
      6.622         nan 
 
 B03  35499.0 
    627.539         nan 
    589.200         nan 
      6.621         nan 
 
 B03  37042.0 
    628.241         nan 
    591.950         nan 
      6.623         nan 
 
 B03  38585.0 
    628.215         nan 
    593.950         nan 
      6.623         nan 
 
 B03  40128.0 
    628.147         nan 
    595.400         nan 
      6.621         nan 
 
 B03  41671.0 
    633.045         nan 
    598.250         nan 
      6.622         nan 
 
 B03  43214.0 
    632.983         nan 
    600.050         nan 
      6.624         nan 
 
 B03  44757.0 
    635.330         nan 
    601.750         nan 
      6.625         nan 
 
 B03  46300.0 
    634.794         nan 
    603.000         nan 
      6.625         nan 
 
 B04  10.0 
     21.325         nan 
      7.950         nan 
      2.866         nan 
 
 B04  1553.0 
    236.280         nan 
    148.700         nan 
      5.079         nan 
 
 B04  3096.0 
    306.666         nan 
    196.000         nan 
      5.134         nan 
 
 B04  4639.0 
    322.072         nan 
    226.950         nan 
      5.154         nan 
 
 B04  6182.0 
    343.894         nan 
    254.250         nan 
      5.166         nan 
 
 B04  7725.0 
    359.265         nan 
    272.150         nan 
      5.164         nan 
 
 B04  9268.0 
    363.720         nan 
    288.150         nan 
      5.186         nan 
 
 B04  10811.0 
    373.656         nan 
    301.650         nan 
      5.183         nan 
 
 B04  12354.0 
    374.147         nan 
    309.650         nan 
      5.182         nan 
 
 B04  13897.0 
    380.587         nan 
    321.100         nan 
      5.191         nan 
 
 B04  15440.0 
    390.064         nan 
    329.800         nan 
      5.193         nan 
 
 B04  16983.0 
    388.978         nan 
    336.550         nan 
      5.190         nan 
 
 B04  18526.0 
    388.941         nan 
    341.450         nan 
      5.196         nan 
 
 B04  20069.0 
    391.786         nan 
    345.200         nan 
      5.194         nan 
 
 B04  21612.0 
    392.218         nan 
    351.250         nan 
      5.197         nan 
 
 B04  23155.0 
    400.442         nan 
    357.750         nan 
      5.196         nan 
 
 B04  24698.0 
    398.091         nan 
    361.300         nan 
      5.199         nan 
 
 B04  26241.0 
    400.787         nan 
    364.100         nan 
      5.200         nan 
 
 B04  27784.0 
    405.560         nan 
    367.850         nan 
      5.199         nan 
 
 B04  29327.0 
    407.521         nan 
    371.900         nan 
      5.200         nan 
 
 B04  30870.0 
    404.192         nan 
    374.950         nan 
      5.200         nan 
 
 B04  32413.0 
    399.209         nan 
    375.450         nan 
      5.202         nan 
 
 B04  33956.0 
    402.404         nan 
    378.900         nan 
      5.202         nan 
 
 B04  35499.0 
    403.400         nan 
    380.750         nan 
      5.203         nan 
 
 B04  37042.0 
    403.689         nan 
    381.350         nan 
      5.202         nan 
 
 B04  38585.0 
    403.622         nan 
    384.000         nan 
      5.203         nan 
 
 B04  40128.0 
    403.239         nan 
    386.150         nan 
      5.206         nan 
 
 B04  41671.0 
    404.471         nan 
    388.000         nan 
      5.204         nan 
 
 B04  43214.0 
    402.986         nan 
    388.150         nan 
      5.202         nan 
 
 B04  44757.0 
    402.631         nan 
    389.950         nan 
      5.205         nan 
 
 B04  46300.0 
    402.601         nan 
    390.950         nan 
      5.205         nan 
 
 B05  10.0 
     26.950         nan 
      8.500         nan 
      2.989         nan 
 
 B05  1553.0 
    203.493         nan 
    136.500         nan 
      5.206         nan 
 
 B05  3096.0 
    251.866         nan 
    179.700         nan 
      5.262         nan 
 
 B05  4639.0 
    276.439         nan 
    209.850         nan 
      5.293         nan 
 
 B05  6182.0 
    294.487         nan 
    228.550         nan 
      5.300         nan 
 
 B05  7725.0 
    301.932         nan 
    242.800         nan 
      5.311         nan 
 
 B05  9268.0 
    299.781         nan 
    254.250         nan 
      5.307         nan 
 
 B05  10811.0 
    305.138         nan 
    263.400         nan 
      5.320         nan 
 
 B05  12354.0 
    311.323         nan 
    272.050         nan 
      5.320         nan 
 
 B05  13897.0 
    311.376         nan 
    278.400         nan 
      5.318         nan 
 
 B05  15440.0 
    320.308         nan 
    284.700         nan 
      5.318         nan 
 
 B05  16983.0 
    319.049         nan 
    288.650         nan 
      5.327         nan 
 
 B05  18526.0 
    324.613         nan 
    294.250         nan 
      5.325         nan 
 
 B05  20069.0 
    328.099         nan 
    298.550         nan 
      5.326         nan 
 
 B05  21612.0 
    323.844         nan 
    300.250         nan 
      5.326         nan 
 
 B05  23155.0 
    325.860         nan 
    302.000         nan 
      5.330         nan 
 
 B05  24698.0 
    328.656         nan 
    305.250         nan 
      5.334         nan 
 
 B05  26241.0 
    332.058         nan 
    307.650         nan 
      5.327         nan 
 
 B05  27784.0 
    328.185         nan 
    309.500         nan 
      5.328         nan 
 
 B05  29327.0 
    329.608         nan 
    311.050         nan 
      5.334         nan 
 
 B05  30870.0 
    330.188         nan 
    312.600         nan 
      5.335         nan 
 
 B05  32413.0 
    334.962         nan 
    314.550         nan 
      5.331         nan 
 
 B05  33956.0 
    332.838         nan 
    315.800         nan 
      5.334         nan 
 
 B05  35499.0 
    332.661         nan 
    316.850         nan 
      5.332         nan 
 
 B05  37042.0 
    333.321         nan 
    318.400         nan 
      5.333         nan 
 
 B05  38585.0 
    332.356         nan 
    318.700         nan 
      5.334         nan 
 
 B05  40128.0 
    333.807         nan 
    320.300         nan 
      5.333         nan 
 
 B05  41671.0 
    333.821         nan 
    321.000         nan 
      5.333         nan 
 
 B05  43214.0 
    334.785         nan 
    322.900         nan 
      5.334         nan 
 
 B05  44757.0 
    334.301         nan 
    323.350         nan 
      5.333         nan 
 
 B05  46300.0 
    334.166         nan 
    324.000         nan 
      5.334         nan 
 
 B06  10.0 
     11.542         nan 
      6.500         nan 
      2.484         nan 
 
 B06  1553.0 
    129.009         nan 
     83.700         nan 
      4.175         nan 
 
 B06  3096.0 
    159.724         nan 
    112.900         nan 
      4.235         nan 
 
 B06  4639.0 
    179.725         nan 
    131.400         nan 
      4.245         nan 
 
 B06  6182.0 
    179.607         nan 
    140.700         nan 
      4.244         nan 
 
 B06  7725.0 
    187.863         nan 
    152.700         nan 
      4.256         nan 
 
 B06  9268.0 
    195.133         nan 
    160.900         nan 
      4.257         nan 
 
 B06  10811.0 
    198.144         nan 
    167.150         nan 
      4.257         nan 
 
 B06  12354.0 
    193.595         nan 
    170.850         nan 
      4.265         nan 
 
 B06  13897.0 
    202.830         nan 
    175.000         nan 
      4.257         nan 
 
 B06  15440.0 
    203.849         nan 
    181.000         nan 
      4.267         nan 
 
 B06  16983.0 
    204.526         nan 
    183.800         nan 
      4.264         nan 
 
 B06  18526.0 
    202.017         nan 
    184.900         nan 
      4.268         nan 
 
 B06  20069.0 
    206.297         nan 
    188.950         nan 
      4.266         nan 
 
 B06  21612.0 
    204.542         nan 
    189.400         nan 
      4.266         nan 
 
 B06  23155.0 
    207.238         nan 
    192.050         nan 
      4.271         nan 
 
 B06  24698.0 
    206.909         nan 
    194.050         nan 
      4.272         nan 
 
 B06  26241.0 
    204.757         nan 
    194.300         nan 
      4.272         nan 
 
 B06  27784.0 
    205.419         nan 
    195.150         nan 
      4.266         nan 
 
 B06  29327.0 
    204.682         nan 
    197.150         nan 
      4.270         nan 
 
 B06  30870.0 
    206.278         nan 
    198.250         nan 
      4.269         nan 
 
 B06  32413.0 
    206.696         nan 
    199.950         nan 
      4.272         nan 
 
 B06  33956.0 
    205.786         nan 
    200.150         nan 
      4.272         nan 
 
 B06  35499.0 
    206.176         nan 
    200.350         nan 
      4.271         nan 
 
 B06  37042.0 
    206.421         nan 
    201.850         nan 
      4.273         nan 
 
 B06  38585.0 
    206.118         nan 
    201.900         nan 
      4.272         nan 
 
 B06  40128.0 
    206.675         nan 
    202.450         nan 
      4.271         nan 
 
 B06  41671.0 
    206.204         nan 
    202.800         nan 
      4.273         nan 
 
 B06  43214.0 
    206.646         nan 
    203.400         nan 
      4.273         nan 
 
 B06  44757.0 
    206.494         nan 
    203.450         nan 
      4.272         nan 
 
 B06  46300.0 
    206.393         nan 
    204.000         nan 
      4.272         nan 
 
 B07  10.0 
     19.585         nan 
      7.500         nan 
      2.754         nan 
 
 B07  1553.0 
    365.426         nan 
    194.350         nan 
      5.196         nan 
 
 B07  3096.0 
    478.774         nan 
    276.200         nan 
      5.288         nan 
 
 B07  4639.0 
    526.519         nan 
    333.250         nan 
      5.317         nan 
 
 B07  6182.0 
    554.288         nan 
    372.950         nan 
      5.330         nan 
 
 B07  7725.0 
    588.041         nan 
    409.950         nan 
      5.362         nan 
 
 B07  9268.0 
    595.200         nan 
    444.300         nan 
      5.371         nan 
 
 B07  10811.0 
    603.369         nan 
    461.900         nan 
      5.367         nan 
 
 B07  12354.0 
    624.162         nan 
    483.000         nan 
      5.375         nan 
 
 B07  13897.0 
    639.121         nan 
    505.300         nan 
      5.372         nan 
 
 B07  15440.0 
    642.792         nan 
    518.000         nan 
      5.383         nan 
 
 B07  16983.0 
    647.245         nan 
    533.600         nan 
      5.390         nan 
 
 B07  18526.0 
    657.001         nan 
    548.200         nan 
      5.391         nan 
 
 B07  20069.0 
    658.580         nan 
    557.400         nan 
      5.390         nan 
 
 B07  21612.0 
    663.647         nan 
    568.800         nan 
      5.391         nan 
 
 B07  23155.0 
    661.659         nan 
    576.000         nan 
      5.397         nan 
 
 B07  24698.0 
    670.033         nan 
    586.550         nan 
      5.402         nan 
 
 B07  26241.0 
    664.923         nan 
    592.850         nan 
      5.396         nan 
 
 B07  27784.0 
    666.547         nan 
    598.850         nan 
      5.401         nan 
 
 B07  29327.0 
    672.648         nan 
    608.250         nan 
      5.401         nan 
 
 B07  30870.0 
    671.942         nan 
    612.900         nan 
      5.405         nan 
 
 B07  32413.0 
    678.599         nan 
    617.400         nan 
      5.403         nan 
 
 B07  33956.0 
    678.934         nan 
    622.800         nan 
      5.405         nan 
 
 B07  35499.0 
    674.104         nan 
    626.350         nan 
      5.404         nan 
 
 B07  37042.0 
    677.612         nan 
    632.000         nan 
      5.406         nan 
 
 B07  38585.0 
    675.150         nan 
    634.800         nan 
      5.404         nan 
 
 B07  40128.0 
    678.134         nan 
    637.850         nan 
      5.407         nan 
 
 B07  41671.0 
    675.284         nan 
    640.700         nan 
      5.407         nan 
 
 B07  43214.0 
    677.970         nan 
    644.600         nan 
      5.407         nan 
 
 B07  44757.0 
    674.857         nan 
    646.600         nan 
      5.406         nan 
 
 B07  46300.0 
        nan         nan 
        nan         nan 
        nan         nan 
 
 B08  10.0 
     31.175         nan 
      9.050         nan 
      3.128         nan 
 
 B08  1553.0 
    710.944         nan 
    401.450         nan 
      7.061         nan 
 
 B08  3096.0 
    815.254         nan 
    561.900         nan 
      7.230         nan 
 
 B08  4639.0 
    848.638         nan 
    660.450         nan 
      7.290         nan 
 
 B08  6182.0 
    875.559         nan 
    727.350         nan 
      7.322         nan 
 
 B08  7725.0 
    900.633         nan 
    772.850         nan 
      7.347         nan 
 
 B08  9268.0 
    907.823         nan 
    807.200         nan 
      7.354         nan 
 
 B08  10811.0 
    914.207         nan 
    829.200         nan 
      7.364         nan 
 
 B08  12354.0 
    920.602         nan 
    851.150         nan 
      7.390         nan 
 
 B08  13897.0 
    928.232         nan 
    866.250         nan 
      7.381         nan 
 
 B08  15440.0 
    926.439         nan 
    877.300         nan 
      7.392         nan 
 
 B08  16983.0 
    928.087         nan 
    883.000         nan 
      7.397         nan 
 
 B08  18526.0 
    937.147         nan 
    894.000         nan 
      7.404         nan 
 
 B08  20069.0 
    938.783         nan 
    900.200         nan 
      7.401         nan 
 
 B08  21612.0 
    942.375         nan 
    906.400         nan 
      7.411         nan 
 
 B08  23155.0 
    944.768         nan 
    911.700         nan 
      7.409         nan 
 
 B08  24698.0 
    948.343         nan 
    915.850         nan 
      7.412         nan 
 
 B08  26241.0 
    950.034         nan 
    921.350         nan 
      7.413         nan 
 
 B08  27784.0 
    946.051         nan 
    921.500         nan 
      7.415         nan 
 
 B08  29327.0 
    955.286         nan 
    927.300         nan 
      7.418         nan 
 
 B08  30870.0 
    953.584         nan 
    928.750         nan 
      7.417         nan 
 
 B08  32413.0 
    950.501         nan 
    930.150         nan 
      7.420         nan 
 
 B08  33956.0 
    958.533         nan 
    933.100         nan 
      7.420         nan 
 
 B08  35499.0 
    955.663         nan 
    934.550         nan 
      7.419         nan 
 
 B08  37042.0 
    959.841         nan 
    937.450         nan 
      7.421         nan 
 
 B08  38585.0 
    959.969         nan 
    938.600         nan 
      7.421         nan 
 
 B08  40128.0 
    963.149         nan 
    940.350         nan 
      7.424         nan 
 
 B08  41671.0 
    964.576         nan 
    942.300         nan 
      7.423         nan 
 
 B08  43214.0 
    966.445         nan 
    942.800         nan 
      7.425         nan 
 
 B08  44757.0 
    968.959         nan 
    944.600         nan 
      7.424         nan 
 
 B08  46300.0 
    970.667         nan 
    946.000         nan 
      7.425         nan 
 
 B09  10.0 
     25.450         nan 
      8.550         nan 
      3.007         nan 
 
 B09  1553.0 
    309.520         nan 
    174.550         nan 
      5.720         nan 
 
 B09  3096.0 
    414.052         nan 
    245.600         nan 
      5.801         nan 
 
 B09  4639.0 
    448.920         nan 
    291.150         nan 
      5.822         nan 
 
 B09  6182.0 
    526.753         nan 
    331.450         nan 
      5.846         nan 
 
 B09  7725.0 
    526.586         nan 
    356.200         nan 
      5.855         nan 
 
 B09  9268.0 
    561.287         nan 
    388.800         nan 
      5.873         nan 
 
 B09  10811.0 
    573.968         nan 
    405.150         nan 
      5.880         nan 
 
 B09  12354.0 
    591.287         nan 
    427.850         nan 
      5.875         nan 
 
 B09  13897.0 
    589.996         nan 
    444.000         nan 
      5.886         nan 
 
 B09  15440.0 
    606.293         nan 
    459.450         nan 
      5.889         nan 
 
 B09  16983.0 
    619.998         nan 
    475.150         nan 
      5.888         nan 
 
 B09  18526.0 
    625.802         nan 
    489.200         nan 
      5.892         nan 
 
 B09  20069.0 
    634.954         nan 
    502.550         nan 
      5.894         nan 
 
 B09  21612.0 
    634.708         nan 
    510.400         nan 
      5.897         nan 
 
 B09  23155.0 
    638.900         nan 
    522.800         nan 
      5.899         nan 
 
 B09  24698.0 
    655.083         nan 
    535.550         nan 
      5.902         nan 
 
 B09  26241.0 
    647.386         nan 
    543.200         nan 
      5.900         nan 
 
 B09  27784.0 
    642.254         nan 
    549.350         nan 
      5.904         nan 
 
 B09  29327.0 
    652.513         nan 
    559.650         nan 
      5.901         nan 
 
 B09  30870.0 
    648.872         nan 
    564.100         nan 
      5.903         nan 
 
 B09  32413.0 
    645.293         nan 
    568.850         nan 
      5.905         nan 
 
 B09  33956.0 
    651.364         nan 
    578.150         nan 
      5.907         nan 
 
 B09  35499.0 
    651.752         nan 
    582.300         nan 
      5.907         nan 
 
 B09  37042.0 
    644.143         nan 
    586.450         nan 
      5.909         nan 
 
 B09  38585.0 
    645.663         nan 
    590.350         nan 
      5.907         nan 
 
 B09  40128.0 
    644.170         nan 
    595.200         nan 
      5.908         nan 
 
 B09  41671.0 
    642.726         nan 
    599.950         nan 
      5.910         nan 
 
 B09  43214.0 
    642.215         nan 
    603.800         nan 
      5.910         nan 
 
 B09  44757.0 
    639.622         nan 
    606.350         nan 
      5.910         nan 
 
 B09  46300.0 
        nan         nan 
        nan         nan 
        nan         nan 
 
 B10  10.0 
     11.717         nan 
      6.050         nan 
      2.355         nan 
 
 B10  1553.0 
    162.420         nan 
     91.600         nan 
      3.786         nan 
 
 B10  3096.0 
    199.462         nan 
    122.350         nan 
      3.802         nan 
 
 B10  4639.0 
    223.711         nan 
    147.150         nan 
      3.813         nan 
 
 B10  6182.0 
    234.353         nan 
    161.100         nan 
      3.822         nan 
 
 B10  7725.0 
    246.529         nan 
    174.800         nan 
      3.814         nan 
 
 B10  9268.0 
    257.838         nan 
    188.800         nan 
      3.831         nan 
 
 B10  10811.0 
    275.462         nan 
    198.350         nan 
      3.839         nan 
 
 B10  12354.0 
    280.012         nan 
    206.450         nan 
      3.838         nan 
 
 B10  13897.0 
    282.757         nan 
    216.850         nan 
      3.846         nan 
 
 B10  15440.0 
    300.006         nan 
    224.700         nan 
      3.843         nan 
 
 B10  16983.0 
    292.657         nan 
    229.200         nan 
      3.845         nan 
 
 B10  18526.0 
    290.710         nan 
    233.250         nan 
      3.846         nan 
 
 B10  20069.0 
    299.672         nan 
    241.500         nan 
      3.844         nan 
 
 B10  21612.0 
    302.795         nan 
    246.150         nan 
      3.853         nan 
 
 B10  23155.0 
    299.232         nan 
    249.400         nan 
      3.849         nan 
 
 B10  24698.0 
    306.505         nan 
    253.950         nan 
      3.854         nan 
 
 B10  26241.0 
    306.703         nan 
    258.600         nan 
      3.852         nan 
 
 B10  27784.0 
    307.653         nan 
    260.700         nan 
      3.852         nan 
 
 B10  29327.0 
    309.012         nan 
    266.000         nan 
      3.853         nan 
 
 B10  30870.0 
    301.291         nan 
    267.450         nan 
      3.852         nan 
 
 B10  32413.0 
    305.888         nan 
    270.650         nan 
      3.849         nan 
 
 B10  33956.0 
    304.022         nan 
    273.550         nan 
      3.856         nan 
 
 B10  35499.0 
    305.442         nan 
    274.950         nan 
      3.848         nan 
 
 B10  37042.0 
    306.511         nan 
    278.400         nan 
      3.852         nan 
 
 B10  38585.0 
    304.608         nan 
    279.400         nan 
      3.852         nan 
 
 B10  40128.0 
    305.814         nan 
    282.450         nan 
      3.854         nan 
 
 B10  41671.0 
    304.761         nan 
    283.600         nan 
      3.853         nan 
 
 B10  43214.0 
    303.013         nan 
    285.050         nan 
      3.854         nan 
 
 B10  44757.0 
    303.521         nan 
    286.250         nan 
      3.855         nan 
 
 B10  46300.0 
    303.945         nan 
    287.950         nan 
      3.854         nan 
 
 
 
 
 
 
