# Supplementary material for: The effects of genital myiasis on the diversity of the vaginal microbiota in female Bactrian camels
Source: BMC Vet Res. 2022 Mar 5;18:87. doi: 10.1186/s12917-022-03189-5 (PMC8897907; doi:10.1186/s12917-022-03189-5)
Supplement: Supplementary file 5 — Additional file 5. [file 12917_2022_3189_MOESM5_ESM.zip › MPL201709200_16s_yy/Treat1/B07_taxa_summary/taxa_summary_plots/bar_charts.html]

Taxa Summaries


|  |  |
| --- | --- |
|  | |
| Taxonomy Summary. Current Level: Phylum | |
| View Figure (.pdf)  View Legend (.pdf) |  |
|  |


|  |
| --- |
| View Table (.txt) |

|  |  |  |  |  |  |  |  |  |  |  |  |  |  |  |  |  |  |  |  |  |  |  |  |  |  |  |
| --- | --- | --- | --- | --- | --- | --- | --- | --- | --- | --- | --- | --- | --- | --- | --- | --- | --- | --- | --- | --- | --- | --- | --- | --- | --- | --- |
|  | | Total | | A01 | A02 | A03 | A04 | A05 | A06 | A07 | A08 | A09 | A10 | A11 | A12 | A13 | B01 | B02 | B03 | B04 | B05 | B06 | B07 | B08 | B09 | B10 |
| Legend | Taxonomy | count | % | % | % | % | % | % | % | % | % | % | % | % | % | % | % | % | % | % | % | % | % | % | % | % |
|  | k\_\_Bacteria;p\_\_Firmicutes | 8 | 34.6% | 33.8% | 53.3% | 32.0% | 30.3% | 83.5% | 27.1% | 52.4% | 41.0% | 24.5% | 24.2% | 32.7% | 41.3% | 35.2% | 39.7% | 9.2% | 20.9% | 10.9% | 19.5% | 71.0% | 23.4% | 32.0% | 45.6% | 11.2% |
|  | k\_\_Bacteria;p\_\_Proteobacteria | 7 | 31.4% | 14.9% | 7.8% | 47.1% | 43.9% | 7.3% | 26.8% | 19.7% | 25.9% | 57.1% | 33.3% | 38.5% | 25.9% | 22.0% | 36.2% | 58.0% | 54.2% | 38.2% | 29.9% | 11.1% | 23.1% | 37.6% | 23.7% | 40.3% |
|  | k\_\_Bacteria;p\_\_Fusobacteria | 4 | 16.4% | 40.0% | 20.4% | 6.0% | 5.5% | 0.0% | 28.5% | 7.4% | 17.8% | 7.6% | 28.5% | 12.7% | 18.4% | 16.7% | 7.0% | 12.7% | 4.3% | 32.4% | 25.5% | 0.1% | 31.6% | 9.9% | 7.5% | 37.6% |
|  | k\_\_Bacteria;p\_\_Bacteroidetes | 2 | 7.3% | 9.2% | 1.8% | 6.9% | 6.0% | 2.0% | 10.6% | 7.6% | 3.0% | 2.2% | 7.5% | 7.5% | 2.2% | 12.0% | 10.6% | 10.2% | 8.0% | 8.3% | 14.7% | 13.0% | 7.5% | 9.9% | 4.8% | 2.5% |
|  | k\_\_Bacteria;p\_\_Actinobacteria | 2 | 6.9% | 1.9% | 8.0% | 4.3% | 11.4% | 6.6% | 4.5% | 12.3% | 10.7% | 7.6% | 5.6% | 7.2% | 10.5% | 6.9% | 2.7% | 5.0% | 6.4% | 4.4% | 6.7% | 4.1% | 3.8% | 3.4% | 17.2% | 8.0% |
|  | No blast hit;Other | 0 | 1.6% | 0.1% | 8.5% | 0.3% | 0.5% | 0.5% | 0.6% | 0.0% | 0.9% | 0.0% | 0.0% | 0.0% | 0.6% | 6.9% | 1.0% | 1.4% | 0.6% | 4.2% | 0.8% | 0.0% | 8.9% | 0.1% | 0.2% | 0.0% |
|  | k\_\_Bacteria;p\_\_Cyanobacteria | 0 | 0.6% | 0.1% | 0.0% | 1.3% | 1.2% | 0.1% | 0.6% | 0.3% | 0.2% | 0.6% | 0.1% | 0.5% | 0.4% | 0.1% | 0.8% | 1.5% | 2.0% | 0.9% | 0.6% | 0.3% | 0.2% | 0.8% | 0.2% | 0.1% |
|  | k\_\_Bacteria;p\_\_Tenericutes | 0 | 0.2% | 0.0% | 0.2% | 0.2% | 0.0% | 0.0% | 0.7% | 0.0% | 0.1% | 0.0% | 0.5% | 0.1% | 0.1% | 0.0% | 0.0% | 0.0% | 0.1% | 0.0% | 1.6% | 0.0% | 0.1% | 0.2% | 0.0% | 0.2% |
|  | k\_\_Bacteria;p\_\_Verrucomicrobia | 0 | 0.2% | 0.0% | 0.0% | 0.1% | 0.0% | 0.0% | 0.0% | 0.0% | 0.2% | 0.0% | 0.1% | 0.1% | 0.2% | 0.0% | 0.0% | 0.0% | 0.8% | 0.0% | 0.0% | 0.0% | 0.4% | 1.5% | 0.1% | 0.0% |
|  | k\_\_Bacteria;p\_\_Acidobacteria | 0 | 0.1% | 0.0% | 0.0% | 0.3% | 0.2% | 0.0% | 0.0% | 0.1% | 0.0% | 0.1% | 0.0% | 0.0% | 0.0% | 0.0% | 0.1% | 0.3% | 0.3% | 0.0% | 0.1% | 0.0% | 0.0% | 1.3% | 0.2% | 0.0% |
|  | k\_\_Bacteria;p\_\_Gemmatimonadetes | 0 | 0.1% | 0.0% | 0.0% | 0.0% | 0.0% | 0.0% | 0.0% | 0.0% | 0.0% | 0.0% | 0.0% | 0.1% | 0.0% | 0.0% | 0.0% | 0.1% | 0.1% | 0.0% | 0.0% | 0.0% | 0.0% | 1.6% | 0.2% | 0.0% |
|  | k\_\_Bacteria;p\_\_Chloroflexi | 0 | 0.1% | 0.0% | 0.0% | 0.4% | 0.1% | 0.0% | 0.0% | 0.0% | 0.0% | 0.0% | 0.0% | 0.0% | 0.0% | 0.0% | 0.2% | 0.4% | 0.4% | 0.1% | 0.0% | 0.1% | 0.0% | 0.4% | 0.0% | 0.1% |
|  | k\_\_Bacteria;p\_\_GN02 | 0 | 0.1% | 0.0% | 0.0% | 0.1% | 0.4% | 0.0% | 0.1% | 0.0% | 0.0% | 0.0% | 0.0% | 0.1% | 0.0% | 0.0% | 0.2% | 0.3% | 0.4% | 0.1% | 0.1% | 0.1% | 0.0% | 0.1% | 0.1% | 0.0% |
|  | k\_\_Bacteria;p\_\_Lentisphaerae | 0 | 0.1% | 0.0% | 0.0% | 0.0% | 0.0% | 0.0% | 0.0% | 0.0% | 0.1% | 0.0% | 0.0% | 0.0% | 0.1% | 0.0% | 0.0% | 0.0% | 0.5% | 0.0% | 0.0% | 0.0% | 0.3% | 0.5% | 0.0% | 0.0% |
|  | k\_\_Bacteria;p\_\_Planctomycetes | 0 | 0.1% | 0.0% | 0.0% | 0.1% | 0.2% | 0.0% | 0.0% | 0.0% | 0.0% | 0.0% | 0.0% | 0.1% | 0.1% | 0.0% | 0.1% | 0.2% | 0.2% | 0.2% | 0.1% | 0.0% | 0.0% | 0.2% | 0.1% | 0.0% |
|  | k\_\_Bacteria;p\_\_Spirochaetes | 0 | 0.1% | 0.0% | 0.0% | 0.1% | 0.1% | 0.0% | 0.3% | 0.0% | 0.0% | 0.1% | 0.1% | 0.0% | 0.1% | 0.1% | 0.1% | 0.0% | 0.1% | 0.0% | 0.1% | 0.0% | 0.0% | 0.2% | 0.0% | 0.1% |
|  | k\_\_Bacteria;p\_\_SR1 | 0 | 0.1% | 0.0% | 0.0% | 0.0% | 0.0% | 0.0% | 0.0% | 0.0% | 0.0% | 0.0% | 0.0% | 0.0% | 0.0% | 0.0% | 0.7% | 0.0% | 0.0% | 0.0% | 0.1% | 0.0% | 0.5% | 0.0% | 0.0% | 0.0% |
|  | k\_\_Bacteria;p\_\_[Thermi] | 0 | 0.1% | 0.0% | 0.0% | 0.2% | 0.1% | 0.0% | 0.1% | 0.0% | 0.0% | 0.1% | 0.0% | 0.0% | 0.0% | 0.0% | 0.1% | 0.2% | 0.2% | 0.1% | 0.0% | 0.0% | 0.0% | 0.1% | 0.0% | 0.0% |
|  | k\_\_Bacteria;p\_\_TM7 | 0 | 0.0% | 0.0% | 0.0% | 0.1% | 0.1% | 0.0% | 0.0% | 0.0% | 0.0% | 0.0% | 0.0% | 0.0% | 0.1% | 0.0% | 0.2% | 0.0% | 0.1% | 0.0% | 0.0% | 0.2% | 0.0% | 0.0% | 0.0% | 0.0% |
|  | k\_\_Bacteria;p\_\_Nitrospirae | 0 | 0.0% | 0.0% | 0.0% | 0.1% | 0.0% | 0.0% | 0.0% | 0.0% | 0.0% | 0.0% | 0.0% | 0.0% | 0.0% | 0.0% | 0.1% | 0.1% | 0.1% | 0.0% | 0.0% | 0.0% | 0.0% | 0.1% | 0.0% | 0.0% |
|  | k\_\_Bacteria;p\_\_Armatimonadetes | 0 | 0.0% | 0.0% | 0.0% | 0.1% | 0.0% | 0.0% | 0.0% | 0.0% | 0.0% | 0.0% | 0.0% | 0.0% | 0.0% | 0.0% | 0.0% | 0.0% | 0.0% | 0.0% | 0.0% | 0.0% | 0.0% | 0.0% | 0.0% | 0.0% |
|  | k\_\_Bacteria;p\_\_Chlamydiae | 0 | 0.0% | 0.0% | 0.0% | 0.0% | 0.0% | 0.0% | 0.0% | 0.0% | 0.0% | 0.0% | 0.0% | 0.0% | 0.0% | 0.0% | 0.0% | 0.1% | 0.0% | 0.0% | 0.0% | 0.0% | 0.0% | 0.0% | 0.0% | 0.0% |
|  | k\_\_Bacteria;p\_\_WS3 | 0 | 0.0% | 0.0% | 0.0% | 0.1% | 0.0% | 0.0% | 0.0% | 0.0% | 0.0% | 0.0% | 0.0% | 0.0% | 0.0% | 0.0% | 0.0% | 0.0% | 0.1% | 0.0% | 0.0% | 0.0% | 0.0% | 0.0% | 0.0% | 0.0% |
|  | k\_\_Bacteria;p\_\_OD1 | 0 | 0.0% | 0.0% | 0.0% | 0.0% | 0.0% | 0.0% | 0.0% | 0.0% | 0.0% | 0.0% | 0.0% | 0.0% | 0.0% | 0.0% | 0.0% | 0.0% | 0.0% | 0.0% | 0.0% | 0.0% | 0.0% | 0.0% | 0.0% | 0.0% |
|  | k\_\_Bacteria;p\_\_WPS-2 | 0 | 0.0% | 0.0% | 0.0% | 0.0% | 0.0% | 0.0% | 0.0% | 0.0% | 0.0% | 0.0% | 0.0% | 0.0% | 0.0% | 0.0% | 0.0% | 0.0% | 0.0% | 0.0% | 0.0% | 0.0% | 0.0% | 0.0% | 0.0% | 0.0% |
|  | k\_\_Bacteria;p\_\_AD3 | 0 | 0.0% | 0.0% | 0.0% | 0.0% | 0.0% | 0.0% | 0.0% | 0.0% | 0.0% | 0.0% | 0.0% | 0.0% | 0.0% | 0.0% | 0.0% | 0.0% | 0.1% | 0.0% | 0.0% | 0.0% | 0.0% | 0.0% | 0.0% | 0.0% |
|  | k\_\_Bacteria;p\_\_Deferribacteres | 0 | 0.0% | 0.0% | 0.0% | 0.0% | 0.0% | 0.0% | 0.0% | 0.0% | 0.0% | 0.0% | 0.0% | 0.0% | 0.0% | 0.0% | 0.0% | 0.0% | 0.0% | 0.0% | 0.0% | 0.0% | 0.0% | 0.0% | 0.0% | 0.0% |
|  | k\_\_Bacteria;p\_\_GAL15 | 0 | 0.0% | 0.0% | 0.0% | 0.0% | 0.0% | 0.0% | 0.0% | 0.0% | 0.0% | 0.0% | 0.0% | 0.0% | 0.0% | 0.0% | 0.0% | 0.0% | 0.1% | 0.0% | 0.0% | 0.0% | 0.0% | 0.0% | 0.0% | 0.0% |
|  | k\_\_Bacteria;p\_\_Chlorobi | 0 | 0.0% | 0.0% | 0.0% | 0.0% | 0.0% | 0.0% | 0.0% | 0.0% | 0.0% | 0.0% | 0.0% | 0.0% | 0.0% | 0.0% | 0.0% | 0.0% | 0.0% | 0.0% | 0.0% | 0.0% | 0.0% | 0.0% | 0.0% | 0.0% |
|  | k\_\_Bacteria;p\_\_Elusimicrobia | 0 | 0.0% | 0.0% | 0.0% | 0.0% | 0.0% | 0.0% | 0.0% | 0.0% | 0.0% | 0.0% | 0.0% | 0.0% | 0.0% | 0.0% | 0.0% | 0.0% | 0.0% | 0.0% | 0.0% | 0.0% | 0.0% | 0.0% | 0.0% | 0.0% |
|  | k\_\_Bacteria;p\_\_Fibrobacteres | 0 | 0.0% | 0.0% | 0.0% | 0.0% | 0.0% | 0.0% | 0.0% | 0.0% | 0.0% | 0.0% | 0.0% | 0.0% | 0.0% | 0.0% | 0.0% | 0.0% | 0.0% | 0.0% | 0.0% | 0.0% | 0.0% | 0.0% | 0.0% | 0.0% |

|  |  |
| --- | --- |
|  | |
| Taxonomy Summary. Current Level: Class | |
| View Figure (.pdf)  View Legend (.pdf) |  |
|  |


|  |
| --- |
| View Table (.txt) |

|  |  |  |  |  |  |  |  |  |  |  |  |  |  |  |  |  |  |  |  |  |  |  |  |  |  |  |
| --- | --- | --- | --- | --- | --- | --- | --- | --- | --- | --- | --- | --- | --- | --- | --- | --- | --- | --- | --- | --- | --- | --- | --- | --- | --- | --- |
|  | | Total | | A01 | A02 | A03 | A04 | A05 | A06 | A07 | A08 | A09 | A10 | A11 | A12 | A13 | B01 | B02 | B03 | B04 | B05 | B06 | B07 | B08 | B09 | B10 |
| Legend | Taxonomy | count | % | % | % | % | % | % | % | % | % | % | % | % | % | % | % | % | % | % | % | % | % | % | % | % |
|  | k\_\_Bacteria;p\_\_Firmicutes;c\_\_Clostridia | 4 | 19.1% | 17.1% | 33.7% | 13.6% | 8.6% | 35.7% | 10.0% | 34.6% | 14.9% | 5.4% | 13.9% | 13.1% | 9.0% | 18.0% | 27.1% | 3.6% | 16.9% | 6.6% | 14.3% | 55.1% | 19.3% | 24.5% | 36.6% | 6.8% |
|  | k\_\_Bacteria;p\_\_Fusobacteria;c\_\_Fusobacteriia | 4 | 16.4% | 40.0% | 20.4% | 6.0% | 5.5% | 0.0% | 28.5% | 7.4% | 17.8% | 7.6% | 28.5% | 12.7% | 18.4% | 16.7% | 7.0% | 12.7% | 4.3% | 32.4% | 25.5% | 0.1% | 31.6% | 9.9% | 7.5% | 37.6% |
|  | k\_\_Bacteria;p\_\_Firmicutes;c\_\_Bacilli | 4 | 15.4% | 16.7% | 19.6% | 18.3% | 21.6% | 47.7% | 17.0% | 17.8% | 26.0% | 19.0% | 10.3% | 19.6% | 32.3% | 17.2% | 12.3% | 5.6% | 3.2% | 4.3% | 5.1% | 15.8% | 4.0% | 7.2% | 8.8% | 4.3% |
|  | k\_\_Bacteria;p\_\_Proteobacteria;c\_\_Alphaproteobacteria | 2 | 10.0% | 3.9% | 0.8% | 24.5% | 25.6% | 3.4% | 8.3% | 4.3% | 4.6% | 14.8% | 1.6% | 12.6% | 7.2% | 1.7% | 15.1% | 28.5% | 22.8% | 16.5% | 8.6% | 5.2% | 1.8% | 9.1% | 6.6% | 1.8% |
|  | k\_\_Bacteria;p\_\_Proteobacteria;c\_\_Epsilonproteobacteria | 2 | 9.4% | 6.8% | 6.6% | 1.6% | 3.8% | 0.0% | 10.7% | 10.9% | 15.1% | 17.9% | 26.8% | 10.7% | 7.5% | 17.2% | 2.6% | 4.4% | 3.2% | 6.3% | 9.0% | 0.1% | 16.1% | 3.2% | 0.5% | 34.1% |
|  | k\_\_Bacteria;p\_\_Actinobacteria;c\_\_Actinobacteria | 2 | 6.6% | 1.9% | 8.0% | 4.0% | 11.3% | 6.6% | 4.5% | 12.3% | 10.7% | 7.5% | 5.6% | 7.0% | 10.4% | 6.9% | 2.6% | 5.0% | 5.8% | 4.3% | 6.6% | 4.0% | 3.7% | 3.1% | 13.0% | 8.0% |
|  | k\_\_Bacteria;p\_\_Proteobacteria;c\_\_Gammaproteobacteria | 1 | 6.4% | 2.5% | 0.1% | 9.9% | 6.3% | 3.1% | 3.2% | 2.2% | 3.7% | 13.0% | 4.0% | 9.1% | 7.4% | 1.8% | 9.8% | 11.7% | 12.7% | 8.6% | 3.8% | 2.3% | 3.9% | 14.7% | 12.1% | 2.3% |
|  | k\_\_Bacteria;p\_\_Proteobacteria;c\_\_Betaproteobacteria | 1 | 5.3% | 1.7% | 0.3% | 10.8% | 7.9% | 0.8% | 4.6% | 2.3% | 2.4% | 11.3% | 0.9% | 5.9% | 3.7% | 1.0% | 8.4% | 12.7% | 13.8% | 6.7% | 8.0% | 3.2% | 0.9% | 8.5% | 4.2% | 1.9% |
|  | k\_\_Bacteria;p\_\_Bacteroidetes;c\_\_Bacteroidia | 1 | 5.2% | 8.4% | 1.6% | 1.8% | 0.9% | 1.7% | 8.9% | 6.7% | 2.4% | 0.2% | 6.4% | 5.8% | 1.2% | 10.3% | 7.2% | 1.1% | 4.0% | 4.7% | 13.0% | 12.1% | 7.2% | 7.9% | 3.2% | 2.0% |
|  | k\_\_Bacteria;p\_\_Bacteroidetes;c\_\_[Saprospirae] | 0 | 1.9% | 0.7% | 0.1% | 5.0% | 4.9% | 0.3% | 1.7% | 0.9% | 0.6% | 2.0% | 0.4% | 1.7% | 1.0% | 0.3% | 3.3% | 8.7% | 3.9% | 3.5% | 1.6% | 0.8% | 0.3% | 1.4% | 1.1% | 0.3% |
|  | No blast hit;Other;Other | 0 | 1.6% | 0.1% | 8.5% | 0.3% | 0.5% | 0.5% | 0.6% | 0.0% | 0.9% | 0.0% | 0.0% | 0.0% | 0.6% | 6.9% | 1.0% | 1.4% | 0.6% | 4.2% | 0.8% | 0.0% | 8.9% | 0.1% | 0.2% | 0.0% |
|  | k\_\_Bacteria;p\_\_Cyanobacteria;c\_\_4C0d-2 | 0 | 0.5% | 0.1% | 0.0% | 0.9% | 1.0% | 0.1% | 0.5% | 0.2% | 0.2% | 0.4% | 0.1% | 0.4% | 0.4% | 0.1% | 0.7% | 1.2% | 1.9% | 0.8% | 0.5% | 0.3% | 0.1% | 0.7% | 0.2% | 0.1% |
|  | k\_\_Bacteria;p\_\_Proteobacteria;c\_\_Deltaproteobacteria | 0 | 0.4% | 0.0% | 0.0% | 0.3% | 0.3% | 0.0% | 0.1% | 0.0% | 0.1% | 0.1% | 0.0% | 0.2% | 0.2% | 0.3% | 0.3% | 0.6% | 1.7% | 0.1% | 0.5% | 0.4% | 0.5% | 2.1% | 0.2% | 0.1% |
|  | k\_\_Bacteria;p\_\_Actinobacteria;c\_\_Coriobacteriia | 0 | 0.2% | 0.0% | 0.0% | 0.0% | 0.0% | 0.0% | 0.0% | 0.0% | 0.0% | 0.1% | 0.0% | 0.1% | 0.0% | 0.0% | 0.0% | 0.0% | 0.3% | 0.1% | 0.0% | 0.0% | 0.1% | 0.1% | 4.1% | 0.0% |
|  | k\_\_Bacteria;p\_\_Tenericutes;c\_\_Mollicutes | 0 | 0.2% | 0.0% | 0.2% | 0.1% | 0.0% | 0.0% | 0.7% | 0.0% | 0.1% | 0.0% | 0.5% | 0.1% | 0.0% | 0.0% | 0.0% | 0.0% | 0.1% | 0.0% | 1.6% | 0.0% | 0.0% | 0.1% | 0.0% | 0.2% |
|  | k\_\_Bacteria;p\_\_Firmicutes;c\_\_Erysipelotrichi | 0 | 0.1% | 0.0% | 0.0% | 0.1% | 0.1% | 0.0% | 0.1% | 0.0% | 0.0% | 0.0% | 0.0% | 0.0% | 0.0% | 0.0% | 0.2% | 0.1% | 0.9% | 0.0% | 0.1% | 0.0% | 0.2% | 0.3% | 0.2% | 0.0% |
|  | k\_\_Bacteria;p\_\_Bacteroidetes;c\_\_Flavobacteriia | 0 | 0.1% | 0.0% | 0.1% | 0.1% | 0.1% | 0.0% | 0.0% | 0.0% | 0.0% | 0.0% | 0.7% | 0.0% | 0.0% | 0.0% | 0.1% | 0.3% | 0.1% | 0.0% | 0.0% | 0.0% | 0.0% | 0.5% | 0.3% | 0.1% |
|  | k\_\_Bacteria;p\_\_Verrucomicrobia;c\_\_Verrucomicrobiae | 0 | 0.1% | 0.0% | 0.0% | 0.1% | 0.0% | 0.0% | 0.0% | 0.0% | 0.1% | 0.0% | 0.0% | 0.1% | 0.1% | 0.0% | 0.0% | 0.0% | 0.3% | 0.0% | 0.0% | 0.0% | 0.3% | 1.0% | 0.1% | 0.0% |
|  | k\_\_Bacteria;p\_\_GN02;c\_\_3BR-5F | 0 | 0.1% | 0.0% | 0.0% | 0.1% | 0.4% | 0.0% | 0.1% | 0.0% | 0.0% | 0.0% | 0.0% | 0.1% | 0.0% | 0.0% | 0.2% | 0.3% | 0.4% | 0.1% | 0.1% | 0.1% | 0.0% | 0.1% | 0.1% | 0.0% |
|  | k\_\_Bacteria;p\_\_Cyanobacteria;c\_\_Chloroplast | 0 | 0.1% | 0.0% | 0.0% | 0.3% | 0.2% | 0.0% | 0.1% | 0.0% | 0.0% | 0.1% | 0.0% | 0.1% | 0.0% | 0.0% | 0.1% | 0.3% | 0.1% | 0.1% | 0.1% | 0.1% | 0.1% | 0.1% | 0.0% | 0.0% |
|  | k\_\_Bacteria;p\_\_Lentisphaerae;c\_\_[Lentisphaeria] | 0 | 0.1% | 0.0% | 0.0% | 0.0% | 0.0% | 0.0% | 0.0% | 0.0% | 0.1% | 0.0% | 0.0% | 0.0% | 0.1% | 0.0% | 0.0% | 0.0% | 0.5% | 0.0% | 0.0% | 0.0% | 0.3% | 0.5% | 0.0% | 0.0% |
|  | k\_\_Bacteria;p\_\_Bacteroidetes;c\_\_Cytophagia | 0 | 0.1% | 0.0% | 0.0% | 0.0% | 0.0% | 0.0% | 0.0% | 0.0% | 0.0% | 0.0% | 0.0% | 0.0% | 0.0% | 1.4% | 0.0% | 0.0% | 0.0% | 0.0% | 0.0% | 0.0% | 0.0% | 0.0% | 0.0% | 0.0% |
|  | k\_\_Bacteria;p\_\_Chloroflexi;c\_\_Anaerolineae | 0 | 0.1% | 0.0% | 0.0% | 0.2% | 0.1% | 0.0% | 0.0% | 0.0% | 0.0% | 0.0% | 0.0% | 0.0% | 0.0% | 0.0% | 0.2% | 0.3% | 0.4% | 0.0% | 0.0% | 0.1% | 0.0% | 0.1% | 0.0% | 0.1% |
|  | k\_\_Bacteria;p\_\_Verrucomicrobia;c\_\_Verruco-5 | 0 | 0.1% | 0.0% | 0.0% | 0.0% | 0.0% | 0.0% | 0.0% | 0.0% | 0.1% | 0.0% | 0.0% | 0.0% | 0.1% | 0.0% | 0.0% | 0.0% | 0.5% | 0.0% | 0.0% | 0.0% | 0.1% | 0.5% | 0.0% | 0.0% |
|  | k\_\_Bacteria;p\_\_Spirochaetes;c\_\_Spirochaetes | 0 | 0.1% | 0.0% | 0.0% | 0.1% | 0.1% | 0.0% | 0.3% | 0.0% | 0.0% | 0.1% | 0.1% | 0.0% | 0.1% | 0.1% | 0.1% | 0.0% | 0.1% | 0.0% | 0.1% | 0.0% | 0.0% | 0.2% | 0.0% | 0.1% |
|  | k\_\_Bacteria;p\_\_SR1;c\_\_Unclassified\_SR1 | 0 | 0.1% | 0.0% | 0.0% | 0.0% | 0.0% | 0.0% | 0.0% | 0.0% | 0.0% | 0.0% | 0.0% | 0.0% | 0.0% | 0.0% | 0.7% | 0.0% | 0.0% | 0.0% | 0.1% | 0.0% | 0.5% | 0.0% | 0.0% | 0.0% |
|  | k\_\_Bacteria;p\_\_[Thermi];c\_\_Deinococci | 0 | 0.1% | 0.0% | 0.0% | 0.2% | 0.1% | 0.0% | 0.1% | 0.0% | 0.0% | 0.1% | 0.0% | 0.0% | 0.0% | 0.0% | 0.1% | 0.2% | 0.2% | 0.1% | 0.0% | 0.0% | 0.0% | 0.1% | 0.0% | 0.0% |
|  | k\_\_Bacteria;p\_\_Planctomycetes;c\_\_Phycisphaerae | 0 | 0.1% | 0.0% | 0.0% | 0.1% | 0.1% | 0.0% | 0.0% | 0.0% | 0.0% | 0.0% | 0.0% | 0.0% | 0.0% | 0.0% | 0.1% | 0.2% | 0.2% | 0.2% | 0.1% | 0.0% | 0.0% | 0.0% | 0.0% | 0.0% |
|  | k\_\_Bacteria;p\_\_Acidobacteria;c\_\_Solibacteres | 0 | 0.1% | 0.0% | 0.0% | 0.1% | 0.1% | 0.0% | 0.0% | 0.0% | 0.0% | 0.0% | 0.0% | 0.0% | 0.0% | 0.0% | 0.0% | 0.1% | 0.2% | 0.0% | 0.1% | 0.0% | 0.0% | 0.2% | 0.1% | 0.0% |
|  | k\_\_Bacteria;p\_\_Acidobacteria;c\_\_Acidobacteria-6 | 0 | 0.1% | 0.0% | 0.0% | 0.1% | 0.0% | 0.0% | 0.0% | 0.0% | 0.0% | 0.0% | 0.0% | 0.0% | 0.0% | 0.0% | 0.0% | 0.1% | 0.0% | 0.0% | 0.0% | 0.0% | 0.0% | 0.8% | 0.0% | 0.0% |
|  | k\_\_Bacteria;p\_\_Gemmatimonadetes;c\_\_Gemmatimonadetes | 0 | 0.0% | 0.0% | 0.0% | 0.0% | 0.0% | 0.0% | 0.0% | 0.0% | 0.0% | 0.0% | 0.0% | 0.1% | 0.0% | 0.0% | 0.0% | 0.1% | 0.0% | 0.0% | 0.0% | 0.0% | 0.0% | 0.8% | 0.1% | 0.0% |
|  | k\_\_Bacteria;p\_\_Gemmatimonadetes;c\_\_Gemm-1 | 0 | 0.0% | 0.0% | 0.0% | 0.0% | 0.0% | 0.0% | 0.0% | 0.0% | 0.0% | 0.0% | 0.0% | 0.0% | 0.0% | 0.0% | 0.0% | 0.0% | 0.1% | 0.0% | 0.0% | 0.0% | 0.0% | 0.8% | 0.1% | 0.0% |
|  | k\_\_Bacteria;p\_\_TM7;c\_\_TM7-3 | 0 | 0.0% | 0.0% | 0.0% | 0.1% | 0.1% | 0.0% | 0.0% | 0.0% | 0.0% | 0.0% | 0.0% | 0.0% | 0.1% | 0.0% | 0.2% | 0.0% | 0.1% | 0.0% | 0.0% | 0.2% | 0.0% | 0.0% | 0.0% | 0.0% |
|  | k\_\_Bacteria;p\_\_Actinobacteria;c\_\_Rubrobacteria | 0 | 0.0% | 0.0% | 0.0% | 0.1% | 0.0% | 0.0% | 0.0% | 0.0% | 0.0% | 0.0% | 0.0% | 0.0% | 0.0% | 0.0% | 0.0% | 0.0% | 0.2% | 0.0% | 0.0% | 0.0% | 0.0% | 0.0% | 0.0% | 0.0% |
|  | k\_\_Bacteria;p\_\_Actinobacteria;c\_\_Thermoleophilia | 0 | 0.0% | 0.0% | 0.0% | 0.2% | 0.1% | 0.0% | 0.0% | 0.0% | 0.0% | 0.0% | 0.0% | 0.0% | 0.0% | 0.0% | 0.0% | 0.0% | 0.0% | 0.0% | 0.0% | 0.0% | 0.0% | 0.1% | 0.0% | 0.0% |
|  | k\_\_Bacteria;p\_\_Actinobacteria;c\_\_Acidimicrobiia | 0 | 0.0% | 0.0% | 0.0% | 0.0% | 0.0% | 0.0% | 0.0% | 0.0% | 0.0% | 0.1% | 0.0% | 0.1% | 0.0% | 0.0% | 0.1% | 0.0% | 0.0% | 0.0% | 0.0% | 0.0% | 0.0% | 0.2% | 0.0% | 0.0% |
|  | k\_\_Bacteria;p\_\_Bacteroidetes;c\_\_Sphingobacteriia | 0 | 0.0% | 0.0% | 0.0% | 0.0% | 0.1% | 0.0% | 0.0% | 0.0% | 0.0% | 0.0% | 0.0% | 0.0% | 0.0% | 0.0% | 0.0% | 0.0% | 0.0% | 0.1% | 0.0% | 0.0% | 0.0% | 0.1% | 0.0% | 0.0% |
|  | k\_\_Bacteria;p\_\_Nitrospirae;c\_\_Nitrospira | 0 | 0.0% | 0.0% | 0.0% | 0.1% | 0.0% | 0.0% | 0.0% | 0.0% | 0.0% | 0.0% | 0.0% | 0.0% | 0.0% | 0.0% | 0.1% | 0.1% | 0.1% | 0.0% | 0.0% | 0.0% | 0.0% | 0.1% | 0.0% | 0.0% |
|  | k\_\_Bacteria;p\_\_Planctomycetes;c\_\_Planctomycetia | 0 | 0.0% | 0.0% | 0.0% | 0.1% | 0.1% | 0.0% | 0.0% | 0.0% | 0.0% | 0.0% | 0.0% | 0.1% | 0.0% | 0.0% | 0.0% | 0.0% | 0.0% | 0.0% | 0.0% | 0.0% | 0.0% | 0.0% | 0.0% | 0.0% |
|  | k\_\_Bacteria;p\_\_Acidobacteria;c\_\_Acidobacteriia | 0 | 0.0% | 0.0% | 0.0% | 0.1% | 0.0% | 0.0% | 0.0% | 0.0% | 0.0% | 0.0% | 0.0% | 0.0% | 0.0% | 0.0% | 0.0% | 0.1% | 0.0% | 0.0% | 0.0% | 0.0% | 0.0% | 0.1% | 0.0% | 0.0% |
|  | k\_\_Bacteria;p\_\_Chlamydiae;c\_\_Chlamydiia | 0 | 0.0% | 0.0% | 0.0% | 0.0% | 0.0% | 0.0% | 0.0% | 0.0% | 0.0% | 0.0% | 0.0% | 0.0% | 0.0% | 0.0% | 0.0% | 0.1% | 0.0% | 0.0% | 0.0% | 0.0% | 0.0% | 0.0% | 0.0% | 0.0% |
|  | k\_\_Bacteria;p\_\_WS3;c\_\_PRR-12 | 0 | 0.0% | 0.0% | 0.0% | 0.1% | 0.0% | 0.0% | 0.0% | 0.0% | 0.0% | 0.0% | 0.0% | 0.0% | 0.0% | 0.0% | 0.0% | 0.0% | 0.1% | 0.0% | 0.0% | 0.0% | 0.0% | 0.0% | 0.0% | 0.0% |
|  | k\_\_Bacteria;p\_\_Acidobacteria;c\_\_[Chloracidobacteria] | 0 | 0.0% | 0.0% | 0.0% | 0.0% | 0.0% | 0.0% | 0.0% | 0.0% | 0.0% | 0.0% | 0.0% | 0.0% | 0.0% | 0.0% | 0.0% | 0.0% | 0.0% | 0.0% | 0.0% | 0.0% | 0.0% | 0.2% | 0.0% | 0.0% |
|  | k\_\_Bacteria;p\_\_Tenericutes;c\_\_RF3 | 0 | 0.0% | 0.0% | 0.0% | 0.0% | 0.0% | 0.0% | 0.0% | 0.0% | 0.0% | 0.0% | 0.0% | 0.0% | 0.0% | 0.0% | 0.0% | 0.0% | 0.0% | 0.0% | 0.0% | 0.0% | 0.1% | 0.1% | 0.0% | 0.0% |
|  | k\_\_Bacteria;p\_\_Chloroflexi;c\_\_Chloroflexi | 0 | 0.0% | 0.0% | 0.0% | 0.2% | 0.0% | 0.0% | 0.0% | 0.0% | 0.0% | 0.0% | 0.0% | 0.0% | 0.0% | 0.0% | 0.0% | 0.0% | 0.0% | 0.0% | 0.0% | 0.0% | 0.0% | 0.0% | 0.0% | 0.0% |
|  | k\_\_Bacteria;p\_\_Armatimonadetes;c\_\_[Fimbriimonadia] | 0 | 0.0% | 0.0% | 0.0% | 0.0% | 0.0% | 0.0% | 0.0% | 0.0% | 0.0% | 0.0% | 0.0% | 0.0% | 0.0% | 0.0% | 0.0% | 0.0% | 0.0% | 0.0% | 0.0% | 0.0% | 0.0% | 0.0% | 0.0% | 0.0% |
|  | k\_\_Bacteria;p\_\_WPS-2;c\_\_Unclassified\_WPS-2 | 0 | 0.0% | 0.0% | 0.0% | 0.0% | 0.0% | 0.0% | 0.0% | 0.0% | 0.0% | 0.0% | 0.0% | 0.0% | 0.0% | 0.0% | 0.0% | 0.0% | 0.0% | 0.0% | 0.0% | 0.0% | 0.0% | 0.0% | 0.0% | 0.0% |
|  | k\_\_Bacteria;p\_\_Chloroflexi;c\_\_S085 | 0 | 0.0% | 0.0% | 0.0% | 0.1% | 0.0% | 0.0% | 0.0% | 0.0% | 0.0% | 0.0% | 0.0% | 0.0% | 0.0% | 0.0% | 0.0% | 0.1% | 0.0% | 0.0% | 0.0% | 0.0% | 0.0% | 0.1% | 0.0% | 0.0% |
|  | k\_\_Bacteria;p\_\_OD1;c\_\_ZB2 | 0 | 0.0% | 0.0% | 0.0% | 0.0% | 0.0% | 0.0% | 0.0% | 0.0% | 0.0% | 0.0% | 0.0% | 0.0% | 0.0% | 0.0% | 0.0% | 0.0% | 0.0% | 0.0% | 0.0% | 0.0% | 0.0% | 0.0% | 0.0% | 0.0% |
|  | k\_\_Bacteria;p\_\_Armatimonadetes;c\_\_Chthonomonadetes | 0 | 0.0% | 0.0% | 0.0% | 0.1% | 0.0% | 0.0% | 0.0% | 0.0% | 0.0% | 0.0% | 0.0% | 0.0% | 0.0% | 0.0% | 0.0% | 0.0% | 0.0% | 0.0% | 0.0% | 0.0% | 0.0% | 0.0% | 0.0% | 0.0% |
|  | k\_\_Bacteria;p\_\_AD3;c\_\_ABS-6 | 0 | 0.0% | 0.0% | 0.0% | 0.0% | 0.0% | 0.0% | 0.0% | 0.0% | 0.0% | 0.0% | 0.0% | 0.0% | 0.0% | 0.0% | 0.0% | 0.0% | 0.1% | 0.0% | 0.0% | 0.0% | 0.0% | 0.0% | 0.0% | 0.0% |
|  | k\_\_Bacteria;p\_\_Tenericutes;c\_\_CK-1C4-19 | 0 | 0.0% | 0.0% | 0.0% | 0.1% | 0.0% | 0.0% | 0.0% | 0.0% | 0.0% | 0.0% | 0.0% | 0.0% | 0.0% | 0.0% | 0.0% | 0.0% | 0.0% | 0.0% | 0.0% | 0.0% | 0.0% | 0.0% | 0.0% | 0.0% |
|  | k\_\_Bacteria;p\_\_Chloroflexi;c\_\_Thermomicrobia | 0 | 0.0% | 0.0% | 0.0% | 0.0% | 0.0% | 0.0% | 0.0% | 0.0% | 0.0% | 0.0% | 0.0% | 0.0% | 0.0% | 0.0% | 0.0% | 0.0% | 0.0% | 0.0% | 0.0% | 0.0% | 0.0% | 0.1% | 0.0% | 0.0% |
|  | k\_\_Bacteria;p\_\_Deferribacteres;c\_\_Deferribacteres | 0 | 0.0% | 0.0% | 0.0% | 0.0% | 0.0% | 0.0% | 0.0% | 0.0% | 0.0% | 0.0% | 0.0% | 0.0% | 0.0% | 0.0% | 0.0% | 0.0% | 0.0% | 0.0% | 0.0% | 0.0% | 0.0% | 0.0% | 0.0% | 0.0% |
|  | k\_\_Bacteria;p\_\_Acidobacteria;c\_\_DA052 | 0 | 0.0% | 0.0% | 0.0% | 0.0% | 0.0% | 0.0% | 0.0% | 0.0% | 0.0% | 0.0% | 0.0% | 0.0% | 0.0% | 0.0% | 0.0% | 0.0% | 0.0% | 0.0% | 0.0% | 0.0% | 0.0% | 0.0% | 0.0% | 0.0% |
|  | k\_\_Bacteria;p\_\_Chloroflexi;c\_\_Ellin6529 | 0 | 0.0% | 0.0% | 0.0% | 0.0% | 0.0% | 0.0% | 0.0% | 0.0% | 0.0% | 0.0% | 0.0% | 0.0% | 0.0% | 0.0% | 0.0% | 0.0% | 0.0% | 0.0% | 0.0% | 0.0% | 0.0% | 0.1% | 0.0% | 0.0% |
|  | k\_\_Bacteria;p\_\_GAL15;c\_\_Unclassified\_GAL15 | 0 | 0.0% | 0.0% | 0.0% | 0.0% | 0.0% | 0.0% | 0.0% | 0.0% | 0.0% | 0.0% | 0.0% | 0.0% | 0.0% | 0.0% | 0.0% | 0.0% | 0.1% | 0.0% | 0.0% | 0.0% | 0.0% | 0.0% | 0.0% | 0.0% |
|  | k\_\_Bacteria;p\_\_Planctomycetes;c\_\_C6 | 0 | 0.0% | 0.0% | 0.0% | 0.0% | 0.0% | 0.0% | 0.0% | 0.0% | 0.0% | 0.0% | 0.0% | 0.0% | 0.0% | 0.0% | 0.0% | 0.0% | 0.0% | 0.0% | 0.0% | 0.0% | 0.0% | 0.1% | 0.0% | 0.0% |
|  | k\_\_Bacteria;p\_\_Chlorobi;c\_\_SJA-28 | 0 | 0.0% | 0.0% | 0.0% | 0.0% | 0.0% | 0.0% | 0.0% | 0.0% | 0.0% | 0.0% | 0.0% | 0.0% | 0.0% | 0.0% | 0.0% | 0.0% | 0.0% | 0.0% | 0.0% | 0.0% | 0.0% | 0.0% | 0.0% | 0.0% |
|  | k\_\_Bacteria;p\_\_Chloroflexi;c\_\_TK17 | 0 | 0.0% | 0.0% | 0.0% | 0.0% | 0.0% | 0.0% | 0.0% | 0.0% | 0.0% | 0.0% | 0.0% | 0.0% | 0.0% | 0.0% | 0.0% | 0.0% | 0.0% | 0.0% | 0.0% | 0.0% | 0.0% | 0.0% | 0.0% | 0.0% |
|  | k\_\_Bacteria;p\_\_Elusimicrobia;c\_\_Elusimicrobia | 0 | 0.0% | 0.0% | 0.0% | 0.0% | 0.0% | 0.0% | 0.0% | 0.0% | 0.0% | 0.0% | 0.0% | 0.0% | 0.0% | 0.0% | 0.0% | 0.0% | 0.0% | 0.0% | 0.0% | 0.0% | 0.0% | 0.0% | 0.0% | 0.0% |
|  | k\_\_Bacteria;p\_\_Chloroflexi;c\_\_Ktedonobacteria | 0 | 0.0% | 0.0% | 0.0% | 0.0% | 0.0% | 0.0% | 0.0% | 0.0% | 0.0% | 0.0% | 0.0% | 0.0% | 0.0% | 0.0% | 0.1% | 0.0% | 0.0% | 0.0% | 0.0% | 0.0% | 0.0% | 0.0% | 0.0% | 0.0% |
|  | k\_\_Bacteria;p\_\_Gemmatimonadetes;c\_\_Gemm-5 | 0 | 0.0% | 0.0% | 0.0% | 0.0% | 0.0% | 0.0% | 0.0% | 0.0% | 0.0% | 0.0% | 0.0% | 0.0% | 0.0% | 0.0% | 0.0% | 0.0% | 0.0% | 0.0% | 0.0% | 0.0% | 0.0% | 0.0% | 0.0% | 0.0% |
|  | k\_\_Bacteria;p\_\_Fibrobacteres;c\_\_Fibrobacteria | 0 | 0.0% | 0.0% | 0.0% | 0.0% | 0.0% | 0.0% | 0.0% | 0.0% | 0.0% | 0.0% | 0.0% | 0.0% | 0.0% | 0.0% | 0.0% | 0.0% | 0.0% | 0.0% | 0.0% | 0.0% | 0.0% | 0.0% | 0.0% | 0.0% |
|  | k\_\_Bacteria;p\_\_Planctomycetes;c\_\_OM190 | 0 | 0.0% | 0.0% | 0.0% | 0.0% | 0.0% | 0.0% | 0.0% | 0.0% | 0.0% | 0.0% | 0.0% | 0.0% | 0.0% | 0.0% | 0.0% | 0.0% | 0.0% | 0.0% | 0.0% | 0.0% | 0.0% | 0.0% | 0.0% | 0.0% |
|  | k\_\_Bacteria;p\_\_Verrucomicrobia;c\_\_Opitutae | 0 | 0.0% | 0.0% | 0.0% | 0.0% | 0.0% | 0.0% | 0.0% | 0.0% | 0.0% | 0.0% | 0.0% | 0.0% | 0.0% | 0.0% | 0.0% | 0.0% | 0.0% | 0.0% | 0.0% | 0.0% | 0.0% | 0.0% | 0.0% | 0.0% |
|  | k\_\_Bacteria;p\_\_TM7;c\_\_TM7-1 | 0 | 0.0% | 0.0% | 0.0% | 0.0% | 0.0% | 0.0% | 0.0% | 0.0% | 0.0% | 0.0% | 0.0% | 0.0% | 0.0% | 0.0% | 0.0% | 0.0% | 0.0% | 0.0% | 0.0% | 0.0% | 0.0% | 0.0% | 0.0% | 0.0% |
|  | k\_\_Bacteria;p\_\_Cyanobacteria;c\_\_ML635J-21 | 0 | 0.0% | 0.0% | 0.0% | 0.0% | 0.0% | 0.0% | 0.0% | 0.0% | 0.0% | 0.0% | 0.0% | 0.0% | 0.0% | 0.0% | 0.0% | 0.0% | 0.0% | 0.0% | 0.0% | 0.0% | 0.0% | 0.0% | 0.0% | 0.0% |
|  | k\_\_Bacteria;p\_\_Cyanobacteria;c\_\_Oscillatoriophycideae | 0 | 0.0% | 0.0% | 0.0% | 0.0% | 0.0% | 0.0% | 0.0% | 0.0% | 0.0% | 0.0% | 0.0% | 0.0% | 0.0% | 0.0% | 0.0% | 0.0% | 0.0% | 0.0% | 0.0% | 0.0% | 0.0% | 0.0% | 0.0% | 0.0% |
|  | k\_\_Bacteria;p\_\_Acidobacteria;c\_\_RB25 | 0 | 0.0% | 0.0% | 0.0% | 0.0% | 0.0% | 0.0% | 0.0% | 0.0% | 0.0% | 0.0% | 0.0% | 0.0% | 0.0% | 0.0% | 0.0% | 0.0% | 0.0% | 0.0% | 0.0% | 0.0% | 0.0% | 0.0% | 0.0% | 0.0% |
|  | k\_\_Bacteria;p\_\_Chloroflexi;c\_\_Gitt-GS-136 | 0 | 0.0% | 0.0% | 0.0% | 0.0% | 0.0% | 0.0% | 0.0% | 0.0% | 0.0% | 0.0% | 0.0% | 0.0% | 0.0% | 0.0% | 0.0% | 0.0% | 0.0% | 0.0% | 0.0% | 0.0% | 0.0% | 0.0% | 0.0% | 0.0% |
|  | k\_\_Bacteria;p\_\_Chloroflexi;c\_\_TK10 | 0 | 0.0% | 0.0% | 0.0% | 0.0% | 0.0% | 0.0% | 0.0% | 0.0% | 0.0% | 0.0% | 0.0% | 0.0% | 0.0% | 0.0% | 0.0% | 0.0% | 0.0% | 0.0% | 0.0% | 0.0% | 0.0% | 0.0% | 0.0% | 0.0% |
|  | k\_\_Bacteria;p\_\_Acidobacteria;c\_\_Sva0725 | 0 | 0.0% | 0.0% | 0.0% | 0.0% | 0.0% | 0.0% | 0.0% | 0.0% | 0.0% | 0.0% | 0.0% | 0.0% | 0.0% | 0.0% | 0.0% | 0.0% | 0.0% | 0.0% | 0.0% | 0.0% | 0.0% | 0.0% | 0.0% | 0.0% |
|  | k\_\_Bacteria;p\_\_Acidobacteria;c\_\_BPC102 | 0 | 0.0% | 0.0% | 0.0% | 0.0% | 0.0% | 0.0% | 0.0% | 0.0% | 0.0% | 0.0% | 0.0% | 0.0% | 0.0% | 0.0% | 0.0% | 0.0% | 0.0% | 0.0% | 0.0% | 0.0% | 0.0% | 0.0% | 0.0% | 0.0% |
|  | k\_\_Bacteria;p\_\_Acidobacteria;c\_\_AT-s54 | 0 | 0.0% | 0.0% | 0.0% | 0.0% | 0.0% | 0.0% | 0.0% | 0.0% | 0.0% | 0.0% | 0.0% | 0.0% | 0.0% | 0.0% | 0.0% | 0.0% | 0.0% | 0.0% | 0.0% | 0.0% | 0.0% | 0.0% | 0.0% | 0.0% |
|  | k\_\_Bacteria;p\_\_OD1;c\_\_ABY1 | 0 | 0.0% | 0.0% | 0.0% | 0.0% | 0.0% | 0.0% | 0.0% | 0.0% | 0.0% | 0.0% | 0.0% | 0.0% | 0.0% | 0.0% | 0.0% | 0.0% | 0.0% | 0.0% | 0.0% | 0.0% | 0.0% | 0.0% | 0.0% | 0.0% |
|  | k\_\_Bacteria;p\_\_Armatimonadetes;c\_\_0319-6E2 | 0 | 0.0% | 0.0% | 0.0% | 0.0% | 0.0% | 0.0% | 0.0% | 0.0% | 0.0% | 0.0% | 0.0% | 0.0% | 0.0% | 0.0% | 0.0% | 0.0% | 0.0% | 0.0% | 0.0% | 0.0% | 0.0% | 0.0% | 0.0% | 0.0% |

|  |  |
| --- | --- |
|  | |
| Taxonomy Summary. Current Level: Order | |
| View Figure (.pdf)  View Legend (.pdf) |  |
|  |


|  |
| --- |
| View Table (.txt) |

|  |  |  |  |  |  |  |  |  |  |  |  |  |  |  |  |  |  |  |  |  |  |  |  |  |  |  |
| --- | --- | --- | --- | --- | --- | --- | --- | --- | --- | --- | --- | --- | --- | --- | --- | --- | --- | --- | --- | --- | --- | --- | --- | --- | --- | --- |
|  | | Total | | A01 | A02 | A03 | A04 | A05 | A06 | A07 | A08 | A09 | A10 | A11 | A12 | A13 | B01 | B02 | B03 | B04 | B05 | B06 | B07 | B08 | B09 | B10 |
| Legend | Taxonomy | count | % | % | % | % | % | % | % | % | % | % | % | % | % | % | % | % | % | % | % | % | % | % | % | % |
|  | k\_\_Bacteria;p\_\_Firmicutes;c\_\_Clostridia;o\_\_Clostridiales | 4 | 19.1% | 17.1% | 33.7% | 13.6% | 8.6% | 35.7% | 10.0% | 34.6% | 14.9% | 5.4% | 13.9% | 13.1% | 9.0% | 18.0% | 27.1% | 3.6% | 16.9% | 6.6% | 14.3% | 55.1% | 19.3% | 24.4% | 36.6% | 6.8% |
|  | k\_\_Bacteria;p\_\_Fusobacteria;c\_\_Fusobacteriia;o\_\_Fusobacteriales | 4 | 16.4% | 40.0% | 20.4% | 6.0% | 5.5% | 0.0% | 28.5% | 7.4% | 17.8% | 7.6% | 28.5% | 12.7% | 18.4% | 16.7% | 7.0% | 12.7% | 4.3% | 32.4% | 25.5% | 0.1% | 31.6% | 9.9% | 7.5% | 37.6% |
|  | k\_\_Bacteria;p\_\_Firmicutes;c\_\_Bacilli;o\_\_Lactobacillales | 3 | 15.0% | 16.3% | 19.6% | 17.8% | 21.2% | 47.7% | 16.8% | 17.7% | 25.9% | 18.8% | 10.2% | 19.3% | 32.1% | 17.1% | 11.9% | 5.3% | 2.4% | 3.6% | 5.0% | 15.8% | 3.9% | 5.7% | 7.0% | 4.3% |
|  | k\_\_Bacteria;p\_\_Proteobacteria;c\_\_Epsilonproteobacteria;o\_\_Campylobacterales | 2 | 9.4% | 6.8% | 6.6% | 1.6% | 3.8% | 0.0% | 10.7% | 10.9% | 15.1% | 17.9% | 26.8% | 10.7% | 7.5% | 17.2% | 2.6% | 4.4% | 3.2% | 6.3% | 9.0% | 0.1% | 16.1% | 3.2% | 0.5% | 34.1% |
|  | k\_\_Bacteria;p\_\_Proteobacteria;c\_\_Alphaproteobacteria;o\_\_Rhizobiales | 2 | 8.1% | 3.3% | 0.7% | 20.2% | 21.5% | 3.1% | 6.8% | 3.6% | 3.8% | 13.1% | 1.2% | 9.7% | 5.9% | 1.4% | 11.2% | 23.1% | 18.1% | 13.4% | 7.2% | 4.2% | 1.2% | 6.9% | 5.0% | 1.3% |
|  | k\_\_Bacteria;p\_\_Actinobacteria;c\_\_Actinobacteria;o\_\_Actinomycetales | 1 | 6.4% | 1.9% | 8.0% | 4.0% | 11.3% | 6.5% | 4.4% | 12.3% | 10.7% | 7.5% | 5.6% | 7.0% | 10.4% | 6.9% | 2.5% | 5.0% | 5.8% | 4.3% | 6.6% | 4.0% | 3.6% | 3.0% | 6.9% | 8.0% |
|  | k\_\_Bacteria;p\_\_Bacteroidetes;c\_\_Bacteroidia;o\_\_Bacteroidales | 1 | 5.2% | 8.4% | 1.6% | 1.8% | 0.9% | 1.7% | 8.9% | 6.7% | 2.4% | 0.2% | 6.4% | 5.8% | 1.2% | 10.3% | 7.2% | 1.1% | 4.0% | 4.7% | 13.0% | 12.1% | 7.2% | 7.9% | 3.2% | 2.0% |
|  | k\_\_Bacteria;p\_\_Proteobacteria;c\_\_Betaproteobacteria;o\_\_Burkholderiales | 1 | 4.6% | 1.6% | 0.2% | 9.9% | 7.2% | 0.8% | 4.3% | 2.1% | 2.2% | 11.1% | 0.9% | 5.5% | 3.5% | 1.0% | 7.8% | 10.9% | 12.0% | 6.2% | 4.2% | 2.1% | 0.8% | 7.1% | 3.8% | 1.6% |
|  | k\_\_Bacteria;p\_\_Proteobacteria;c\_\_Gammaproteobacteria;o\_\_Pseudomonadales | 1 | 3.1% | 0.3% | 0.0% | 4.6% | 2.1% | 2.7% | 1.5% | 1.6% | 1.0% | 10.5% | 3.6% | 7.7% | 4.6% | 1.5% | 3.5% | 1.9% | 3.8% | 2.7% | 1.7% | 0.5% | 2.7% | 3.4% | 8.5% | 2.0% |
|  | k\_\_Bacteria;p\_\_Proteobacteria;c\_\_Gammaproteobacteria;o\_\_Xanthomonadales | 0 | 2.1% | 0.7% | 0.1% | 4.1% | 2.6% | 0.4% | 1.5% | 0.6% | 0.6% | 2.4% | 0.3% | 1.3% | 1.0% | 0.2% | 2.7% | 9.3% | 6.7% | 5.7% | 1.7% | 1.2% | 0.5% | 1.9% | 1.5% | 0.4% |
|  | k\_\_Bacteria;p\_\_Bacteroidetes;c\_\_[Saprospirae];o\_\_[Saprospirales] | 0 | 1.9% | 0.7% | 0.1% | 5.0% | 4.9% | 0.3% | 1.7% | 0.9% | 0.6% | 2.0% | 0.4% | 1.7% | 1.0% | 0.3% | 3.3% | 8.7% | 3.9% | 3.5% | 1.6% | 0.8% | 0.3% | 1.4% | 1.1% | 0.3% |
|  | No blast hit;Other;Other;Other | 0 | 1.6% | 0.1% | 8.5% | 0.3% | 0.5% | 0.5% | 0.6% | 0.0% | 0.9% | 0.0% | 0.0% | 0.0% | 0.6% | 6.9% | 1.0% | 1.4% | 0.6% | 4.2% | 0.8% | 0.0% | 8.9% | 0.1% | 0.2% | 0.0% |
|  | k\_\_Bacteria;p\_\_Proteobacteria;c\_\_Alphaproteobacteria;o\_\_Sphingomonadales | 0 | 1.1% | 0.3% | 0.1% | 2.5% | 2.5% | 0.1% | 0.9% | 0.4% | 0.4% | 1.0% | 0.2% | 1.9% | 0.8% | 0.2% | 2.4% | 3.1% | 2.6% | 1.7% | 0.8% | 0.6% | 0.3% | 1.1% | 0.9% | 0.2% |
|  | k\_\_Bacteria;p\_\_Proteobacteria;c\_\_Gammaproteobacteria;o\_\_Enterobacteriales | 0 | 0.9% | 0.2% | 0.0% | 0.5% | 1.5% | 0.0% | 0.0% | 0.1% | 2.1% | 0.1% | 0.0% | 0.0% | 1.8% | 0.0% | 3.6% | 0.2% | 1.8% | 0.0% | 0.2% | 0.5% | 0.1% | 6.2% | 1.7% | 0.0% |
|  | k\_\_Bacteria;p\_\_Proteobacteria;c\_\_Alphaproteobacteria;o\_\_Caulobacterales | 0 | 0.6% | 0.3% | 0.1% | 1.5% | 1.2% | 0.1% | 0.5% | 0.4% | 0.3% | 0.6% | 0.1% | 0.8% | 0.5% | 0.1% | 1.1% | 2.0% | 1.6% | 1.1% | 0.5% | 0.3% | 0.1% | 0.7% | 0.6% | 0.1% |
|  | k\_\_Bacteria;p\_\_Cyanobacteria;c\_\_4C0d-2;o\_\_MLE1-12 | 0 | 0.5% | 0.1% | 0.0% | 0.9% | 1.0% | 0.1% | 0.5% | 0.2% | 0.2% | 0.4% | 0.1% | 0.4% | 0.3% | 0.1% | 0.7% | 1.2% | 1.9% | 0.7% | 0.5% | 0.3% | 0.1% | 0.4% | 0.2% | 0.1% |
|  | k\_\_Bacteria;p\_\_Proteobacteria;c\_\_Betaproteobacteria;o\_\_Rhodocyclales | 0 | 0.4% | 0.2% | 0.0% | 0.8% | 0.6% | 0.0% | 0.3% | 0.2% | 0.1% | 0.2% | 0.0% | 0.4% | 0.2% | 0.0% | 0.6% | 1.3% | 1.5% | 0.5% | 0.4% | 0.2% | 0.1% | 0.6% | 0.3% | 0.1% |
|  | k\_\_Bacteria;p\_\_Firmicutes;c\_\_Bacilli;o\_\_Bacillales | 0 | 0.3% | 0.2% | 0.0% | 0.4% | 0.3% | 0.0% | 0.2% | 0.1% | 0.1% | 0.2% | 0.1% | 0.2% | 0.1% | 0.0% | 0.4% | 0.3% | 0.8% | 0.7% | 0.1% | 0.1% | 0.1% | 1.4% | 1.7% | 0.0% |
|  | k\_\_Bacteria;p\_\_Actinobacteria;c\_\_Actinobacteria;o\_\_Bifidobacteriales | 0 | 0.3% | 0.0% | 0.0% | 0.0% | 0.0% | 0.1% | 0.0% | 0.0% | 0.0% | 0.0% | 0.0% | 0.1% | 0.0% | 0.0% | 0.1% | 0.0% | 0.0% | 0.0% | 0.0% | 0.0% | 0.0% | 0.1% | 6.2% | 0.0% |
|  | k\_\_Bacteria;p\_\_Actinobacteria;c\_\_Coriobacteriia;o\_\_Coriobacteriales | 0 | 0.2% | 0.0% | 0.0% | 0.0% | 0.0% | 0.0% | 0.0% | 0.0% | 0.0% | 0.1% | 0.0% | 0.1% | 0.0% | 0.0% | 0.0% | 0.0% | 0.3% | 0.1% | 0.0% | 0.0% | 0.1% | 0.1% | 4.1% | 0.0% |
|  | k\_\_Bacteria;p\_\_Proteobacteria;c\_\_Betaproteobacteria;o\_\_Neisseriales | 0 | 0.2% | 0.0% | 0.0% | 0.0% | 0.2% | 0.0% | 0.0% | 0.0% | 0.0% | 0.0% | 0.0% | 0.0% | 0.0% | 0.0% | 0.0% | 0.5% | 0.0% | 0.0% | 3.4% | 0.8% | 0.0% | 0.0% | 0.0% | 0.0% |
|  | k\_\_Bacteria;p\_\_Proteobacteria;c\_\_Gammaproteobacteria;o\_\_Pasteurellales | 0 | 0.2% | 1.2% | 0.0% | 0.2% | 0.0% | 0.0% | 0.0% | 0.0% | 0.0% | 0.0% | 0.0% | 0.0% | 0.0% | 0.0% | 0.0% | 0.0% | 0.0% | 0.0% | 0.0% | 0.1% | 0.1% | 3.0% | 0.3% | 0.0% |
|  | k\_\_Bacteria;p\_\_Proteobacteria;c\_\_Deltaproteobacteria;o\_\_Desulfovibrionales | 0 | 0.2% | 0.0% | 0.0% | 0.0% | 0.0% | 0.0% | 0.0% | 0.0% | 0.1% | 0.0% | 0.0% | 0.0% | 0.1% | 0.2% | 0.0% | 0.0% | 0.7% | 0.0% | 0.2% | 0.3% | 0.4% | 1.3% | 0.1% | 0.0% |
|  | k\_\_Bacteria;p\_\_Proteobacteria;c\_\_Deltaproteobacteria;o\_\_Myxococcales | 0 | 0.1% | 0.0% | 0.0% | 0.2% | 0.2% | 0.0% | 0.0% | 0.0% | 0.0% | 0.1% | 0.0% | 0.1% | 0.0% | 0.0% | 0.2% | 0.4% | 0.6% | 0.0% | 0.2% | 0.1% | 0.0% | 0.6% | 0.1% | 0.0% |
|  | k\_\_Bacteria;p\_\_Tenericutes;c\_\_Mollicutes;o\_\_Acholeplasmatales | 0 | 0.1% | 0.0% | 0.2% | 0.1% | 0.0% | 0.0% | 0.7% | 0.0% | 0.0% | 0.0% | 0.5% | 0.0% | 0.0% | 0.0% | 0.0% | 0.0% | 0.0% | 0.0% | 1.2% | 0.0% | 0.0% | 0.0% | 0.0% | 0.0% |
|  | k\_\_Bacteria;p\_\_Firmicutes;c\_\_Erysipelotrichi;o\_\_Erysipelotrichales | 0 | 0.1% | 0.0% | 0.0% | 0.1% | 0.1% | 0.0% | 0.1% | 0.0% | 0.0% | 0.0% | 0.0% | 0.0% | 0.0% | 0.0% | 0.2% | 0.1% | 0.9% | 0.0% | 0.1% | 0.0% | 0.2% | 0.3% | 0.2% | 0.0% |
|  | k\_\_Bacteria;p\_\_Bacteroidetes;c\_\_Flavobacteriia;o\_\_Flavobacteriales | 0 | 0.1% | 0.0% | 0.1% | 0.1% | 0.1% | 0.0% | 0.0% | 0.0% | 0.0% | 0.0% | 0.7% | 0.0% | 0.0% | 0.0% | 0.1% | 0.3% | 0.1% | 0.0% | 0.0% | 0.0% | 0.0% | 0.5% | 0.3% | 0.1% |
|  | k\_\_Bacteria;p\_\_Proteobacteria;c\_\_Alphaproteobacteria;o\_\_Rhodospirillales | 0 | 0.1% | 0.0% | 0.0% | 0.3% | 0.1% | 0.0% | 0.1% | 0.0% | 0.0% | 0.1% | 0.0% | 0.1% | 0.0% | 0.0% | 0.3% | 0.2% | 0.2% | 0.1% | 0.1% | 0.1% | 0.0% | 0.2% | 0.1% | 0.0% |
|  | k\_\_Bacteria;p\_\_Verrucomicrobia;c\_\_Verrucomicrobiae;o\_\_Verrucomicrobiales | 0 | 0.1% | 0.0% | 0.0% | 0.1% | 0.0% | 0.0% | 0.0% | 0.0% | 0.1% | 0.0% | 0.0% | 0.1% | 0.1% | 0.0% | 0.0% | 0.0% | 0.3% | 0.0% | 0.0% | 0.0% | 0.3% | 1.0% | 0.1% | 0.0% |
|  | k\_\_Bacteria;p\_\_GN02;c\_\_3BR-5F;o\_\_Unclassified\_3BR-5F | 0 | 0.1% | 0.0% | 0.0% | 0.1% | 0.4% | 0.0% | 0.1% | 0.0% | 0.0% | 0.0% | 0.0% | 0.1% | 0.0% | 0.0% | 0.2% | 0.3% | 0.4% | 0.1% | 0.1% | 0.1% | 0.0% | 0.1% | 0.1% | 0.0% |
|  | k\_\_Bacteria;p\_\_Cyanobacteria;c\_\_Chloroplast;o\_\_Streptophyta | 0 | 0.1% | 0.0% | 0.0% | 0.3% | 0.1% | 0.0% | 0.1% | 0.0% | 0.0% | 0.1% | 0.0% | 0.1% | 0.0% | 0.0% | 0.1% | 0.2% | 0.1% | 0.1% | 0.1% | 0.1% | 0.1% | 0.1% | 0.0% | 0.0% |
|  | k\_\_Bacteria;p\_\_Proteobacteria;c\_\_Gammaproteobacteria;o\_\_Aeromonadales | 0 | 0.1% | 0.0% | 0.0% | 0.4% | 0.0% | 0.0% | 0.0% | 0.0% | 0.0% | 0.0% | 0.0% | 0.1% | 0.0% | 0.0% | 0.0% | 0.3% | 0.2% | 0.1% | 0.1% | 0.0% | 0.0% | 0.1% | 0.1% | 0.0% |
|  | k\_\_Bacteria;p\_\_Bacteroidetes;c\_\_Cytophagia;o\_\_Cytophagales | 0 | 0.1% | 0.0% | 0.0% | 0.0% | 0.0% | 0.0% | 0.0% | 0.0% | 0.0% | 0.0% | 0.0% | 0.0% | 0.0% | 1.4% | 0.0% | 0.0% | 0.0% | 0.0% | 0.0% | 0.0% | 0.0% | 0.0% | 0.0% | 0.0% |
|  | k\_\_Bacteria;p\_\_Verrucomicrobia;c\_\_Verruco-5;o\_\_WCHB1-41 | 0 | 0.1% | 0.0% | 0.0% | 0.0% | 0.0% | 0.0% | 0.0% | 0.0% | 0.1% | 0.0% | 0.0% | 0.0% | 0.1% | 0.0% | 0.0% | 0.0% | 0.5% | 0.0% | 0.0% | 0.0% | 0.1% | 0.5% | 0.0% | 0.0% |
|  | k\_\_Bacteria;p\_\_SR1;c\_\_Unclassified\_SR1;o\_\_Unclassified\_SR1 | 0 | 0.1% | 0.0% | 0.0% | 0.0% | 0.0% | 0.0% | 0.0% | 0.0% | 0.0% | 0.0% | 0.0% | 0.0% | 0.0% | 0.0% | 0.7% | 0.0% | 0.0% | 0.0% | 0.1% | 0.0% | 0.5% | 0.0% | 0.0% | 0.0% |
|  | k\_\_Bacteria;p\_\_Lentisphaerae;c\_\_[Lentisphaeria];o\_\_Victivallales | 0 | 0.1% | 0.0% | 0.0% | 0.0% | 0.0% | 0.0% | 0.0% | 0.0% | 0.1% | 0.0% | 0.0% | 0.0% | 0.0% | 0.0% | 0.0% | 0.0% | 0.5% | 0.0% | 0.0% | 0.0% | 0.1% | 0.5% | 0.0% | 0.0% |
|  | k\_\_Bacteria;p\_\_Spirochaetes;c\_\_Spirochaetes;o\_\_Spirochaetales | 0 | 0.1% | 0.0% | 0.0% | 0.1% | 0.1% | 0.0% | 0.1% | 0.0% | 0.0% | 0.1% | 0.1% | 0.0% | 0.1% | 0.1% | 0.1% | 0.0% | 0.1% | 0.0% | 0.1% | 0.0% | 0.0% | 0.2% | 0.0% | 0.1% |
|  | k\_\_Bacteria;p\_\_Planctomycetes;c\_\_Phycisphaerae;o\_\_Phycisphaerales | 0 | 0.1% | 0.0% | 0.0% | 0.1% | 0.1% | 0.0% | 0.0% | 0.0% | 0.0% | 0.0% | 0.0% | 0.0% | 0.0% | 0.0% | 0.1% | 0.2% | 0.2% | 0.2% | 0.1% | 0.0% | 0.0% | 0.0% | 0.0% | 0.0% |
|  | k\_\_Bacteria;p\_\_[Thermi];c\_\_Deinococci;o\_\_Deinococcales | 0 | 0.1% | 0.0% | 0.0% | 0.1% | 0.1% | 0.0% | 0.1% | 0.0% | 0.0% | 0.0% | 0.0% | 0.0% | 0.0% | 0.0% | 0.1% | 0.2% | 0.2% | 0.1% | 0.0% | 0.0% | 0.0% | 0.1% | 0.0% | 0.0% |
|  | k\_\_Bacteria;p\_\_Acidobacteria;c\_\_Solibacteres;o\_\_Solibacterales | 0 | 0.1% | 0.0% | 0.0% | 0.1% | 0.1% | 0.0% | 0.0% | 0.0% | 0.0% | 0.0% | 0.0% | 0.0% | 0.0% | 0.0% | 0.0% | 0.1% | 0.2% | 0.0% | 0.1% | 0.0% | 0.0% | 0.2% | 0.1% | 0.0% |
|  | k\_\_Bacteria;p\_\_Gemmatimonadetes;c\_\_Gemm-1;o\_\_Unclassified\_Gemm-1 | 0 | 0.0% | 0.0% | 0.0% | 0.0% | 0.0% | 0.0% | 0.0% | 0.0% | 0.0% | 0.0% | 0.0% | 0.0% | 0.0% | 0.0% | 0.0% | 0.0% | 0.1% | 0.0% | 0.0% | 0.0% | 0.0% | 0.8% | 0.1% | 0.0% |
|  | k\_\_Bacteria;p\_\_Proteobacteria;c\_\_Alphaproteobacteria;o\_\_Rhodobacterales | 0 | 0.0% | 0.0% | 0.0% | 0.1% | 0.1% | 0.1% | 0.0% | 0.0% | 0.0% | 0.0% | 0.0% | 0.0% | 0.0% | 0.0% | 0.0% | 0.1% | 0.1% | 0.1% | 0.0% | 0.0% | 0.0% | 0.1% | 0.0% | 0.2% |
|  | k\_\_Bacteria;p\_\_Acidobacteria;c\_\_Acidobacteria-6;o\_\_iii1-15 | 0 | 0.0% | 0.0% | 0.0% | 0.1% | 0.0% | 0.0% | 0.0% | 0.0% | 0.0% | 0.0% | 0.0% | 0.0% | 0.0% | 0.0% | 0.0% | 0.1% | 0.0% | 0.0% | 0.0% | 0.0% | 0.0% | 0.7% | 0.0% | 0.0% |
|  | k\_\_Bacteria;p\_\_Chloroflexi;c\_\_Anaerolineae;o\_\_SBR1031 | 0 | 0.0% | 0.0% | 0.0% | 0.1% | 0.1% | 0.0% | 0.0% | 0.0% | 0.0% | 0.0% | 0.0% | 0.0% | 0.0% | 0.0% | 0.1% | 0.2% | 0.0% | 0.0% | 0.0% | 0.1% | 0.0% | 0.1% | 0.0% | 0.1% |
|  | k\_\_Bacteria;p\_\_TM7;c\_\_TM7-3;o\_\_CW040 | 0 | 0.0% | 0.0% | 0.0% | 0.1% | 0.1% | 0.0% | 0.0% | 0.0% | 0.0% | 0.0% | 0.0% | 0.0% | 0.1% | 0.0% | 0.2% | 0.0% | 0.1% | 0.0% | 0.0% | 0.0% | 0.0% | 0.0% | 0.0% | 0.0% |
|  | k\_\_Bacteria;p\_\_Proteobacteria;c\_\_Betaproteobacteria;o\_\_SC-I-84 | 0 | 0.0% | 0.0% | 0.0% | 0.0% | 0.0% | 0.0% | 0.0% | 0.0% | 0.0% | 0.0% | 0.0% | 0.0% | 0.0% | 0.0% | 0.0% | 0.0% | 0.0% | 0.0% | 0.0% | 0.0% | 0.0% | 0.5% | 0.0% | 0.0% |
|  | k\_\_Bacteria;p\_\_Cyanobacteria;c\_\_4C0d-2;o\_\_YS2 | 0 | 0.0% | 0.0% | 0.0% | 0.0% | 0.0% | 0.0% | 0.0% | 0.0% | 0.0% | 0.0% | 0.0% | 0.0% | 0.1% | 0.0% | 0.0% | 0.0% | 0.1% | 0.0% | 0.0% | 0.0% | 0.0% | 0.3% | 0.0% | 0.0% |
|  | k\_\_Bacteria;p\_\_Actinobacteria;c\_\_Rubrobacteria;o\_\_Rubrobacterales | 0 | 0.0% | 0.0% | 0.0% | 0.1% | 0.0% | 0.0% | 0.0% | 0.0% | 0.0% | 0.0% | 0.0% | 0.0% | 0.0% | 0.0% | 0.0% | 0.0% | 0.2% | 0.0% | 0.0% | 0.0% | 0.0% | 0.0% | 0.0% | 0.0% |
|  | k\_\_Bacteria;p\_\_Actinobacteria;c\_\_Acidimicrobiia;o\_\_Acidimicrobiales | 0 | 0.0% | 0.0% | 0.0% | 0.0% | 0.0% | 0.0% | 0.0% | 0.0% | 0.0% | 0.1% | 0.0% | 0.1% | 0.0% | 0.0% | 0.1% | 0.0% | 0.0% | 0.0% | 0.0% | 0.0% | 0.0% | 0.2% | 0.0% | 0.0% |
|  | k\_\_Bacteria;p\_\_Firmicutes;c\_\_Bacilli;o\_\_Turicibacterales | 0 | 0.0% | 0.0% | 0.0% | 0.0% | 0.0% | 0.0% | 0.0% | 0.0% | 0.0% | 0.0% | 0.0% | 0.1% | 0.0% | 0.0% | 0.1% | 0.0% | 0.1% | 0.0% | 0.0% | 0.0% | 0.0% | 0.1% | 0.1% | 0.0% |
|  | k\_\_Bacteria;p\_\_Bacteroidetes;c\_\_Sphingobacteriia;o\_\_Sphingobacteriales | 0 | 0.0% | 0.0% | 0.0% | 0.0% | 0.1% | 0.0% | 0.0% | 0.0% | 0.0% | 0.0% | 0.0% | 0.0% | 0.0% | 0.0% | 0.0% | 0.0% | 0.0% | 0.1% | 0.0% | 0.0% | 0.0% | 0.1% | 0.0% | 0.0% |
|  | k\_\_Bacteria;p\_\_Nitrospirae;c\_\_Nitrospira;o\_\_Nitrospirales | 0 | 0.0% | 0.0% | 0.0% | 0.1% | 0.0% | 0.0% | 0.0% | 0.0% | 0.0% | 0.0% | 0.0% | 0.0% | 0.0% | 0.0% | 0.1% | 0.1% | 0.1% | 0.0% | 0.0% | 0.0% | 0.0% | 0.1% | 0.0% | 0.0% |
|  | k\_\_Bacteria;p\_\_Tenericutes;c\_\_Mollicutes;o\_\_Mycoplasmatales | 0 | 0.0% | 0.0% | 0.0% | 0.0% | 0.0% | 0.0% | 0.0% | 0.0% | 0.0% | 0.0% | 0.0% | 0.0% | 0.0% | 0.0% | 0.0% | 0.0% | 0.0% | 0.0% | 0.3% | 0.0% | 0.0% | 0.0% | 0.0% | 0.1% |
|  | k\_\_Bacteria;p\_\_Proteobacteria;c\_\_Gammaproteobacteria;o\_\_Cardiobacteriales | 0 | 0.0% | 0.0% | 0.0% | 0.0% | 0.0% | 0.0% | 0.0% | 0.0% | 0.0% | 0.0% | 0.0% | 0.0% | 0.0% | 0.0% | 0.0% | 0.0% | 0.0% | 0.0% | 0.0% | 0.0% | 0.4% | 0.0% | 0.0% | 0.0% |
|  | k\_\_Bacteria;p\_\_Tenericutes;c\_\_Mollicutes;o\_\_RF39 | 0 | 0.0% | 0.0% | 0.0% | 0.0% | 0.0% | 0.0% | 0.0% | 0.0% | 0.1% | 0.0% | 0.0% | 0.1% | 0.0% | 0.0% | 0.0% | 0.0% | 0.1% | 0.0% | 0.0% | 0.0% | 0.0% | 0.1% | 0.0% | 0.0% |
|  | k\_\_Bacteria;p\_\_Gemmatimonadetes;c\_\_Gemmatimonadetes;o\_\_Gemmatimonadales | 0 | 0.0% | 0.0% | 0.0% | 0.0% | 0.0% | 0.0% | 0.0% | 0.0% | 0.0% | 0.0% | 0.0% | 0.1% | 0.0% | 0.0% | 0.0% | 0.1% | 0.0% | 0.0% | 0.0% | 0.0% | 0.0% | 0.2% | 0.0% | 0.0% |
|  | k\_\_Bacteria;p\_\_Gemmatimonadetes;c\_\_Gemmatimonadetes;o\_\_Unclassified\_Gemmatimonadetes | 0 | 0.0% | 0.0% | 0.0% | 0.0% | 0.0% | 0.0% | 0.0% | 0.0% | 0.0% | 0.0% | 0.0% | 0.0% | 0.0% | 0.0% | 0.0% | 0.1% | 0.0% | 0.0% | 0.0% | 0.0% | 0.0% | 0.2% | 0.0% | 0.0% |
|  | k\_\_Bacteria;p\_\_Planctomycetes;c\_\_Planctomycetia;o\_\_Gemmatales | 0 | 0.0% | 0.0% | 0.0% | 0.1% | 0.1% | 0.0% | 0.0% | 0.0% | 0.0% | 0.0% | 0.0% | 0.1% | 0.0% | 0.0% | 0.0% | 0.0% | 0.0% | 0.0% | 0.0% | 0.0% | 0.0% | 0.0% | 0.0% | 0.0% |
|  | k\_\_Bacteria;p\_\_Acidobacteria;c\_\_Acidobacteriia;o\_\_Acidobacteriales | 0 | 0.0% | 0.0% | 0.0% | 0.1% | 0.0% | 0.0% | 0.0% | 0.0% | 0.0% | 0.0% | 0.0% | 0.0% | 0.0% | 0.0% | 0.0% | 0.1% | 0.0% | 0.0% | 0.0% | 0.0% | 0.0% | 0.1% | 0.0% | 0.0% |
|  | k\_\_Bacteria;p\_\_Proteobacteria;c\_\_Alphaproteobacteria;o\_\_Unclassified\_Alphaproteobacteria | 0 | 0.0% | 0.0% | 0.0% | 0.0% | 0.0% | 0.0% | 0.0% | 0.0% | 0.0% | 0.0% | 0.0% | 0.0% | 0.0% | 0.0% | 0.0% | 0.0% | 0.0% | 0.0% | 0.0% | 0.0% | 0.1% | 0.1% | 0.0% | 0.0% |
|  | k\_\_Bacteria;p\_\_Proteobacteria;c\_\_Alphaproteobacteria;o\_\_Rickettsiales | 0 | 0.0% | 0.0% | 0.0% | 0.0% | 0.1% | 0.0% | 0.0% | 0.0% | 0.0% | 0.0% | 0.0% | 0.0% | 0.0% | 0.0% | 0.0% | 0.0% | 0.1% | 0.0% | 0.0% | 0.0% | 0.0% | 0.0% | 0.0% | 0.0% |
|  | k\_\_Bacteria;p\_\_Firmicutes;c\_\_Bacilli;o\_\_Gemellales | 0 | 0.0% | 0.2% | 0.0% | 0.0% | 0.0% | 0.0% | 0.0% | 0.0% | 0.0% | 0.0% | 0.0% | 0.0% | 0.0% | 0.0% | 0.0% | 0.0% | 0.0% | 0.0% | 0.0% | 0.0% | 0.0% | 0.0% | 0.0% | 0.0% |
|  | k\_\_Bacteria;p\_\_Proteobacteria;c\_\_Betaproteobacteria;o\_\_MND1 | 0 | 0.0% | 0.0% | 0.0% | 0.0% | 0.0% | 0.0% | 0.0% | 0.0% | 0.0% | 0.0% | 0.0% | 0.0% | 0.0% | 0.0% | 0.0% | 0.0% | 0.1% | 0.0% | 0.0% | 0.0% | 0.0% | 0.2% | 0.0% | 0.0% |
|  | k\_\_Bacteria;p\_\_Actinobacteria;c\_\_Thermoleophilia;o\_\_Gaiellales | 0 | 0.0% | 0.0% | 0.0% | 0.2% | 0.0% | 0.0% | 0.0% | 0.0% | 0.0% | 0.0% | 0.0% | 0.0% | 0.0% | 0.0% | 0.0% | 0.0% | 0.0% | 0.0% | 0.0% | 0.0% | 0.0% | 0.0% | 0.0% | 0.0% |
|  | k\_\_Bacteria;p\_\_Proteobacteria;c\_\_Deltaproteobacteria;o\_\_Syntrophobacterales | 0 | 0.0% | 0.0% | 0.0% | 0.0% | 0.0% | 0.0% | 0.0% | 0.0% | 0.0% | 0.0% | 0.0% | 0.0% | 0.0% | 0.0% | 0.0% | 0.1% | 0.1% | 0.0% | 0.0% | 0.0% | 0.0% | 0.1% | 0.0% | 0.0% |
|  | k\_\_Bacteria;p\_\_Lentisphaerae;c\_\_[Lentisphaeria];o\_\_Unclassified\_[Lentisphaeria] | 0 | 0.0% | 0.0% | 0.0% | 0.0% | 0.0% | 0.0% | 0.0% | 0.0% | 0.0% | 0.0% | 0.0% | 0.0% | 0.1% | 0.0% | 0.0% | 0.0% | 0.0% | 0.0% | 0.0% | 0.0% | 0.2% | 0.0% | 0.0% | 0.0% |
|  | k\_\_Bacteria;p\_\_Chlamydiae;c\_\_Chlamydiia;o\_\_Chlamydiales | 0 | 0.0% | 0.0% | 0.0% | 0.0% | 0.0% | 0.0% | 0.0% | 0.0% | 0.0% | 0.0% | 0.0% | 0.0% | 0.0% | 0.0% | 0.0% | 0.1% | 0.0% | 0.0% | 0.0% | 0.0% | 0.0% | 0.0% | 0.0% | 0.0% |
|  | k\_\_Bacteria;p\_\_WS3;c\_\_PRR-12;o\_\_Sediment-1 | 0 | 0.0% | 0.0% | 0.0% | 0.1% | 0.0% | 0.0% | 0.0% | 0.0% | 0.0% | 0.0% | 0.0% | 0.0% | 0.0% | 0.0% | 0.0% | 0.0% | 0.1% | 0.0% | 0.0% | 0.0% | 0.0% | 0.0% | 0.0% | 0.0% |
|  | k\_\_Bacteria;p\_\_TM7;c\_\_TM7-3;o\_\_Unclassified\_TM7-3 | 0 | 0.0% | 0.0% | 0.0% | 0.0% | 0.0% | 0.0% | 0.0% | 0.0% | 0.0% | 0.0% | 0.0% | 0.0% | 0.0% | 0.0% | 0.1% | 0.0% | 0.0% | 0.0% | 0.0% | 0.2% | 0.0% | 0.0% | 0.0% | 0.0% |
|  | k\_\_Bacteria;p\_\_Proteobacteria;c\_\_Deltaproteobacteria;o\_\_MIZ46 | 0 | 0.0% | 0.0% | 0.0% | 0.0% | 0.0% | 0.0% | 0.0% | 0.0% | 0.0% | 0.0% | 0.0% | 0.0% | 0.0% | 0.0% | 0.0% | 0.1% | 0.0% | 0.0% | 0.0% | 0.0% | 0.0% | 0.0% | 0.0% | 0.0% |
|  | k\_\_Bacteria;p\_\_Gemmatimonadetes;c\_\_Gemmatimonadetes;o\_\_N1423WL | 0 | 0.0% | 0.0% | 0.0% | 0.0% | 0.0% | 0.0% | 0.0% | 0.0% | 0.0% | 0.0% | 0.0% | 0.0% | 0.0% | 0.0% | 0.0% | 0.0% | 0.0% | 0.0% | 0.0% | 0.0% | 0.0% | 0.2% | 0.0% | 0.0% |
|  | k\_\_Bacteria;p\_\_Proteobacteria;c\_\_Deltaproteobacteria;o\_\_Bdellovibrionales | 0 | 0.0% | 0.0% | 0.0% | 0.0% | 0.0% | 0.0% | 0.0% | 0.0% | 0.0% | 0.0% | 0.0% | 0.0% | 0.0% | 0.0% | 0.0% | 0.0% | 0.1% | 0.0% | 0.0% | 0.0% | 0.0% | 0.0% | 0.0% | 0.0% |
|  | k\_\_Bacteria;p\_\_Proteobacteria;c\_\_Deltaproteobacteria;o\_\_NB1-j | 0 | 0.0% | 0.0% | 0.0% | 0.0% | 0.0% | 0.0% | 0.0% | 0.0% | 0.0% | 0.0% | 0.0% | 0.0% | 0.0% | 0.0% | 0.0% | 0.0% | 0.1% | 0.0% | 0.0% | 0.0% | 0.0% | 0.0% | 0.0% | 0.0% |
|  | k\_\_Bacteria;p\_\_Actinobacteria;c\_\_Thermoleophilia;o\_\_Solirubrobacterales | 0 | 0.0% | 0.0% | 0.0% | 0.0% | 0.1% | 0.0% | 0.0% | 0.0% | 0.0% | 0.0% | 0.0% | 0.0% | 0.0% | 0.0% | 0.0% | 0.0% | 0.0% | 0.0% | 0.0% | 0.0% | 0.0% | 0.0% | 0.0% | 0.0% |
|  | k\_\_Bacteria;p\_\_Tenericutes;c\_\_RF3;o\_\_ML615J-28 | 0 | 0.0% | 0.0% | 0.0% | 0.0% | 0.0% | 0.0% | 0.0% | 0.0% | 0.0% | 0.0% | 0.0% | 0.0% | 0.0% | 0.0% | 0.0% | 0.0% | 0.0% | 0.0% | 0.0% | 0.0% | 0.1% | 0.1% | 0.0% | 0.0% |
|  | k\_\_Bacteria;p\_\_Chloroflexi;c\_\_Anaerolineae;o\_\_H39 | 0 | 0.0% | 0.0% | 0.0% | 0.0% | 0.0% | 0.0% | 0.0% | 0.0% | 0.0% | 0.0% | 0.0% | 0.0% | 0.0% | 0.0% | 0.0% | 0.0% | 0.2% | 0.0% | 0.0% | 0.0% | 0.0% | 0.0% | 0.0% | 0.0% |
|  | k\_\_Bacteria;p\_\_Proteobacteria;c\_\_Gammaproteobacteria;o\_\_Alteromonadales | 0 | 0.0% | 0.0% | 0.0% | 0.1% | 0.0% | 0.0% | 0.1% | 0.0% | 0.0% | 0.0% | 0.0% | 0.0% | 0.0% | 0.0% | 0.0% | 0.0% | 0.0% | 0.0% | 0.0% | 0.0% | 0.0% | 0.0% | 0.0% | 0.0% |
|  | k\_\_Bacteria;p\_\_Proteobacteria;c\_\_Betaproteobacteria;o\_\_Unclassified\_Betaproteobacteria | 0 | 0.0% | 0.0% | 0.0% | 0.0% | 0.0% | 0.0% | 0.0% | 0.0% | 0.0% | 0.0% | 0.0% | 0.0% | 0.0% | 0.0% | 0.0% | 0.0% | 0.1% | 0.0% | 0.0% | 0.0% | 0.0% | 0.0% | 0.0% | 0.0% |
|  | k\_\_Bacteria;p\_\_Armatimonadetes;c\_\_[Fimbriimonadia];o\_\_[Fimbriimonadales] | 0 | 0.0% | 0.0% | 0.0% | 0.0% | 0.0% | 0.0% | 0.0% | 0.0% | 0.0% | 0.0% | 0.0% | 0.0% | 0.0% | 0.0% | 0.0% | 0.0% | 0.0% | 0.0% | 0.0% | 0.0% | 0.0% | 0.0% | 0.0% | 0.0% |
|  | k\_\_Bacteria;p\_\_Proteobacteria;c\_\_Gammaproteobacteria;o\_\_Thiotrichales | 0 | 0.0% | 0.0% | 0.0% | 0.0% | 0.0% | 0.0% | 0.0% | 0.0% | 0.0% | 0.0% | 0.0% | 0.0% | 0.0% | 0.0% | 0.0% | 0.0% | 0.1% | 0.0% | 0.0% | 0.0% | 0.0% | 0.0% | 0.0% | 0.0% |
|  | k\_\_Bacteria;p\_\_WPS-2;c\_\_Unclassified\_WPS-2;o\_\_Unclassified\_WPS-2 | 0 | 0.0% | 0.0% | 0.0% | 0.0% | 0.0% | 0.0% | 0.0% | 0.0% | 0.0% | 0.0% | 0.0% | 0.0% | 0.0% | 0.0% | 0.0% | 0.0% | 0.0% | 0.0% | 0.0% | 0.0% | 0.0% | 0.0% | 0.0% | 0.0% |
|  | k\_\_Bacteria;p\_\_Acidobacteria;c\_\_[Chloracidobacteria];o\_\_RB41 | 0 | 0.0% | 0.0% | 0.0% | 0.0% | 0.0% | 0.0% | 0.0% | 0.0% | 0.0% | 0.0% | 0.0% | 0.0% | 0.0% | 0.0% | 0.0% | 0.0% | 0.0% | 0.0% | 0.0% | 0.0% | 0.0% | 0.1% | 0.0% | 0.0% |
|  | k\_\_Bacteria;p\_\_Chloroflexi;c\_\_S085;o\_\_Unclassified\_S085 | 0 | 0.0% | 0.0% | 0.0% | 0.1% | 0.0% | 0.0% | 0.0% | 0.0% | 0.0% | 0.0% | 0.0% | 0.0% | 0.0% | 0.0% | 0.0% | 0.1% | 0.0% | 0.0% | 0.0% | 0.0% | 0.0% | 0.1% | 0.0% | 0.0% |
|  | k\_\_Bacteria;p\_\_OD1;c\_\_ZB2;o\_\_Unclassified\_ZB2 | 0 | 0.0% | 0.0% | 0.0% | 0.0% | 0.0% | 0.0% | 0.0% | 0.0% | 0.0% | 0.0% | 0.0% | 0.0% | 0.0% | 0.0% | 0.0% | 0.0% | 0.0% | 0.0% | 0.0% | 0.0% | 0.0% | 0.0% | 0.0% | 0.0% |
|  | k\_\_Bacteria;p\_\_Proteobacteria;c\_\_Betaproteobacteria;o\_\_Hydrogenophilales | 0 | 0.0% | 0.0% | 0.0% | 0.0% | 0.0% | 0.0% | 0.0% | 0.0% | 0.0% | 0.0% | 0.0% | 0.0% | 0.0% | 0.0% | 0.0% | 0.0% | 0.0% | 0.0% | 0.0% | 0.0% | 0.0% | 0.0% | 0.0% | 0.2% |
|  | k\_\_Bacteria;p\_\_Proteobacteria;c\_\_Deltaproteobacteria;o\_\_GMD14H09 | 0 | 0.0% | 0.0% | 0.0% | 0.0% | 0.0% | 0.0% | 0.0% | 0.0% | 0.0% | 0.0% | 0.0% | 0.0% | 0.0% | 0.0% | 0.0% | 0.0% | 0.0% | 0.0% | 0.1% | 0.0% | 0.0% | 0.0% | 0.0% | 0.1% |
|  | k\_\_Bacteria;p\_\_Chloroflexi;c\_\_Chloroflexi;o\_\_[Roseiflexales] | 0 | 0.0% | 0.0% | 0.0% | 0.1% | 0.0% | 0.0% | 0.0% | 0.0% | 0.0% | 0.0% | 0.0% | 0.0% | 0.0% | 0.0% | 0.0% | 0.0% | 0.0% | 0.0% | 0.0% | 0.0% | 0.0% | 0.0% | 0.0% | 0.0% |
|  | k\_\_Bacteria;p\_\_[Thermi];c\_\_Deinococci;o\_\_Thermales | 0 | 0.0% | 0.0% | 0.0% | 0.0% | 0.0% | 0.0% | 0.0% | 0.0% | 0.0% | 0.1% | 0.0% | 0.0% | 0.0% | 0.0% | 0.0% | 0.0% | 0.0% | 0.0% | 0.0% | 0.0% | 0.0% | 0.0% | 0.0% | 0.0% |
|  | k\_\_Bacteria;p\_\_Armatimonadetes;c\_\_Chthonomonadetes;o\_\_Chthonomonadales | 0 | 0.0% | 0.0% | 0.0% | 0.1% | 0.0% | 0.0% | 0.0% | 0.0% | 0.0% | 0.0% | 0.0% | 0.0% | 0.0% | 0.0% | 0.0% | 0.0% | 0.0% | 0.0% | 0.0% | 0.0% | 0.0% | 0.0% | 0.0% | 0.0% |
|  | k\_\_Bacteria;p\_\_AD3;c\_\_ABS-6;o\_\_Unclassified\_ABS-6 | 0 | 0.0% | 0.0% | 0.0% | 0.0% | 0.0% | 0.0% | 0.0% | 0.0% | 0.0% | 0.0% | 0.0% | 0.0% | 0.0% | 0.0% | 0.0% | 0.0% | 0.1% | 0.0% | 0.0% | 0.0% | 0.0% | 0.0% | 0.0% | 0.0% |
|  | k\_\_Bacteria;p\_\_Acidobacteria;c\_\_Acidobacteria-6;o\_\_CCU21 | 0 | 0.0% | 0.0% | 0.0% | 0.0% | 0.0% | 0.0% | 0.0% | 0.0% | 0.0% | 0.0% | 0.0% | 0.0% | 0.0% | 0.0% | 0.0% | 0.0% | 0.0% | 0.0% | 0.0% | 0.0% | 0.0% | 0.1% | 0.0% | 0.0% |
|  | k\_\_Bacteria;p\_\_Spirochaetes;c\_\_Spirochaetes;o\_\_Sphaerochaetales | 0 | 0.0% | 0.0% | 0.0% | 0.0% | 0.0% | 0.0% | 0.1% | 0.0% | 0.0% | 0.0% | 0.0% | 0.0% | 0.0% | 0.0% | 0.0% | 0.0% | 0.0% | 0.0% | 0.0% | 0.0% | 0.0% | 0.0% | 0.0% | 0.0% |
|  | k\_\_Bacteria;p\_\_Tenericutes;c\_\_CK-1C4-19;o\_\_Unclassified\_CK-1C4-19 | 0 | 0.0% | 0.0% | 0.0% | 0.1% | 0.0% | 0.0% | 0.0% | 0.0% | 0.0% | 0.0% | 0.0% | 0.0% | 0.0% | 0.0% | 0.0% | 0.0% | 0.0% | 0.0% | 0.0% | 0.0% | 0.0% | 0.0% | 0.0% | 0.0% |
|  | k\_\_Bacteria;p\_\_Chloroflexi;c\_\_Thermomicrobia;o\_\_JG30-KF-CM45 | 0 | 0.0% | 0.0% | 0.0% | 0.0% | 0.0% | 0.0% | 0.0% | 0.0% | 0.0% | 0.0% | 0.0% | 0.0% | 0.0% | 0.0% | 0.0% | 0.0% | 0.0% | 0.0% | 0.0% | 0.0% | 0.0% | 0.1% | 0.0% | 0.0% |
|  | k\_\_Bacteria;p\_\_Proteobacteria;c\_\_Alphaproteobacteria;o\_\_BD7-3 | 0 | 0.0% | 0.0% | 0.0% | 0.0% | 0.0% | 0.0% | 0.0% | 0.0% | 0.0% | 0.0% | 0.0% | 0.0% | 0.0% | 0.0% | 0.0% | 0.0% | 0.0% | 0.0% | 0.0% | 0.0% | 0.0% | 0.0% | 0.0% | 0.0% |
|  | k\_\_Bacteria;p\_\_Deferribacteres;c\_\_Deferribacteres;o\_\_Deferribacterales | 0 | 0.0% | 0.0% | 0.0% | 0.0% | 0.0% | 0.0% | 0.0% | 0.0% | 0.0% | 0.0% | 0.0% | 0.0% | 0.0% | 0.0% | 0.0% | 0.0% | 0.0% | 0.0% | 0.0% | 0.0% | 0.0% | 0.0% | 0.0% | 0.0% |
|  | k\_\_Bacteria;p\_\_Proteobacteria;c\_\_Betaproteobacteria;o\_\_Ellin6067 | 0 | 0.0% | 0.0% | 0.0% | 0.0% | 0.0% | 0.0% | 0.0% | 0.0% | 0.0% | 0.0% | 0.0% | 0.0% | 0.0% | 0.0% | 0.0% | 0.0% | 0.0% | 0.0% | 0.0% | 0.0% | 0.0% | 0.1% | 0.0% | 0.0% |
|  | k\_\_Bacteria;p\_\_Gemmatimonadetes;c\_\_Gemmatimonadetes;o\_\_Ellin5290 | 0 | 0.0% | 0.0% | 0.0% | 0.0% | 0.0% | 0.0% | 0.0% | 0.0% | 0.0% | 0.0% | 0.0% | 0.0% | 0.0% | 0.0% | 0.0% | 0.0% | 0.0% | 0.0% | 0.0% | 0.0% | 0.0% | 0.1% | 0.0% | 0.0% |
|  | k\_\_Bacteria;p\_\_Proteobacteria;c\_\_Deltaproteobacteria;o\_\_MBNT15 | 0 | 0.0% | 0.0% | 0.0% | 0.0% | 0.0% | 0.0% | 0.0% | 0.0% | 0.0% | 0.0% | 0.0% | 0.0% | 0.0% | 0.0% | 0.0% | 0.0% | 0.1% | 0.0% | 0.0% | 0.0% | 0.0% | 0.0% | 0.0% | 0.0% |
|  | k\_\_Bacteria;p\_\_Cyanobacteria;c\_\_Chloroplast;o\_\_Stramenopiles | 0 | 0.0% | 0.0% | 0.0% | 0.0% | 0.0% | 0.0% | 0.0% | 0.0% | 0.0% | 0.0% | 0.0% | 0.0% | 0.0% | 0.0% | 0.0% | 0.0% | 0.0% | 0.0% | 0.0% | 0.0% | 0.0% | 0.0% | 0.0% | 0.0% |
|  | k\_\_Bacteria;p\_\_Chloroflexi;c\_\_Anaerolineae;o\_\_CFB-26 | 0 | 0.0% | 0.0% | 0.0% | 0.0% | 0.0% | 0.0% | 0.0% | 0.0% | 0.0% | 0.0% | 0.0% | 0.0% | 0.0% | 0.0% | 0.0% | 0.0% | 0.0% | 0.0% | 0.0% | 0.0% | 0.0% | 0.0% | 0.0% | 0.0% |
|  | k\_\_Bacteria;p\_\_Chloroflexi;c\_\_Anaerolineae;o\_\_GCA004 | 0 | 0.0% | 0.0% | 0.0% | 0.0% | 0.0% | 0.0% | 0.0% | 0.0% | 0.0% | 0.0% | 0.0% | 0.0% | 0.0% | 0.0% | 0.0% | 0.0% | 0.1% | 0.0% | 0.0% | 0.0% | 0.0% | 0.0% | 0.0% | 0.0% |
|  | k\_\_Bacteria;p\_\_Proteobacteria;c\_\_Deltaproteobacteria;o\_\_Desulfobacterales | 0 | 0.0% | 0.0% | 0.0% | 0.0% | 0.0% | 0.0% | 0.0% | 0.0% | 0.0% | 0.0% | 0.0% | 0.0% | 0.0% | 0.0% | 0.0% | 0.0% | 0.1% | 0.0% | 0.0% | 0.0% | 0.0% | 0.0% | 0.0% | 0.0% |
|  | k\_\_Bacteria;p\_\_Proteobacteria;c\_\_Betaproteobacteria;o\_\_IS-44 | 0 | 0.0% | 0.0% | 0.0% | 0.0% | 0.0% | 0.0% | 0.0% | 0.0% | 0.0% | 0.0% | 0.0% | 0.0% | 0.0% | 0.0% | 0.0% | 0.0% | 0.1% | 0.0% | 0.0% | 0.0% | 0.0% | 0.0% | 0.0% | 0.0% |
|  | k\_\_Bacteria;p\_\_Acidobacteria;c\_\_DA052;o\_\_Ellin6513 | 0 | 0.0% | 0.0% | 0.0% | 0.0% | 0.0% | 0.0% | 0.0% | 0.0% | 0.0% | 0.0% | 0.0% | 0.0% | 0.0% | 0.0% | 0.0% | 0.0% | 0.0% | 0.0% | 0.0% | 0.0% | 0.0% | 0.0% | 0.0% | 0.0% |
|  | k\_\_Bacteria;p\_\_Chloroflexi;c\_\_Ellin6529;o\_\_Unclassified\_Ellin6529 | 0 | 0.0% | 0.0% | 0.0% | 0.0% | 0.0% | 0.0% | 0.0% | 0.0% | 0.0% | 0.0% | 0.0% | 0.0% | 0.0% | 0.0% | 0.0% | 0.0% | 0.0% | 0.0% | 0.0% | 0.0% | 0.0% | 0.1% | 0.0% | 0.0% |
|  | k\_\_Bacteria;p\_\_GAL15;c\_\_Unclassified\_GAL15;o\_\_Unclassified\_GAL15 | 0 | 0.0% | 0.0% | 0.0% | 0.0% | 0.0% | 0.0% | 0.0% | 0.0% | 0.0% | 0.0% | 0.0% | 0.0% | 0.0% | 0.0% | 0.0% | 0.0% | 0.1% | 0.0% | 0.0% | 0.0% | 0.0% | 0.0% | 0.0% | 0.0% |
|  | k\_\_Bacteria;p\_\_Lentisphaerae;c\_\_[Lentisphaeria];o\_\_Z20 | 0 | 0.0% | 0.0% | 0.0% | 0.0% | 0.0% | 0.0% | 0.0% | 0.0% | 0.0% | 0.0% | 0.0% | 0.0% | 0.0% | 0.0% | 0.0% | 0.0% | 0.0% | 0.0% | 0.0% | 0.0% | 0.0% | 0.1% | 0.0% | 0.0% |
|  | k\_\_Bacteria;p\_\_Planctomycetes;c\_\_C6;o\_\_MVS-107 | 0 | 0.0% | 0.0% | 0.0% | 0.0% | 0.0% | 0.0% | 0.0% | 0.0% | 0.0% | 0.0% | 0.0% | 0.0% | 0.0% | 0.0% | 0.0% | 0.0% | 0.0% | 0.0% | 0.0% | 0.0% | 0.0% | 0.1% | 0.0% | 0.0% |
|  | k\_\_Bacteria;p\_\_Chlorobi;c\_\_SJA-28;o\_\_Unclassified\_SJA-28 | 0 | 0.0% | 0.0% | 0.0% | 0.0% | 0.0% | 0.0% | 0.0% | 0.0% | 0.0% | 0.0% | 0.0% | 0.0% | 0.0% | 0.0% | 0.0% | 0.0% | 0.0% | 0.0% | 0.0% | 0.0% | 0.0% | 0.0% | 0.0% | 0.0% |
|  | k\_\_Bacteria;p\_\_Chloroflexi;c\_\_TK17;o\_\_Unclassified\_TK17 | 0 | 0.0% | 0.0% | 0.0% | 0.0% | 0.0% | 0.0% | 0.0% | 0.0% | 0.0% | 0.0% | 0.0% | 0.0% | 0.0% | 0.0% | 0.0% | 0.0% | 0.0% | 0.0% | 0.0% | 0.0% | 0.0% | 0.0% | 0.0% | 0.0% |
|  | k\_\_Bacteria;p\_\_Chloroflexi;c\_\_Anaerolineae;o\_\_DRC31 | 0 | 0.0% | 0.0% | 0.0% | 0.0% | 0.0% | 0.0% | 0.0% | 0.0% | 0.0% | 0.0% | 0.0% | 0.0% | 0.0% | 0.0% | 0.0% | 0.0% | 0.1% | 0.0% | 0.0% | 0.0% | 0.0% | 0.0% | 0.0% | 0.0% |
|  | k\_\_Bacteria;p\_\_Elusimicrobia;c\_\_Elusimicrobia;o\_\_Elusimicrobiales | 0 | 0.0% | 0.0% | 0.0% | 0.0% | 0.0% | 0.0% | 0.0% | 0.0% | 0.0% | 0.0% | 0.0% | 0.0% | 0.0% | 0.0% | 0.0% | 0.0% | 0.0% | 0.0% | 0.0% | 0.0% | 0.0% | 0.0% | 0.0% | 0.0% |
|  | k\_\_Bacteria;p\_\_Firmicutes;c\_\_Clostridia;o\_\_Thermoanaerobacterales | 0 | 0.0% | 0.0% | 0.0% | 0.0% | 0.0% | 0.0% | 0.0% | 0.0% | 0.0% | 0.0% | 0.0% | 0.0% | 0.0% | 0.0% | 0.0% | 0.0% | 0.0% | 0.0% | 0.0% | 0.0% | 0.0% | 0.0% | 0.0% | 0.0% |
|  | k\_\_Bacteria;p\_\_Chloroflexi;c\_\_Ktedonobacteria;o\_\_JG30-KF-AS9 | 0 | 0.0% | 0.0% | 0.0% | 0.0% | 0.0% | 0.0% | 0.0% | 0.0% | 0.0% | 0.0% | 0.0% | 0.0% | 0.0% | 0.0% | 0.1% | 0.0% | 0.0% | 0.0% | 0.0% | 0.0% | 0.0% | 0.0% | 0.0% | 0.0% |
|  | k\_\_Bacteria;p\_\_Gemmatimonadetes;c\_\_Gemm-5;o\_\_Unclassified\_Gemm-5 | 0 | 0.0% | 0.0% | 0.0% | 0.0% | 0.0% | 0.0% | 0.0% | 0.0% | 0.0% | 0.0% | 0.0% | 0.0% | 0.0% | 0.0% | 0.0% | 0.0% | 0.0% | 0.0% | 0.0% | 0.0% | 0.0% | 0.0% | 0.0% | 0.0% |
|  | k\_\_Bacteria;p\_\_Proteobacteria;c\_\_Gammaproteobacteria;o\_\_Vibrionales | 0 | 0.0% | 0.0% | 0.0% | 0.1% | 0.0% | 0.0% | 0.0% | 0.0% | 0.0% | 0.0% | 0.0% | 0.0% | 0.0% | 0.0% | 0.0% | 0.0% | 0.0% | 0.0% | 0.0% | 0.0% | 0.0% | 0.0% | 0.0% | 0.0% |
|  | k\_\_Bacteria;p\_\_Proteobacteria;c\_\_Deltaproteobacteria;o\_\_Desulfuromonadales | 0 | 0.0% | 0.0% | 0.0% | 0.0% | 0.0% | 0.0% | 0.0% | 0.0% | 0.0% | 0.0% | 0.0% | 0.0% | 0.0% | 0.0% | 0.0% | 0.0% | 0.0% | 0.0% | 0.0% | 0.0% | 0.0% | 0.0% | 0.0% | 0.0% |
|  | k\_\_Bacteria;p\_\_Proteobacteria;c\_\_Alphaproteobacteria;o\_\_RF32 | 0 | 0.0% | 0.0% | 0.0% | 0.0% | 0.0% | 0.0% | 0.0% | 0.0% | 0.0% | 0.0% | 0.0% | 0.0% | 0.0% | 0.0% | 0.0% | 0.0% | 0.0% | 0.0% | 0.0% | 0.0% | 0.0% | 0.0% | 0.0% | 0.0% |
|  | k\_\_Bacteria;p\_\_Verrucomicrobia;c\_\_Opitutae;o\_\_[Cerasicoccales] | 0 | 0.0% | 0.0% | 0.0% | 0.0% | 0.0% | 0.0% | 0.0% | 0.0% | 0.0% | 0.0% | 0.0% | 0.0% | 0.0% | 0.0% | 0.0% | 0.0% | 0.0% | 0.0% | 0.0% | 0.0% | 0.0% | 0.0% | 0.0% | 0.0% |
|  | k\_\_Bacteria;p\_\_Chloroflexi;c\_\_Anaerolineae;o\_\_Caldilineales | 0 | 0.0% | 0.0% | 0.0% | 0.0% | 0.0% | 0.0% | 0.0% | 0.0% | 0.0% | 0.0% | 0.0% | 0.0% | 0.0% | 0.0% | 0.0% | 0.1% | 0.0% | 0.0% | 0.0% | 0.0% | 0.0% | 0.0% | 0.0% | 0.0% |
|  | k\_\_Bacteria;p\_\_Proteobacteria;c\_\_Gammaproteobacteria;o\_\_Unclassified\_Gammaproteobacteria | 0 | 0.0% | 0.0% | 0.0% | 0.0% | 0.0% | 0.0% | 0.0% | 0.0% | 0.0% | 0.0% | 0.0% | 0.0% | 0.0% | 0.0% | 0.0% | 0.0% | 0.0% | 0.0% | 0.0% | 0.0% | 0.0% | 0.0% | 0.0% | 0.0% |
|  | k\_\_Bacteria;p\_\_Proteobacteria;c\_\_Betaproteobacteria;o\_\_Methylophilales | 0 | 0.0% | 0.0% | 0.0% | 0.0% | 0.0% | 0.0% | 0.0% | 0.0% | 0.0% | 0.0% | 0.0% | 0.0% | 0.0% | 0.0% | 0.0% | 0.0% | 0.0% | 0.0% | 0.0% | 0.0% | 0.0% | 0.0% | 0.0% | 0.0% |
|  | k\_\_Bacteria;p\_\_TM7;c\_\_TM7-1;o\_\_Unclassified\_TM7-1 | 0 | 0.0% | 0.0% | 0.0% | 0.0% | 0.0% | 0.0% | 0.0% | 0.0% | 0.0% | 0.0% | 0.0% | 0.0% | 0.0% | 0.0% | 0.0% | 0.0% | 0.0% | 0.0% | 0.0% | 0.0% | 0.0% | 0.0% | 0.0% | 0.0% |
|  | k\_\_Bacteria;p\_\_Proteobacteria;c\_\_Gammaproteobacteria;o\_\_Oceanospirillales | 0 | 0.0% | 0.0% | 0.0% | 0.0% | 0.0% | 0.0% | 0.0% | 0.0% | 0.0% | 0.0% | 0.0% | 0.0% | 0.0% | 0.0% | 0.0% | 0.0% | 0.0% | 0.0% | 0.0% | 0.0% | 0.0% | 0.0% | 0.0% | 0.0% |
|  | k\_\_Bacteria;p\_\_Proteobacteria;c\_\_Gammaproteobacteria;o\_\_[Marinicellales] | 0 | 0.0% | 0.0% | 0.0% | 0.0% | 0.0% | 0.0% | 0.0% | 0.0% | 0.0% | 0.0% | 0.0% | 0.0% | 0.0% | 0.0% | 0.0% | 0.0% | 0.0% | 0.0% | 0.0% | 0.0% | 0.0% | 0.0% | 0.0% | 0.0% |
|  | k\_\_Bacteria;p\_\_Cyanobacteria;c\_\_ML635J-21;o\_\_Unclassified\_ML635J-21 | 0 | 0.0% | 0.0% | 0.0% | 0.0% | 0.0% | 0.0% | 0.0% | 0.0% | 0.0% | 0.0% | 0.0% | 0.0% | 0.0% | 0.0% | 0.0% | 0.0% | 0.0% | 0.0% | 0.0% | 0.0% | 0.0% | 0.0% | 0.0% | 0.0% |
|  | k\_\_Bacteria;p\_\_Planctomycetes;c\_\_OM190;o\_\_agg27 | 0 | 0.0% | 0.0% | 0.0% | 0.0% | 0.0% | 0.0% | 0.0% | 0.0% | 0.0% | 0.0% | 0.0% | 0.0% | 0.0% | 0.0% | 0.0% | 0.0% | 0.0% | 0.0% | 0.0% | 0.0% | 0.0% | 0.0% | 0.0% | 0.0% |
|  | k\_\_Bacteria;p\_\_Chloroflexi;c\_\_Anaerolineae;o\_\_S0208 | 0 | 0.0% | 0.0% | 0.0% | 0.0% | 0.0% | 0.0% | 0.0% | 0.0% | 0.0% | 0.0% | 0.0% | 0.0% | 0.0% | 0.0% | 0.0% | 0.0% | 0.0% | 0.0% | 0.0% | 0.0% | 0.0% | 0.0% | 0.0% | 0.0% |
|  | k\_\_Bacteria;p\_\_Fibrobacteres;c\_\_Fibrobacteria;o\_\_Fibrobacterales | 0 | 0.0% | 0.0% | 0.0% | 0.0% | 0.0% | 0.0% | 0.0% | 0.0% | 0.0% | 0.0% | 0.0% | 0.0% | 0.0% | 0.0% | 0.0% | 0.0% | 0.0% | 0.0% | 0.0% | 0.0% | 0.0% | 0.0% | 0.0% | 0.0% |
|  | k\_\_Bacteria;p\_\_Cyanobacteria;c\_\_Oscillatoriophycideae;o\_\_Chroococcales | 0 | 0.0% | 0.0% | 0.0% | 0.0% | 0.0% | 0.0% | 0.0% | 0.0% | 0.0% | 0.0% | 0.0% | 0.0% | 0.0% | 0.0% | 0.0% | 0.0% | 0.0% | 0.0% | 0.0% | 0.0% | 0.0% | 0.0% | 0.0% | 0.0% |
|  | k\_\_Bacteria;p\_\_Acidobacteria;c\_\_RB25;o\_\_Unclassified\_RB25 | 0 | 0.0% | 0.0% | 0.0% | 0.0% | 0.0% | 0.0% | 0.0% | 0.0% | 0.0% | 0.0% | 0.0% | 0.0% | 0.0% | 0.0% | 0.0% | 0.0% | 0.0% | 0.0% | 0.0% | 0.0% | 0.0% | 0.0% | 0.0% | 0.0% |
|  | k\_\_Bacteria;p\_\_Chloroflexi;c\_\_Chloroflexi;o\_\_AKIW781 | 0 | 0.0% | 0.0% | 0.0% | 0.0% | 0.0% | 0.0% | 0.0% | 0.0% | 0.0% | 0.0% | 0.0% | 0.0% | 0.0% | 0.0% | 0.0% | 0.0% | 0.0% | 0.0% | 0.0% | 0.0% | 0.0% | 0.0% | 0.0% | 0.0% |
|  | k\_\_Bacteria;p\_\_Acidobacteria;c\_\_[Chloracidobacteria];o\_\_DS-100 | 0 | 0.0% | 0.0% | 0.0% | 0.0% | 0.0% | 0.0% | 0.0% | 0.0% | 0.0% | 0.0% | 0.0% | 0.0% | 0.0% | 0.0% | 0.0% | 0.0% | 0.0% | 0.0% | 0.0% | 0.0% | 0.0% | 0.0% | 0.0% | 0.0% |
|  | k\_\_Bacteria;p\_\_Chloroflexi;c\_\_Gitt-GS-136;o\_\_Unclassified\_Gitt-GS-136 | 0 | 0.0% | 0.0% | 0.0% | 0.0% | 0.0% | 0.0% | 0.0% | 0.0% | 0.0% | 0.0% | 0.0% | 0.0% | 0.0% | 0.0% | 0.0% | 0.0% | 0.0% | 0.0% | 0.0% | 0.0% | 0.0% | 0.0% | 0.0% | 0.0% |
|  | k\_\_Bacteria;p\_\_Chloroflexi;c\_\_TK10;o\_\_B07\_WMSP1 | 0 | 0.0% | 0.0% | 0.0% | 0.0% | 0.0% | 0.0% | 0.0% | 0.0% | 0.0% | 0.0% | 0.0% | 0.0% | 0.0% | 0.0% | 0.0% | 0.0% | 0.0% | 0.0% | 0.0% | 0.0% | 0.0% | 0.0% | 0.0% | 0.0% |
|  | k\_\_Bacteria;p\_\_Proteobacteria;c\_\_Gammaproteobacteria;o\_\_HOC36 | 0 | 0.0% | 0.0% | 0.0% | 0.0% | 0.0% | 0.0% | 0.0% | 0.0% | 0.0% | 0.0% | 0.0% | 0.0% | 0.0% | 0.0% | 0.0% | 0.0% | 0.0% | 0.0% | 0.0% | 0.0% | 0.0% | 0.0% | 0.0% | 0.0% |
|  | k\_\_Bacteria;p\_\_Tenericutes;c\_\_Mollicutes;o\_\_Unclassified\_Mollicutes | 0 | 0.0% | 0.0% | 0.0% | 0.0% | 0.0% | 0.0% | 0.0% | 0.0% | 0.0% | 0.0% | 0.0% | 0.0% | 0.0% | 0.0% | 0.0% | 0.0% | 0.0% | 0.0% | 0.0% | 0.0% | 0.0% | 0.0% | 0.0% | 0.0% |
|  | k\_\_Bacteria;p\_\_Proteobacteria;c\_\_Deltaproteobacteria;o\_\_BPC076 | 0 | 0.0% | 0.0% | 0.0% | 0.0% | 0.0% | 0.0% | 0.0% | 0.0% | 0.0% | 0.0% | 0.0% | 0.0% | 0.0% | 0.0% | 0.0% | 0.0% | 0.0% | 0.0% | 0.0% | 0.0% | 0.0% | 0.0% | 0.0% | 0.0% |
|  | k\_\_Bacteria;p\_\_Acidobacteria;c\_\_Sva0725;o\_\_Sva0725 | 0 | 0.0% | 0.0% | 0.0% | 0.0% | 0.0% | 0.0% | 0.0% | 0.0% | 0.0% | 0.0% | 0.0% | 0.0% | 0.0% | 0.0% | 0.0% | 0.0% | 0.0% | 0.0% | 0.0% | 0.0% | 0.0% | 0.0% | 0.0% | 0.0% |
|  | k\_\_Bacteria;p\_\_Gemmatimonadetes;c\_\_Gemmatimonadetes;o\_\_C114 | 0 | 0.0% | 0.0% | 0.0% | 0.0% | 0.0% | 0.0% | 0.0% | 0.0% | 0.0% | 0.0% | 0.0% | 0.0% | 0.0% | 0.0% | 0.0% | 0.0% | 0.0% | 0.0% | 0.0% | 0.0% | 0.0% | 0.0% | 0.0% | 0.0% |
|  | k\_\_Bacteria;p\_\_Acidobacteria;c\_\_BPC102;o\_\_MVS-40 | 0 | 0.0% | 0.0% | 0.0% | 0.0% | 0.0% | 0.0% | 0.0% | 0.0% | 0.0% | 0.0% | 0.0% | 0.0% | 0.0% | 0.0% | 0.0% | 0.0% | 0.0% | 0.0% | 0.0% | 0.0% | 0.0% | 0.0% | 0.0% | 0.0% |
|  | k\_\_Bacteria;p\_\_Fibrobacteres;c\_\_Fibrobacteria;o\_\_258ds10 | 0 | 0.0% | 0.0% | 0.0% | 0.0% | 0.0% | 0.0% | 0.0% | 0.0% | 0.0% | 0.0% | 0.0% | 0.0% | 0.0% | 0.0% | 0.0% | 0.0% | 0.0% | 0.0% | 0.0% | 0.0% | 0.0% | 0.0% | 0.0% | 0.0% |
|  | k\_\_Bacteria;p\_\_Chloroflexi;c\_\_Anaerolineae;o\_\_Anaerolineales | 0 | 0.0% | 0.0% | 0.0% | 0.0% | 0.0% | 0.0% | 0.0% | 0.0% | 0.0% | 0.0% | 0.0% | 0.0% | 0.0% | 0.0% | 0.0% | 0.0% | 0.0% | 0.0% | 0.0% | 0.0% | 0.0% | 0.0% | 0.0% | 0.0% |
|  | k\_\_Bacteria;p\_\_Acidobacteria;c\_\_AT-s54;o\_\_Unclassified\_AT-s54 | 0 | 0.0% | 0.0% | 0.0% | 0.0% | 0.0% | 0.0% | 0.0% | 0.0% | 0.0% | 0.0% | 0.0% | 0.0% | 0.0% | 0.0% | 0.0% | 0.0% | 0.0% | 0.0% | 0.0% | 0.0% | 0.0% | 0.0% | 0.0% | 0.0% |
|  | k\_\_Bacteria;p\_\_OD1;c\_\_ABY1;o\_\_Unclassified\_ABY1 | 0 | 0.0% | 0.0% | 0.0% | 0.0% | 0.0% | 0.0% | 0.0% | 0.0% | 0.0% | 0.0% | 0.0% | 0.0% | 0.0% | 0.0% | 0.0% | 0.0% | 0.0% | 0.0% | 0.0% | 0.0% | 0.0% | 0.0% | 0.0% | 0.0% |
|  | k\_\_Bacteria;p\_\_Proteobacteria;c\_\_Deltaproteobacteria;o\_\_Spirobacillales | 0 | 0.0% | 0.0% | 0.0% | 0.0% | 0.0% | 0.0% | 0.0% | 0.0% | 0.0% | 0.0% | 0.0% | 0.0% | 0.0% | 0.0% | 0.0% | 0.0% | 0.0% | 0.0% | 0.0% | 0.0% | 0.0% | 0.0% | 0.0% | 0.0% |
|  | k\_\_Bacteria;p\_\_Armatimonadetes;c\_\_0319-6E2;o\_\_Unclassified\_0319-6E2 | 0 | 0.0% | 0.0% | 0.0% | 0.0% | 0.0% | 0.0% | 0.0% | 0.0% | 0.0% | 0.0% | 0.0% | 0.0% | 0.0% | 0.0% | 0.0% | 0.0% | 0.0% | 0.0% | 0.0% | 0.0% | 0.0% | 0.0% | 0.0% | 0.0% |
|  | k\_\_Bacteria;p\_\_Gemmatimonadetes;c\_\_Gemmatimonadetes;o\_\_KD8-87 | 0 | 0.0% | 0.0% | 0.0% | 0.0% | 0.0% | 0.0% | 0.0% | 0.0% | 0.0% | 0.0% | 0.0% | 0.0% | 0.0% | 0.0% | 0.0% | 0.0% | 0.0% | 0.0% | 0.0% | 0.0% | 0.0% | 0.0% | 0.0% | 0.0% |
|  | k\_\_Bacteria;p\_\_Planctomycetes;c\_\_OM190;o\_\_CL500-15 | 0 | 0.0% | 0.0% | 0.0% | 0.0% | 0.0% | 0.0% | 0.0% | 0.0% | 0.0% | 0.0% | 0.0% | 0.0% | 0.0% | 0.0% | 0.0% | 0.0% | 0.0% | 0.0% | 0.0% | 0.0% | 0.0% | 0.0% | 0.0% | 0.0% |

|  |  |
| --- | --- |
|  | |
| Taxonomy Summary. Current Level: Family | |
| View Figure (.pdf)  View Legend (.pdf) |  |
|  |


|  |
| --- |
| View Table (.txt) |

|  |  |  |  |  |  |  |  |  |  |  |  |  |  |  |  |  |  |  |  |  |  |  |  |  |  |  |
| --- | --- | --- | --- | --- | --- | --- | --- | --- | --- | --- | --- | --- | --- | --- | --- | --- | --- | --- | --- | --- | --- | --- | --- | --- | --- | --- |
|  | | Total | | A01 | A02 | A03 | A04 | A05 | A06 | A07 | A08 | A09 | A10 | A11 | A12 | A13 | B01 | B02 | B03 | B04 | B05 | B06 | B07 | B08 | B09 | B10 |
| Legend | Taxonomy | count | % | % | % | % | % | % | % | % | % | % | % | % | % | % | % | % | % | % | % | % | % | % | % | % |
|  | k\_\_Bacteria;p\_\_Firmicutes;c\_\_Clostridia;o\_\_Clostridiales;f\_\_[Tissierellaceae] | 3 | 13.4% | 16.5% | 30.7% | 8.9% | 2.1% | 35.3% | 4.1% | 25.1% | 11.3% | 4.6% | 7.5% | 9.4% | 6.0% | 14.6% | 22.2% | 0.9% | 4.7% | 4.5% | 5.9% | 55.0% | 10.7% | 4.2% | 21.2% | 2.3% |
|  | k\_\_Bacteria;p\_\_Firmicutes;c\_\_Bacilli;o\_\_Lactobacillales;f\_\_Aerococcaceae | 2 | 10.5% | 15.4% | 11.0% | 15.9% | 18.3% | 20.9% | 16.5% | 9.6% | 16.7% | 11.6% | 7.5% | 17.4% | 20.7% | 9.0% | 9.8% | 4.8% | 1.5% | 2.7% | 4.5% | 15.7% | 3.6% | 1.1% | 3.2% | 4.3% |
|  | k\_\_Bacteria;p\_\_Fusobacteria;c\_\_Fusobacteriia;o\_\_Fusobacteriales;f\_\_Leptotrichiaceae | 2 | 9.7% | 21.1% | 8.0% | 3.1% | 3.3% | 0.0% | 14.4% | 3.1% | 14.1% | 7.1% | 22.1% | 4.9% | 13.3% | 10.1% | 4.2% | 6.2% | 1.6% | 12.1% | 17.3% | 0.0% | 19.9% | 5.0% | 1.7% | 31.0% |
|  | k\_\_Bacteria;p\_\_Proteobacteria;c\_\_Epsilonproteobacteria;o\_\_Campylobacterales;f\_\_Campylobacteraceae | 2 | 9.3% | 6.8% | 6.6% | 1.6% | 3.8% | 0.0% | 10.6% | 10.9% | 15.1% | 17.9% | 26.8% | 10.7% | 7.5% | 17.2% | 2.6% | 4.4% | 3.2% | 6.3% | 9.0% | 0.1% | 16.1% | 3.1% | 0.5% | 34.1% |
|  | k\_\_Bacteria;p\_\_Fusobacteria;c\_\_Fusobacteriia;o\_\_Fusobacteriales;f\_\_Fusobacteriaceae | 2 | 6.7% | 18.9% | 12.4% | 2.9% | 2.2% | 0.0% | 14.1% | 4.3% | 3.7% | 0.5% | 6.3% | 7.8% | 5.1% | 6.7% | 2.8% | 6.5% | 2.7% | 20.3% | 8.2% | 0.1% | 11.6% | 4.9% | 5.7% | 6.6% |
|  | k\_\_Bacteria;p\_\_Proteobacteria;c\_\_Alphaproteobacteria;o\_\_Rhizobiales;f\_\_Brucellaceae | 2 | 6.5% | 2.8% | 0.5% | 16.9% | 18.5% | 0.8% | 5.4% | 2.8% | 3.2% | 10.8% | 0.9% | 6.7% | 4.6% | 1.0% | 9.4% | 19.0% | 14.8% | 11.4% | 6.1% | 3.5% | 1.0% | 5.3% | 4.1% | 1.0% |
|  | k\_\_Bacteria;p\_\_Bacteroidetes;c\_\_Bacteroidia;o\_\_Bacteroidales;f\_\_Porphyromonadaceae | 1 | 4.5% | 8.4% | 1.5% | 1.0% | 0.6% | 1.6% | 8.3% | 6.7% | 1.9% | 0.1% | 6.1% | 5.7% | 0.9% | 10.2% | 6.6% | 0.7% | 1.5% | 4.5% | 11.4% | 12.0% | 5.3% | 2.9% | 2.8% | 2.0% |
|  | k\_\_Bacteria;p\_\_Firmicutes;c\_\_Bacilli;o\_\_Lactobacillales;f\_\_Carnobacteriaceae | 1 | 4.0% | 0.1% | 8.6% | 1.7% | 2.8% | 26.7% | 0.3% | 8.0% | 9.2% | 7.2% | 2.7% | 1.8% | 11.4% | 8.1% | 2.0% | 0.2% | 0.1% | 0.5% | 0.4% | 0.0% | 0.0% | 0.0% | 0.0% | 0.0% |
|  | k\_\_Bacteria;p\_\_Proteobacteria;c\_\_Betaproteobacteria;o\_\_Burkholderiales;f\_\_Alcaligenaceae | 1 | 2.8% | 1.2% | 0.2% | 6.1% | 4.9% | 0.3% | 2.8% | 1.1% | 1.5% | 9.1% | 0.4% | 2.5% | 1.5% | 0.5% | 4.3% | 7.0% | 7.5% | 4.1% | 3.1% | 1.2% | 0.4% | 2.5% | 1.9% | 0.6% |
|  | k\_\_Bacteria;p\_\_Actinobacteria;c\_\_Actinobacteria;o\_\_Actinomycetales;f\_\_Corynebacteriaceae | 1 | 2.7% | 1.2% | 4.9% | 0.8% | 3.2% | 2.7% | 0.6% | 7.3% | 9.1% | 3.9% | 2.8% | 3.2% | 8.7% | 4.7% | 0.6% | 0.2% | 0.4% | 1.1% | 0.3% | 2.1% | 2.1% | 0.3% | 0.9% | 0.7% |
|  | k\_\_Bacteria;p\_\_Proteobacteria;c\_\_Gammaproteobacteria;o\_\_Xanthomonadales;f\_\_Xanthomonadaceae | 0 | 2.0% | 0.7% | 0.1% | 3.9% | 2.4% | 0.4% | 1.4% | 0.5% | 0.6% | 2.4% | 0.3% | 1.3% | 0.9% | 0.2% | 2.5% | 9.1% | 6.5% | 5.5% | 1.7% | 1.0% | 0.5% | 1.7% | 1.4% | 0.3% |
|  | k\_\_Bacteria;p\_\_Bacteroidetes;c\_\_[Saprospirae];o\_\_[Saprospirales];f\_\_Chitinophagaceae | 0 | 1.9% | 0.7% | 0.1% | 5.0% | 4.9% | 0.3% | 1.7% | 0.9% | 0.6% | 2.0% | 0.4% | 1.7% | 1.0% | 0.3% | 3.3% | 8.7% | 3.9% | 3.5% | 1.6% | 0.8% | 0.3% | 1.4% | 1.1% | 0.3% |
|  | k\_\_Bacteria;p\_\_Proteobacteria;c\_\_Gammaproteobacteria;o\_\_Pseudomonadales;f\_\_Pseudomonadaceae | 0 | 1.6% | 0.0% | 0.0% | 0.7% | 0.4% | 0.0% | 0.5% | 1.0% | 0.6% | 6.9% | 3.2% | 6.6% | 4.3% | 1.5% | 0.3% | 0.8% | 2.4% | 1.7% | 0.2% | 0.2% | 1.9% | 1.6% | 0.2% | 1.3% |
|  | k\_\_Bacteria;p\_\_Proteobacteria;c\_\_Gammaproteobacteria;o\_\_Pseudomonadales;f\_\_Moraxellaceae | 0 | 1.6% | 0.3% | 0.0% | 3.9% | 1.7% | 2.7% | 1.1% | 0.5% | 0.4% | 3.6% | 0.4% | 1.1% | 0.3% | 0.1% | 3.2% | 1.1% | 1.3% | 0.9% | 1.5% | 0.4% | 0.8% | 1.8% | 8.2% | 0.7% |
|  | No blast hit;Other;Other;Other;Other | 0 | 1.6% | 0.1% | 8.5% | 0.3% | 0.5% | 0.5% | 0.6% | 0.0% | 0.9% | 0.0% | 0.0% | 0.0% | 0.6% | 6.9% | 1.0% | 1.4% | 0.6% | 4.2% | 0.8% | 0.0% | 8.9% | 0.1% | 0.2% | 0.0% |
|  | k\_\_Bacteria;p\_\_Actinobacteria;c\_\_Actinobacteria;o\_\_Actinomycetales;f\_\_Actinomycetaceae | 0 | 1.5% | 0.2% | 2.6% | 0.9% | 0.3% | 1.3% | 1.9% | 4.3% | 0.9% | 0.9% | 2.4% | 2.0% | 0.6% | 1.8% | 0.2% | 0.6% | 0.6% | 0.8% | 4.9% | 1.1% | 1.1% | 0.9% | 1.8% | 3.5% |
|  | k\_\_Bacteria;p\_\_Firmicutes;c\_\_Clostridia;o\_\_Clostridiales;f\_\_Clostridiaceae | 0 | 1.3% | 0.0% | 1.4% | 0.7% | 2.4% | 0.4% | 3.0% | 3.4% | 1.0% | 0.4% | 0.7% | 0.5% | 0.6% | 1.5% | 0.4% | 0.7% | 0.4% | 0.5% | 3.7% | 0.0% | 1.1% | 0.4% | 3.0% | 2.6% |
|  | k\_\_Bacteria;p\_\_Proteobacteria;c\_\_Betaproteobacteria;o\_\_Burkholderiales;f\_\_Comamonadaceae | 0 | 1.2% | 0.2% | 0.0% | 2.3% | 1.7% | 0.1% | 0.9% | 0.8% | 0.5% | 1.6% | 0.3% | 2.2% | 1.5% | 0.4% | 2.2% | 2.4% | 2.6% | 1.3% | 0.6% | 0.6% | 0.4% | 3.7% | 1.4% | 0.7% |
|  | k\_\_Bacteria;p\_\_Firmicutes;c\_\_Clostridia;o\_\_Clostridiales;f\_\_Ruminococcaceae | 0 | 1.2% | 0.0% | 0.0% | 0.7% | 0.4% | 0.0% | 0.5% | 0.2% | 1.0% | 0.1% | 0.5% | 1.1% | 1.2% | 0.0% | 0.8% | 0.3% | 2.5% | 0.2% | 0.4% | 0.0% | 2.4% | 11.4% | 2.8% | 0.0% |
|  | k\_\_Bacteria;p\_\_Firmicutes;c\_\_Clostridia;o\_\_Clostridiales;f\_\_Unclassified\_Clostridiales | 0 | 1.0% | 0.2% | 0.6% | 0.7% | 1.5% | 0.0% | 1.2% | 0.3% | 1.2% | 0.1% | 0.8% | 0.6% | 0.6% | 0.0% | 0.8% | 0.6% | 3.1% | 0.0% | 2.4% | 0.0% | 2.2% | 3.9% | 0.5% | 1.2% |
|  | k\_\_Bacteria;p\_\_Proteobacteria;c\_\_Alphaproteobacteria;o\_\_Sphingomonadales;f\_\_Sphingomonadaceae | 0 | 1.0% | 0.3% | 0.1% | 2.1% | 1.7% | 0.1% | 0.8% | 0.3% | 0.4% | 0.8% | 0.2% | 1.7% | 0.7% | 0.2% | 2.2% | 2.8% | 2.4% | 1.4% | 0.7% | 0.5% | 0.2% | 1.0% | 0.8% | 0.2% |
|  | k\_\_Bacteria;p\_\_Firmicutes;c\_\_Clostridia;o\_\_Clostridiales;f\_\_[Acidaminobacteraceae] | 0 | 0.9% | 0.0% | 0.7% | 1.7% | 1.8% | 0.0% | 1.0% | 5.4% | 0.0% | 0.1% | 3.9% | 0.4% | 0.0% | 1.8% | 0.0% | 0.6% | 1.5% | 0.7% | 1.5% | 0.0% | 0.1% | 0.0% | 0.0% | 0.0% |
|  | k\_\_Bacteria;p\_\_Proteobacteria;c\_\_Gammaproteobacteria;o\_\_Enterobacteriales;f\_\_Enterobacteriaceae | 0 | 0.9% | 0.2% | 0.0% | 0.5% | 1.5% | 0.0% | 0.0% | 0.1% | 2.1% | 0.1% | 0.0% | 0.0% | 1.8% | 0.0% | 3.6% | 0.2% | 1.8% | 0.0% | 0.2% | 0.5% | 0.1% | 6.2% | 1.7% | 0.0% |
|  | k\_\_Bacteria;p\_\_Proteobacteria;c\_\_Alphaproteobacteria;o\_\_Rhizobiales;f\_\_Methylobacteriaceae | 0 | 0.8% | 0.3% | 0.0% | 1.8% | 1.6% | 1.7% | 0.8% | 0.4% | 0.4% | 1.1% | 0.1% | 1.0% | 0.6% | 0.1% | 0.9% | 1.8% | 2.1% | 0.7% | 0.4% | 0.3% | 0.1% | 0.7% | 0.4% | 0.1% |
|  | k\_\_Bacteria;p\_\_Actinobacteria;c\_\_Actinobacteria;o\_\_Actinomycetales;f\_\_Micrococcaceae | 0 | 0.7% | 0.1% | 0.3% | 0.6% | 4.7% | 2.3% | 0.5% | 0.0% | 0.0% | 0.1% | 0.0% | 0.1% | 0.0% | 0.0% | 0.1% | 0.4% | 0.2% | 0.1% | 0.4% | 0.0% | 0.0% | 0.2% | 3.6% | 3.5% |
|  | k\_\_Bacteria;p\_\_Firmicutes;c\_\_Clostridia;o\_\_Clostridiales;f\_\_Lachnospiraceae | 0 | 0.7% | 0.0% | 0.0% | 0.6% | 0.3% | 0.0% | 0.1% | 0.1% | 0.3% | 0.0% | 0.2% | 0.4% | 0.2% | 0.0% | 0.9% | 0.4% | 3.4% | 0.1% | 0.1% | 0.1% | 0.8% | 2.0% | 6.6% | 0.0% |
|  | k\_\_Bacteria;p\_\_Proteobacteria;c\_\_Alphaproteobacteria;o\_\_Caulobacterales;f\_\_Caulobacteraceae | 0 | 0.6% | 0.3% | 0.1% | 1.5% | 1.2% | 0.1% | 0.5% | 0.4% | 0.3% | 0.6% | 0.1% | 0.8% | 0.5% | 0.1% | 1.1% | 2.0% | 1.6% | 1.1% | 0.5% | 0.3% | 0.1% | 0.7% | 0.6% | 0.1% |
|  | k\_\_Bacteria;p\_\_Proteobacteria;c\_\_Betaproteobacteria;o\_\_Burkholderiales;f\_\_Oxalobacteraceae | 0 | 0.6% | 0.1% | 0.0% | 1.5% | 0.6% | 0.4% | 0.5% | 0.2% | 0.2% | 0.4% | 0.1% | 0.7% | 0.5% | 0.1% | 1.2% | 1.5% | 1.8% | 0.8% | 0.5% | 0.3% | 0.0% | 0.8% | 0.5% | 0.2% |
|  | k\_\_Bacteria;p\_\_Actinobacteria;c\_\_Actinobacteria;o\_\_Actinomycetales;f\_\_Pseudonocardiaceae | 0 | 0.5% | 0.2% | 0.0% | 0.6% | 0.9% | 0.1% | 0.3% | 0.2% | 0.3% | 1.1% | 0.1% | 0.5% | 0.3% | 0.1% | 0.7% | 1.1% | 1.9% | 0.8% | 0.4% | 0.3% | 0.1% | 0.5% | 0.3% | 0.1% |
|  | k\_\_Bacteria;p\_\_Cyanobacteria;c\_\_4C0d-2;o\_\_MLE1-12;f\_\_Unclassified\_MLE1-12 | 0 | 0.5% | 0.1% | 0.0% | 0.9% | 1.0% | 0.1% | 0.5% | 0.2% | 0.2% | 0.4% | 0.1% | 0.4% | 0.3% | 0.1% | 0.7% | 1.2% | 1.9% | 0.7% | 0.5% | 0.3% | 0.1% | 0.4% | 0.2% | 0.1% |
|  | k\_\_Bacteria;p\_\_Proteobacteria;c\_\_Betaproteobacteria;o\_\_Rhodocyclales;f\_\_Rhodocyclaceae | 0 | 0.4% | 0.2% | 0.0% | 0.8% | 0.6% | 0.0% | 0.3% | 0.2% | 0.1% | 0.2% | 0.0% | 0.4% | 0.2% | 0.0% | 0.6% | 1.3% | 1.5% | 0.5% | 0.4% | 0.2% | 0.1% | 0.6% | 0.3% | 0.1% |
|  | k\_\_Bacteria;p\_\_Actinobacteria;c\_\_Actinobacteria;o\_\_Actinomycetales;f\_\_Microbacteriaceae | 0 | 0.3% | 0.1% | 0.0% | 0.3% | 0.6% | 0.1% | 0.2% | 0.2% | 0.2% | 0.8% | 0.1% | 0.4% | 0.2% | 0.0% | 0.2% | 1.7% | 0.7% | 0.5% | 0.2% | 0.2% | 0.1% | 0.4% | 0.1% | 0.1% |
|  | k\_\_Bacteria;p\_\_Actinobacteria;c\_\_Actinobacteria;o\_\_Bifidobacteriales;f\_\_Bifidobacteriaceae | 0 | 0.3% | 0.0% | 0.0% | 0.0% | 0.0% | 0.1% | 0.0% | 0.0% | 0.0% | 0.0% | 0.0% | 0.1% | 0.0% | 0.0% | 0.1% | 0.0% | 0.0% | 0.0% | 0.0% | 0.0% | 0.0% | 0.1% | 6.2% | 0.0% |
|  | k\_\_Bacteria;p\_\_Firmicutes;c\_\_Bacilli;o\_\_Lactobacillales;f\_\_Streptococcaceae | 0 | 0.3% | 0.7% | 0.0% | 0.1% | 0.0% | 0.0% | 0.0% | 0.0% | 0.0% | 0.1% | 0.0% | 0.1% | 0.0% | 0.0% | 0.1% | 0.1% | 0.1% | 0.3% | 0.0% | 0.0% | 0.1% | 4.4% | 0.4% | 0.0% |
|  | k\_\_Bacteria;p\_\_Firmicutes;c\_\_Clostridia;o\_\_Clostridiales;f\_\_Peptostreptococcaceae | 0 | 0.2% | 0.0% | 0.1% | 0.1% | 0.1% | 0.0% | 0.0% | 0.1% | 0.1% | 0.1% | 0.1% | 0.2% | 0.2% | 0.0% | 1.0% | 0.1% | 0.2% | 0.0% | 0.0% | 0.0% | 0.5% | 0.3% | 1.4% | 0.8% |
|  | k\_\_Bacteria;p\_\_Actinobacteria;c\_\_Coriobacteriia;o\_\_Coriobacteriales;f\_\_Coriobacteriaceae | 0 | 0.2% | 0.0% | 0.0% | 0.0% | 0.0% | 0.0% | 0.0% | 0.0% | 0.0% | 0.1% | 0.0% | 0.1% | 0.0% | 0.0% | 0.0% | 0.0% | 0.3% | 0.1% | 0.0% | 0.0% | 0.1% | 0.1% | 4.1% | 0.0% |
|  | k\_\_Bacteria;p\_\_Firmicutes;c\_\_Bacilli;o\_\_Lactobacillales;f\_\_Lactobacillaceae | 0 | 0.2% | 0.0% | 0.0% | 0.1% | 0.2% | 0.0% | 0.0% | 0.0% | 0.0% | 0.0% | 0.0% | 0.1% | 0.0% | 0.0% | 0.1% | 0.2% | 0.6% | 0.1% | 0.0% | 0.1% | 0.1% | 0.1% | 3.4% | 0.0% |
|  | k\_\_Bacteria;p\_\_Proteobacteria;c\_\_Betaproteobacteria;o\_\_Neisseriales;f\_\_Neisseriaceae | 0 | 0.2% | 0.0% | 0.0% | 0.0% | 0.2% | 0.0% | 0.0% | 0.0% | 0.0% | 0.0% | 0.0% | 0.0% | 0.0% | 0.0% | 0.0% | 0.5% | 0.0% | 0.0% | 3.4% | 0.8% | 0.0% | 0.0% | 0.0% | 0.0% |
|  | k\_\_Bacteria;p\_\_Proteobacteria;c\_\_Gammaproteobacteria;o\_\_Pasteurellales;f\_\_Pasteurellaceae | 0 | 0.2% | 1.2% | 0.0% | 0.2% | 0.0% | 0.0% | 0.0% | 0.0% | 0.0% | 0.0% | 0.0% | 0.0% | 0.0% | 0.0% | 0.0% | 0.0% | 0.0% | 0.0% | 0.0% | 0.1% | 0.1% | 3.0% | 0.3% | 0.0% |
|  | k\_\_Bacteria;p\_\_Proteobacteria;c\_\_Alphaproteobacteria;o\_\_Rhizobiales;f\_\_Rhizobiaceae | 0 | 0.2% | 0.1% | 0.0% | 0.2% | 0.1% | 0.0% | 0.1% | 0.3% | 0.0% | 0.6% | 0.1% | 1.3% | 0.3% | 0.2% | 0.1% | 0.5% | 0.1% | 0.2% | 0.1% | 0.1% | 0.1% | 0.3% | 0.2% | 0.0% |
|  | k\_\_Bacteria;p\_\_Actinobacteria;c\_\_Actinobacteria;o\_\_Actinomycetales;f\_\_Nocardiaceae | 0 | 0.2% | 0.0% | 0.0% | 0.2% | 0.4% | 0.0% | 0.7% | 0.1% | 0.1% | 0.3% | 0.1% | 0.2% | 0.2% | 0.1% | 0.1% | 0.6% | 0.8% | 0.5% | 0.1% | 0.2% | 0.0% | 0.0% | 0.0% | 0.0% |
|  | k\_\_Bacteria;p\_\_Bacteroidetes;c\_\_Bacteroidia;o\_\_Bacteroidales;f\_\_Bacteroidaceae | 0 | 0.2% | 0.0% | 0.0% | 0.1% | 0.2% | 0.0% | 0.1% | 0.0% | 0.1% | 0.0% | 0.1% | 0.1% | 0.1% | 0.0% | 0.1% | 0.2% | 0.7% | 0.1% | 0.1% | 0.2% | 0.8% | 1.1% | 0.1% | 0.0% |
|  | k\_\_Bacteria;p\_\_Proteobacteria;c\_\_Alphaproteobacteria;o\_\_Rhizobiales;f\_\_Unclassified\_Rhizobiales | 0 | 0.2% | 0.0% | 0.0% | 0.3% | 0.4% | 0.0% | 0.2% | 0.1% | 0.1% | 0.3% | 0.0% | 0.2% | 0.1% | 0.0% | 0.3% | 0.5% | 0.5% | 0.4% | 0.2% | 0.1% | 0.1% | 0.1% | 0.1% | 0.0% |
|  | k\_\_Bacteria;p\_\_Bacteroidetes;c\_\_Bacteroidia;o\_\_Bacteroidales;f\_\_Unclassified\_Bacteroidales | 0 | 0.2% | 0.0% | 0.0% | 0.5% | 0.0% | 0.0% | 0.3% | 0.0% | 0.2% | 0.0% | 0.1% | 0.0% | 0.0% | 0.0% | 0.0% | 0.0% | 0.0% | 0.0% | 0.2% | 0.0% | 0.3% | 1.9% | 0.2% | 0.0% |
|  | k\_\_Bacteria;p\_\_Bacteroidetes;c\_\_Bacteroidia;o\_\_Bacteroidales;f\_\_Rikenellaceae | 0 | 0.2% | 0.0% | 0.0% | 0.0% | 0.0% | 0.0% | 0.0% | 0.0% | 0.1% | 0.0% | 0.0% | 0.0% | 0.0% | 0.0% | 0.1% | 0.0% | 0.4% | 0.0% | 1.4% | 0.0% | 0.2% | 1.4% | 0.1% | 0.0% |
|  | k\_\_Bacteria;p\_\_Proteobacteria;c\_\_Alphaproteobacteria;o\_\_Rhizobiales;f\_\_Phyllobacteriaceae | 0 | 0.2% | 0.0% | 0.0% | 0.5% | 0.3% | 0.0% | 0.1% | 0.0% | 0.1% | 0.3% | 0.0% | 0.2% | 0.1% | 0.0% | 0.2% | 0.6% | 0.3% | 0.5% | 0.2% | 0.1% | 0.1% | 0.1% | 0.1% | 0.0% |
|  | k\_\_Bacteria;p\_\_Proteobacteria;c\_\_Deltaproteobacteria;o\_\_Desulfovibrionales;f\_\_Desulfovibrionaceae | 0 | 0.2% | 0.0% | 0.0% | 0.0% | 0.0% | 0.0% | 0.0% | 0.0% | 0.1% | 0.0% | 0.0% | 0.0% | 0.1% | 0.2% | 0.0% | 0.0% | 0.7% | 0.0% | 0.2% | 0.3% | 0.4% | 1.3% | 0.1% | 0.0% |
|  | k\_\_Bacteria;p\_\_Firmicutes;c\_\_Clostridia;o\_\_Clostridiales;f\_\_Peptococcaceae | 0 | 0.2% | 0.2% | 0.0% | 0.0% | 0.0% | 0.0% | 0.0% | 0.0% | 0.1% | 0.0% | 0.0% | 0.2% | 0.0% | 0.1% | 0.0% | 0.0% | 0.5% | 0.2% | 0.3% | 0.0% | 0.6% | 0.9% | 0.5% | 0.0% |
|  | k\_\_Bacteria;p\_\_Proteobacteria;c\_\_Alphaproteobacteria;o\_\_Rhizobiales;f\_\_Bradyrhizobiaceae | 0 | 0.2% | 0.0% | 0.0% | 0.4% | 0.3% | 0.4% | 0.2% | 0.1% | 0.0% | 0.1% | 0.0% | 0.2% | 0.2% | 0.0% | 0.2% | 0.4% | 0.3% | 0.2% | 0.1% | 0.1% | 0.0% | 0.2% | 0.1% | 0.0% |
|  | k\_\_Bacteria;p\_\_Firmicutes;c\_\_Bacilli;o\_\_Bacillales;f\_\_Planococcaceae | 0 | 0.2% | 0.1% | 0.0% | 0.2% | 0.1% | 0.0% | 0.1% | 0.0% | 0.1% | 0.1% | 0.0% | 0.1% | 0.1% | 0.0% | 0.1% | 0.2% | 0.2% | 0.2% | 0.0% | 0.0% | 0.0% | 0.1% | 1.6% | 0.0% |
|  | k\_\_Bacteria;p\_\_Firmicutes;c\_\_Bacilli;o\_\_Bacillales;f\_\_Bacillaceae | 0 | 0.1% | 0.1% | 0.0% | 0.1% | 0.0% | 0.0% | 0.0% | 0.0% | 0.0% | 0.0% | 0.0% | 0.1% | 0.1% | 0.0% | 0.2% | 0.1% | 0.5% | 0.4% | 0.0% | 0.0% | 0.0% | 1.2% | 0.1% | 0.0% |
|  | k\_\_Bacteria;p\_\_Firmicutes;c\_\_Clostridia;o\_\_Clostridiales;f\_\_Veillonellaceae | 0 | 0.1% | 0.0% | 0.0% | 0.0% | 0.0% | 0.0% | 0.1% | 0.0% | 0.0% | 0.0% | 0.0% | 0.0% | 0.0% | 0.0% | 0.8% | 0.0% | 0.4% | 0.4% | 0.0% | 0.0% | 0.5% | 0.2% | 0.3% | 0.0% |
|  | k\_\_Bacteria;p\_\_Tenericutes;c\_\_Mollicutes;o\_\_Acholeplasmatales;f\_\_Acholeplasmataceae | 0 | 0.1% | 0.0% | 0.2% | 0.1% | 0.0% | 0.0% | 0.7% | 0.0% | 0.0% | 0.0% | 0.5% | 0.0% | 0.0% | 0.0% | 0.0% | 0.0% | 0.0% | 0.0% | 1.2% | 0.0% | 0.0% | 0.0% | 0.0% | 0.0% |
|  | k\_\_Bacteria;p\_\_Proteobacteria;c\_\_Alphaproteobacteria;o\_\_Sphingomonadales;f\_\_Unclassified\_Sphingomonadales | 0 | 0.1% | 0.0% | 0.0% | 0.4% | 0.8% | 0.0% | 0.0% | 0.0% | 0.0% | 0.1% | 0.0% | 0.1% | 0.0% | 0.0% | 0.2% | 0.2% | 0.1% | 0.2% | 0.1% | 0.0% | 0.0% | 0.1% | 0.1% | 0.0% |
|  | k\_\_Bacteria;p\_\_Firmicutes;c\_\_Erysipelotrichi;o\_\_Erysipelotrichales;f\_\_Erysipelotrichaceae | 0 | 0.1% | 0.0% | 0.0% | 0.1% | 0.1% | 0.0% | 0.1% | 0.0% | 0.0% | 0.0% | 0.0% | 0.0% | 0.0% | 0.0% | 0.2% | 0.1% | 0.9% | 0.0% | 0.1% | 0.0% | 0.2% | 0.3% | 0.2% | 0.0% |
|  | k\_\_Bacteria;p\_\_Verrucomicrobia;c\_\_Verrucomicrobiae;o\_\_Verrucomicrobiales;f\_\_Verrucomicrobiaceae | 0 | 0.1% | 0.0% | 0.0% | 0.1% | 0.0% | 0.0% | 0.0% | 0.0% | 0.1% | 0.0% | 0.0% | 0.1% | 0.1% | 0.0% | 0.0% | 0.0% | 0.3% | 0.0% | 0.0% | 0.0% | 0.3% | 1.0% | 0.1% | 0.0% |
|  | k\_\_Bacteria;p\_\_GN02;c\_\_3BR-5F;o\_\_Unclassified\_3BR-5F;f\_\_Unclassified\_3BR-5F | 0 | 0.1% | 0.0% | 0.0% | 0.1% | 0.4% | 0.0% | 0.1% | 0.0% | 0.0% | 0.0% | 0.0% | 0.1% | 0.0% | 0.0% | 0.2% | 0.3% | 0.4% | 0.1% | 0.1% | 0.1% | 0.0% | 0.1% | 0.1% | 0.0% |
|  | k\_\_Bacteria;p\_\_Bacteroidetes;c\_\_Bacteroidia;o\_\_Bacteroidales;f\_\_S24-7 | 0 | 0.1% | 0.0% | 0.0% | 0.1% | 0.0% | 0.0% | 0.1% | 0.0% | 0.0% | 0.0% | 0.0% | 0.0% | 0.0% | 0.0% | 0.5% | 0.2% | 0.9% | 0.1% | 0.0% | 0.0% | 0.0% | 0.1% | 0.0% | 0.0% |
|  | k\_\_Bacteria;p\_\_Actinobacteria;c\_\_Actinobacteria;o\_\_Actinomycetales;f\_\_Streptomycetaceae | 0 | 0.1% | 0.0% | 0.0% | 0.2% | 0.2% | 0.0% | 0.1% | 0.0% | 0.0% | 0.0% | 0.0% | 0.1% | 0.0% | 0.0% | 0.2% | 0.0% | 0.5% | 0.2% | 0.1% | 0.0% | 0.0% | 0.1% | 0.1% | 0.0% |
|  | k\_\_Bacteria;p\_\_Proteobacteria;c\_\_Gammaproteobacteria;o\_\_Xanthomonadales;f\_\_Sinobacteraceae | 0 | 0.1% | 0.0% | 0.0% | 0.2% | 0.2% | 0.0% | 0.0% | 0.0% | 0.0% | 0.0% | 0.0% | 0.0% | 0.1% | 0.0% | 0.1% | 0.3% | 0.2% | 0.2% | 0.0% | 0.2% | 0.0% | 0.2% | 0.1% | 0.1% |
|  | k\_\_Bacteria;p\_\_Cyanobacteria;c\_\_Chloroplast;o\_\_Streptophyta;f\_\_Unclassified\_Streptophyta | 0 | 0.1% | 0.0% | 0.0% | 0.3% | 0.1% | 0.0% | 0.1% | 0.0% | 0.0% | 0.1% | 0.0% | 0.1% | 0.0% | 0.0% | 0.1% | 0.2% | 0.1% | 0.1% | 0.1% | 0.1% | 0.1% | 0.1% | 0.0% | 0.0% |
|  | k\_\_Bacteria;p\_\_Proteobacteria;c\_\_Gammaproteobacteria;o\_\_Aeromonadales;f\_\_Aeromonadaceae | 0 | 0.1% | 0.0% | 0.0% | 0.4% | 0.0% | 0.0% | 0.0% | 0.0% | 0.0% | 0.0% | 0.0% | 0.1% | 0.0% | 0.0% | 0.0% | 0.3% | 0.2% | 0.1% | 0.1% | 0.0% | 0.0% | 0.1% | 0.1% | 0.0% |
|  | k\_\_Bacteria;p\_\_Firmicutes;c\_\_Clostridia;o\_\_Clostridiales;f\_\_Christensenellaceae | 0 | 0.1% | 0.0% | 0.0% | 0.1% | 0.0% | 0.0% | 0.0% | 0.0% | 0.0% | 0.0% | 0.0% | 0.0% | 0.0% | 0.0% | 0.1% | 0.0% | 0.2% | 0.0% | 0.0% | 0.0% | 0.1% | 0.8% | 0.1% | 0.0% |
|  | k\_\_Bacteria;p\_\_Actinobacteria;c\_\_Actinobacteria;o\_\_Actinomycetales;f\_\_Propionibacteriaceae | 0 | 0.1% | 0.1% | 0.1% | 0.1% | 0.1% | 0.0% | 0.1% | 0.0% | 0.1% | 0.0% | 0.1% | 0.1% | 0.0% | 0.1% | 0.0% | 0.1% | 0.0% | 0.0% | 0.1% | 0.0% | 0.0% | 0.2% | 0.0% | 0.1% |
|  | k\_\_Bacteria;p\_\_SR1;c\_\_Unclassified\_SR1;o\_\_Unclassified\_SR1;f\_\_Unclassified\_SR1 | 0 | 0.1% | 0.0% | 0.0% | 0.0% | 0.0% | 0.0% | 0.0% | 0.0% | 0.0% | 0.0% | 0.0% | 0.0% | 0.0% | 0.0% | 0.7% | 0.0% | 0.0% | 0.0% | 0.1% | 0.0% | 0.5% | 0.0% | 0.0% | 0.0% |
|  | k\_\_Bacteria;p\_\_Bacteroidetes;c\_\_Cytophagia;o\_\_Cytophagales;f\_\_Cytophagaceae | 0 | 0.1% | 0.0% | 0.0% | 0.0% | 0.0% | 0.0% | 0.0% | 0.0% | 0.0% | 0.0% | 0.0% | 0.0% | 0.0% | 1.4% | 0.0% | 0.0% | 0.0% | 0.0% | 0.0% | 0.0% | 0.0% | 0.0% | 0.0% | 0.0% |
|  | k\_\_Bacteria;p\_\_Proteobacteria;c\_\_Deltaproteobacteria;o\_\_Myxococcales;f\_\_Myxococcaceae | 0 | 0.1% | 0.0% | 0.0% | 0.1% | 0.1% | 0.0% | 0.0% | 0.0% | 0.0% | 0.0% | 0.0% | 0.1% | 0.0% | 0.0% | 0.1% | 0.1% | 0.4% | 0.0% | 0.1% | 0.0% | 0.0% | 0.1% | 0.1% | 0.0% |
|  | k\_\_Bacteria;p\_\_Actinobacteria;c\_\_Actinobacteria;o\_\_Actinomycetales;f\_\_Nocardioidaceae | 0 | 0.1% | 0.0% | 0.0% | 0.1% | 0.0% | 0.1% | 0.0% | 0.1% | 0.0% | 0.2% | 0.0% | 0.4% | 0.2% | 0.0% | 0.0% | 0.1% | 0.0% | 0.0% | 0.0% | 0.0% | 0.1% | 0.1% | 0.0% | 0.0% |
|  | k\_\_Bacteria;p\_\_Bacteroidetes;c\_\_Flavobacteriia;o\_\_Flavobacteriales;f\_\_Flavobacteriaceae | 0 | 0.1% | 0.0% | 0.1% | 0.0% | 0.0% | 0.0% | 0.0% | 0.0% | 0.0% | 0.0% | 0.7% | 0.0% | 0.0% | 0.0% | 0.0% | 0.2% | 0.1% | 0.0% | 0.0% | 0.0% | 0.0% | 0.0% | 0.0% | 0.1% |
|  | k\_\_Bacteria;p\_\_Lentisphaerae;c\_\_[Lentisphaeria];o\_\_Victivallales;f\_\_Victivallaceae | 0 | 0.1% | 0.0% | 0.0% | 0.0% | 0.0% | 0.0% | 0.0% | 0.0% | 0.1% | 0.0% | 0.0% | 0.0% | 0.0% | 0.0% | 0.0% | 0.0% | 0.5% | 0.0% | 0.0% | 0.0% | 0.1% | 0.5% | 0.0% | 0.0% |
|  | k\_\_Bacteria;p\_\_Actinobacteria;c\_\_Actinobacteria;o\_\_Actinomycetales;f\_\_Brevibacteriaceae | 0 | 0.1% | 0.0% | 0.0% | 0.1% | 0.5% | 0.0% | 0.0% | 0.0% | 0.0% | 0.0% | 0.0% | 0.0% | 0.0% | 0.0% | 0.0% | 0.1% | 0.2% | 0.1% | 0.0% | 0.0% | 0.0% | 0.1% | 0.0% | 0.0% |
|  | k\_\_Bacteria;p\_\_Verrucomicrobia;c\_\_Verruco-5;o\_\_WCHB1-41;f\_\_RFP12 | 0 | 0.1% | 0.0% | 0.0% | 0.0% | 0.0% | 0.0% | 0.0% | 0.0% | 0.1% | 0.0% | 0.0% | 0.0% | 0.1% | 0.0% | 0.0% | 0.0% | 0.4% | 0.0% | 0.0% | 0.0% | 0.1% | 0.5% | 0.0% | 0.0% |
|  | k\_\_Bacteria;p\_\_Spirochaetes;c\_\_Spirochaetes;o\_\_Spirochaetales;f\_\_Spirochaetaceae | 0 | 0.1% | 0.0% | 0.0% | 0.1% | 0.1% | 0.0% | 0.1% | 0.0% | 0.0% | 0.1% | 0.1% | 0.0% | 0.1% | 0.1% | 0.1% | 0.0% | 0.1% | 0.0% | 0.1% | 0.0% | 0.0% | 0.2% | 0.0% | 0.1% |
|  | k\_\_Bacteria;p\_\_[Thermi];c\_\_Deinococci;o\_\_Deinococcales;f\_\_Deinococcaceae | 0 | 0.1% | 0.0% | 0.0% | 0.1% | 0.1% | 0.0% | 0.1% | 0.0% | 0.0% | 0.0% | 0.0% | 0.0% | 0.0% | 0.0% | 0.1% | 0.2% | 0.2% | 0.1% | 0.0% | 0.0% | 0.0% | 0.1% | 0.0% | 0.0% |
|  | k\_\_Bacteria;p\_\_Proteobacteria;c\_\_Deltaproteobacteria;o\_\_Myxococcales;f\_\_Unclassified\_Myxococcales | 0 | 0.1% | 0.0% | 0.0% | 0.0% | 0.0% | 0.0% | 0.0% | 0.0% | 0.0% | 0.0% | 0.0% | 0.0% | 0.0% | 0.0% | 0.1% | 0.2% | 0.2% | 0.0% | 0.1% | 0.0% | 0.0% | 0.4% | 0.0% | 0.0% |
|  | k\_\_Bacteria;p\_\_Planctomycetes;c\_\_Phycisphaerae;o\_\_Phycisphaerales;f\_\_Unclassified\_Phycisphaerales | 0 | 0.0% | 0.0% | 0.0% | 0.1% | 0.1% | 0.0% | 0.0% | 0.0% | 0.0% | 0.0% | 0.0% | 0.0% | 0.0% | 0.0% | 0.1% | 0.1% | 0.2% | 0.2% | 0.1% | 0.0% | 0.0% | 0.0% | 0.0% | 0.0% |
|  | k\_\_Bacteria;p\_\_Proteobacteria;c\_\_Alphaproteobacteria;o\_\_Rhodospirillales;f\_\_Rhodospirillaceae | 0 | 0.0% | 0.0% | 0.0% | 0.1% | 0.1% | 0.0% | 0.0% | 0.0% | 0.0% | 0.1% | 0.0% | 0.1% | 0.0% | 0.0% | 0.1% | 0.1% | 0.1% | 0.1% | 0.0% | 0.1% | 0.0% | 0.1% | 0.0% | 0.0% |
|  | k\_\_Bacteria;p\_\_Gemmatimonadetes;c\_\_Gemm-1;o\_\_Unclassified\_Gemm-1;f\_\_Unclassified\_Gemm-1 | 0 | 0.0% | 0.0% | 0.0% | 0.0% | 0.0% | 0.0% | 0.0% | 0.0% | 0.0% | 0.0% | 0.0% | 0.0% | 0.0% | 0.0% | 0.0% | 0.0% | 0.1% | 0.0% | 0.0% | 0.0% | 0.0% | 0.8% | 0.1% | 0.0% |
|  | k\_\_Bacteria;p\_\_Proteobacteria;c\_\_Alphaproteobacteria;o\_\_Rhodospirillales;f\_\_Acetobacteraceae | 0 | 0.0% | 0.0% | 0.0% | 0.1% | 0.1% | 0.0% | 0.0% | 0.0% | 0.0% | 0.1% | 0.0% | 0.0% | 0.0% | 0.0% | 0.2% | 0.1% | 0.1% | 0.1% | 0.1% | 0.0% | 0.0% | 0.1% | 0.0% | 0.0% |
|  | k\_\_Bacteria;p\_\_Bacteroidetes;c\_\_Flavobacteriia;o\_\_Flavobacteriales;f\_\_[Weeksellaceae] | 0 | 0.0% | 0.0% | 0.0% | 0.1% | 0.0% | 0.0% | 0.0% | 0.0% | 0.0% | 0.0% | 0.0% | 0.0% | 0.0% | 0.0% | 0.1% | 0.1% | 0.0% | 0.0% | 0.0% | 0.0% | 0.0% | 0.5% | 0.3% | 0.0% |
|  | k\_\_Bacteria;p\_\_Proteobacteria;c\_\_Alphaproteobacteria;o\_\_Rhizobiales;f\_\_Hyphomicrobiaceae | 0 | 0.0% | 0.0% | 0.0% | 0.1% | 0.1% | 0.0% | 0.0% | 0.0% | 0.0% | 0.0% | 0.0% | 0.0% | 0.0% | 0.0% | 0.1% | 0.2% | 0.1% | 0.0% | 0.0% | 0.0% | 0.0% | 0.1% | 0.0% | 0.1% |
|  | k\_\_Bacteria;p\_\_Acidobacteria;c\_\_Solibacteres;o\_\_Solibacterales;f\_\_Unclassified\_Solibacterales | 0 | 0.0% | 0.0% | 0.0% | 0.1% | 0.1% | 0.0% | 0.0% | 0.0% | 0.0% | 0.0% | 0.0% | 0.0% | 0.0% | 0.0% | 0.0% | 0.1% | 0.2% | 0.0% | 0.0% | 0.0% | 0.0% | 0.1% | 0.0% | 0.0% |
|  | k\_\_Bacteria;p\_\_Firmicutes;c\_\_Clostridia;o\_\_Clostridiales;f\_\_[Mogibacteriaceae] | 0 | 0.0% | 0.0% | 0.0% | 0.1% | 0.0% | 0.0% | 0.0% | 0.0% | 0.0% | 0.0% | 0.0% | 0.1% | 0.1% | 0.0% | 0.0% | 0.0% | 0.0% | 0.0% | 0.0% | 0.0% | 0.3% | 0.3% | 0.0% | 0.0% |
|  | k\_\_Bacteria;p\_\_Acidobacteria;c\_\_Acidobacteria-6;o\_\_iii1-15;f\_\_Unclassified\_iii1-15 | 0 | 0.0% | 0.0% | 0.0% | 0.1% | 0.0% | 0.0% | 0.0% | 0.0% | 0.0% | 0.0% | 0.0% | 0.0% | 0.0% | 0.0% | 0.0% | 0.1% | 0.0% | 0.0% | 0.0% | 0.0% | 0.0% | 0.6% | 0.0% | 0.0% |
|  | k\_\_Bacteria;p\_\_Proteobacteria;c\_\_Alphaproteobacteria;o\_\_Rhodobacterales;f\_\_Rhodobacteraceae | 0 | 0.0% | 0.0% | 0.0% | 0.1% | 0.1% | 0.1% | 0.0% | 0.0% | 0.0% | 0.0% | 0.0% | 0.0% | 0.0% | 0.0% | 0.0% | 0.1% | 0.1% | 0.1% | 0.0% | 0.0% | 0.0% | 0.0% | 0.0% | 0.0% |
|  | k\_\_Bacteria;p\_\_TM7;c\_\_TM7-3;o\_\_CW040;f\_\_F16 | 0 | 0.0% | 0.0% | 0.0% | 0.1% | 0.1% | 0.0% | 0.0% | 0.0% | 0.0% | 0.0% | 0.0% | 0.0% | 0.1% | 0.0% | 0.2% | 0.0% | 0.1% | 0.0% | 0.0% | 0.0% | 0.0% | 0.0% | 0.0% | 0.0% |
|  | k\_\_Bacteria;p\_\_Actinobacteria;c\_\_Actinobacteria;o\_\_Actinomycetales;f\_\_Unclassified\_Actinomycetales | 0 | 0.0% | 0.0% | 0.0% | 0.0% | 0.3% | 0.0% | 0.0% | 0.0% | 0.0% | 0.0% | 0.0% | 0.1% | 0.0% | 0.0% | 0.0% | 0.0% | 0.0% | 0.1% | 0.0% | 0.0% | 0.0% | 0.1% | 0.0% | 0.0% |
|  | k\_\_Bacteria;p\_\_Proteobacteria;c\_\_Betaproteobacteria;o\_\_SC-I-84;f\_\_Unclassified\_SC-I-84 | 0 | 0.0% | 0.0% | 0.0% | 0.0% | 0.0% | 0.0% | 0.0% | 0.0% | 0.0% | 0.0% | 0.0% | 0.0% | 0.0% | 0.0% | 0.0% | 0.0% | 0.0% | 0.0% | 0.0% | 0.0% | 0.0% | 0.5% | 0.0% | 0.0% |
|  | k\_\_Bacteria;p\_\_Cyanobacteria;c\_\_4C0d-2;o\_\_YS2;f\_\_Unclassified\_YS2 | 0 | 0.0% | 0.0% | 0.0% | 0.0% | 0.0% | 0.0% | 0.0% | 0.0% | 0.0% | 0.0% | 0.0% | 0.0% | 0.1% | 0.0% | 0.0% | 0.0% | 0.1% | 0.0% | 0.0% | 0.0% | 0.0% | 0.3% | 0.0% | 0.0% |
|  | k\_\_Bacteria;p\_\_Bacteroidetes;c\_\_Bacteroidia;o\_\_Bacteroidales;f\_\_[Paraprevotellaceae] | 0 | 0.0% | 0.0% | 0.0% | 0.0% | 0.0% | 0.0% | 0.0% | 0.0% | 0.0% | 0.0% | 0.0% | 0.0% | 0.0% | 0.0% | 0.0% | 0.0% | 0.1% | 0.0% | 0.0% | 0.0% | 0.1% | 0.1% | 0.0% | 0.0% |
|  | k\_\_Bacteria;p\_\_Actinobacteria;c\_\_Rubrobacteria;o\_\_Rubrobacterales;f\_\_Rubrobacteraceae | 0 | 0.0% | 0.0% | 0.0% | 0.1% | 0.0% | 0.0% | 0.0% | 0.0% | 0.0% | 0.0% | 0.0% | 0.0% | 0.0% | 0.0% | 0.0% | 0.0% | 0.2% | 0.0% | 0.0% | 0.0% | 0.0% | 0.0% | 0.0% | 0.0% |
|  | k\_\_Bacteria;p\_\_Bacteroidetes;c\_\_Bacteroidia;o\_\_Bacteroidales;f\_\_[Barnesiellaceae] | 0 | 0.0% | 0.0% | 0.0% | 0.0% | 0.0% | 0.0% | 0.0% | 0.0% | 0.0% | 0.0% | 0.0% | 0.0% | 0.0% | 0.0% | 0.0% | 0.0% | 0.2% | 0.0% | 0.0% | 0.0% | 0.0% | 0.2% | 0.0% | 0.0% |
|  | k\_\_Bacteria;p\_\_Chloroflexi;c\_\_Anaerolineae;o\_\_SBR1031;f\_\_A4b | 0 | 0.0% | 0.0% | 0.0% | 0.0% | 0.0% | 0.0% | 0.0% | 0.0% | 0.0% | 0.0% | 0.0% | 0.0% | 0.0% | 0.0% | 0.1% | 0.1% | 0.0% | 0.0% | 0.0% | 0.0% | 0.0% | 0.0% | 0.0% | 0.1% |
|  | k\_\_Bacteria;p\_\_Firmicutes;c\_\_Bacilli;o\_\_Turicibacterales;f\_\_Turicibacteraceae | 0 | 0.0% | 0.0% | 0.0% | 0.0% | 0.0% | 0.0% | 0.0% | 0.0% | 0.0% | 0.0% | 0.0% | 0.1% | 0.0% | 0.0% | 0.1% | 0.0% | 0.1% | 0.0% | 0.0% | 0.0% | 0.0% | 0.1% | 0.1% | 0.0% |
|  | k\_\_Bacteria;p\_\_Tenericutes;c\_\_Mollicutes;o\_\_Mycoplasmatales;f\_\_Mycoplasmataceae | 0 | 0.0% | 0.0% | 0.0% | 0.0% | 0.0% | 0.0% | 0.0% | 0.0% | 0.0% | 0.0% | 0.0% | 0.0% | 0.0% | 0.0% | 0.0% | 0.0% | 0.0% | 0.0% | 0.3% | 0.0% | 0.0% | 0.0% | 0.0% | 0.1% |
|  | k\_\_Bacteria;p\_\_Proteobacteria;c\_\_Gammaproteobacteria;o\_\_Cardiobacteriales;f\_\_Cardiobacteriaceae | 0 | 0.0% | 0.0% | 0.0% | 0.0% | 0.0% | 0.0% | 0.0% | 0.0% | 0.0% | 0.0% | 0.0% | 0.0% | 0.0% | 0.0% | 0.0% | 0.0% | 0.0% | 0.0% | 0.0% | 0.0% | 0.4% | 0.0% | 0.0% | 0.0% |
|  | k\_\_Bacteria;p\_\_Firmicutes;c\_\_Bacilli;o\_\_Lactobacillales;f\_\_Enterococcaceae | 0 | 0.0% | 0.1% | 0.0% | 0.0% | 0.0% | 0.1% | 0.0% | 0.0% | 0.0% | 0.0% | 0.0% | 0.0% | 0.0% | 0.0% | 0.0% | 0.0% | 0.1% | 0.0% | 0.0% | 0.0% | 0.0% | 0.1% | 0.1% | 0.0% |
|  | k\_\_Bacteria;p\_\_Actinobacteria;c\_\_Actinobacteria;o\_\_Actinomycetales;f\_\_Micromonosporaceae | 0 | 0.0% | 0.0% | 0.0% | 0.0% | 0.0% | 0.0% | 0.0% | 0.0% | 0.0% | 0.0% | 0.0% | 0.0% | 0.0% | 0.0% | 0.1% | 0.0% | 0.2% | 0.0% | 0.0% | 0.0% | 0.0% | 0.0% | 0.0% | 0.0% |
|  | k\_\_Bacteria;p\_\_Firmicutes;c\_\_Bacilli;o\_\_Bacillales;f\_\_Staphylococcaceae | 0 | 0.0% | 0.0% | 0.0% | 0.1% | 0.0% | 0.0% | 0.0% | 0.0% | 0.0% | 0.1% | 0.0% | 0.0% | 0.0% | 0.0% | 0.0% | 0.0% | 0.0% | 0.1% | 0.0% | 0.0% | 0.0% | 0.0% | 0.0% | 0.0% |
|  | k\_\_Bacteria;p\_\_Actinobacteria;c\_\_Actinobacteria;o\_\_Actinomycetales;f\_\_Mycobacteriaceae | 0 | 0.0% | 0.0% | 0.0% | 0.0% | 0.0% | 0.0% | 0.0% | 0.0% | 0.0% | 0.0% | 0.0% | 0.0% | 0.0% | 0.0% | 0.1% | 0.0% | 0.1% | 0.0% | 0.0% | 0.0% | 0.0% | 0.0% | 0.0% | 0.0% |
|  | k\_\_Bacteria;p\_\_Tenericutes;c\_\_Mollicutes;o\_\_RF39;f\_\_Unclassified\_RF39 | 0 | 0.0% | 0.0% | 0.0% | 0.0% | 0.0% | 0.0% | 0.0% | 0.0% | 0.1% | 0.0% | 0.0% | 0.1% | 0.0% | 0.0% | 0.0% | 0.0% | 0.1% | 0.0% | 0.0% | 0.0% | 0.0% | 0.1% | 0.0% | 0.0% |
|  | k\_\_Bacteria;p\_\_Gemmatimonadetes;c\_\_Gemmatimonadetes;o\_\_Unclassified\_Gemmatimonadetes;f\_\_Unclassified\_Gemmatimonadetes | 0 | 0.0% | 0.0% | 0.0% | 0.0% | 0.0% | 0.0% | 0.0% | 0.0% | 0.0% | 0.0% | 0.0% | 0.0% | 0.0% | 0.0% | 0.0% | 0.1% | 0.0% | 0.0% | 0.0% | 0.0% | 0.0% | 0.2% | 0.0% | 0.0% |
|  | k\_\_Bacteria;p\_\_Bacteroidetes;c\_\_Bacteroidia;o\_\_Bacteroidales;f\_\_RF16 | 0 | 0.0% | 0.0% | 0.0% | 0.0% | 0.0% | 0.0% | 0.0% | 0.0% | 0.0% | 0.0% | 0.0% | 0.0% | 0.0% | 0.0% | 0.0% | 0.0% | 0.0% | 0.0% | 0.0% | 0.0% | 0.1% | 0.2% | 0.0% | 0.0% |
|  | k\_\_Bacteria;p\_\_Actinobacteria;c\_\_Actinobacteria;o\_\_Actinomycetales;f\_\_Dietziaceae | 0 | 0.0% | 0.0% | 0.0% | 0.0% | 0.0% | 0.0% | 0.0% | 0.0% | 0.0% | 0.0% | 0.0% | 0.0% | 0.0% | 0.0% | 0.1% | 0.0% | 0.1% | 0.0% | 0.0% | 0.0% | 0.0% | 0.0% | 0.0% | 0.0% |
|  | k\_\_Bacteria;p\_\_Actinobacteria;c\_\_Acidimicrobiia;o\_\_Acidimicrobiales;f\_\_Unclassified\_Acidimicrobiales | 0 | 0.0% | 0.0% | 0.0% | 0.0% | 0.0% | 0.0% | 0.0% | 0.0% | 0.0% | 0.1% | 0.0% | 0.0% | 0.0% | 0.0% | 0.1% | 0.0% | 0.0% | 0.0% | 0.0% | 0.0% | 0.0% | 0.1% | 0.0% | 0.0% |
|  | k\_\_Bacteria;p\_\_Acidobacteria;c\_\_Acidobacteriia;o\_\_Acidobacteriales;f\_\_Koribacteraceae | 0 | 0.0% | 0.0% | 0.0% | 0.1% | 0.0% | 0.0% | 0.0% | 0.0% | 0.0% | 0.0% | 0.0% | 0.0% | 0.0% | 0.0% | 0.0% | 0.1% | 0.0% | 0.0% | 0.0% | 0.0% | 0.0% | 0.1% | 0.0% | 0.0% |
|  | k\_\_Bacteria;p\_\_Proteobacteria;c\_\_Alphaproteobacteria;o\_\_Unclassified\_Alphaproteobacteria;f\_\_Unclassified\_Alphaproteobacteria | 0 | 0.0% | 0.0% | 0.0% | 0.0% | 0.0% | 0.0% | 0.0% | 0.0% | 0.0% | 0.0% | 0.0% | 0.0% | 0.0% | 0.0% | 0.0% | 0.0% | 0.0% | 0.0% | 0.0% | 0.0% | 0.1% | 0.1% | 0.0% | 0.0% |
|  | k\_\_Bacteria;p\_\_Firmicutes;c\_\_Bacilli;o\_\_Gemellales;f\_\_Gemellaceae | 0 | 0.0% | 0.2% | 0.0% | 0.0% | 0.0% | 0.0% | 0.0% | 0.0% | 0.0% | 0.0% | 0.0% | 0.0% | 0.0% | 0.0% | 0.0% | 0.0% | 0.0% | 0.0% | 0.0% | 0.0% | 0.0% | 0.0% | 0.0% | 0.0% |
|  | k\_\_Bacteria;p\_\_Bacteroidetes;c\_\_Bacteroidia;o\_\_Bacteroidales;f\_\_Prevotellaceae | 0 | 0.0% | 0.0% | 0.0% | 0.0% | 0.0% | 0.0% | 0.0% | 0.0% | 0.0% | 0.0% | 0.0% | 0.0% | 0.0% | 0.0% | 0.0% | 0.0% | 0.0% | 0.0% | 0.0% | 0.0% | 0.2% | 0.0% | 0.0% | 0.0% |
|  | k\_\_Bacteria;p\_\_Proteobacteria;c\_\_Betaproteobacteria;o\_\_MND1;f\_\_Unclassified\_MND1 | 0 | 0.0% | 0.0% | 0.0% | 0.0% | 0.0% | 0.0% | 0.0% | 0.0% | 0.0% | 0.0% | 0.0% | 0.0% | 0.0% | 0.0% | 0.0% | 0.0% | 0.1% | 0.0% | 0.0% | 0.0% | 0.0% | 0.2% | 0.0% | 0.0% |
|  | k\_\_Bacteria;p\_\_Proteobacteria;c\_\_Deltaproteobacteria;o\_\_Syntrophobacterales;f\_\_Syntrophobacteraceae | 0 | 0.0% | 0.0% | 0.0% | 0.0% | 0.0% | 0.0% | 0.0% | 0.0% | 0.0% | 0.0% | 0.0% | 0.0% | 0.0% | 0.0% | 0.0% | 0.1% | 0.1% | 0.0% | 0.0% | 0.0% | 0.0% | 0.1% | 0.0% | 0.0% |
|  | k\_\_Bacteria;p\_\_Lentisphaerae;c\_\_[Lentisphaeria];o\_\_Unclassified\_[Lentisphaeria];f\_\_Unclassified\_[Lentisphaeria] | 0 | 0.0% | 0.0% | 0.0% | 0.0% | 0.0% | 0.0% | 0.0% | 0.0% | 0.0% | 0.0% | 0.0% | 0.0% | 0.1% | 0.0% | 0.0% | 0.0% | 0.0% | 0.0% | 0.0% | 0.0% | 0.2% | 0.0% | 0.0% | 0.0% |
|  | k\_\_Bacteria;p\_\_Chlamydiae;c\_\_Chlamydiia;o\_\_Chlamydiales;f\_\_Rhabdochlamydiaceae | 0 | 0.0% | 0.0% | 0.0% | 0.0% | 0.0% | 0.0% | 0.0% | 0.0% | 0.0% | 0.0% | 0.0% | 0.0% | 0.0% | 0.0% | 0.0% | 0.1% | 0.0% | 0.0% | 0.0% | 0.0% | 0.0% | 0.0% | 0.0% | 0.0% |
|  | k\_\_Bacteria;p\_\_Actinobacteria;c\_\_Actinobacteria;o\_\_Actinomycetales;f\_\_Geodermatophilaceae | 0 | 0.0% | 0.0% | 0.0% | 0.0% | 0.0% | 0.0% | 0.0% | 0.0% | 0.0% | 0.0% | 0.0% | 0.0% | 0.1% | 0.0% | 0.0% | 0.1% | 0.0% | 0.0% | 0.0% | 0.0% | 0.0% | 0.0% | 0.0% | 0.0% |
|  | k\_\_Bacteria;p\_\_Firmicutes;c\_\_Bacilli;o\_\_Bacillales;f\_\_Unclassified\_Bacillales | 0 | 0.0% | 0.0% | 0.0% | 0.0% | 0.1% | 0.0% | 0.0% | 0.0% | 0.0% | 0.0% | 0.0% | 0.0% | 0.0% | 0.0% | 0.0% | 0.0% | 0.1% | 0.0% | 0.0% | 0.0% | 0.0% | 0.0% | 0.0% | 0.0% |
|  | k\_\_Bacteria;p\_\_Actinobacteria;c\_\_Thermoleophilia;o\_\_Gaiellales;f\_\_Gaiellaceae | 0 | 0.0% | 0.0% | 0.0% | 0.2% | 0.0% | 0.0% | 0.0% | 0.0% | 0.0% | 0.0% | 0.0% | 0.0% | 0.0% | 0.0% | 0.0% | 0.0% | 0.0% | 0.0% | 0.0% | 0.0% | 0.0% | 0.0% | 0.0% | 0.0% |
|  | k\_\_Bacteria;p\_\_TM7;c\_\_TM7-3;o\_\_Unclassified\_TM7-3;f\_\_Unclassified\_TM7-3 | 0 | 0.0% | 0.0% | 0.0% | 0.0% | 0.0% | 0.0% | 0.0% | 0.0% | 0.0% | 0.0% | 0.0% | 0.0% | 0.0% | 0.0% | 0.1% | 0.0% | 0.0% | 0.0% | 0.0% | 0.2% | 0.0% | 0.0% | 0.0% | 0.0% |
|  | k\_\_Bacteria;p\_\_Proteobacteria;c\_\_Deltaproteobacteria;o\_\_MIZ46;f\_\_Unclassified\_MIZ46 | 0 | 0.0% | 0.0% | 0.0% | 0.0% | 0.0% | 0.0% | 0.0% | 0.0% | 0.0% | 0.0% | 0.0% | 0.0% | 0.0% | 0.0% | 0.0% | 0.1% | 0.0% | 0.0% | 0.0% | 0.0% | 0.0% | 0.0% | 0.0% | 0.0% |
|  | k\_\_Bacteria;p\_\_Bacteroidetes;c\_\_Sphingobacteriia;o\_\_Sphingobacteriales;f\_\_Unclassified\_Sphingobacteriales | 0 | 0.0% | 0.0% | 0.0% | 0.0% | 0.1% | 0.0% | 0.0% | 0.0% | 0.0% | 0.0% | 0.0% | 0.0% | 0.0% | 0.0% | 0.0% | 0.0% | 0.0% | 0.0% | 0.0% | 0.0% | 0.0% | 0.0% | 0.0% | 0.0% |
|  | k\_\_Bacteria;p\_\_Proteobacteria;c\_\_Deltaproteobacteria;o\_\_Myxococcales;f\_\_Haliangiaceae | 0 | 0.0% | 0.0% | 0.0% | 0.1% | 0.0% | 0.0% | 0.0% | 0.0% | 0.0% | 0.0% | 0.0% | 0.0% | 0.0% | 0.0% | 0.0% | 0.0% | 0.0% | 0.0% | 0.0% | 0.0% | 0.0% | 0.1% | 0.0% | 0.0% |
|  | k\_\_Bacteria;p\_\_Gemmatimonadetes;c\_\_Gemmatimonadetes;o\_\_N1423WL;f\_\_Unclassified\_N1423WL | 0 | 0.0% | 0.0% | 0.0% | 0.0% | 0.0% | 0.0% | 0.0% | 0.0% | 0.0% | 0.0% | 0.0% | 0.0% | 0.0% | 0.0% | 0.0% | 0.0% | 0.0% | 0.0% | 0.0% | 0.0% | 0.0% | 0.2% | 0.0% | 0.0% |
|  | k\_\_Bacteria;p\_\_Proteobacteria;c\_\_Deltaproteobacteria;o\_\_Bdellovibrionales;f\_\_Bdellovibrionaceae | 0 | 0.0% | 0.0% | 0.0% | 0.0% | 0.0% | 0.0% | 0.0% | 0.0% | 0.0% | 0.0% | 0.0% | 0.0% | 0.0% | 0.0% | 0.0% | 0.0% | 0.1% | 0.0% | 0.0% | 0.0% | 0.0% | 0.0% | 0.0% | 0.0% |
|  | k\_\_Bacteria;p\_\_Bacteroidetes;c\_\_Sphingobacteriia;o\_\_Sphingobacteriales;f\_\_Sphingobacteriaceae | 0 | 0.0% | 0.0% | 0.0% | 0.0% | 0.0% | 0.0% | 0.0% | 0.0% | 0.0% | 0.0% | 0.0% | 0.0% | 0.0% | 0.0% | 0.0% | 0.0% | 0.0% | 0.0% | 0.0% | 0.0% | 0.0% | 0.1% | 0.0% | 0.0% |
|  | k\_\_Bacteria;p\_\_Tenericutes;c\_\_RF3;o\_\_ML615J-28;f\_\_Unclassified\_ML615J-28 | 0 | 0.0% | 0.0% | 0.0% | 0.0% | 0.0% | 0.0% | 0.0% | 0.0% | 0.0% | 0.0% | 0.0% | 0.0% | 0.0% | 0.0% | 0.0% | 0.0% | 0.0% | 0.0% | 0.0% | 0.0% | 0.1% | 0.1% | 0.0% | 0.0% |
|  | k\_\_Bacteria;p\_\_Chloroflexi;c\_\_Anaerolineae;o\_\_H39;f\_\_Unclassified\_H39 | 0 | 0.0% | 0.0% | 0.0% | 0.0% | 0.0% | 0.0% | 0.0% | 0.0% | 0.0% | 0.0% | 0.0% | 0.0% | 0.0% | 0.0% | 0.0% | 0.0% | 0.2% | 0.0% | 0.0% | 0.0% | 0.0% | 0.0% | 0.0% | 0.0% |
|  | k\_\_Bacteria;p\_\_Proteobacteria;c\_\_Alphaproteobacteria;o\_\_Rhodobacterales;f\_\_Hyphomonadaceae | 0 | 0.0% | 0.0% | 0.0% | 0.0% | 0.0% | 0.0% | 0.0% | 0.0% | 0.0% | 0.0% | 0.0% | 0.0% | 0.0% | 0.0% | 0.0% | 0.0% | 0.0% | 0.0% | 0.0% | 0.0% | 0.0% | 0.0% | 0.0% | 0.1% |
|  | k\_\_Bacteria;p\_\_Proteobacteria;c\_\_Betaproteobacteria;o\_\_Unclassified\_Betaproteobacteria;f\_\_Unclassified\_Betaproteobacteria | 0 | 0.0% | 0.0% | 0.0% | 0.0% | 0.0% | 0.0% | 0.0% | 0.0% | 0.0% | 0.0% | 0.0% | 0.0% | 0.0% | 0.0% | 0.0% | 0.0% | 0.1% | 0.0% | 0.0% | 0.0% | 0.0% | 0.0% | 0.0% | 0.0% |
|  | k\_\_Bacteria;p\_\_Armatimonadetes;c\_\_[Fimbriimonadia];o\_\_[Fimbriimonadales];f\_\_[Fimbriimonadaceae] | 0 | 0.0% | 0.0% | 0.0% | 0.0% | 0.0% | 0.0% | 0.0% | 0.0% | 0.0% | 0.0% | 0.0% | 0.0% | 0.0% | 0.0% | 0.0% | 0.0% | 0.0% | 0.0% | 0.0% | 0.0% | 0.0% | 0.0% | 0.0% | 0.0% |
|  | k\_\_Bacteria;p\_\_Chloroflexi;c\_\_Anaerolineae;o\_\_SBR1031;f\_\_SHA-31 | 0 | 0.0% | 0.0% | 0.0% | 0.1% | 0.1% | 0.0% | 0.0% | 0.0% | 0.0% | 0.0% | 0.0% | 0.0% | 0.0% | 0.0% | 0.0% | 0.1% | 0.0% | 0.0% | 0.0% | 0.0% | 0.0% | 0.0% | 0.0% | 0.0% |
|  | k\_\_Bacteria;p\_\_Proteobacteria;c\_\_Alphaproteobacteria;o\_\_Sphingomonadales;f\_\_Erythrobacteraceae | 0 | 0.0% | 0.0% | 0.0% | 0.0% | 0.0% | 0.0% | 0.0% | 0.0% | 0.0% | 0.0% | 0.0% | 0.0% | 0.0% | 0.0% | 0.0% | 0.0% | 0.1% | 0.1% | 0.0% | 0.0% | 0.0% | 0.0% | 0.0% | 0.0% |
|  | k\_\_Bacteria;p\_\_Actinobacteria;c\_\_Actinobacteria;o\_\_Actinomycetales;f\_\_Dermabacteraceae | 0 | 0.0% | 0.0% | 0.0% | 0.0% | 0.0% | 0.0% | 0.0% | 0.0% | 0.0% | 0.0% | 0.0% | 0.0% | 0.0% | 0.0% | 0.0% | 0.0% | 0.0% | 0.0% | 0.0% | 0.0% | 0.0% | 0.0% | 0.0% | 0.0% |
|  | k\_\_Bacteria;p\_\_Gemmatimonadetes;c\_\_Gemmatimonadetes;o\_\_Gemmatimonadales;f\_\_Ellin5301 | 0 | 0.0% | 0.0% | 0.0% | 0.0% | 0.0% | 0.0% | 0.0% | 0.0% | 0.0% | 0.0% | 0.0% | 0.0% | 0.0% | 0.0% | 0.0% | 0.0% | 0.0% | 0.0% | 0.0% | 0.0% | 0.0% | 0.2% | 0.0% | 0.0% |
|  | k\_\_Bacteria;p\_\_Nitrospirae;c\_\_Nitrospira;o\_\_Nitrospirales;f\_\_0319-6A21 | 0 | 0.0% | 0.0% | 0.0% | 0.0% | 0.0% | 0.0% | 0.0% | 0.0% | 0.0% | 0.0% | 0.0% | 0.0% | 0.0% | 0.0% | 0.1% | 0.0% | 0.0% | 0.0% | 0.0% | 0.0% | 0.0% | 0.1% | 0.0% | 0.0% |
|  | k\_\_Bacteria;p\_\_WPS-2;c\_\_Unclassified\_WPS-2;o\_\_Unclassified\_WPS-2;f\_\_Unclassified\_WPS-2 | 0 | 0.0% | 0.0% | 0.0% | 0.0% | 0.0% | 0.0% | 0.0% | 0.0% | 0.0% | 0.0% | 0.0% | 0.0% | 0.0% | 0.0% | 0.0% | 0.0% | 0.0% | 0.0% | 0.0% | 0.0% | 0.0% | 0.0% | 0.0% | 0.0% |
|  | k\_\_Bacteria;p\_\_Actinobacteria;c\_\_Actinobacteria;o\_\_Actinomycetales;f\_\_Cellulomonadaceae | 0 | 0.0% | 0.0% | 0.0% | 0.0% | 0.0% | 0.0% | 0.0% | 0.0% | 0.0% | 0.0% | 0.0% | 0.0% | 0.0% | 0.0% | 0.0% | 0.0% | 0.1% | 0.0% | 0.0% | 0.0% | 0.0% | 0.1% | 0.0% | 0.0% |
|  | k\_\_Bacteria;p\_\_WS3;c\_\_PRR-12;o\_\_Sediment-1;f\_\_Unclassified\_Sediment-1 | 0 | 0.0% | 0.0% | 0.0% | 0.1% | 0.0% | 0.0% | 0.0% | 0.0% | 0.0% | 0.0% | 0.0% | 0.0% | 0.0% | 0.0% | 0.0% | 0.0% | 0.0% | 0.0% | 0.0% | 0.0% | 0.0% | 0.0% | 0.0% | 0.0% |
|  | k\_\_Bacteria;p\_\_Chloroflexi;c\_\_S085;o\_\_Unclassified\_S085;f\_\_Unclassified\_S085 | 0 | 0.0% | 0.0% | 0.0% | 0.1% | 0.0% | 0.0% | 0.0% | 0.0% | 0.0% | 0.0% | 0.0% | 0.0% | 0.0% | 0.0% | 0.0% | 0.1% | 0.0% | 0.0% | 0.0% | 0.0% | 0.0% | 0.1% | 0.0% | 0.0% |
|  | k\_\_Bacteria;p\_\_Actinobacteria;c\_\_Thermoleophilia;o\_\_Solirubrobacterales;f\_\_Unclassified\_Solirubrobacterales | 0 | 0.0% | 0.0% | 0.0% | 0.0% | 0.1% | 0.0% | 0.0% | 0.0% | 0.0% | 0.0% | 0.0% | 0.0% | 0.0% | 0.0% | 0.0% | 0.0% | 0.0% | 0.0% | 0.0% | 0.0% | 0.0% | 0.0% | 0.0% | 0.0% |
|  | k\_\_Bacteria;p\_\_OD1;c\_\_ZB2;o\_\_Unclassified\_ZB2;f\_\_Unclassified\_ZB2 | 0 | 0.0% | 0.0% | 0.0% | 0.0% | 0.0% | 0.0% | 0.0% | 0.0% | 0.0% | 0.0% | 0.0% | 0.0% | 0.0% | 0.0% | 0.0% | 0.0% | 0.0% | 0.0% | 0.0% | 0.0% | 0.0% | 0.0% | 0.0% | 0.0% |
|  | k\_\_Bacteria;p\_\_Planctomycetes;c\_\_Planctomycetia;o\_\_Gemmatales;f\_\_Isosphaeraceae | 0 | 0.0% | 0.0% | 0.0% | 0.0% | 0.0% | 0.0% | 0.0% | 0.0% | 0.0% | 0.0% | 0.0% | 0.1% | 0.0% | 0.0% | 0.0% | 0.0% | 0.0% | 0.0% | 0.0% | 0.0% | 0.0% | 0.0% | 0.0% | 0.0% |
|  | k\_\_Bacteria;p\_\_Proteobacteria;c\_\_Betaproteobacteria;o\_\_Hydrogenophilales;f\_\_Hydrogenophilaceae | 0 | 0.0% | 0.0% | 0.0% | 0.0% | 0.0% | 0.0% | 0.0% | 0.0% | 0.0% | 0.0% | 0.0% | 0.0% | 0.0% | 0.0% | 0.0% | 0.0% | 0.0% | 0.0% | 0.0% | 0.0% | 0.0% | 0.0% | 0.0% | 0.2% |
|  | k\_\_Bacteria;p\_\_Proteobacteria;c\_\_Alphaproteobacteria;o\_\_Rickettsiales;f\_\_mitochondria | 0 | 0.0% | 0.0% | 0.0% | 0.0% | 0.1% | 0.0% | 0.0% | 0.0% | 0.0% | 0.0% | 0.0% | 0.0% | 0.0% | 0.0% | 0.0% | 0.0% | 0.0% | 0.0% | 0.0% | 0.0% | 0.0% | 0.0% | 0.0% | 0.0% |
|  | k\_\_Bacteria;p\_\_Proteobacteria;c\_\_Deltaproteobacteria;o\_\_GMD14H09;f\_\_Unclassified\_GMD14H09 | 0 | 0.0% | 0.0% | 0.0% | 0.0% | 0.0% | 0.0% | 0.0% | 0.0% | 0.0% | 0.0% | 0.0% | 0.0% | 0.0% | 0.0% | 0.0% | 0.0% | 0.0% | 0.0% | 0.1% | 0.0% | 0.0% | 0.0% | 0.0% | 0.1% |
|  | k\_\_Bacteria;p\_\_Gemmatimonadetes;c\_\_Gemmatimonadetes;o\_\_Gemmatimonadales;f\_\_Unclassified\_Gemmatimonadales | 0 | 0.0% | 0.0% | 0.0% | 0.0% | 0.0% | 0.0% | 0.0% | 0.0% | 0.0% | 0.0% | 0.0% | 0.0% | 0.0% | 0.0% | 0.0% | 0.1% | 0.0% | 0.0% | 0.0% | 0.0% | 0.0% | 0.1% | 0.0% | 0.0% |
|  | k\_\_Bacteria;p\_\_Planctomycetes;c\_\_Planctomycetia;o\_\_Gemmatales;f\_\_Gemmataceae | 0 | 0.0% | 0.0% | 0.0% | 0.1% | 0.0% | 0.0% | 0.0% | 0.0% | 0.0% | 0.0% | 0.0% | 0.0% | 0.0% | 0.0% | 0.0% | 0.0% | 0.0% | 0.0% | 0.0% | 0.0% | 0.0% | 0.0% | 0.0% | 0.0% |
|  | k\_\_Bacteria;p\_\_Chloroflexi;c\_\_Chloroflexi;o\_\_[Roseiflexales];f\_\_[Kouleothrixaceae] | 0 | 0.0% | 0.0% | 0.0% | 0.1% | 0.0% | 0.0% | 0.0% | 0.0% | 0.0% | 0.0% | 0.0% | 0.0% | 0.0% | 0.0% | 0.0% | 0.0% | 0.0% | 0.0% | 0.0% | 0.0% | 0.0% | 0.0% | 0.0% | 0.0% |
|  | k\_\_Bacteria;p\_\_Bacteroidetes;c\_\_Bacteroidia;o\_\_Bacteroidales;f\_\_BS11 | 0 | 0.0% | 0.0% | 0.0% | 0.0% | 0.0% | 0.0% | 0.0% | 0.0% | 0.0% | 0.0% | 0.0% | 0.0% | 0.0% | 0.0% | 0.0% | 0.0% | 0.0% | 0.0% | 0.0% | 0.0% | 0.0% | 0.0% | 0.0% | 0.0% |
|  | k\_\_Bacteria;p\_\_Acidobacteria;c\_\_[Chloracidobacteria];o\_\_RB41;f\_\_Unclassified\_RB41 | 0 | 0.0% | 0.0% | 0.0% | 0.0% | 0.0% | 0.0% | 0.0% | 0.0% | 0.0% | 0.0% | 0.0% | 0.0% | 0.0% | 0.0% | 0.0% | 0.0% | 0.0% | 0.0% | 0.0% | 0.0% | 0.0% | 0.1% | 0.0% | 0.0% |
|  | k\_\_Bacteria;p\_\_[Thermi];c\_\_Deinococci;o\_\_Thermales;f\_\_Thermaceae | 0 | 0.0% | 0.0% | 0.0% | 0.0% | 0.0% | 0.0% | 0.0% | 0.0% | 0.0% | 0.1% | 0.0% | 0.0% | 0.0% | 0.0% | 0.0% | 0.0% | 0.0% | 0.0% | 0.0% | 0.0% | 0.0% | 0.0% | 0.0% | 0.0% |
|  | k\_\_Bacteria;p\_\_Armatimonadetes;c\_\_Chthonomonadetes;o\_\_Chthonomonadales;f\_\_Chthonomonadaceae | 0 | 0.0% | 0.0% | 0.0% | 0.1% | 0.0% | 0.0% | 0.0% | 0.0% | 0.0% | 0.0% | 0.0% | 0.0% | 0.0% | 0.0% | 0.0% | 0.0% | 0.0% | 0.0% | 0.0% | 0.0% | 0.0% | 0.0% | 0.0% | 0.0% |
|  | k\_\_Bacteria;p\_\_Proteobacteria;c\_\_Alphaproteobacteria;o\_\_Rhizobiales;f\_\_Xanthobacteraceae | 0 | 0.0% | 0.0% | 0.0% | 0.0% | 0.0% | 0.0% | 0.0% | 0.0% | 0.0% | 0.0% | 0.0% | 0.0% | 0.0% | 0.0% | 0.0% | 0.0% | 0.0% | 0.0% | 0.0% | 0.0% | 0.0% | 0.0% | 0.0% | 0.0% |
|  | k\_\_Bacteria;p\_\_AD3;c\_\_ABS-6;o\_\_Unclassified\_ABS-6;f\_\_Unclassified\_ABS-6 | 0 | 0.0% | 0.0% | 0.0% | 0.0% | 0.0% | 0.0% | 0.0% | 0.0% | 0.0% | 0.0% | 0.0% | 0.0% | 0.0% | 0.0% | 0.0% | 0.0% | 0.1% | 0.0% | 0.0% | 0.0% | 0.0% | 0.0% | 0.0% | 0.0% |
|  | k\_\_Bacteria;p\_\_Proteobacteria;c\_\_Gammaproteobacteria;o\_\_Thiotrichales;f\_\_Piscirickettsiaceae | 0 | 0.0% | 0.0% | 0.0% | 0.0% | 0.0% | 0.0% | 0.0% | 0.0% | 0.0% | 0.0% | 0.0% | 0.0% | 0.0% | 0.0% | 0.0% | 0.0% | 0.1% | 0.0% | 0.0% | 0.0% | 0.0% | 0.0% | 0.0% | 0.0% |
|  | k\_\_Bacteria;p\_\_Acidobacteria;c\_\_Acidobacteria-6;o\_\_CCU21;f\_\_Unclassified\_CCU21 | 0 | 0.0% | 0.0% | 0.0% | 0.0% | 0.0% | 0.0% | 0.0% | 0.0% | 0.0% | 0.0% | 0.0% | 0.0% | 0.0% | 0.0% | 0.0% | 0.0% | 0.0% | 0.0% | 0.0% | 0.0% | 0.0% | 0.1% | 0.0% | 0.0% |
|  | k\_\_Bacteria;p\_\_Spirochaetes;c\_\_Spirochaetes;o\_\_Sphaerochaetales;f\_\_Sphaerochaetaceae | 0 | 0.0% | 0.0% | 0.0% | 0.0% | 0.0% | 0.0% | 0.1% | 0.0% | 0.0% | 0.0% | 0.0% | 0.0% | 0.0% | 0.0% | 0.0% | 0.0% | 0.0% | 0.0% | 0.0% | 0.0% | 0.0% | 0.0% | 0.0% | 0.0% |
|  | k\_\_Bacteria;p\_\_Tenericutes;c\_\_CK-1C4-19;o\_\_Unclassified\_CK-1C4-19;f\_\_Unclassified\_CK-1C4-19 | 0 | 0.0% | 0.0% | 0.0% | 0.1% | 0.0% | 0.0% | 0.0% | 0.0% | 0.0% | 0.0% | 0.0% | 0.0% | 0.0% | 0.0% | 0.0% | 0.0% | 0.0% | 0.0% | 0.0% | 0.0% | 0.0% | 0.0% | 0.0% | 0.0% |
|  | k\_\_Bacteria;p\_\_Chloroflexi;c\_\_Thermomicrobia;o\_\_JG30-KF-CM45;f\_\_Unclassified\_JG30-KF-CM45 | 0 | 0.0% | 0.0% | 0.0% | 0.0% | 0.0% | 0.0% | 0.0% | 0.0% | 0.0% | 0.0% | 0.0% | 0.0% | 0.0% | 0.0% | 0.0% | 0.0% | 0.0% | 0.0% | 0.0% | 0.0% | 0.0% | 0.1% | 0.0% | 0.0% |
|  | k\_\_Bacteria;p\_\_Nitrospirae;c\_\_Nitrospira;o\_\_Nitrospirales;f\_\_[Thermodesulfovibrionaceae] | 0 | 0.0% | 0.0% | 0.0% | 0.0% | 0.0% | 0.0% | 0.0% | 0.0% | 0.0% | 0.0% | 0.0% | 0.0% | 0.0% | 0.0% | 0.0% | 0.1% | 0.0% | 0.0% | 0.0% | 0.0% | 0.0% | 0.0% | 0.0% | 0.0% |
|  | k\_\_Bacteria;p\_\_Proteobacteria;c\_\_Betaproteobacteria;o\_\_Burkholderiales;f\_\_Burkholderiaceae | 0 | 0.0% | 0.0% | 0.0% | 0.0% | 0.0% | 0.0% | 0.0% | 0.0% | 0.0% | 0.0% | 0.0% | 0.0% | 0.0% | 0.0% | 0.0% | 0.0% | 0.0% | 0.0% | 0.0% | 0.0% | 0.0% | 0.1% | 0.0% | 0.0% |
|  | k\_\_Bacteria;p\_\_Verrucomicrobia;c\_\_Verruco-5;o\_\_WCHB1-41;f\_\_Unclassified\_WCHB1-41 | 0 | 0.0% | 0.0% | 0.0% | 0.0% | 0.0% | 0.0% | 0.0% | 0.0% | 0.0% | 0.0% | 0.0% | 0.0% | 0.0% | 0.0% | 0.0% | 0.0% | 0.1% | 0.0% | 0.0% | 0.0% | 0.0% | 0.0% | 0.0% | 0.0% |
|  | k\_\_Bacteria;p\_\_Proteobacteria;c\_\_Alphaproteobacteria;o\_\_Rickettsiales;f\_\_Unclassified\_Rickettsiales | 0 | 0.0% | 0.0% | 0.0% | 0.0% | 0.0% | 0.0% | 0.0% | 0.0% | 0.0% | 0.0% | 0.0% | 0.0% | 0.0% | 0.0% | 0.0% | 0.0% | 0.1% | 0.0% | 0.0% | 0.0% | 0.0% | 0.0% | 0.0% | 0.0% |
|  | k\_\_Bacteria;p\_\_Proteobacteria;c\_\_Alphaproteobacteria;o\_\_BD7-3;f\_\_Unclassified\_BD7-3 | 0 | 0.0% | 0.0% | 0.0% | 0.0% | 0.0% | 0.0% | 0.0% | 0.0% | 0.0% | 0.0% | 0.0% | 0.0% | 0.0% | 0.0% | 0.0% | 0.0% | 0.0% | 0.0% | 0.0% | 0.0% | 0.0% | 0.0% | 0.0% | 0.0% |
|  | k\_\_Bacteria;p\_\_Deferribacteres;c\_\_Deferribacteres;o\_\_Deferribacterales;f\_\_Deferribacteraceae | 0 | 0.0% | 0.0% | 0.0% | 0.0% | 0.0% | 0.0% | 0.0% | 0.0% | 0.0% | 0.0% | 0.0% | 0.0% | 0.0% | 0.0% | 0.0% | 0.0% | 0.0% | 0.0% | 0.0% | 0.0% | 0.0% | 0.0% | 0.0% | 0.0% |
|  | k\_\_Bacteria;p\_\_Proteobacteria;c\_\_Betaproteobacteria;o\_\_Ellin6067;f\_\_Unclassified\_Ellin6067 | 0 | 0.0% | 0.0% | 0.0% | 0.0% | 0.0% | 0.0% | 0.0% | 0.0% | 0.0% | 0.0% | 0.0% | 0.0% | 0.0% | 0.0% | 0.0% | 0.0% | 0.0% | 0.0% | 0.0% | 0.0% | 0.0% | 0.1% | 0.0% | 0.0% |
|  | k\_\_Bacteria;p\_\_Proteobacteria;c\_\_Deltaproteobacteria;o\_\_NB1-j;f\_\_NB1-i | 0 | 0.0% | 0.0% | 0.0% | 0.0% | 0.0% | 0.0% | 0.0% | 0.0% | 0.0% | 0.0% | 0.0% | 0.0% | 0.0% | 0.0% | 0.0% | 0.0% | 0.1% | 0.0% | 0.0% | 0.0% | 0.0% | 0.0% | 0.0% | 0.0% |
|  | k\_\_Bacteria;p\_\_Actinobacteria;c\_\_Actinobacteria;o\_\_Actinomycetales;f\_\_Actinosynnemataceae | 0 | 0.0% | 0.0% | 0.0% | 0.1% | 0.0% | 0.0% | 0.0% | 0.0% | 0.0% | 0.0% | 0.0% | 0.0% | 0.0% | 0.0% | 0.0% | 0.0% | 0.0% | 0.0% | 0.0% | 0.0% | 0.0% | 0.0% | 0.0% | 0.0% |
|  | k\_\_Bacteria;p\_\_Gemmatimonadetes;c\_\_Gemmatimonadetes;o\_\_Ellin5290;f\_\_Unclassified\_Ellin5290 | 0 | 0.0% | 0.0% | 0.0% | 0.0% | 0.0% | 0.0% | 0.0% | 0.0% | 0.0% | 0.0% | 0.0% | 0.0% | 0.0% | 0.0% | 0.0% | 0.0% | 0.0% | 0.0% | 0.0% | 0.0% | 0.0% | 0.1% | 0.0% | 0.0% |
|  | k\_\_Bacteria;p\_\_Proteobacteria;c\_\_Deltaproteobacteria;o\_\_MBNT15;f\_\_Unclassified\_MBNT15 | 0 | 0.0% | 0.0% | 0.0% | 0.0% | 0.0% | 0.0% | 0.0% | 0.0% | 0.0% | 0.0% | 0.0% | 0.0% | 0.0% | 0.0% | 0.0% | 0.0% | 0.1% | 0.0% | 0.0% | 0.0% | 0.0% | 0.0% | 0.0% | 0.0% |
|  | k\_\_Bacteria;p\_\_Cyanobacteria;c\_\_Chloroplast;o\_\_Stramenopiles;f\_\_Unclassified\_Stramenopiles | 0 | 0.0% | 0.0% | 0.0% | 0.0% | 0.0% | 0.0% | 0.0% | 0.0% | 0.0% | 0.0% | 0.0% | 0.0% | 0.0% | 0.0% | 0.0% | 0.0% | 0.0% | 0.0% | 0.0% | 0.0% | 0.0% | 0.0% | 0.0% | 0.0% |
|  | k\_\_Bacteria;p\_\_Chloroflexi;c\_\_Anaerolineae;o\_\_CFB-26;f\_\_Unclassified\_CFB-26 | 0 | 0.0% | 0.0% | 0.0% | 0.0% | 0.0% | 0.0% | 0.0% | 0.0% | 0.0% | 0.0% | 0.0% | 0.0% | 0.0% | 0.0% | 0.0% | 0.0% | 0.0% | 0.0% | 0.0% | 0.0% | 0.0% | 0.0% | 0.0% | 0.0% |
|  | k\_\_Bacteria;p\_\_Chloroflexi;c\_\_Anaerolineae;o\_\_GCA004;f\_\_Unclassified\_GCA004 | 0 | 0.0% | 0.0% | 0.0% | 0.0% | 0.0% | 0.0% | 0.0% | 0.0% | 0.0% | 0.0% | 0.0% | 0.0% | 0.0% | 0.0% | 0.0% | 0.0% | 0.1% | 0.0% | 0.0% | 0.0% | 0.0% | 0.0% | 0.0% | 0.0% |
|  | k\_\_Bacteria;p\_\_Chloroflexi;c\_\_Anaerolineae;o\_\_SBR1031;f\_\_oc28 | 0 | 0.0% | 0.0% | 0.0% | 0.0% | 0.0% | 0.0% | 0.0% | 0.0% | 0.0% | 0.0% | 0.0% | 0.0% | 0.0% | 0.0% | 0.0% | 0.0% | 0.0% | 0.0% | 0.0% | 0.0% | 0.0% | 0.0% | 0.0% | 0.0% |
|  | k\_\_Bacteria;p\_\_Nitrospirae;c\_\_Nitrospira;o\_\_Nitrospirales;f\_\_Nitrospiraceae | 0 | 0.0% | 0.0% | 0.0% | 0.0% | 0.0% | 0.0% | 0.0% | 0.0% | 0.0% | 0.0% | 0.0% | 0.0% | 0.0% | 0.0% | 0.0% | 0.0% | 0.1% | 0.0% | 0.0% | 0.0% | 0.0% | 0.0% | 0.0% | 0.0% |
|  | k\_\_Bacteria;p\_\_Proteobacteria;c\_\_Gammaproteobacteria;o\_\_Alteromonadales;f\_\_Shewanellaceae | 0 | 0.0% | 0.0% | 0.0% | 0.1% | 0.0% | 0.0% | 0.0% | 0.0% | 0.0% | 0.0% | 0.0% | 0.0% | 0.0% | 0.0% | 0.0% | 0.0% | 0.0% | 0.0% | 0.0% | 0.0% | 0.0% | 0.0% | 0.0% | 0.0% |
|  | k\_\_Bacteria;p\_\_Proteobacteria;c\_\_Epsilonproteobacteria;o\_\_Campylobacterales;f\_\_Helicobacteraceae | 0 | 0.0% | 0.0% | 0.0% | 0.0% | 0.0% | 0.0% | 0.0% | 0.0% | 0.0% | 0.0% | 0.0% | 0.0% | 0.0% | 0.0% | 0.0% | 0.0% | 0.0% | 0.0% | 0.0% | 0.0% | 0.0% | 0.0% | 0.0% | 0.0% |
|  | k\_\_Bacteria;p\_\_Actinobacteria;c\_\_Acidimicrobiia;o\_\_Acidimicrobiales;f\_\_EB1017 | 0 | 0.0% | 0.0% | 0.0% | 0.0% | 0.0% | 0.0% | 0.0% | 0.0% | 0.0% | 0.0% | 0.0% | 0.0% | 0.0% | 0.0% | 0.0% | 0.0% | 0.0% | 0.0% | 0.0% | 0.0% | 0.0% | 0.1% | 0.0% | 0.0% |
|  | k\_\_Bacteria;p\_\_Actinobacteria;c\_\_Actinobacteria;o\_\_Actinomycetales;f\_\_Intrasporangiaceae | 0 | 0.0% | 0.0% | 0.0% | 0.0% | 0.0% | 0.0% | 0.0% | 0.0% | 0.0% | 0.0% | 0.0% | 0.0% | 0.0% | 0.0% | 0.0% | 0.0% | 0.0% | 0.0% | 0.0% | 0.0% | 0.0% | 0.0% | 0.0% | 0.0% |
|  | k\_\_Bacteria;p\_\_Proteobacteria;c\_\_Betaproteobacteria;o\_\_IS-44;f\_\_Unclassified\_IS-44 | 0 | 0.0% | 0.0% | 0.0% | 0.0% | 0.0% | 0.0% | 0.0% | 0.0% | 0.0% | 0.0% | 0.0% | 0.0% | 0.0% | 0.0% | 0.0% | 0.0% | 0.1% | 0.0% | 0.0% | 0.0% | 0.0% | 0.0% | 0.0% | 0.0% |
|  | k\_\_Bacteria;p\_\_Acidobacteria;c\_\_DA052;o\_\_Ellin6513;f\_\_Unclassified\_Ellin6513 | 0 | 0.0% | 0.0% | 0.0% | 0.0% | 0.0% | 0.0% | 0.0% | 0.0% | 0.0% | 0.0% | 0.0% | 0.0% | 0.0% | 0.0% | 0.0% | 0.0% | 0.0% | 0.0% | 0.0% | 0.0% | 0.0% | 0.0% | 0.0% | 0.0% |
|  | k\_\_Bacteria;p\_\_Chloroflexi;c\_\_Ellin6529;o\_\_Unclassified\_Ellin6529;f\_\_Unclassified\_Ellin6529 | 0 | 0.0% | 0.0% | 0.0% | 0.0% | 0.0% | 0.0% | 0.0% | 0.0% | 0.0% | 0.0% | 0.0% | 0.0% | 0.0% | 0.0% | 0.0% | 0.0% | 0.0% | 0.0% | 0.0% | 0.0% | 0.0% | 0.1% | 0.0% | 0.0% |
|  | k\_\_Bacteria;p\_\_Proteobacteria;c\_\_Deltaproteobacteria;o\_\_NB1-j;f\_\_Unclassified\_NB1-j | 0 | 0.0% | 0.0% | 0.0% | 0.0% | 0.0% | 0.0% | 0.0% | 0.0% | 0.0% | 0.0% | 0.0% | 0.0% | 0.0% | 0.0% | 0.0% | 0.0% | 0.0% | 0.0% | 0.0% | 0.0% | 0.0% | 0.0% | 0.0% | 0.0% |
|  | k\_\_Bacteria;p\_\_GAL15;c\_\_Unclassified\_GAL15;o\_\_Unclassified\_GAL15;f\_\_Unclassified\_GAL15 | 0 | 0.0% | 0.0% | 0.0% | 0.0% | 0.0% | 0.0% | 0.0% | 0.0% | 0.0% | 0.0% | 0.0% | 0.0% | 0.0% | 0.0% | 0.0% | 0.0% | 0.1% | 0.0% | 0.0% | 0.0% | 0.0% | 0.0% | 0.0% | 0.0% |
|  | k\_\_Bacteria;p\_\_Acidobacteria;c\_\_Solibacteres;o\_\_Solibacterales;f\_\_Solibacteraceae | 0 | 0.0% | 0.0% | 0.0% | 0.0% | 0.0% | 0.0% | 0.0% | 0.0% | 0.0% | 0.0% | 0.0% | 0.0% | 0.0% | 0.0% | 0.0% | 0.0% | 0.0% | 0.0% | 0.0% | 0.0% | 0.0% | 0.1% | 0.0% | 0.0% |
|  | k\_\_Bacteria;p\_\_Lentisphaerae;c\_\_[Lentisphaeria];o\_\_Z20;f\_\_R4-45B | 0 | 0.0% | 0.0% | 0.0% | 0.0% | 0.0% | 0.0% | 0.0% | 0.0% | 0.0% | 0.0% | 0.0% | 0.0% | 0.0% | 0.0% | 0.0% | 0.0% | 0.0% | 0.0% | 0.0% | 0.0% | 0.0% | 0.1% | 0.0% | 0.0% |
|  | k\_\_Bacteria;p\_\_Proteobacteria;c\_\_Deltaproteobacteria;o\_\_Myxococcales;f\_\_0319-6G20 | 0 | 0.0% | 0.0% | 0.0% | 0.0% | 0.0% | 0.0% | 0.0% | 0.0% | 0.0% | 0.0% | 0.0% | 0.0% | 0.0% | 0.0% | 0.0% | 0.0% | 0.0% | 0.0% | 0.0% | 0.0% | 0.0% | 0.0% | 0.0% | 0.0% |
|  | k\_\_Bacteria;p\_\_Firmicutes;c\_\_Clostridia;o\_\_Clostridiales;f\_\_Eubacteriaceae | 0 | 0.0% | 0.0% | 0.0% | 0.0% | 0.0% | 0.0% | 0.0% | 0.0% | 0.0% | 0.0% | 0.0% | 0.0% | 0.0% | 0.0% | 0.0% | 0.0% | 0.0% | 0.0% | 0.0% | 0.0% | 0.0% | 0.0% | 0.0% | 0.0% |
|  | k\_\_Bacteria;p\_\_Planctomycetes;c\_\_C6;o\_\_MVS-107;f\_\_Unclassified\_MVS-107 | 0 | 0.0% | 0.0% | 0.0% | 0.0% | 0.0% | 0.0% | 0.0% | 0.0% | 0.0% | 0.0% | 0.0% | 0.0% | 0.0% | 0.0% | 0.0% | 0.0% | 0.0% | 0.0% | 0.0% | 0.0% | 0.0% | 0.1% | 0.0% | 0.0% |
|  | k\_\_Bacteria;p\_\_Chlorobi;c\_\_SJA-28;o\_\_Unclassified\_SJA-28;f\_\_Unclassified\_SJA-28 | 0 | 0.0% | 0.0% | 0.0% | 0.0% | 0.0% | 0.0% | 0.0% | 0.0% | 0.0% | 0.0% | 0.0% | 0.0% | 0.0% | 0.0% | 0.0% | 0.0% | 0.0% | 0.0% | 0.0% | 0.0% | 0.0% | 0.0% | 0.0% | 0.0% |
|  | k\_\_Bacteria;p\_\_Proteobacteria;c\_\_Betaproteobacteria;o\_\_Burkholderiales;f\_\_Unclassified\_Burkholderiales | 0 | 0.0% | 0.0% | 0.0% | 0.0% | 0.0% | 0.0% | 0.0% | 0.0% | 0.0% | 0.0% | 0.0% | 0.0% | 0.0% | 0.0% | 0.0% | 0.0% | 0.0% | 0.0% | 0.0% | 0.0% | 0.0% | 0.0% | 0.0% | 0.0% |
|  | k\_\_Bacteria;p\_\_Chloroflexi;c\_\_TK17;o\_\_Unclassified\_TK17;f\_\_Unclassified\_TK17 | 0 | 0.0% | 0.0% | 0.0% | 0.0% | 0.0% | 0.0% | 0.0% | 0.0% | 0.0% | 0.0% | 0.0% | 0.0% | 0.0% | 0.0% | 0.0% | 0.0% | 0.0% | 0.0% | 0.0% | 0.0% | 0.0% | 0.0% | 0.0% | 0.0% |
|  | k\_\_Bacteria;p\_\_Firmicutes;c\_\_Bacilli;o\_\_Lactobacillales;f\_\_Unclassified\_Lactobacillales | 0 | 0.0% | 0.0% | 0.0% | 0.0% | 0.0% | 0.0% | 0.0% | 0.0% | 0.0% | 0.0% | 0.0% | 0.0% | 0.0% | 0.0% | 0.0% | 0.0% | 0.0% | 0.0% | 0.0% | 0.0% | 0.0% | 0.0% | 0.0% | 0.0% |
|  | k\_\_Bacteria;p\_\_Chloroflexi;c\_\_Anaerolineae;o\_\_DRC31;f\_\_Unclassified\_DRC31 | 0 | 0.0% | 0.0% | 0.0% | 0.0% | 0.0% | 0.0% | 0.0% | 0.0% | 0.0% | 0.0% | 0.0% | 0.0% | 0.0% | 0.0% | 0.0% | 0.0% | 0.1% | 0.0% | 0.0% | 0.0% | 0.0% | 0.0% | 0.0% | 0.0% |
|  | k\_\_Bacteria;p\_\_Actinobacteria;c\_\_Actinobacteria;o\_\_Actinomycetales;f\_\_Bogoriellaceae | 0 | 0.0% | 0.0% | 0.0% | 0.0% | 0.0% | 0.0% | 0.0% | 0.0% | 0.0% | 0.0% | 0.0% | 0.0% | 0.0% | 0.0% | 0.0% | 0.0% | 0.0% | 0.0% | 0.0% | 0.0% | 0.0% | 0.0% | 0.0% | 0.0% |
|  | k\_\_Bacteria;p\_\_Bacteroidetes;c\_\_Cytophagia;o\_\_Cytophagales;f\_\_Cyclobacteriaceae | 0 | 0.0% | 0.0% | 0.0% | 0.0% | 0.0% | 0.0% | 0.0% | 0.0% | 0.0% | 0.0% | 0.0% | 0.0% | 0.0% | 0.0% | 0.0% | 0.0% | 0.0% | 0.0% | 0.0% | 0.0% | 0.0% | 0.0% | 0.0% | 0.0% |
|  | k\_\_Bacteria;p\_\_Firmicutes;c\_\_Clostridia;o\_\_Thermoanaerobacterales;f\_\_Thermoanaerobacteraceae | 0 | 0.0% | 0.0% | 0.0% | 0.0% | 0.0% | 0.0% | 0.0% | 0.0% | 0.0% | 0.0% | 0.0% | 0.0% | 0.0% | 0.0% | 0.0% | 0.0% | 0.0% | 0.0% | 0.0% | 0.0% | 0.0% | 0.0% | 0.0% | 0.0% |
|  | k\_\_Bacteria;p\_\_Chloroflexi;c\_\_Ktedonobacteria;o\_\_JG30-KF-AS9;f\_\_Unclassified\_JG30-KF-AS9 | 0 | 0.0% | 0.0% | 0.0% | 0.0% | 0.0% | 0.0% | 0.0% | 0.0% | 0.0% | 0.0% | 0.0% | 0.0% | 0.0% | 0.0% | 0.1% | 0.0% | 0.0% | 0.0% | 0.0% | 0.0% | 0.0% | 0.0% | 0.0% | 0.0% |
|  | k\_\_Bacteria;p\_\_Gemmatimonadetes;c\_\_Gemm-5;o\_\_Unclassified\_Gemm-5;f\_\_Unclassified\_Gemm-5 | 0 | 0.0% | 0.0% | 0.0% | 0.0% | 0.0% | 0.0% | 0.0% | 0.0% | 0.0% | 0.0% | 0.0% | 0.0% | 0.0% | 0.0% | 0.0% | 0.0% | 0.0% | 0.0% | 0.0% | 0.0% | 0.0% | 0.0% | 0.0% | 0.0% |
|  | k\_\_Bacteria;p\_\_Acidobacteria;c\_\_Acidobacteria-6;o\_\_iii1-15;f\_\_mb2424 | 0 | 0.0% | 0.0% | 0.0% | 0.0% | 0.0% | 0.0% | 0.0% | 0.0% | 0.0% | 0.0% | 0.0% | 0.0% | 0.0% | 0.0% | 0.0% | 0.0% | 0.0% | 0.0% | 0.0% | 0.0% | 0.0% | 0.0% | 0.0% | 0.0% |
|  | k\_\_Bacteria;p\_\_Proteobacteria;c\_\_Gammaproteobacteria;o\_\_Alteromonadales;f\_\_[Chromatiaceae] | 0 | 0.0% | 0.0% | 0.0% | 0.0% | 0.0% | 0.0% | 0.0% | 0.0% | 0.0% | 0.0% | 0.0% | 0.0% | 0.0% | 0.0% | 0.0% | 0.0% | 0.0% | 0.0% | 0.0% | 0.0% | 0.0% | 0.0% | 0.0% | 0.0% |
|  | k\_\_Bacteria;p\_\_Proteobacteria;c\_\_Gammaproteobacteria;o\_\_Vibrionales;f\_\_Pseudoalteromonadaceae | 0 | 0.0% | 0.0% | 0.0% | 0.1% | 0.0% | 0.0% | 0.0% | 0.0% | 0.0% | 0.0% | 0.0% | 0.0% | 0.0% | 0.0% | 0.0% | 0.0% | 0.0% | 0.0% | 0.0% | 0.0% | 0.0% | 0.0% | 0.0% | 0.0% |
|  | k\_\_Bacteria;p\_\_Actinobacteria;c\_\_Actinobacteria;o\_\_Actinomycetales;f\_\_Yaniellaceae | 0 | 0.0% | 0.0% | 0.0% | 0.0% | 0.0% | 0.0% | 0.0% | 0.0% | 0.0% | 0.0% | 0.0% | 0.0% | 0.0% | 0.0% | 0.0% | 0.0% | 0.0% | 0.0% | 0.0% | 0.0% | 0.0% | 0.0% | 0.0% | 0.0% |
|  | k\_\_Bacteria;p\_\_Proteobacteria;c\_\_Deltaproteobacteria;o\_\_Desulfuromonadales;f\_\_Geobacteraceae | 0 | 0.0% | 0.0% | 0.0% | 0.0% | 0.0% | 0.0% | 0.0% | 0.0% | 0.0% | 0.0% | 0.0% | 0.0% | 0.0% | 0.0% | 0.0% | 0.0% | 0.0% | 0.0% | 0.0% | 0.0% | 0.0% | 0.0% | 0.0% | 0.0% |
|  | k\_\_Bacteria;p\_\_WS3;c\_\_PRR-12;o\_\_Sediment-1;f\_\_PRR-10 | 0 | 0.0% | 0.0% | 0.0% | 0.0% | 0.0% | 0.0% | 0.0% | 0.0% | 0.0% | 0.0% | 0.0% | 0.0% | 0.0% | 0.0% | 0.0% | 0.0% | 0.1% | 0.0% | 0.0% | 0.0% | 0.0% | 0.0% | 0.0% | 0.0% |
|  | k\_\_Bacteria;p\_\_Firmicutes;c\_\_Bacilli;o\_\_Bacillales;f\_\_[Exiguobacteraceae] | 0 | 0.0% | 0.0% | 0.0% | 0.0% | 0.0% | 0.0% | 0.0% | 0.0% | 0.0% | 0.0% | 0.0% | 0.0% | 0.0% | 0.0% | 0.0% | 0.0% | 0.0% | 0.0% | 0.0% | 0.0% | 0.0% | 0.0% | 0.0% | 0.0% |
|  | k\_\_Bacteria;p\_\_Actinobacteria;c\_\_Acidimicrobiia;o\_\_Acidimicrobiales;f\_\_C111 | 0 | 0.0% | 0.0% | 0.0% | 0.0% | 0.0% | 0.0% | 0.0% | 0.0% | 0.0% | 0.0% | 0.0% | 0.0% | 0.0% | 0.0% | 0.0% | 0.0% | 0.0% | 0.0% | 0.0% | 0.0% | 0.0% | 0.0% | 0.0% | 0.0% |
|  | k\_\_Bacteria;p\_\_Proteobacteria;c\_\_Alphaproteobacteria;o\_\_Rhodospirillales;f\_\_Unclassified\_Rhodospirillales | 0 | 0.0% | 0.0% | 0.0% | 0.0% | 0.0% | 0.0% | 0.0% | 0.0% | 0.0% | 0.0% | 0.0% | 0.0% | 0.0% | 0.0% | 0.0% | 0.0% | 0.0% | 0.0% | 0.0% | 0.0% | 0.0% | 0.0% | 0.0% | 0.0% |
|  | k\_\_Bacteria;p\_\_Bacteroidetes;c\_\_Flavobacteriia;o\_\_Flavobacteriales;f\_\_Cryomorphaceae | 0 | 0.0% | 0.0% | 0.0% | 0.0% | 0.0% | 0.0% | 0.0% | 0.0% | 0.0% | 0.0% | 0.0% | 0.0% | 0.0% | 0.0% | 0.0% | 0.0% | 0.0% | 0.0% | 0.0% | 0.0% | 0.0% | 0.0% | 0.0% | 0.1% |
|  | k\_\_Bacteria;p\_\_Planctomycetes;c\_\_Phycisphaerae;o\_\_Phycisphaerales;f\_\_Phycisphaeraceae | 0 | 0.0% | 0.0% | 0.0% | 0.0% | 0.0% | 0.0% | 0.0% | 0.0% | 0.0% | 0.0% | 0.0% | 0.0% | 0.0% | 0.0% | 0.0% | 0.0% | 0.0% | 0.0% | 0.0% | 0.0% | 0.0% | 0.0% | 0.0% | 0.0% |
|  | k\_\_Bacteria;p\_\_Firmicutes;c\_\_Clostridia;o\_\_Clostridiales;f\_\_Dehalobacteriaceae | 0 | 0.0% | 0.0% | 0.0% | 0.0% | 0.0% | 0.0% | 0.0% | 0.0% | 0.0% | 0.0% | 0.0% | 0.0% | 0.0% | 0.0% | 0.0% | 0.0% | 0.0% | 0.0% | 0.0% | 0.0% | 0.0% | 0.0% | 0.0% | 0.0% |
|  | k\_\_Bacteria;p\_\_Proteobacteria;c\_\_Alphaproteobacteria;o\_\_RF32;f\_\_Unclassified\_RF32 | 0 | 0.0% | 0.0% | 0.0% | 0.0% | 0.0% | 0.0% | 0.0% | 0.0% | 0.0% | 0.0% | 0.0% | 0.0% | 0.0% | 0.0% | 0.0% | 0.0% | 0.0% | 0.0% | 0.0% | 0.0% | 0.0% | 0.0% | 0.0% | 0.0% |
|  | k\_\_Bacteria;p\_\_Proteobacteria;c\_\_Deltaproteobacteria;o\_\_Desulfobacterales;f\_\_Desulfobacteraceae | 0 | 0.0% | 0.0% | 0.0% | 0.0% | 0.0% | 0.0% | 0.0% | 0.0% | 0.0% | 0.0% | 0.0% | 0.0% | 0.0% | 0.0% | 0.0% | 0.0% | 0.1% | 0.0% | 0.0% | 0.0% | 0.0% | 0.0% | 0.0% | 0.0% |
|  | k\_\_Bacteria;p\_\_Verrucomicrobia;c\_\_Opitutae;o\_\_[Cerasicoccales];f\_\_[Cerasicoccaceae] | 0 | 0.0% | 0.0% | 0.0% | 0.0% | 0.0% | 0.0% | 0.0% | 0.0% | 0.0% | 0.0% | 0.0% | 0.0% | 0.0% | 0.0% | 0.0% | 0.0% | 0.0% | 0.0% | 0.0% | 0.0% | 0.0% | 0.0% | 0.0% | 0.0% |
|  | k\_\_Bacteria;p\_\_Firmicutes;c\_\_Bacilli;o\_\_Bacillales;f\_\_Thermoactinomycetaceae | 0 | 0.0% | 0.0% | 0.0% | 0.0% | 0.0% | 0.0% | 0.0% | 0.0% | 0.0% | 0.0% | 0.0% | 0.0% | 0.0% | 0.0% | 0.0% | 0.0% | 0.0% | 0.0% | 0.0% | 0.0% | 0.0% | 0.0% | 0.0% | 0.0% |
|  | k\_\_Bacteria;p\_\_Chloroflexi;c\_\_Anaerolineae;o\_\_Caldilineales;f\_\_Caldilineaceae | 0 | 0.0% | 0.0% | 0.0% | 0.0% | 0.0% | 0.0% | 0.0% | 0.0% | 0.0% | 0.0% | 0.0% | 0.0% | 0.0% | 0.0% | 0.0% | 0.1% | 0.0% | 0.0% | 0.0% | 0.0% | 0.0% | 0.0% | 0.0% | 0.0% |
|  | k\_\_Bacteria;p\_\_Proteobacteria;c\_\_Gammaproteobacteria;o\_\_Unclassified\_Gammaproteobacteria;f\_\_Unclassified\_Gammaproteobacteria | 0 | 0.0% | 0.0% | 0.0% | 0.0% | 0.0% | 0.0% | 0.0% | 0.0% | 0.0% | 0.0% | 0.0% | 0.0% | 0.0% | 0.0% | 0.0% | 0.0% | 0.0% | 0.0% | 0.0% | 0.0% | 0.0% | 0.0% | 0.0% | 0.0% |
|  | k\_\_Bacteria;p\_\_Proteobacteria;c\_\_Deltaproteobacteria;o\_\_Desulfobacterales;f\_\_Desulfobulbaceae | 0 | 0.0% | 0.0% | 0.0% | 0.0% | 0.0% | 0.0% | 0.0% | 0.0% | 0.0% | 0.0% | 0.0% | 0.0% | 0.0% | 0.0% | 0.0% | 0.0% | 0.0% | 0.0% | 0.0% | 0.0% | 0.0% | 0.0% | 0.0% | 0.0% |
|  | k\_\_Bacteria;p\_\_Proteobacteria;c\_\_Deltaproteobacteria;o\_\_Myxococcales;f\_\_OM27 | 0 | 0.0% | 0.0% | 0.0% | 0.0% | 0.0% | 0.0% | 0.0% | 0.0% | 0.0% | 0.0% | 0.0% | 0.0% | 0.0% | 0.0% | 0.0% | 0.0% | 0.0% | 0.0% | 0.0% | 0.0% | 0.0% | 0.0% | 0.0% | 0.0% |
|  | k\_\_Bacteria;p\_\_Proteobacteria;c\_\_Betaproteobacteria;o\_\_Methylophilales;f\_\_Methylophilaceae | 0 | 0.0% | 0.0% | 0.0% | 0.0% | 0.0% | 0.0% | 0.0% | 0.0% | 0.0% | 0.0% | 0.0% | 0.0% | 0.0% | 0.0% | 0.0% | 0.0% | 0.0% | 0.0% | 0.0% | 0.0% | 0.0% | 0.0% | 0.0% | 0.0% |
|  | k\_\_Bacteria;p\_\_Firmicutes;c\_\_Bacilli;o\_\_Lactobacillales;f\_\_Leuconostocaceae | 0 | 0.0% | 0.0% | 0.0% | 0.0% | 0.0% | 0.0% | 0.0% | 0.0% | 0.0% | 0.0% | 0.0% | 0.0% | 0.0% | 0.0% | 0.0% | 0.0% | 0.0% | 0.0% | 0.0% | 0.0% | 0.0% | 0.0% | 0.0% | 0.0% |
|  | k\_\_Bacteria;p\_\_TM7;c\_\_TM7-1;o\_\_Unclassified\_TM7-1;f\_\_Unclassified\_TM7-1 | 0 | 0.0% | 0.0% | 0.0% | 0.0% | 0.0% | 0.0% | 0.0% | 0.0% | 0.0% | 0.0% | 0.0% | 0.0% | 0.0% | 0.0% | 0.0% | 0.0% | 0.0% | 0.0% | 0.0% | 0.0% | 0.0% | 0.0% | 0.0% | 0.0% |
|  | k\_\_Bacteria;p\_\_Proteobacteria;c\_\_Gammaproteobacteria;o\_\_Oceanospirillales;f\_\_Halomonadaceae | 0 | 0.0% | 0.0% | 0.0% | 0.0% | 0.0% | 0.0% | 0.0% | 0.0% | 0.0% | 0.0% | 0.0% | 0.0% | 0.0% | 0.0% | 0.0% | 0.0% | 0.0% | 0.0% | 0.0% | 0.0% | 0.0% | 0.0% | 0.0% | 0.0% |
|  | k\_\_Bacteria;p\_\_Proteobacteria;c\_\_Gammaproteobacteria;o\_\_[Marinicellales];f\_\_[Marinicellaceae] | 0 | 0.0% | 0.0% | 0.0% | 0.0% | 0.0% | 0.0% | 0.0% | 0.0% | 0.0% | 0.0% | 0.0% | 0.0% | 0.0% | 0.0% | 0.0% | 0.0% | 0.0% | 0.0% | 0.0% | 0.0% | 0.0% | 0.0% | 0.0% | 0.0% |
|  | k\_\_Bacteria;p\_\_Actinobacteria;c\_\_Actinobacteria;o\_\_Actinomycetales;f\_\_Sporichthyaceae | 0 | 0.0% | 0.0% | 0.0% | 0.0% | 0.0% | 0.0% | 0.0% | 0.0% | 0.0% | 0.0% | 0.0% | 0.0% | 0.0% | 0.0% | 0.0% | 0.0% | 0.0% | 0.0% | 0.0% | 0.0% | 0.0% | 0.0% | 0.0% | 0.0% |
|  | k\_\_Bacteria;p\_\_Cyanobacteria;c\_\_ML635J-21;o\_\_Unclassified\_ML635J-21;f\_\_Unclassified\_ML635J-21 | 0 | 0.0% | 0.0% | 0.0% | 0.0% | 0.0% | 0.0% | 0.0% | 0.0% | 0.0% | 0.0% | 0.0% | 0.0% | 0.0% | 0.0% | 0.0% | 0.0% | 0.0% | 0.0% | 0.0% | 0.0% | 0.0% | 0.0% | 0.0% | 0.0% |
|  | k\_\_Bacteria;p\_\_Acidobacteria;c\_\_Solibacteres;o\_\_Solibacterales;f\_\_PAUC26f | 0 | 0.0% | 0.0% | 0.0% | 0.0% | 0.0% | 0.0% | 0.0% | 0.0% | 0.0% | 0.0% | 0.0% | 0.0% | 0.0% | 0.0% | 0.0% | 0.0% | 0.0% | 0.0% | 0.0% | 0.0% | 0.0% | 0.0% | 0.0% | 0.0% |
|  | k\_\_Bacteria;p\_\_Actinobacteria;c\_\_Actinobacteria;o\_\_Actinomycetales;f\_\_Nakamurellaceae | 0 | 0.0% | 0.0% | 0.0% | 0.0% | 0.0% | 0.0% | 0.0% | 0.0% | 0.0% | 0.0% | 0.0% | 0.0% | 0.0% | 0.0% | 0.0% | 0.0% | 0.0% | 0.0% | 0.0% | 0.0% | 0.0% | 0.0% | 0.0% | 0.0% |
|  | k\_\_Bacteria;p\_\_Planctomycetes;c\_\_OM190;o\_\_agg27;f\_\_Unclassified\_agg27 | 0 | 0.0% | 0.0% | 0.0% | 0.0% | 0.0% | 0.0% | 0.0% | 0.0% | 0.0% | 0.0% | 0.0% | 0.0% | 0.0% | 0.0% | 0.0% | 0.0% | 0.0% | 0.0% | 0.0% | 0.0% | 0.0% | 0.0% | 0.0% | 0.0% |
|  | k\_\_Bacteria;p\_\_Chloroflexi;c\_\_Anaerolineae;o\_\_S0208;f\_\_Unclassified\_S0208 | 0 | 0.0% | 0.0% | 0.0% | 0.0% | 0.0% | 0.0% | 0.0% | 0.0% | 0.0% | 0.0% | 0.0% | 0.0% | 0.0% | 0.0% | 0.0% | 0.0% | 0.0% | 0.0% | 0.0% | 0.0% | 0.0% | 0.0% | 0.0% | 0.0% |
|  | k\_\_Bacteria;p\_\_Elusimicrobia;c\_\_Elusimicrobia;o\_\_Elusimicrobiales;f\_\_Unclassified\_Elusimicrobiales | 0 | 0.0% | 0.0% | 0.0% | 0.0% | 0.0% | 0.0% | 0.0% | 0.0% | 0.0% | 0.0% | 0.0% | 0.0% | 0.0% | 0.0% | 0.0% | 0.0% | 0.0% | 0.0% | 0.0% | 0.0% | 0.0% | 0.0% | 0.0% | 0.0% |
|  | k\_\_Bacteria;p\_\_Proteobacteria;c\_\_Gammaproteobacteria;o\_\_Thiotrichales;f\_\_Thiotrichaceae | 0 | 0.0% | 0.0% | 0.0% | 0.0% | 0.0% | 0.0% | 0.0% | 0.0% | 0.0% | 0.0% | 0.0% | 0.0% | 0.0% | 0.0% | 0.0% | 0.0% | 0.0% | 0.0% | 0.0% | 0.0% | 0.0% | 0.0% | 0.0% | 0.0% |
|  | k\_\_Bacteria;p\_\_Fibrobacteres;c\_\_Fibrobacteria;o\_\_Fibrobacterales;f\_\_Fibrobacteraceae | 0 | 0.0% | 0.0% | 0.0% | 0.0% | 0.0% | 0.0% | 0.0% | 0.0% | 0.0% | 0.0% | 0.0% | 0.0% | 0.0% | 0.0% | 0.0% | 0.0% | 0.0% | 0.0% | 0.0% | 0.0% | 0.0% | 0.0% | 0.0% | 0.0% |
|  | k\_\_Bacteria;p\_\_Cyanobacteria;c\_\_Oscillatoriophycideae;o\_\_Chroococcales;f\_\_Gomphosphaeriaceae | 0 | 0.0% | 0.0% | 0.0% | 0.0% | 0.0% | 0.0% | 0.0% | 0.0% | 0.0% | 0.0% | 0.0% | 0.0% | 0.0% | 0.0% | 0.0% | 0.0% | 0.0% | 0.0% | 0.0% | 0.0% | 0.0% | 0.0% | 0.0% | 0.0% |
|  | k\_\_Bacteria;p\_\_Acidobacteria;c\_\_RB25;o\_\_Unclassified\_RB25;f\_\_Unclassified\_RB25 | 0 | 0.0% | 0.0% | 0.0% | 0.0% | 0.0% | 0.0% | 0.0% | 0.0% | 0.0% | 0.0% | 0.0% | 0.0% | 0.0% | 0.0% | 0.0% | 0.0% | 0.0% | 0.0% | 0.0% | 0.0% | 0.0% | 0.0% | 0.0% | 0.0% |
|  | k\_\_Bacteria;p\_\_Bacteroidetes;c\_\_Bacteroidia;o\_\_Bacteroidales;f\_\_BA008 | 0 | 0.0% | 0.0% | 0.0% | 0.0% | 0.0% | 0.0% | 0.0% | 0.0% | 0.0% | 0.0% | 0.0% | 0.0% | 0.0% | 0.0% | 0.0% | 0.0% | 0.0% | 0.0% | 0.0% | 0.0% | 0.0% | 0.0% | 0.0% | 0.0% |
|  | k\_\_Bacteria;p\_\_Chloroflexi;c\_\_Chloroflexi;o\_\_AKIW781;f\_\_Unclassified\_AKIW781 | 0 | 0.0% | 0.0% | 0.0% | 0.0% | 0.0% | 0.0% | 0.0% | 0.0% | 0.0% | 0.0% | 0.0% | 0.0% | 0.0% | 0.0% | 0.0% | 0.0% | 0.0% | 0.0% | 0.0% | 0.0% | 0.0% | 0.0% | 0.0% | 0.0% |
|  | k\_\_Bacteria;p\_\_Acidobacteria;c\_\_[Chloracidobacteria];o\_\_DS-100;f\_\_Unclassified\_DS-100 | 0 | 0.0% | 0.0% | 0.0% | 0.0% | 0.0% | 0.0% | 0.0% | 0.0% | 0.0% | 0.0% | 0.0% | 0.0% | 0.0% | 0.0% | 0.0% | 0.0% | 0.0% | 0.0% | 0.0% | 0.0% | 0.0% | 0.0% | 0.0% | 0.0% |
|  | k\_\_Bacteria;p\_\_Bacteroidetes;c\_\_Bacteroidia;o\_\_Bacteroidales;f\_\_[Odoribacteraceae] | 0 | 0.0% | 0.0% | 0.0% | 0.0% | 0.0% | 0.0% | 0.0% | 0.0% | 0.0% | 0.0% | 0.0% | 0.0% | 0.0% | 0.0% | 0.0% | 0.0% | 0.0% | 0.0% | 0.0% | 0.0% | 0.0% | 0.0% | 0.0% | 0.0% |
|  | k\_\_Bacteria;p\_\_Proteobacteria;c\_\_Alphaproteobacteria;o\_\_Rhizobiales;f\_\_Beijerinckiaceae | 0 | 0.0% | 0.0% | 0.0% | 0.0% | 0.0% | 0.0% | 0.0% | 0.0% | 0.0% | 0.0% | 0.0% | 0.0% | 0.0% | 0.0% | 0.0% | 0.0% | 0.0% | 0.0% | 0.0% | 0.0% | 0.0% | 0.0% | 0.0% | 0.0% |
|  | k\_\_Bacteria;p\_\_Actinobacteria;c\_\_Thermoleophilia;o\_\_Solirubrobacterales;f\_\_Solirubrobacteraceae | 0 | 0.0% | 0.0% | 0.0% | 0.0% | 0.0% | 0.0% | 0.0% | 0.0% | 0.0% | 0.0% | 0.0% | 0.0% | 0.0% | 0.0% | 0.0% | 0.0% | 0.0% | 0.0% | 0.0% | 0.0% | 0.0% | 0.0% | 0.0% | 0.0% |
|  | k\_\_Bacteria;p\_\_Chloroflexi;c\_\_Gitt-GS-136;o\_\_Unclassified\_Gitt-GS-136;f\_\_Unclassified\_Gitt-GS-136 | 0 | 0.0% | 0.0% | 0.0% | 0.0% | 0.0% | 0.0% | 0.0% | 0.0% | 0.0% | 0.0% | 0.0% | 0.0% | 0.0% | 0.0% | 0.0% | 0.0% | 0.0% | 0.0% | 0.0% | 0.0% | 0.0% | 0.0% | 0.0% | 0.0% |
|  | k\_\_Bacteria;p\_\_Bacteroidetes;c\_\_Cytophagia;o\_\_Cytophagales;f\_\_Unclassified\_Cytophagales | 0 | 0.0% | 0.0% | 0.0% | 0.0% | 0.0% | 0.0% | 0.0% | 0.0% | 0.0% | 0.0% | 0.0% | 0.0% | 0.0% | 0.0% | 0.0% | 0.0% | 0.0% | 0.0% | 0.0% | 0.0% | 0.0% | 0.0% | 0.0% | 0.0% |
|  | k\_\_Bacteria;p\_\_Elusimicrobia;c\_\_Elusimicrobia;o\_\_Elusimicrobiales;f\_\_Elusimicrobiaceae | 0 | 0.0% | 0.0% | 0.0% | 0.0% | 0.0% | 0.0% | 0.0% | 0.0% | 0.0% | 0.0% | 0.0% | 0.0% | 0.0% | 0.0% | 0.0% | 0.0% | 0.0% | 0.0% | 0.0% | 0.0% | 0.0% | 0.0% | 0.0% | 0.0% |
|  | k\_\_Bacteria;p\_\_Chloroflexi;c\_\_TK10;o\_\_B07\_WMSP1;f\_\_Unclassified\_B07\_WMSP1 | 0 | 0.0% | 0.0% | 0.0% | 0.0% | 0.0% | 0.0% | 0.0% | 0.0% | 0.0% | 0.0% | 0.0% | 0.0% | 0.0% | 0.0% | 0.0% | 0.0% | 0.0% | 0.0% | 0.0% | 0.0% | 0.0% | 0.0% | 0.0% | 0.0% |
|  | k\_\_Bacteria;p\_\_Firmicutes;c\_\_Bacilli;o\_\_Bacillales;f\_\_[Thermicanaceae] | 0 | 0.0% | 0.0% | 0.0% | 0.0% | 0.0% | 0.0% | 0.0% | 0.0% | 0.0% | 0.0% | 0.0% | 0.0% | 0.0% | 0.0% | 0.0% | 0.0% | 0.0% | 0.0% | 0.0% | 0.0% | 0.0% | 0.0% | 0.0% | 0.0% |
|  | k\_\_Bacteria;p\_\_Proteobacteria;c\_\_Gammaproteobacteria;o\_\_HOC36;f\_\_Unclassified\_HOC36 | 0 | 0.0% | 0.0% | 0.0% | 0.0% | 0.0% | 0.0% | 0.0% | 0.0% | 0.0% | 0.0% | 0.0% | 0.0% | 0.0% | 0.0% | 0.0% | 0.0% | 0.0% | 0.0% | 0.0% | 0.0% | 0.0% | 0.0% | 0.0% | 0.0% |
|  | k\_\_Bacteria;p\_\_Acidobacteria;c\_\_Solibacteres;o\_\_Solibacterales;f\_\_AKIW659 | 0 | 0.0% | 0.0% | 0.0% | 0.0% | 0.0% | 0.0% | 0.0% | 0.0% | 0.0% | 0.0% | 0.0% | 0.0% | 0.0% | 0.0% | 0.0% | 0.0% | 0.0% | 0.0% | 0.0% | 0.0% | 0.0% | 0.0% | 0.0% | 0.0% |
|  | k\_\_Bacteria;p\_\_Tenericutes;c\_\_Mollicutes;o\_\_Unclassified\_Mollicutes;f\_\_Unclassified\_Mollicutes | 0 | 0.0% | 0.0% | 0.0% | 0.0% | 0.0% | 0.0% | 0.0% | 0.0% | 0.0% | 0.0% | 0.0% | 0.0% | 0.0% | 0.0% | 0.0% | 0.0% | 0.0% | 0.0% | 0.0% | 0.0% | 0.0% | 0.0% | 0.0% | 0.0% |
|  | k\_\_Bacteria;p\_\_Proteobacteria;c\_\_Alphaproteobacteria;o\_\_Rhizobiales;f\_\_Methylocystaceae | 0 | 0.0% | 0.0% | 0.0% | 0.0% | 0.0% | 0.0% | 0.0% | 0.0% | 0.0% | 0.0% | 0.0% | 0.0% | 0.0% | 0.0% | 0.0% | 0.0% | 0.0% | 0.0% | 0.0% | 0.0% | 0.0% | 0.0% | 0.0% | 0.0% |
|  | k\_\_Bacteria;p\_\_Acidobacteria;c\_\_[Chloracidobacteria];o\_\_RB41;f\_\_Ellin6075 | 0 | 0.0% | 0.0% | 0.0% | 0.0% | 0.0% | 0.0% | 0.0% | 0.0% | 0.0% | 0.0% | 0.0% | 0.0% | 0.0% | 0.0% | 0.0% | 0.0% | 0.0% | 0.0% | 0.0% | 0.0% | 0.0% | 0.0% | 0.0% | 0.0% |
|  | k\_\_Bacteria;p\_\_Proteobacteria;c\_\_Deltaproteobacteria;o\_\_BPC076;f\_\_Unclassified\_BPC076 | 0 | 0.0% | 0.0% | 0.0% | 0.0% | 0.0% | 0.0% | 0.0% | 0.0% | 0.0% | 0.0% | 0.0% | 0.0% | 0.0% | 0.0% | 0.0% | 0.0% | 0.0% | 0.0% | 0.0% | 0.0% | 0.0% | 0.0% | 0.0% | 0.0% |
|  | k\_\_Bacteria;p\_\_Acidobacteria;c\_\_Sva0725;o\_\_Sva0725;f\_\_Unclassified\_Sva0725 | 0 | 0.0% | 0.0% | 0.0% | 0.0% | 0.0% | 0.0% | 0.0% | 0.0% | 0.0% | 0.0% | 0.0% | 0.0% | 0.0% | 0.0% | 0.0% | 0.0% | 0.0% | 0.0% | 0.0% | 0.0% | 0.0% | 0.0% | 0.0% | 0.0% |
|  | k\_\_Bacteria;p\_\_Gemmatimonadetes;c\_\_Gemmatimonadetes;o\_\_C114;f\_\_Unclassified\_C114 | 0 | 0.0% | 0.0% | 0.0% | 0.0% | 0.0% | 0.0% | 0.0% | 0.0% | 0.0% | 0.0% | 0.0% | 0.0% | 0.0% | 0.0% | 0.0% | 0.0% | 0.0% | 0.0% | 0.0% | 0.0% | 0.0% | 0.0% | 0.0% | 0.0% |
|  | k\_\_Bacteria;p\_\_Proteobacteria;c\_\_Gammaproteobacteria;o\_\_Alteromonadales;f\_\_Alteromonadaceae | 0 | 0.0% | 0.0% | 0.0% | 0.0% | 0.0% | 0.0% | 0.0% | 0.0% | 0.0% | 0.0% | 0.0% | 0.0% | 0.0% | 0.0% | 0.0% | 0.0% | 0.0% | 0.0% | 0.0% | 0.0% | 0.0% | 0.0% | 0.0% | 0.0% |
|  | k\_\_Bacteria;p\_\_Acidobacteria;c\_\_BPC102;o\_\_MVS-40;f\_\_Unclassified\_MVS-40 | 0 | 0.0% | 0.0% | 0.0% | 0.0% | 0.0% | 0.0% | 0.0% | 0.0% | 0.0% | 0.0% | 0.0% | 0.0% | 0.0% | 0.0% | 0.0% | 0.0% | 0.0% | 0.0% | 0.0% | 0.0% | 0.0% | 0.0% | 0.0% | 0.0% |
|  | k\_\_Bacteria;p\_\_Chloroflexi;c\_\_Anaerolineae;o\_\_SBR1031;f\_\_SJA-101 | 0 | 0.0% | 0.0% | 0.0% | 0.0% | 0.0% | 0.0% | 0.0% | 0.0% | 0.0% | 0.0% | 0.0% | 0.0% | 0.0% | 0.0% | 0.0% | 0.0% | 0.0% | 0.0% | 0.0% | 0.0% | 0.0% | 0.0% | 0.0% | 0.0% |
|  | k\_\_Bacteria;p\_\_Bacteroidetes;c\_\_Bacteroidia;o\_\_Bacteroidales;f\_\_Marinilabiaceae | 0 | 0.0% | 0.0% | 0.0% | 0.0% | 0.0% | 0.0% | 0.0% | 0.0% | 0.0% | 0.0% | 0.0% | 0.0% | 0.0% | 0.0% | 0.0% | 0.0% | 0.0% | 0.0% | 0.0% | 0.0% | 0.0% | 0.0% | 0.0% | 0.0% |
|  | k\_\_Bacteria;p\_\_Fibrobacteres;c\_\_Fibrobacteria;o\_\_258ds10;f\_\_Unclassified\_258ds10 | 0 | 0.0% | 0.0% | 0.0% | 0.0% | 0.0% | 0.0% | 0.0% | 0.0% | 0.0% | 0.0% | 0.0% | 0.0% | 0.0% | 0.0% | 0.0% | 0.0% | 0.0% | 0.0% | 0.0% | 0.0% | 0.0% | 0.0% | 0.0% | 0.0% |
|  | k\_\_Bacteria;p\_\_Chloroflexi;c\_\_Anaerolineae;o\_\_Anaerolineales;f\_\_Anaerolinaceae | 0 | 0.0% | 0.0% | 0.0% | 0.0% | 0.0% | 0.0% | 0.0% | 0.0% | 0.0% | 0.0% | 0.0% | 0.0% | 0.0% | 0.0% | 0.0% | 0.0% | 0.0% | 0.0% | 0.0% | 0.0% | 0.0% | 0.0% | 0.0% | 0.0% |
|  | k\_\_Bacteria;p\_\_Actinobacteria;c\_\_Thermoleophilia;o\_\_Gaiellales;f\_\_Unclassified\_Gaiellales | 0 | 0.0% | 0.0% | 0.0% | 0.0% | 0.0% | 0.0% | 0.0% | 0.0% | 0.0% | 0.0% | 0.0% | 0.0% | 0.0% | 0.0% | 0.0% | 0.0% | 0.0% | 0.0% | 0.0% | 0.0% | 0.0% | 0.0% | 0.0% | 0.0% |
|  | k\_\_Bacteria;p\_\_Acidobacteria;c\_\_AT-s54;o\_\_Unclassified\_AT-s54;f\_\_Unclassified\_AT-s54 | 0 | 0.0% | 0.0% | 0.0% | 0.0% | 0.0% | 0.0% | 0.0% | 0.0% | 0.0% | 0.0% | 0.0% | 0.0% | 0.0% | 0.0% | 0.0% | 0.0% | 0.0% | 0.0% | 0.0% | 0.0% | 0.0% | 0.0% | 0.0% | 0.0% |
|  | k\_\_Bacteria;p\_\_Bacteroidetes;c\_\_[Saprospirae];o\_\_[Saprospirales];f\_\_Unclassified\_[Saprospirales] | 0 | 0.0% | 0.0% | 0.0% | 0.0% | 0.0% | 0.0% | 0.0% | 0.0% | 0.0% | 0.0% | 0.0% | 0.0% | 0.0% | 0.0% | 0.0% | 0.0% | 0.0% | 0.0% | 0.0% | 0.0% | 0.0% | 0.0% | 0.0% | 0.0% |
|  | k\_\_Bacteria;p\_\_Acidobacteria;c\_\_Acidobacteria-6;o\_\_iii1-15;f\_\_RB40 | 0 | 0.0% | 0.0% | 0.0% | 0.0% | 0.0% | 0.0% | 0.0% | 0.0% | 0.0% | 0.0% | 0.0% | 0.0% | 0.0% | 0.0% | 0.0% | 0.0% | 0.0% | 0.0% | 0.0% | 0.0% | 0.0% | 0.0% | 0.0% | 0.0% |
|  | k\_\_Bacteria;p\_\_Proteobacteria;c\_\_Deltaproteobacteria;o\_\_Myxococcales;f\_\_Cystobacterineae | 0 | 0.0% | 0.0% | 0.0% | 0.0% | 0.0% | 0.0% | 0.0% | 0.0% | 0.0% | 0.0% | 0.0% | 0.0% | 0.0% | 0.0% | 0.0% | 0.0% | 0.0% | 0.0% | 0.0% | 0.0% | 0.0% | 0.0% | 0.0% | 0.0% |
|  | k\_\_Bacteria;p\_\_OD1;c\_\_ABY1;o\_\_Unclassified\_ABY1;f\_\_Unclassified\_ABY1 | 0 | 0.0% | 0.0% | 0.0% | 0.0% | 0.0% | 0.0% | 0.0% | 0.0% | 0.0% | 0.0% | 0.0% | 0.0% | 0.0% | 0.0% | 0.0% | 0.0% | 0.0% | 0.0% | 0.0% | 0.0% | 0.0% | 0.0% | 0.0% | 0.0% |
|  | k\_\_Bacteria;p\_\_Proteobacteria;c\_\_Deltaproteobacteria;o\_\_Spirobacillales;f\_\_Unclassified\_Spirobacillales | 0 | 0.0% | 0.0% | 0.0% | 0.0% | 0.0% | 0.0% | 0.0% | 0.0% | 0.0% | 0.0% | 0.0% | 0.0% | 0.0% | 0.0% | 0.0% | 0.0% | 0.0% | 0.0% | 0.0% | 0.0% | 0.0% | 0.0% | 0.0% | 0.0% |
|  | k\_\_Bacteria;p\_\_Armatimonadetes;c\_\_0319-6E2;o\_\_Unclassified\_0319-6E2;f\_\_Unclassified\_0319-6E2 | 0 | 0.0% | 0.0% | 0.0% | 0.0% | 0.0% | 0.0% | 0.0% | 0.0% | 0.0% | 0.0% | 0.0% | 0.0% | 0.0% | 0.0% | 0.0% | 0.0% | 0.0% | 0.0% | 0.0% | 0.0% | 0.0% | 0.0% | 0.0% | 0.0% |
|  | k\_\_Bacteria;p\_\_Gemmatimonadetes;c\_\_Gemmatimonadetes;o\_\_KD8-87;f\_\_Unclassified\_KD8-87 | 0 | 0.0% | 0.0% | 0.0% | 0.0% | 0.0% | 0.0% | 0.0% | 0.0% | 0.0% | 0.0% | 0.0% | 0.0% | 0.0% | 0.0% | 0.0% | 0.0% | 0.0% | 0.0% | 0.0% | 0.0% | 0.0% | 0.0% | 0.0% | 0.0% |
|  | k\_\_Bacteria;p\_\_Planctomycetes;c\_\_OM190;o\_\_CL500-15;f\_\_Unclassified\_CL500-15 | 0 | 0.0% | 0.0% | 0.0% | 0.0% | 0.0% | 0.0% | 0.0% | 0.0% | 0.0% | 0.0% | 0.0% | 0.0% | 0.0% | 0.0% | 0.0% | 0.0% | 0.0% | 0.0% | 0.0% | 0.0% | 0.0% | 0.0% | 0.0% | 0.0% |

|  |  |
| --- | --- |
|  | |
| Taxonomy Summary. Current Level: Genus | |
| View Figure (.pdf)  View Legend (.pdf) |  |
|  |


|  |
| --- |
| View Table (.txt) |

|  |  |  |  |  |  |  |  |  |  |  |  |  |  |  |  |  |  |  |  |  |  |  |  |  |  |  |
| --- | --- | --- | --- | --- | --- | --- | --- | --- | --- | --- | --- | --- | --- | --- | --- | --- | --- | --- | --- | --- | --- | --- | --- | --- | --- | --- |
|  | | Total | | A01 | A02 | A03 | A04 | A05 | A06 | A07 | A08 | A09 | A10 | A11 | A12 | A13 | B01 | B02 | B03 | B04 | B05 | B06 | B07 | B08 | B09 | B10 |
| Legend | Taxonomy | count | % | % | % | % | % | % | % | % | % | % | % | % | % | % | % | % | % | % | % | % | % | % | % | % |
|  | k\_\_Bacteria;p\_\_Fusobacteria;c\_\_Fusobacteriia;o\_\_Fusobacteriales;f\_\_Leptotrichiaceae;g\_\_Unclassified\_Leptotrichiaceae | 2 | 9.7% | 21.1% | 8.0% | 3.1% | 3.3% | 0.0% | 14.4% | 3.1% | 14.1% | 7.1% | 22.1% | 4.9% | 13.3% | 10.1% | 4.2% | 6.2% | 1.6% | 12.1% | 17.3% | 0.0% | 19.9% | 5.0% | 1.7% | 31.0% |
|  | k\_\_Bacteria;p\_\_Proteobacteria;c\_\_Epsilonproteobacteria;o\_\_Campylobacterales;f\_\_Campylobacteraceae;g\_\_Campylobacter | 2 | 9.3% | 6.8% | 6.5% | 1.6% | 3.7% | 0.0% | 10.6% | 10.9% | 15.1% | 17.9% | 26.8% | 10.7% | 7.5% | 17.2% | 2.6% | 4.4% | 3.2% | 6.2% | 9.0% | 0.1% | 16.1% | 3.0% | 0.5% | 34.1% |
|  | k\_\_Bacteria;p\_\_Proteobacteria;c\_\_Alphaproteobacteria;o\_\_Rhizobiales;f\_\_Brucellaceae;g\_\_Ochrobactrum | 2 | 6.5% | 2.8% | 0.5% | 16.9% | 18.5% | 0.8% | 5.4% | 2.8% | 3.2% | 10.8% | 0.9% | 6.7% | 4.6% | 1.0% | 9.4% | 19.0% | 14.8% | 11.4% | 6.1% | 3.5% | 1.0% | 5.3% | 4.1% | 1.0% |
|  | k\_\_Bacteria;p\_\_Fusobacteria;c\_\_Fusobacteriia;o\_\_Fusobacteriales;f\_\_Fusobacteriaceae;g\_\_Fusobacterium | 1 | 6.5% | 18.9% | 12.3% | 2.2% | 2.1% | 0.0% | 13.9% | 4.3% | 3.6% | 0.5% | 6.3% | 7.6% | 5.1% | 6.6% | 2.7% | 6.5% | 2.4% | 20.3% | 8.1% | 0.1% | 11.6% | 4.8% | 2.9% | 6.6% |
|  | k\_\_Bacteria;p\_\_Firmicutes;c\_\_Clostridia;o\_\_Clostridiales;f\_\_[Tissierellaceae];g\_\_GW-34 | 1 | 5.8% | 0.3% | 18.5% | 5.9% | 1.3% | 23.7% | 0.6% | 12.6% | 3.1% | 2.3% | 1.0% | 1.5% | 2.3% | 4.4% | 10.3% | 0.4% | 0.9% | 0.1% | 0.2% | 39.0% | 3.2% | 0.4% | 0.8% | 0.5% |
|  | k\_\_Bacteria;p\_\_Firmicutes;c\_\_Bacilli;o\_\_Lactobacillales;f\_\_Aerococcaceae;g\_\_Unclassified\_Aerococcaceae | 1 | 5.6% | 0.7% | 6.8% | 9.7% | 4.1% | 18.7% | 3.9% | 4.3% | 12.9% | 7.2% | 3.3% | 9.6% | 14.9% | 5.1% | 6.6% | 0.7% | 0.8% | 1.3% | 1.2% | 13.5% | 1.3% | 0.3% | 0.7% | 0.7% |
|  | k\_\_Bacteria;p\_\_Bacteroidetes;c\_\_Bacteroidia;o\_\_Bacteroidales;f\_\_Porphyromonadaceae;g\_\_Porphyromonas | 1 | 4.5% | 8.4% | 1.5% | 1.0% | 0.6% | 1.6% | 8.3% | 6.7% | 1.9% | 0.1% | 6.1% | 5.7% | 0.9% | 10.2% | 6.6% | 0.7% | 1.4% | 4.5% | 11.4% | 12.0% | 5.3% | 2.9% | 2.8% | 2.0% |
|  | k\_\_Bacteria;p\_\_Firmicutes;c\_\_Bacilli;o\_\_Lactobacillales;f\_\_Aerococcaceae;g\_\_Facklamia | 1 | 4.4% | 14.6% | 4.2% | 6.0% | 14.1% | 2.2% | 12.5% | 4.5% | 2.9% | 3.2% | 3.2% | 6.4% | 3.4% | 1.8% | 3.1% | 4.0% | 0.7% | 1.3% | 3.3% | 2.1% | 2.2% | 0.8% | 2.3% | 3.5% |
|  | k\_\_Bacteria;p\_\_Firmicutes;c\_\_Bacilli;o\_\_Lactobacillales;f\_\_Carnobacteriaceae;g\_\_Unclassified\_Carnobacteriaceae | 1 | 4.0% | 0.1% | 8.6% | 1.7% | 2.7% | 26.7% | 0.3% | 8.0% | 9.2% | 7.2% | 2.7% | 1.8% | 11.4% | 8.1% | 2.0% | 0.1% | 0.1% | 0.4% | 0.4% | 0.0% | 0.0% | 0.0% | 0.0% | 0.0% |
|  | k\_\_Bacteria;p\_\_Proteobacteria;c\_\_Betaproteobacteria;o\_\_Burkholderiales;f\_\_Alcaligenaceae;g\_\_Achromobacter | 1 | 2.8% | 1.2% | 0.2% | 6.1% | 4.9% | 0.3% | 2.8% | 1.1% | 1.5% | 9.0% | 0.4% | 2.5% | 1.5% | 0.5% | 4.3% | 7.0% | 7.4% | 4.1% | 3.1% | 1.2% | 0.4% | 2.4% | 1.9% | 0.5% |
|  | k\_\_Bacteria;p\_\_Actinobacteria;c\_\_Actinobacteria;o\_\_Actinomycetales;f\_\_Corynebacteriaceae;g\_\_Corynebacterium | 1 | 2.7% | 1.2% | 4.9% | 0.8% | 3.2% | 2.7% | 0.6% | 7.3% | 9.1% | 3.9% | 2.8% | 3.2% | 8.7% | 4.7% | 0.6% | 0.2% | 0.4% | 1.1% | 0.3% | 2.1% | 2.1% | 0.3% | 0.9% | 0.7% |
|  | k\_\_Bacteria;p\_\_Proteobacteria;c\_\_Gammaproteobacteria;o\_\_Xanthomonadales;f\_\_Xanthomonadaceae;g\_\_Unclassified\_Xanthomonadaceae | 0 | 1.9% | 0.6% | 0.1% | 3.8% | 2.4% | 0.4% | 1.4% | 0.5% | 0.6% | 2.3% | 0.3% | 1.2% | 0.9% | 0.2% | 2.5% | 9.0% | 6.5% | 5.4% | 1.6% | 1.0% | 0.3% | 1.7% | 1.4% | 0.3% |
|  | k\_\_Bacteria;p\_\_Bacteroidetes;c\_\_[Saprospirae];o\_\_[Saprospirales];f\_\_Chitinophagaceae;g\_\_Sediminibacterium | 0 | 1.9% | 0.7% | 0.1% | 5.0% | 4.9% | 0.3% | 1.7% | 0.7% | 0.6% | 1.6% | 0.3% | 1.7% | 1.0% | 0.3% | 3.3% | 8.7% | 3.9% | 3.5% | 1.6% | 0.8% | 0.2% | 1.4% | 1.1% | 0.3% |
|  | k\_\_Bacteria;p\_\_Firmicutes;c\_\_Clostridia;o\_\_Clostridiales;f\_\_[Tissierellaceae];g\_\_Helcococcus | 0 | 1.7% | 8.8% | 1.2% | 0.8% | 0.1% | 0.3% | 0.5% | 0.2% | 1.1% | 1.1% | 0.9% | 0.3% | 0.2% | 0.8% | 0.2% | 0.1% | 1.6% | 1.1% | 0.0% | 3.6% | 2.0% | 1.0% | 13.5% | 0.7% |
|  | No blast hit;Other;Other;Other;Other;Other | 0 | 1.6% | 0.1% | 8.5% | 0.3% | 0.5% | 0.5% | 0.6% | 0.0% | 0.9% | 0.0% | 0.0% | 0.0% | 0.6% | 6.9% | 1.0% | 1.4% | 0.6% | 4.2% | 0.8% | 0.0% | 8.9% | 0.1% | 0.2% | 0.0% |
|  | k\_\_Bacteria;p\_\_Firmicutes;c\_\_Clostridia;o\_\_Clostridiales;f\_\_[Tissierellaceae];g\_\_Peptoniphilus | 0 | 1.5% | 3.2% | 1.7% | 0.4% | 0.1% | 3.0% | 0.5% | 0.6% | 0.4% | 0.1% | 1.4% | 0.4% | 0.3% | 1.4% | 3.0% | 0.1% | 0.5% | 1.0% | 4.1% | 2.6% | 1.3% | 2.5% | 5.8% | 0.2% |
|  | k\_\_Bacteria;p\_\_Proteobacteria;c\_\_Gammaproteobacteria;o\_\_Pseudomonadales;f\_\_Pseudomonadaceae;g\_\_Unclassified\_Pseudomonadaceae | 0 | 1.4% | 0.0% | 0.0% | 0.4% | 0.3% | 0.0% | 0.4% | 0.9% | 0.5% | 6.6% | 3.1% | 6.4% | 4.2% | 1.4% | 0.1% | 0.5% | 2.0% | 1.5% | 0.2% | 0.1% | 1.8% | 1.3% | 0.2% | 1.3% |
|  | k\_\_Bacteria;p\_\_Firmicutes;c\_\_Clostridia;o\_\_Clostridiales;f\_\_[Tissierellaceae];g\_\_1-68 | 0 | 1.3% | 0.4% | 0.0% | 0.0% | 0.0% | 2.8% | 0.1% | 7.6% | 2.0% | 0.0% | 1.1% | 1.8% | 0.7% | 4.2% | 4.9% | 0.0% | 1.1% | 0.6% | 0.1% | 0.0% | 2.2% | 0.0% | 0.4% | 0.0% |
|  | k\_\_Bacteria;p\_\_Firmicutes;c\_\_Clostridia;o\_\_Clostridiales;f\_\_[Tissierellaceae];g\_\_Unclassified\_[Tissierellaceae] | 0 | 1.3% | 0.8% | 5.6% | 0.6% | 0.0% | 3.3% | 0.9% | 1.8% | 1.3% | 0.8% | 0.6% | 0.6% | 1.0% | 0.8% | 1.1% | 0.3% | 0.2% | 0.6% | 0.9% | 6.0% | 0.9% | 0.2% | 0.2% | 0.8% |
|  | k\_\_Bacteria;p\_\_Firmicutes;c\_\_Clostridia;o\_\_Clostridiales;f\_\_Clostridiaceae;g\_\_Clostridium | 0 | 1.2% | 0.0% | 1.4% | 0.6% | 2.4% | 0.4% | 3.0% | 3.4% | 1.0% | 0.4% | 0.7% | 0.5% | 0.6% | 1.5% | 0.4% | 0.6% | 0.4% | 0.5% | 3.7% | 0.0% | 1.0% | 0.3% | 2.8% | 2.6% |
|  | k\_\_Bacteria;p\_\_Proteobacteria;c\_\_Gammaproteobacteria;o\_\_Pseudomonadales;f\_\_Moraxellaceae;g\_\_Acinetobacter | 0 | 1.1% | 0.3% | 0.0% | 3.6% | 1.6% | 2.7% | 1.0% | 0.5% | 0.4% | 3.5% | 0.4% | 0.9% | 0.3% | 0.1% | 2.9% | 0.7% | 1.1% | 0.9% | 1.3% | 0.3% | 0.2% | 1.4% | 0.6% | 0.6% |
|  | k\_\_Bacteria;p\_\_Firmicutes;c\_\_Clostridia;o\_\_Clostridiales;f\_\_Unclassified\_Clostridiales;g\_\_Unclassified\_Clostridiales | 0 | 1.0% | 0.2% | 0.6% | 0.7% | 1.5% | 0.0% | 1.2% | 0.3% | 1.2% | 0.1% | 0.8% | 0.6% | 0.6% | 0.0% | 0.8% | 0.6% | 3.1% | 0.0% | 2.4% | 0.0% | 2.2% | 3.9% | 0.5% | 1.2% |
|  | k\_\_Bacteria;p\_\_Firmicutes;c\_\_Clostridia;o\_\_Clostridiales;f\_\_[Tissierellaceae];g\_\_Anaerococcus | 0 | 1.0% | 3.0% | 1.3% | 0.5% | 0.3% | 0.0% | 1.0% | 0.4% | 1.8% | 0.1% | 2.2% | 4.6% | 0.6% | 1.9% | 1.0% | 0.2% | 0.3% | 1.0% | 0.4% | 0.6% | 0.6% | 0.0% | 0.5% | 0.0% |
|  | k\_\_Bacteria;p\_\_Firmicutes;c\_\_Clostridia;o\_\_Clostridiales;f\_\_Ruminococcaceae;g\_\_Unclassified\_Ruminococcaceae | 0 | 0.9% | 0.0% | 0.0% | 0.5% | 0.3% | 0.0% | 0.1% | 0.2% | 0.9% | 0.1% | 0.5% | 1.0% | 1.0% | 0.0% | 0.8% | 0.2% | 2.1% | 0.1% | 0.1% | 0.0% | 2.1% | 10.3% | 1.3% | 0.0% |
|  | k\_\_Bacteria;p\_\_Firmicutes;c\_\_Clostridia;o\_\_Clostridiales;f\_\_[Acidaminobacteraceae];g\_\_Fusibacter | 0 | 0.9% | 0.0% | 0.7% | 1.7% | 1.8% | 0.0% | 1.0% | 5.4% | 0.0% | 0.1% | 3.9% | 0.4% | 0.0% | 1.8% | 0.0% | 0.6% | 1.5% | 0.7% | 1.5% | 0.0% | 0.1% | 0.0% | 0.0% | 0.0% |
|  | k\_\_Bacteria;p\_\_Proteobacteria;c\_\_Gammaproteobacteria;o\_\_Enterobacteriales;f\_\_Enterobacteriaceae;g\_\_Unclassified\_Enterobacteriaceae | 0 | 0.9% | 0.2% | 0.0% | 0.5% | 1.5% | 0.0% | 0.0% | 0.0% | 2.1% | 0.0% | 0.0% | 0.0% | 1.8% | 0.0% | 3.6% | 0.1% | 1.5% | 0.0% | 0.2% | 0.5% | 0.1% | 6.2% | 1.7% | 0.0% |
|  | k\_\_Bacteria;p\_\_Proteobacteria;c\_\_Alphaproteobacteria;o\_\_Sphingomonadales;f\_\_Sphingomonadaceae;g\_\_Sphingomonas | 0 | 0.8% | 0.2% | 0.1% | 1.9% | 1.6% | 0.1% | 0.8% | 0.3% | 0.4% | 0.8% | 0.2% | 1.6% | 0.6% | 0.2% | 1.9% | 2.5% | 2.3% | 1.3% | 0.6% | 0.5% | 0.2% | 0.7% | 0.7% | 0.2% |
|  | k\_\_Bacteria;p\_\_Firmicutes;c\_\_Clostridia;o\_\_Clostridiales;f\_\_[Tissierellaceae];g\_\_ph2 | 0 | 0.8% | 0.0% | 2.3% | 0.7% | 0.2% | 2.2% | 0.4% | 1.9% | 1.5% | 0.3% | 0.3% | 0.1% | 0.8% | 1.1% | 1.5% | 0.0% | 0.0% | 0.1% | 0.1% | 3.0% | 0.5% | 0.0% | 0.0% | 0.1% |
|  | k\_\_Bacteria;p\_\_Proteobacteria;c\_\_Betaproteobacteria;o\_\_Burkholderiales;f\_\_Comamonadaceae;g\_\_Unclassified\_Comamonadaceae | 0 | 0.7% | 0.1% | 0.0% | 1.3% | 0.9% | 0.1% | 0.5% | 0.2% | 0.2% | 0.4% | 0.1% | 0.8% | 0.6% | 0.1% | 1.7% | 1.5% | 1.2% | 0.8% | 0.3% | 0.4% | 0.2% | 2.7% | 0.9% | 0.5% |
|  | k\_\_Bacteria;p\_\_Actinobacteria;c\_\_Actinobacteria;o\_\_Actinomycetales;f\_\_Micrococcaceae;g\_\_Unclassified\_Micrococcaceae | 0 | 0.6% | 0.1% | 0.3% | 0.5% | 4.6% | 0.2% | 0.4% | 0.0% | 0.0% | 0.0% | 0.0% | 0.1% | 0.0% | 0.0% | 0.0% | 0.3% | 0.2% | 0.0% | 0.3% | 0.0% | 0.0% | 0.1% | 3.5% | 3.5% |
|  | k\_\_Bacteria;p\_\_Proteobacteria;c\_\_Alphaproteobacteria;o\_\_Caulobacterales;f\_\_Caulobacteraceae;g\_\_Unclassified\_Caulobacteraceae | 0 | 0.6% | 0.2% | 0.1% | 1.4% | 1.2% | 0.1% | 0.5% | 0.3% | 0.2% | 0.3% | 0.1% | 0.6% | 0.4% | 0.1% | 1.1% | 1.9% | 1.5% | 1.0% | 0.5% | 0.3% | 0.1% | 0.5% | 0.5% | 0.1% |
|  | k\_\_Bacteria;p\_\_Proteobacteria;c\_\_Alphaproteobacteria;o\_\_Rhizobiales;f\_\_Methylobacteriaceae;g\_\_Unclassified\_Methylobacteriaceae | 0 | 0.6% | 0.2% | 0.0% | 1.4% | 1.3% | 1.6% | 0.7% | 0.2% | 0.2% | 0.4% | 0.1% | 0.9% | 0.4% | 0.1% | 0.6% | 1.3% | 1.7% | 0.4% | 0.2% | 0.1% | 0.1% | 0.5% | 0.3% | 0.1% |
|  | k\_\_Bacteria;p\_\_Actinobacteria;c\_\_Actinobacteria;o\_\_Actinomycetales;f\_\_Actinomycetaceae;g\_\_Trueperella | 0 | 0.5% | 0.0% | 2.2% | 0.1% | 0.0% | 0.9% | 0.0% | 2.8% | 0.0% | 0.0% | 0.0% | 1.2% | 0.0% | 0.3% | 0.2% | 0.0% | 0.2% | 0.2% | 0.2% | 1.1% | 0.3% | 0.8% | 1.7% | 0.2% |
|  | k\_\_Bacteria;p\_\_Actinobacteria;c\_\_Actinobacteria;o\_\_Actinomycetales;f\_\_Pseudonocardiaceae;g\_\_Amycolatopsis | 0 | 0.5% | 0.2% | 0.0% | 0.6% | 0.9% | 0.1% | 0.3% | 0.2% | 0.3% | 1.1% | 0.1% | 0.5% | 0.2% | 0.1% | 0.7% | 1.1% | 1.9% | 0.8% | 0.4% | 0.3% | 0.1% | 0.5% | 0.3% | 0.1% |
|  | k\_\_Bacteria;p\_\_Cyanobacteria;c\_\_4C0d-2;o\_\_MLE1-12;f\_\_Unclassified\_MLE1-12;g\_\_Unclassified\_MLE1-12 | 0 | 0.5% | 0.1% | 0.0% | 0.9% | 1.0% | 0.1% | 0.5% | 0.2% | 0.2% | 0.4% | 0.1% | 0.4% | 0.3% | 0.1% | 0.7% | 1.2% | 1.9% | 0.7% | 0.5% | 0.3% | 0.1% | 0.4% | 0.2% | 0.1% |
|  | k\_\_Bacteria;p\_\_Proteobacteria;c\_\_Betaproteobacteria;o\_\_Burkholderiales;f\_\_Oxalobacteraceae;g\_\_Cupriavidus | 0 | 0.4% | 0.1% | 0.0% | 1.2% | 0.3% | 0.0% | 0.3% | 0.1% | 0.1% | 0.1% | 0.1% | 0.6% | 0.3% | 0.1% | 0.8% | 1.3% | 1.2% | 0.7% | 0.3% | 0.2% | 0.0% | 0.5% | 0.4% | 0.2% |
|  | k\_\_Bacteria;p\_\_Actinobacteria;c\_\_Actinobacteria;o\_\_Actinomycetales;f\_\_Actinomycetaceae;g\_\_Mobiluncus | 0 | 0.4% | 0.0% | 0.1% | 0.1% | 0.1% | 0.0% | 0.4% | 0.6% | 0.0% | 0.0% | 0.4% | 0.7% | 0.0% | 0.4% | 0.0% | 0.3% | 0.0% | 0.0% | 4.1% | 0.0% | 0.2% | 0.0% | 0.0% | 1.1% |
|  | k\_\_Bacteria;p\_\_Proteobacteria;c\_\_Gammaproteobacteria;o\_\_Pseudomonadales;f\_\_Moraxellaceae;g\_\_Moraxella | 0 | 0.4% | 0.0% | 0.0% | 0.0% | 0.0% | 0.0% | 0.0% | 0.0% | 0.0% | 0.0% | 0.0% | 0.0% | 0.0% | 0.0% | 0.0% | 0.0% | 0.1% | 0.0% | 0.0% | 0.0% | 0.5% | 0.3% | 7.5% | 0.0% |
|  | k\_\_Bacteria;p\_\_Actinobacteria;c\_\_Actinobacteria;o\_\_Actinomycetales;f\_\_Actinomycetaceae;g\_\_Arcanobacterium | 0 | 0.3% | 0.2% | 0.1% | 0.6% | 0.1% | 0.0% | 1.5% | 0.1% | 0.0% | 0.7% | 1.9% | 0.0% | 0.0% | 1.2% | 0.0% | 0.3% | 0.2% | 0.5% | 0.6% | 0.0% | 0.1% | 0.0% | 0.0% | 0.0% |
|  | k\_\_Bacteria;p\_\_Proteobacteria;c\_\_Betaproteobacteria;o\_\_Burkholderiales;f\_\_Comamonadaceae;g\_\_Delftia | 0 | 0.3% | 0.1% | 0.0% | 0.9% | 0.7% | 0.0% | 0.4% | 0.1% | 0.1% | 0.3% | 0.1% | 0.3% | 0.2% | 0.0% | 0.5% | 0.8% | 1.0% | 0.4% | 0.2% | 0.1% | 0.1% | 0.2% | 0.4% | 0.1% |
|  | k\_\_Bacteria;p\_\_Firmicutes;c\_\_Clostridia;o\_\_Clostridiales;f\_\_Lachnospiraceae;g\_\_Unclassified\_Lachnospiraceae | 0 | 0.3% | 0.0% | 0.0% | 0.3% | 0.2% | 0.0% | 0.0% | 0.1% | 0.2% | 0.0% | 0.1% | 0.1% | 0.1% | 0.0% | 0.4% | 0.1% | 3.0% | 0.1% | 0.1% | 0.0% | 0.4% | 1.2% | 0.6% | 0.0% |
|  | k\_\_Bacteria;p\_\_Actinobacteria;c\_\_Actinobacteria;o\_\_Bifidobacteriales;f\_\_Bifidobacteriaceae;g\_\_Bifidobacterium | 0 | 0.3% | 0.0% | 0.0% | 0.0% | 0.0% | 0.1% | 0.0% | 0.0% | 0.0% | 0.0% | 0.0% | 0.1% | 0.0% | 0.0% | 0.1% | 0.0% | 0.0% | 0.0% | 0.0% | 0.0% | 0.0% | 0.1% | 6.2% | 0.0% |
|  | k\_\_Bacteria;p\_\_Firmicutes;c\_\_Bacilli;o\_\_Lactobacillales;f\_\_Streptococcaceae;g\_\_Streptococcus | 0 | 0.3% | 0.7% | 0.0% | 0.1% | 0.0% | 0.0% | 0.0% | 0.0% | 0.0% | 0.1% | 0.0% | 0.1% | 0.0% | 0.0% | 0.1% | 0.1% | 0.0% | 0.3% | 0.0% | 0.0% | 0.1% | 4.4% | 0.4% | 0.0% |
|  | k\_\_Bacteria;p\_\_Firmicutes;c\_\_Bacilli;o\_\_Lactobacillales;f\_\_Aerococcaceae;g\_\_Aerococcus | 0 | 0.3% | 0.1% | 0.0% | 0.1% | 0.0% | 0.0% | 0.0% | 0.6% | 0.3% | 1.1% | 0.4% | 0.9% | 2.0% | 0.2% | 0.1% | 0.0% | 0.0% | 0.0% | 0.0% | 0.0% | 0.0% | 0.0% | 0.2% | 0.0% |
|  | k\_\_Bacteria;p\_\_Actinobacteria;c\_\_Actinobacteria;o\_\_Actinomycetales;f\_\_Microbacteriaceae;g\_\_Cryocola | 0 | 0.2% | 0.1% | 0.0% | 0.2% | 0.3% | 0.0% | 0.1% | 0.1% | 0.1% | 0.6% | 0.0% | 0.2% | 0.1% | 0.0% | 0.2% | 1.4% | 0.6% | 0.5% | 0.2% | 0.1% | 0.0% | 0.2% | 0.1% | 0.0% |
|  | k\_\_Bacteria;p\_\_Fusobacteria;c\_\_Fusobacteriia;o\_\_Fusobacteriales;f\_\_Fusobacteriaceae;g\_\_Cetobacterium | 0 | 0.2% | 0.0% | 0.0% | 0.7% | 0.1% | 0.0% | 0.1% | 0.0% | 0.1% | 0.0% | 0.0% | 0.2% | 0.0% | 0.0% | 0.1% | 0.1% | 0.3% | 0.1% | 0.1% | 0.0% | 0.0% | 0.1% | 2.8% | 0.0% |
|  | k\_\_Bacteria;p\_\_Proteobacteria;c\_\_Betaproteobacteria;o\_\_Neisseriales;f\_\_Neisseriaceae;g\_\_Unclassified\_Neisseriaceae | 0 | 0.2% | 0.0% | 0.0% | 0.0% | 0.1% | 0.0% | 0.0% | 0.0% | 0.0% | 0.0% | 0.0% | 0.0% | 0.0% | 0.0% | 0.0% | 0.5% | 0.0% | 0.0% | 3.4% | 0.8% | 0.0% | 0.0% | 0.0% | 0.0% |
|  | k\_\_Bacteria;p\_\_Firmicutes;c\_\_Bacilli;o\_\_Lactobacillales;f\_\_Lactobacillaceae;g\_\_Lactobacillus | 0 | 0.2% | 0.0% | 0.0% | 0.1% | 0.2% | 0.0% | 0.0% | 0.0% | 0.0% | 0.0% | 0.0% | 0.1% | 0.0% | 0.0% | 0.1% | 0.2% | 0.5% | 0.1% | 0.0% | 0.0% | 0.1% | 0.1% | 3.3% | 0.0% |
|  | k\_\_Bacteria;p\_\_Actinobacteria;c\_\_Actinobacteria;o\_\_Actinomycetales;f\_\_Nocardiaceae;g\_\_Rhodococcus | 0 | 0.2% | 0.0% | 0.0% | 0.2% | 0.4% | 0.0% | 0.7% | 0.1% | 0.1% | 0.3% | 0.1% | 0.2% | 0.2% | 0.1% | 0.1% | 0.6% | 0.8% | 0.5% | 0.1% | 0.2% | 0.0% | 0.0% | 0.0% | 0.0% |
|  | k\_\_Bacteria;p\_\_Proteobacteria;c\_\_Alphaproteobacteria;o\_\_Rhizobiales;f\_\_Methylobacteriaceae;g\_\_Methylobacterium | 0 | 0.2% | 0.1% | 0.0% | 0.4% | 0.4% | 0.0% | 0.2% | 0.1% | 0.1% | 0.7% | 0.0% | 0.2% | 0.2% | 0.0% | 0.2% | 0.6% | 0.4% | 0.3% | 0.2% | 0.2% | 0.0% | 0.2% | 0.2% | 0.0% |
|  | k\_\_Bacteria;p\_\_Proteobacteria;c\_\_Betaproteobacteria;o\_\_Burkholderiales;f\_\_Comamonadaceae;g\_\_Ramlibacter | 0 | 0.2% | 0.0% | 0.0% | 0.0% | 0.0% | 0.0% | 0.0% | 0.4% | 0.2% | 0.9% | 0.1% | 1.1% | 0.6% | 0.3% | 0.0% | 0.0% | 0.0% | 0.0% | 0.0% | 0.0% | 0.1% | 0.8% | 0.1% | 0.2% |
|  | k\_\_Bacteria;p\_\_Firmicutes;c\_\_Bacilli;o\_\_Lactobacillales;f\_\_Aerococcaceae;g\_\_Alloiococcus | 0 | 0.2% | 0.0% | 0.0% | 0.2% | 0.1% | 0.0% | 0.1% | 0.2% | 0.6% | 0.1% | 0.5% | 0.5% | 0.4% | 1.8% | 0.1% | 0.1% | 0.0% | 0.0% | 0.0% | 0.0% | 0.0% | 0.0% | 0.0% | 0.0% |
|  | k\_\_Bacteria;p\_\_Actinobacteria;c\_\_Coriobacteriia;o\_\_Coriobacteriales;f\_\_Coriobacteriaceae;g\_\_Collinsella | 0 | 0.2% | 0.0% | 0.0% | 0.0% | 0.0% | 0.0% | 0.0% | 0.0% | 0.0% | 0.0% | 0.0% | 0.0% | 0.0% | 0.0% | 0.0% | 0.0% | 0.1% | 0.0% | 0.0% | 0.0% | 0.0% | 0.0% | 4.1% | 0.0% |
|  | k\_\_Bacteria;p\_\_Proteobacteria;c\_\_Betaproteobacteria;o\_\_Rhodocyclales;f\_\_Rhodocyclaceae;g\_\_Azospira | 0 | 0.2% | 0.1% | 0.0% | 0.4% | 0.3% | 0.0% | 0.1% | 0.1% | 0.1% | 0.1% | 0.0% | 0.2% | 0.1% | 0.0% | 0.3% | 0.5% | 0.9% | 0.3% | 0.2% | 0.1% | 0.1% | 0.2% | 0.1% | 0.1% |
|  | k\_\_Bacteria;p\_\_Proteobacteria;c\_\_Alphaproteobacteria;o\_\_Rhizobiales;f\_\_Unclassified\_Rhizobiales;g\_\_Unclassified\_Rhizobiales | 0 | 0.2% | 0.0% | 0.0% | 0.3% | 0.4% | 0.0% | 0.2% | 0.1% | 0.1% | 0.3% | 0.0% | 0.2% | 0.1% | 0.0% | 0.3% | 0.5% | 0.5% | 0.4% | 0.2% | 0.1% | 0.1% | 0.1% | 0.1% | 0.0% |
|  | k\_\_Bacteria;p\_\_Proteobacteria;c\_\_Alphaproteobacteria;o\_\_Rhizobiales;f\_\_Rhizobiaceae;g\_\_Agrobacterium | 0 | 0.2% | 0.0% | 0.0% | 0.1% | 0.1% | 0.0% | 0.1% | 0.3% | 0.0% | 0.5% | 0.1% | 1.3% | 0.3% | 0.2% | 0.0% | 0.2% | 0.0% | 0.1% | 0.1% | 0.0% | 0.1% | 0.3% | 0.1% | 0.0% |
|  | k\_\_Bacteria;p\_\_Bacteroidetes;c\_\_Bacteroidia;o\_\_Bacteroidales;f\_\_Unclassified\_Bacteroidales;g\_\_Unclassified\_Bacteroidales | 0 | 0.2% | 0.0% | 0.0% | 0.5% | 0.0% | 0.0% | 0.3% | 0.0% | 0.2% | 0.0% | 0.1% | 0.0% | 0.0% | 0.0% | 0.0% | 0.0% | 0.0% | 0.0% | 0.2% | 0.0% | 0.3% | 1.9% | 0.2% | 0.0% |
|  | k\_\_Bacteria;p\_\_Proteobacteria;c\_\_Alphaproteobacteria;o\_\_Rhizobiales;f\_\_Phyllobacteriaceae;g\_\_Aminobacter | 0 | 0.2% | 0.0% | 0.0% | 0.5% | 0.3% | 0.0% | 0.1% | 0.0% | 0.1% | 0.3% | 0.0% | 0.2% | 0.1% | 0.0% | 0.2% | 0.6% | 0.3% | 0.5% | 0.2% | 0.1% | 0.1% | 0.1% | 0.1% | 0.0% |
|  | k\_\_Bacteria;p\_\_Proteobacteria;c\_\_Betaproteobacteria;o\_\_Rhodocyclales;f\_\_Rhodocyclaceae;g\_\_Zoogloea | 0 | 0.2% | 0.1% | 0.0% | 0.3% | 0.3% | 0.0% | 0.2% | 0.1% | 0.1% | 0.1% | 0.0% | 0.2% | 0.1% | 0.0% | 0.2% | 0.7% | 0.6% | 0.2% | 0.1% | 0.1% | 0.0% | 0.2% | 0.1% | 0.0% |
|  | k\_\_Bacteria;p\_\_Firmicutes;c\_\_Clostridia;o\_\_Clostridiales;f\_\_Peptostreptococcaceae;g\_\_Unclassified\_Peptostreptococcaceae | 0 | 0.2% | 0.0% | 0.0% | 0.1% | 0.1% | 0.0% | 0.0% | 0.1% | 0.1% | 0.1% | 0.1% | 0.2% | 0.2% | 0.0% | 0.1% | 0.1% | 0.1% | 0.0% | 0.0% | 0.0% | 0.1% | 0.3% | 1.4% | 0.8% |
|  | k\_\_Bacteria;p\_\_Proteobacteria;c\_\_Gammaproteobacteria;o\_\_Pasteurellales;f\_\_Pasteurellaceae;g\_\_Haemophilus | 0 | 0.2% | 0.0% | 0.0% | 0.0% | 0.0% | 0.0% | 0.0% | 0.0% | 0.0% | 0.0% | 0.0% | 0.0% | 0.0% | 0.0% | 0.0% | 0.0% | 0.0% | 0.0% | 0.0% | 0.1% | 0.1% | 3.0% | 0.3% | 0.0% |
|  | k\_\_Bacteria;p\_\_Proteobacteria;c\_\_Betaproteobacteria;o\_\_Burkholderiales;f\_\_Oxalobacteraceae;g\_\_Herbaspirillum | 0 | 0.2% | 0.0% | 0.0% | 0.3% | 0.2% | 0.0% | 0.2% | 0.1% | 0.1% | 0.2% | 0.0% | 0.1% | 0.2% | 0.0% | 0.3% | 0.2% | 0.6% | 0.1% | 0.2% | 0.2% | 0.0% | 0.3% | 0.1% | 0.0% |
|  | k\_\_Bacteria;p\_\_Actinobacteria;c\_\_Actinobacteria;o\_\_Actinomycetales;f\_\_Actinomycetaceae;g\_\_Actinomyces | 0 | 0.1% | 0.0% | 0.0% | 0.0% | 0.0% | 0.0% | 0.0% | 0.0% | 0.1% | 0.0% | 0.0% | 0.0% | 0.2% | 0.0% | 0.0% | 0.0% | 0.2% | 0.0% | 0.0% | 0.0% | 0.5% | 0.1% | 0.0% | 2.1% |
|  | k\_\_Bacteria;p\_\_Firmicutes;c\_\_Clostridia;o\_\_Clostridiales;f\_\_Lachnospiraceae;g\_\_Blautia | 0 | 0.1% | 0.0% | 0.0% | 0.0% | 0.0% | 0.0% | 0.0% | 0.0% | 0.0% | 0.0% | 0.0% | 0.0% | 0.0% | 0.0% | 0.1% | 0.0% | 0.1% | 0.0% | 0.0% | 0.0% | 0.0% | 0.1% | 2.6% | 0.0% |
|  | k\_\_Bacteria;p\_\_Proteobacteria;c\_\_Alphaproteobacteria;o\_\_Rhizobiales;f\_\_Bradyrhizobiaceae;g\_\_Unclassified\_Bradyrhizobiaceae | 0 | 0.1% | 0.0% | 0.0% | 0.4% | 0.3% | 0.0% | 0.2% | 0.1% | 0.0% | 0.1% | 0.0% | 0.2% | 0.1% | 0.0% | 0.1% | 0.4% | 0.3% | 0.2% | 0.1% | 0.1% | 0.0% | 0.2% | 0.1% | 0.0% |
|  | k\_\_Bacteria;p\_\_Proteobacteria;c\_\_Gammaproteobacteria;o\_\_Pseudomonadales;f\_\_Pseudomonadaceae;g\_\_Pseudomonas | 0 | 0.1% | 0.0% | 0.0% | 0.3% | 0.1% | 0.0% | 0.1% | 0.1% | 0.1% | 0.3% | 0.1% | 0.3% | 0.1% | 0.0% | 0.1% | 0.3% | 0.4% | 0.2% | 0.0% | 0.1% | 0.1% | 0.2% | 0.0% | 0.0% |
|  | k\_\_Bacteria;p\_\_Actinobacteria;c\_\_Actinobacteria;o\_\_Actinomycetales;f\_\_Actinomycetaceae;g\_\_Unclassified\_Actinomycetaceae | 0 | 0.1% | 0.0% | 0.3% | 0.0% | 0.0% | 0.4% | 0.0% | 0.7% | 0.8% | 0.2% | 0.0% | 0.0% | 0.4% | 0.0% | 0.0% | 0.0% | 0.0% | 0.0% | 0.0% | 0.0% | 0.0% | 0.0% | 0.0% | 0.0% |
|  | k\_\_Bacteria;p\_\_Firmicutes;c\_\_Clostridia;o\_\_Clostridiales;f\_\_Lachnospiraceae;g\_\_Dorea | 0 | 0.1% | 0.0% | 0.0% | 0.1% | 0.0% | 0.0% | 0.0% | 0.0% | 0.0% | 0.0% | 0.0% | 0.1% | 0.0% | 0.0% | 0.3% | 0.0% | 0.0% | 0.0% | 0.0% | 0.0% | 0.2% | 0.4% | 1.4% | 0.0% |
|  | k\_\_Bacteria;p\_\_Firmicutes;c\_\_Bacilli;o\_\_Bacillales;f\_\_Planococcaceae;g\_\_Unclassified\_Planococcaceae | 0 | 0.1% | 0.1% | 0.0% | 0.2% | 0.1% | 0.0% | 0.1% | 0.0% | 0.1% | 0.0% | 0.0% | 0.0% | 0.0% | 0.0% | 0.0% | 0.1% | 0.0% | 0.0% | 0.0% | 0.0% | 0.0% | 0.1% | 1.6% | 0.0% |
|  | k\_\_Bacteria;p\_\_Proteobacteria;c\_\_Alphaproteobacteria;o\_\_Sphingomonadales;f\_\_Unclassified\_Sphingomonadales;g\_\_Unclassified\_Sphingomonadales | 0 | 0.1% | 0.0% | 0.0% | 0.4% | 0.8% | 0.0% | 0.0% | 0.0% | 0.0% | 0.1% | 0.0% | 0.1% | 0.0% | 0.0% | 0.2% | 0.2% | 0.1% | 0.2% | 0.1% | 0.0% | 0.0% | 0.1% | 0.1% | 0.0% |
|  | k\_\_Bacteria;p\_\_Firmicutes;c\_\_Clostridia;o\_\_Clostridiales;f\_\_Lachnospiraceae;g\_\_Coprococcus | 0 | 0.1% | 0.0% | 0.0% | 0.1% | 0.0% | 0.0% | 0.0% | 0.0% | 0.0% | 0.0% | 0.0% | 0.0% | 0.0% | 0.0% | 0.0% | 0.1% | 0.2% | 0.1% | 0.0% | 0.0% | 0.1% | 0.2% | 1.8% | 0.0% |
|  | k\_\_Bacteria;p\_\_Proteobacteria;c\_\_Deltaproteobacteria;o\_\_Desulfovibrionales;f\_\_Desulfovibrionaceae;g\_\_Unclassified\_Desulfovibrionaceae | 0 | 0.1% | 0.0% | 0.0% | 0.0% | 0.0% | 0.0% | 0.0% | 0.0% | 0.1% | 0.0% | 0.0% | 0.0% | 0.1% | 0.0% | 0.0% | 0.0% | 0.7% | 0.0% | 0.0% | 0.0% | 0.4% | 1.1% | 0.1% | 0.0% |
|  | k\_\_Bacteria;p\_\_Actinobacteria;c\_\_Actinobacteria;o\_\_Actinomycetales;f\_\_Micrococcaceae;g\_\_Arthrobacter | 0 | 0.1% | 0.0% | 0.0% | 0.0% | 0.0% | 2.1% | 0.0% | 0.0% | 0.0% | 0.0% | 0.0% | 0.0% | 0.0% | 0.0% | 0.0% | 0.0% | 0.0% | 0.1% | 0.0% | 0.0% | 0.0% | 0.0% | 0.0% | 0.0% |
|  | k\_\_Bacteria;p\_\_Bacteroidetes;c\_\_Bacteroidia;o\_\_Bacteroidales;f\_\_Rikenellaceae;g\_\_Unclassified\_Rikenellaceae | 0 | 0.1% | 0.0% | 0.0% | 0.0% | 0.0% | 0.0% | 0.0% | 0.0% | 0.1% | 0.0% | 0.0% | 0.0% | 0.0% | 0.0% | 0.1% | 0.0% | 0.4% | 0.0% | 0.0% | 0.0% | 0.2% | 1.4% | 0.1% | 0.0% |
|  | k\_\_Bacteria;p\_\_Tenericutes;c\_\_Mollicutes;o\_\_Acholeplasmatales;f\_\_Acholeplasmataceae;g\_\_Acholeplasma | 0 | 0.1% | 0.0% | 0.1% | 0.1% | 0.0% | 0.0% | 0.7% | 0.0% | 0.0% | 0.0% | 0.2% | 0.0% | 0.0% | 0.0% | 0.0% | 0.0% | 0.0% | 0.0% | 1.2% | 0.0% | 0.0% | 0.0% | 0.0% | 0.0% |
|  | k\_\_Bacteria;p\_\_Verrucomicrobia;c\_\_Verrucomicrobiae;o\_\_Verrucomicrobiales;f\_\_Verrucomicrobiaceae;g\_\_Akkermansia | 0 | 0.1% | 0.0% | 0.0% | 0.1% | 0.0% | 0.0% | 0.0% | 0.0% | 0.1% | 0.0% | 0.0% | 0.1% | 0.1% | 0.0% | 0.0% | 0.0% | 0.3% | 0.0% | 0.0% | 0.0% | 0.3% | 1.0% | 0.1% | 0.0% |
|  | k\_\_Bacteria;p\_\_GN02;c\_\_3BR-5F;o\_\_Unclassified\_3BR-5F;f\_\_Unclassified\_3BR-5F;g\_\_Unclassified\_3BR-5F | 0 | 0.1% | 0.0% | 0.0% | 0.1% | 0.4% | 0.0% | 0.1% | 0.0% | 0.0% | 0.0% | 0.0% | 0.1% | 0.0% | 0.0% | 0.2% | 0.3% | 0.4% | 0.1% | 0.1% | 0.1% | 0.0% | 0.1% | 0.1% | 0.0% |
|  | k\_\_Bacteria;p\_\_Firmicutes;c\_\_Clostridia;o\_\_Clostridiales;f\_\_Veillonellaceae;g\_\_Dialister | 0 | 0.1% | 0.0% | 0.0% | 0.0% | 0.0% | 0.0% | 0.1% | 0.0% | 0.0% | 0.0% | 0.0% | 0.0% | 0.0% | 0.0% | 0.7% | 0.0% | 0.1% | 0.4% | 0.0% | 0.0% | 0.5% | 0.0% | 0.3% | 0.0% |
|  | k\_\_Bacteria;p\_\_Proteobacteria;c\_\_Gammaproteobacteria;o\_\_Pseudomonadales;f\_\_Moraxellaceae;g\_\_Enhydrobacter | 0 | 0.1% | 0.0% | 0.0% | 0.3% | 0.1% | 0.0% | 0.0% | 0.1% | 0.0% | 0.1% | 0.0% | 0.2% | 0.0% | 0.0% | 0.2% | 0.4% | 0.1% | 0.1% | 0.1% | 0.0% | 0.0% | 0.2% | 0.1% | 0.0% |
|  | k\_\_Bacteria;p\_\_Bacteroidetes;c\_\_Bacteroidia;o\_\_Bacteroidales;f\_\_S24-7;g\_\_Unclassified\_S24-7 | 0 | 0.1% | 0.0% | 0.0% | 0.1% | 0.0% | 0.0% | 0.1% | 0.0% | 0.0% | 0.0% | 0.0% | 0.0% | 0.0% | 0.0% | 0.5% | 0.2% | 0.9% | 0.1% | 0.0% | 0.0% | 0.0% | 0.1% | 0.0% | 0.0% |
|  | k\_\_Bacteria;p\_\_Bacteroidetes;c\_\_Bacteroidia;o\_\_Bacteroidales;f\_\_Bacteroidaceae;g\_\_5-7N15 | 0 | 0.1% | 0.0% | 0.0% | 0.0% | 0.0% | 0.0% | 0.0% | 0.0% | 0.1% | 0.0% | 0.1% | 0.0% | 0.0% | 0.0% | 0.0% | 0.0% | 0.5% | 0.0% | 0.0% | 0.0% | 0.4% | 0.8% | 0.1% | 0.0% |
|  | k\_\_Bacteria;p\_\_Firmicutes;c\_\_Clostridia;o\_\_Clostridiales;f\_\_Ruminococcaceae;g\_\_Ruminococcus | 0 | 0.1% | 0.0% | 0.0% | 0.2% | 0.1% | 0.0% | 0.4% | 0.0% | 0.1% | 0.0% | 0.0% | 0.1% | 0.0% | 0.0% | 0.0% | 0.0% | 0.0% | 0.0% | 0.3% | 0.0% | 0.0% | 0.5% | 0.2% | 0.0% |
|  | k\_\_Bacteria;p\_\_Actinobacteria;c\_\_Actinobacteria;o\_\_Actinomycetales;f\_\_Streptomycetaceae;g\_\_Streptomyces | 0 | 0.1% | 0.0% | 0.0% | 0.2% | 0.2% | 0.0% | 0.1% | 0.0% | 0.0% | 0.0% | 0.0% | 0.1% | 0.0% | 0.0% | 0.2% | 0.0% | 0.5% | 0.2% | 0.1% | 0.0% | 0.0% | 0.1% | 0.1% | 0.0% |
|  | k\_\_Bacteria;p\_\_Proteobacteria;c\_\_Gammaproteobacteria;o\_\_Xanthomonadales;f\_\_Sinobacteraceae;g\_\_Unclassified\_Sinobacteraceae | 0 | 0.1% | 0.0% | 0.0% | 0.2% | 0.2% | 0.0% | 0.0% | 0.0% | 0.0% | 0.0% | 0.0% | 0.0% | 0.1% | 0.0% | 0.1% | 0.3% | 0.2% | 0.2% | 0.0% | 0.2% | 0.0% | 0.2% | 0.1% | 0.1% |
|  | k\_\_Bacteria;p\_\_Firmicutes;c\_\_Clostridia;o\_\_Clostridiales;f\_\_Ruminococcaceae;g\_\_Faecalibacterium | 0 | 0.1% | 0.0% | 0.0% | 0.0% | 0.0% | 0.0% | 0.0% | 0.0% | 0.0% | 0.0% | 0.0% | 0.0% | 0.0% | 0.0% | 0.0% | 0.0% | 0.3% | 0.1% | 0.0% | 0.0% | 0.1% | 0.0% | 1.2% | 0.0% |
|  | k\_\_Bacteria;p\_\_Firmicutes;c\_\_Clostridia;o\_\_Clostridiales;f\_\_Peptococcaceae;g\_\_Peptococcus | 0 | 0.1% | 0.2% | 0.0% | 0.0% | 0.0% | 0.0% | 0.0% | 0.0% | 0.0% | 0.0% | 0.0% | 0.2% | 0.0% | 0.1% | 0.0% | 0.0% | 0.2% | 0.2% | 0.3% | 0.0% | 0.3% | 0.0% | 0.4% | 0.0% |
|  | k\_\_Bacteria;p\_\_Cyanobacteria;c\_\_Chloroplast;o\_\_Streptophyta;f\_\_Unclassified\_Streptophyta;g\_\_Unclassified\_Streptophyta | 0 | 0.1% | 0.0% | 0.0% | 0.3% | 0.1% | 0.0% | 0.1% | 0.0% | 0.0% | 0.1% | 0.0% | 0.1% | 0.0% | 0.0% | 0.1% | 0.2% | 0.1% | 0.1% | 0.1% | 0.1% | 0.1% | 0.1% | 0.0% | 0.0% |
|  | k\_\_Bacteria;p\_\_Firmicutes;c\_\_Clostridia;o\_\_Clostridiales;f\_\_Peptococcaceae;g\_\_Unclassified\_Peptococcaceae | 0 | 0.1% | 0.0% | 0.0% | 0.0% | 0.0% | 0.0% | 0.0% | 0.0% | 0.1% | 0.0% | 0.0% | 0.0% | 0.0% | 0.0% | 0.0% | 0.0% | 0.3% | 0.0% | 0.0% | 0.0% | 0.2% | 0.9% | 0.1% | 0.0% |
|  | k\_\_Bacteria;p\_\_Actinobacteria;c\_\_Actinobacteria;o\_\_Actinomycetales;f\_\_Microbacteriaceae;g\_\_Microbacterium | 0 | 0.1% | 0.0% | 0.0% | 0.0% | 0.3% | 0.0% | 0.0% | 0.1% | 0.1% | 0.1% | 0.0% | 0.1% | 0.1% | 0.0% | 0.0% | 0.2% | 0.1% | 0.1% | 0.0% | 0.0% | 0.0% | 0.1% | 0.0% | 0.0% |
|  | k\_\_Bacteria;p\_\_Proteobacteria;c\_\_Gammaproteobacteria;o\_\_Aeromonadales;f\_\_Aeromonadaceae;g\_\_Unclassified\_Aeromonadaceae | 0 | 0.1% | 0.0% | 0.0% | 0.4% | 0.0% | 0.0% | 0.0% | 0.0% | 0.0% | 0.0% | 0.0% | 0.1% | 0.0% | 0.0% | 0.0% | 0.3% | 0.2% | 0.1% | 0.1% | 0.0% | 0.0% | 0.1% | 0.1% | 0.0% |
|  | k\_\_Bacteria;p\_\_Firmicutes;c\_\_Bacilli;o\_\_Bacillales;f\_\_Bacillaceae;g\_\_Bacillus | 0 | 0.1% | 0.0% | 0.0% | 0.1% | 0.0% | 0.0% | 0.0% | 0.0% | 0.0% | 0.0% | 0.0% | 0.0% | 0.0% | 0.0% | 0.2% | 0.1% | 0.4% | 0.3% | 0.0% | 0.0% | 0.0% | 0.1% | 0.0% | 0.0% |
|  | k\_\_Bacteria;p\_\_Bacteroidetes;c\_\_Bacteroidia;o\_\_Bacteroidales;f\_\_Bacteroidaceae;g\_\_Bacteroides | 0 | 0.1% | 0.0% | 0.0% | 0.1% | 0.1% | 0.0% | 0.0% | 0.0% | 0.0% | 0.0% | 0.0% | 0.0% | 0.0% | 0.0% | 0.1% | 0.2% | 0.1% | 0.1% | 0.0% | 0.1% | 0.4% | 0.1% | 0.0% | 0.0% |
|  | k\_\_Bacteria;p\_\_Firmicutes;c\_\_Clostridia;o\_\_Clostridiales;f\_\_Christensenellaceae;g\_\_Unclassified\_Christensenellaceae | 0 | 0.1% | 0.0% | 0.0% | 0.1% | 0.0% | 0.0% | 0.0% | 0.0% | 0.0% | 0.0% | 0.0% | 0.0% | 0.0% | 0.0% | 0.1% | 0.0% | 0.2% | 0.0% | 0.0% | 0.0% | 0.1% | 0.8% | 0.1% | 0.0% |
|  | k\_\_Bacteria;p\_\_Bacteroidetes;c\_\_Bacteroidia;o\_\_Bacteroidales;f\_\_Rikenellaceae;g\_\_Blvii28 | 0 | 0.1% | 0.0% | 0.0% | 0.0% | 0.0% | 0.0% | 0.0% | 0.0% | 0.0% | 0.0% | 0.0% | 0.0% | 0.0% | 0.0% | 0.0% | 0.0% | 0.0% | 0.0% | 1.4% | 0.0% | 0.0% | 0.0% | 0.0% | 0.0% |
|  | k\_\_Bacteria;p\_\_SR1;c\_\_Unclassified\_SR1;o\_\_Unclassified\_SR1;f\_\_Unclassified\_SR1;g\_\_Unclassified\_SR1 | 0 | 0.1% | 0.0% | 0.0% | 0.0% | 0.0% | 0.0% | 0.0% | 0.0% | 0.0% | 0.0% | 0.0% | 0.0% | 0.0% | 0.0% | 0.7% | 0.0% | 0.0% | 0.0% | 0.1% | 0.0% | 0.5% | 0.0% | 0.0% | 0.0% |
|  | k\_\_Bacteria;p\_\_Bacteroidetes;c\_\_Cytophagia;o\_\_Cytophagales;f\_\_Cytophagaceae;g\_\_Hymenobacter | 0 | 0.1% | 0.0% | 0.0% | 0.0% | 0.0% | 0.0% | 0.0% | 0.0% | 0.0% | 0.0% | 0.0% | 0.0% | 0.0% | 1.4% | 0.0% | 0.0% | 0.0% | 0.0% | 0.0% | 0.0% | 0.0% | 0.0% | 0.0% | 0.0% |
|  | k\_\_Bacteria;p\_\_Proteobacteria;c\_\_Gammaproteobacteria;o\_\_Pasteurellales;f\_\_Pasteurellaceae;g\_\_Actinobacillus | 0 | 0.1% | 1.2% | 0.0% | 0.2% | 0.0% | 0.0% | 0.0% | 0.0% | 0.0% | 0.0% | 0.0% | 0.0% | 0.0% | 0.0% | 0.0% | 0.0% | 0.0% | 0.0% | 0.0% | 0.0% | 0.0% | 0.0% | 0.0% | 0.0% |
|  | k\_\_Bacteria;p\_\_Lentisphaerae;c\_\_[Lentisphaeria];o\_\_Victivallales;f\_\_Victivallaceae;g\_\_Unclassified\_Victivallaceae | 0 | 0.1% | 0.0% | 0.0% | 0.0% | 0.0% | 0.0% | 0.0% | 0.0% | 0.1% | 0.0% | 0.0% | 0.0% | 0.0% | 0.0% | 0.0% | 0.0% | 0.5% | 0.0% | 0.0% | 0.0% | 0.1% | 0.5% | 0.0% | 0.0% |
|  | k\_\_Bacteria;p\_\_Proteobacteria;c\_\_Deltaproteobacteria;o\_\_Myxococcales;f\_\_Myxococcaceae;g\_\_Unclassified\_Myxococcaceae | 0 | 0.1% | 0.0% | 0.0% | 0.1% | 0.1% | 0.0% | 0.0% | 0.0% | 0.0% | 0.0% | 0.0% | 0.0% | 0.0% | 0.0% | 0.1% | 0.1% | 0.4% | 0.0% | 0.1% | 0.0% | 0.0% | 0.1% | 0.1% | 0.0% |
|  | k\_\_Bacteria;p\_\_Firmicutes;c\_\_Clostridia;o\_\_Clostridiales;f\_\_Ruminococcaceae;g\_\_Oscillospira | 0 | 0.1% | 0.0% | 0.0% | 0.1% | 0.0% | 0.0% | 0.0% | 0.0% | 0.1% | 0.0% | 0.0% | 0.0% | 0.1% | 0.0% | 0.0% | 0.1% | 0.0% | 0.0% | 0.0% | 0.0% | 0.1% | 0.6% | 0.1% | 0.0% |
|  | k\_\_Bacteria;p\_\_Actinobacteria;c\_\_Actinobacteria;o\_\_Actinomycetales;f\_\_Brevibacteriaceae;g\_\_Brevibacterium | 0 | 0.1% | 0.0% | 0.0% | 0.1% | 0.5% | 0.0% | 0.0% | 0.0% | 0.0% | 0.0% | 0.0% | 0.0% | 0.0% | 0.0% | 0.0% | 0.1% | 0.2% | 0.1% | 0.0% | 0.0% | 0.0% | 0.1% | 0.0% | 0.0% |
|  | k\_\_Bacteria;p\_\_Verrucomicrobia;c\_\_Verruco-5;o\_\_WCHB1-41;f\_\_RFP12;g\_\_Unclassified\_RFP12 | 0 | 0.1% | 0.0% | 0.0% | 0.0% | 0.0% | 0.0% | 0.0% | 0.0% | 0.1% | 0.0% | 0.0% | 0.0% | 0.1% | 0.0% | 0.0% | 0.0% | 0.4% | 0.0% | 0.0% | 0.0% | 0.1% | 0.5% | 0.0% | 0.0% |
|  | k\_\_Bacteria;p\_\_Proteobacteria;c\_\_Alphaproteobacteria;o\_\_Caulobacterales;f\_\_Caulobacteraceae;g\_\_Brevundimonas | 0 | 0.1% | 0.0% | 0.0% | 0.0% | 0.0% | 0.0% | 0.0% | 0.1% | 0.1% | 0.2% | 0.0% | 0.2% | 0.1% | 0.0% | 0.0% | 0.0% | 0.0% | 0.1% | 0.0% | 0.0% | 0.0% | 0.1% | 0.1% | 0.0% |
|  | k\_\_Bacteria;p\_\_Spirochaetes;c\_\_Spirochaetes;o\_\_Spirochaetales;f\_\_Spirochaetaceae;g\_\_Treponema | 0 | 0.1% | 0.0% | 0.0% | 0.1% | 0.1% | 0.0% | 0.1% | 0.0% | 0.0% | 0.1% | 0.1% | 0.0% | 0.1% | 0.1% | 0.1% | 0.0% | 0.1% | 0.0% | 0.1% | 0.0% | 0.0% | 0.2% | 0.0% | 0.1% |
|  | k\_\_Bacteria;p\_\_[Thermi];c\_\_Deinococci;o\_\_Deinococcales;f\_\_Deinococcaceae;g\_\_Deinococcus | 0 | 0.1% | 0.0% | 0.0% | 0.1% | 0.1% | 0.0% | 0.1% | 0.0% | 0.0% | 0.0% | 0.0% | 0.0% | 0.0% | 0.0% | 0.1% | 0.2% | 0.2% | 0.1% | 0.0% | 0.0% | 0.0% | 0.1% | 0.0% | 0.0% |
|  | k\_\_Bacteria;p\_\_Proteobacteria;c\_\_Deltaproteobacteria;o\_\_Myxococcales;f\_\_Unclassified\_Myxococcales;g\_\_Unclassified\_Myxococcales | 0 | 0.1% | 0.0% | 0.0% | 0.0% | 0.0% | 0.0% | 0.0% | 0.0% | 0.0% | 0.0% | 0.0% | 0.0% | 0.0% | 0.0% | 0.1% | 0.2% | 0.2% | 0.0% | 0.1% | 0.0% | 0.0% | 0.4% | 0.0% | 0.0% |
|  | k\_\_Bacteria;p\_\_Actinobacteria;c\_\_Actinobacteria;o\_\_Actinomycetales;f\_\_Nocardioidaceae;g\_\_Aeromicrobium | 0 | 0.1% | 0.0% | 0.0% | 0.0% | 0.0% | 0.1% | 0.0% | 0.1% | 0.0% | 0.2% | 0.0% | 0.3% | 0.2% | 0.0% | 0.0% | 0.0% | 0.0% | 0.0% | 0.0% | 0.0% | 0.0% | 0.1% | 0.0% | 0.0% |
|  | k\_\_Bacteria;p\_\_Bacteroidetes;c\_\_Flavobacteriia;o\_\_Flavobacteriales;f\_\_Flavobacteriaceae;g\_\_Unclassified\_Flavobacteriaceae | 0 | 0.0% | 0.0% | 0.1% | 0.0% | 0.0% | 0.0% | 0.0% | 0.0% | 0.0% | 0.0% | 0.7% | 0.0% | 0.0% | 0.0% | 0.0% | 0.2% | 0.0% | 0.0% | 0.0% | 0.0% | 0.0% | 0.0% | 0.0% | 0.1% |
|  | k\_\_Bacteria;p\_\_Planctomycetes;c\_\_Phycisphaerae;o\_\_Phycisphaerales;f\_\_Unclassified\_Phycisphaerales;g\_\_Unclassified\_Phycisphaerales | 0 | 0.0% | 0.0% | 0.0% | 0.1% | 0.1% | 0.0% | 0.0% | 0.0% | 0.0% | 0.0% | 0.0% | 0.0% | 0.0% | 0.0% | 0.1% | 0.1% | 0.2% | 0.2% | 0.1% | 0.0% | 0.0% | 0.0% | 0.0% | 0.0% |
|  | k\_\_Bacteria;p\_\_Gemmatimonadetes;c\_\_Gemm-1;o\_\_Unclassified\_Gemm-1;f\_\_Unclassified\_Gemm-1;g\_\_Unclassified\_Gemm-1 | 0 | 0.0% | 0.0% | 0.0% | 0.0% | 0.0% | 0.0% | 0.0% | 0.0% | 0.0% | 0.0% | 0.0% | 0.0% | 0.0% | 0.0% | 0.0% | 0.0% | 0.1% | 0.0% | 0.0% | 0.0% | 0.0% | 0.8% | 0.1% | 0.0% |
|  | k\_\_Bacteria;p\_\_Proteobacteria;c\_\_Alphaproteobacteria;o\_\_Sphingomonadales;f\_\_Sphingomonadaceae;g\_\_Sphingobium | 0 | 0.0% | 0.0% | 0.0% | 0.1% | 0.1% | 0.0% | 0.0% | 0.0% | 0.0% | 0.0% | 0.0% | 0.1% | 0.0% | 0.0% | 0.1% | 0.1% | 0.1% | 0.1% | 0.0% | 0.0% | 0.0% | 0.1% | 0.0% | 0.0% |
|  | k\_\_Bacteria;p\_\_Acidobacteria;c\_\_Solibacteres;o\_\_Solibacterales;f\_\_Unclassified\_Solibacterales;g\_\_Unclassified\_Solibacterales | 0 | 0.0% | 0.0% | 0.0% | 0.1% | 0.1% | 0.0% | 0.0% | 0.0% | 0.0% | 0.0% | 0.0% | 0.0% | 0.0% | 0.0% | 0.0% | 0.1% | 0.2% | 0.0% | 0.0% | 0.0% | 0.0% | 0.1% | 0.0% | 0.0% |
|  | k\_\_Bacteria;p\_\_Proteobacteria;c\_\_Alphaproteobacteria;o\_\_Rhodospirillales;f\_\_Acetobacteraceae;g\_\_Unclassified\_Acetobacteraceae | 0 | 0.0% | 0.0% | 0.0% | 0.1% | 0.1% | 0.0% | 0.0% | 0.0% | 0.0% | 0.1% | 0.0% | 0.0% | 0.0% | 0.0% | 0.1% | 0.1% | 0.1% | 0.1% | 0.1% | 0.0% | 0.0% | 0.1% | 0.0% | 0.0% |
|  | k\_\_Bacteria;p\_\_Firmicutes;c\_\_Clostridia;o\_\_Clostridiales;f\_\_[Mogibacteriaceae];g\_\_Unclassified\_[Mogibacteriaceae] | 0 | 0.0% | 0.0% | 0.0% | 0.1% | 0.0% | 0.0% | 0.0% | 0.0% | 0.0% | 0.0% | 0.0% | 0.1% | 0.1% | 0.0% | 0.0% | 0.0% | 0.0% | 0.0% | 0.0% | 0.0% | 0.3% | 0.3% | 0.0% | 0.0% |
|  | k\_\_Bacteria;p\_\_Proteobacteria;c\_\_Alphaproteobacteria;o\_\_Rhodospirillales;f\_\_Rhodospirillaceae;g\_\_Unclassified\_Rhodospirillaceae | 0 | 0.0% | 0.0% | 0.0% | 0.1% | 0.1% | 0.0% | 0.0% | 0.0% | 0.0% | 0.1% | 0.0% | 0.0% | 0.0% | 0.0% | 0.1% | 0.0% | 0.1% | 0.1% | 0.0% | 0.1% | 0.0% | 0.1% | 0.0% | 0.0% |
|  | k\_\_Bacteria;p\_\_Firmicutes;c\_\_Clostridia;o\_\_Clostridiales;f\_\_Peptostreptococcaceae;g\_\_Peptostreptococcus | 0 | 0.0% | 0.0% | 0.1% | 0.0% | 0.0% | 0.0% | 0.0% | 0.0% | 0.0% | 0.0% | 0.0% | 0.0% | 0.0% | 0.0% | 0.3% | 0.0% | 0.1% | 0.0% | 0.0% | 0.0% | 0.4% | 0.0% | 0.0% | 0.0% |
|  | k\_\_Bacteria;p\_\_Proteobacteria;c\_\_Alphaproteobacteria;o\_\_Rhizobiales;f\_\_Rhizobiaceae;g\_\_Rhizobium | 0 | 0.0% | 0.0% | 0.0% | 0.0% | 0.1% | 0.0% | 0.0% | 0.0% | 0.0% | 0.0% | 0.0% | 0.0% | 0.0% | 0.0% | 0.0% | 0.4% | 0.0% | 0.1% | 0.0% | 0.0% | 0.0% | 0.0% | 0.0% | 0.0% |
|  | k\_\_Bacteria;p\_\_Proteobacteria;c\_\_Betaproteobacteria;o\_\_Burkholderiales;f\_\_Comamonadaceae;g\_\_Comamonas | 0 | 0.0% | 0.0% | 0.0% | 0.0% | 0.0% | 0.0% | 0.1% | 0.0% | 0.0% | 0.0% | 0.0% | 0.0% | 0.1% | 0.0% | 0.0% | 0.1% | 0.3% | 0.1% | 0.1% | 0.0% | 0.0% | 0.0% | 0.0% | 0.0% |
|  | k\_\_Bacteria;p\_\_Acidobacteria;c\_\_Acidobacteria-6;o\_\_iii1-15;f\_\_Unclassified\_iii1-15;g\_\_Unclassified\_iii1-15 | 0 | 0.0% | 0.0% | 0.0% | 0.1% | 0.0% | 0.0% | 0.0% | 0.0% | 0.0% | 0.0% | 0.0% | 0.0% | 0.0% | 0.0% | 0.0% | 0.1% | 0.0% | 0.0% | 0.0% | 0.0% | 0.0% | 0.6% | 0.0% | 0.0% |
|  | k\_\_Bacteria;p\_\_Firmicutes;c\_\_Erysipelotrichi;o\_\_Erysipelotrichales;f\_\_Erysipelotrichaceae;g\_\_Unclassified\_Erysipelotrichaceae | 0 | 0.0% | 0.0% | 0.0% | 0.0% | 0.0% | 0.0% | 0.0% | 0.0% | 0.0% | 0.0% | 0.0% | 0.0% | 0.0% | 0.0% | 0.0% | 0.0% | 0.2% | 0.0% | 0.0% | 0.0% | 0.1% | 0.2% | 0.2% | 0.0% |
|  | k\_\_Bacteria;p\_\_Firmicutes;c\_\_Bacilli;o\_\_Bacillales;f\_\_Bacillaceae;g\_\_Geobacillus | 0 | 0.0% | 0.0% | 0.0% | 0.0% | 0.0% | 0.0% | 0.0% | 0.0% | 0.0% | 0.0% | 0.0% | 0.0% | 0.0% | 0.0% | 0.0% | 0.0% | 0.0% | 0.0% | 0.0% | 0.0% | 0.0% | 0.8% | 0.1% | 0.0% |
|  | k\_\_Bacteria;p\_\_Proteobacteria;c\_\_Deltaproteobacteria;o\_\_Desulfovibrionales;f\_\_Desulfovibrionaceae;g\_\_Bilophila | 0 | 0.0% | 0.0% | 0.0% | 0.0% | 0.0% | 0.0% | 0.0% | 0.0% | 0.0% | 0.0% | 0.0% | 0.0% | 0.0% | 0.2% | 0.0% | 0.0% | 0.0% | 0.0% | 0.2% | 0.3% | 0.0% | 0.0% | 0.0% | 0.0% |
|  | k\_\_Bacteria;p\_\_Actinobacteria;c\_\_Actinobacteria;o\_\_Actinomycetales;f\_\_Propionibacteriaceae;g\_\_Propionibacterium | 0 | 0.0% | 0.1% | 0.0% | 0.1% | 0.1% | 0.0% | 0.0% | 0.0% | 0.0% | 0.0% | 0.0% | 0.1% | 0.0% | 0.0% | 0.0% | 0.0% | 0.0% | 0.0% | 0.0% | 0.0% | 0.0% | 0.1% | 0.0% | 0.0% |
|  | k\_\_Bacteria;p\_\_Bacteroidetes;c\_\_[Saprospirae];o\_\_[Saprospirales];f\_\_Chitinophagaceae;g\_\_Flavisolibacter | 0 | 0.0% | 0.0% | 0.0% | 0.0% | 0.0% | 0.0% | 0.0% | 0.2% | 0.0% | 0.5% | 0.1% | 0.0% | 0.0% | 0.0% | 0.0% | 0.0% | 0.0% | 0.0% | 0.0% | 0.0% | 0.0% | 0.0% | 0.0% | 0.0% |
|  | k\_\_Bacteria;p\_\_Proteobacteria;c\_\_Alphaproteobacteria;o\_\_Rhizobiales;f\_\_Hyphomicrobiaceae;g\_\_Rhodoplanes | 0 | 0.0% | 0.0% | 0.0% | 0.1% | 0.1% | 0.0% | 0.0% | 0.0% | 0.0% | 0.0% | 0.0% | 0.0% | 0.0% | 0.0% | 0.1% | 0.2% | 0.1% | 0.0% | 0.0% | 0.0% | 0.0% | 0.1% | 0.0% | 0.0% |
|  | k\_\_Bacteria;p\_\_Firmicutes;c\_\_Clostridia;o\_\_Clostridiales;f\_\_Lachnospiraceae;g\_\_[Ruminococcus] | 0 | 0.0% | 0.0% | 0.0% | 0.0% | 0.1% | 0.0% | 0.0% | 0.0% | 0.0% | 0.0% | 0.0% | 0.0% | 0.0% | 0.0% | 0.2% | 0.1% | 0.0% | 0.0% | 0.0% | 0.1% | 0.0% | 0.0% | 0.1% | 0.0% |
|  | k\_\_Bacteria;p\_\_Firmicutes;c\_\_Clostridia;o\_\_Clostridiales;f\_\_Clostridiaceae;g\_\_Unclassified\_Clostridiaceae | 0 | 0.0% | 0.0% | 0.0% | 0.0% | 0.0% | 0.0% | 0.0% | 0.0% | 0.0% | 0.1% | 0.0% | 0.0% | 0.0% | 0.0% | 0.0% | 0.1% | 0.0% | 0.0% | 0.0% | 0.0% | 0.1% | 0.1% | 0.2% | 0.0% |
|  | k\_\_Bacteria;p\_\_Firmicutes;c\_\_Clostridia;o\_\_Clostridiales;f\_\_Peptostreptococcaceae;g\_\_Filifactor | 0 | 0.0% | 0.0% | 0.0% | 0.0% | 0.0% | 0.0% | 0.0% | 0.0% | 0.0% | 0.0% | 0.0% | 0.0% | 0.0% | 0.0% | 0.7% | 0.0% | 0.0% | 0.0% | 0.0% | 0.0% | 0.0% | 0.0% | 0.0% | 0.0% |
|  | k\_\_Bacteria;p\_\_Proteobacteria;c\_\_Alphaproteobacteria;o\_\_Sphingomonadales;f\_\_Sphingomonadaceae;g\_\_Unclassified\_Sphingomonadaceae | 0 | 0.0% | 0.0% | 0.0% | 0.1% | 0.0% | 0.0% | 0.0% | 0.0% | 0.0% | 0.0% | 0.0% | 0.0% | 0.0% | 0.0% | 0.0% | 0.1% | 0.0% | 0.0% | 0.1% | 0.0% | 0.0% | 0.0% | 0.0% | 0.0% |
|  | k\_\_Bacteria;p\_\_Firmicutes;c\_\_Erysipelotrichi;o\_\_Erysipelotrichales;f\_\_Erysipelotrichaceae;g\_\_Bulleidia | 0 | 0.0% | 0.0% | 0.0% | 0.0% | 0.0% | 0.0% | 0.0% | 0.0% | 0.0% | 0.0% | 0.0% | 0.0% | 0.0% | 0.0% | 0.1% | 0.0% | 0.4% | 0.0% | 0.0% | 0.0% | 0.0% | 0.0% | 0.0% | 0.0% |
|  | k\_\_Bacteria;p\_\_TM7;c\_\_TM7-3;o\_\_CW040;f\_\_F16;g\_\_Unclassified\_F16 | 0 | 0.0% | 0.0% | 0.0% | 0.1% | 0.1% | 0.0% | 0.0% | 0.0% | 0.0% | 0.0% | 0.0% | 0.0% | 0.1% | 0.0% | 0.2% | 0.0% | 0.1% | 0.0% | 0.0% | 0.0% | 0.0% | 0.0% | 0.0% | 0.0% |
|  | k\_\_Bacteria;p\_\_Proteobacteria;c\_\_Betaproteobacteria;o\_\_Burkholderiales;f\_\_Oxalobacteraceae;g\_\_Unclassified\_Oxalobacteraceae | 0 | 0.0% | 0.0% | 0.0% | 0.0% | 0.0% | 0.3% | 0.0% | 0.0% | 0.0% | 0.0% | 0.0% | 0.0% | 0.0% | 0.0% | 0.0% | 0.0% | 0.1% | 0.0% | 0.0% | 0.0% | 0.0% | 0.0% | 0.0% | 0.0% |
|  | k\_\_Bacteria;p\_\_Actinobacteria;c\_\_Actinobacteria;o\_\_Actinomycetales;f\_\_Unclassified\_Actinomycetales;g\_\_Unclassified\_Actinomycetales | 0 | 0.0% | 0.0% | 0.0% | 0.0% | 0.3% | 0.0% | 0.0% | 0.0% | 0.0% | 0.0% | 0.0% | 0.1% | 0.0% | 0.0% | 0.0% | 0.0% | 0.0% | 0.1% | 0.0% | 0.0% | 0.0% | 0.1% | 0.0% | 0.0% |
|  | k\_\_Bacteria;p\_\_Actinobacteria;c\_\_Actinobacteria;o\_\_Actinomycetales;f\_\_Propionibacteriaceae;g\_\_Unclassified\_Propionibacteriaceae | 0 | 0.0% | 0.0% | 0.1% | 0.0% | 0.0% | 0.0% | 0.0% | 0.0% | 0.0% | 0.0% | 0.1% | 0.0% | 0.0% | 0.1% | 0.0% | 0.0% | 0.0% | 0.0% | 0.1% | 0.0% | 0.0% | 0.0% | 0.0% | 0.1% |
|  | k\_\_Bacteria;p\_\_Proteobacteria;c\_\_Betaproteobacteria;o\_\_SC-I-84;f\_\_Unclassified\_SC-I-84;g\_\_Unclassified\_SC-I-84 | 0 | 0.0% | 0.0% | 0.0% | 0.0% | 0.0% | 0.0% | 0.0% | 0.0% | 0.0% | 0.0% | 0.0% | 0.0% | 0.0% | 0.0% | 0.0% | 0.0% | 0.0% | 0.0% | 0.0% | 0.0% | 0.0% | 0.5% | 0.0% | 0.0% |
|  | k\_\_Bacteria;p\_\_Proteobacteria;c\_\_Epsilonproteobacteria;o\_\_Campylobacterales;f\_\_Campylobacteraceae;g\_\_Sulfurospirillum | 0 | 0.0% | 0.0% | 0.0% | 0.1% | 0.0% | 0.0% | 0.0% | 0.0% | 0.0% | 0.0% | 0.0% | 0.0% | 0.0% | 0.0% | 0.0% | 0.0% | 0.0% | 0.1% | 0.1% | 0.0% | 0.0% | 0.1% | 0.0% | 0.0% |
|  | k\_\_Bacteria;p\_\_Cyanobacteria;c\_\_4C0d-2;o\_\_YS2;f\_\_Unclassified\_YS2;g\_\_Unclassified\_YS2 | 0 | 0.0% | 0.0% | 0.0% | 0.0% | 0.0% | 0.0% | 0.0% | 0.0% | 0.0% | 0.0% | 0.0% | 0.0% | 0.1% | 0.0% | 0.0% | 0.0% | 0.1% | 0.0% | 0.0% | 0.0% | 0.0% | 0.3% | 0.0% | 0.0% |
|  | k\_\_Bacteria;p\_\_Actinobacteria;c\_\_Rubrobacteria;o\_\_Rubrobacterales;f\_\_Rubrobacteraceae;g\_\_Rubrobacter | 0 | 0.0% | 0.0% | 0.0% | 0.1% | 0.0% | 0.0% | 0.0% | 0.0% | 0.0% | 0.0% | 0.0% | 0.0% | 0.0% | 0.0% | 0.0% | 0.0% | 0.2% | 0.0% | 0.0% | 0.0% | 0.0% | 0.0% | 0.0% | 0.0% |
|  | k\_\_Bacteria;p\_\_Bacteroidetes;c\_\_Bacteroidia;o\_\_Bacteroidales;f\_\_Bacteroidaceae;g\_\_Unclassified\_Bacteroidaceae | 0 | 0.0% | 0.0% | 0.0% | 0.0% | 0.0% | 0.0% | 0.0% | 0.0% | 0.0% | 0.0% | 0.0% | 0.0% | 0.0% | 0.0% | 0.0% | 0.0% | 0.1% | 0.0% | 0.0% | 0.0% | 0.1% | 0.2% | 0.0% | 0.0% |
|  | k\_\_Bacteria;p\_\_Bacteroidetes;c\_\_Flavobacteriia;o\_\_Flavobacteriales;f\_\_[Weeksellaceae];g\_\_Cloacibacterium | 0 | 0.0% | 0.0% | 0.0% | 0.0% | 0.0% | 0.0% | 0.0% | 0.0% | 0.0% | 0.0% | 0.0% | 0.0% | 0.0% | 0.0% | 0.0% | 0.1% | 0.0% | 0.0% | 0.0% | 0.0% | 0.0% | 0.4% | 0.0% | 0.0% |
|  | k\_\_Bacteria;p\_\_Actinobacteria;c\_\_Coriobacteriia;o\_\_Coriobacteriales;f\_\_Coriobacteriaceae;g\_\_Unclassified\_Coriobacteriaceae | 0 | 0.0% | 0.0% | 0.0% | 0.0% | 0.0% | 0.0% | 0.0% | 0.0% | 0.0% | 0.1% | 0.0% | 0.0% | 0.0% | 0.0% | 0.0% | 0.0% | 0.2% | 0.0% | 0.0% | 0.0% | 0.0% | 0.0% | 0.0% | 0.0% |
|  | k\_\_Bacteria;p\_\_Proteobacteria;c\_\_Alphaproteobacteria;o\_\_Rhizobiales;f\_\_Bradyrhizobiaceae;g\_\_Balneimonas | 0 | 0.0% | 0.0% | 0.0% | 0.0% | 0.0% | 0.4% | 0.0% | 0.0% | 0.0% | 0.0% | 0.0% | 0.0% | 0.0% | 0.0% | 0.0% | 0.0% | 0.0% | 0.0% | 0.0% | 0.0% | 0.0% | 0.0% | 0.0% | 0.0% |
|  | k\_\_Bacteria;p\_\_Proteobacteria;c\_\_Alphaproteobacteria;o\_\_Sphingomonadales;f\_\_Sphingomonadaceae;g\_\_Novosphingobium | 0 | 0.0% | 0.0% | 0.0% | 0.0% | 0.0% | 0.0% | 0.0% | 0.0% | 0.0% | 0.0% | 0.0% | 0.0% | 0.0% | 0.0% | 0.1% | 0.1% | 0.1% | 0.0% | 0.0% | 0.0% | 0.0% | 0.1% | 0.0% | 0.0% |
|  | k\_\_Bacteria;p\_\_Chloroflexi;c\_\_Anaerolineae;o\_\_SBR1031;f\_\_A4b;g\_\_Unclassified\_A4b | 0 | 0.0% | 0.0% | 0.0% | 0.0% | 0.0% | 0.0% | 0.0% | 0.0% | 0.0% | 0.0% | 0.0% | 0.0% | 0.0% | 0.0% | 0.1% | 0.1% | 0.0% | 0.0% | 0.0% | 0.0% | 0.0% | 0.0% | 0.0% | 0.1% |
|  | k\_\_Bacteria;p\_\_Firmicutes;c\_\_Bacilli;o\_\_Turicibacterales;f\_\_Turicibacteraceae;g\_\_Turicibacter | 0 | 0.0% | 0.0% | 0.0% | 0.0% | 0.0% | 0.0% | 0.0% | 0.0% | 0.0% | 0.0% | 0.0% | 0.1% | 0.0% | 0.0% | 0.1% | 0.0% | 0.1% | 0.0% | 0.0% | 0.0% | 0.0% | 0.1% | 0.1% | 0.0% |
|  | k\_\_Bacteria;p\_\_Tenericutes;c\_\_Mollicutes;o\_\_Mycoplasmatales;f\_\_Mycoplasmataceae;g\_\_Mycoplasma | 0 | 0.0% | 0.0% | 0.0% | 0.0% | 0.0% | 0.0% | 0.0% | 0.0% | 0.0% | 0.0% | 0.0% | 0.0% | 0.0% | 0.0% | 0.0% | 0.0% | 0.0% | 0.0% | 0.3% | 0.0% | 0.0% | 0.0% | 0.0% | 0.1% |
|  | k\_\_Bacteria;p\_\_Proteobacteria;c\_\_Gammaproteobacteria;o\_\_Cardiobacteriales;f\_\_Cardiobacteriaceae;g\_\_Suttonella | 0 | 0.0% | 0.0% | 0.0% | 0.0% | 0.0% | 0.0% | 0.0% | 0.0% | 0.0% | 0.0% | 0.0% | 0.0% | 0.0% | 0.0% | 0.0% | 0.0% | 0.0% | 0.0% | 0.0% | 0.0% | 0.4% | 0.0% | 0.0% | 0.0% |
|  | k\_\_Bacteria;p\_\_Proteobacteria;c\_\_Gammaproteobacteria;o\_\_Xanthomonadales;f\_\_Xanthomonadaceae;g\_\_Luteimonas | 0 | 0.0% | 0.0% | 0.0% | 0.0% | 0.0% | 0.0% | 0.0% | 0.0% | 0.0% | 0.1% | 0.0% | 0.1% | 0.0% | 0.0% | 0.0% | 0.0% | 0.0% | 0.0% | 0.0% | 0.0% | 0.2% | 0.0% | 0.0% | 0.0% |
|  | k\_\_Bacteria;p\_\_Firmicutes;c\_\_Erysipelotrichi;o\_\_Erysipelotrichales;f\_\_Erysipelotrichaceae;g\_\_p-75-a5 | 0 | 0.0% | 0.0% | 0.0% | 0.0% | 0.0% | 0.0% | 0.1% | 0.0% | 0.0% | 0.0% | 0.0% | 0.0% | 0.0% | 0.0% | 0.0% | 0.0% | 0.0% | 0.0% | 0.1% | 0.0% | 0.1% | 0.0% | 0.0% | 0.0% |
|  | k\_\_Bacteria;p\_\_Tenericutes;c\_\_Mollicutes;o\_\_Acholeplasmatales;f\_\_Acholeplasmataceae;g\_\_Unclassified\_Acholeplasmataceae | 0 | 0.0% | 0.0% | 0.1% | 0.0% | 0.0% | 0.0% | 0.0% | 0.0% | 0.0% | 0.0% | 0.3% | 0.0% | 0.0% | 0.0% | 0.0% | 0.0% | 0.0% | 0.0% | 0.0% | 0.0% | 0.0% | 0.0% | 0.0% | 0.0% |
|  | k\_\_Bacteria;p\_\_Actinobacteria;c\_\_Actinobacteria;o\_\_Actinomycetales;f\_\_Mycobacteriaceae;g\_\_Mycobacterium | 0 | 0.0% | 0.0% | 0.0% | 0.0% | 0.0% | 0.0% | 0.0% | 0.0% | 0.0% | 0.0% | 0.0% | 0.0% | 0.0% | 0.0% | 0.1% | 0.0% | 0.1% | 0.0% | 0.0% | 0.0% | 0.0% | 0.0% | 0.0% | 0.0% |
|  | k\_\_Bacteria;p\_\_Firmicutes;c\_\_Bacilli;o\_\_Bacillales;f\_\_Bacillaceae;g\_\_Anoxybacillus | 0 | 0.0% | 0.0% | 0.0% | 0.0% | 0.0% | 0.0% | 0.0% | 0.0% | 0.0% | 0.0% | 0.0% | 0.0% | 0.0% | 0.0% | 0.0% | 0.0% | 0.0% | 0.0% | 0.0% | 0.0% | 0.0% | 0.3% | 0.0% | 0.0% |
|  | k\_\_Bacteria;p\_\_Bacteroidetes;c\_\_Bacteroidia;o\_\_Bacteroidales;f\_\_[Barnesiellaceae];g\_\_Unclassified\_[Barnesiellaceae] | 0 | 0.0% | 0.0% | 0.0% | 0.0% | 0.0% | 0.0% | 0.0% | 0.0% | 0.0% | 0.0% | 0.0% | 0.0% | 0.0% | 0.0% | 0.0% | 0.0% | 0.1% | 0.0% | 0.0% | 0.0% | 0.0% | 0.2% | 0.0% | 0.0% |
|  | k\_\_Bacteria;p\_\_Firmicutes;c\_\_Bacilli;o\_\_Lactobacillales;f\_\_Enterococcaceae;g\_\_Unclassified\_Enterococcaceae | 0 | 0.0% | 0.1% | 0.0% | 0.0% | 0.0% | 0.1% | 0.0% | 0.0% | 0.0% | 0.0% | 0.0% | 0.0% | 0.0% | 0.0% | 0.0% | 0.0% | 0.1% | 0.0% | 0.0% | 0.0% | 0.0% | 0.0% | 0.1% | 0.0% |
|  | k\_\_Bacteria;p\_\_Proteobacteria;c\_\_Gammaproteobacteria;o\_\_Enterobacteriales;f\_\_Enterobacteriaceae;g\_\_Serratia | 0 | 0.0% | 0.0% | 0.0% | 0.0% | 0.1% | 0.0% | 0.0% | 0.0% | 0.0% | 0.0% | 0.0% | 0.0% | 0.0% | 0.0% | 0.0% | 0.1% | 0.1% | 0.0% | 0.0% | 0.0% | 0.0% | 0.0% | 0.0% | 0.0% |
|  | k\_\_Bacteria;p\_\_Tenericutes;c\_\_Mollicutes;o\_\_RF39;f\_\_Unclassified\_RF39;g\_\_Unclassified\_RF39 | 0 | 0.0% | 0.0% | 0.0% | 0.0% | 0.0% | 0.0% | 0.0% | 0.0% | 0.1% | 0.0% | 0.0% | 0.1% | 0.0% | 0.0% | 0.0% | 0.0% | 0.1% | 0.0% | 0.0% | 0.0% | 0.0% | 0.1% | 0.0% | 0.0% |
|  | k\_\_Bacteria;p\_\_Proteobacteria;c\_\_Alphaproteobacteria;o\_\_Rhodobacterales;f\_\_Rhodobacteraceae;g\_\_Unclassified\_Rhodobacteraceae | 0 | 0.0% | 0.0% | 0.0% | 0.1% | 0.0% | 0.1% | 0.0% | 0.0% | 0.0% | 0.0% | 0.0% | 0.0% | 0.0% | 0.0% | 0.0% | 0.0% | 0.0% | 0.0% | 0.0% | 0.0% | 0.0% | 0.0% | 0.0% | 0.0% |
|  | k\_\_Bacteria;p\_\_Firmicutes;c\_\_Erysipelotrichi;o\_\_Erysipelotrichales;f\_\_Erysipelotrichaceae;g\_\_Allobaculum | 0 | 0.0% | 0.0% | 0.0% | 0.0% | 0.0% | 0.0% | 0.0% | 0.0% | 0.0% | 0.0% | 0.0% | 0.0% | 0.0% | 0.0% | 0.1% | 0.0% | 0.1% | 0.0% | 0.0% | 0.0% | 0.0% | 0.0% | 0.0% | 0.0% |
|  | k\_\_Bacteria;p\_\_Proteobacteria;c\_\_Deltaproteobacteria;o\_\_Desulfovibrionales;f\_\_Desulfovibrionaceae;g\_\_Desulfovibrio | 0 | 0.0% | 0.0% | 0.0% | 0.0% | 0.0% | 0.0% | 0.0% | 0.0% | 0.0% | 0.0% | 0.0% | 0.0% | 0.0% | 0.0% | 0.0% | 0.0% | 0.0% | 0.0% | 0.0% | 0.0% | 0.0% | 0.2% | 0.0% | 0.0% |
|  | k\_\_Bacteria;p\_\_Gemmatimonadetes;c\_\_Gemmatimonadetes;o\_\_Unclassified\_Gemmatimonadetes;f\_\_Unclassified\_Gemmatimonadetes;g\_\_Unclassified\_Gemmatimonadetes | 0 | 0.0% | 0.0% | 0.0% | 0.0% | 0.0% | 0.0% | 0.0% | 0.0% | 0.0% | 0.0% | 0.0% | 0.0% | 0.0% | 0.0% | 0.0% | 0.1% | 0.0% | 0.0% | 0.0% | 0.0% | 0.0% | 0.2% | 0.0% | 0.0% |
|  | k\_\_Bacteria;p\_\_Bacteroidetes;c\_\_Flavobacteriia;o\_\_Flavobacteriales;f\_\_[Weeksellaceae];g\_\_Unclassified\_[Weeksellaceae] | 0 | 0.0% | 0.0% | 0.0% | 0.0% | 0.0% | 0.0% | 0.0% | 0.0% | 0.0% | 0.0% | 0.0% | 0.0% | 0.0% | 0.0% | 0.0% | 0.0% | 0.0% | 0.0% | 0.0% | 0.0% | 0.0% | 0.1% | 0.3% | 0.0% |
|  | k\_\_Bacteria;p\_\_Bacteroidetes;c\_\_Bacteroidia;o\_\_Bacteroidales;f\_\_RF16;g\_\_Unclassified\_RF16 | 0 | 0.0% | 0.0% | 0.0% | 0.0% | 0.0% | 0.0% | 0.0% | 0.0% | 0.0% | 0.0% | 0.0% | 0.0% | 0.0% | 0.0% | 0.0% | 0.0% | 0.0% | 0.0% | 0.0% | 0.0% | 0.1% | 0.2% | 0.0% | 0.0% |
|  | k\_\_Bacteria;p\_\_Actinobacteria;c\_\_Actinobacteria;o\_\_Actinomycetales;f\_\_Dietziaceae;g\_\_Dietzia | 0 | 0.0% | 0.0% | 0.0% | 0.0% | 0.0% | 0.0% | 0.0% | 0.0% | 0.0% | 0.0% | 0.0% | 0.0% | 0.0% | 0.0% | 0.1% | 0.0% | 0.1% | 0.0% | 0.0% | 0.0% | 0.0% | 0.0% | 0.0% | 0.0% |
|  | k\_\_Bacteria;p\_\_Firmicutes;c\_\_Clostridia;o\_\_Clostridiales;f\_\_Veillonellaceae;g\_\_Phascolarctobacterium | 0 | 0.0% | 0.0% | 0.0% | 0.0% | 0.0% | 0.0% | 0.0% | 0.0% | 0.0% | 0.0% | 0.0% | 0.0% | 0.0% | 0.0% | 0.0% | 0.0% | 0.1% | 0.0% | 0.0% | 0.0% | 0.0% | 0.1% | 0.0% | 0.0% |
|  | k\_\_Bacteria;p\_\_Actinobacteria;c\_\_Actinobacteria;o\_\_Actinomycetales;f\_\_Micromonosporaceae;g\_\_Unclassified\_Micromonosporaceae | 0 | 0.0% | 0.0% | 0.0% | 0.0% | 0.0% | 0.0% | 0.0% | 0.0% | 0.0% | 0.0% | 0.0% | 0.0% | 0.0% | 0.0% | 0.1% | 0.0% | 0.2% | 0.0% | 0.0% | 0.0% | 0.0% | 0.0% | 0.0% | 0.0% |
|  | k\_\_Bacteria;p\_\_Proteobacteria;c\_\_Betaproteobacteria;o\_\_Rhodocyclales;f\_\_Rhodocyclaceae;g\_\_Unclassified\_Rhodocyclaceae | 0 | 0.0% | 0.0% | 0.0% | 0.0% | 0.0% | 0.0% | 0.0% | 0.0% | 0.0% | 0.0% | 0.0% | 0.0% | 0.0% | 0.0% | 0.1% | 0.0% | 0.0% | 0.0% | 0.0% | 0.0% | 0.0% | 0.1% | 0.0% | 0.0% |
|  | k\_\_Bacteria;p\_\_Firmicutes;c\_\_Clostridia;o\_\_Clostridiales;f\_\_[Tissierellaceae];g\_\_Tissierella\_Soehngenia | 0 | 0.0% | 0.0% | 0.0% | 0.0% | 0.1% | 0.0% | 0.0% | 0.0% | 0.1% | 0.0% | 0.0% | 0.0% | 0.1% | 0.0% | 0.0% | 0.0% | 0.0% | 0.0% | 0.0% | 0.0% | 0.0% | 0.0% | 0.0% | 0.0% |
|  | k\_\_Bacteria;p\_\_Firmicutes;c\_\_Bacilli;o\_\_Bacillales;f\_\_Bacillaceae;g\_\_Unclassified\_Bacillaceae | 0 | 0.0% | 0.0% | 0.0% | 0.0% | 0.0% | 0.0% | 0.0% | 0.0% | 0.0% | 0.0% | 0.0% | 0.1% | 0.0% | 0.0% | 0.0% | 0.0% | 0.1% | 0.0% | 0.0% | 0.0% | 0.0% | 0.0% | 0.0% | 0.0% |
|  | k\_\_Bacteria;p\_\_Firmicutes;c\_\_Clostridia;o\_\_Clostridiales;f\_\_[Tissierellaceae];g\_\_Finegoldia | 0 | 0.0% | 0.0% | 0.0% | 0.0% | 0.0% | 0.0% | 0.0% | 0.0% | 0.0% | 0.0% | 0.0% | 0.0% | 0.0% | 0.0% | 0.1% | 0.0% | 0.0% | 0.0% | 0.1% | 0.0% | 0.0% | 0.0% | 0.0% | 0.0% |
|  | k\_\_Bacteria;p\_\_Actinobacteria;c\_\_Acidimicrobiia;o\_\_Acidimicrobiales;f\_\_Unclassified\_Acidimicrobiales;g\_\_Unclassified\_Acidimicrobiales | 0 | 0.0% | 0.0% | 0.0% | 0.0% | 0.0% | 0.0% | 0.0% | 0.0% | 0.0% | 0.1% | 0.0% | 0.0% | 0.0% | 0.0% | 0.1% | 0.0% | 0.0% | 0.0% | 0.0% | 0.0% | 0.0% | 0.1% | 0.0% | 0.0% |
|  | k\_\_Bacteria;p\_\_Proteobacteria;c\_\_Alphaproteobacteria;o\_\_Unclassified\_Alphaproteobacteria;f\_\_Unclassified\_Alphaproteobacteria;g\_\_Unclassified\_Alphaproteobacteria | 0 | 0.0% | 0.0% | 0.0% | 0.0% | 0.0% | 0.0% | 0.0% | 0.0% | 0.0% | 0.0% | 0.0% | 0.0% | 0.0% | 0.0% | 0.0% | 0.0% | 0.0% | 0.0% | 0.0% | 0.0% | 0.1% | 0.1% | 0.0% | 0.0% |
|  | k\_\_Bacteria;p\_\_Firmicutes;c\_\_Bacilli;o\_\_Bacillales;f\_\_Planococcaceae;g\_\_Planococcus | 0 | 0.0% | 0.0% | 0.0% | 0.0% | 0.0% | 0.0% | 0.0% | 0.0% | 0.0% | 0.0% | 0.0% | 0.0% | 0.0% | 0.0% | 0.0% | 0.0% | 0.1% | 0.1% | 0.0% | 0.0% | 0.0% | 0.0% | 0.0% | 0.0% |
|  | k\_\_Bacteria;p\_\_Acidobacteria;c\_\_Acidobacteriia;o\_\_Acidobacteriales;f\_\_Koribacteraceae;g\_\_Unclassified\_Koribacteraceae | 0 | 0.0% | 0.0% | 0.0% | 0.1% | 0.0% | 0.0% | 0.0% | 0.0% | 0.0% | 0.0% | 0.0% | 0.0% | 0.0% | 0.0% | 0.0% | 0.1% | 0.0% | 0.0% | 0.0% | 0.0% | 0.0% | 0.1% | 0.0% | 0.0% |
|  | k\_\_Bacteria;p\_\_Bacteroidetes;c\_\_Bacteroidia;o\_\_Bacteroidales;f\_\_Prevotellaceae;g\_\_Prevotella | 0 | 0.0% | 0.0% | 0.0% | 0.0% | 0.0% | 0.0% | 0.0% | 0.0% | 0.0% | 0.0% | 0.0% | 0.0% | 0.0% | 0.0% | 0.0% | 0.0% | 0.0% | 0.0% | 0.0% | 0.0% | 0.2% | 0.0% | 0.0% | 0.0% |
|  | k\_\_Bacteria;p\_\_Proteobacteria;c\_\_Betaproteobacteria;o\_\_MND1;f\_\_Unclassified\_MND1;g\_\_Unclassified\_MND1 | 0 | 0.0% | 0.0% | 0.0% | 0.0% | 0.0% | 0.0% | 0.0% | 0.0% | 0.0% | 0.0% | 0.0% | 0.0% | 0.0% | 0.0% | 0.0% | 0.0% | 0.1% | 0.0% | 0.0% | 0.0% | 0.0% | 0.2% | 0.0% | 0.0% |
|  | k\_\_Bacteria;p\_\_Lentisphaerae;c\_\_[Lentisphaeria];o\_\_Unclassified\_[Lentisphaeria];f\_\_Unclassified\_[Lentisphaeria];g\_\_Unclassified\_[Lentisphaeria] | 0 | 0.0% | 0.0% | 0.0% | 0.0% | 0.0% | 0.0% | 0.0% | 0.0% | 0.0% | 0.0% | 0.0% | 0.0% | 0.1% | 0.0% | 0.0% | 0.0% | 0.0% | 0.0% | 0.0% | 0.0% | 0.2% | 0.0% | 0.0% | 0.0% |
|  | k\_\_Bacteria;p\_\_Firmicutes;c\_\_Bacilli;o\_\_Gemellales;f\_\_Gemellaceae;g\_\_Gemella | 0 | 0.0% | 0.2% | 0.0% | 0.0% | 0.0% | 0.0% | 0.0% | 0.0% | 0.0% | 0.0% | 0.0% | 0.0% | 0.0% | 0.0% | 0.0% | 0.0% | 0.0% | 0.0% | 0.0% | 0.0% | 0.0% | 0.0% | 0.0% | 0.0% |
|  | k\_\_Bacteria;p\_\_Chlamydiae;c\_\_Chlamydiia;o\_\_Chlamydiales;f\_\_Rhabdochlamydiaceae;g\_\_Candidatus\_Rhabdochlamydia | 0 | 0.0% | 0.0% | 0.0% | 0.0% | 0.0% | 0.0% | 0.0% | 0.0% | 0.0% | 0.0% | 0.0% | 0.0% | 0.0% | 0.0% | 0.0% | 0.1% | 0.0% | 0.0% | 0.0% | 0.0% | 0.0% | 0.0% | 0.0% | 0.0% |
|  | k\_\_Bacteria;p\_\_Firmicutes;c\_\_Bacilli;o\_\_Bacillales;f\_\_Unclassified\_Bacillales;g\_\_Unclassified\_Bacillales | 0 | 0.0% | 0.0% | 0.0% | 0.0% | 0.1% | 0.0% | 0.0% | 0.0% | 0.0% | 0.0% | 0.0% | 0.0% | 0.0% | 0.0% | 0.0% | 0.0% | 0.1% | 0.0% | 0.0% | 0.0% | 0.0% | 0.0% | 0.0% | 0.0% |
|  | k\_\_Bacteria;p\_\_Actinobacteria;c\_\_Actinobacteria;o\_\_Actinomycetales;f\_\_Microbacteriaceae;g\_\_Salinibacterium | 0 | 0.0% | 0.0% | 0.0% | 0.0% | 0.0% | 0.0% | 0.0% | 0.0% | 0.0% | 0.0% | 0.0% | 0.1% | 0.0% | 0.0% | 0.0% | 0.0% | 0.0% | 0.0% | 0.0% | 0.0% | 0.0% | 0.1% | 0.0% | 0.0% |
|  | k\_\_Bacteria;p\_\_Actinobacteria;c\_\_Thermoleophilia;o\_\_Gaiellales;f\_\_Gaiellaceae;g\_\_Unclassified\_Gaiellaceae | 0 | 0.0% | 0.0% | 0.0% | 0.2% | 0.0% | 0.0% | 0.0% | 0.0% | 0.0% | 0.0% | 0.0% | 0.0% | 0.0% | 0.0% | 0.0% | 0.0% | 0.0% | 0.0% | 0.0% | 0.0% | 0.0% | 0.0% | 0.0% | 0.0% |
|  | k\_\_Bacteria;p\_\_Firmicutes;c\_\_Bacilli;o\_\_Bacillales;f\_\_Staphylococcaceae;g\_\_Jeotgalicoccus | 0 | 0.0% | 0.0% | 0.0% | 0.0% | 0.0% | 0.0% | 0.0% | 0.0% | 0.0% | 0.0% | 0.0% | 0.0% | 0.0% | 0.0% | 0.0% | 0.0% | 0.0% | 0.1% | 0.0% | 0.0% | 0.0% | 0.0% | 0.0% | 0.0% |
|  | k\_\_Bacteria;p\_\_TM7;c\_\_TM7-3;o\_\_Unclassified\_TM7-3;f\_\_Unclassified\_TM7-3;g\_\_Unclassified\_TM7-3 | 0 | 0.0% | 0.0% | 0.0% | 0.0% | 0.0% | 0.0% | 0.0% | 0.0% | 0.0% | 0.0% | 0.0% | 0.0% | 0.0% | 0.0% | 0.1% | 0.0% | 0.0% | 0.0% | 0.0% | 0.2% | 0.0% | 0.0% | 0.0% | 0.0% |
|  | k\_\_Bacteria;p\_\_Proteobacteria;c\_\_Deltaproteobacteria;o\_\_MIZ46;f\_\_Unclassified\_MIZ46;g\_\_Unclassified\_MIZ46 | 0 | 0.0% | 0.0% | 0.0% | 0.0% | 0.0% | 0.0% | 0.0% | 0.0% | 0.0% | 0.0% | 0.0% | 0.0% | 0.0% | 0.0% | 0.0% | 0.1% | 0.0% | 0.0% | 0.0% | 0.0% | 0.0% | 0.0% | 0.0% | 0.0% |
|  | k\_\_Bacteria;p\_\_Bacteroidetes;c\_\_Sphingobacteriia;o\_\_Sphingobacteriales;f\_\_Unclassified\_Sphingobacteriales;g\_\_Unclassified\_Sphingobacteriales | 0 | 0.0% | 0.0% | 0.0% | 0.0% | 0.1% | 0.0% | 0.0% | 0.0% | 0.0% | 0.0% | 0.0% | 0.0% | 0.0% | 0.0% | 0.0% | 0.0% | 0.0% | 0.0% | 0.0% | 0.0% | 0.0% | 0.0% | 0.0% | 0.0% |
|  | k\_\_Bacteria;p\_\_Proteobacteria;c\_\_Deltaproteobacteria;o\_\_Myxococcales;f\_\_Haliangiaceae;g\_\_Unclassified\_Haliangiaceae | 0 | 0.0% | 0.0% | 0.0% | 0.1% | 0.0% | 0.0% | 0.0% | 0.0% | 0.0% | 0.0% | 0.0% | 0.0% | 0.0% | 0.0% | 0.0% | 0.0% | 0.0% | 0.0% | 0.0% | 0.0% | 0.0% | 0.1% | 0.0% | 0.0% |
|  | k\_\_Bacteria;p\_\_Gemmatimonadetes;c\_\_Gemmatimonadetes;o\_\_N1423WL;f\_\_Unclassified\_N1423WL;g\_\_Unclassified\_N1423WL | 0 | 0.0% | 0.0% | 0.0% | 0.0% | 0.0% | 0.0% | 0.0% | 0.0% | 0.0% | 0.0% | 0.0% | 0.0% | 0.0% | 0.0% | 0.0% | 0.0% | 0.0% | 0.0% | 0.0% | 0.0% | 0.0% | 0.2% | 0.0% | 0.0% |
|  | k\_\_Bacteria;p\_\_Proteobacteria;c\_\_Deltaproteobacteria;o\_\_Syntrophobacterales;f\_\_Syntrophobacteraceae;g\_\_Unclassified\_Syntrophobacteraceae | 0 | 0.0% | 0.0% | 0.0% | 0.0% | 0.0% | 0.0% | 0.0% | 0.0% | 0.0% | 0.0% | 0.0% | 0.0% | 0.0% | 0.0% | 0.0% | 0.0% | 0.1% | 0.0% | 0.0% | 0.0% | 0.0% | 0.1% | 0.0% | 0.0% |
|  | k\_\_Bacteria;p\_\_Proteobacteria;c\_\_Alphaproteobacteria;o\_\_Rhodobacterales;f\_\_Rhodobacteraceae;g\_\_Paracoccus | 0 | 0.0% | 0.0% | 0.0% | 0.0% | 0.0% | 0.0% | 0.0% | 0.0% | 0.0% | 0.0% | 0.0% | 0.0% | 0.0% | 0.0% | 0.0% | 0.0% | 0.0% | 0.0% | 0.0% | 0.0% | 0.0% | 0.0% | 0.0% | 0.0% |
|  | k\_\_Bacteria;p\_\_Firmicutes;c\_\_Bacilli;o\_\_Lactobacillales;f\_\_Lactobacillaceae;g\_\_Unclassified\_Lactobacillaceae | 0 | 0.0% | 0.0% | 0.0% | 0.0% | 0.0% | 0.0% | 0.0% | 0.0% | 0.0% | 0.0% | 0.0% | 0.0% | 0.0% | 0.0% | 0.0% | 0.0% | 0.2% | 0.0% | 0.0% | 0.0% | 0.0% | 0.0% | 0.0% | 0.0% |
|  | k\_\_Bacteria;p\_\_Bacteroidetes;c\_\_Bacteroidia;o\_\_Bacteroidales;f\_\_[Paraprevotellaceae];g\_\_CF231 | 0 | 0.0% | 0.0% | 0.0% | 0.0% | 0.0% | 0.0% | 0.0% | 0.0% | 0.0% | 0.0% | 0.0% | 0.0% | 0.0% | 0.0% | 0.0% | 0.0% | 0.0% | 0.0% | 0.0% | 0.0% | 0.1% | 0.1% | 0.0% | 0.0% |
|  | k\_\_Bacteria;p\_\_Proteobacteria;c\_\_Deltaproteobacteria;o\_\_Bdellovibrionales;f\_\_Bdellovibrionaceae;g\_\_Bdellovibrio | 0 | 0.0% | 0.0% | 0.0% | 0.0% | 0.0% | 0.0% | 0.0% | 0.0% | 0.0% | 0.0% | 0.0% | 0.0% | 0.0% | 0.0% | 0.0% | 0.0% | 0.1% | 0.0% | 0.0% | 0.0% | 0.0% | 0.0% | 0.0% | 0.0% |
|  | k\_\_Bacteria;p\_\_Bacteroidetes;c\_\_Flavobacteriia;o\_\_Flavobacteriales;f\_\_[Weeksellaceae];g\_\_Chryseobacterium | 0 | 0.0% | 0.0% | 0.0% | 0.1% | 0.0% | 0.0% | 0.0% | 0.0% | 0.0% | 0.0% | 0.0% | 0.0% | 0.0% | 0.0% | 0.1% | 0.0% | 0.0% | 0.0% | 0.0% | 0.0% | 0.0% | 0.0% | 0.0% | 0.0% |
|  | k\_\_Bacteria;p\_\_Proteobacteria;c\_\_Gammaproteobacteria;o\_\_Xanthomonadales;f\_\_Xanthomonadaceae;g\_\_Lysobacter | 0 | 0.0% | 0.0% | 0.0% | 0.0% | 0.0% | 0.0% | 0.0% | 0.0% | 0.0% | 0.0% | 0.0% | 0.0% | 0.0% | 0.0% | 0.0% | 0.0% | 0.0% | 0.1% | 0.0% | 0.0% | 0.0% | 0.0% | 0.0% | 0.0% |
|  | k\_\_Bacteria;p\_\_Tenericutes;c\_\_RF3;o\_\_ML615J-28;f\_\_Unclassified\_ML615J-28;g\_\_Unclassified\_ML615J-28 | 0 | 0.0% | 0.0% | 0.0% | 0.0% | 0.0% | 0.0% | 0.0% | 0.0% | 0.0% | 0.0% | 0.0% | 0.0% | 0.0% | 0.0% | 0.0% | 0.0% | 0.0% | 0.0% | 0.0% | 0.0% | 0.1% | 0.1% | 0.0% | 0.0% |
|  | k\_\_Bacteria;p\_\_Chloroflexi;c\_\_Anaerolineae;o\_\_H39;f\_\_Unclassified\_H39;g\_\_Unclassified\_H39 | 0 | 0.0% | 0.0% | 0.0% | 0.0% | 0.0% | 0.0% | 0.0% | 0.0% | 0.0% | 0.0% | 0.0% | 0.0% | 0.0% | 0.0% | 0.0% | 0.0% | 0.2% | 0.0% | 0.0% | 0.0% | 0.0% | 0.0% | 0.0% | 0.0% |
|  | k\_\_Bacteria;p\_\_Proteobacteria;c\_\_Gammaproteobacteria;o\_\_Enterobacteriales;f\_\_Enterobacteriaceae;g\_\_Proteus | 0 | 0.0% | 0.0% | 0.0% | 0.0% | 0.0% | 0.0% | 0.0% | 0.0% | 0.0% | 0.0% | 0.0% | 0.0% | 0.0% | 0.0% | 0.0% | 0.0% | 0.2% | 0.0% | 0.0% | 0.0% | 0.0% | 0.0% | 0.0% | 0.0% |
|  | k\_\_Bacteria;p\_\_Proteobacteria;c\_\_Alphaproteobacteria;o\_\_Rhodobacterales;f\_\_Hyphomonadaceae;g\_\_Unclassified\_Hyphomonadaceae | 0 | 0.0% | 0.0% | 0.0% | 0.0% | 0.0% | 0.0% | 0.0% | 0.0% | 0.0% | 0.0% | 0.0% | 0.0% | 0.0% | 0.0% | 0.0% | 0.0% | 0.0% | 0.0% | 0.0% | 0.0% | 0.0% | 0.0% | 0.0% | 0.1% |
|  | k\_\_Bacteria;p\_\_Actinobacteria;c\_\_Actinobacteria;o\_\_Actinomycetales;f\_\_Micrococcaceae;g\_\_Micrococcus | 0 | 0.0% | 0.0% | 0.0% | 0.0% | 0.1% | 0.0% | 0.0% | 0.0% | 0.0% | 0.0% | 0.0% | 0.0% | 0.0% | 0.0% | 0.0% | 0.0% | 0.0% | 0.0% | 0.0% | 0.0% | 0.0% | 0.0% | 0.0% | 0.0% |
|  | k\_\_Bacteria;p\_\_Proteobacteria;c\_\_Alphaproteobacteria;o\_\_Sphingomonadales;f\_\_Sphingomonadaceae;g\_\_Kaistobacter | 0 | 0.0% | 0.0% | 0.0% | 0.0% | 0.0% | 0.0% | 0.0% | 0.0% | 0.0% | 0.0% | 0.0% | 0.0% | 0.0% | 0.0% | 0.0% | 0.0% | 0.0% | 0.0% | 0.0% | 0.0% | 0.0% | 0.1% | 0.0% | 0.0% |
|  | k\_\_Bacteria;p\_\_Proteobacteria;c\_\_Betaproteobacteria;o\_\_Unclassified\_Betaproteobacteria;f\_\_Unclassified\_Betaproteobacteria;g\_\_Unclassified\_Betaproteobacteria | 0 | 0.0% | 0.0% | 0.0% | 0.0% | 0.0% | 0.0% | 0.0% | 0.0% | 0.0% | 0.0% | 0.0% | 0.0% | 0.0% | 0.0% | 0.0% | 0.0% | 0.1% | 0.0% | 0.0% | 0.0% | 0.0% | 0.0% | 0.0% | 0.0% |
|  | k\_\_Bacteria;p\_\_Bacteroidetes;c\_\_Bacteroidia;o\_\_Bacteroidales;f\_\_[Paraprevotellaceae];g\_\_[Prevotella] | 0 | 0.0% | 0.0% | 0.0% | 0.0% | 0.0% | 0.0% | 0.0% | 0.0% | 0.0% | 0.0% | 0.0% | 0.0% | 0.0% | 0.0% | 0.0% | 0.0% | 0.1% | 0.0% | 0.0% | 0.0% | 0.0% | 0.0% | 0.0% | 0.0% |
|  | k\_\_Bacteria;p\_\_Armatimonadetes;c\_\_[Fimbriimonadia];o\_\_[Fimbriimonadales];f\_\_[Fimbriimonadaceae];g\_\_Fimbriimonas | 0 | 0.0% | 0.0% | 0.0% | 0.0% | 0.0% | 0.0% | 0.0% | 0.0% | 0.0% | 0.0% | 0.0% | 0.0% | 0.0% | 0.0% | 0.0% | 0.0% | 0.0% | 0.0% | 0.0% | 0.0% | 0.0% | 0.0% | 0.0% | 0.0% |
|  | k\_\_Bacteria;p\_\_Chloroflexi;c\_\_Anaerolineae;o\_\_SBR1031;f\_\_SHA-31;g\_\_Unclassified\_SHA-31 | 0 | 0.0% | 0.0% | 0.0% | 0.1% | 0.1% | 0.0% | 0.0% | 0.0% | 0.0% | 0.0% | 0.0% | 0.0% | 0.0% | 0.0% | 0.0% | 0.1% | 0.0% | 0.0% | 0.0% | 0.0% | 0.0% | 0.0% | 0.0% | 0.0% |
|  | k\_\_Bacteria;p\_\_Proteobacteria;c\_\_Alphaproteobacteria;o\_\_Sphingomonadales;f\_\_Erythrobacteraceae;g\_\_Unclassified\_Erythrobacteraceae | 0 | 0.0% | 0.0% | 0.0% | 0.0% | 0.0% | 0.0% | 0.0% | 0.0% | 0.0% | 0.0% | 0.0% | 0.0% | 0.0% | 0.0% | 0.0% | 0.0% | 0.1% | 0.1% | 0.0% | 0.0% | 0.0% | 0.0% | 0.0% | 0.0% |
|  | k\_\_Bacteria;p\_\_Proteobacteria;c\_\_Alphaproteobacteria;o\_\_Caulobacterales;f\_\_Caulobacteraceae;g\_\_Asticcacaulis | 0 | 0.0% | 0.0% | 0.0% | 0.0% | 0.0% | 0.0% | 0.0% | 0.0% | 0.0% | 0.0% | 0.0% | 0.0% | 0.0% | 0.0% | 0.0% | 0.0% | 0.0% | 0.0% | 0.0% | 0.0% | 0.0% | 0.0% | 0.0% | 0.0% |
|  | k\_\_Bacteria;p\_\_Gemmatimonadetes;c\_\_Gemmatimonadetes;o\_\_Gemmatimonadales;f\_\_Ellin5301;g\_\_Unclassified\_Ellin5301 | 0 | 0.0% | 0.0% | 0.0% | 0.0% | 0.0% | 0.0% | 0.0% | 0.0% | 0.0% | 0.0% | 0.0% | 0.0% | 0.0% | 0.0% | 0.0% | 0.0% | 0.0% | 0.0% | 0.0% | 0.0% | 0.0% | 0.2% | 0.0% | 0.0% |
|  | k\_\_Bacteria;p\_\_Nitrospirae;c\_\_Nitrospira;o\_\_Nitrospirales;f\_\_0319-6A21;g\_\_Unclassified\_0319-6A21 | 0 | 0.0% | 0.0% | 0.0% | 0.0% | 0.0% | 0.0% | 0.0% | 0.0% | 0.0% | 0.0% | 0.0% | 0.0% | 0.0% | 0.0% | 0.1% | 0.0% | 0.0% | 0.0% | 0.0% | 0.0% | 0.0% | 0.1% | 0.0% | 0.0% |
|  | k\_\_Bacteria;p\_\_WPS-2;c\_\_Unclassified\_WPS-2;o\_\_Unclassified\_WPS-2;f\_\_Unclassified\_WPS-2;g\_\_Unclassified\_WPS-2 | 0 | 0.0% | 0.0% | 0.0% | 0.0% | 0.0% | 0.0% | 0.0% | 0.0% | 0.0% | 0.0% | 0.0% | 0.0% | 0.0% | 0.0% | 0.0% | 0.0% | 0.0% | 0.0% | 0.0% | 0.0% | 0.0% | 0.0% | 0.0% | 0.0% |
|  | k\_\_Bacteria;p\_\_Proteobacteria;c\_\_Betaproteobacteria;o\_\_Rhodocyclales;f\_\_Rhodocyclaceae;g\_\_Petrobacter | 0 | 0.0% | 0.0% | 0.0% | 0.0% | 0.0% | 0.0% | 0.0% | 0.0% | 0.0% | 0.0% | 0.0% | 0.0% | 0.0% | 0.0% | 0.0% | 0.0% | 0.0% | 0.0% | 0.0% | 0.0% | 0.0% | 0.2% | 0.0% | 0.0% |
|  | k\_\_Bacteria;p\_\_Actinobacteria;c\_\_Coriobacteriia;o\_\_Coriobacteriales;f\_\_Coriobacteriaceae;g\_\_Adlercreutzia | 0 | 0.0% | 0.0% | 0.0% | 0.0% | 0.0% | 0.0% | 0.0% | 0.0% | 0.0% | 0.0% | 0.0% | 0.0% | 0.0% | 0.0% | 0.0% | 0.0% | 0.0% | 0.0% | 0.0% | 0.0% | 0.0% | 0.1% | 0.0% | 0.0% |
|  | k\_\_Bacteria;p\_\_Actinobacteria;c\_\_Actinobacteria;o\_\_Actinomycetales;f\_\_Micrococcaceae;g\_\_Kocuria | 0 | 0.0% | 0.0% | 0.0% | 0.0% | 0.0% | 0.0% | 0.0% | 0.0% | 0.0% | 0.0% | 0.0% | 0.0% | 0.0% | 0.0% | 0.0% | 0.0% | 0.1% | 0.0% | 0.0% | 0.0% | 0.0% | 0.0% | 0.0% | 0.0% |
|  | k\_\_Bacteria;p\_\_WS3;c\_\_PRR-12;o\_\_Sediment-1;f\_\_Unclassified\_Sediment-1;g\_\_Unclassified\_Sediment-1 | 0 | 0.0% | 0.0% | 0.0% | 0.1% | 0.0% | 0.0% | 0.0% | 0.0% | 0.0% | 0.0% | 0.0% | 0.0% | 0.0% | 0.0% | 0.0% | 0.0% | 0.0% | 0.0% | 0.0% | 0.0% | 0.0% | 0.0% | 0.0% | 0.0% |
|  | k\_\_Bacteria;p\_\_Chloroflexi;c\_\_S085;o\_\_Unclassified\_S085;f\_\_Unclassified\_S085;g\_\_Unclassified\_S085 | 0 | 0.0% | 0.0% | 0.0% | 0.1% | 0.0% | 0.0% | 0.0% | 0.0% | 0.0% | 0.0% | 0.0% | 0.0% | 0.0% | 0.0% | 0.0% | 0.1% | 0.0% | 0.0% | 0.0% | 0.0% | 0.0% | 0.1% | 0.0% | 0.0% |
|  | k\_\_Bacteria;p\_\_Actinobacteria;c\_\_Thermoleophilia;o\_\_Solirubrobacterales;f\_\_Unclassified\_Solirubrobacterales;g\_\_Unclassified\_Solirubrobacterales | 0 | 0.0% | 0.0% | 0.0% | 0.0% | 0.1% | 0.0% | 0.0% | 0.0% | 0.0% | 0.0% | 0.0% | 0.0% | 0.0% | 0.0% | 0.0% | 0.0% | 0.0% | 0.0% | 0.0% | 0.0% | 0.0% | 0.0% | 0.0% | 0.0% |
|  | k\_\_Bacteria;p\_\_OD1;c\_\_ZB2;o\_\_Unclassified\_ZB2;f\_\_Unclassified\_ZB2;g\_\_Unclassified\_ZB2 | 0 | 0.0% | 0.0% | 0.0% | 0.0% | 0.0% | 0.0% | 0.0% | 0.0% | 0.0% | 0.0% | 0.0% | 0.0% | 0.0% | 0.0% | 0.0% | 0.0% | 0.0% | 0.0% | 0.0% | 0.0% | 0.0% | 0.0% | 0.0% | 0.0% |
|  | k\_\_Bacteria;p\_\_Planctomycetes;c\_\_Planctomycetia;o\_\_Gemmatales;f\_\_Isosphaeraceae;g\_\_Unclassified\_Isosphaeraceae | 0 | 0.0% | 0.0% | 0.0% | 0.0% | 0.0% | 0.0% | 0.0% | 0.0% | 0.0% | 0.0% | 0.0% | 0.1% | 0.0% | 0.0% | 0.0% | 0.0% | 0.0% | 0.0% | 0.0% | 0.0% | 0.0% | 0.0% | 0.0% | 0.0% |
|  | k\_\_Bacteria;p\_\_Proteobacteria;c\_\_Betaproteobacteria;o\_\_Hydrogenophilales;f\_\_Hydrogenophilaceae;g\_\_Thiobacillus | 0 | 0.0% | 0.0% | 0.0% | 0.0% | 0.0% | 0.0% | 0.0% | 0.0% | 0.0% | 0.0% | 0.0% | 0.0% | 0.0% | 0.0% | 0.0% | 0.0% | 0.0% | 0.0% | 0.0% | 0.0% | 0.0% | 0.0% | 0.0% | 0.2% |
|  | k\_\_Bacteria;p\_\_Proteobacteria;c\_\_Alphaproteobacteria;o\_\_Rickettsiales;f\_\_mitochondria;g\_\_Zea | 0 | 0.0% | 0.0% | 0.0% | 0.0% | 0.1% | 0.0% | 0.0% | 0.0% | 0.0% | 0.0% | 0.0% | 0.0% | 0.0% | 0.0% | 0.0% | 0.0% | 0.0% | 0.0% | 0.0% | 0.0% | 0.0% | 0.0% | 0.0% | 0.0% |
|  | k\_\_Bacteria;p\_\_Proteobacteria;c\_\_Gammaproteobacteria;o\_\_Pseudomonadales;f\_\_Moraxellaceae;g\_\_Unclassified\_Moraxellaceae | 0 | 0.0% | 0.0% | 0.0% | 0.0% | 0.0% | 0.0% | 0.0% | 0.0% | 0.0% | 0.0% | 0.0% | 0.0% | 0.0% | 0.0% | 0.0% | 0.0% | 0.0% | 0.0% | 0.0% | 0.0% | 0.0% | 0.0% | 0.0% | 0.0% |
|  | k\_\_Bacteria;p\_\_Proteobacteria;c\_\_Deltaproteobacteria;o\_\_GMD14H09;f\_\_Unclassified\_GMD14H09;g\_\_Unclassified\_GMD14H09 | 0 | 0.0% | 0.0% | 0.0% | 0.0% | 0.0% | 0.0% | 0.0% | 0.0% | 0.0% | 0.0% | 0.0% | 0.0% | 0.0% | 0.0% | 0.0% | 0.0% | 0.0% | 0.0% | 0.1% | 0.0% | 0.0% | 0.0% | 0.0% | 0.1% |
|  | k\_\_Bacteria;p\_\_Gemmatimonadetes;c\_\_Gemmatimonadetes;o\_\_Gemmatimonadales;f\_\_Unclassified\_Gemmatimonadales;g\_\_Unclassified\_Gemmatimonadales | 0 | 0.0% | 0.0% | 0.0% | 0.0% | 0.0% | 0.0% | 0.0% | 0.0% | 0.0% | 0.0% | 0.0% | 0.0% | 0.0% | 0.0% | 0.0% | 0.1% | 0.0% | 0.0% | 0.0% | 0.0% | 0.0% | 0.1% | 0.0% | 0.0% |
|  | k\_\_Bacteria;p\_\_Proteobacteria;c\_\_Betaproteobacteria;o\_\_Burkholderiales;f\_\_Alcaligenaceae;g\_\_Sutterella | 0 | 0.0% | 0.0% | 0.0% | 0.0% | 0.0% | 0.0% | 0.0% | 0.0% | 0.0% | 0.0% | 0.0% | 0.0% | 0.0% | 0.0% | 0.0% | 0.0% | 0.1% | 0.0% | 0.0% | 0.0% | 0.0% | 0.1% | 0.0% | 0.0% |
|  | k\_\_Bacteria;p\_\_Firmicutes;c\_\_Bacilli;o\_\_Bacillales;f\_\_Staphylococcaceae;g\_\_Salinicoccus | 0 | 0.0% | 0.0% | 0.0% | 0.1% | 0.0% | 0.0% | 0.0% | 0.0% | 0.0% | 0.0% | 0.0% | 0.0% | 0.0% | 0.0% | 0.0% | 0.0% | 0.0% | 0.0% | 0.0% | 0.0% | 0.0% | 0.0% | 0.0% | 0.0% |
|  | k\_\_Bacteria;p\_\_Planctomycetes;c\_\_Planctomycetia;o\_\_Gemmatales;f\_\_Gemmataceae;g\_\_Unclassified\_Gemmataceae | 0 | 0.0% | 0.0% | 0.0% | 0.1% | 0.0% | 0.0% | 0.0% | 0.0% | 0.0% | 0.0% | 0.0% | 0.0% | 0.0% | 0.0% | 0.0% | 0.0% | 0.0% | 0.0% | 0.0% | 0.0% | 0.0% | 0.0% | 0.0% | 0.0% |
|  | k\_\_Bacteria;p\_\_Chloroflexi;c\_\_Chloroflexi;o\_\_[Roseiflexales];f\_\_[Kouleothrixaceae];g\_\_Unclassified\_[Kouleothrixaceae] | 0 | 0.0% | 0.0% | 0.0% | 0.1% | 0.0% | 0.0% | 0.0% | 0.0% | 0.0% | 0.0% | 0.0% | 0.0% | 0.0% | 0.0% | 0.0% | 0.0% | 0.0% | 0.0% | 0.0% | 0.0% | 0.0% | 0.0% | 0.0% | 0.0% |
|  | k\_\_Bacteria;p\_\_Proteobacteria;c\_\_Betaproteobacteria;o\_\_Burkholderiales;f\_\_Comamonadaceae;g\_\_Methylibium | 0 | 0.0% | 0.0% | 0.0% | 0.0% | 0.0% | 0.0% | 0.0% | 0.0% | 0.0% | 0.0% | 0.0% | 0.0% | 0.0% | 0.0% | 0.0% | 0.0% | 0.0% | 0.0% | 0.0% | 0.0% | 0.0% | 0.0% | 0.0% | 0.0% |
|  | k\_\_Bacteria;p\_\_Bacteroidetes;c\_\_Bacteroidia;o\_\_Bacteroidales;f\_\_BS11;g\_\_Unclassified\_BS11 | 0 | 0.0% | 0.0% | 0.0% | 0.0% | 0.0% | 0.0% | 0.0% | 0.0% | 0.0% | 0.0% | 0.0% | 0.0% | 0.0% | 0.0% | 0.0% | 0.0% | 0.0% | 0.0% | 0.0% | 0.0% | 0.0% | 0.0% | 0.0% | 0.0% |
|  | k\_\_Bacteria;p\_\_Proteobacteria;c\_\_Alphaproteobacteria;o\_\_Rhodobacterales;f\_\_Rhodobacteraceae;g\_\_Rhodobacter | 0 | 0.0% | 0.0% | 0.0% | 0.0% | 0.0% | 0.0% | 0.0% | 0.0% | 0.0% | 0.0% | 0.0% | 0.0% | 0.0% | 0.0% | 0.0% | 0.0% | 0.1% | 0.0% | 0.0% | 0.0% | 0.0% | 0.0% | 0.0% | 0.0% |
|  | k\_\_Bacteria;p\_\_Acidobacteria;c\_\_[Chloracidobacteria];o\_\_RB41;f\_\_Unclassified\_RB41;g\_\_Unclassified\_RB41 | 0 | 0.0% | 0.0% | 0.0% | 0.0% | 0.0% | 0.0% | 0.0% | 0.0% | 0.0% | 0.0% | 0.0% | 0.0% | 0.0% | 0.0% | 0.0% | 0.0% | 0.0% | 0.0% | 0.0% | 0.0% | 0.0% | 0.1% | 0.0% | 0.0% |
|  | k\_\_Bacteria;p\_\_Actinobacteria;c\_\_Actinobacteria;o\_\_Actinomycetales;f\_\_Dermabacteraceae;g\_\_Brachybacterium | 0 | 0.0% | 0.0% | 0.0% | 0.0% | 0.0% | 0.0% | 0.0% | 0.0% | 0.0% | 0.0% | 0.0% | 0.0% | 0.0% | 0.0% | 0.0% | 0.0% | 0.0% | 0.0% | 0.0% | 0.0% | 0.0% | 0.0% | 0.0% | 0.0% |
|  | k\_\_Bacteria;p\_\_[Thermi];c\_\_Deinococci;o\_\_Thermales;f\_\_Thermaceae;g\_\_Thermus | 0 | 0.0% | 0.0% | 0.0% | 0.0% | 0.0% | 0.0% | 0.0% | 0.0% | 0.0% | 0.1% | 0.0% | 0.0% | 0.0% | 0.0% | 0.0% | 0.0% | 0.0% | 0.0% | 0.0% | 0.0% | 0.0% | 0.0% | 0.0% | 0.0% |
|  | k\_\_Bacteria;p\_\_Armatimonadetes;c\_\_Chthonomonadetes;o\_\_Chthonomonadales;f\_\_Chthonomonadaceae;g\_\_Chthonomonas | 0 | 0.0% | 0.0% | 0.0% | 0.1% | 0.0% | 0.0% | 0.0% | 0.0% | 0.0% | 0.0% | 0.0% | 0.0% | 0.0% | 0.0% | 0.0% | 0.0% | 0.0% | 0.0% | 0.0% | 0.0% | 0.0% | 0.0% | 0.0% | 0.0% |
|  | k\_\_Bacteria;p\_\_AD3;c\_\_ABS-6;o\_\_Unclassified\_ABS-6;f\_\_Unclassified\_ABS-6;g\_\_Unclassified\_ABS-6 | 0 | 0.0% | 0.0% | 0.0% | 0.0% | 0.0% | 0.0% | 0.0% | 0.0% | 0.0% | 0.0% | 0.0% | 0.0% | 0.0% | 0.0% | 0.0% | 0.0% | 0.1% | 0.0% | 0.0% | 0.0% | 0.0% | 0.0% | 0.0% | 0.0% |
|  | k\_\_Bacteria;p\_\_Proteobacteria;c\_\_Gammaproteobacteria;o\_\_Thiotrichales;f\_\_Piscirickettsiaceae;g\_\_Unclassified\_Piscirickettsiaceae | 0 | 0.0% | 0.0% | 0.0% | 0.0% | 0.0% | 0.0% | 0.0% | 0.0% | 0.0% | 0.0% | 0.0% | 0.0% | 0.0% | 0.0% | 0.0% | 0.0% | 0.1% | 0.0% | 0.0% | 0.0% | 0.0% | 0.0% | 0.0% | 0.0% |
|  | k\_\_Bacteria;p\_\_Actinobacteria;c\_\_Actinobacteria;o\_\_Actinomycetales;f\_\_Geodermatophilaceae;g\_\_Geodermatophilus | 0 | 0.0% | 0.0% | 0.0% | 0.0% | 0.0% | 0.0% | 0.0% | 0.0% | 0.0% | 0.0% | 0.0% | 0.0% | 0.0% | 0.0% | 0.0% | 0.1% | 0.0% | 0.0% | 0.0% | 0.0% | 0.0% | 0.0% | 0.0% | 0.0% |
|  | k\_\_Bacteria;p\_\_Acidobacteria;c\_\_Acidobacteria-6;o\_\_CCU21;f\_\_Unclassified\_CCU21;g\_\_Unclassified\_CCU21 | 0 | 0.0% | 0.0% | 0.0% | 0.0% | 0.0% | 0.0% | 0.0% | 0.0% | 0.0% | 0.0% | 0.0% | 0.0% | 0.0% | 0.0% | 0.0% | 0.0% | 0.0% | 0.0% | 0.0% | 0.0% | 0.0% | 0.1% | 0.0% | 0.0% |
|  | k\_\_Bacteria;p\_\_Proteobacteria;c\_\_Betaproteobacteria;o\_\_Burkholderiales;f\_\_Alcaligenaceae;g\_\_Unclassified\_Alcaligenaceae | 0 | 0.0% | 0.0% | 0.0% | 0.0% | 0.0% | 0.0% | 0.0% | 0.0% | 0.0% | 0.0% | 0.0% | 0.0% | 0.0% | 0.0% | 0.0% | 0.0% | 0.0% | 0.0% | 0.0% | 0.0% | 0.0% | 0.0% | 0.0% | 0.1% |
|  | k\_\_Bacteria;p\_\_Spirochaetes;c\_\_Spirochaetes;o\_\_Sphaerochaetales;f\_\_Sphaerochaetaceae;g\_\_Sphaerochaeta | 0 | 0.0% | 0.0% | 0.0% | 0.0% | 0.0% | 0.0% | 0.1% | 0.0% | 0.0% | 0.0% | 0.0% | 0.0% | 0.0% | 0.0% | 0.0% | 0.0% | 0.0% | 0.0% | 0.0% | 0.0% | 0.0% | 0.0% | 0.0% | 0.0% |
|  | k\_\_Bacteria;p\_\_Bacteroidetes;c\_\_Sphingobacteriia;o\_\_Sphingobacteriales;f\_\_Sphingobacteriaceae;g\_\_Unclassified\_Sphingobacteriaceae | 0 | 0.0% | 0.0% | 0.0% | 0.0% | 0.0% | 0.0% | 0.0% | 0.0% | 0.0% | 0.0% | 0.0% | 0.0% | 0.0% | 0.0% | 0.0% | 0.0% | 0.0% | 0.0% | 0.0% | 0.0% | 0.0% | 0.1% | 0.0% | 0.0% |
|  | k\_\_Bacteria;p\_\_Tenericutes;c\_\_CK-1C4-19;o\_\_Unclassified\_CK-1C4-19;f\_\_Unclassified\_CK-1C4-19;g\_\_Unclassified\_CK-1C4-19 | 0 | 0.0% | 0.0% | 0.0% | 0.1% | 0.0% | 0.0% | 0.0% | 0.0% | 0.0% | 0.0% | 0.0% | 0.0% | 0.0% | 0.0% | 0.0% | 0.0% | 0.0% | 0.0% | 0.0% | 0.0% | 0.0% | 0.0% | 0.0% | 0.0% |
|  | k\_\_Bacteria;p\_\_Chloroflexi;c\_\_Thermomicrobia;o\_\_JG30-KF-CM45;f\_\_Unclassified\_JG30-KF-CM45;g\_\_Unclassified\_JG30-KF-CM45 | 0 | 0.0% | 0.0% | 0.0% | 0.0% | 0.0% | 0.0% | 0.0% | 0.0% | 0.0% | 0.0% | 0.0% | 0.0% | 0.0% | 0.0% | 0.0% | 0.0% | 0.0% | 0.0% | 0.0% | 0.0% | 0.0% | 0.1% | 0.0% | 0.0% |
|  | k\_\_Bacteria;p\_\_Actinobacteria;c\_\_Actinobacteria;o\_\_Actinomycetales;f\_\_Nocardioidaceae;g\_\_Unclassified\_Nocardioidaceae | 0 | 0.0% | 0.0% | 0.0% | 0.0% | 0.0% | 0.0% | 0.0% | 0.0% | 0.0% | 0.0% | 0.0% | 0.0% | 0.0% | 0.0% | 0.0% | 0.1% | 0.0% | 0.0% | 0.0% | 0.0% | 0.0% | 0.0% | 0.0% | 0.0% |
|  | k\_\_Bacteria;p\_\_Firmicutes;c\_\_Clostridia;o\_\_Clostridiales;f\_\_Lachnospiraceae;g\_\_Roseburia | 0 | 0.0% | 0.0% | 0.0% | 0.0% | 0.0% | 0.0% | 0.0% | 0.0% | 0.0% | 0.0% | 0.0% | 0.0% | 0.0% | 0.0% | 0.0% | 0.0% | 0.0% | 0.0% | 0.0% | 0.0% | 0.1% | 0.0% | 0.0% | 0.0% |
|  | k\_\_Bacteria;p\_\_Proteobacteria;c\_\_Betaproteobacteria;o\_\_Burkholderiales;f\_\_Burkholderiaceae;g\_\_Burkholderia | 0 | 0.0% | 0.0% | 0.0% | 0.0% | 0.0% | 0.0% | 0.0% | 0.0% | 0.0% | 0.0% | 0.0% | 0.0% | 0.0% | 0.0% | 0.0% | 0.0% | 0.0% | 0.0% | 0.0% | 0.0% | 0.0% | 0.1% | 0.0% | 0.0% |
|  | k\_\_Bacteria;p\_\_Verrucomicrobia;c\_\_Verruco-5;o\_\_WCHB1-41;f\_\_Unclassified\_WCHB1-41;g\_\_Unclassified\_WCHB1-41 | 0 | 0.0% | 0.0% | 0.0% | 0.0% | 0.0% | 0.0% | 0.0% | 0.0% | 0.0% | 0.0% | 0.0% | 0.0% | 0.0% | 0.0% | 0.0% | 0.0% | 0.1% | 0.0% | 0.0% | 0.0% | 0.0% | 0.0% | 0.0% | 0.0% |
|  | k\_\_Bacteria;p\_\_Firmicutes;c\_\_Bacilli;o\_\_Bacillales;f\_\_Planococcaceae;g\_\_Planomicrobium | 0 | 0.0% | 0.0% | 0.0% | 0.0% | 0.0% | 0.0% | 0.0% | 0.0% | 0.0% | 0.0% | 0.0% | 0.0% | 0.0% | 0.0% | 0.1% | 0.0% | 0.0% | 0.0% | 0.0% | 0.0% | 0.0% | 0.0% | 0.0% | 0.0% |
|  | k\_\_Bacteria;p\_\_Proteobacteria;c\_\_Alphaproteobacteria;o\_\_Rickettsiales;f\_\_Unclassified\_Rickettsiales;g\_\_Unclassified\_Rickettsiales | 0 | 0.0% | 0.0% | 0.0% | 0.0% | 0.0% | 0.0% | 0.0% | 0.0% | 0.0% | 0.0% | 0.0% | 0.0% | 0.0% | 0.0% | 0.0% | 0.0% | 0.1% | 0.0% | 0.0% | 0.0% | 0.0% | 0.0% | 0.0% | 0.0% |
|  | k\_\_Bacteria;p\_\_Proteobacteria;c\_\_Alphaproteobacteria;o\_\_BD7-3;f\_\_Unclassified\_BD7-3;g\_\_Unclassified\_BD7-3 | 0 | 0.0% | 0.0% | 0.0% | 0.0% | 0.0% | 0.0% | 0.0% | 0.0% | 0.0% | 0.0% | 0.0% | 0.0% | 0.0% | 0.0% | 0.0% | 0.0% | 0.0% | 0.0% | 0.0% | 0.0% | 0.0% | 0.0% | 0.0% | 0.0% |
|  | k\_\_Bacteria;p\_\_Deferribacteres;c\_\_Deferribacteres;o\_\_Deferribacterales;f\_\_Deferribacteraceae;g\_\_Mucispirillum | 0 | 0.0% | 0.0% | 0.0% | 0.0% | 0.0% | 0.0% | 0.0% | 0.0% | 0.0% | 0.0% | 0.0% | 0.0% | 0.0% | 0.0% | 0.0% | 0.0% | 0.0% | 0.0% | 0.0% | 0.0% | 0.0% | 0.0% | 0.0% | 0.0% |
|  | k\_\_Bacteria;p\_\_Firmicutes;c\_\_Clostridia;o\_\_Clostridiales;f\_\_[Tissierellaceae];g\_\_Tepidimicrobium | 0 | 0.0% | 0.0% | 0.0% | 0.0% | 0.0% | 0.0% | 0.0% | 0.0% | 0.0% | 0.0% | 0.0% | 0.0% | 0.0% | 0.0% | 0.0% | 0.0% | 0.1% | 0.0% | 0.0% | 0.0% | 0.0% | 0.0% | 0.0% | 0.0% |
|  | k\_\_Bacteria;p\_\_Proteobacteria;c\_\_Betaproteobacteria;o\_\_Ellin6067;f\_\_Unclassified\_Ellin6067;g\_\_Unclassified\_Ellin6067 | 0 | 0.0% | 0.0% | 0.0% | 0.0% | 0.0% | 0.0% | 0.0% | 0.0% | 0.0% | 0.0% | 0.0% | 0.0% | 0.0% | 0.0% | 0.0% | 0.0% | 0.0% | 0.0% | 0.0% | 0.0% | 0.0% | 0.1% | 0.0% | 0.0% |
|  | k\_\_Bacteria;p\_\_Firmicutes;c\_\_Clostridia;o\_\_Clostridiales;f\_\_Lachnospiraceae;g\_\_Butyrivibrio | 0 | 0.0% | 0.0% | 0.0% | 0.0% | 0.0% | 0.0% | 0.0% | 0.0% | 0.0% | 0.0% | 0.0% | 0.0% | 0.0% | 0.0% | 0.0% | 0.0% | 0.0% | 0.0% | 0.0% | 0.0% | 0.0% | 0.0% | 0.0% | 0.0% |
|  | k\_\_Bacteria;p\_\_Proteobacteria;c\_\_Deltaproteobacteria;o\_\_NB1-j;f\_\_NB1-i;g\_\_Unclassified\_NB1-i | 0 | 0.0% | 0.0% | 0.0% | 0.0% | 0.0% | 0.0% | 0.0% | 0.0% | 0.0% | 0.0% | 0.0% | 0.0% | 0.0% | 0.0% | 0.0% | 0.0% | 0.1% | 0.0% | 0.0% | 0.0% | 0.0% | 0.0% | 0.0% | 0.0% |
|  | k\_\_Bacteria;p\_\_Bacteroidetes;c\_\_Flavobacteriia;o\_\_Flavobacteriales;f\_\_Flavobacteriaceae;g\_\_Robiginitalea | 0 | 0.0% | 0.0% | 0.0% | 0.0% | 0.0% | 0.0% | 0.0% | 0.0% | 0.0% | 0.0% | 0.0% | 0.0% | 0.0% | 0.0% | 0.0% | 0.0% | 0.0% | 0.0% | 0.0% | 0.0% | 0.0% | 0.0% | 0.0% | 0.0% |
|  | k\_\_Bacteria;p\_\_Proteobacteria;c\_\_Betaproteobacteria;o\_\_Rhodocyclales;f\_\_Rhodocyclaceae;g\_\_Dechloromonas | 0 | 0.0% | 0.0% | 0.0% | 0.0% | 0.0% | 0.0% | 0.0% | 0.0% | 0.0% | 0.0% | 0.0% | 0.0% | 0.0% | 0.0% | 0.0% | 0.0% | 0.0% | 0.0% | 0.0% | 0.0% | 0.0% | 0.0% | 0.0% | 0.0% |
|  | k\_\_Bacteria;p\_\_Gemmatimonadetes;c\_\_Gemmatimonadetes;o\_\_Ellin5290;f\_\_Unclassified\_Ellin5290;g\_\_Unclassified\_Ellin5290 | 0 | 0.0% | 0.0% | 0.0% | 0.0% | 0.0% | 0.0% | 0.0% | 0.0% | 0.0% | 0.0% | 0.0% | 0.0% | 0.0% | 0.0% | 0.0% | 0.0% | 0.0% | 0.0% | 0.0% | 0.0% | 0.0% | 0.1% | 0.0% | 0.0% |
|  | k\_\_Bacteria;p\_\_Proteobacteria;c\_\_Deltaproteobacteria;o\_\_MBNT15;f\_\_Unclassified\_MBNT15;g\_\_Unclassified\_MBNT15 | 0 | 0.0% | 0.0% | 0.0% | 0.0% | 0.0% | 0.0% | 0.0% | 0.0% | 0.0% | 0.0% | 0.0% | 0.0% | 0.0% | 0.0% | 0.0% | 0.0% | 0.1% | 0.0% | 0.0% | 0.0% | 0.0% | 0.0% | 0.0% | 0.0% |
|  | k\_\_Bacteria;p\_\_Actinobacteria;c\_\_Actinobacteria;o\_\_Actinomycetales;f\_\_Propionibacteriaceae;g\_\_Brooklawnia | 0 | 0.0% | 0.0% | 0.0% | 0.0% | 0.0% | 0.0% | 0.0% | 0.0% | 0.0% | 0.0% | 0.0% | 0.0% | 0.0% | 0.0% | 0.0% | 0.0% | 0.0% | 0.0% | 0.0% | 0.0% | 0.0% | 0.0% | 0.0% | 0.0% |
|  | k\_\_Bacteria;p\_\_Actinobacteria;c\_\_Actinobacteria;o\_\_Actinomycetales;f\_\_Cellulomonadaceae;g\_\_Cellulomonas | 0 | 0.0% | 0.0% | 0.0% | 0.0% | 0.0% | 0.0% | 0.0% | 0.0% | 0.0% | 0.0% | 0.0% | 0.0% | 0.0% | 0.0% | 0.0% | 0.0% | 0.1% | 0.0% | 0.0% | 0.0% | 0.0% | 0.0% | 0.0% | 0.0% |
|  | k\_\_Bacteria;p\_\_Actinobacteria;c\_\_Actinobacteria;o\_\_Actinomycetales;f\_\_Geodermatophilaceae;g\_\_Unclassified\_Geodermatophilaceae | 0 | 0.0% | 0.0% | 0.0% | 0.0% | 0.0% | 0.0% | 0.0% | 0.0% | 0.0% | 0.0% | 0.0% | 0.0% | 0.0% | 0.0% | 0.0% | 0.0% | 0.0% | 0.0% | 0.0% | 0.0% | 0.0% | 0.0% | 0.0% | 0.0% |
|  | k\_\_Bacteria;p\_\_Proteobacteria;c\_\_Alphaproteobacteria;o\_\_Rhizobiales;f\_\_Hyphomicrobiaceae;g\_\_Devosia | 0 | 0.0% | 0.0% | 0.0% | 0.0% | 0.0% | 0.0% | 0.0% | 0.0% | 0.0% | 0.0% | 0.0% | 0.0% | 0.0% | 0.0% | 0.0% | 0.0% | 0.0% | 0.0% | 0.0% | 0.0% | 0.0% | 0.0% | 0.0% | 0.0% |
|  | k\_\_Bacteria;p\_\_Cyanobacteria;c\_\_Chloroplast;o\_\_Stramenopiles;f\_\_Unclassified\_Stramenopiles;g\_\_Unclassified\_Stramenopiles | 0 | 0.0% | 0.0% | 0.0% | 0.0% | 0.0% | 0.0% | 0.0% | 0.0% | 0.0% | 0.0% | 0.0% | 0.0% | 0.0% | 0.0% | 0.0% | 0.0% | 0.0% | 0.0% | 0.0% | 0.0% | 0.0% | 0.0% | 0.0% | 0.0% |
|  | k\_\_Bacteria;p\_\_Chloroflexi;c\_\_Anaerolineae;o\_\_CFB-26;f\_\_Unclassified\_CFB-26;g\_\_Unclassified\_CFB-26 | 0 | 0.0% | 0.0% | 0.0% | 0.0% | 0.0% | 0.0% | 0.0% | 0.0% | 0.0% | 0.0% | 0.0% | 0.0% | 0.0% | 0.0% | 0.0% | 0.0% | 0.0% | 0.0% | 0.0% | 0.0% | 0.0% | 0.0% | 0.0% | 0.0% |
|  | k\_\_Bacteria;p\_\_Chloroflexi;c\_\_Anaerolineae;o\_\_GCA004;f\_\_Unclassified\_GCA004;g\_\_Unclassified\_GCA004 | 0 | 0.0% | 0.0% | 0.0% | 0.0% | 0.0% | 0.0% | 0.0% | 0.0% | 0.0% | 0.0% | 0.0% | 0.0% | 0.0% | 0.0% | 0.0% | 0.0% | 0.1% | 0.0% | 0.0% | 0.0% | 0.0% | 0.0% | 0.0% | 0.0% |
|  | k\_\_Bacteria;p\_\_Firmicutes;c\_\_Bacilli;o\_\_Bacillales;f\_\_Planococcaceae;g\_\_Solibacillus | 0 | 0.0% | 0.0% | 0.0% | 0.0% | 0.0% | 0.0% | 0.0% | 0.0% | 0.0% | 0.0% | 0.0% | 0.0% | 0.0% | 0.0% | 0.0% | 0.1% | 0.0% | 0.0% | 0.0% | 0.0% | 0.0% | 0.0% | 0.0% | 0.0% |
|  | k\_\_Bacteria;p\_\_Chloroflexi;c\_\_Anaerolineae;o\_\_SBR1031;f\_\_oc28;g\_\_Unclassified\_oc28 | 0 | 0.0% | 0.0% | 0.0% | 0.0% | 0.0% | 0.0% | 0.0% | 0.0% | 0.0% | 0.0% | 0.0% | 0.0% | 0.0% | 0.0% | 0.0% | 0.0% | 0.0% | 0.0% | 0.0% | 0.0% | 0.0% | 0.0% | 0.0% | 0.0% |
|  | k\_\_Bacteria;p\_\_Nitrospirae;c\_\_Nitrospira;o\_\_Nitrospirales;f\_\_[Thermodesulfovibrionaceae];g\_\_GOUTA19 | 0 | 0.0% | 0.0% | 0.0% | 0.0% | 0.0% | 0.0% | 0.0% | 0.0% | 0.0% | 0.0% | 0.0% | 0.0% | 0.0% | 0.0% | 0.0% | 0.1% | 0.0% | 0.0% | 0.0% | 0.0% | 0.0% | 0.0% | 0.0% | 0.0% |
|  | k\_\_Bacteria;p\_\_Bacteroidetes;c\_\_Bacteroidia;o\_\_Bacteroidales;f\_\_Porphyromonadaceae;g\_\_Parabacteroides | 0 | 0.0% | 0.0% | 0.0% | 0.0% | 0.0% | 0.0% | 0.0% | 0.0% | 0.0% | 0.0% | 0.0% | 0.0% | 0.0% | 0.0% | 0.0% | 0.0% | 0.1% | 0.0% | 0.0% | 0.0% | 0.0% | 0.0% | 0.0% | 0.0% |
|  | k\_\_Bacteria;p\_\_Nitrospirae;c\_\_Nitrospira;o\_\_Nitrospirales;f\_\_Nitrospiraceae;g\_\_Nitrospira | 0 | 0.0% | 0.0% | 0.0% | 0.0% | 0.0% | 0.0% | 0.0% | 0.0% | 0.0% | 0.0% | 0.0% | 0.0% | 0.0% | 0.0% | 0.0% | 0.0% | 0.1% | 0.0% | 0.0% | 0.0% | 0.0% | 0.0% | 0.0% | 0.0% |
|  | k\_\_Bacteria;p\_\_Bacteroidetes;c\_\_Bacteroidia;o\_\_Bacteroidales;f\_\_[Barnesiellaceae];g\_\_Barnesiella | 0 | 0.0% | 0.0% | 0.0% | 0.0% | 0.0% | 0.0% | 0.0% | 0.0% | 0.0% | 0.0% | 0.0% | 0.0% | 0.0% | 0.0% | 0.0% | 0.0% | 0.1% | 0.0% | 0.0% | 0.0% | 0.0% | 0.0% | 0.0% | 0.0% |
|  | k\_\_Bacteria;p\_\_Firmicutes;c\_\_Clostridia;o\_\_Clostridiales;f\_\_Veillonellaceae;g\_\_Veillonella | 0 | 0.0% | 0.0% | 0.0% | 0.0% | 0.0% | 0.0% | 0.0% | 0.0% | 0.0% | 0.0% | 0.0% | 0.0% | 0.0% | 0.0% | 0.0% | 0.0% | 0.0% | 0.0% | 0.0% | 0.0% | 0.0% | 0.0% | 0.0% | 0.0% |
|  | k\_\_Bacteria;p\_\_Proteobacteria;c\_\_Gammaproteobacteria;o\_\_Alteromonadales;f\_\_Shewanellaceae;g\_\_Shewanella | 0 | 0.0% | 0.0% | 0.0% | 0.1% | 0.0% | 0.0% | 0.0% | 0.0% | 0.0% | 0.0% | 0.0% | 0.0% | 0.0% | 0.0% | 0.0% | 0.0% | 0.0% | 0.0% | 0.0% | 0.0% | 0.0% | 0.0% | 0.0% | 0.0% |
|  | k\_\_Bacteria;p\_\_Actinobacteria;c\_\_Acidimicrobiia;o\_\_Acidimicrobiales;f\_\_EB1017;g\_\_Unclassified\_EB1017 | 0 | 0.0% | 0.0% | 0.0% | 0.0% | 0.0% | 0.0% | 0.0% | 0.0% | 0.0% | 0.0% | 0.0% | 0.0% | 0.0% | 0.0% | 0.0% | 0.0% | 0.0% | 0.0% | 0.0% | 0.0% | 0.0% | 0.1% | 0.0% | 0.0% |
|  | k\_\_Bacteria;p\_\_Firmicutes;c\_\_Erysipelotrichi;o\_\_Erysipelotrichales;f\_\_Erysipelotrichaceae;g\_\_Catenibacterium | 0 | 0.0% | 0.0% | 0.0% | 0.0% | 0.0% | 0.0% | 0.0% | 0.0% | 0.0% | 0.0% | 0.0% | 0.0% | 0.0% | 0.0% | 0.0% | 0.0% | 0.0% | 0.0% | 0.0% | 0.0% | 0.0% | 0.0% | 0.0% | 0.0% |
|  | k\_\_Bacteria;p\_\_Proteobacteria;c\_\_Betaproteobacteria;o\_\_IS-44;f\_\_Unclassified\_IS-44;g\_\_Unclassified\_IS-44 | 0 | 0.0% | 0.0% | 0.0% | 0.0% | 0.0% | 0.0% | 0.0% | 0.0% | 0.0% | 0.0% | 0.0% | 0.0% | 0.0% | 0.0% | 0.0% | 0.0% | 0.1% | 0.0% | 0.0% | 0.0% | 0.0% | 0.0% | 0.0% | 0.0% |
|  | k\_\_Bacteria;p\_\_Acidobacteria;c\_\_DA052;o\_\_Ellin6513;f\_\_Unclassified\_Ellin6513;g\_\_Unclassified\_Ellin6513 | 0 | 0.0% | 0.0% | 0.0% | 0.0% | 0.0% | 0.0% | 0.0% | 0.0% | 0.0% | 0.0% | 0.0% | 0.0% | 0.0% | 0.0% | 0.0% | 0.0% | 0.0% | 0.0% | 0.0% | 0.0% | 0.0% | 0.0% | 0.0% | 0.0% |
|  | k\_\_Bacteria;p\_\_Proteobacteria;c\_\_Alphaproteobacteria;o\_\_Rhizobiales;f\_\_Xanthobacteraceae;g\_\_Azorhizobium | 0 | 0.0% | 0.0% | 0.0% | 0.0% | 0.0% | 0.0% | 0.0% | 0.0% | 0.0% | 0.0% | 0.0% | 0.0% | 0.0% | 0.0% | 0.0% | 0.0% | 0.0% | 0.0% | 0.0% | 0.0% | 0.0% | 0.0% | 0.0% | 0.0% |
|  | k\_\_Bacteria;p\_\_Firmicutes;c\_\_Bacilli;o\_\_Lactobacillales;f\_\_Carnobacteriaceae;g\_\_Desemzia | 0 | 0.0% | 0.0% | 0.0% | 0.0% | 0.0% | 0.0% | 0.0% | 0.0% | 0.0% | 0.0% | 0.0% | 0.0% | 0.0% | 0.0% | 0.0% | 0.0% | 0.0% | 0.0% | 0.0% | 0.0% | 0.0% | 0.0% | 0.0% | 0.0% |
|  | k\_\_Bacteria;p\_\_Chloroflexi;c\_\_Ellin6529;o\_\_Unclassified\_Ellin6529;f\_\_Unclassified\_Ellin6529;g\_\_Unclassified\_Ellin6529 | 0 | 0.0% | 0.0% | 0.0% | 0.0% | 0.0% | 0.0% | 0.0% | 0.0% | 0.0% | 0.0% | 0.0% | 0.0% | 0.0% | 0.0% | 0.0% | 0.0% | 0.0% | 0.0% | 0.0% | 0.0% | 0.0% | 0.1% | 0.0% | 0.0% |
|  | k\_\_Bacteria;p\_\_Proteobacteria;c\_\_Deltaproteobacteria;o\_\_NB1-j;f\_\_Unclassified\_NB1-j;g\_\_Unclassified\_NB1-j | 0 | 0.0% | 0.0% | 0.0% | 0.0% | 0.0% | 0.0% | 0.0% | 0.0% | 0.0% | 0.0% | 0.0% | 0.0% | 0.0% | 0.0% | 0.0% | 0.0% | 0.0% | 0.0% | 0.0% | 0.0% | 0.0% | 0.0% | 0.0% | 0.0% |
|  | k\_\_Bacteria;p\_\_Proteobacteria;c\_\_Alphaproteobacteria;o\_\_Rhodospirillales;f\_\_Rhodospirillaceae;g\_\_Telmatospirillum | 0 | 0.0% | 0.0% | 0.0% | 0.0% | 0.0% | 0.0% | 0.0% | 0.0% | 0.0% | 0.0% | 0.0% | 0.0% | 0.0% | 0.0% | 0.0% | 0.1% | 0.0% | 0.0% | 0.0% | 0.0% | 0.0% | 0.0% | 0.0% | 0.0% |
|  | k\_\_Bacteria;p\_\_Firmicutes;c\_\_Bacilli;o\_\_Bacillales;f\_\_Planococcaceae;g\_\_Lysinibacillus | 0 | 0.0% | 0.0% | 0.0% | 0.0% | 0.0% | 0.0% | 0.0% | 0.0% | 0.0% | 0.0% | 0.0% | 0.0% | 0.0% | 0.0% | 0.0% | 0.0% | 0.0% | 0.0% | 0.0% | 0.0% | 0.0% | 0.0% | 0.0% | 0.0% |
|  | k\_\_Bacteria;p\_\_Proteobacteria;c\_\_Alphaproteobacteria;o\_\_Rhodospirillales;f\_\_Acetobacteraceae;g\_\_Roseomonas | 0 | 0.0% | 0.0% | 0.0% | 0.0% | 0.0% | 0.0% | 0.0% | 0.0% | 0.0% | 0.0% | 0.0% | 0.0% | 0.0% | 0.0% | 0.1% | 0.0% | 0.0% | 0.0% | 0.0% | 0.0% | 0.0% | 0.0% | 0.0% | 0.0% |
|  | k\_\_Bacteria;p\_\_Proteobacteria;c\_\_Betaproteobacteria;o\_\_Neisseriales;f\_\_Neisseriaceae;g\_\_Neisseria | 0 | 0.0% | 0.0% | 0.0% | 0.0% | 0.0% | 0.0% | 0.0% | 0.0% | 0.0% | 0.0% | 0.0% | 0.0% | 0.0% | 0.0% | 0.0% | 0.0% | 0.0% | 0.0% | 0.0% | 0.0% | 0.0% | 0.0% | 0.0% | 0.0% |
|  | k\_\_Bacteria;p\_\_Proteobacteria;c\_\_Alphaproteobacteria;o\_\_Rhizobiales;f\_\_Hyphomicrobiaceae;g\_\_Hyphomicrobium | 0 | 0.0% | 0.0% | 0.0% | 0.0% | 0.0% | 0.0% | 0.0% | 0.0% | 0.0% | 0.0% | 0.0% | 0.0% | 0.0% | 0.0% | 0.0% | 0.0% | 0.0% | 0.0% | 0.0% | 0.0% | 0.0% | 0.0% | 0.0% | 0.0% |
|  | k\_\_Bacteria;p\_\_GAL15;c\_\_Unclassified\_GAL15;o\_\_Unclassified\_GAL15;f\_\_Unclassified\_GAL15;g\_\_Unclassified\_GAL15 | 0 | 0.0% | 0.0% | 0.0% | 0.0% | 0.0% | 0.0% | 0.0% | 0.0% | 0.0% | 0.0% | 0.0% | 0.0% | 0.0% | 0.0% | 0.0% | 0.0% | 0.1% | 0.0% | 0.0% | 0.0% | 0.0% | 0.0% | 0.0% | 0.0% |
|  | k\_\_Bacteria;p\_\_Firmicutes;c\_\_Erysipelotrichi;o\_\_Erysipelotrichales;f\_\_Erysipelotrichaceae;g\_\_[Eubacterium] | 0 | 0.0% | 0.0% | 0.0% | 0.0% | 0.0% | 0.0% | 0.0% | 0.0% | 0.0% | 0.0% | 0.0% | 0.0% | 0.0% | 0.0% | 0.0% | 0.0% | 0.1% | 0.0% | 0.0% | 0.0% | 0.0% | 0.0% | 0.0% | 0.0% |
|  | k\_\_Bacteria;p\_\_Firmicutes;c\_\_Clostridia;o\_\_Clostridiales;f\_\_[Tissierellaceae];g\_\_Parvimonas | 0 | 0.0% | 0.0% | 0.0% | 0.0% | 0.0% | 0.0% | 0.0% | 0.0% | 0.0% | 0.0% | 0.0% | 0.0% | 0.0% | 0.0% | 0.1% | 0.0% | 0.0% | 0.0% | 0.0% | 0.0% | 0.0% | 0.0% | 0.0% | 0.0% |
|  | k\_\_Bacteria;p\_\_Acidobacteria;c\_\_Solibacteres;o\_\_Solibacterales;f\_\_Solibacteraceae;g\_\_Candidatus\_Solibacter | 0 | 0.0% | 0.0% | 0.0% | 0.0% | 0.0% | 0.0% | 0.0% | 0.0% | 0.0% | 0.0% | 0.0% | 0.0% | 0.0% | 0.0% | 0.0% | 0.0% | 0.0% | 0.0% | 0.0% | 0.0% | 0.0% | 0.1% | 0.0% | 0.0% |
|  | k\_\_Bacteria;p\_\_Bacteroidetes;c\_\_Bacteroidia;o\_\_Bacteroidales;f\_\_Rikenellaceae;g\_\_Alistipes | 0 | 0.0% | 0.0% | 0.0% | 0.0% | 0.0% | 0.0% | 0.0% | 0.0% | 0.0% | 0.0% | 0.0% | 0.0% | 0.0% | 0.0% | 0.0% | 0.0% | 0.0% | 0.0% | 0.0% | 0.0% | 0.0% | 0.0% | 0.0% | 0.0% |
|  | k\_\_Bacteria;p\_\_Proteobacteria;c\_\_Deltaproteobacteria;o\_\_Myxococcales;f\_\_Myxococcaceae;g\_\_Anaeromyxobacter | 0 | 0.0% | 0.0% | 0.0% | 0.0% | 0.0% | 0.0% | 0.0% | 0.0% | 0.0% | 0.0% | 0.0% | 0.1% | 0.0% | 0.0% | 0.0% | 0.0% | 0.0% | 0.0% | 0.0% | 0.0% | 0.0% | 0.0% | 0.0% | 0.0% |
|  | k\_\_Bacteria;p\_\_Lentisphaerae;c\_\_[Lentisphaeria];o\_\_Z20;f\_\_R4-45B;g\_\_Unclassified\_R4-45B | 0 | 0.0% | 0.0% | 0.0% | 0.0% | 0.0% | 0.0% | 0.0% | 0.0% | 0.0% | 0.0% | 0.0% | 0.0% | 0.0% | 0.0% | 0.0% | 0.0% | 0.0% | 0.0% | 0.0% | 0.0% | 0.0% | 0.1% | 0.0% | 0.0% |
|  | k\_\_Bacteria;p\_\_Proteobacteria;c\_\_Deltaproteobacteria;o\_\_Myxococcales;f\_\_0319-6G20;g\_\_Unclassified\_0319-6G20 | 0 | 0.0% | 0.0% | 0.0% | 0.0% | 0.0% | 0.0% | 0.0% | 0.0% | 0.0% | 0.0% | 0.0% | 0.0% | 0.0% | 0.0% | 0.0% | 0.0% | 0.0% | 0.0% | 0.0% | 0.0% | 0.0% | 0.0% | 0.0% | 0.0% |
|  | k\_\_Bacteria;p\_\_Proteobacteria;c\_\_Betaproteobacteria;o\_\_Burkholderiales;f\_\_Comamonadaceae;g\_\_Curvibacter | 0 | 0.0% | 0.0% | 0.0% | 0.0% | 0.0% | 0.0% | 0.0% | 0.0% | 0.0% | 0.0% | 0.0% | 0.0% | 0.0% | 0.0% | 0.0% | 0.0% | 0.0% | 0.0% | 0.0% | 0.0% | 0.0% | 0.0% | 0.0% | 0.0% |
|  | k\_\_Bacteria;p\_\_Firmicutes;c\_\_Clostridia;o\_\_Clostridiales;f\_\_Eubacteriaceae;g\_\_Anaerofustis | 0 | 0.0% | 0.0% | 0.0% | 0.0% | 0.0% | 0.0% | 0.0% | 0.0% | 0.0% | 0.0% | 0.0% | 0.0% | 0.0% | 0.0% | 0.0% | 0.0% | 0.0% | 0.0% | 0.0% | 0.0% | 0.0% | 0.0% | 0.0% | 0.0% |
|  | k\_\_Bacteria;p\_\_Planctomycetes;c\_\_C6;o\_\_MVS-107;f\_\_Unclassified\_MVS-107;g\_\_Unclassified\_MVS-107 | 0 | 0.0% | 0.0% | 0.0% | 0.0% | 0.0% | 0.0% | 0.0% | 0.0% | 0.0% | 0.0% | 0.0% | 0.0% | 0.0% | 0.0% | 0.0% | 0.0% | 0.0% | 0.0% | 0.0% | 0.0% | 0.0% | 0.1% | 0.0% | 0.0% |
|  | k\_\_Bacteria;p\_\_Proteobacteria;c\_\_Alphaproteobacteria;o\_\_Caulobacterales;f\_\_Caulobacteraceae;g\_\_Mycoplana | 0 | 0.0% | 0.0% | 0.0% | 0.0% | 0.0% | 0.0% | 0.0% | 0.0% | 0.0% | 0.0% | 0.0% | 0.0% | 0.0% | 0.0% | 0.0% | 0.0% | 0.0% | 0.0% | 0.0% | 0.0% | 0.0% | 0.0% | 0.0% | 0.0% |
|  | k\_\_Bacteria;p\_\_Firmicutes;c\_\_Bacilli;o\_\_Lactobacillales;f\_\_Streptococcaceae;g\_\_Lactococcus | 0 | 0.0% | 0.0% | 0.0% | 0.0% | 0.0% | 0.0% | 0.0% | 0.0% | 0.0% | 0.0% | 0.0% | 0.0% | 0.0% | 0.0% | 0.0% | 0.0% | 0.0% | 0.0% | 0.0% | 0.0% | 0.0% | 0.0% | 0.0% | 0.0% |
|  | k\_\_Bacteria;p\_\_Actinobacteria;c\_\_Actinobacteria;o\_\_Actinomycetales;f\_\_Actinosynnemataceae;g\_\_Lentzea | 0 | 0.0% | 0.0% | 0.0% | 0.1% | 0.0% | 0.0% | 0.0% | 0.0% | 0.0% | 0.0% | 0.0% | 0.0% | 0.0% | 0.0% | 0.0% | 0.0% | 0.0% | 0.0% | 0.0% | 0.0% | 0.0% | 0.0% | 0.0% | 0.0% |
|  | k\_\_Bacteria;p\_\_Bacteroidetes;c\_\_Flavobacteriia;o\_\_Flavobacteriales;f\_\_Flavobacteriaceae;g\_\_Flavobacterium | 0 | 0.0% | 0.0% | 0.0% | 0.0% | 0.0% | 0.0% | 0.0% | 0.0% | 0.0% | 0.0% | 0.0% | 0.0% | 0.0% | 0.0% | 0.0% | 0.0% | 0.0% | 0.0% | 0.0% | 0.0% | 0.0% | 0.0% | 0.0% | 0.0% |
|  | k\_\_Bacteria;p\_\_Chlorobi;c\_\_SJA-28;o\_\_Unclassified\_SJA-28;f\_\_Unclassified\_SJA-28;g\_\_Unclassified\_SJA-28 | 0 | 0.0% | 0.0% | 0.0% | 0.0% | 0.0% | 0.0% | 0.0% | 0.0% | 0.0% | 0.0% | 0.0% | 0.0% | 0.0% | 0.0% | 0.0% | 0.0% | 0.0% | 0.0% | 0.0% | 0.0% | 0.0% | 0.0% | 0.0% | 0.0% |
|  | k\_\_Bacteria;p\_\_Proteobacteria;c\_\_Betaproteobacteria;o\_\_Burkholderiales;f\_\_Unclassified\_Burkholderiales;g\_\_Unclassified\_Burkholderiales | 0 | 0.0% | 0.0% | 0.0% | 0.0% | 0.0% | 0.0% | 0.0% | 0.0% | 0.0% | 0.0% | 0.0% | 0.0% | 0.0% | 0.0% | 0.0% | 0.0% | 0.0% | 0.0% | 0.0% | 0.0% | 0.0% | 0.0% | 0.0% | 0.0% |
|  | k\_\_Bacteria;p\_\_Chloroflexi;c\_\_TK17;o\_\_Unclassified\_TK17;f\_\_Unclassified\_TK17;g\_\_Unclassified\_TK17 | 0 | 0.0% | 0.0% | 0.0% | 0.0% | 0.0% | 0.0% | 0.0% | 0.0% | 0.0% | 0.0% | 0.0% | 0.0% | 0.0% | 0.0% | 0.0% | 0.0% | 0.0% | 0.0% | 0.0% | 0.0% | 0.0% | 0.0% | 0.0% | 0.0% |
|  | k\_\_Bacteria;p\_\_Firmicutes;c\_\_Bacilli;o\_\_Lactobacillales;f\_\_Unclassified\_Lactobacillales;g\_\_Unclassified\_Lactobacillales | 0 | 0.0% | 0.0% | 0.0% | 0.0% | 0.0% | 0.0% | 0.0% | 0.0% | 0.0% | 0.0% | 0.0% | 0.0% | 0.0% | 0.0% | 0.0% | 0.0% | 0.0% | 0.0% | 0.0% | 0.0% | 0.0% | 0.0% | 0.0% | 0.0% |
|  | k\_\_Bacteria;p\_\_Chloroflexi;c\_\_Anaerolineae;o\_\_DRC31;f\_\_Unclassified\_DRC31;g\_\_Unclassified\_DRC31 | 0 | 0.0% | 0.0% | 0.0% | 0.0% | 0.0% | 0.0% | 0.0% | 0.0% | 0.0% | 0.0% | 0.0% | 0.0% | 0.0% | 0.0% | 0.0% | 0.0% | 0.1% | 0.0% | 0.0% | 0.0% | 0.0% | 0.0% | 0.0% | 0.0% |
|  | k\_\_Bacteria;p\_\_Actinobacteria;c\_\_Actinobacteria;o\_\_Actinomycetales;f\_\_Intrasporangiaceae;g\_\_Intrasporangium | 0 | 0.0% | 0.0% | 0.0% | 0.0% | 0.0% | 0.0% | 0.0% | 0.0% | 0.0% | 0.0% | 0.0% | 0.0% | 0.0% | 0.0% | 0.0% | 0.0% | 0.0% | 0.0% | 0.0% | 0.0% | 0.0% | 0.0% | 0.0% | 0.0% |
|  | k\_\_Bacteria;p\_\_Bacteroidetes;c\_\_[Saprospirae];o\_\_[Saprospirales];f\_\_Chitinophagaceae;g\_\_Unclassified\_Chitinophagaceae | 0 | 0.0% | 0.0% | 0.0% | 0.0% | 0.0% | 0.0% | 0.0% | 0.0% | 0.0% | 0.0% | 0.0% | 0.0% | 0.0% | 0.0% | 0.0% | 0.0% | 0.0% | 0.0% | 0.0% | 0.0% | 0.0% | 0.0% | 0.0% | 0.0% |
|  | k\_\_Bacteria;p\_\_Actinobacteria;c\_\_Actinobacteria;o\_\_Actinomycetales;f\_\_Bogoriellaceae;g\_\_Georgenia | 0 | 0.0% | 0.0% | 0.0% | 0.0% | 0.0% | 0.0% | 0.0% | 0.0% | 0.0% | 0.0% | 0.0% | 0.0% | 0.0% | 0.0% | 0.0% | 0.0% | 0.0% | 0.0% | 0.0% | 0.0% | 0.0% | 0.0% | 0.0% | 0.0% |
|  | k\_\_Bacteria;p\_\_Firmicutes;c\_\_Erysipelotrichi;o\_\_Erysipelotrichales;f\_\_Erysipelotrichaceae;g\_\_L7A\_E11 | 0 | 0.0% | 0.0% | 0.0% | 0.0% | 0.0% | 0.0% | 0.0% | 0.0% | 0.0% | 0.0% | 0.0% | 0.0% | 0.0% | 0.0% | 0.0% | 0.0% | 0.1% | 0.0% | 0.0% | 0.0% | 0.0% | 0.0% | 0.0% | 0.0% |
|  | k\_\_Bacteria;p\_\_Proteobacteria;c\_\_Betaproteobacteria;o\_\_Burkholderiales;f\_\_Oxalobacteraceae;g\_\_Janthinobacterium | 0 | 0.0% | 0.0% | 0.0% | 0.0% | 0.0% | 0.0% | 0.0% | 0.0% | 0.0% | 0.0% | 0.0% | 0.0% | 0.0% | 0.0% | 0.0% | 0.0% | 0.0% | 0.0% | 0.0% | 0.0% | 0.0% | 0.0% | 0.0% | 0.0% |
|  | k\_\_Bacteria;p\_\_Firmicutes;c\_\_Bacilli;o\_\_Lactobacillales;f\_\_Lactobacillaceae;g\_\_Pediococcus | 0 | 0.0% | 0.0% | 0.0% | 0.0% | 0.0% | 0.0% | 0.0% | 0.0% | 0.0% | 0.0% | 0.0% | 0.0% | 0.0% | 0.0% | 0.0% | 0.0% | 0.0% | 0.0% | 0.0% | 0.0% | 0.0% | 0.0% | 0.0% | 0.0% |
|  | k\_\_Bacteria;p\_\_Bacteroidetes;c\_\_Cytophagia;o\_\_Cytophagales;f\_\_Cyclobacteriaceae;g\_\_Unclassified\_Cyclobacteriaceae | 0 | 0.0% | 0.0% | 0.0% | 0.0% | 0.0% | 0.0% | 0.0% | 0.0% | 0.0% | 0.0% | 0.0% | 0.0% | 0.0% | 0.0% | 0.0% | 0.0% | 0.0% | 0.0% | 0.0% | 0.0% | 0.0% | 0.0% | 0.0% | 0.0% |
|  | k\_\_Bacteria;p\_\_Bacteroidetes;c\_\_Bacteroidia;o\_\_Bacteroidales;f\_\_[Paraprevotellaceae];g\_\_Paraprevotella | 0 | 0.0% | 0.0% | 0.0% | 0.0% | 0.0% | 0.0% | 0.0% | 0.0% | 0.0% | 0.0% | 0.0% | 0.0% | 0.0% | 0.0% | 0.0% | 0.0% | 0.0% | 0.0% | 0.0% | 0.0% | 0.0% | 0.0% | 0.0% | 0.0% |
|  | k\_\_Bacteria;p\_\_Firmicutes;c\_\_Clostridia;o\_\_Thermoanaerobacterales;f\_\_Thermoanaerobacteraceae;g\_\_Thermoanaerobacter | 0 | 0.0% | 0.0% | 0.0% | 0.0% | 0.0% | 0.0% | 0.0% | 0.0% | 0.0% | 0.0% | 0.0% | 0.0% | 0.0% | 0.0% | 0.0% | 0.0% | 0.0% | 0.0% | 0.0% | 0.0% | 0.0% | 0.0% | 0.0% | 0.0% |
|  | k\_\_Bacteria;p\_\_Chloroflexi;c\_\_Ktedonobacteria;o\_\_JG30-KF-AS9;f\_\_Unclassified\_JG30-KF-AS9;g\_\_Unclassified\_JG30-KF-AS9 | 0 | 0.0% | 0.0% | 0.0% | 0.0% | 0.0% | 0.0% | 0.0% | 0.0% | 0.0% | 0.0% | 0.0% | 0.0% | 0.0% | 0.0% | 0.1% | 0.0% | 0.0% | 0.0% | 0.0% | 0.0% | 0.0% | 0.0% | 0.0% | 0.0% |
|  | k\_\_Bacteria;p\_\_Proteobacteria;c\_\_Betaproteobacteria;o\_\_Burkholderiales;f\_\_Comamonadaceae;g\_\_Rubrivivax | 0 | 0.0% | 0.0% | 0.0% | 0.0% | 0.0% | 0.0% | 0.0% | 0.0% | 0.0% | 0.0% | 0.0% | 0.0% | 0.0% | 0.0% | 0.0% | 0.0% | 0.0% | 0.0% | 0.0% | 0.0% | 0.0% | 0.0% | 0.0% | 0.0% |
|  | k\_\_Bacteria;p\_\_Gemmatimonadetes;c\_\_Gemm-5;o\_\_Unclassified\_Gemm-5;f\_\_Unclassified\_Gemm-5;g\_\_Unclassified\_Gemm-5 | 0 | 0.0% | 0.0% | 0.0% | 0.0% | 0.0% | 0.0% | 0.0% | 0.0% | 0.0% | 0.0% | 0.0% | 0.0% | 0.0% | 0.0% | 0.0% | 0.0% | 0.0% | 0.0% | 0.0% | 0.0% | 0.0% | 0.0% | 0.0% | 0.0% |
|  | k\_\_Bacteria;p\_\_Firmicutes;c\_\_Clostridia;o\_\_Clostridiales;f\_\_Lachnospiraceae;g\_\_Oribacterium | 0 | 0.0% | 0.0% | 0.0% | 0.0% | 0.0% | 0.0% | 0.0% | 0.0% | 0.0% | 0.0% | 0.0% | 0.0% | 0.0% | 0.0% | 0.0% | 0.0% | 0.0% | 0.0% | 0.0% | 0.0% | 0.1% | 0.0% | 0.0% | 0.0% |
|  | k\_\_Bacteria;p\_\_Actinobacteria;c\_\_Actinobacteria;o\_\_Actinomycetales;f\_\_Cellulomonadaceae;g\_\_Actinotalea | 0 | 0.0% | 0.0% | 0.0% | 0.0% | 0.0% | 0.0% | 0.0% | 0.0% | 0.0% | 0.0% | 0.0% | 0.0% | 0.0% | 0.0% | 0.0% | 0.0% | 0.0% | 0.0% | 0.0% | 0.0% | 0.0% | 0.1% | 0.0% | 0.0% |
|  | k\_\_Bacteria;p\_\_Acidobacteria;c\_\_Acidobacteria-6;o\_\_iii1-15;f\_\_mb2424;g\_\_Unclassified\_mb2424 | 0 | 0.0% | 0.0% | 0.0% | 0.0% | 0.0% | 0.0% | 0.0% | 0.0% | 0.0% | 0.0% | 0.0% | 0.0% | 0.0% | 0.0% | 0.0% | 0.0% | 0.0% | 0.0% | 0.0% | 0.0% | 0.0% | 0.0% | 0.0% | 0.0% |
|  | k\_\_Bacteria;p\_\_Bacteroidetes;c\_\_Bacteroidia;o\_\_Bacteroidales;f\_\_Bacteroidaceae;g\_\_BF311 | 0 | 0.0% | 0.0% | 0.0% | 0.0% | 0.0% | 0.0% | 0.1% | 0.0% | 0.0% | 0.0% | 0.0% | 0.0% | 0.0% | 0.0% | 0.0% | 0.0% | 0.0% | 0.0% | 0.0% | 0.0% | 0.0% | 0.0% | 0.0% | 0.0% |
|  | k\_\_Bacteria;p\_\_Bacteroidetes;c\_\_Sphingobacteriia;o\_\_Sphingobacteriales;f\_\_Sphingobacteriaceae;g\_\_Sphingobacterium | 0 | 0.0% | 0.0% | 0.0% | 0.0% | 0.0% | 0.0% | 0.0% | 0.0% | 0.0% | 0.0% | 0.0% | 0.0% | 0.0% | 0.0% | 0.0% | 0.0% | 0.0% | 0.0% | 0.0% | 0.0% | 0.0% | 0.0% | 0.0% | 0.0% |
|  | k\_\_Bacteria;p\_\_Firmicutes;c\_\_Bacilli;o\_\_Bacillales;f\_\_Planococcaceae;g\_\_Sporosarcina | 0 | 0.0% | 0.0% | 0.0% | 0.0% | 0.0% | 0.0% | 0.0% | 0.0% | 0.0% | 0.0% | 0.0% | 0.0% | 0.0% | 0.0% | 0.0% | 0.0% | 0.0% | 0.0% | 0.0% | 0.0% | 0.0% | 0.0% | 0.0% | 0.0% |
|  | k\_\_Bacteria;p\_\_Proteobacteria;c\_\_Gammaproteobacteria;o\_\_Alteromonadales;f\_\_[Chromatiaceae];g\_\_Rheinheimera | 0 | 0.0% | 0.0% | 0.0% | 0.0% | 0.0% | 0.0% | 0.0% | 0.0% | 0.0% | 0.0% | 0.0% | 0.0% | 0.0% | 0.0% | 0.0% | 0.0% | 0.0% | 0.0% | 0.0% | 0.0% | 0.0% | 0.0% | 0.0% | 0.0% |
|  | k\_\_Bacteria;p\_\_Proteobacteria;c\_\_Gammaproteobacteria;o\_\_Vibrionales;f\_\_Pseudoalteromonadaceae;g\_\_Unclassified\_Pseudoalteromonadaceae | 0 | 0.0% | 0.0% | 0.0% | 0.1% | 0.0% | 0.0% | 0.0% | 0.0% | 0.0% | 0.0% | 0.0% | 0.0% | 0.0% | 0.0% | 0.0% | 0.0% | 0.0% | 0.0% | 0.0% | 0.0% | 0.0% | 0.0% | 0.0% | 0.0% |
|  | k\_\_Bacteria;p\_\_Actinobacteria;c\_\_Actinobacteria;o\_\_Actinomycetales;f\_\_Yaniellaceae;g\_\_Yaniella | 0 | 0.0% | 0.0% | 0.0% | 0.0% | 0.0% | 0.0% | 0.0% | 0.0% | 0.0% | 0.0% | 0.0% | 0.0% | 0.0% | 0.0% | 0.0% | 0.0% | 0.0% | 0.0% | 0.0% | 0.0% | 0.0% | 0.0% | 0.0% | 0.0% |
|  | k\_\_Bacteria;p\_\_Proteobacteria;c\_\_Deltaproteobacteria;o\_\_Desulfuromonadales;f\_\_Geobacteraceae;g\_\_Geobacter | 0 | 0.0% | 0.0% | 0.0% | 0.0% | 0.0% | 0.0% | 0.0% | 0.0% | 0.0% | 0.0% | 0.0% | 0.0% | 0.0% | 0.0% | 0.0% | 0.0% | 0.0% | 0.0% | 0.0% | 0.0% | 0.0% | 0.0% | 0.0% | 0.0% |
|  | k\_\_Bacteria;p\_\_Firmicutes;c\_\_Bacilli;o\_\_Bacillales;f\_\_Planococcaceae;g\_\_Kurthia | 0 | 0.0% | 0.0% | 0.0% | 0.0% | 0.0% | 0.0% | 0.0% | 0.0% | 0.0% | 0.0% | 0.0% | 0.0% | 0.0% | 0.0% | 0.0% | 0.0% | 0.1% | 0.0% | 0.0% | 0.0% | 0.0% | 0.0% | 0.0% | 0.0% |
|  | k\_\_Bacteria;p\_\_Firmicutes;c\_\_Clostridia;o\_\_Clostridiales;f\_\_Veillonellaceae;g\_\_Sporomusa | 0 | 0.0% | 0.0% | 0.0% | 0.0% | 0.0% | 0.0% | 0.0% | 0.0% | 0.0% | 0.0% | 0.0% | 0.0% | 0.0% | 0.0% | 0.0% | 0.0% | 0.1% | 0.0% | 0.0% | 0.0% | 0.0% | 0.0% | 0.0% | 0.0% |
|  | k\_\_Bacteria;p\_\_Proteobacteria;c\_\_Gammaproteobacteria;o\_\_Enterobacteriales;f\_\_Enterobacteriaceae;g\_\_Edwardsiella | 0 | 0.0% | 0.0% | 0.0% | 0.0% | 0.0% | 0.0% | 0.0% | 0.0% | 0.0% | 0.0% | 0.0% | 0.0% | 0.0% | 0.0% | 0.0% | 0.0% | 0.1% | 0.0% | 0.0% | 0.0% | 0.0% | 0.0% | 0.0% | 0.0% |
|  | k\_\_Bacteria;p\_\_WS3;c\_\_PRR-12;o\_\_Sediment-1;f\_\_PRR-10;g\_\_Unclassified\_PRR-10 | 0 | 0.0% | 0.0% | 0.0% | 0.0% | 0.0% | 0.0% | 0.0% | 0.0% | 0.0% | 0.0% | 0.0% | 0.0% | 0.0% | 0.0% | 0.0% | 0.0% | 0.1% | 0.0% | 0.0% | 0.0% | 0.0% | 0.0% | 0.0% | 0.0% |
|  | k\_\_Bacteria;p\_\_Proteobacteria;c\_\_Epsilonproteobacteria;o\_\_Campylobacterales;f\_\_Helicobacteraceae;g\_\_Helicobacter | 0 | 0.0% | 0.0% | 0.0% | 0.0% | 0.0% | 0.0% | 0.0% | 0.0% | 0.0% | 0.0% | 0.0% | 0.0% | 0.0% | 0.0% | 0.0% | 0.0% | 0.0% | 0.0% | 0.0% | 0.0% | 0.0% | 0.0% | 0.0% | 0.0% |
|  | k\_\_Bacteria;p\_\_Proteobacteria;c\_\_Alphaproteobacteria;o\_\_Rhizobiales;f\_\_Hyphomicrobiaceae;g\_\_Unclassified\_Hyphomicrobiaceae | 0 | 0.0% | 0.0% | 0.0% | 0.0% | 0.0% | 0.0% | 0.0% | 0.0% | 0.0% | 0.0% | 0.0% | 0.0% | 0.0% | 0.0% | 0.0% | 0.0% | 0.0% | 0.0% | 0.0% | 0.0% | 0.0% | 0.0% | 0.0% | 0.0% |
|  | k\_\_Bacteria;p\_\_Firmicutes;c\_\_Bacilli;o\_\_Bacillales;f\_\_[Exiguobacteraceae];g\_\_Unclassified\_[Exiguobacteraceae] | 0 | 0.0% | 0.0% | 0.0% | 0.0% | 0.0% | 0.0% | 0.0% | 0.0% | 0.0% | 0.0% | 0.0% | 0.0% | 0.0% | 0.0% | 0.0% | 0.0% | 0.0% | 0.0% | 0.0% | 0.0% | 0.0% | 0.0% | 0.0% | 0.0% |
|  | k\_\_Bacteria;p\_\_Proteobacteria;c\_\_Alphaproteobacteria;o\_\_Rhizobiales;f\_\_Xanthobacteraceae;g\_\_Labrys | 0 | 0.0% | 0.0% | 0.0% | 0.0% | 0.0% | 0.0% | 0.0% | 0.0% | 0.0% | 0.0% | 0.0% | 0.0% | 0.0% | 0.0% | 0.0% | 0.0% | 0.0% | 0.0% | 0.0% | 0.0% | 0.0% | 0.0% | 0.0% | 0.0% |
|  | k\_\_Bacteria;p\_\_Actinobacteria;c\_\_Acidimicrobiia;o\_\_Acidimicrobiales;f\_\_C111;g\_\_Unclassified\_C111 | 0 | 0.0% | 0.0% | 0.0% | 0.0% | 0.0% | 0.0% | 0.0% | 0.0% | 0.0% | 0.0% | 0.0% | 0.0% | 0.0% | 0.0% | 0.0% | 0.0% | 0.0% | 0.0% | 0.0% | 0.0% | 0.0% | 0.0% | 0.0% | 0.0% |
|  | k\_\_Bacteria;p\_\_Proteobacteria;c\_\_Alphaproteobacteria;o\_\_Rhodospirillales;f\_\_Unclassified\_Rhodospirillales;g\_\_Unclassified\_Rhodospirillales | 0 | 0.0% | 0.0% | 0.0% | 0.0% | 0.0% | 0.0% | 0.0% | 0.0% | 0.0% | 0.0% | 0.0% | 0.0% | 0.0% | 0.0% | 0.0% | 0.0% | 0.0% | 0.0% | 0.0% | 0.0% | 0.0% | 0.0% | 0.0% | 0.0% |
|  | k\_\_Bacteria;p\_\_Bacteroidetes;c\_\_Flavobacteriia;o\_\_Flavobacteriales;f\_\_Cryomorphaceae;g\_\_Unclassified\_Cryomorphaceae | 0 | 0.0% | 0.0% | 0.0% | 0.0% | 0.0% | 0.0% | 0.0% | 0.0% | 0.0% | 0.0% | 0.0% | 0.0% | 0.0% | 0.0% | 0.0% | 0.0% | 0.0% | 0.0% | 0.0% | 0.0% | 0.0% | 0.0% | 0.0% | 0.1% |
|  | k\_\_Bacteria;p\_\_Firmicutes;c\_\_Clostridia;o\_\_Clostridiales;f\_\_Veillonellaceae;g\_\_Unclassified\_Veillonellaceae | 0 | 0.0% | 0.0% | 0.0% | 0.0% | 0.0% | 0.0% | 0.0% | 0.0% | 0.0% | 0.0% | 0.0% | 0.0% | 0.0% | 0.0% | 0.0% | 0.0% | 0.0% | 0.0% | 0.0% | 0.0% | 0.0% | 0.0% | 0.0% | 0.0% |
|  | k\_\_Bacteria;p\_\_Planctomycetes;c\_\_Phycisphaerae;o\_\_Phycisphaerales;f\_\_Phycisphaeraceae;g\_\_Unclassified\_Phycisphaeraceae | 0 | 0.0% | 0.0% | 0.0% | 0.0% | 0.0% | 0.0% | 0.0% | 0.0% | 0.0% | 0.0% | 0.0% | 0.0% | 0.0% | 0.0% | 0.0% | 0.0% | 0.0% | 0.0% | 0.0% | 0.0% | 0.0% | 0.0% | 0.0% | 0.0% |
|  | k\_\_Bacteria;p\_\_Firmicutes;c\_\_Clostridia;o\_\_Clostridiales;f\_\_Lachnospiraceae;g\_\_Anaerostipes | 0 | 0.0% | 0.0% | 0.0% | 0.0% | 0.0% | 0.0% | 0.0% | 0.0% | 0.0% | 0.0% | 0.0% | 0.0% | 0.0% | 0.0% | 0.0% | 0.0% | 0.0% | 0.0% | 0.0% | 0.0% | 0.0% | 0.0% | 0.0% | 0.0% |
|  | k\_\_Bacteria;p\_\_Firmicutes;c\_\_Clostridia;o\_\_Clostridiales;f\_\_Dehalobacteriaceae;g\_\_Unclassified\_Dehalobacteriaceae | 0 | 0.0% | 0.0% | 0.0% | 0.0% | 0.0% | 0.0% | 0.0% | 0.0% | 0.0% | 0.0% | 0.0% | 0.0% | 0.0% | 0.0% | 0.0% | 0.0% | 0.0% | 0.0% | 0.0% | 0.0% | 0.0% | 0.0% | 0.0% | 0.0% |
|  | k\_\_Bacteria;p\_\_Proteobacteria;c\_\_Alphaproteobacteria;o\_\_RF32;f\_\_Unclassified\_RF32;g\_\_Unclassified\_RF32 | 0 | 0.0% | 0.0% | 0.0% | 0.0% | 0.0% | 0.0% | 0.0% | 0.0% | 0.0% | 0.0% | 0.0% | 0.0% | 0.0% | 0.0% | 0.0% | 0.0% | 0.0% | 0.0% | 0.0% | 0.0% | 0.0% | 0.0% | 0.0% | 0.0% |
|  | k\_\_Bacteria;p\_\_Proteobacteria;c\_\_Alphaproteobacteria;o\_\_Rhodobacterales;f\_\_Rhodobacteraceae;g\_\_Rubellimicrobium | 0 | 0.0% | 0.0% | 0.0% | 0.0% | 0.0% | 0.0% | 0.0% | 0.0% | 0.0% | 0.0% | 0.0% | 0.0% | 0.0% | 0.0% | 0.0% | 0.0% | 0.0% | 0.0% | 0.0% | 0.0% | 0.0% | 0.0% | 0.0% | 0.0% |
|  | k\_\_Bacteria;p\_\_Actinobacteria;c\_\_Actinobacteria;o\_\_Actinomycetales;f\_\_Pseudonocardiaceae;g\_\_Pseudonocardia | 0 | 0.0% | 0.0% | 0.0% | 0.0% | 0.0% | 0.0% | 0.0% | 0.0% | 0.0% | 0.0% | 0.0% | 0.0% | 0.0% | 0.0% | 0.0% | 0.0% | 0.0% | 0.0% | 0.0% | 0.0% | 0.0% | 0.0% | 0.0% | 0.0% |
|  | k\_\_Bacteria;p\_\_Proteobacteria;c\_\_Deltaproteobacteria;o\_\_Desulfobacterales;f\_\_Desulfobacteraceae;g\_\_Desulfococcus | 0 | 0.0% | 0.0% | 0.0% | 0.0% | 0.0% | 0.0% | 0.0% | 0.0% | 0.0% | 0.0% | 0.0% | 0.0% | 0.0% | 0.0% | 0.0% | 0.0% | 0.1% | 0.0% | 0.0% | 0.0% | 0.0% | 0.0% | 0.0% | 0.0% |
|  | k\_\_Bacteria;p\_\_Verrucomicrobia;c\_\_Opitutae;o\_\_[Cerasicoccales];f\_\_[Cerasicoccaceae];g\_\_Unclassified\_[Cerasicoccaceae] | 0 | 0.0% | 0.0% | 0.0% | 0.0% | 0.0% | 0.0% | 0.0% | 0.0% | 0.0% | 0.0% | 0.0% | 0.0% | 0.0% | 0.0% | 0.0% | 0.0% | 0.0% | 0.0% | 0.0% | 0.0% | 0.0% | 0.0% | 0.0% | 0.0% |
|  | k\_\_Bacteria;p\_\_Firmicutes;c\_\_Bacilli;o\_\_Bacillales;f\_\_Thermoactinomycetaceae;g\_\_Thermoactinomyces | 0 | 0.0% | 0.0% | 0.0% | 0.0% | 0.0% | 0.0% | 0.0% | 0.0% | 0.0% | 0.0% | 0.0% | 0.0% | 0.0% | 0.0% | 0.0% | 0.0% | 0.0% | 0.0% | 0.0% | 0.0% | 0.0% | 0.0% | 0.0% | 0.0% |
|  | k\_\_Bacteria;p\_\_Chloroflexi;c\_\_Anaerolineae;o\_\_Caldilineales;f\_\_Caldilineaceae;g\_\_Caldilinea | 0 | 0.0% | 0.0% | 0.0% | 0.0% | 0.0% | 0.0% | 0.0% | 0.0% | 0.0% | 0.0% | 0.0% | 0.0% | 0.0% | 0.0% | 0.0% | 0.1% | 0.0% | 0.0% | 0.0% | 0.0% | 0.0% | 0.0% | 0.0% | 0.0% |
|  | k\_\_Bacteria;p\_\_Spirochaetes;c\_\_Spirochaetes;o\_\_Spirochaetales;f\_\_Spirochaetaceae;g\_\_Unclassified\_Spirochaetaceae | 0 | 0.0% | 0.0% | 0.0% | 0.0% | 0.0% | 0.0% | 0.0% | 0.0% | 0.0% | 0.0% | 0.0% | 0.0% | 0.0% | 0.0% | 0.0% | 0.0% | 0.0% | 0.0% | 0.0% | 0.0% | 0.0% | 0.0% | 0.0% | 0.0% |
|  | k\_\_Bacteria;p\_\_Proteobacteria;c\_\_Betaproteobacteria;o\_\_Burkholderiales;f\_\_Oxalobacteraceae;g\_\_Polynucleobacter | 0 | 0.0% | 0.0% | 0.0% | 0.0% | 0.0% | 0.0% | 0.0% | 0.0% | 0.0% | 0.0% | 0.0% | 0.0% | 0.0% | 0.0% | 0.0% | 0.0% | 0.0% | 0.0% | 0.0% | 0.0% | 0.0% | 0.0% | 0.0% | 0.0% |
|  | k\_\_Bacteria;p\_\_Proteobacteria;c\_\_Gammaproteobacteria;o\_\_Xanthomonadales;f\_\_Xanthomonadaceae;g\_\_Thermomonas | 0 | 0.0% | 0.0% | 0.0% | 0.0% | 0.0% | 0.0% | 0.0% | 0.0% | 0.0% | 0.0% | 0.0% | 0.0% | 0.0% | 0.0% | 0.0% | 0.0% | 0.0% | 0.0% | 0.0% | 0.0% | 0.0% | 0.0% | 0.0% | 0.0% |
|  | k\_\_Bacteria;p\_\_Proteobacteria;c\_\_Gammaproteobacteria;o\_\_Unclassified\_Gammaproteobacteria;f\_\_Unclassified\_Gammaproteobacteria;g\_\_Unclassified\_Gammaproteobacteria | 0 | 0.0% | 0.0% | 0.0% | 0.0% | 0.0% | 0.0% | 0.0% | 0.0% | 0.0% | 0.0% | 0.0% | 0.0% | 0.0% | 0.0% | 0.0% | 0.0% | 0.0% | 0.0% | 0.0% | 0.0% | 0.0% | 0.0% | 0.0% | 0.0% |
|  | k\_\_Bacteria;p\_\_Proteobacteria;c\_\_Deltaproteobacteria;o\_\_Syntrophobacterales;f\_\_Syntrophobacteraceae;g\_\_Syntrophobacter | 0 | 0.0% | 0.0% | 0.0% | 0.0% | 0.0% | 0.0% | 0.0% | 0.0% | 0.0% | 0.0% | 0.0% | 0.0% | 0.0% | 0.0% | 0.0% | 0.1% | 0.0% | 0.0% | 0.0% | 0.0% | 0.0% | 0.0% | 0.0% | 0.0% |
|  | k\_\_Bacteria;p\_\_Proteobacteria;c\_\_Deltaproteobacteria;o\_\_Desulfobacterales;f\_\_Desulfobulbaceae;g\_\_Unclassified\_Desulfobulbaceae | 0 | 0.0% | 0.0% | 0.0% | 0.0% | 0.0% | 0.0% | 0.0% | 0.0% | 0.0% | 0.0% | 0.0% | 0.0% | 0.0% | 0.0% | 0.0% | 0.0% | 0.0% | 0.0% | 0.0% | 0.0% | 0.0% | 0.0% | 0.0% | 0.0% |
|  | k\_\_Bacteria;p\_\_Proteobacteria;c\_\_Deltaproteobacteria;o\_\_Myxococcales;f\_\_OM27;g\_\_Unclassified\_OM27 | 0 | 0.0% | 0.0% | 0.0% | 0.0% | 0.0% | 0.0% | 0.0% | 0.0% | 0.0% | 0.0% | 0.0% | 0.0% | 0.0% | 0.0% | 0.0% | 0.0% | 0.0% | 0.0% | 0.0% | 0.0% | 0.0% | 0.0% | 0.0% | 0.0% |
|  | k\_\_Bacteria;p\_\_Proteobacteria;c\_\_Betaproteobacteria;o\_\_Methylophilales;f\_\_Methylophilaceae;g\_\_Unclassified\_Methylophilaceae | 0 | 0.0% | 0.0% | 0.0% | 0.0% | 0.0% | 0.0% | 0.0% | 0.0% | 0.0% | 0.0% | 0.0% | 0.0% | 0.0% | 0.0% | 0.0% | 0.0% | 0.0% | 0.0% | 0.0% | 0.0% | 0.0% | 0.0% | 0.0% | 0.0% |
|  | k\_\_Bacteria;p\_\_Firmicutes;c\_\_Bacilli;o\_\_Lactobacillales;f\_\_Leuconostocaceae;g\_\_Unclassified\_Leuconostocaceae | 0 | 0.0% | 0.0% | 0.0% | 0.0% | 0.0% | 0.0% | 0.0% | 0.0% | 0.0% | 0.0% | 0.0% | 0.0% | 0.0% | 0.0% | 0.0% | 0.0% | 0.0% | 0.0% | 0.0% | 0.0% | 0.0% | 0.0% | 0.0% | 0.0% |
|  | k\_\_Bacteria;p\_\_Firmicutes;c\_\_Clostridia;o\_\_Clostridiales;f\_\_Veillonellaceae;g\_\_Megamonas | 0 | 0.0% | 0.0% | 0.0% | 0.0% | 0.0% | 0.0% | 0.0% | 0.0% | 0.0% | 0.0% | 0.0% | 0.0% | 0.0% | 0.0% | 0.0% | 0.0% | 0.0% | 0.0% | 0.0% | 0.0% | 0.0% | 0.0% | 0.0% | 0.0% |
|  | k\_\_Bacteria;p\_\_Firmicutes;c\_\_Bacilli;o\_\_Lactobacillales;f\_\_Enterococcaceae;g\_\_Enterococcus | 0 | 0.0% | 0.0% | 0.0% | 0.0% | 0.0% | 0.0% | 0.0% | 0.0% | 0.0% | 0.0% | 0.0% | 0.0% | 0.0% | 0.0% | 0.0% | 0.0% | 0.0% | 0.0% | 0.0% | 0.0% | 0.0% | 0.0% | 0.0% | 0.0% |
|  | k\_\_Bacteria;p\_\_TM7;c\_\_TM7-1;o\_\_Unclassified\_TM7-1;f\_\_Unclassified\_TM7-1;g\_\_Unclassified\_TM7-1 | 0 | 0.0% | 0.0% | 0.0% | 0.0% | 0.0% | 0.0% | 0.0% | 0.0% | 0.0% | 0.0% | 0.0% | 0.0% | 0.0% | 0.0% | 0.0% | 0.0% | 0.0% | 0.0% | 0.0% | 0.0% | 0.0% | 0.0% | 0.0% | 0.0% |
|  | k\_\_Bacteria;p\_\_Proteobacteria;c\_\_Gammaproteobacteria;o\_\_Oceanospirillales;f\_\_Halomonadaceae;g\_\_Halomonas | 0 | 0.0% | 0.0% | 0.0% | 0.0% | 0.0% | 0.0% | 0.0% | 0.0% | 0.0% | 0.0% | 0.0% | 0.0% | 0.0% | 0.0% | 0.0% | 0.0% | 0.0% | 0.0% | 0.0% | 0.0% | 0.0% | 0.0% | 0.0% | 0.0% |
|  | k\_\_Bacteria;p\_\_Actinobacteria;c\_\_Actinobacteria;o\_\_Actinomycetales;f\_\_Micromonosporaceae;g\_\_Phytohabitans | 0 | 0.0% | 0.0% | 0.0% | 0.0% | 0.0% | 0.0% | 0.0% | 0.0% | 0.0% | 0.0% | 0.0% | 0.0% | 0.0% | 0.0% | 0.0% | 0.0% | 0.0% | 0.0% | 0.0% | 0.0% | 0.0% | 0.0% | 0.0% | 0.0% |
|  | k\_\_Bacteria;p\_\_Actinobacteria;c\_\_Actinobacteria;o\_\_Actinomycetales;f\_\_Actinosynnemataceae;g\_\_Saccharothrix | 0 | 0.0% | 0.0% | 0.0% | 0.0% | 0.0% | 0.0% | 0.0% | 0.0% | 0.0% | 0.0% | 0.0% | 0.0% | 0.0% | 0.0% | 0.0% | 0.0% | 0.0% | 0.0% | 0.0% | 0.0% | 0.0% | 0.0% | 0.0% | 0.0% |
|  | k\_\_Bacteria;p\_\_Proteobacteria;c\_\_Gammaproteobacteria;o\_\_[Marinicellales];f\_\_[Marinicellaceae];g\_\_Unclassified\_[Marinicellaceae] | 0 | 0.0% | 0.0% | 0.0% | 0.0% | 0.0% | 0.0% | 0.0% | 0.0% | 0.0% | 0.0% | 0.0% | 0.0% | 0.0% | 0.0% | 0.0% | 0.0% | 0.0% | 0.0% | 0.0% | 0.0% | 0.0% | 0.0% | 0.0% | 0.0% |
|  | k\_\_Bacteria;p\_\_Proteobacteria;c\_\_Epsilonproteobacteria;o\_\_Campylobacterales;f\_\_Helicobacteraceae;g\_\_Unclassified\_Helicobacteraceae | 0 | 0.0% | 0.0% | 0.0% | 0.0% | 0.0% | 0.0% | 0.0% | 0.0% | 0.0% | 0.0% | 0.0% | 0.0% | 0.0% | 0.0% | 0.0% | 0.0% | 0.0% | 0.0% | 0.0% | 0.0% | 0.0% | 0.0% | 0.0% | 0.0% |
|  | k\_\_Bacteria;p\_\_Actinobacteria;c\_\_Actinobacteria;o\_\_Actinomycetales;f\_\_Sporichthyaceae;g\_\_Unclassified\_Sporichthyaceae | 0 | 0.0% | 0.0% | 0.0% | 0.0% | 0.0% | 0.0% | 0.0% | 0.0% | 0.0% | 0.0% | 0.0% | 0.0% | 0.0% | 0.0% | 0.0% | 0.0% | 0.0% | 0.0% | 0.0% | 0.0% | 0.0% | 0.0% | 0.0% | 0.0% |
|  | k\_\_Bacteria;p\_\_Cyanobacteria;c\_\_ML635J-21;o\_\_Unclassified\_ML635J-21;f\_\_Unclassified\_ML635J-21;g\_\_Unclassified\_ML635J-21 | 0 | 0.0% | 0.0% | 0.0% | 0.0% | 0.0% | 0.0% | 0.0% | 0.0% | 0.0% | 0.0% | 0.0% | 0.0% | 0.0% | 0.0% | 0.0% | 0.0% | 0.0% | 0.0% | 0.0% | 0.0% | 0.0% | 0.0% | 0.0% | 0.0% |
|  | k\_\_Bacteria;p\_\_Acidobacteria;c\_\_Solibacteres;o\_\_Solibacterales;f\_\_PAUC26f;g\_\_Unclassified\_PAUC26f | 0 | 0.0% | 0.0% | 0.0% | 0.0% | 0.0% | 0.0% | 0.0% | 0.0% | 0.0% | 0.0% | 0.0% | 0.0% | 0.0% | 0.0% | 0.0% | 0.0% | 0.0% | 0.0% | 0.0% | 0.0% | 0.0% | 0.0% | 0.0% | 0.0% |
|  | k\_\_Bacteria;p\_\_Actinobacteria;c\_\_Actinobacteria;o\_\_Actinomycetales;f\_\_Nakamurellaceae;g\_\_Unclassified\_Nakamurellaceae | 0 | 0.0% | 0.0% | 0.0% | 0.0% | 0.0% | 0.0% | 0.0% | 0.0% | 0.0% | 0.0% | 0.0% | 0.0% | 0.0% | 0.0% | 0.0% | 0.0% | 0.0% | 0.0% | 0.0% | 0.0% | 0.0% | 0.0% | 0.0% | 0.0% |
|  | k\_\_Bacteria;p\_\_Proteobacteria;c\_\_Gammaproteobacteria;o\_\_Xanthomonadales;f\_\_Xanthomonadaceae;g\_\_Dyella | 0 | 0.0% | 0.0% | 0.0% | 0.0% | 0.0% | 0.0% | 0.0% | 0.0% | 0.0% | 0.0% | 0.0% | 0.0% | 0.0% | 0.0% | 0.0% | 0.0% | 0.0% | 0.0% | 0.0% | 0.0% | 0.0% | 0.0% | 0.0% | 0.0% |
|  | k\_\_Bacteria;p\_\_Planctomycetes;c\_\_OM190;o\_\_agg27;f\_\_Unclassified\_agg27;g\_\_Unclassified\_agg27 | 0 | 0.0% | 0.0% | 0.0% | 0.0% | 0.0% | 0.0% | 0.0% | 0.0% | 0.0% | 0.0% | 0.0% | 0.0% | 0.0% | 0.0% | 0.0% | 0.0% | 0.0% | 0.0% | 0.0% | 0.0% | 0.0% | 0.0% | 0.0% | 0.0% |
|  | k\_\_Bacteria;p\_\_Chloroflexi;c\_\_Anaerolineae;o\_\_S0208;f\_\_Unclassified\_S0208;g\_\_Unclassified\_S0208 | 0 | 0.0% | 0.0% | 0.0% | 0.0% | 0.0% | 0.0% | 0.0% | 0.0% | 0.0% | 0.0% | 0.0% | 0.0% | 0.0% | 0.0% | 0.0% | 0.0% | 0.0% | 0.0% | 0.0% | 0.0% | 0.0% | 0.0% | 0.0% | 0.0% |
|  | k\_\_Bacteria;p\_\_Proteobacteria;c\_\_Alphaproteobacteria;o\_\_Rhodospirillales;f\_\_Rhodospirillaceae;g\_\_Skermanella | 0 | 0.0% | 0.0% | 0.0% | 0.0% | 0.0% | 0.0% | 0.0% | 0.0% | 0.0% | 0.0% | 0.0% | 0.0% | 0.0% | 0.0% | 0.0% | 0.0% | 0.0% | 0.0% | 0.0% | 0.0% | 0.0% | 0.0% | 0.0% | 0.0% |
|  | k\_\_Bacteria;p\_\_Elusimicrobia;c\_\_Elusimicrobia;o\_\_Elusimicrobiales;f\_\_Unclassified\_Elusimicrobiales;g\_\_Unclassified\_Elusimicrobiales | 0 | 0.0% | 0.0% | 0.0% | 0.0% | 0.0% | 0.0% | 0.0% | 0.0% | 0.0% | 0.0% | 0.0% | 0.0% | 0.0% | 0.0% | 0.0% | 0.0% | 0.0% | 0.0% | 0.0% | 0.0% | 0.0% | 0.0% | 0.0% | 0.0% |
|  | k\_\_Bacteria;p\_\_Proteobacteria;c\_\_Gammaproteobacteria;o\_\_Thiotrichales;f\_\_Thiotrichaceae;g\_\_Thiothrix | 0 | 0.0% | 0.0% | 0.0% | 0.0% | 0.0% | 0.0% | 0.0% | 0.0% | 0.0% | 0.0% | 0.0% | 0.0% | 0.0% | 0.0% | 0.0% | 0.0% | 0.0% | 0.0% | 0.0% | 0.0% | 0.0% | 0.0% | 0.0% | 0.0% |
|  | k\_\_Bacteria;p\_\_Actinobacteria;c\_\_Coriobacteriia;o\_\_Coriobacteriales;f\_\_Coriobacteriaceae;g\_\_Eggerthella | 0 | 0.0% | 0.0% | 0.0% | 0.0% | 0.0% | 0.0% | 0.0% | 0.0% | 0.0% | 0.0% | 0.0% | 0.0% | 0.0% | 0.0% | 0.0% | 0.0% | 0.0% | 0.0% | 0.0% | 0.0% | 0.0% | 0.0% | 0.0% | 0.0% |
|  | k\_\_Bacteria;p\_\_Fibrobacteres;c\_\_Fibrobacteria;o\_\_Fibrobacterales;f\_\_Fibrobacteraceae;g\_\_Fibrobacter | 0 | 0.0% | 0.0% | 0.0% | 0.0% | 0.0% | 0.0% | 0.0% | 0.0% | 0.0% | 0.0% | 0.0% | 0.0% | 0.0% | 0.0% | 0.0% | 0.0% | 0.0% | 0.0% | 0.0% | 0.0% | 0.0% | 0.0% | 0.0% | 0.0% |
|  | k\_\_Bacteria;p\_\_Cyanobacteria;c\_\_Oscillatoriophycideae;o\_\_Chroococcales;f\_\_Gomphosphaeriaceae;g\_\_Unclassified\_Gomphosphaeriaceae | 0 | 0.0% | 0.0% | 0.0% | 0.0% | 0.0% | 0.0% | 0.0% | 0.0% | 0.0% | 0.0% | 0.0% | 0.0% | 0.0% | 0.0% | 0.0% | 0.0% | 0.0% | 0.0% | 0.0% | 0.0% | 0.0% | 0.0% | 0.0% | 0.0% |
|  | k\_\_Bacteria;p\_\_Acidobacteria;c\_\_RB25;o\_\_Unclassified\_RB25;f\_\_Unclassified\_RB25;g\_\_Unclassified\_RB25 | 0 | 0.0% | 0.0% | 0.0% | 0.0% | 0.0% | 0.0% | 0.0% | 0.0% | 0.0% | 0.0% | 0.0% | 0.0% | 0.0% | 0.0% | 0.0% | 0.0% | 0.0% | 0.0% | 0.0% | 0.0% | 0.0% | 0.0% | 0.0% | 0.0% |
|  | k\_\_Bacteria;p\_\_Proteobacteria;c\_\_Betaproteobacteria;o\_\_Burkholderiales;f\_\_Oxalobacteraceae;g\_\_Oxalobacter | 0 | 0.0% | 0.0% | 0.0% | 0.0% | 0.0% | 0.0% | 0.0% | 0.0% | 0.0% | 0.0% | 0.0% | 0.0% | 0.0% | 0.0% | 0.0% | 0.0% | 0.0% | 0.0% | 0.0% | 0.0% | 0.0% | 0.0% | 0.0% | 0.0% |
|  | k\_\_Bacteria;p\_\_Proteobacteria;c\_\_Betaproteobacteria;o\_\_Burkholderiales;f\_\_Comamonadaceae;g\_\_Alicycliphilus | 0 | 0.0% | 0.0% | 0.0% | 0.0% | 0.0% | 0.0% | 0.0% | 0.0% | 0.0% | 0.0% | 0.0% | 0.0% | 0.0% | 0.0% | 0.0% | 0.0% | 0.0% | 0.0% | 0.0% | 0.0% | 0.0% | 0.0% | 0.0% | 0.0% |
|  | k\_\_Bacteria;p\_\_Bacteroidetes;c\_\_Bacteroidia;o\_\_Bacteroidales;f\_\_BA008;g\_\_Unclassified\_BA008 | 0 | 0.0% | 0.0% | 0.0% | 0.0% | 0.0% | 0.0% | 0.0% | 0.0% | 0.0% | 0.0% | 0.0% | 0.0% | 0.0% | 0.0% | 0.0% | 0.0% | 0.0% | 0.0% | 0.0% | 0.0% | 0.0% | 0.0% | 0.0% | 0.0% |
|  | k\_\_Bacteria;p\_\_Chloroflexi;c\_\_Chloroflexi;o\_\_AKIW781;f\_\_Unclassified\_AKIW781;g\_\_Unclassified\_AKIW781 | 0 | 0.0% | 0.0% | 0.0% | 0.0% | 0.0% | 0.0% | 0.0% | 0.0% | 0.0% | 0.0% | 0.0% | 0.0% | 0.0% | 0.0% | 0.0% | 0.0% | 0.0% | 0.0% | 0.0% | 0.0% | 0.0% | 0.0% | 0.0% | 0.0% |
|  | k\_\_Bacteria;p\_\_Acidobacteria;c\_\_[Chloracidobacteria];o\_\_DS-100;f\_\_Unclassified\_DS-100;g\_\_Unclassified\_DS-100 | 0 | 0.0% | 0.0% | 0.0% | 0.0% | 0.0% | 0.0% | 0.0% | 0.0% | 0.0% | 0.0% | 0.0% | 0.0% | 0.0% | 0.0% | 0.0% | 0.0% | 0.0% | 0.0% | 0.0% | 0.0% | 0.0% | 0.0% | 0.0% | 0.0% |
|  | k\_\_Bacteria;p\_\_Proteobacteria;c\_\_Gammaproteobacteria;o\_\_Enterobacteriales;f\_\_Enterobacteriaceae;g\_\_Erwinia | 0 | 0.0% | 0.0% | 0.0% | 0.0% | 0.0% | 0.0% | 0.0% | 0.0% | 0.0% | 0.0% | 0.0% | 0.0% | 0.0% | 0.0% | 0.0% | 0.0% | 0.0% | 0.0% | 0.0% | 0.0% | 0.0% | 0.0% | 0.0% | 0.0% |
|  | k\_\_Bacteria;p\_\_Bacteroidetes;c\_\_Bacteroidia;o\_\_Bacteroidales;f\_\_[Odoribacteraceae];g\_\_Odoribacter | 0 | 0.0% | 0.0% | 0.0% | 0.0% | 0.0% | 0.0% | 0.0% | 0.0% | 0.0% | 0.0% | 0.0% | 0.0% | 0.0% | 0.0% | 0.0% | 0.0% | 0.0% | 0.0% | 0.0% | 0.0% | 0.0% | 0.0% | 0.0% | 0.0% |
|  | k\_\_Bacteria;p\_\_Proteobacteria;c\_\_Alphaproteobacteria;o\_\_Caulobacterales;f\_\_Caulobacteraceae;g\_\_Phenylobacterium | 0 | 0.0% | 0.0% | 0.0% | 0.0% | 0.0% | 0.0% | 0.0% | 0.0% | 0.0% | 0.0% | 0.0% | 0.0% | 0.0% | 0.0% | 0.0% | 0.0% | 0.0% | 0.0% | 0.0% | 0.0% | 0.0% | 0.0% | 0.0% | 0.0% |
|  | k\_\_Bacteria;p\_\_Proteobacteria;c\_\_Alphaproteobacteria;o\_\_Rhizobiales;f\_\_Beijerinckiaceae;g\_\_Beijerinckia | 0 | 0.0% | 0.0% | 0.0% | 0.0% | 0.0% | 0.0% | 0.0% | 0.0% | 0.0% | 0.0% | 0.0% | 0.0% | 0.0% | 0.0% | 0.0% | 0.0% | 0.0% | 0.0% | 0.0% | 0.0% | 0.0% | 0.0% | 0.0% | 0.0% |
|  | k\_\_Bacteria;p\_\_Proteobacteria;c\_\_Alphaproteobacteria;o\_\_Rhizobiales;f\_\_Hyphomicrobiaceae;g\_\_Pedomicrobium | 0 | 0.0% | 0.0% | 0.0% | 0.0% | 0.0% | 0.0% | 0.0% | 0.0% | 0.0% | 0.0% | 0.0% | 0.0% | 0.0% | 0.0% | 0.0% | 0.0% | 0.0% | 0.0% | 0.0% | 0.0% | 0.0% | 0.0% | 0.0% | 0.0% |
|  | k\_\_Bacteria;p\_\_Actinobacteria;c\_\_Thermoleophilia;o\_\_Solirubrobacterales;f\_\_Solirubrobacteraceae;g\_\_Unclassified\_Solirubrobacteraceae | 0 | 0.0% | 0.0% | 0.0% | 0.0% | 0.0% | 0.0% | 0.0% | 0.0% | 0.0% | 0.0% | 0.0% | 0.0% | 0.0% | 0.0% | 0.0% | 0.0% | 0.0% | 0.0% | 0.0% | 0.0% | 0.0% | 0.0% | 0.0% | 0.0% |
|  | k\_\_Bacteria;p\_\_Actinobacteria;c\_\_Actinobacteria;o\_\_Actinomycetales;f\_\_Nocardioidaceae;g\_\_Kribbella | 0 | 0.0% | 0.0% | 0.0% | 0.0% | 0.0% | 0.0% | 0.0% | 0.0% | 0.0% | 0.0% | 0.0% | 0.0% | 0.0% | 0.0% | 0.0% | 0.0% | 0.0% | 0.0% | 0.0% | 0.0% | 0.0% | 0.0% | 0.0% | 0.0% |
|  | k\_\_Bacteria;p\_\_Chloroflexi;c\_\_Gitt-GS-136;o\_\_Unclassified\_Gitt-GS-136;f\_\_Unclassified\_Gitt-GS-136;g\_\_Unclassified\_Gitt-GS-136 | 0 | 0.0% | 0.0% | 0.0% | 0.0% | 0.0% | 0.0% | 0.0% | 0.0% | 0.0% | 0.0% | 0.0% | 0.0% | 0.0% | 0.0% | 0.0% | 0.0% | 0.0% | 0.0% | 0.0% | 0.0% | 0.0% | 0.0% | 0.0% | 0.0% |
|  | k\_\_Bacteria;p\_\_Bacteroidetes;c\_\_Cytophagia;o\_\_Cytophagales;f\_\_Unclassified\_Cytophagales;g\_\_Unclassified\_Cytophagales | 0 | 0.0% | 0.0% | 0.0% | 0.0% | 0.0% | 0.0% | 0.0% | 0.0% | 0.0% | 0.0% | 0.0% | 0.0% | 0.0% | 0.0% | 0.0% | 0.0% | 0.0% | 0.0% | 0.0% | 0.0% | 0.0% | 0.0% | 0.0% | 0.0% |
|  | k\_\_Bacteria;p\_\_Elusimicrobia;c\_\_Elusimicrobia;o\_\_Elusimicrobiales;f\_\_Elusimicrobiaceae;g\_\_Unclassified\_Elusimicrobiaceae | 0 | 0.0% | 0.0% | 0.0% | 0.0% | 0.0% | 0.0% | 0.0% | 0.0% | 0.0% | 0.0% | 0.0% | 0.0% | 0.0% | 0.0% | 0.0% | 0.0% | 0.0% | 0.0% | 0.0% | 0.0% | 0.0% | 0.0% | 0.0% | 0.0% |
|  | k\_\_Bacteria;p\_\_Chloroflexi;c\_\_TK10;o\_\_B07\_WMSP1;f\_\_Unclassified\_B07\_WMSP1;g\_\_Unclassified\_B07\_WMSP1 | 0 | 0.0% | 0.0% | 0.0% | 0.0% | 0.0% | 0.0% | 0.0% | 0.0% | 0.0% | 0.0% | 0.0% | 0.0% | 0.0% | 0.0% | 0.0% | 0.0% | 0.0% | 0.0% | 0.0% | 0.0% | 0.0% | 0.0% | 0.0% | 0.0% |
|  | k\_\_Bacteria;p\_\_Actinobacteria;c\_\_Actinobacteria;o\_\_Actinomycetales;f\_\_Micromonosporaceae;g\_\_Micromonospora | 0 | 0.0% | 0.0% | 0.0% | 0.0% | 0.0% | 0.0% | 0.0% | 0.0% | 0.0% | 0.0% | 0.0% | 0.0% | 0.0% | 0.0% | 0.0% | 0.0% | 0.0% | 0.0% | 0.0% | 0.0% | 0.0% | 0.0% | 0.0% | 0.0% |
|  | k\_\_Bacteria;p\_\_Actinobacteria;c\_\_Actinobacteria;o\_\_Actinomycetales;f\_\_Microbacteriaceae;g\_\_Agrococcus | 0 | 0.0% | 0.0% | 0.0% | 0.0% | 0.0% | 0.0% | 0.0% | 0.0% | 0.0% | 0.0% | 0.0% | 0.0% | 0.0% | 0.0% | 0.0% | 0.0% | 0.0% | 0.0% | 0.0% | 0.0% | 0.0% | 0.0% | 0.0% | 0.0% |
|  | k\_\_Bacteria;p\_\_Firmicutes;c\_\_Bacilli;o\_\_Bacillales;f\_\_[Thermicanaceae];g\_\_Thermicanus | 0 | 0.0% | 0.0% | 0.0% | 0.0% | 0.0% | 0.0% | 0.0% | 0.0% | 0.0% | 0.0% | 0.0% | 0.0% | 0.0% | 0.0% | 0.0% | 0.0% | 0.0% | 0.0% | 0.0% | 0.0% | 0.0% | 0.0% | 0.0% | 0.0% |
|  | k\_\_Bacteria;p\_\_Proteobacteria;c\_\_Gammaproteobacteria;o\_\_HOC36;f\_\_Unclassified\_HOC36;g\_\_Unclassified\_HOC36 | 0 | 0.0% | 0.0% | 0.0% | 0.0% | 0.0% | 0.0% | 0.0% | 0.0% | 0.0% | 0.0% | 0.0% | 0.0% | 0.0% | 0.0% | 0.0% | 0.0% | 0.0% | 0.0% | 0.0% | 0.0% | 0.0% | 0.0% | 0.0% | 0.0% |
|  | k\_\_Bacteria;p\_\_Acidobacteria;c\_\_Acidobacteriia;o\_\_Acidobacteriales;f\_\_Koribacteraceae;g\_\_Candidatus\_Koribacter | 0 | 0.0% | 0.0% | 0.0% | 0.0% | 0.0% | 0.0% | 0.0% | 0.0% | 0.0% | 0.0% | 0.0% | 0.0% | 0.0% | 0.0% | 0.0% | 0.0% | 0.0% | 0.0% | 0.0% | 0.0% | 0.0% | 0.0% | 0.0% | 0.0% |
|  | k\_\_Bacteria;p\_\_Actinobacteria;c\_\_Actinobacteria;o\_\_Actinomycetales;f\_\_Dermabacteraceae;g\_\_Dermabacter | 0 | 0.0% | 0.0% | 0.0% | 0.0% | 0.0% | 0.0% | 0.0% | 0.0% | 0.0% | 0.0% | 0.0% | 0.0% | 0.0% | 0.0% | 0.0% | 0.0% | 0.0% | 0.0% | 0.0% | 0.0% | 0.0% | 0.0% | 0.0% | 0.0% |
|  | k\_\_Bacteria;p\_\_Acidobacteria;c\_\_Solibacteres;o\_\_Solibacterales;f\_\_AKIW659;g\_\_Unclassified\_AKIW659 | 0 | 0.0% | 0.0% | 0.0% | 0.0% | 0.0% | 0.0% | 0.0% | 0.0% | 0.0% | 0.0% | 0.0% | 0.0% | 0.0% | 0.0% | 0.0% | 0.0% | 0.0% | 0.0% | 0.0% | 0.0% | 0.0% | 0.0% | 0.0% | 0.0% |
|  | k\_\_Bacteria;p\_\_Actinobacteria;c\_\_Actinobacteria;o\_\_Actinomycetales;f\_\_Pseudonocardiaceae;g\_\_Unclassified\_Pseudonocardiaceae | 0 | 0.0% | 0.0% | 0.0% | 0.0% | 0.0% | 0.0% | 0.0% | 0.0% | 0.0% | 0.0% | 0.0% | 0.0% | 0.0% | 0.0% | 0.0% | 0.0% | 0.0% | 0.0% | 0.0% | 0.0% | 0.0% | 0.0% | 0.0% | 0.0% |
|  | k\_\_Bacteria;p\_\_Bacteroidetes;c\_\_Bacteroidia;o\_\_Bacteroidales;f\_\_[Paraprevotellaceae];g\_\_YRC22 | 0 | 0.0% | 0.0% | 0.0% | 0.0% | 0.0% | 0.0% | 0.0% | 0.0% | 0.0% | 0.0% | 0.0% | 0.0% | 0.0% | 0.0% | 0.0% | 0.0% | 0.0% | 0.0% | 0.0% | 0.0% | 0.0% | 0.0% | 0.0% | 0.0% |
|  | k\_\_Bacteria;p\_\_Tenericutes;c\_\_Mollicutes;o\_\_Unclassified\_Mollicutes;f\_\_Unclassified\_Mollicutes;g\_\_Unclassified\_Mollicutes | 0 | 0.0% | 0.0% | 0.0% | 0.0% | 0.0% | 0.0% | 0.0% | 0.0% | 0.0% | 0.0% | 0.0% | 0.0% | 0.0% | 0.0% | 0.0% | 0.0% | 0.0% | 0.0% | 0.0% | 0.0% | 0.0% | 0.0% | 0.0% | 0.0% |
|  | k\_\_Bacteria;p\_\_Proteobacteria;c\_\_Alphaproteobacteria;o\_\_Rhizobiales;f\_\_Methylocystaceae;g\_\_Methylosinus | 0 | 0.0% | 0.0% | 0.0% | 0.0% | 0.0% | 0.0% | 0.0% | 0.0% | 0.0% | 0.0% | 0.0% | 0.0% | 0.0% | 0.0% | 0.0% | 0.0% | 0.0% | 0.0% | 0.0% | 0.0% | 0.0% | 0.0% | 0.0% | 0.0% |
|  | k\_\_Bacteria;p\_\_Acidobacteria;c\_\_[Chloracidobacteria];o\_\_RB41;f\_\_Ellin6075;g\_\_Unclassified\_Ellin6075 | 0 | 0.0% | 0.0% | 0.0% | 0.0% | 0.0% | 0.0% | 0.0% | 0.0% | 0.0% | 0.0% | 0.0% | 0.0% | 0.0% | 0.0% | 0.0% | 0.0% | 0.0% | 0.0% | 0.0% | 0.0% | 0.0% | 0.0% | 0.0% | 0.0% |
|  | k\_\_Bacteria;p\_\_Proteobacteria;c\_\_Deltaproteobacteria;o\_\_BPC076;f\_\_Unclassified\_BPC076;g\_\_Unclassified\_BPC076 | 0 | 0.0% | 0.0% | 0.0% | 0.0% | 0.0% | 0.0% | 0.0% | 0.0% | 0.0% | 0.0% | 0.0% | 0.0% | 0.0% | 0.0% | 0.0% | 0.0% | 0.0% | 0.0% | 0.0% | 0.0% | 0.0% | 0.0% | 0.0% | 0.0% |
|  | k\_\_Bacteria;p\_\_Nitrospirae;c\_\_Nitrospira;o\_\_Nitrospirales;f\_\_[Thermodesulfovibrionaceae];g\_\_LCP-6 | 0 | 0.0% | 0.0% | 0.0% | 0.0% | 0.0% | 0.0% | 0.0% | 0.0% | 0.0% | 0.0% | 0.0% | 0.0% | 0.0% | 0.0% | 0.0% | 0.0% | 0.0% | 0.0% | 0.0% | 0.0% | 0.0% | 0.0% | 0.0% | 0.0% |
|  | k\_\_Bacteria;p\_\_Proteobacteria;c\_\_Betaproteobacteria;o\_\_Rhodocyclales;f\_\_Rhodocyclaceae;g\_\_KD1-23 | 0 | 0.0% | 0.0% | 0.0% | 0.0% | 0.0% | 0.0% | 0.0% | 0.0% | 0.0% | 0.0% | 0.0% | 0.0% | 0.0% | 0.0% | 0.0% | 0.0% | 0.0% | 0.0% | 0.0% | 0.0% | 0.0% | 0.0% | 0.0% | 0.0% |
|  | k\_\_Bacteria;p\_\_Actinobacteria;c\_\_Actinobacteria;o\_\_Actinomycetales;f\_\_Intrasporangiaceae;g\_\_Unclassified\_Intrasporangiaceae | 0 | 0.0% | 0.0% | 0.0% | 0.0% | 0.0% | 0.0% | 0.0% | 0.0% | 0.0% | 0.0% | 0.0% | 0.0% | 0.0% | 0.0% | 0.0% | 0.0% | 0.0% | 0.0% | 0.0% | 0.0% | 0.0% | 0.0% | 0.0% | 0.0% |
|  | k\_\_Bacteria;p\_\_Acidobacteria;c\_\_Sva0725;o\_\_Sva0725;f\_\_Unclassified\_Sva0725;g\_\_Unclassified\_Sva0725 | 0 | 0.0% | 0.0% | 0.0% | 0.0% | 0.0% | 0.0% | 0.0% | 0.0% | 0.0% | 0.0% | 0.0% | 0.0% | 0.0% | 0.0% | 0.0% | 0.0% | 0.0% | 0.0% | 0.0% | 0.0% | 0.0% | 0.0% | 0.0% | 0.0% |
|  | k\_\_Bacteria;p\_\_Firmicutes;c\_\_Bacilli;o\_\_Lactobacillales;f\_\_Carnobacteriaceae;g\_\_Granulicatella | 0 | 0.0% | 0.0% | 0.0% | 0.0% | 0.0% | 0.0% | 0.0% | 0.0% | 0.0% | 0.0% | 0.0% | 0.0% | 0.0% | 0.0% | 0.0% | 0.0% | 0.0% | 0.0% | 0.0% | 0.0% | 0.0% | 0.0% | 0.0% | 0.0% |
|  | k\_\_Bacteria;p\_\_Bacteroidetes;c\_\_Bacteroidia;o\_\_Bacteroidales;f\_\_Porphyromonadaceae;g\_\_Paludibacter | 0 | 0.0% | 0.0% | 0.0% | 0.0% | 0.0% | 0.0% | 0.0% | 0.0% | 0.0% | 0.0% | 0.0% | 0.0% | 0.0% | 0.0% | 0.0% | 0.0% | 0.0% | 0.0% | 0.0% | 0.0% | 0.0% | 0.0% | 0.0% | 0.0% |
|  | k\_\_Bacteria;p\_\_Actinobacteria;c\_\_Actinobacteria;o\_\_Actinomycetales;f\_\_Nocardioidaceae;g\_\_Propionicimonas | 0 | 0.0% | 0.0% | 0.0% | 0.0% | 0.0% | 0.0% | 0.0% | 0.0% | 0.0% | 0.0% | 0.0% | 0.0% | 0.0% | 0.0% | 0.0% | 0.0% | 0.0% | 0.0% | 0.0% | 0.0% | 0.0% | 0.0% | 0.0% | 0.0% |
|  | k\_\_Bacteria;p\_\_Gemmatimonadetes;c\_\_Gemmatimonadetes;o\_\_C114;f\_\_Unclassified\_C114;g\_\_Unclassified\_C114 | 0 | 0.0% | 0.0% | 0.0% | 0.0% | 0.0% | 0.0% | 0.0% | 0.0% | 0.0% | 0.0% | 0.0% | 0.0% | 0.0% | 0.0% | 0.0% | 0.0% | 0.0% | 0.0% | 0.0% | 0.0% | 0.0% | 0.0% | 0.0% | 0.0% |
|  | k\_\_Bacteria;p\_\_Proteobacteria;c\_\_Gammaproteobacteria;o\_\_Alteromonadales;f\_\_Alteromonadaceae;g\_\_Marinobacter | 0 | 0.0% | 0.0% | 0.0% | 0.0% | 0.0% | 0.0% | 0.0% | 0.0% | 0.0% | 0.0% | 0.0% | 0.0% | 0.0% | 0.0% | 0.0% | 0.0% | 0.0% | 0.0% | 0.0% | 0.0% | 0.0% | 0.0% | 0.0% | 0.0% |
|  | k\_\_Bacteria;p\_\_Acidobacteria;c\_\_BPC102;o\_\_MVS-40;f\_\_Unclassified\_MVS-40;g\_\_Unclassified\_MVS-40 | 0 | 0.0% | 0.0% | 0.0% | 0.0% | 0.0% | 0.0% | 0.0% | 0.0% | 0.0% | 0.0% | 0.0% | 0.0% | 0.0% | 0.0% | 0.0% | 0.0% | 0.0% | 0.0% | 0.0% | 0.0% | 0.0% | 0.0% | 0.0% | 0.0% |
|  | k\_\_Bacteria;p\_\_Firmicutes;c\_\_Clostridia;o\_\_Clostridiales;f\_\_Clostridiaceae;g\_\_02d06 | 0 | 0.0% | 0.0% | 0.0% | 0.0% | 0.0% | 0.0% | 0.0% | 0.0% | 0.0% | 0.0% | 0.0% | 0.0% | 0.0% | 0.0% | 0.0% | 0.0% | 0.0% | 0.0% | 0.0% | 0.0% | 0.0% | 0.0% | 0.0% | 0.0% |
|  | k\_\_Bacteria;p\_\_Proteobacteria;c\_\_Betaproteobacteria;o\_\_Burkholderiales;f\_\_Comamonadaceae;g\_\_Rhodoferax | 0 | 0.0% | 0.0% | 0.0% | 0.0% | 0.0% | 0.0% | 0.0% | 0.0% | 0.0% | 0.0% | 0.0% | 0.0% | 0.0% | 0.0% | 0.0% | 0.0% | 0.0% | 0.0% | 0.0% | 0.0% | 0.0% | 0.0% | 0.0% | 0.0% |
|  | k\_\_Bacteria;p\_\_Actinobacteria;c\_\_Actinobacteria;o\_\_Actinomycetales;f\_\_Nocardioidaceae;g\_\_Nocardioides | 0 | 0.0% | 0.0% | 0.0% | 0.0% | 0.0% | 0.0% | 0.0% | 0.0% | 0.0% | 0.0% | 0.0% | 0.0% | 0.0% | 0.0% | 0.0% | 0.0% | 0.0% | 0.0% | 0.0% | 0.0% | 0.0% | 0.0% | 0.0% | 0.0% |
|  | k\_\_Bacteria;p\_\_Chloroflexi;c\_\_Anaerolineae;o\_\_SBR1031;f\_\_SJA-101;g\_\_Unclassified\_SJA-101 | 0 | 0.0% | 0.0% | 0.0% | 0.0% | 0.0% | 0.0% | 0.0% | 0.0% | 0.0% | 0.0% | 0.0% | 0.0% | 0.0% | 0.0% | 0.0% | 0.0% | 0.0% | 0.0% | 0.0% | 0.0% | 0.0% | 0.0% | 0.0% | 0.0% |
|  | k\_\_Bacteria;p\_\_Firmicutes;c\_\_Clostridia;o\_\_Clostridiales;f\_\_Ruminococcaceae;g\_\_Butyricicoccus | 0 | 0.0% | 0.0% | 0.0% | 0.0% | 0.0% | 0.0% | 0.0% | 0.0% | 0.0% | 0.0% | 0.0% | 0.0% | 0.0% | 0.0% | 0.0% | 0.0% | 0.0% | 0.0% | 0.0% | 0.0% | 0.0% | 0.0% | 0.0% | 0.0% |
|  | k\_\_Bacteria;p\_\_Bacteroidetes;c\_\_Bacteroidia;o\_\_Bacteroidales;f\_\_Marinilabiaceae;g\_\_Unclassified\_Marinilabiaceae | 0 | 0.0% | 0.0% | 0.0% | 0.0% | 0.0% | 0.0% | 0.0% | 0.0% | 0.0% | 0.0% | 0.0% | 0.0% | 0.0% | 0.0% | 0.0% | 0.0% | 0.0% | 0.0% | 0.0% | 0.0% | 0.0% | 0.0% | 0.0% | 0.0% |
|  | k\_\_Bacteria;p\_\_Firmicutes;c\_\_Bacilli;o\_\_Gemellales;f\_\_Gemellaceae;g\_\_Unclassified\_Gemellaceae | 0 | 0.0% | 0.0% | 0.0% | 0.0% | 0.0% | 0.0% | 0.0% | 0.0% | 0.0% | 0.0% | 0.0% | 0.0% | 0.0% | 0.0% | 0.0% | 0.0% | 0.0% | 0.0% | 0.0% | 0.0% | 0.0% | 0.0% | 0.0% | 0.0% |
|  | k\_\_Bacteria;p\_\_Fibrobacteres;c\_\_Fibrobacteria;o\_\_258ds10;f\_\_Unclassified\_258ds10;g\_\_Unclassified\_258ds10 | 0 | 0.0% | 0.0% | 0.0% | 0.0% | 0.0% | 0.0% | 0.0% | 0.0% | 0.0% | 0.0% | 0.0% | 0.0% | 0.0% | 0.0% | 0.0% | 0.0% | 0.0% | 0.0% | 0.0% | 0.0% | 0.0% | 0.0% | 0.0% | 0.0% |
|  | k\_\_Bacteria;p\_\_Chloroflexi;c\_\_Anaerolineae;o\_\_Anaerolineales;f\_\_Anaerolinaceae;g\_\_Unclassified\_Anaerolinaceae | 0 | 0.0% | 0.0% | 0.0% | 0.0% | 0.0% | 0.0% | 0.0% | 0.0% | 0.0% | 0.0% | 0.0% | 0.0% | 0.0% | 0.0% | 0.0% | 0.0% | 0.0% | 0.0% | 0.0% | 0.0% | 0.0% | 0.0% | 0.0% | 0.0% |
|  | k\_\_Bacteria;p\_\_Fusobacteria;c\_\_Fusobacteriia;o\_\_Fusobacteriales;f\_\_Fusobacteriaceae;g\_\_u114 | 0 | 0.0% | 0.0% | 0.0% | 0.0% | 0.0% | 0.0% | 0.0% | 0.0% | 0.0% | 0.0% | 0.0% | 0.0% | 0.0% | 0.0% | 0.0% | 0.0% | 0.0% | 0.0% | 0.0% | 0.0% | 0.0% | 0.0% | 0.0% | 0.0% |
|  | k\_\_Bacteria;p\_\_Proteobacteria;c\_\_Gammaproteobacteria;o\_\_Pasteurellales;f\_\_Pasteurellaceae;g\_\_Aggregatibacter | 0 | 0.0% | 0.0% | 0.0% | 0.0% | 0.0% | 0.0% | 0.0% | 0.0% | 0.0% | 0.0% | 0.0% | 0.0% | 0.0% | 0.0% | 0.0% | 0.0% | 0.0% | 0.0% | 0.0% | 0.0% | 0.0% | 0.0% | 0.0% | 0.0% |
|  | k\_\_Bacteria;p\_\_Actinobacteria;c\_\_Thermoleophilia;o\_\_Gaiellales;f\_\_Unclassified\_Gaiellales;g\_\_Unclassified\_Gaiellales | 0 | 0.0% | 0.0% | 0.0% | 0.0% | 0.0% | 0.0% | 0.0% | 0.0% | 0.0% | 0.0% | 0.0% | 0.0% | 0.0% | 0.0% | 0.0% | 0.0% | 0.0% | 0.0% | 0.0% | 0.0% | 0.0% | 0.0% | 0.0% | 0.0% |
|  | k\_\_Bacteria;p\_\_Acidobacteria;c\_\_AT-s54;o\_\_Unclassified\_AT-s54;f\_\_Unclassified\_AT-s54;g\_\_Unclassified\_AT-s54 | 0 | 0.0% | 0.0% | 0.0% | 0.0% | 0.0% | 0.0% | 0.0% | 0.0% | 0.0% | 0.0% | 0.0% | 0.0% | 0.0% | 0.0% | 0.0% | 0.0% | 0.0% | 0.0% | 0.0% | 0.0% | 0.0% | 0.0% | 0.0% | 0.0% |
|  | k\_\_Bacteria;p\_\_Actinobacteria;c\_\_Actinobacteria;o\_\_Actinomycetales;f\_\_Micromonosporaceae;g\_\_Dactylosporangium | 0 | 0.0% | 0.0% | 0.0% | 0.0% | 0.0% | 0.0% | 0.0% | 0.0% | 0.0% | 0.0% | 0.0% | 0.0% | 0.0% | 0.0% | 0.0% | 0.0% | 0.0% | 0.0% | 0.0% | 0.0% | 0.0% | 0.0% | 0.0% | 0.0% |
|  | k\_\_Bacteria;p\_\_Bacteroidetes;c\_\_[Saprospirae];o\_\_[Saprospirales];f\_\_Unclassified\_[Saprospirales];g\_\_Unclassified\_[Saprospirales] | 0 | 0.0% | 0.0% | 0.0% | 0.0% | 0.0% | 0.0% | 0.0% | 0.0% | 0.0% | 0.0% | 0.0% | 0.0% | 0.0% | 0.0% | 0.0% | 0.0% | 0.0% | 0.0% | 0.0% | 0.0% | 0.0% | 0.0% | 0.0% | 0.0% |
|  | k\_\_Bacteria;p\_\_Acidobacteria;c\_\_Acidobacteria-6;o\_\_iii1-15;f\_\_RB40;g\_\_Unclassified\_RB40 | 0 | 0.0% | 0.0% | 0.0% | 0.0% | 0.0% | 0.0% | 0.0% | 0.0% | 0.0% | 0.0% | 0.0% | 0.0% | 0.0% | 0.0% | 0.0% | 0.0% | 0.0% | 0.0% | 0.0% | 0.0% | 0.0% | 0.0% | 0.0% | 0.0% |
|  | k\_\_Bacteria;p\_\_Proteobacteria;c\_\_Deltaproteobacteria;o\_\_Myxococcales;f\_\_Cystobacterineae;g\_\_Unclassified\_Cystobacterineae | 0 | 0.0% | 0.0% | 0.0% | 0.0% | 0.0% | 0.0% | 0.0% | 0.0% | 0.0% | 0.0% | 0.0% | 0.0% | 0.0% | 0.0% | 0.0% | 0.0% | 0.0% | 0.0% | 0.0% | 0.0% | 0.0% | 0.0% | 0.0% | 0.0% |
|  | k\_\_Bacteria;p\_\_OD1;c\_\_ABY1;o\_\_Unclassified\_ABY1;f\_\_Unclassified\_ABY1;g\_\_Unclassified\_ABY1 | 0 | 0.0% | 0.0% | 0.0% | 0.0% | 0.0% | 0.0% | 0.0% | 0.0% | 0.0% | 0.0% | 0.0% | 0.0% | 0.0% | 0.0% | 0.0% | 0.0% | 0.0% | 0.0% | 0.0% | 0.0% | 0.0% | 0.0% | 0.0% | 0.0% |
|  | k\_\_Bacteria;p\_\_Proteobacteria;c\_\_Deltaproteobacteria;o\_\_Spirobacillales;f\_\_Unclassified\_Spirobacillales;g\_\_Unclassified\_Spirobacillales | 0 | 0.0% | 0.0% | 0.0% | 0.0% | 0.0% | 0.0% | 0.0% | 0.0% | 0.0% | 0.0% | 0.0% | 0.0% | 0.0% | 0.0% | 0.0% | 0.0% | 0.0% | 0.0% | 0.0% | 0.0% | 0.0% | 0.0% | 0.0% | 0.0% |
|  | k\_\_Bacteria;p\_\_Actinobacteria;c\_\_Actinobacteria;o\_\_Actinomycetales;f\_\_Microbacteriaceae;g\_\_Unclassified\_Microbacteriaceae | 0 | 0.0% | 0.0% | 0.0% | 0.0% | 0.0% | 0.0% | 0.0% | 0.0% | 0.0% | 0.0% | 0.0% | 0.0% | 0.0% | 0.0% | 0.0% | 0.0% | 0.0% | 0.0% | 0.0% | 0.0% | 0.0% | 0.0% | 0.0% | 0.0% |
|  | k\_\_Bacteria;p\_\_Armatimonadetes;c\_\_0319-6E2;o\_\_Unclassified\_0319-6E2;f\_\_Unclassified\_0319-6E2;g\_\_Unclassified\_0319-6E2 | 0 | 0.0% | 0.0% | 0.0% | 0.0% | 0.0% | 0.0% | 0.0% | 0.0% | 0.0% | 0.0% | 0.0% | 0.0% | 0.0% | 0.0% | 0.0% | 0.0% | 0.0% | 0.0% | 0.0% | 0.0% | 0.0% | 0.0% | 0.0% | 0.0% |
|  | k\_\_Bacteria;p\_\_Gemmatimonadetes;c\_\_Gemmatimonadetes;o\_\_KD8-87;f\_\_Unclassified\_KD8-87;g\_\_Unclassified\_KD8-87 | 0 | 0.0% | 0.0% | 0.0% | 0.0% | 0.0% | 0.0% | 0.0% | 0.0% | 0.0% | 0.0% | 0.0% | 0.0% | 0.0% | 0.0% | 0.0% | 0.0% | 0.0% | 0.0% | 0.0% | 0.0% | 0.0% | 0.0% | 0.0% | 0.0% |
|  | k\_\_Bacteria;p\_\_Planctomycetes;c\_\_OM190;o\_\_CL500-15;f\_\_Unclassified\_CL500-15;g\_\_Unclassified\_CL500-15 | 0 | 0.0% | 0.0% | 0.0% | 0.0% | 0.0% | 0.0% | 0.0% | 0.0% | 0.0% | 0.0% | 0.0% | 0.0% | 0.0% | 0.0% | 0.0% | 0.0% | 0.0% | 0.0% | 0.0% | 0.0% | 0.0% | 0.0% | 0.0% | 0.0% |
|  | k\_\_Bacteria;p\_\_Proteobacteria;c\_\_Gammaproteobacteria;o\_\_Xanthomonadales;f\_\_Xanthomonadaceae;g\_\_Stenotrophomonas | 0 | 0.0% | 0.0% | 0.0% | 0.0% | 0.0% | 0.0% | 0.0% | 0.0% | 0.0% | 0.0% | 0.0% | 0.0% | 0.0% | 0.0% | 0.0% | 0.0% | 0.0% | 0.0% | 0.0% | 0.0% | 0.0% | 0.0% | 0.0% | 0.0% |
