# Supplementary material for: The effects of genital myiasis on the diversity of the vaginal microbiota in female Bactrian camels
Source: BMC Vet Res. 2022 Mar 5;18:87. doi: 10.1186/s12917-022-03189-5 (PMC8897907; doi:10.1186/s12917-022-03189-5)
Supplement: Supplementary file 5 — Additional file 5. [file 12917_2022_3189_MOESM5_ESM.zip › MPL201709200_16s_yy/Treat1/B07_taxa_summary_group/taxa_summary_plots/charts/DbtzqzZQa5UQSuDzxnOXZRJCqrtL0Y_legend.pdf]

k__Bacteria;p__Firmicutes;c__Clostridia;o__Clostridiales
k__Bacteria;p__Fusobacteria;c__Fusobacteriia;o__Fusobacteriales
k__Bacteria;p__Firmicutes;c__Bacilli;o__Lactobacillales
k__Bacteria;p__Proteobacteria;c__Epsilonproteobacteria;o__Campylobacterales
k__Bacteria;p__Proteobacteria;c__Alphaproteobacteria;o__Rhizobiales
k__Bacteria;p__Actinobacteria;c__Actinobacteria;o__Actinomycetales
k__Bacteria;p__Bacteroidetes;c__Bacteroidia;o__Bacteroidales
k__Bacteria;p__Proteobacteria;c__Betaproteobacteria;o__Burkholderiales
k__Bacteria;p__Proteobacteria;c__Gammaproteobacteria;o__Pseudomonadales
k__Bacteria;p__Proteobacteria;c__Gammaproteobacteria;o__Xanthomonadales
k__Bacteria;p__Bacteroidetes;c__[Saprospirae];o__[Saprospirales]
No blast hit;Other;Other;Other
k__Bacteria;p__Proteobacteria;c__Alphaproteobacteria;o__Sphingomonadales
k__Bacteria;p__Proteobacteria;c__Gammaproteobacteria;o__Enterobacteriales
k__Bacteria;p__Proteobacteria;c__Alphaproteobacteria;o__Caulobacterales
k__Bacteria;p__Cyanobacteria;c__4C0d-2;o__MLE1-12
k__Bacteria;p__Proteobacteria;c__Betaproteobacteria;o__Rhodocyclales
k__Bacteria;p__Firmicutes;c__Bacilli;o__Bacillales
k__Bacteria;p__Actinobacteria;c__Actinobacteria;o__Bifidobacteriales
k__Bacteria;p__Actinobacteria;c__Coriobacteriia;o__Coriobacteriales
k__Bacteria;p__Proteobacteria;c__Betaproteobacteria;o__Neisseriales
k__Bacteria;p__Proteobacteria;c__Gammaproteobacteria;o__Pasteurellales
k__Bacteria;p__Proteobacteria;c__Deltaproteobacteria;o__Desulfovibrionales
k__Bacteria;p__Proteobacteria;c__Deltaproteobacteria;o__Myxococcales
k__Bacteria;p__Firmicutes;c__Erysipelotrichi;o__Erysipelotrichales
k__Bacteria;p__Tenericutes;c__Mollicutes;o__Acholeplasmatales
k__Bacteria;p__Bacteroidetes;c__Flavobacteriia;o__Flavobacteriales
k__Bacteria;p__Verrucomicrobia;c__Verrucomicrobiae;o__Verrucomicrobiales
k__Bacteria;p__Proteobacteria;c__Alphaproteobacteria;o__Rhodospirillales
k__Bacteria;p__GN02;c__3BR-5F;o__Unclassified_3BR-5F
k__Bacteria;p__Cyanobacteria;c__Chloroplast;o__Streptophyta
k__Bacteria;p__Proteobacteria;c__Gammaproteobacteria;o__Aeromonadales
k__Bacteria;p__SR1;c__Unclassified_SR1;o__Unclassified_SR1
k__Bacteria;p__Verrucomicrobia;c__Verruco-5;o__WCHB1-41
k__Bacteria;p__Lentisphaerae;c__[Lentisphaeria];o__Victivallales
k__Bacteria;p__Bacteroidetes;c__Cytophagia;o__Cytophagales
k__Bacteria;p__Spirochaetes;c__Spirochaetes;o__Spirochaetales
k__Bacteria;p__Planctomycetes;c__Phycisphaerae;o__Phycisphaerales
k__Bacteria;p__Gemmatimonadetes;c__Gemm-1;o__Unclassified_Gemm-1
k__Bacteria;p__Acidobacteria;c__Solibacteres;o__Solibacterales
k__Bacteria;p__[Thermi];c__Deinococci;o__Deinococcales
k__Bacteria;p__Acidobacteria;c__Acidobacteria-6;o__iii1-15
k__Bacteria;p__Proteobacteria;c__Alphaproteobacteria;o__Rhodobacterales
k__Bacteria;p__Chloroflexi;c__Anaerolineae;o__SBR1031
k__Bacteria;p__Proteobacteria;c__Betaproteobacteria;o__SC-I-84
k__Bacteria;p__TM7;c__TM7-3;o__CW040
k__Bacteria;p__Cyanobacteria;c__4C0d-2;o__YS2
k__Bacteria;p__Actinobacteria;c__Rubrobacteria;o__Rubrobacterales
k__Bacteria;p__Actinobacteria;c__Acidimicrobiia;o__Acidimicrobiales
k__Bacteria;p__Tenericutes;c__Mollicutes;o__Mycoplasmatales
k__Bacteria;p__Firmicutes;c__Bacilli;o__Turicibacterales
k__Bacteria;p__Proteobacteria;c__Gammaproteobacteria;o__Cardiobacteriales
k__Bacteria;p__Nitrospirae;c__Nitrospira;o__Nitrospirales
k__Bacteria;p__Bacteroidetes;c__Sphingobacteriia;o__Sphingobacteriales
k__Bacteria;p__Gemmatimonadetes;c__Gemmatimonadetes;o__Gemmatimonadales
k__Bacteria;p__Gemmatimonadetes;c__Gemmatimonadetes;o__Unclassified_Gemmatimonadetes
k__Bacteria;p__Tenericutes;c__Mollicutes;o__RF39
k__Bacteria;p__Proteobacteria;c__Alphaproteobacteria;o__Unclassified_Alphaproteobacteria
k__Bacteria;p__Acidobacteria;c__Acidobacteriia;o__Acidobacteriales
k__Bacteria;p__Planctomycetes;c__Planctomycetia;o__Gemmatales
k__Bacteria;p__Proteobacteria;c__Betaproteobacteria;o__MND1
k__Bacteria;p__Proteobacteria;c__Alphaproteobacteria;o__Rickettsiales
k__Bacteria;p__Proteobacteria;c__Deltaproteobacteria;o__Syntrophobacterales
k__Bacteria;p__Lentisphaerae;c__[Lentisphaeria];o__Unclassified_[Lentisphaeria]
k__Bacteria;p__Firmicutes;c__Bacilli;o__Gemellales
k__Bacteria;p__Chlamydiae;c__Chlamydiia;o__Chlamydiales
k__Bacteria;p__Actinobacteria;c__Thermoleophilia;o__Gaiellales
k__Bacteria;p__TM7;c__TM7-3;o__Unclassified_TM7-3
k__Bacteria;p__Gemmatimonadetes;c__Gemmatimonadetes;o__N1423WL
k__Bacteria;p__WS3;c__PRR-12;o__Sediment-1
k__Bacteria;p__Proteobacteria;c__Deltaproteobacteria;o__MIZ46
k__Bacteria;p__Proteobacteria;c__Deltaproteobacteria;o__NB1-j
k__Bacteria;p__Proteobacteria;c__Deltaproteobacteria;o__Bdellovibrionales
k__Bacteria;p__Chloroflexi;c__Anaerolineae;o__H39
k__Bacteria;p__Proteobacteria;c__Betaproteobacteria;o__Unclassified_Betaproteobacteria
k__Bacteria;p__Tenericutes;c__RF3;o__ML615J-28
k__Bacteria;p__Actinobacteria;c__Thermoleophilia;o__Solirubrobacterales
k__Bacteria;p__Proteobacteria;c__Gammaproteobacteria;o__Thiotrichales
k__Bacteria;p__Acidobacteria;c__[Chloracidobacteria];o__RB41
k__Bacteria;p__Armatimonadetes;c__[Fimbriimonadia];o__[Fimbriimonadales]
k__Bacteria;p__Proteobacteria;c__Betaproteobacteria;o__Hydrogenophilales
k__Bacteria;p__Proteobacteria;c__Gammaproteobacteria;o__Alteromonadales
k__Bacteria;p__Proteobacteria;c__Deltaproteobacteria;o__GMD14H09
k__Bacteria;p__Chloroflexi;c__S085;o__Unclassified_S085
k__Bacteria;p__OD1;c__ZB2;o__Unclassified_ZB2
k__Bacteria;p__WPS-2;c__Unclassified_WPS-2;o__Unclassified_WPS-2
k__Bacteria;p__AD3;c__ABS-6;o__Unclassified_ABS-6
k__Bacteria;p__Chloroflexi;c__Thermomicrobia;o__JG30-KF-CM45
k__Bacteria;p__Chloroflexi;c__Chloroflexi;o__[Roseiflexales]
k__Bacteria;p__[Thermi];c__Deinococci;o__Thermales
k__Bacteria;p__Acidobacteria;c__Acidobacteria-6;o__CCU21
k__Bacteria;p__Proteobacteria;c__Betaproteobacteria;o__Ellin6067
k__Bacteria;p__Armatimonadetes;c__Chthonomonadetes;o__Chthonomonadales
k__Bacteria;p__Proteobacteria;c__Alphaproteobacteria;o__BD7-3
k__Bacteria;p__Proteobacteria;c__Deltaproteobacteria;o__MBNT15
k__Bacteria;p__Chloroflexi;c__Anaerolineae;o__GCA004
k__Bacteria;p__Tenericutes;c__CK-1C4-19;o__Unclassified_CK-1C4-19
k__Bacteria;p__Spirochaetes;c__Spirochaetes;o__Sphaerochaetales
k__Bacteria;p__Deferribacteres;c__Deferribacteres;o__Deferribacterales
k__Bacteria;p__Gemmatimonadetes;c__Gemmatimonadetes;o__Ellin5290
k__Bacteria;p__Proteobacteria;c__Deltaproteobacteria;o__Desulfobacterales
k__Bacteria;p__Cyanobacteria;c__Chloroplast;o__Stramenopiles
k__Bacteria;p__Proteobacteria;c__Betaproteobacteria;o__IS-44
k__Bacteria;p__Chloroflexi;c__Anaerolineae;o__CFB-26
k__Bacteria;p__Acidobacteria;c__DA052;o__Ellin6513
k__Bacteria;p__Chloroflexi;c__Ellin6529;o__Unclassified_Ellin6529
k__Bacteria;p__GAL15;c__Unclassified_GAL15;o__Unclassified_GAL15
k__Bacteria;p__Planctomycetes;c__C6;o__MVS-107
k__Bacteria;p__Lentisphaerae;c__[Lentisphaeria];o__Z20
k__Bacteria;p__Chloroflexi;c__TK17;o__Unclassified_TK17
k__Bacteria;p__Chloroflexi;c__Anaerolineae;o__DRC31
k__Bacteria;p__Chlorobi;c__SJA-28;o__Unclassified_SJA-28
k__Bacteria;p__Chloroflexi;c__Ktedonobacteria;o__JG30-KF-AS9
k__Bacteria;p__Elusimicrobia;c__Elusimicrobia;o__Elusimicrobiales
k__Bacteria;p__Gemmatimonadetes;c__Gemm-5;o__Unclassified_Gemm-5
k__Bacteria;p__Proteobacteria;c__Deltaproteobacteria;o__Desulfuromonadales
k__Bacteria;p__Firmicutes;c__Clostridia;o__Thermoanaerobacterales
k__Bacteria;p__Chloroflexi;c__Anaerolineae;o__Caldilineales
k__Bacteria;p__Proteobacteria;c__Alphaproteobacteria;o__RF32
k__Bacteria;p__Proteobacteria;c__Gammaproteobacteria;o__Vibrionales
k__Bacteria;p__Proteobacteria;c__Gammaproteobacteria;o__Unclassified_Gammaproteobacteria
k__Bacteria;p__Verrucomicrobia;c__Opitutae;o__[Cerasicoccales]
k__Bacteria;p__Proteobacteria;c__Betaproteobacteria;o__Methylophilales
k__Bacteria;p__Proteobacteria;c__Gammaproteobacteria;o__[Marinicellales]
k__Bacteria;p__Proteobacteria;c__Gammaproteobacteria;o__Oceanospirillales
k__Bacteria;p__Chloroflexi;c__Anaerolineae;o__S0208
k__Bacteria;p__Planctomycetes;c__OM190;o__agg27
k__Bacteria;p__Acidobacteria;c__RB25;o__Unclassified_RB25
k__Bacteria;p__TM7;c__TM7-1;o__Unclassified_TM7-1
k__Bacteria;p__Acidobacteria;c__[Chloracidobacteria];o__DS-100
k__Bacteria;p__Chloroflexi;c__Gitt-GS-136;o__Unclassified_Gitt-GS-136
k__Bacteria;p__Cyanobacteria;c__ML635J-21;o__Unclassified_ML635J-21
k__Bacteria;p__Chloroflexi;c__TK10;o__B07_WMSP1
k__Bacteria;p__Proteobacteria;c__Gammaproteobacteria;o__HOC36
k__Bacteria;p__Cyanobacteria;c__Oscillatoriophycideae;o__Chroococcales
k__Bacteria;p__Fibrobacteres;c__Fibrobacteria;o__Fibrobacterales
k__Bacteria;p__Tenericutes;c__Mollicutes;o__Unclassified_Mollicutes
k__Bacteria;p__Chloroflexi;c__Chloroflexi;o__AKIW781
k__Bacteria;p__Acidobacteria;c__Sva0725;o__Sva0725
k__Bacteria;p__Gemmatimonadetes;c__Gemmatimonadetes;o__C114
k__Bacteria;p__Acidobacteria;c__AT-s54;o__Unclassified_AT-s54
k__Bacteria;p__Proteobacteria;c__Deltaproteobacteria;o__BPC076
k__Bacteria;p__Armatimonadetes;c__0319-6E2;o__Unclassified_0319-6E2
k__Bacteria;p__Gemmatimonadetes;c__Gemmatimonadetes;o__KD8-87
k__Bacteria;p__Planctomycetes;c__OM190;o__CL500-15
k__Bacteria;p__Acidobacteria;c__BPC102;o__MVS-40
k__Bacteria;p__Chloroflexi;c__Anaerolineae;o__Anaerolineales
k__Bacteria;p__Fibrobacteres;c__Fibrobacteria;o__258ds10
k__Bacteria;p__OD1;c__ABY1;o__Unclassified_ABY1
k__Bacteria;p__Proteobacteria;c__Deltaproteobacteria;o__Spirobacillales
