# Supplementary material for: The effects of genital myiasis on the diversity of the vaginal microbiota in female Bactrian camels
Source: BMC Vet Res. 2022 Mar 5;18:87. doi: 10.1186/s12917-022-03189-5 (PMC8897907; doi:10.1186/s12917-022-03189-5)
Supplement: Supplementary file 5 — Additional file 5. [file 12917_2022_3189_MOESM5_ESM.zip › MPL201709200_16s_yy/Treat1/B07_taxa_summary_group/taxa_summary_plots/charts/KriMcTu5Hl7hszkXJ0WgLraurHuhS6_legend.pdf]

k__Bacteria;p__Firmicutes
k__Bacteria;p__Proteobacteria
k__Bacteria;p__Fusobacteria
k__Bacteria;p__Bacteroidetes
k__Bacteria;p__Actinobacteria
No blast hit;Other
k__Bacteria;p__Cyanobacteria
k__Bacteria;p__Verrucomicrobia
k__Bacteria;p__Tenericutes
k__Bacteria;p__Acidobacteria
k__Bacteria;p__Gemmatimonadetes
k__Bacteria;p__Chloroflexi
k__Bacteria;p__GN02
k__Bacteria;p__Lentisphaerae
k__Bacteria;p__Planctomycetes
k__Bacteria;p__SR1
k__Bacteria;p__Spirochaetes
k__Bacteria;p__[Thermi]
k__Bacteria;p__TM7
k__Bacteria;p__Nitrospirae
k__Bacteria;p__Armatimonadetes
k__Bacteria;p__Chlamydiae
k__Bacteria;p__WS3
k__Bacteria;p__OD1
k__Bacteria;p__WPS-2
k__Bacteria;p__AD3
k__Bacteria;p__Deferribacteres
k__Bacteria;p__GAL15
k__Bacteria;p__Chlorobi
k__Bacteria;p__Elusimicrobia
k__Bacteria;p__Fibrobacteres
