# Supplementary material for: The effects of genital myiasis on the diversity of the vaginal microbiota in female Bactrian camels
Source: BMC Vet Res. 2022 Mar 5;18:87. doi: 10.1186/s12917-022-03189-5 (PMC8897907; doi:10.1186/s12917-022-03189-5)
Supplement: Supplementary file 5 — Additional file 5. [file 12917_2022_3189_MOESM5_ESM.zip › MPL201709200_16s_yy/Treat1/B07_taxa_summary_group/taxa_summary_plots/charts/WqeQyjQiPz074miwdKPr5YLE92h7kI_legend.pdf]

k__Bacteria;p__Firmicutes;c__Clostridia
k__Bacteria;p__Fusobacteria;c__Fusobacteriia
k__Bacteria;p__Firmicutes;c__Bacilli
k__Bacteria;p__Proteobacteria;c__Alphaproteobacteria
k__Bacteria;p__Proteobacteria;c__Epsilonproteobacteria
k__Bacteria;p__Proteobacteria;c__Gammaproteobacteria
k__Bacteria;p__Actinobacteria;c__Actinobacteria
k__Bacteria;p__Proteobacteria;c__Betaproteobacteria
k__Bacteria;p__Bacteroidetes;c__Bacteroidia
k__Bacteria;p__Bacteroidetes;c__[Saprospirae]
No blast hit;Other;Other
k__Bacteria;p__Cyanobacteria;c__4C0d-2
k__Bacteria;p__Proteobacteria;c__Deltaproteobacteria
k__Bacteria;p__Actinobacteria;c__Coriobacteriia
k__Bacteria;p__Tenericutes;c__Mollicutes
k__Bacteria;p__Firmicutes;c__Erysipelotrichi
k__Bacteria;p__Bacteroidetes;c__Flavobacteriia
k__Bacteria;p__Verrucomicrobia;c__Verrucomicrobiae
k__Bacteria;p__GN02;c__3BR-5F
k__Bacteria;p__Cyanobacteria;c__Chloroplast
k__Bacteria;p__Lentisphaerae;c__[Lentisphaeria]
k__Bacteria;p__SR1;c__Unclassified_SR1
k__Bacteria;p__Chloroflexi;c__Anaerolineae
k__Bacteria;p__Verrucomicrobia;c__Verruco-5
k__Bacteria;p__Spirochaetes;c__Spirochaetes
k__Bacteria;p__[Thermi];c__Deinococci
k__Bacteria;p__Bacteroidetes;c__Cytophagia
k__Bacteria;p__Acidobacteria;c__Acidobacteria-6
k__Bacteria;p__Gemmatimonadetes;c__Gemmatimonadetes
k__Bacteria;p__Planctomycetes;c__Phycisphaerae
k__Bacteria;p__Gemmatimonadetes;c__Gemm-1
k__Bacteria;p__Acidobacteria;c__Solibacteres
k__Bacteria;p__TM7;c__TM7-3
k__Bacteria;p__Actinobacteria;c__Rubrobacteria
k__Bacteria;p__Actinobacteria;c__Acidimicrobiia
k__Bacteria;p__Nitrospirae;c__Nitrospira
k__Bacteria;p__Actinobacteria;c__Thermoleophilia
k__Bacteria;p__Bacteroidetes;c__Sphingobacteriia
k__Bacteria;p__Acidobacteria;c__Acidobacteriia
k__Bacteria;p__Planctomycetes;c__Planctomycetia
k__Bacteria;p__Chlamydiae;c__Chlamydiia
k__Bacteria;p__WS3;c__PRR-12
k__Bacteria;p__Acidobacteria;c__[Chloracidobacteria]
k__Bacteria;p__Tenericutes;c__RF3
k__Bacteria;p__Armatimonadetes;c__[Fimbriimonadia]
k__Bacteria;p__Chloroflexi;c__S085
k__Bacteria;p__Chloroflexi;c__Chloroflexi
k__Bacteria;p__OD1;c__ZB2
k__Bacteria;p__WPS-2;c__Unclassified_WPS-2
k__Bacteria;p__AD3;c__ABS-6
k__Bacteria;p__Chloroflexi;c__Thermomicrobia
k__Bacteria;p__Armatimonadetes;c__Chthonomonadetes
k__Bacteria;p__Tenericutes;c__CK-1C4-19
k__Bacteria;p__Deferribacteres;c__Deferribacteres
k__Bacteria;p__Acidobacteria;c__DA052
k__Bacteria;p__Chloroflexi;c__Ellin6529
k__Bacteria;p__GAL15;c__Unclassified_GAL15
k__Bacteria;p__Planctomycetes;c__C6
k__Bacteria;p__Chloroflexi;c__TK17
k__Bacteria;p__Chlorobi;c__SJA-28
k__Bacteria;p__Chloroflexi;c__Ktedonobacteria
k__Bacteria;p__Elusimicrobia;c__Elusimicrobia
k__Bacteria;p__Gemmatimonadetes;c__Gemm-5
k__Bacteria;p__Planctomycetes;c__OM190
k__Bacteria;p__Fibrobacteres;c__Fibrobacteria
k__Bacteria;p__Verrucomicrobia;c__Opitutae
k__Bacteria;p__Acidobacteria;c__RB25
k__Bacteria;p__TM7;c__TM7-1
k__Bacteria;p__Chloroflexi;c__Gitt-GS-136
k__Bacteria;p__Cyanobacteria;c__ML635J-21
k__Bacteria;p__Chloroflexi;c__TK10
k__Bacteria;p__Cyanobacteria;c__Oscillatoriophycideae
k__Bacteria;p__Acidobacteria;c__Sva0725
k__Bacteria;p__Acidobacteria;c__AT-s54
k__Bacteria;p__Armatimonadetes;c__0319-6E2
k__Bacteria;p__Acidobacteria;c__BPC102
k__Bacteria;p__OD1;c__ABY1
