# Supplementary material for: The effects of genital myiasis on the diversity of the vaginal microbiota in female Bactrian camels
Source: BMC Vet Res. 2022 Mar 5;18:87. doi: 10.1186/s12917-022-03189-5 (PMC8897907; doi:10.1186/s12917-022-03189-5)
Supplement: Supplementary file 5 — Additional file 5. [file 12917_2022_3189_MOESM5_ESM.zip › MPL201709200_16s_yy/Treat1/B07_taxa_summary_group/taxa_summary_plots/charts/uZyDXQ0O9HrAMAPkbI39GDAd1SUjpg_legend.pdf]

k__Bacteria;p__Firmicutes;c__Clostridia;o__Clostridiales;f__[Tissierellaceae]
k__Bacteria;p__Firmicutes;c__Bacilli;o__Lactobacillales;f__Aerococcaceae
k__Bacteria;p__Fusobacteria;c__Fusobacteriia;o__Fusobacteriales;f__Leptotrichiaceae
k__Bacteria;p__Proteobacteria;c__Epsilonproteobacteria;o__Campylobacterales;f__Campylobacteraceae
k__Bacteria;p__Fusobacteria;c__Fusobacteriia;o__Fusobacteriales;f__Fusobacteriaceae
k__Bacteria;p__Proteobacteria;c__Alphaproteobacteria;o__Rhizobiales;f__Brucellaceae
k__Bacteria;p__Bacteroidetes;c__Bacteroidia;o__Bacteroidales;f__Porphyromonadaceae
k__Bacteria;p__Firmicutes;c__Bacilli;o__Lactobacillales;f__Carnobacteriaceae
k__Bacteria;p__Proteobacteria;c__Betaproteobacteria;o__Burkholderiales;f__Alcaligenaceae
k__Bacteria;p__Actinobacteria;c__Actinobacteria;o__Actinomycetales;f__Corynebacteriaceae
k__Bacteria;p__Proteobacteria;c__Gammaproteobacteria;o__Xanthomonadales;f__Xanthomonadaceae
k__Bacteria;p__Bacteroidetes;c__[Saprospirae];o__[Saprospirales];f__Chitinophagaceae
k__Bacteria;p__Proteobacteria;c__Gammaproteobacteria;o__Pseudomonadales;f__Moraxellaceae
No blast hit;Other;Other;Other;Other
k__Bacteria;p__Actinobacteria;c__Actinobacteria;o__Actinomycetales;f__Actinomycetaceae
k__Bacteria;p__Proteobacteria;c__Gammaproteobacteria;o__Pseudomonadales;f__Pseudomonadaceae
k__Bacteria;p__Proteobacteria;c__Betaproteobacteria;o__Burkholderiales;f__Comamonadaceae
k__Bacteria;p__Firmicutes;c__Clostridia;o__Clostridiales;f__Ruminococcaceae
k__Bacteria;p__Firmicutes;c__Clostridia;o__Clostridiales;f__Clostridiaceae
k__Bacteria;p__Firmicutes;c__Clostridia;o__Clostridiales;f__Unclassified_Clostridiales
k__Bacteria;p__Proteobacteria;c__Alphaproteobacteria;o__Sphingomonadales;f__Sphingomonadaceae
k__Bacteria;p__Proteobacteria;c__Gammaproteobacteria;o__Enterobacteriales;f__Enterobacteriaceae
k__Bacteria;p__Firmicutes;c__Clostridia;o__Clostridiales;f__[Acidaminobacteraceae]
k__Bacteria;p__Firmicutes;c__Clostridia;o__Clostridiales;f__Lachnospiraceae
k__Bacteria;p__Actinobacteria;c__Actinobacteria;o__Actinomycetales;f__Micrococcaceae
k__Bacteria;p__Proteobacteria;c__Alphaproteobacteria;o__Rhizobiales;f__Methylobacteriaceae
k__Bacteria;p__Proteobacteria;c__Alphaproteobacteria;o__Caulobacterales;f__Caulobacteraceae
k__Bacteria;p__Proteobacteria;c__Betaproteobacteria;o__Burkholderiales;f__Oxalobacteraceae
k__Bacteria;p__Actinobacteria;c__Actinobacteria;o__Actinomycetales;f__Pseudonocardiaceae
k__Bacteria;p__Cyanobacteria;c__4C0d-2;o__MLE1-12;f__Unclassified_MLE1-12
k__Bacteria;p__Proteobacteria;c__Betaproteobacteria;o__Rhodocyclales;f__Rhodocyclaceae
k__Bacteria;p__Actinobacteria;c__Actinobacteria;o__Bifidobacteriales;f__Bifidobacteriaceae
k__Bacteria;p__Actinobacteria;c__Actinobacteria;o__Actinomycetales;f__Microbacteriaceae
k__Bacteria;p__Firmicutes;c__Bacilli;o__Lactobacillales;f__Streptococcaceae
k__Bacteria;p__Firmicutes;c__Clostridia;o__Clostridiales;f__Peptostreptococcaceae
k__Bacteria;p__Actinobacteria;c__Coriobacteriia;o__Coriobacteriales;f__Coriobacteriaceae
k__Bacteria;p__Proteobacteria;c__Betaproteobacteria;o__Neisseriales;f__Neisseriaceae
k__Bacteria;p__Firmicutes;c__Bacilli;o__Lactobacillales;f__Lactobacillaceae
k__Bacteria;p__Proteobacteria;c__Gammaproteobacteria;o__Pasteurellales;f__Pasteurellaceae
k__Bacteria;p__Proteobacteria;c__Alphaproteobacteria;o__Rhizobiales;f__Rhizobiaceae
k__Bacteria;p__Actinobacteria;c__Actinobacteria;o__Actinomycetales;f__Nocardiaceae
k__Bacteria;p__Bacteroidetes;c__Bacteroidia;o__Bacteroidales;f__Bacteroidaceae
k__Bacteria;p__Bacteroidetes;c__Bacteroidia;o__Bacteroidales;f__Rikenellaceae
k__Bacteria;p__Proteobacteria;c__Alphaproteobacteria;o__Rhizobiales;f__Unclassified_Rhizobiales
k__Bacteria;p__Bacteroidetes;c__Bacteroidia;o__Bacteroidales;f__Unclassified_Bacteroidales
k__Bacteria;p__Proteobacteria;c__Deltaproteobacteria;o__Desulfovibrionales;f__Desulfovibrionaceae
k__Bacteria;p__Firmicutes;c__Clostridia;o__Clostridiales;f__Peptococcaceae
k__Bacteria;p__Proteobacteria;c__Alphaproteobacteria;o__Rhizobiales;f__Phyllobacteriaceae
k__Bacteria;p__Firmicutes;c__Bacilli;o__Bacillales;f__Planococcaceae
k__Bacteria;p__Proteobacteria;c__Alphaproteobacteria;o__Rhizobiales;f__Bradyrhizobiaceae
k__Bacteria;p__Firmicutes;c__Bacilli;o__Bacillales;f__Bacillaceae
k__Bacteria;p__Firmicutes;c__Clostridia;o__Clostridiales;f__Veillonellaceae
k__Bacteria;p__Firmicutes;c__Erysipelotrichi;o__Erysipelotrichales;f__Erysipelotrichaceae
k__Bacteria;p__Tenericutes;c__Mollicutes;o__Acholeplasmatales;f__Acholeplasmataceae
k__Bacteria;p__Proteobacteria;c__Alphaproteobacteria;o__Sphingomonadales;f__Unclassified_Sphingomonadales
k__Bacteria;p__Verrucomicrobia;c__Verrucomicrobiae;o__Verrucomicrobiales;f__Verrucomicrobiaceae
k__Bacteria;p__Bacteroidetes;c__Bacteroidia;o__Bacteroidales;f__S24-7
k__Bacteria;p__GN02;c__3BR-5F;o__Unclassified_3BR-5F;f__Unclassified_3BR-5F
k__Bacteria;p__Proteobacteria;c__Gammaproteobacteria;o__Xanthomonadales;f__Sinobacteraceae
k__Bacteria;p__Actinobacteria;c__Actinobacteria;o__Actinomycetales;f__Streptomycetaceae
k__Bacteria;p__Cyanobacteria;c__Chloroplast;o__Streptophyta;f__Unclassified_Streptophyta
k__Bacteria;p__Proteobacteria;c__Gammaproteobacteria;o__Aeromonadales;f__Aeromonadaceae
k__Bacteria;p__Firmicutes;c__Clostridia;o__Clostridiales;f__Christensenellaceae
k__Bacteria;p__SR1;c__Unclassified_SR1;o__Unclassified_SR1;f__Unclassified_SR1
k__Bacteria;p__Proteobacteria;c__Deltaproteobacteria;o__Myxococcales;f__Myxococcaceae
k__Bacteria;p__Lentisphaerae;c__[Lentisphaeria];o__Victivallales;f__Victivallaceae
k__Bacteria;p__Actinobacteria;c__Actinobacteria;o__Actinomycetales;f__Propionibacteriaceae
k__Bacteria;p__Verrucomicrobia;c__Verruco-5;o__WCHB1-41;f__RFP12
k__Bacteria;p__Proteobacteria;c__Deltaproteobacteria;o__Myxococcales;f__Unclassified_Myxococcales
k__Bacteria;p__Actinobacteria;c__Actinobacteria;o__Actinomycetales;f__Nocardioidaceae
k__Bacteria;p__Bacteroidetes;c__Flavobacteriia;o__Flavobacteriales;f__Flavobacteriaceae
k__Bacteria;p__Actinobacteria;c__Actinobacteria;o__Actinomycetales;f__Brevibacteriaceae
k__Bacteria;p__Spirochaetes;c__Spirochaetes;o__Spirochaetales;f__Spirochaetaceae
k__Bacteria;p__Gemmatimonadetes;c__Gemm-1;o__Unclassified_Gemm-1;f__Unclassified_Gemm-1
k__Bacteria;p__Bacteroidetes;c__Cytophagia;o__Cytophagales;f__Cytophagaceae
k__Bacteria;p__[Thermi];c__Deinococci;o__Deinococcales;f__Deinococcaceae
k__Bacteria;p__Bacteroidetes;c__Flavobacteriia;o__Flavobacteriales;f__[Weeksellaceae]
k__Bacteria;p__Planctomycetes;c__Phycisphaerae;o__Phycisphaerales;f__Unclassified_Phycisphaerales
k__Bacteria;p__Proteobacteria;c__Alphaproteobacteria;o__Rhodospirillales;f__Rhodospirillaceae
k__Bacteria;p__Proteobacteria;c__Alphaproteobacteria;o__Rhodospirillales;f__Acetobacteraceae
k__Bacteria;p__Proteobacteria;c__Alphaproteobacteria;o__Rhizobiales;f__Hyphomicrobiaceae
k__Bacteria;p__Acidobacteria;c__Solibacteres;o__Solibacterales;f__Unclassified_Solibacterales
k__Bacteria;p__Firmicutes;c__Clostridia;o__Clostridiales;f__[Mogibacteriaceae]
k__Bacteria;p__Acidobacteria;c__Acidobacteria-6;o__iii1-15;f__Unclassified_iii1-15
k__Bacteria;p__Proteobacteria;c__Alphaproteobacteria;o__Rhodobacterales;f__Rhodobacteraceae
k__Bacteria;p__Proteobacteria;c__Betaproteobacteria;o__SC-I-84;f__Unclassified_SC-I-84
k__Bacteria;p__TM7;c__TM7-3;o__CW040;f__F16
k__Bacteria;p__Cyanobacteria;c__4C0d-2;o__YS2;f__Unclassified_YS2
k__Bacteria;p__Bacteroidetes;c__Bacteroidia;o__Bacteroidales;f__[Paraprevotellaceae]
k__Bacteria;p__Bacteroidetes;c__Bacteroidia;o__Bacteroidales;f__[Barnesiellaceae]
k__Bacteria;p__Actinobacteria;c__Actinobacteria;o__Actinomycetales;f__Unclassified_Actinomycetales
k__Bacteria;p__Chloroflexi;c__Anaerolineae;o__SBR1031;f__A4b
k__Bacteria;p__Actinobacteria;c__Rubrobacteria;o__Rubrobacterales;f__Rubrobacteraceae
k__Bacteria;p__Tenericutes;c__Mollicutes;o__Mycoplasmatales;f__Mycoplasmataceae
k__Bacteria;p__Firmicutes;c__Bacilli;o__Turicibacterales;f__Turicibacteraceae
k__Bacteria;p__Proteobacteria;c__Gammaproteobacteria;o__Cardiobacteriales;f__Cardiobacteriaceae
k__Bacteria;p__Actinobacteria;c__Actinobacteria;o__Actinomycetales;f__Micromonosporaceae
k__Bacteria;p__Actinobacteria;c__Actinobacteria;o__Actinomycetales;f__Mycobacteriaceae
k__Bacteria;p__Firmicutes;c__Bacilli;o__Lactobacillales;f__Enterococcaceae
k__Bacteria;p__Firmicutes;c__Bacilli;o__Bacillales;f__Staphylococcaceae
k__Bacteria;p__Gemmatimonadetes;c__Gemmatimonadetes;o__Unclassified_Gemmatimonadetes;f__Unclassified_Gemmatimonadetes
k__Bacteria;p__Bacteroidetes;c__Bacteroidia;o__Bacteroidales;f__RF16
k__Bacteria;p__Tenericutes;c__Mollicutes;o__RF39;f__Unclassified_RF39
k__Bacteria;p__Actinobacteria;c__Actinobacteria;o__Actinomycetales;f__Dietziaceae
k__Bacteria;p__Proteobacteria;c__Alphaproteobacteria;o__Unclassified_Alphaproteobacteria;f__Unclassified_Alphaproteobacteria
k__Bacteria;p__Acidobacteria;c__Acidobacteriia;o__Acidobacteriales;f__Koribacteraceae
k__Bacteria;p__Actinobacteria;c__Acidimicrobiia;o__Acidimicrobiales;f__Unclassified_Acidimicrobiales
k__Bacteria;p__Proteobacteria;c__Betaproteobacteria;o__MND1;f__Unclassified_MND1
k__Bacteria;p__Bacteroidetes;c__Bacteroidia;o__Bacteroidales;f__Prevotellaceae
k__Bacteria;p__Proteobacteria;c__Deltaproteobacteria;o__Syntrophobacterales;f__Syntrophobacteraceae
k__Bacteria;p__Lentisphaerae;c__[Lentisphaeria];o__Unclassified_[Lentisphaeria];f__Unclassified_[Lentisphaeria]
k__Bacteria;p__Actinobacteria;c__Actinobacteria;o__Actinomycetales;f__Geodermatophilaceae
k__Bacteria;p__Firmicutes;c__Bacilli;o__Gemellales;f__Gemellaceae
k__Bacteria;p__Chlamydiae;c__Chlamydiia;o__Chlamydiales;f__Rhabdochlamydiaceae
k__Bacteria;p__TM7;c__TM7-3;o__Unclassified_TM7-3;f__Unclassified_TM7-3
k__Bacteria;p__Gemmatimonadetes;c__Gemmatimonadetes;o__N1423WL;f__Unclassified_N1423WL
k__Bacteria;p__Firmicutes;c__Bacilli;o__Bacillales;f__Unclassified_Bacillales
k__Bacteria;p__Proteobacteria;c__Deltaproteobacteria;o__MIZ46;f__Unclassified_MIZ46
k__Bacteria;p__Proteobacteria;c__Deltaproteobacteria;o__Myxococcales;f__Haliangiaceae
k__Bacteria;p__Actinobacteria;c__Thermoleophilia;o__Gaiellales;f__Gaiellaceae
k__Bacteria;p__Proteobacteria;c__Deltaproteobacteria;o__Bdellovibrionales;f__Bdellovibrionaceae
k__Bacteria;p__Bacteroidetes;c__Sphingobacteriia;o__Sphingobacteriales;f__Unclassified_Sphingobacteriales
k__Bacteria;p__Proteobacteria;c__Alphaproteobacteria;o__Rhodobacterales;f__Hyphomonadaceae
k__Bacteria;p__Chloroflexi;c__Anaerolineae;o__H39;f__Unclassified_H39
k__Bacteria;p__Bacteroidetes;c__Sphingobacteriia;o__Sphingobacteriales;f__Sphingobacteriaceae
k__Bacteria;p__Proteobacteria;c__Betaproteobacteria;o__Unclassified_Betaproteobacteria;f__Unclassified_Betaproteobacteria
k__Bacteria;p__Tenericutes;c__RF3;o__ML615J-28;f__Unclassified_ML615J-28
k__Bacteria;p__Nitrospirae;c__Nitrospira;o__Nitrospirales;f__0319-6A21
k__Bacteria;p__Proteobacteria;c__Alphaproteobacteria;o__Sphingomonadales;f__Erythrobacteraceae
k__Bacteria;p__Gemmatimonadetes;c__Gemmatimonadetes;o__Gemmatimonadales;f__Ellin5301
k__Bacteria;p__Armatimonadetes;c__[Fimbriimonadia];o__[Fimbriimonadales];f__[Fimbriimonadaceae]
k__Bacteria;p__Actinobacteria;c__Actinobacteria;o__Actinomycetales;f__Cellulomonadaceae
k__Bacteria;p__Proteobacteria;c__Betaproteobacteria;o__Hydrogenophilales;f__Hydrogenophilaceae
k__Bacteria;p__Proteobacteria;c__Deltaproteobacteria;o__GMD14H09;f__Unclassified_GMD14H09
k__Bacteria;p__Actinobacteria;c__Actinobacteria;o__Actinomycetales;f__Dermabacteraceae
k__Bacteria;p__Chloroflexi;c__Anaerolineae;o__SBR1031;f__SHA-31
k__Bacteria;p__Chloroflexi;c__S085;o__Unclassified_S085;f__Unclassified_S085
k__Bacteria;p__OD1;c__ZB2;o__Unclassified_ZB2;f__Unclassified_ZB2
k__Bacteria;p__WS3;c__PRR-12;o__Sediment-1;f__Unclassified_Sediment-1
k__Bacteria;p__WPS-2;c__Unclassified_WPS-2;o__Unclassified_WPS-2;f__Unclassified_WPS-2
k__Bacteria;p__Gemmatimonadetes;c__Gemmatimonadetes;o__Gemmatimonadales;f__Unclassified_Gemmatimonadales
k__Bacteria;p__Acidobacteria;c__[Chloracidobacteria];o__RB41;f__Unclassified_RB41
k__Bacteria;p__Actinobacteria;c__Thermoleophilia;o__Solirubrobacterales;f__Unclassified_Solirubrobacterales
k__Bacteria;p__Proteobacteria;c__Gammaproteobacteria;o__Thiotrichales;f__Piscirickettsiaceae
k__Bacteria;p__Planctomycetes;c__Planctomycetia;o__Gemmatales;f__Isosphaeraceae
k__Bacteria;p__AD3;c__ABS-6;o__Unclassified_ABS-6;f__Unclassified_ABS-6
k__Bacteria;p__Bacteroidetes;c__Bacteroidia;o__Bacteroidales;f__BS11
k__Bacteria;p__Proteobacteria;c__Alphaproteobacteria;o__Rickettsiales;f__mitochondria
k__Bacteria;p__Proteobacteria;c__Alphaproteobacteria;o__Rhizobiales;f__Xanthobacteraceae
k__Bacteria;p__Planctomycetes;c__Planctomycetia;o__Gemmatales;f__Gemmataceae
k__Bacteria;p__Chloroflexi;c__Thermomicrobia;o__JG30-KF-CM45;f__Unclassified_JG30-KF-CM45
k__Bacteria;p__Chloroflexi;c__Chloroflexi;o__[Roseiflexales];f__[Kouleothrixaceae]
k__Bacteria;p__Verrucomicrobia;c__Verruco-5;o__WCHB1-41;f__Unclassified_WCHB1-41
k__Bacteria;p__[Thermi];c__Deinococci;o__Thermales;f__Thermaceae
k__Bacteria;p__Acidobacteria;c__Acidobacteria-6;o__CCU21;f__Unclassified_CCU21
k__Bacteria;p__Nitrospirae;c__Nitrospira;o__Nitrospirales;f__[Thermodesulfovibrionaceae]
k__Bacteria;p__Proteobacteria;c__Betaproteobacteria;o__Ellin6067;f__Unclassified_Ellin6067
k__Bacteria;p__Proteobacteria;c__Alphaproteobacteria;o__Rickettsiales;f__Unclassified_Rickettsiales
k__Bacteria;p__Proteobacteria;c__Betaproteobacteria;o__Burkholderiales;f__Burkholderiaceae
k__Bacteria;p__Proteobacteria;c__Deltaproteobacteria;o__NB1-j;f__NB1-i
k__Bacteria;p__Armatimonadetes;c__Chthonomonadetes;o__Chthonomonadales;f__Chthonomonadaceae
k__Bacteria;p__Proteobacteria;c__Alphaproteobacteria;o__BD7-3;f__Unclassified_BD7-3
k__Bacteria;p__Proteobacteria;c__Deltaproteobacteria;o__MBNT15;f__Unclassified_MBNT15
k__Bacteria;p__Chloroflexi;c__Anaerolineae;o__GCA004;f__Unclassified_GCA004
k__Bacteria;p__Tenericutes;c__CK-1C4-19;o__Unclassified_CK-1C4-19;f__Unclassified_CK-1C4-19
k__Bacteria;p__Spirochaetes;c__Spirochaetes;o__Sphaerochaetales;f__Sphaerochaetaceae
k__Bacteria;p__Deferribacteres;c__Deferribacteres;o__Deferribacterales;f__Deferribacteraceae
k__Bacteria;p__Gemmatimonadetes;c__Gemmatimonadetes;o__Ellin5290;f__Unclassified_Ellin5290
k__Bacteria;p__Actinobacteria;c__Actinobacteria;o__Actinomycetales;f__Actinosynnemataceae
k__Bacteria;p__Chloroflexi;c__Anaerolineae;o__SBR1031;f__oc28
k__Bacteria;p__Nitrospirae;c__Nitrospira;o__Nitrospirales;f__Nitrospiraceae
k__Bacteria;p__Cyanobacteria;c__Chloroplast;o__Stramenopiles;f__Unclassified_Stramenopiles
k__Bacteria;p__Proteobacteria;c__Betaproteobacteria;o__IS-44;f__Unclassified_IS-44
k__Bacteria;p__Chloroflexi;c__Anaerolineae;o__CFB-26;f__Unclassified_CFB-26
k__Bacteria;p__Actinobacteria;c__Acidimicrobiia;o__Acidimicrobiales;f__EB1017
k__Bacteria;p__Proteobacteria;c__Epsilonproteobacteria;o__Campylobacterales;f__Helicobacteraceae
k__Bacteria;p__Proteobacteria;c__Deltaproteobacteria;o__NB1-j;f__Unclassified_NB1-j
k__Bacteria;p__Acidobacteria;c__DA052;o__Ellin6513;f__Unclassified_Ellin6513
k__Bacteria;p__Actinobacteria;c__Actinobacteria;o__Actinomycetales;f__Intrasporangiaceae
k__Bacteria;p__Chloroflexi;c__Ellin6529;o__Unclassified_Ellin6529;f__Unclassified_Ellin6529
k__Bacteria;p__Proteobacteria;c__Gammaproteobacteria;o__Alteromonadales;f__Shewanellaceae
k__Bacteria;p__GAL15;c__Unclassified_GAL15;o__Unclassified_GAL15;f__Unclassified_GAL15
k__Bacteria;p__Acidobacteria;c__Solibacteres;o__Solibacterales;f__Solibacteraceae
k__Bacteria;p__Planctomycetes;c__C6;o__MVS-107;f__Unclassified_MVS-107
k__Bacteria;p__Lentisphaerae;c__[Lentisphaeria];o__Z20;f__R4-45B
k__Bacteria;p__Chloroflexi;c__TK17;o__Unclassified_TK17;f__Unclassified_TK17
k__Bacteria;p__Proteobacteria;c__Deltaproteobacteria;o__Myxococcales;f__0319-6G20
k__Bacteria;p__Chloroflexi;c__Anaerolineae;o__DRC31;f__Unclassified_DRC31
k__Bacteria;p__Chlorobi;c__SJA-28;o__Unclassified_SJA-28;f__Unclassified_SJA-28
k__Bacteria;p__Firmicutes;c__Clostridia;o__Clostridiales;f__Eubacteriaceae
k__Bacteria;p__Chloroflexi;c__Ktedonobacteria;o__JG30-KF-AS9;f__Unclassified_JG30-KF-AS9
k__Bacteria;p__Firmicutes;c__Bacilli;o__Lactobacillales;f__Unclassified_Lactobacillales
k__Bacteria;p__Proteobacteria;c__Betaproteobacteria;o__Burkholderiales;f__Unclassified_Burkholderiales
k__Bacteria;p__Gemmatimonadetes;c__Gemm-5;o__Unclassified_Gemm-5;f__Unclassified_Gemm-5
k__Bacteria;p__Bacteroidetes;c__Cytophagia;o__Cytophagales;f__Cyclobacteriaceae
k__Bacteria;p__Proteobacteria;c__Deltaproteobacteria;o__Desulfuromonadales;f__Geobacteraceae
k__Bacteria;p__Proteobacteria;c__Gammaproteobacteria;o__Alteromonadales;f__[Chromatiaceae]
k__Bacteria;p__Firmicutes;c__Clostridia;o__Thermoanaerobacterales;f__Thermoanaerobacteraceae
k__Bacteria;p__WS3;c__PRR-12;o__Sediment-1;f__PRR-10
k__Bacteria;p__Actinobacteria;c__Actinobacteria;o__Actinomycetales;f__Bogoriellaceae
k__Bacteria;p__Actinobacteria;c__Acidimicrobiia;o__Acidimicrobiales;f__C111
k__Bacteria;p__Firmicutes;c__Bacilli;o__Bacillales;f__[Exiguobacteraceae]
k__Bacteria;p__Bacteroidetes;c__Flavobacteriia;o__Flavobacteriales;f__Cryomorphaceae
k__Bacteria;p__Acidobacteria;c__Acidobacteria-6;o__iii1-15;f__mb2424
k__Bacteria;p__Actinobacteria;c__Actinobacteria;o__Actinomycetales;f__Yaniellaceae
k__Bacteria;p__Planctomycetes;c__Phycisphaerae;o__Phycisphaerales;f__Phycisphaeraceae
k__Bacteria;p__Chloroflexi;c__Anaerolineae;o__Caldilineales;f__Caldilineaceae
k__Bacteria;p__Proteobacteria;c__Deltaproteobacteria;o__Desulfobacterales;f__Desulfobacteraceae
k__Bacteria;p__Proteobacteria;c__Alphaproteobacteria;o__RF32;f__Unclassified_RF32
k__Bacteria;p__Proteobacteria;c__Alphaproteobacteria;o__Rhodospirillales;f__Unclassified_Rhodospirillales
k__Bacteria;p__Proteobacteria;c__Gammaproteobacteria;o__Vibrionales;f__Pseudoalteromonadaceae
k__Bacteria;p__Firmicutes;c__Clostridia;o__Clostridiales;f__Dehalobacteriaceae
k__Bacteria;p__Proteobacteria;c__Gammaproteobacteria;o__Unclassified_Gammaproteobacteria;f__Unclassified_Gammaproteobacteria
k__Bacteria;p__Proteobacteria;c__Deltaproteobacteria;o__Desulfobacterales;f__Desulfobulbaceae
k__Bacteria;p__Proteobacteria;c__Deltaproteobacteria;o__Myxococcales;f__OM27
k__Bacteria;p__Verrucomicrobia;c__Opitutae;o__[Cerasicoccales];f__[Cerasicoccaceae]
k__Bacteria;p__Proteobacteria;c__Betaproteobacteria;o__Methylophilales;f__Methylophilaceae
k__Bacteria;p__Firmicutes;c__Bacilli;o__Lactobacillales;f__Leuconostocaceae
k__Bacteria;p__Firmicutes;c__Bacilli;o__Bacillales;f__Thermoactinomycetaceae
k__Bacteria;p__Acidobacteria;c__Solibacteres;o__Solibacterales;f__PAUC26f
k__Bacteria;p__Proteobacteria;c__Gammaproteobacteria;o__[Marinicellales];f__[Marinicellaceae]
k__Bacteria;p__Proteobacteria;c__Gammaproteobacteria;o__Oceanospirillales;f__Halomonadaceae
k__Bacteria;p__Chloroflexi;c__Anaerolineae;o__S0208;f__Unclassified_S0208
k__Bacteria;p__Planctomycetes;c__OM190;o__agg27;f__Unclassified_agg27
k__Bacteria;p__Actinobacteria;c__Actinobacteria;o__Actinomycetales;f__Nakamurellaceae
k__Bacteria;p__Actinobacteria;c__Actinobacteria;o__Actinomycetales;f__Sporichthyaceae
k__Bacteria;p__Elusimicrobia;c__Elusimicrobia;o__Elusimicrobiales;f__Unclassified_Elusimicrobiales
k__Bacteria;p__Acidobacteria;c__RB25;o__Unclassified_RB25;f__Unclassified_RB25
k__Bacteria;p__TM7;c__TM7-1;o__Unclassified_TM7-1;f__Unclassified_TM7-1
k__Bacteria;p__Acidobacteria;c__[Chloracidobacteria];o__DS-100;f__Unclassified_DS-100
k__Bacteria;p__Bacteroidetes;c__Bacteroidia;o__Bacteroidales;f__[Odoribacteraceae]
k__Bacteria;p__Bacteroidetes;c__Cytophagia;o__Cytophagales;f__Unclassified_Cytophagales
k__Bacteria;p__Chloroflexi;c__Gitt-GS-136;o__Unclassified_Gitt-GS-136;f__Unclassified_Gitt-GS-136
k__Bacteria;p__Actinobacteria;c__Thermoleophilia;o__Solirubrobacterales;f__Solirubrobacteraceae
k__Bacteria;p__Proteobacteria;c__Alphaproteobacteria;o__Rhizobiales;f__Beijerinckiaceae
k__Bacteria;p__Proteobacteria;c__Gammaproteobacteria;o__Thiotrichales;f__Thiotrichaceae
k__Bacteria;p__Cyanobacteria;c__ML635J-21;o__Unclassified_ML635J-21;f__Unclassified_ML635J-21
k__Bacteria;p__Chloroflexi;c__TK10;o__B07_WMSP1;f__Unclassified_B07_WMSP1
k__Bacteria;p__Elusimicrobia;c__Elusimicrobia;o__Elusimicrobiales;f__Elusimicrobiaceae
k__Bacteria;p__Firmicutes;c__Bacilli;o__Bacillales;f__[Thermicanaceae]
k__Bacteria;p__Proteobacteria;c__Gammaproteobacteria;o__HOC36;f__Unclassified_HOC36
k__Bacteria;p__Acidobacteria;c__Solibacteres;o__Solibacterales;f__AKIW659
k__Bacteria;p__Acidobacteria;c__[Chloracidobacteria];o__RB41;f__Ellin6075
k__Bacteria;p__Cyanobacteria;c__Oscillatoriophycideae;o__Chroococcales;f__Gomphosphaeriaceae
k__Bacteria;p__Fibrobacteres;c__Fibrobacteria;o__Fibrobacterales;f__Fibrobacteraceae
k__Bacteria;p__Tenericutes;c__Mollicutes;o__Unclassified_Mollicutes;f__Unclassified_Mollicutes
k__Bacteria;p__Bacteroidetes;c__Bacteroidia;o__Bacteroidales;f__BA008
k__Bacteria;p__Chloroflexi;c__Chloroflexi;o__AKIW781;f__Unclassified_AKIW781
k__Bacteria;p__Acidobacteria;c__Sva0725;o__Sva0725;f__Unclassified_Sva0725
k__Bacteria;p__Chloroflexi;c__Anaerolineae;o__SBR1031;f__SJA-101
k__Bacteria;p__Gemmatimonadetes;c__Gemmatimonadetes;o__C114;f__Unclassified_C114
k__Bacteria;p__Acidobacteria;c__AT-s54;o__Unclassified_AT-s54;f__Unclassified_AT-s54
k__Bacteria;p__Acidobacteria;c__Acidobacteria-6;o__iii1-15;f__RB40
k__Bacteria;p__Actinobacteria;c__Thermoleophilia;o__Gaiellales;f__Unclassified_Gaiellales
k__Bacteria;p__Proteobacteria;c__Deltaproteobacteria;o__Myxococcales;f__Cystobacterineae
k__Bacteria;p__Proteobacteria;c__Alphaproteobacteria;o__Rhizobiales;f__Methylocystaceae
k__Bacteria;p__Proteobacteria;c__Deltaproteobacteria;o__BPC076;f__Unclassified_BPC076
k__Bacteria;p__Armatimonadetes;c__0319-6E2;o__Unclassified_0319-6E2;f__Unclassified_0319-6E2
k__Bacteria;p__Gemmatimonadetes;c__Gemmatimonadetes;o__KD8-87;f__Unclassified_KD8-87
k__Bacteria;p__Planctomycetes;c__OM190;o__CL500-15;f__Unclassified_CL500-15
k__Bacteria;p__Acidobacteria;c__BPC102;o__MVS-40;f__Unclassified_MVS-40
k__Bacteria;p__Proteobacteria;c__Gammaproteobacteria;o__Alteromonadales;f__Alteromonadaceae
k__Bacteria;p__Bacteroidetes;c__Bacteroidia;o__Bacteroidales;f__Marinilabiaceae
k__Bacteria;p__Chloroflexi;c__Anaerolineae;o__Anaerolineales;f__Anaerolinaceae
k__Bacteria;p__Fibrobacteres;c__Fibrobacteria;o__258ds10;f__Unclassified_258ds10
k__Bacteria;p__Bacteroidetes;c__[Saprospirae];o__[Saprospirales];f__Unclassified_[Saprospirales]
k__Bacteria;p__OD1;c__ABY1;o__Unclassified_ABY1;f__Unclassified_ABY1
k__Bacteria;p__Proteobacteria;c__Deltaproteobacteria;o__Spirobacillales;f__Unclassified_Spirobacillales
