# Supplementary material for: The effects of genital myiasis on the diversity of the vaginal microbiota in female Bactrian camels
Source: BMC Vet Res. 2022 Mar 5;18:87. doi: 10.1186/s12917-022-03189-5 (PMC8897907; doi:10.1186/s12917-022-03189-5)
Supplement: Supplementary file 5 — Additional file 5. [file 12917_2022_3189_MOESM5_ESM.zip › MPL201709200_16s_yy/Treat1/B09_graphlan/tree.pdf]

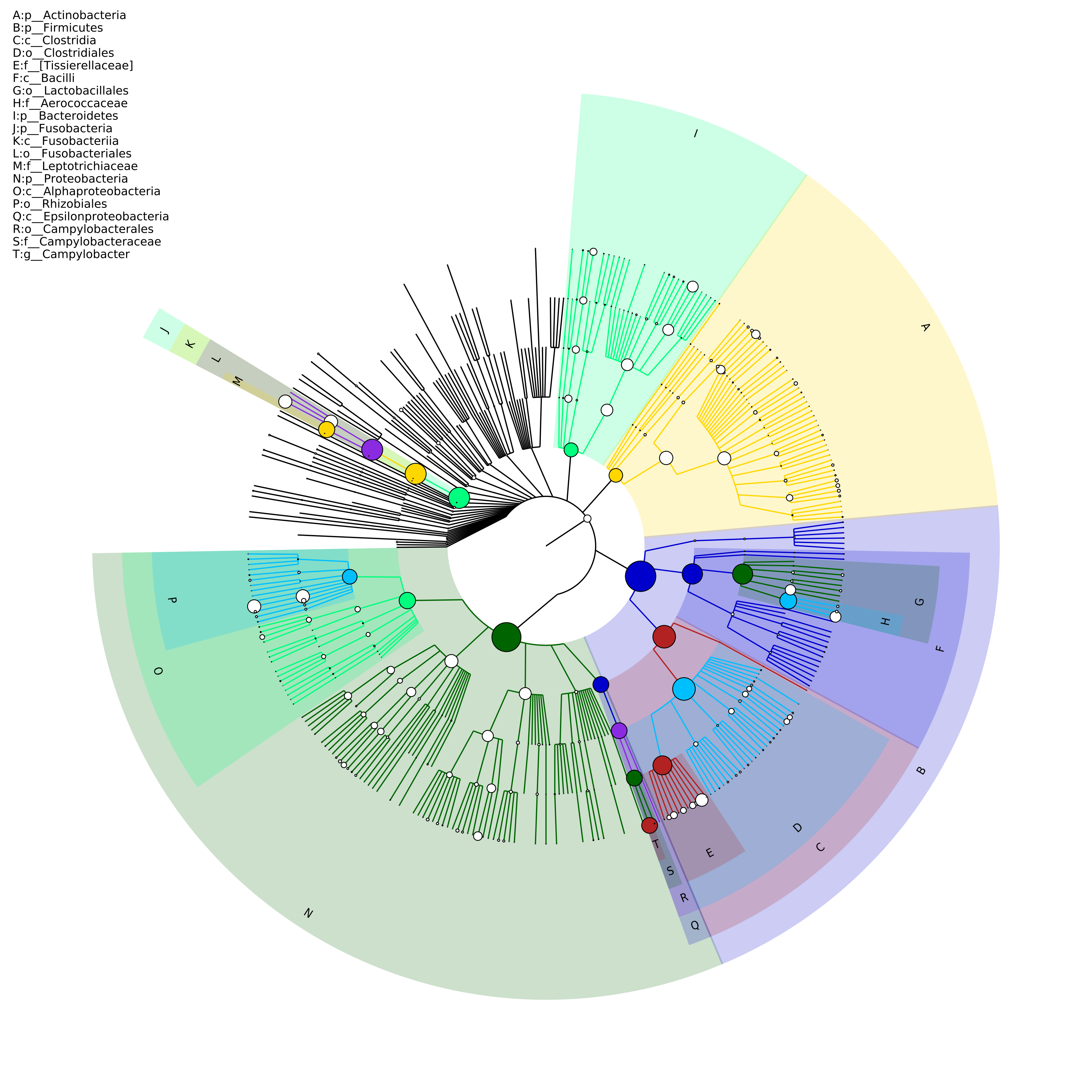
A:p__Actinobacteria
B:p__Firmicutes
C:c__Clostridia
D:o__Clostridiales
E:f__[Tissierellaceae]
F:c__Bacilli
G:o__Lactobacillales
H:f__Aerococcaceae
I:p__Bacteroidetes
J:p__Fusobacteria
K:c__Fusobacteriia
L:o__Fusobacteriales
M:f__Leptotrichiaceae
N:p__Proteobacteria
O:c__Alphaproteobacteria
P:o__Rhizobiales
Q:c__Epsilonproteobacteria
R:o__Campylobacterales
S:f__Campylobacteraceae
T:g__Campylobacter
I
A
J
K
L
M
P
O
G
H
F
B
D
C
E
T
S
R
Q
N
