# Supplementary material for: The effects of genital myiasis on the diversity of the vaginal microbiota in female Bactrian camels
Source: BMC Vet Res. 2022 Mar 5;18:87. doi: 10.1186/s12917-022-03189-5 (PMC8897907; doi:10.1186/s12917-022-03189-5)
Supplement: Supplementary file 5 — Additional file 5. [file 12917_2022_3189_MOESM5_ESM.zip › MPL201709200_16s_yy/Treat1/B10_krona/A01.html]

Javascript must be enabled to view this page.

members
magnitude
magnitudeUnassigned

A01

46250

46250

6

0

0

0

0

0

0

0

0

0

0

0

0

0

0

0

0

0

6

6

0

0

6

6

0

0

0

0

0

0

0

0

0

0

0

0

6886

2

0

0

0

0

0

0

0

0

0

0

0

0

2

0

0

0

0

2

2

0

0

0

0

0

0

0

0

0

0

0

0

0

0

0

0

0

0

0

0

0

0

0

0

0

0

0

0

0

0

0

0

0

0

0

791

0

0

0

0

0

0

0

720

0

0

578

578

0

0

45

0

0

0

0

20

25

0

0

97

0

4

0

0

0

0

45

48

0

0

0

0

0

0

0

0

0

0

0

0

0

0

0

0

71

71

0

34

0

0

31

6

0

0

0

3162

3162

3160

3

3157

2

2

0

1141

2

2

2

0

0

0

95

95

0

4

91

0

0

0

0

0

2

2

2

0

0

0

308

6

6

302

0

0

0

298

4

0

570

570

0

570

0

0

0

0

0

0

0

0

159

13

8

5

146

0

0

14

132

5

5

5

0

0

0

0

0

0

0

0

1790

0

0

0

0

0

4

2

2

0

0

0

2

0

2

0

119

119

2

2

2

1

112

4

4

2

0

0

2

0

0

0

0

0

1506

0

0

0

1284

1284

33

22

11

138

77

61

9

9

0

0

0

20

20

0

0

0

0

0

0

22

22

0

0

0

0

0

2

2

2

155

133

5

11

0

2

115

18

18

4

4

867

3

3

3

0

0

3

0

0

0

0

0

0

0

0

0

0

0

0

0

0

0

0

0

0

0

0

0

0

0

0

864

864

0

0

7

7

26

24

0

0

2

4

4

3

3

0

0

0

8

0

8

34

0

0

0

29

5

15

15

0

0

0

0

0

0

8

0

5

0

3

28

28

0

0

0

0

0

0

0

104

104

0

0

0

0

4

4

77

0

0

2

71

4

542

542

0

0

0

0

0

3

0

3

1

1

0

0

0

2

0

0

0

0

2

2

2

0

2

0

0

0

0

0

0

0

0

0

0

0

0

0

0

0

0

0

0

0

0

0

0

0

0

0

0

0

0

0

0

0

0

0

0

0

0

0

0

0

0

0

0

0

0

0

0

0

0

0

0

0

0

0

0

0

0

10

10

2

2

2

8

8

8

0

0

0

0

0

2

2

0

0

0

2

2

2

0

0

0

0

0

0

0

0

0

0

0

0

0

0

0

0

0

0

0

0

0

0

0

0

0

0

0

0

0

0

0

0

0

0

0

0

0

0

0

0

0

0

0

0

0

0

0

0

0

0

0

0

0

0

0

0

0

0

0

0

0

0

0

0

18

18

18

18

18

0

0

0

0

0

0

0

0

0

0

0

0

0

0

0

4253

3906

3906

0

0

0

0

16

0

11

3

2

0

0

0

0

0

0

0

9

9

3

3

0

0

2

0

0

2

0

3876

3876

0

0

0

0

0

0

0

0

340

340

340

0

340

0

0

0

0

0

0

0

0

0

0

0

2

2

0

0

2

2

0

0

0

0

0

0

5

5

0

0

5

3

2

0

0

0

0

0

15646

7910

7910

3

3

105

105

1

1

12

0

0

0

0

12

0

10

0

0

10

89

89

0

14

0

0

4

0

0

0

10

0

0

0

0

7662

1401

0

1497

170

126

4081

6

375

4

0

2

0

0

14

2

3

0

3

6

0

0

0

0

0

0

0

0

0

6

6

6

0

0

0

0

6

0

0

7730

2

2

2

7560

339

339

0

2

2

10

0

7

3

7131

43

2

6777

309

4

4

25

0

25

0

49

0

49

72

72

72

0

96

67

0

61

0

4

2

0

0

0

0

4

4

0

25

15

10

0

0

0

0

0

0

0

0

0

0

0

0

0

56

0

0

0

0

0

0

0

0

7

0

0

0

7

7

7

49

49

49

49

0

0

0

1

0

0

0

0

1

1

1

1

0

0

0

0

0

0

0

0

0

0

0

0

0

0

0

0

0

0

0

0

0

0

0

0

0

0

0

0

0

0

0

0

0

0

0

0

0

0

0

18501

18501

18501

8751

0

16

8735

9750

9750

0

0

0

0

0

0

0

2

0

0

0

0

0

0

0

0

0

0

0

0

0

0

0

0

0

0

0

0

0

0

0

0

0

0

0

0

0

0

0

0

0

0

0

0

2

2

0

0

2

2

0

0

0

0

0

0

0

0

0

0

0

0

0

0

0

0

0

0

0

0

0

0

0

0

0

0

0

0

0

0

0

0

0

0
