# Supplementary material for: The effects of genital myiasis on the diversity of the vaginal microbiota in female Bactrian camels
Source: BMC Vet Res. 2022 Mar 5;18:87. doi: 10.1186/s12917-022-03189-5 (PMC8897907; doi:10.1186/s12917-022-03189-5)
Supplement: Supplementary file 5 — Additional file 5. [file 12917_2022_3189_MOESM5_ESM.zip › MPL201709200_16s_yy/Treat1/B10_krona/A02.html]

Javascript must be enabled to view this page.

members
magnitude
magnitudeUnassigned

A02

42380

42380

85

85

0

0

0

0

0

0

0

0

0

85

85

40

45

0

0

0

0

0

0

0

0

0

0

0

0

0

0

0

0

0

0

0

0

0

0

0

0

0

0

0

0

0

0

0

0

0

0

0

0

0

0

7

0

0

0

0

0

0

0

0

0

0

0

0

0

0

0

0

0

7

7

0

0

7

7

2

0

0

0

0

0

0

0

0

0

0

0

0

0

0

0

0

0

0

0

0

0

0

0

0

0

0

0

0

0

0

0

0

0

0

0

0

0

0

0

0

0

0

0

0

0

0

0

2

0

0

0

2

2

2

0

0

0

0

0

0

0

0

0

0

0

0

0

0

0

0

0

0

0

0

0

0

0

0

0

0

0

0

0

0

0

3722

9

9

9

6

0

0

3

0

0

0

0

0

0

0

0

3713

3713

3

0

3

0

0

1221

55

36

5

1006

119

31

0

29

2

0

0

0

0

0

0

0

149

147

2

0

0

5

0

5

0

0

0

15

15

0

0

0

0

0

0

0

0

0

2285

2285

0

0

2

2

0

0

2

0

2

0

0

0

0

0

0

0

0

0

0

0

0

0

0

0

0

0

0

0

0

0

0

0

0

0

0

0

0

0

0

0

0

0

0

0

0

0

0

0

0

0

0

0

0

0

0

0

0

0

0

0

0

0

0

0

0

0

0

0

0

0

0

0

0

0

0

0

0

0

0

0

0

0

0

0

0

0

0

0

0

0

0

0

0

0

0

0

0

0

0

0

0

0

0

0

0

0

0

0

24687

15584

0

0

0

15584

12

8

2

0

0

0

0

2

0

0

2

2

14218

0

1081

8585

2590

9

597

0

779

0

577

0

0

0

0

0

0

0

0

19

5

0

0

0

14

0

0

0

0

294

294

44

2

42

0

346

346

0

0

0

649

647

0

2

0

0

9085

9076

0

0

0

5106

2

3154

0

1950

0

0

0

0

0

0

0

0

3967

0

3967

0

3

0

3

0

0

0

0

0

0

0

9

0

0

0

0

9

0

9

0

0

0

0

0

0

0

0

0

0

0

0

0

0

0

0

0

18

18

18

2

0

16

0

0

0

0

0

0

0

0

0

0

0

0

0

0

0

0

0

0

0

0

0

0

0

0

0

0

0

0

0

0

9431

9431

9431

5722

0

5

5717

3709

3709

12

12

0

0

0

12

12

0

12

0

0

0

0

0

0

0

0

0

0

0

0

0

0

0

0

0

0

0

0

0

0

0

0

0

0

0

0

0

0

0

0

0

0

0

0

0

0

0

0

0

0

0

0

0

8

8

0

0

0

8

8

8

0

0

0

0

0

0

0

0

0

0

0

0

0

0

0

0

0

0

0

0

0

0

0

0

0

0

0

0

0

0

3602

3034

3034

0

0

0

3034

3030

4

118

0

0

0

0

0

0

0

0

0

0

0

0

0

106

10

0

0

2

0

8

0

18

0

0

0

6

0

0

12

0

0

78

0

78

0

0

0

0

0

0

0

0

12

12

0

0

2

10

0

0

0

0

0

0

0

0

0

0

0

64

51

51

0

0

0

51

0

0

0

0

0

0

0

0

0

0

0

0

0

0

11

0

0

0

11

0

0

11

0

0

0

0

0

0

0

2

2

2

0

0

0

0

0

0

0

0

0

0

0

0

0

0

0

0

0

0

0

0

0

0

0

0

0

0

386

305

0

0

0

0

0

0

0

2

0

0

0

0

2

12

4

8

0

0

0

23

20

3

251

251

13

13

4

4

0

0

0

0

0

33

33

0

3

0

30

0

0

0

0

2

0

0

2

0

2

0

0

0

0

0

0

0

0

36

0

0

33

26

4

0

3

0

3

3

10

5

5

0

5

3

0

2

0

0

0

0

0

0

0

0

0

0

0

0

0

0

0

0

0

0

0

0

0

0

0

0

0

0

0

0

0

0

0

0

0

0

0

0

0

0

0

0

0

0

0

0

0

0

0

0

0

0

0

0

0

0

0

0

0

0

0

0

0

0

0

0

0

0

0

0

0

0

0

0

0

0

0

0

0

0

0

0

0

0

0

0

0

824

0

0

0

0

0

0

0

0

0

0

0

0

0

0

0

727

727

0

0

0

0

0

0

0

0

0

0

0

0

0

0

0

0

0

0

0

0

0

19

19

7

0

0

7

701

701

0

0

0

0

0

0

0

0

33

33

0

0

0

0

0

0

33

0

33

0

64

64

0

0

64

61

3

0
