# Supplementary material for: The effects of genital myiasis on the diversity of the vaginal microbiota in female Bactrian camels
Source: BMC Vet Res. 2022 Mar 5;18:87. doi: 10.1186/s12917-022-03189-5 (PMC8897907; doi:10.1186/s12917-022-03189-5)
Supplement: Supplementary file 5 — Additional file 5. [file 12917_2022_3189_MOESM5_ESM.zip › MPL201709200_16s_yy/Treat1/B10_krona/A03.html]

Javascript must be enabled to view this page.

members
magnitude
magnitudeUnassigned

A03

46201

46201

9

9

9

9

9

0

0

0

0

0

13

0

0

0

0

0

0

0

0

0

0

0

0

0

0

0

0

0

0

0

0

0

13

13

13

13

0

0

0

0

6

6

6

0

0

6

6

68

68

0

0

0

68

68

68

0

74

74

67

67

67

7

7

7

41

41

41

0

0

41

41

85

0

0

0

0

47

47

47

47

38

38

38

38

0

0

0

0

0

0

0

0

0

0

21809

130

0

0

0

14

14

14

0

0

0

93

0

0

31

31

62

0

62

0

0

0

0

0

0

0

0

0

0

0

0

9

9

9

0

0

0

0

0

0

0

0

0

0

0

0

0

0

0

0

0

0

0

14

14

14

11362

0

0

0

120

65

0

0

65

0

0

55

50

5

680

680

3

0

21

0

656

0

0

0

56

0

0

56

0

0

44

12

1142

0

0

961

45

18

9

861

28

181

181

0

0

0

6

0

0

6

6

9358

44

0

28

0

8

8

168

168

0

0

0

245

245

836

178

658

0

0

81

20

61

0

0

0

7827

7827

157

157

749

749

0

0

0

749

25

724

4981

0

0

0

0

0

0

22

22

3

19

0

0

0

0

0

0

0

0

0

0

0

0

4598

12

12

1064

0

23

428

13

0

582

0

0

18

2843

0

0

2843

679

1

0

130

539

0

9

0

0

358

358

0

2

144

208

4

0

3

3

3

4587

81

81

78

0

3

2143

1827

7

134

1686

0

316

118

198

1883

91

91

1792

1752

19

21

0

0

0

0

0

0

15

15

15

0

0

0

0

0

6

6

6

0

0

0

27

27

27

234

234

0

0

0

0

234

24

0

0

0

0

24

24

173

173

173

1

1

1

15

15

15

15

15

0

0

0

0

0

0

0

0

0

0

0

0

0

0

0

0

30

30

30

23

23

0

0

0

7

7

162

2

0

0

0

2

2

2

0

0

56

0

0

0

56

39

39

0

0

17

17

32

32

32

0

32

0

0

0

0

0

0

0

0

0

0

0

0

59

59

0

0

0

0

59

59

0

0

13

13

13

13

0

0

0

0

3195

839

839

0

0

0

0

0

462

462

0

0

17

17

0

0

23

13

10

0

0

0

0

0

0

0

0

238

238

0

0

0

0

41

41

58

50

0

8

0

0

0

0

0

0

0

0

0

9

9

0

0

0

9

9

39

39

0

0

0

0

0

0

39

39

0

0

2308

2308

2305

2305

0

0

3

3

58

0

0

0

0

0

0

0

28

28

0

0

28

28

0

0

0

0

30

30

0

0

30

30

29

29

29

29

29

0

0

0

0

0

0

0

0

0

0

0

0

0

47

45

45

45

45

0

0

0

0

2

2

0

0

2

2

191

0

0

0

0

3

3

3

3

75

12

12

12

0

0

0

0

0

0

0

0

0

21

21

21

42

14

14

0

0

28

28

0

0

0

0

0

0

0

0

37

37

37

37

0

0

0

0

76

17

17

17

59

59

59

0

0

0

0

0

0

0

0

0

0

0

0

2015

22

22

22

10

0

0

12

1843

13

13

13

1830

0

0

0

369

369

267

0

0

267

3

3

108

108

119

0

104

14

1

0

8

8

18

0

8

0

10

63

0

4

59

29

0

29

0

0

0

0

0

0

0

0

396

58

0

273

49

16

20

20

95

95

32

0

16

16

0

0

0

0

0

0

0

0

0

0

0

33

33

270

15

3

241

11

61

61

61

61

77

77

77

77

0

0

0

0

0

0

0

12

12

12

12

0

0

0

0

65

43

43

43

43

0

0

0

22

22

22

22

45

45

45

45

45

14831

8490

8264

0

0

7388

85

34

4505

2764

11

11

0

24

24

0

31

0

31

0

806

4

802

0

4

4

207

20

20

103

96

0

3

0

4

0

0

0

0

3

3

47

0

47

0

0

0

0

34

34

0

0

0

0

19

19

19

0

51

51

51

17

0

21

3

0

0

10

6290

6290

307

293

13

1

342

211

0

82

6

43

30

0

30

0

333

333

4112

306

0

288

228

0

16

0

2722

367

185

0

40

40

2

2

792

792

5

0

5

2

0

0

2

0

0

0

44

44

278

6

32

10

0

158

54

0

0

18

3

3

0

0

0

585

18

18

18

18

157

142

142

142

15

15

15

410

410

410

410

0

0

0

0

0

0

0

0

0

0

0

0

13

13

13

13

13

0

0

0

0

2787

2787

2787

1442

1442

1345

1022

311

12

7

7

0

0

0

7

7

7

21

21

21

21

21
