# Supplementary material for: The effects of genital myiasis on the diversity of the vaginal microbiota in female Bactrian camels
Source: BMC Vet Res. 2022 Mar 5;18:87. doi: 10.1186/s12917-022-03189-5 (PMC8897907; doi:10.1186/s12917-022-03189-5)
Supplement: Supplementary file 5 — Additional file 5. [file 12917_2022_3189_MOESM5_ESM.zip › MPL201709200_16s_yy/Treat1/B10_krona/A04.html]

Javascript must be enabled to view this page.

members
magnitude
magnitudeUnassigned

A04

46101

46101

32

32

0

0

0

32

32

32

0

14028

4003

0

0

0

4003

1108

14

0

1094

201

129

5

55

0

12

148

0

0

0

84

21

31

10

2

0

827

827

4

4

0

0

0

0

712

712

0

0

0

0

0

978

0

63

0

156

30

0

12

31

586

100

0

25

25

0

0

0

0

0

0

0

0

0

9986

9832

1284

0

1272

12

72

72

0

0

0

0

2

0

2

0

0

8474

24

6537

0

1913

0

0

0

0

0

0

16

16

3

13

138

22

0

22

0

0

58

58

0

0

52

2

11

0

39

0

0

0

6

0

3

3

0

0

0

39

39

39

10

0

16

0

0

4

9

0

0

0

0

0

0

0

0

77

0

0

0

0

0

0

0

49

49

49

49

0

0

0

0

0

0

28

28

23

23

5

5

23

23

23

23

23

7

0

0

0

0

0

0

0

0

7

0

0

0

0

0

0

0

0

0

0

7

7

7

35

0

0

0

0

35

27

27

27

8

8

8

20314

2898

12

12

12

0

0

0

0

0

0

0

0

0

1

1

0

1

0

0

0

0

713

713

689

0

0

24

0

0

0

0

0

0

0

0

0

0

0

0

0

0

0

0

1200

82

82

1118

4

3

0

2

0

1109

972

803

0

41

762

0

169

126

43

0

0

0

3682

0

0

0

0

0

0

0

0

0

0

0

0

72

72

16

56

0

0

0

271

271

0

0

4

139

0

128

0

0

0

0

0

0

3339

769

0

0

326

22

2

2

0

417

0

18

18

0

0

2271

0

0

2271

281

115

147

19

0

0

0

121

0

0

0

96

0

0

0

0

17

17

10

10

61

0

61

8

8

0

0

0

0

0

0

0

0

0

13

13

13

0

0

0

0

0

0

0

0

0

0

0

0

0

0

2

2

2

10

10

0

0

10

0

0

0

11854

10

10

10

67

43

0

43

24

24

0

0

0

0

46

0

0

46

19

16

6

5

9954

753

583

170

0

0

0

0

200

200

8584

8584

16

16

0

118

118

151

0

151

66

25

41

66

0

0

61

0

5

0

0

0

1161

0

0

360

360

801

44

0

15

0

742

53

53

53

0

0

0

0

0

563

563

11

537

0

14

1

1759

1759

2

2

0

1757

1737

20

19

0

0

0

0

19

0

0

0

19

19

19

0

0

0

0

0

0

0

0

0

0

0

0

0

0

0

0

0

0

0

0

0

0

0

0

0

0

0

0

0

0

5293

49

49

49

49

0

0

0

0

0

0

0

9

9

9

9

5215

0

0

0

5215

5

5

0

0

269

0

0

123

0

146

0

0

0

0

0

0

0

0

0

7

7

0

0

0

0

0

0

0

232

232

176

176

68

68

0

0

123

7

0

17

65

34

0

0

0

20

20

6

6

0

0

0

0

148

148

0

0

410

0

0

410

1492

1492

2171

29

2142

0

0

0

0

0

88

88

20

20

20

20

0

0

0

0

0

0

0

0

0

0

0

20

12

12

12

12

8

8

8

8

0

0

0

0

554

78

17

17

17

61

61

61

461

461

461

461

0

0

0

0

0

0

0

15

15

15

15

21

21

21

21

21

2

2

2

2

2

76

0

0

0

0

61

61

61

61

0

0

0

0

0

0

0

0

0

0

0

0

0

0

0

0

0

0

0

0

0

0

0

0

0

0

0

0

15

15

15

15

0

0

0

0

0

0

0

0

0

0

0

0

0

0

0

0

0

0

0

0

0

0

0

0

0

0

2769

34

34

16

0

2

14

18

18

0

0

0

0

0

0

0

0

0

0

0

0

427

427

0

0

0

0

0

0

0

76

56

11

0

9

0

0

19

19

10

0

0

0

10

0

0

21

21

0

0

13

0

13

0

288

288

0

0

0

0

48

48

5

5

0

43

43

2260

2260

8

8

2252

2252

0

0

0

0

0

0

0

0

0

0

0

0

21

0

0

0

0

0

0

21

21

21

21

0

0

0

0

0

0

0

0

0

0

0

0

0

0

0

0

34

34

0

0

0

34

34

34

2546

2546

2546

1515

1515

1031

43

0

988

184

184

184

184

184

5

5

5

5

5

0

0

0

0

0

32

0

0

0

0

0

0

0

0

0

0

0

0

32

0

0

0

0

0

0

0

0

0

0

0

0

32

0

0

0

0

32

32

0

0

0

0

0

0

0

0

0

0

0

0

0

0

0

0

0

0

0

0

0

0

0

0

0

0

0

0

0

0

0

0

0

0

9

0

0

0

0

9

9

9

9
