# Supplementary material for: The effects of genital myiasis on the diversity of the vaginal microbiota in female Bactrian camels
Source: BMC Vet Res. 2022 Mar 5;18:87. doi: 10.1186/s12917-022-03189-5 (PMC8897907; doi:10.1186/s12917-022-03189-5)
Supplement: Supplementary file 5 — Additional file 5. [file 12917_2022_3189_MOESM5_ESM.zip › MPL201709200_16s_yy/Treat1/B10_krona/A05.html]

Javascript must be enabled to view this page.

members
magnitude
magnitudeUnassigned

A05

46121

46121

2

0

0

0

0

0

0

0

0

0

0

0

0

0

0

0

0

0

0

0

0

0

0

0

0

0

0

0

0

0

0

0

0

0

0

0

0

0

0

0

0

0

2

2

0

0

0

0

0

0

2

2

0

0

0

0

11

11

11

2

2

9

6

0

3

0

0

0

0

0

0

0

0

0

0

0

0

0

0

0

0

0

0

0

0

0

0

0

0

0

0

0

0

0

0

0

0

0

0

0

0

0

0

0

0

0

0

0

0

0

0

0

0

0

0

0

0

0

0

0

0

0

0

0

0

0

0

0

0

0

0

0

0

0

0

0

0

0

0

0

0

0

0

0

0

0

0

0

0

0

0

0

0

0

0

0

0

0

0

0

0

0

0

0

0

0

0

0

0

0

0

0

0

0

0

0

0

0

0

0

0

0

0

0

0

0

0

0

0

0

2

0

0

0

0

2

2

2

2

0

0

0

0

0

0

0

0

0

0

0

0

0

0

0

6

6

6

6

6

918

0

0

0

0

0

0

0

0

148

148

148

146

0

2

0

0

765

765

0

0

0

0

0

0

0

0

0

0

0

0

2

0

0

2

0

0

0

0

0

763

0

763

0

0

0

0

0

0

0

0

0

0

0

0

0

5

5

0

0

5

0

5

0

0

0

0

0

0

0

0

0

0

0

0

0

0

0

0

0

38

0

0

0

0

11

11

11

11

0

0

0

0

0

0

0

27

27

27

27

0

0

0

3403

386

0

0

0

23

23

0

0

16

0

7

0

0

0

0

0

0

0

0

0

0

0

0

0

0

0

0

0

363

0

0

44

0

15

0

27

0

2

0

0

0

156

156

0

0

0

0

163

138

0

12

13

0

0

0

0

0

0

0

0

1431

0

0

0

0

0

0

0

0

5

5

0

0

2

3

0

0

0

0

0

0

0

0

0

0

0

0

0

0

1241

1233

1233

0

0

0

8

6

2

0

0

0

0

0

0

0

0

0

181

5

5

176

176

0

0

0

0

0

4

4

4

0

0

0

0

0

7

0

0

0

0

0

0

0

0

0

7

0

0

0

0

7

7

0

0

0

0

0

0

0

0

0

0

0

0

0

0

0

0

0

0

0

0

0

0

0

0

0

0

0

0

0

0

0

0

0

0

0

0

0

0

16

16

16

2

14

0

0

0

1563

49

6

6

0

0

43

0

0

38

3

2

1415

13

13

2

0

2

389

389

204

193

11

0

0

8

0

8

22

0

0

0

18

4

768

12

756

0

0

9

9

0

0

0

0

0

0

0

0

0

0

0

63

0

0

63

0

55

8

0

0

0

0

2

0

0

0

0

0

2

0

0

2

34

34

0

0

4

28

2

0

0

0

0

0

0

0

0

0

0

0

0

0

3048

8

8

8

0

8

0

0

0

0

0

0

0

0

0

0

3038

25

25

25

3013

2

0

2

0

0

0

0

0

0

0

2

0

0

0

2

607

429

0

10

0

168

2

2

0

0

0

1060

4

82

0

974

0

0

0

0

1228

1228

0

0

31

0

0

31

34

0

0

12

0

22

2

2

2

2

36

36

0

0

0

0

0

0

0

0

0

0

0

4

4

3

3

0

0

0

0

0

0

0

2

0

0

0

0

0

2

0

0

2

2

0

0

0

0

0

0

0

0

0

0

0

0

0

0

0

0

0

0

0

0

0

0

0

0

0

0

0

0

0

0

0

0

0

0

0

0

0

0

4

4

4

4

4

0

0

0

0

0

0

0

0

0

0

0

0

0

0

0

0

0

0

0

0

0

0

0

0

0

0

0

0

0

0

0

0

5

5

0

0

0

5

5

5

0

0

0

0

0

0

0

0

0

0

0

0

0

0

0

0

0

0

0

0

0

0

0

0

0

0

0

0

0

0

0

38684

0

0

0

0

0

0

0

0

0

0

22122

0

0

0

0

22096

0

0

0

0

9696

1005

0

19

8672

0

0

0

12366

0

12366

0

26

0

26

8

0

0

8

3

3

3

23

3

0

0

0

0

0

0

3

0

0

20

0

0

0

20

0

0

0

0

0

0

0

0

0

16562

16562

0

0

0

0

0

0

0

16359

18

0

1317

1534

0

1368

0

10986

1003

0

133

0

0

0

0

0

0

0

0

0

0

0

0

200

8

192

0

0

0

0

3

0

0

0

0

3

0

0

0

0

0

0

0

0

0

0

0

0

0

0

0

0

0

0

0

0

0

0

0

0
