# Supplementary material for: The effects of genital myiasis on the diversity of the vaginal microbiota in female Bactrian camels
Source: BMC Vet Res. 2022 Mar 5;18:87. doi: 10.1186/s12917-022-03189-5 (PMC8897907; doi:10.1186/s12917-022-03189-5)
Supplement: Supplementary file 5 — Additional file 5. [file 12917_2022_3189_MOESM5_ESM.zip › MPL201709200_16s_yy/Treat1/B10_krona/A06.html]

Javascript must be enabled to view this page.

members
magnitude
magnitudeUnassigned

A06

46065

46065

0

0

0

0

0

0

0

0

0

0

0

0

8

0

0

0

0

8

8

8

8

0

0

0

0

0

0

0

0

0

0

0

0

0

0

12430

4940

4940

4933

4933

0

7

7

0

3846

3160

51

51

11

0

3

0

8

0

0

0

2506

2506

387

314

73

79

79

47

21

26

79

0

79

0

0

0

0

0

8

0

0

8

8

7

7

7

4

4

4

399

0

0

18

18

381

358

9

0

7

7

227

227

2

5

210

10

0

34

16

16

0

18

1

17

0

0

0

0

0

0

7

0

0

7

0

0

0

7

1464

684

15

15

669

0

669

0

0

0

0

0

0

0

6

6

6

0

0

0

0

0

0

0

0

0

7

7

6

1

0

20

20

0

0

20

0

0

0

0

0

716

493

13

8

14

458

223

171

52

31

13

13

0

0

18

18

0

0

0

0

0

0

0

0

29

0

0

0

11

11

11

18

0

0

0

0

0

0

3

3

15

15

0

0

0

0

0

0

0

0

0

0

0

0

0

0

0

0

0

0

0

0

0

0

0

0

0

0

0

0

0

0

0

0

0

0

0

0

0

2151

4

4

4

0

0

0

145

145

72

56

15

2

0

0

0

0

0

0

0

0

0

0

0

0

0

0

5

5

0

5

0

0

0

1997

436

0

218

0

24

0

194

0

0

0

0

0

252

134

0

108

4

0

6

1309

10

0

1299

0

0

4

4

4

4

4

0

0

0

0

0

0

0

0

0

0

0

0

0

0

0

0

0

0

0

0

0

0

0

0

0

40

40

40

40

40

13187

13187

13187

6509

0

48

6461

6678

6678

0

0

0

0

0

20

0

0

0

0

0

0

0

0

0

0

0

0

0

0

0

0

0

0

0

0

0

0

0

0

0

0

0

0

0

0

12

0

0

0

0

0

0

0

12

12

12

8

8

8

8

0

0

0

0

0

0

0

0

0

0

257

5

5

5

5

0

0

0

0

32

0

0

0

32

32

32

220

220

220

220

0

0

0

2083

2

2

2

0

2

0

0

2069

21

21

21

2048

0

0

11

11

215

19

0

196

0

0

0

0

159

0

159

0

0

0

0

0

0

0

0

17

17

0

0

0

38

38

0

0

82

19

2

0

61

0

2

0

0

0

2

0

0

0

882

672

196

0

2

12

0

0

0

0

0

294

294

0

0

0

0

0

27

14

6

7

316

316

5

0

5

12

0

0

0

0

0

12

0

0

12

12

0

0

0

0

0

0

0

0

0

0

0

0

0

0

0

0

0

0

0

0

0

0

12560

4639

0

0

0

4639

49

0

0

0

0

49

0

0

0

225

9

0

168

41

7

560

560

1891

212

425

2

217

178

64

0

480

0

299

14

0

0

0

0

8

8

0

0

0

0

0

1389

14

1375

0

0

0

38

2

0

2

13

19

2

0

0

0

479

479

7888

7800

5

0

5

0

0

2

2

145

0

0

145

0

0

0

7625

1816

0

5779

30

23

0

6

17

0

0

0

0

0

0

0

88

0

0

5

0

5

0

0

63

0

44

0

0

11

1

7

0

0

0

0

20

5

0

0

15

33

33

33

0

26

0

0

5

0

2

2

2

2

2

2

0

0

0

0

15

15

0

0

0

15

15

15

0

0

0

0

0

4911

5

5

0

0

0

0

5

5

0

0

0

0

0

0

0

781

781

0

0

781

781

0

0

4125

4125

44

2

0

32

10

3867

0

0

3867

0

0

12

12

33

33

6

0

6

0

0

0

0

0

134

134

2

2

13

13

0

0

0

14

0

2

10

2

0

0

0

0

0

0

0

0

0

0

0

0

0

0

0

0

0

9

0

0

0

0

0

0

9

9

9

9

0

0

0

0

122

122

69

69

69

53

53

0

53

0

0

0

0

0

16

0

0

0

0

11

11

11

11

5

5

5

5

0

0

0

0

0

0

0

40

40

0

0

0

40

40

40

14

0

0

0

0

0

0

0

0

0

0

0

0

14

0

0

0

0

0

0

14

14

14

0

0

0

0

0

0

0

0

0

0

0

0

0

0

0

0

0

0

0

0

0

0

0

0

0

0

0

0

0

0

0

0

0

0

0

0

0

0

0

0

0

0

0

0

331

324

324

324

324

0

0

0

0

0

0

0

0

0

0

0

0

0

0

7

7

7

7

0

0

0

0

0

10

0

0

0

0

0

0

0

0

0

0

0

0

0

0

0

0

0

10

10

0

0

10

10

6

6

6

6

6
