# Supplementary material for: The effects of genital myiasis on the diversity of the vaginal microbiota in female Bactrian camels
Source: BMC Vet Res. 2022 Mar 5;18:87. doi: 10.1186/s12917-022-03189-5 (PMC8897907; doi:10.1186/s12917-022-03189-5)
Supplement: Supplementary file 5 — Additional file 5. [file 12917_2022_3189_MOESM5_ESM.zip › MPL201709200_16s_yy/Treat1/B10_krona/A07.html]

Javascript must be enabled to view this page.

members
magnitude
magnitudeUnassigned

A07

46310

46310

0

0

0

0

0

0

0

0

0

0

0

0

0

0

0

0

0

0

0

0

0

0

0

0

0

0

0

0

0

0

0

0

0

0

0

0

0

0

0

0

0

0

0

0

0

0

0

0

0

0

0

0

0

0

0

0

0

0

0

0

0

0

0

0

0

0

0

0

0

0

0

0

0

0

0

0

0

0

0

0

0

0

0

0

0

0

0

0

0

0

0

0

0

0

0

0

0

0

0

0

0

0

0

0

0

0

0

0

0

0

0

0

0

0

0

0

2

2

0

0

0

2

2

2

0

0

0

0

0

0

0

0

0

0

0

0

0

0

0

0

0

0

0

0

0

0

0

0

0

0

0

0

0

14

14

0

0

0

14

14

14

2

2

2

2

2

0

0

0

0

4

4

0

0

0

0

0

0

0

0

0

0

4

4

4

0

0

0

0

0

0

0

0

18

18

18

18

18

3537

4

4

0

0

0

4

4

3108

3108

9

9

0

0

0

0

0

0

0

0

0

2

2

0

0

3086

0

3086

0

10

5

5

0

0

0

0

1

0

1

0

0

0

0

0

0

0

0

0

0

0

0

0

0

0

0

0

0

0

0

425

425

0

0

425

337

88

0

0

0

0

0

0

0

0

0

9139

1022

0

0

0

3

3

0

0

3

0

0

0

0

0

0

0

0

0

0

0

718

483

439

44

235

0

0

209

26

0

0

0

0

0

0

266

14

14

252

0

17

0

0

0

235

0

0

0

30

30

23

0

0

0

7

0

0

0

0

0

0

0

5

5

5

21

0

0

0

0

0

10

10

0

2

8

6

0

0

0

0

5

5

1

1

0

0

0

0

0

0

0

0

0

0

0

0

0

0

0

0

2

2

2

3

3

3

0

0

0

0

0

0

0

0

0

0

0

0

0

2010

1654

30

30

0

1279

1279

0

0

0

0

0

0

124

4

120

0

0

0

0

0

37

37

19

19

2

2

163

115

48

15

8

0

8

0

7

7

0

0

0

0

0

0

0

0

3

3

3

171

0

0

19

19

152

0

9

2

137

4

163

163

0

117

39

3

4

0

0

0

0

0

0

4

4

0

2

2

0

0

0

5043

5043

5043

0

5043

0

0

0

1043

0

0

0

0

2

2

2

0

0

0

0

0

0

0

0

0

91

91

0

0

29

3

59

0

0

0

0

0

0

0

0

0

0

950

488

0

0

488

0

0

375

0

201

0

2

3

5

0

112

52

83

0

47

36

0

0

0

4

4

3432

3432

3432

2006

8

1998

0

1426

1426

0

0

0

0

0

19

18

18

18

18

1

1

0

0

1

1

0

0

0

0

5701

0

0

0

0

5701

5701

3397

3397

21

9

0

12

28

28

2002

331

293

0

1314

64

0

0

5

5

0

0

0

0

0

0

0

0

18

6

10

0

2

0

0

0

0

0

0

0

0

0

0

0

0

14

14

31

0

0

31

0

0

0

0

2

0

2

6

6

9

9

71

0

0

36

5

30

0

0

0

97

0

97

0

0

0

0

0

0

0

0

0

0

0

0

0

0

0

0

0

0

0

0

0

0

0

0

0

0

0

0

0

0

0

0

0

0

0

24280

16032

0

0

0

16032

2

0

0

0

0

0

2

0

0

11644

0

846

3510

898

5815

18

114

0

170

273

0

4

4

0

0

2517

2517

121

121

36

36

0

0

3

3

0

36

0

0

0

4

0

2

0

2

28

0

0

1564

0

3

1561

105

0

13

6

86

0

8238

8194

4466

1995

2101

277

93

0

0

0

0

0

5

5

0

5

5

0

0

3718

0

3718

0

0

0

1

1

1

0

0

0

0

43

22

19

0

0

0

0

0

3

6

4

2

15

0

12

0

3

0

0

0

0

0

0

0

0

10

10

10

0

0

6

0

4

0

0

0

0

0

0

0

116

15

15

15

15

0

0

0

0

0

0

0

101

2

2

2

99

99

99

0

0

0

0

2

0

0

0

0

2

2

2

2

0

0

0

0

3

3

3

3

3

0

0

0

0

0

0

0

0

0

0

0

0

0

31

0

0

0

0

3

3

3

3

8

8

0

0

0

0

8

8

0

0

0

0

0

0

0

7

7

0

0

7

7

0

0

0

0

0

0

0

0

0

0

0

0

0

0

0

13

13

0

0

0

0

13

13

0

0

0

0

0

0

0

0

0

0

0

0

0

10

0

0

0

0

0

0

0

0

0

0

0

0

0

0

0

0

0

10

10

0

0

10

10
