# Supplementary material for: The effects of genital myiasis on the diversity of the vaginal microbiota in female Bactrian camels
Source: BMC Vet Res. 2022 Mar 5;18:87. doi: 10.1186/s12917-022-03189-5 (PMC8897907; doi:10.1186/s12917-022-03189-5)
Supplement: Supplementary file 5 — Additional file 5. [file 12917_2022_3189_MOESM5_ESM.zip › MPL201709200_16s_yy/Treat1/B10_krona/A08.html]

Javascript must be enabled to view this page.

members
magnitude
magnitudeUnassigned

A08

45890

45890

99

86

10

10

10

76

76

76

0

0

0

0

0

0

0

0

13

0

0

0

13

13

13

0

0

0

0

0

18974

18

18

18

0

0

18

0

0

0

0

6920

0

0

0

6920

445

0

440

5

560

560

11

0

9

0

2

0

0

8

8

3

3

27

27

0

125

2

0

22

0

71

0

18

12

0

44

0

44

0

3

3

5212

51

0

834

175

2

701

506

606

913

1424

0

460

35

0

394

0

31

22

22

0

0

12036

36

0

0

0

0

0

0

0

0

0

9

3

0

6

0

26

0

0

0

0

0

26

0

1

1

11987

7724

263

1342

5973

146

0

0

4251

4251

0

0

5

5

0

0

0

0

0

0

7

4

3

0

2

2

0

2

11

11

11

8239

8239

8239

6523

6523

1716

0

31

1685

2

0

0

0

0

0

0

0

0

0

0

0

0

0

0

0

0

0

0

0

0

0

0

0

0

0

0

0

0

0

0

0

0

0

0

0

0

0

0

0

0

2

2

0

0

0

0

0

0

2

2

0

0

0

0

0

0

0

0

0

0

0

0

0

0

0

0

0

0

0

0

0

0

0

0

0

0

0

0

0

0

0

0

0

0

0

0

0

0

0

0

2

2

2

2

2

1383

0

0

0

0

0

0

0

5

5

0

0

0

0

0

0

5

5

0

0

276

276

0

0

276

0

0

276

0

0

0

0

0

0

0

0

1102

1102

3

3

0

0

66

0

8

7

51

895

5

888

2

0

0

33

0

0

33

82

82

4

4

3

3

0

0

10

0

10

0

0

6

6

0

0

0

4

0

0

0

0

4

2

2

2

2

2

2

0

0

0

0

0

4969

0

0

0

0

0

0

0

0

8

8

8

0

8

0

0

0

0

0

0

0

0

0

0

0

0

0

4956

0

0

0

4956

0

0

0

29

0

10

19

438

51

0

0

376

11

7

0

5

0

2

16

0

0

0

16

0

0

0

0

0

0

0

0

0

8

8

0

0

31

31

0

0

5

0

5

13

13

2

2

77

0

0

44

31

2

0

0

0

0

0

118

0

0

118

3

0

3

0

0

2

2

0

0

4207

4207

5

5

5

5

48

48

5

5

5

1

1

1

42

42

42

11997

1097

0

0

0

65

65

0

26

2

0

35

2

2

2

0

2

0

0

0

0

0

0

0

0

0

0

0

0

1030

250

50

0

0

2

0

114

84

0

0

0

0

0

0

692

0

4

688

88

0

61

0

27

0

0

0

0

0

0

0

0

60

0

0

0

0

0

0

0

0

0

46

46

11

35

0

0

0

0

14

9

9

0

0

0

2

2

0

0

3

3

0

0

0

0

0

0

0

0

0

0

0

0

0

0

0

0

0

0

0

0

0

0

0

0

0

1734

982

982

0

3

979

0

0

0

0

0

0

0

0

0

0

0

0

0

0

0

452

183

17

0

3

163

269

42

227

0

0

0

0

0

0

0

0

0

0

0

298

292

0

0

0

290

0

2

6

6

0

0

0

0

0

0

0

0

0

0

0

2

2

2

2110

5

3

3

0

2

2

0

0

0

0

0

0

0

0

0

204

195

0

10

177

2

6

0

0

9

9

1750

164

104

60

17

17

0

2

0

2

0

0

0

0

0

17

5

12

29

29

0

0

1477

1477

44

44

0

0

0

132

132

0

20

26

0

86

5

5

5

9

0

0

9

0

0

0

9

5

5

5

0

0

0

6996

6996

6991

6989

2

5

0

5

0

0

0

0

0

14

6

6

6

6

0

0

0

0

0

0

0

0

0

0

0

0

0

8

8

0

0

8

8

16

16

12

12

12

4

4

4

0

0

0

0

0

0

0

0

0

0

0

0

0

0

0

0

0

0

0

0

0

0

0

0

0

0

0

0

0

0

0

0

0

0

0

0

0

0

0

0

0

0

0

0

0

0

0

0

0

0

0

0

0

0

0

0

0

0

0

0

0

0

0

0

0

0

0

0

0

0

0

0

0

0

0

0

0

0

0

0

0

0

0

0

0

0

0

0

0

0

0

0

0

0

0

0

0

0

0

0

0

0

0

0

0

0

0

0

0

0

0

0

0

101

43

43

39

39

4

4

2

2

2

2

56

56

56

56

0

0

0

0

0

0

0

12

12

12

12

12

0

0

0

0

0

0

0

0

0

0

0

0

0

30

5

5

5

5

0

0

0

0

25

0

0

0

25

25

25

0

0

0

0

0

0

0

0

0

0

0

0

0

0
