# Supplementary material for: The effects of genital myiasis on the diversity of the vaginal microbiota in female Bactrian camels
Source: BMC Vet Res. 2022 Mar 5;18:87. doi: 10.1186/s12917-022-03189-5 (PMC8897907; doi:10.1186/s12917-022-03189-5)
Supplement: Supplementary file 5 — Additional file 5. [file 12917_2022_3189_MOESM5_ESM.zip › MPL201709200_16s_yy/Treat1/B10_krona/A09.html]

Javascript must be enabled to view this page.

members
magnitude
magnitudeUnassigned

A09

46326

46326

26446

8290

8290

8290

8284

6

0

0

0

48

37

9

9

0

5

5

23

23

0

0

0

0

0

0

8

8

8

0

0

0

0

0

0

0

0

0

0

0

0

0

0

0

0

0

0

0

0

0

0

0

0

0

0

3

3

3

0

0

0

0

0

0

0

0

5237

102

102

0

5

44

0

0

53

0

0

0

7

7

0

7

0

0

0

0

0

0

0

0

0

2

2

2

5126

165

6

91

0

0

68

0

0

0

0

0

761

401

0

0

2

125

205

12

16

0

4200

4189

11

0

0

0

0

0

0

0

6838

261

261

0

4

101

156

0

54

26

0

26

0

28

28

0

0

0

6056

493

179

314

0

0

122

122

4988

4988

130

130

0

0

0

50

50

0

7

0

4

0

0

3

266

247

19

0

0

0

0

0

4

0

0

4

0

0

4

0

460

0

0

67

67

393

350

10

5

12

16

0

0

0

3

3

3

0

0

0

0

0

6033

0

0

0

4866

1669

0

55

1614

0

3197

126

3071

0

0

0

0

0

0

0

0

0

0

7

7

0

2

5

0

0

0

0

0

0

0

0

0

0

0

1116

14

14

1102

0

0

38

0

0

1064

0

0

0

0

0

0

34

34

20

14

0

0

0

10

10

10

11338

22

22

22

0

22

0

0

0

0

0

8801

0

0

0

0

8725

5373

3336

512

1501

24

3

0

3

0

0

0

0

0

0

3325

3325

0

0

0

0

24

24

0

0

0

0

76

0

0

0

0

13

13

13

0

0

3

10

26

18

8

0

0

24

2

0

9

2

0

9

2

2515

2515

2148

0

0

1052

11

524

350

0

33

0

30

148

0

0

46

0

0

0

40

6

7

7

0

0

198

24

0

174

2

0

0

0

0

0

2

32

32

0

0

0

0

0

23

0

5

13

2

3

0

0

0

0

25

25

34

0

34

0

0

0

0

0

0

0

0

0

0

0

0

0

0

0

0

0

0

0

0

0

0

0

0

0

0

0

0

0

0

0

0

0

0

0

0

0

0

0

0

0

0

0

0

0

0

0

0

0

0

0

0

0

0

0

0

0

0

0

0

0

0

0

0

0

0

0

0

0

0

0

0

0

0

0

0

18

18

18

18

18

0

0

0

0

0

16

16

0

0

0

0

0

0

0

0

0

0

0

0

0

0

16

16

16

0

0

0

0

0

0

0

0

0

0

0

53

53

14

14

14

39

39

39

0

0

0

0

0

0

0

0

0

0

267

63

0

0

0

63

63

63

204

0

0

0

204

204

204

0

0

0

0

0

0

0

0

6

6

6

6

6

0

0

0

0

0

0

0

0

0

0

0

0

0

0

0

0

0

0

0

0

0

0

0

3531

11

11

11

11

35

35

0

0

0

0

35

35

33

33

33

0

0

33

0

3452

0

0

0

3452

12

12

0

0

0

0

0

529

0

529

0

4

4

0

0

6

6

0

0

0

0

4

4

0

0

0

0

0

1816

1816

0

0

88

88

0

0

0

0

349

0

22

278

0

49

23

23

10

10

14

14

0

0

0

0

0

422

101

0

315

6

0

34

16

0

4

14

129

129

12

0

0

12

0

0

0

0

0

0

0

0

0

0

0

0

36

0

0

0

0

0

0

0

0

0

0

0

15

15

15

15

0

0

0

0

0

0

0

0

0

0

0

0

0

0

0

0

0

0

16

16

16

16

0

0

0

0

0

0

0

0

5

5

5

5

0

0

0

0

0

0

0

0

0

0

0

8

8

8

8

8

0

0

0

0

0

0

0

0

24

24

0

0

0

24

24

24

0

0

0

0

0

0

0

0

0

0

0

0

1030

5

5

3

0

3

2

2

0

0

0

0

0

0

0

0

10

10

10

0

10

0

0

0

0

0

0

0

936

936

0

0

936

726

0

210

79

79

0

0

0

0

0

0

0

0

0

0

8

0

0

0

8

54

0

0

54

0

0

13

3

0

4

6

4

4

0

0

0

0

0

0

0

0

0

0

0

0

0

0

0

0

0

0

0

0

0

3520

3520

3520

3301

3301

219

4

215

0

11

2

2

0

0

2

2

0

0

0

0

9

9

9

9

0

0

0

0

0

0

0

0

0

0

0

0

0

0

0

0

0

0

0

0

0

0

5

5

5

5

5

0

0

0

0

0

0

0

0

0

0

0

0

0

0

0

0

0

0

0

17

0

0

0

0

0

0

0

0

0

0

0

0

0

0

0

0

0

17

17

12

12

5

5
