# Supplementary material for: The effects of genital myiasis on the diversity of the vaginal microbiota in female Bactrian camels
Source: BMC Vet Res. 2022 Mar 5;18:87. doi: 10.1186/s12917-022-03189-5 (PMC8897907; doi:10.1186/s12917-022-03189-5)
Supplement: Supplementary file 5 — Additional file 5. [file 12917_2022_3189_MOESM5_ESM.zip › MPL201709200_16s_yy/Treat1/B10_krona/A10.html]

Javascript must be enabled to view this page.

members
magnitude
magnitudeUnassigned

A10

46272

46272

3

0

0

0

0

3

3

3

3

0

0

0

0

0

0

0

0

0

0

0

0

0

0

0

0

0

0

0

0

0

0

0

0

0

0

0

0

0

0

0

0

0

0

0

0

0

0

0

0

0

0

0

0

0

0

0

0

0

0

0

0

0

0

0

0

0

0

0

0

0

0

0

0

0

0

0

0

0

0

3

3

3

3

3

0

0

0

3483

191

191

191

0

33

158

0

0

0

0

0

0

0

0

0

0

0

0

0

0

0

0

0

341

341

341

340

1

0

0

0

0

0

0

0

2951

2951

66

8

48

10

0

5

5

12

0

12

0

7

7

0

0

0

3

3

3

3

2816

0

0

2816

0

0

0

0

35

35

0

0

4

0

0

3

1

232

2

2

2

2

3

3

3

3

227

1

1

1

226

226

82

144

0

0

0

0

0

0

1

1

1

1

1

11221

4772

2

2

2

3

3

0

3

4730

3

0

0

3

0

0

0

1270

0

0

1270

3456

201

1476

234

1545

0

0

0

0

1

1

0

37

0

0

18

2

0

0

9

2

1

4

0

0

16

2

0

14

0

0

0

3

3

0

0

0

6444

6444

1811

1811

4

0

0

0

0

4

0

70

4

8

9

39

9

0

0

0

1

14

14

381

381

16

16

0

0

0

345

339

6

0

3492

647

0

265

0

511

0

140

481

421

1027

0

253

3

238

5

0

7

0

0

7

7

51

51

0

0

0

0

0

5

5

5

0

0

0

5

0

0

0

2

0

0

0

0

0

0

0

0

2

2

2

2

15413

12391

12391

1

1

0

12390

0

12390

419

20

20

0

9

0

11

0

0

0

0

0

0

0

0

0

0

0

0

0

0

399

0

0

61

31

8

20

0

0

2

0

0

140

55

52

0

0

26

0

0

0

7

198

0

2

196

0

0

0

0

0

0

0

0

0

0

0

0

0

1830

0

0

0

0

0

0

137

3

3

134

11

0

0

0

123

0

0

0

0

0

0

0

0

0

0

1676

174

0

162

12

0

1502

1448

54

0

0

0

0

0

0

0

7

7

7

8

8

5

3

0

0

0

0

0

0

0

0

0

0

0

2

2

0

2

0

22

0

0

0

0

0

0

0

0

0

0

0

0

0

0

17

17

5

12

0

5

0

0

0

0

0

0

0

0

5

0

5

0

0

0

0

0

0

0

0

0

0

0

0

0

0

0

0

0

0

0

0

0

0

0

751

65

65

0

44

0

21

0

2

0

0

2

2

0

0

0

0

0

3

0

0

3

0

0

3

0

3

3

3

102

0

0

4

4

98

85

1

1

11

0

0

0

0

0

0

0

0

0

0

0

0

576

418

418

6

6

0

0

0

0

2

0

0

0

2

0

0

0

10

10

10

10

68

62

6

62

54

8

0

0

2

0

0

0

0

2

2

2

2

0

0

0

0

0

0

0

0

0

0

0

0

0

0

0

0

0

0

0

0

0

0

0

0

0

0

0

0

0

0

0

0

0

0

0

0

0

0

0

0

0

0

0

0

0

0

0

2

2

2

2

2

0

0

0

0

0

0

0

0

0

0

0

0

0

0

0

0

0

0

0

0

0

0

0

0

0

0

0

0

0

0

0

0

0

0

0

3

3

0

0

0

3

3

3

42

10

10

10

10

23

23

23

23

0

0

9

9

9

9

0

0

0

0

0

0

0

0

0

0

0

0

16

16

11

11

11

2

2

2

3

3

3

1

1

1

1

1

7

7

7

7

7

2

2

2

0

0

2

2

0

0

0

0

0

0

0

0

0

0

0

0

0

0

0

0

0

13

13

13

13

13

2584

0

0

0

0

0

0

0

0

0

0

0

2580

0

0

0

2580

0

0

3

3

53

53

1277

1277

0

0

2

2

0

3

0

3

2

2

0

0

0

0

31

31

0

0

0

0

0

0

0

0

0

3

3

0

0

0

0

0

0

0

0

0

36

10

4

2

0

20

4

1

0

3

0

40

1

38

1

19

0

0

19

0

0

1107

6

886

13

188

14

0

0

0

0

0

0

0

0

0

0

0

0

0

0

4

4

4

4

0

0

0

0

0

0

0

0

0

0

0

0

0

0

0

0

0

0

0

0

0

0

0

0

34

34

34

34

0

34

0

0

0

35

29

1

1

1

28

28

28

0

0

0

0

6

0

0

0

6

6

6

0

0

0

0

13173

13173

13173

2936

0

0

2936

10237

10237

0

0

0

0

0

0

0

0

0

0
