# Supplementary material for: The effects of genital myiasis on the diversity of the vaginal microbiota in female Bactrian camels
Source: BMC Vet Res. 2022 Mar 5;18:87. doi: 10.1186/s12917-022-03189-5 (PMC8897907; doi:10.1186/s12917-022-03189-5)
Supplement: Supplementary file 5 — Additional file 5. [file 12917_2022_3189_MOESM5_ESM.zip › MPL201709200_16s_yy/Treat1/B10_krona/A11.html]

Javascript must be enabled to view this page.

members
magnitude
magnitudeUnassigned

A11

46314

46314

56

34

34

34

34

22

22

0

0

22

22

0

0

0

0

21

0

0

0

0

0

0

0

0

21

21

21

21

240

0

0

0

0

44

0

0

0

44

44

44

0

0

0

0

196

175

175

175

21

21

21

16

0

0

0

0

0

0

0

0

0

0

0

0

0

0

0

13

0

0

0

0

0

0

0

0

0

13

0

0

0

0

13

13

0

0

0

0

0

0

0

0

0

0

0

0

0

0

0

0

0

0

3

3

3

3

0

0

0

0

0

0

0

0

0

0

0

0

0

0

0

0

0

0

0

0

0

0

0

0

0

0

0

0

0

0

0

45

0

0

0

0

19

19

0

0

19

19

0

0

0

0

0

0

0

26

26

26

26

0

0

9

0

0

0

0

9

0

0

0

9

9

9

8

8

8

8

8

0

0

0

0

0

22

0

0

0

0

0

0

0

0

0

0

0

0

0

22

22

0

0

3

3

19

19

0

0

0

0

0

0

0

0

0

0

0

0

0

0

0

0

0

0

0

0

0

0

0

0

0

0

0

0

0

0

0

0

0

0

10

10

10

10

10

0

0

0

0

15170

18

18

18

17

0

0

0

1

0

0

9067

94

0

0

0

0

8

0

8

0

0

0

0

62

0

17

0

45

24

0

0

5

0

0

3

16

35

35

35

8935

34

0

34

0

0

8050

241

419

2963

4427

817

0

0

817

3

0

3

0

0

31

0

0

31

3

3

3

0

6085

6085

4347

0

0

2108

284

840

0

0

60

148

706

201

185

15

2

0

21

0

68

65

14

0

298

298

522

463

13

0

35

11

200

200

7

7

93

12

81

239

4

0

235

12

0

0

0

0

5

7

2

2

108

0

0

108

22

22

50

50

0

0

0

18

18

16

16

16

1

1

1

1

1

1

3495

7

7

7

7

0

0

0

0

0

0

0

0

0

0

0

7

7

0

0

0

0

0

0

7

2

5

0

2692

2692

17

0

17

0

0

0

16

16

31

0

6

12

13

0

0

3

3

0

0

0

0

0

0

0

2625

0

2622

3

0

0

0

0

0

0

0

0

0

789

789

0

0

789

0

789

0

27

27

0

0

0

0

0

0

0

0

0

27

10

10

17

17

0

0

0

0

0

0

0

0

0

0

0

0

0

0

0

0

0

0

0

30

30

30

30

30

0

0

0

0

0

0

0

11

11

11

11

11

0

0

0

0

0

21

21

21

21

21

0

0

0

0

0

0

0

0

0

0

13

13

13

13

13

38

0

0

0

0

28

0

0

0

24

24

24

4

4

1

3

0

0

0

10

10

10

10

0

0

0

0

0

0

0

0

17847

4947

4947

4

0

4

4943

4942

1

5852

5

5

5

0

0

0

393

393

0

1

106

0

286

877

808

10

743

0

36

19

69

69

0

0

64

24

19

5

0

18

18

22

0

22

0

0

0

0

0

0

0

0

0

0

0

0

0

0

0

0

4513

15

2

0

0

7

6

0

0

103

103

3097

3097

107

107

0

0

0

14

14

598

588

10

484

73

411

95

95

0

4236

37

37

37

0

0

0

0

0

0

0

0

0

2

2

2

619

19

19

600

0

0

560

24

16

0

0

0

0

0

0

0

0

0

8

8

0

2

6

0

0

0

0

0

3567

3079

2946

133

488

2

73

401

12

0

0

0

0

0

0

0

3

3

0

3

0

2739

0

0

0

188

188

14

0

0

0

86

88

7

7

7

0

0

0

0

0

0

0

0

0

0

12

12

12

0

0

0

0

0

0

2532

0

0

0

0

344

0

4

66

274

0

0

1154

0

1154

0

1034

0

7

0

140

513

362

12

0

0

73

0

0

0

0

0

0

0

10

10

10

0

0

0

0

0

43

0

0

38

13

25

5

5

0

0

0

0

0

0

7

7

0

7

0

0

0

0

0

0

0

0

0

0

0

0

0

0

0

7

7

7

6

6

6

5870

5870

5870

3593

3504

89

0

2277

2277

13

13

13

13

13

3334

39

39

39

0

16

23

0

0

0

0

0

0

0

0

0

0

0

0

3261

3226

165

0

0

151

14

0

0

0

0

0

0

0

0

0

7

7

0

70

70

51

51

8

8

169

56

24

5

84

0

4

4

227

227

0

0

0

0

25

25

0

0

0

0

0

928

559

11

16

0

342

40

35

5

0

0

0

0

0

9

9

0

0

0

53

0

0

7

46

1463

1463

7

0

7

35

35

35

3

3

3

3

31

31

14

14

0

0

17

17
