# Supplementary material for: The effects of genital myiasis on the diversity of the vaginal microbiota in female Bactrian camels
Source: BMC Vet Res. 2022 Mar 5;18:87. doi: 10.1186/s12917-022-03189-5 (PMC8897907; doi:10.1186/s12917-022-03189-5)
Supplement: Supplementary file 5 — Additional file 5. [file 12917_2022_3189_MOESM5_ESM.zip › MPL201709200_16s_yy/Treat1/B10_krona/A12.html]

Javascript must be enabled to view this page.

members
magnitude
magnitudeUnassigned

A12

45993

45993

6

6

6

6

6

19107

4

4

4

4

0

0

0

0

0

0

4155

4145

6

0

0

2

0

4

0

538

480

16

25

16

1

36

36

5

5

101

0

0

23

21

2

0

1

0

54

7

7

91

0

0

91

299

0

7

292

265

265

18

0

18

19

19

2755

1076

0

319

385

0

39

99

127

442

0

268

5

5

10

10

10

14948

14866

2

2

0

0

0

0

0

5

5

0

0

5281

0

5272

9

6

6

0

9572

174

946

1561

6891

69

0

0

4

4

24

21

0

0

3

3

3

0

8

8

30

12

12

0

0

0

0

6

0

0

13

13

13

0

0

0

0

3

3

3

3

3

14

14

14

14

14

0

0

0

8507

8507

8507

2366

0

2351

15

6141

6141

0

0

0

0

0

0

0

0

0

0

0

0

0

0

0

0

0

28

16

16

16

16

0

0

12

12

12

12

0

0

0

0

0

0

0

0

0

0

0

0

0

7

0

0

0

0

7

7

7

7

192

179

151

151

151

28

28

28

13

0

0

0

13

13

13

0

0

0

0

0

0

0

0

0

0

0

0

0

28

4

4

4

4

20

3

3

3

0

0

0

17

17

17

0

0

0

0

4

4

4

4

0

0

0

0

0

0

0

0

22

0

0

0

0

0

0

0

0

0

0

0

0

0

3

0

0

0

3

0

0

3

3

0

0

0

0

0

0

0

0

0

0

0

0

0

0

0

0

0

0

0

19

19

0

0

0

0

0

0

19

19

0

0

0

0

78

40

40

40

40

36

36

0

0

36

36

2

2

2

2

11970

71

11

0

0

11

11

0

0

0

0

0

0

0

0

0

0

0

0

0

0

0

0

0

0

0

0

0

1

1

1

3

3

3

0

0

0

56

56

4

52

0

0

0

0

0

0

0

0

0

0

0

0

0

0

0

3317

3

3

3

20

8

0

8

0

12

0

12

0

0

2720

76

16

60

33

33

271

191

80

6

0

6

7

0

5

0

0

2

49

49

2117

2117

161

151

10

0

0

0

0

1

1

1

0

0

0

0

0

2

2

2

348

20

20

0

0

328

9

11

296

8

4

12

12

2

0

2

8

0

0

211

211

0

4

28

177

2

3423

0

0

0

0

0

0

2113

143

0

139

0

4

1970

48

1922

6

6

6

0

0

0

0

0

461

432

0

0

0

0

5

427

29

29

0

0

0

0

0

0

0

0

0

0

0

0

0

0

0

0

0

0

821

821

5

816

0

0

0

0

0

0

22

22

22

1690

1606

686

0

104

0

7

24

284

265

0

2

691

689

0

2

227

71

0

2

5

0

149

0

0

2

2

0

0

0

0

0

0

0

0

0

0

0

0

8

8

0

8

0

0

0

76

76

0

31

3

0

42

0

0

0

0

0

0

0

3469

3469

3467

3453

14

2

2

0

0

0

0

0

0

54

54

18

18

18

3

3

3

33

33

33

15

0

0

0

0

15

0

0

0

0

0

0

0

0

0

0

0

0

5

0

0

0

0

0

0

5

5

0

0

0

10

10

10

0

0

0

0

0

0

0

0

0

0

0

0

0

0

0

0

0

0

0

0

0

0

0

0

0

0

0

0

0

0

0

0

0

0

0

0

0

0

0

9

9

9

9

9

1

1

1

0

0

1

1

13

13

13

13

13

4848

4813

4809

22

22

0

91

0

2

89

0

0

0

0

120

0

6

114

4

0

4

276

0

163

105

8

0

10

10

0

0

0

0

0

0

0

0

0

11

5

4

2

0

18

0

10

8

0

0

0

12

0

12

8

0

6

2

104

39

10

0

0

55

90

90

0

0

24

4

20

4019

4019

0

0

0

0

4

4

4

17

17

17

1

0

0

16

1

1

1

1

0

0

0

0

0

0

0

0

0

0

0

0

0

0

0

17

17

17

17

8

4

4

4

4

4

4

4

4

0

0

0

0

25

25

25

25

25

0

0

0

0

0

0

0

0

0

0

0

0

1020

545

545

17

17

0

0

423

423

0

0

18

18

0

9

0

0

0

9

0

0

0

0

12

0

12

0

0

0

0

0

19

19

9

9

38

20

0

7

11

4

4

2

2

2

0

2

0

0

0

0

0

0

0

0

0

0

0

0

0

466

466

0

0

466

0

0

466

5

5

3

3

0

2

2

38

38

0

0

0

38

38

36

2

0

0

0

0

0

0

0

0

0

0

0

0

0

0

0

0

0

0

0

0

0

0

0

0

0

0

0

0

0

0
