# Supplementary material for: The effects of genital myiasis on the diversity of the vaginal microbiota in female Bactrian camels
Source: BMC Vet Res. 2022 Mar 5;18:87. doi: 10.1186/s12917-022-03189-5 (PMC8897907; doi:10.1186/s12917-022-03189-5)
Supplement: Supplementary file 5 — Additional file 5. [file 12917_2022_3189_MOESM5_ESM.zip › MPL201709200_16s_yy/Treat1/B10_krona/A13.html]

Javascript must be enabled to view this page.

members
magnitude
magnitudeUnassigned

A13

43109

43109

0

0

0

0

0

0

0

0

0

0

0

0

0

0

0

0

0

0

0

0

0

3215

0

0

0

0

0

0

0

0

0

0

0

0

0

0

0

0

0

0

0

14

14

14

0

0

0

14

3

3

3

3

3198

1

1

1

3197

19

0

0

3

16

0

0

0

0

0

0

21

0

2

0

10

9

0

0

2175

2175

0

0

0

0

0

0

3

3

0

0

0

0

0

41

6

2

33

6

0

3

0

3

3

3

3

3

0

2

2

0

0

0

844

131

0

534

0

179

3

3

0

0

4

4

0

0

40

40

33

0

0

33

2

2

2

2

2

0

0

0

0

0

0

0

0

0

0

44

39

39

39

39

0

0

0

0

0

0

0

5

5

5

5

0

0

0

0

0

0

0

0

0

0

0

0

0

0

0

0

0

0

10200

784

0

0

0

51

51

0

15

36

0

0

0

0

0

0

0

82

0

0

3

3

79

0

72

2

2

3

0

0

0

11

0

0

8

8

0

3

3

0

0

637

21

21

13

13

11

0

11

81

2

79

0

0

0

0

0

53

11

42

4

3

0

1

0

0

0

0

454

454

3

3

3

0

0

0

0

0

0

0

0

7972

7972

7972

7969

3

0

0

0

484

0

0

0

0

0

0

0

0

0

20

20

0

0

11

9

0

0

0

0

0

464

0

0

207

0

0

129

1

0

0

0

22

55

45

2

0

31

0

0

12

0

0

212

0

0

212

0

0

0

0

0

0

0

0

0

0

0

0

0

843

0

0

0

711

672

18

654

39

0

0

6

33

0

0

0

0

0

0

0

0

0

0

0

0

0

0

0

0

0

0

0

0

0

0

0

22

22

0

0

0

22

0

110

0

0

110

0

0

110

0

0

0

0

0

0

0

0

0

0

0

0

0

0

0

117

0

0

0

111

111

0

111

0

0

0

0

0

0

0

0

0

0

0

0

0

0

0

0

0

0

0

0

0

0

0

0

0

0

0

4

0

0

3

3

0

0

0

0

0

0

1

0

1

2

2

2

0

0

0

16301

7945

0

0

0

0

7917

0

0

0

3

0

0

3

3766

0

3766

0

0

0

0

0

1

1

0

4147

841

105

2367

834

22

4

0

0

4

0

0

0

0

0

17

0

0

0

0

0

0

17

1

0

1

0

0

0

0

6

6

6

7

7

7

0

0

0

0

0

6

1

8349

8349

12

0

0

0

0

6

6

0

0

0

692

0

0

692

6740

0

0

502

658

0

0

2015

359

1966

365

875

8

0

0

8

0

0

0

0

27

27

0

7

7

19

0

0

14

3

2

844

844

0

0

0

0

0

0

0

0

0

0

0

0

0

0

0

0

0

0

0

0

0

0

0

0

0

0

0

0

0

0

0

0

0

0

0

0

0

0

0

0

0

0

0

0

0

0

0

0

0

0

0

0

0

0

0

0

0

0

0

0

0

0

0

0

0

0

0

0

0

0

0

0

0

0

0

0

0

0

0

0

0

0

0

0

0

0

0

0

0

0

0

0

0

0

0

0

0

0

0

0

0

0

0

0

0

0

0

0

0

0

0

0

0

0

0

0

0

0

0

0

0

0

0

0

0

0

0

0

0

0

0

0

0

0

0

0

0

0

0

0

0

0

0

0

0

0

0

0

0

0

0

0

0

0

0

0

0

0

0

0

0

0

0

0

0

0

0

0

0

0

0

0

0

0

0

0

0

0

0

0

0

0

0

0

0

0

0

0

0

25

25

25

25

25

0

0

0

0

7

7

7

7

7

0

0

0

0

0

0

0

0

0

0

0

0

0

0

0

0

0

0

0

0

0

0

0

0

0

0

0

0

0

4

3

3

3

3

0

0

0

0

1

1

0

0

1

1

9

9

9

9

9

0

0

0

0

0

0

0

0

0

0

0

0

0

0

0

0

0

0

0

0

0

0

0

0

0

0

0

0

0

0

0

0

0

0

0

0

0

0

0

0

5549

0

0

0

0

0

0

0

0

0

0

0

0

140

140

140

0

0

140

0

0

644

644

644

644

0

0

0

0

4765

4765

1

0

1

0

0

3

3

4

4

21

4

1

16

0

0

0

0

0

0

0

1

1

0

0

0

0

0

0

0

0

0

4735

4735

0

0

0

0

0

0

0

0

0

0

0

6

6

6

0

0

6

6

0

0

0

0

0

0

0

0

0

0

0

0

0

0

0

0

0

7747

7747

7747

4661

4661

3086

3077

9

0
