# Supplementary material for: The effects of genital myiasis on the diversity of the vaginal microbiota in female Bactrian camels
Source: BMC Vet Res. 2022 Mar 5;18:87. doi: 10.1186/s12917-022-03189-5 (PMC8897907; doi:10.1186/s12917-022-03189-5)
Supplement: Supplementary file 5 — Additional file 5. [file 12917_2022_3189_MOESM5_ESM.zip › MPL201709200_16s_yy/Treat1/B10_krona/B01.html]

Javascript must be enabled to view this page.

members
magnitude
magnitudeUnassigned

B01

45847

45847

45

45

17

17

17

28

28

28

0

0

0

0

0

0

0

52

52

0

0

0

52

52

52

0

25

0

0

0

0

7

7

0

0

0

0

0

0

7

7

0

0

0

0

0

0

0

0

0

0

0

0

0

0

0

0

0

0

18

16

16

16

0

0

0

0

2

2

2

0

0

0

0

0

0

0

0

0

0

0

0

113

0

0

0

0

0

0

0

0

80

61

0

0

61

61

0

0

0

0

0

0

0

0

0

0

0

0

0

0

0

0

0

0

0

19

19

19

0

0

0

0

0

0

0

0

0

0

0

33

33

33

33

0

0

0

0

0

0

0

0

0

0

0

0

0

0

0

0

0

0

0

0

0

0

0

0

0

0

0

0

0

0

0

0

0

0

0

0

0

0

0

0

0

0

0

0

0

5

5

5

5

5

10

10

10

10

10

2

0

0

0

0

2

2

2

2

0

0

0

0

0

0

0

0

0

0

0

0

0

0

0

106

106

24

24

24

82

82

82

0

0

0

0

0

0

0

0

0

0

0

0

346

346

346

346

346

1261

3

3

3

3

1205

1180

0

0

0

34

34

0

0

0

115

0

23

0

92

0

0

0

0

0

20

0

20

0

0

0

0

0

0

26

26

7

7

38

38

0

0

0

0

0

0

323

4

0

319

26

0

3

0

23

110

2

8

22

0

78

8

0

0

8

296

296

0

0

0

0

0

0

0

0

0

67

67

110

110

25

25

25

0

0

0

0

0

0

0

11

11

11

11

0

0

0

0

0

0

0

42

42

42

42

0

0

0

0

0

0

0

0

0

0

0

0

0

0

0

0

0

0

0

0

32

32

32

32

32

0

0

0

0

0

3243

3243

3243

1949

1949

1294

1239

55

0

0

0

0

0

0

0

0

0

0

0

0

0

0

0

0

0

0

0

0

0

0

0

0

0

0

0

0

0

0

0

16785

1212

1212

1202

7

1195

10

0

10

3895

294

294

0

7

134

114

0

39

0

0

0

0

0

0

0

2

2

2

3599

0

0

555

2

13

373

152

0

15

0

0

2013

2013

0

0

1031

0

15

244

3

766

3

0

0

0

0

0

0

0

0

0

0

0

0

0

0

0

0

0

0

143

0

0

0

100

0

0

0

0

0

0

0

0

39

39

0

61

61

14

14

14

0

0

13

13

0

0

13

0

0

0

0

0

0

0

0

0

0

0

0

0

0

0

0

0

16

16

16

0

0

0

0

0

0

0

4553

1234

64

64

1170

1158

0

0

6

0

6

0

0

0

0

0

1675

1675

1675

0

0

0

0

0

0

0

0

0

0

0

0

0

0

0

0

0

0

0

0

0

0

0

9

9

9

13

13

13

13

13

0

0

13

0

0

0

1609

127

64

63

1482

10

1350

10

112

6982

0

0

0

140

51

0

0

51

89

52

37

0

0

2

0

0

2

2

5173

0

0

0

0

395

108

287

147

147

33

6

27

0

0

0

20

20

0

36

17

19

71

58

13

102

102

4369

4369

7

7

7

531

531

0

0

0

0

531

1113

98

98

0

0

1015

32

0

69

897

17

11

11

11

5

0

0

5

0

5

0

0

12

12

12

12

12

0

0

0

0

0

0

0

0

89

89

89

89

89

52

0

0

0

0

0

0

0

0

0

0

0

3

3

3

3

0

0

49

49

0

0

49

49

368

37

35

35

35

2

2

2

0

0

0

0

331

317

317

317

14

14

14

0

0

0

0

4907

1516

1516

0

0

1516

0

0

1516

2

2

0

0

2

2

0

0

7

7

7

7

0

0

0

3353

3353

0

0

25

0

0

25

40

35

5

0

0

0

0

7

7

0

0

0

0

0

212

212

0

0

21

0

0

21

0

0

0

0

0

3048

3043

0

5

29

29

0

0

29

3

0

26

0

0

0

0

7

0

0

0

0

0

0

0

0

0

0

7

7

7

7

18387

112

112

112

23

26

0

38

0

23

2

12555

0

0

0

12555

0

0

4

4

420

0

79

0

0

0

50

0

174

117

0

0

384

357

0

0

13

14

348

346

0

2

0

0

0

369

369

482

145

311

26

10274

0

0

89

677

41

460

2250

1403

4766

66

522

0

0

0

14

14

196

0

0

196

64

64

5720

31

31

31

170

49

26

21

2

0

0

0

0

21

8

13

0

0

0

0

6

6

91

0

0

91

0

3

3

0

0

0

0

5519

906

0

0

906

0

0

0

37

0

37

0

27

24

3

4549

1430

3048

41

30

0

0

0

0
