# Supplementary material for: The effects of genital myiasis on the diversity of the vaginal microbiota in female Bactrian camels
Source: BMC Vet Res. 2022 Mar 5;18:87. doi: 10.1186/s12917-022-03189-5 (PMC8897907; doi:10.1186/s12917-022-03189-5)
Supplement: Supplementary file 5 — Additional file 5. [file 12917_2022_3189_MOESM5_ESM.zip › MPL201709200_16s_yy/Treat1/B10_krona/B02.html]

Javascript must be enabled to view this page.

members
magnitude
magnitudeUnassigned

B02

45696

45696

0

0

0

0

0

11

4

4

4

4

0

0

0

0

7

7

7

7

150

150

150

150

150

73

73

0

0

0

73

73

73

199

0

0

0

0

136

0

0

0

26

26

26

0

0

0

0

0

0

110

62

62

0

0

15

15

33

33

0

0

0

0

0

0

0

0

0

23

23

23

23

0

0

0

0

0

0

0

0

24

24

24

24

16

16

16

16

0

0

0

0

0

0

0

0

0

0

0

0

0

0

0

0

85

0

0

0

0

0

0

0

0

0

0

0

13

13

13

13

0

0

72

72

12

12

60

60

0

0

0

0

0

4

4

4

0

0

4

4

697

119

21

21

21

98

98

98

0

0

0

0

578

0

0

0

578

578

578

0

0

0

0

0

0

0

0

0

0

0

0

0

0

0

0

0

153

0

0

0

0

45

0

0

0

45

0

0

45

45

0

0

44

44

44

44

0

0

0

0

0

0

0

0

0

0

0

0

0

0

0

0

0

0

0

0

0

0

64

64

0

0

64

64

0

0

0

0

0

0

0

0

4718

519

519

0

0

106

106

11

11

0

0

0

0

0

0

0

0

0

0

0

0

329

329

0

0

73

0

73

0

0

0

0

0

0

0

0

0

0

0

0

137

137

36

2

34

0

101

101

0

0

0

0

9

9

0

0

0

9

9

16

16

0

0

0

0

16

16

4037

4037

4037

4019

0

18

0

0

12

12

12

12

12

0

0

0

0

0

0

0

0

0

0

0

0

0

0

0

0

0

0

0

68

0

0

0

0

3

3

3

3

65

0

0

0

0

0

0

0

0

0

30

0

0

30

30

35

35

35

0

0

0

0

0

0

0

0

0

0

0

0

0

0

2337

0

0

0

0

0

0

0

0

0

0

0

0

0

0

0

0

0

0

0

0

0

0

0

0

0

0

0

0

0

0

2337

2337

0

0

272

272

0

0

0

511

511

0

0

182

0

4

160

18

0

0

0

0

98

98

6

6

44

0

0

4

11

29

15

15

2

2

0

0

0

0

0

0

0

0

0

0

0

0

0

49

49

19

19

298

22

0

153

123

0

42

42

0

31

13

18

0

0

0

0

768

100

0

0

668

0

0

0

0

0

0

0

0

0

0

0

0

0

37

37

37

37

37

20

20

20

20

20

0

0

0

0

0

0

0

0

0

0

0

0

0

0

0

0

0

0

0

0

0

0

0

0

0

0

0

0

0

0

0

0

0

0

0

26878

299

19

19

19

18

18

18

16

0

0

16

16

0

0

0

0

0

0

0

0

0

0

0

0

0

24

24

0

24

38

38

38

164

0

0

0

0

20

20

110

110

0

0

34

0

34

20

20

20

0

0

0

0

0

0

5886

0

0

0

3

3

3

0

0

0

0

0

0

589

589

320

0

10

240

19

0

0

0

0

15

15

15

242

242

0

242

5037

0

0

3224

0

3224

0

0

0

1119

0

360

16

12

0

0

0

694

37

694

585

0

0

0

6

103

0

0

0

13215

10721

0

0

11

0

11

244

244

838

257

581

290

290

8821

8821

80

0

0

0

80

0

234

163

71

203

203

0

0

0

1415

0

0

1317

28

61

56

1172

0

98

98

35

0

0

35

6

0

21

8

925

925

0

18

11

896

0

0

0

0

16

12

12

4

4

9

9

9

0

0

0

94

44

44

0

0

0

50

35

15

0

2035

2035

0

0

0

2035

2018

17

5443

0

0

0

127

127

127

0

0

0

4333

130

130

4203

4190

3

0

0

0

10

0

0

0

899

523

3

184

0

336

376

133

243

0

0

0

0

0

0

0

0

0

0

0

0

0

0

0

0

0

0

84

84

0

35

0

0

49

0

0

0

0

0

0

0

0

23

0

0

0

0

23

23

23

23

0

0

0

4285

2595

17

17

17

117

0

0

0

0

0

0

0

0

0

31

0

0

0

31

86

33

0

0

7

4

0

42

0

0

3

3

3

0

2458

75

0

0

75

2237

307

45

1875

10

60

7

53

78

67

0

11

0

0

0

0

8

8

0

30

30

30

0

11

0

0

0

19

0

1660

1660

0

0

132

0

18

36

73

5

0

0

0

0

0

0

0

260

260

6

0

0

0

0

6

0

0

0

316

284

0

32

30

30

0

0

290

290

192

0

0

60

46

19

0

67

0

0

434

0

0

116

28

0

18

74

166

0

32

0

0

0

0

5885

5885

5885

3033

2993

40

0

2852

2852

61

61

61

17

17

44

30

14

0

0
