# Supplementary material for: The effects of genital myiasis on the diversity of the vaginal microbiota in female Bactrian camels
Source: BMC Vet Res. 2022 Mar 5;18:87. doi: 10.1186/s12917-022-03189-5 (PMC8897907; doi:10.1186/s12917-022-03189-5)
Supplement: Supplementary file 5 — Additional file 5. [file 12917_2022_3189_MOESM5_ESM.zip › MPL201709200_16s_yy/Treat1/B10_krona/B03.html]

Javascript must be enabled to view this page.

members
magnitude
magnitudeUnassigned

B03

46042

46042

923

28

0

0

0

28

28

28

0

0

0

0

895

25

25

25

870

870

870

0

0

0

0

45

45

45

29

29

16

16

0

0

0

0

0

61

0

0

0

0

2

0

0

0

0

0

0

0

0

0

0

0

0

0

0

2

2

2

0

0

0

59

59

59

59

0

0

0

0

0

0

0

0

9689

7819

7819

21

21

1567

22

55

0

6

0

0

1390

94

0

191

2

189

0

710

710

228

77

151

0

0

0

0

76

76

1416

1416

107

0

43

64

172

8

0

30

64

52

18

2160

747

0

400

79

0

61

17

490

0

135

231

1171

993

19

21

138

0

0

0

0

398

398

398

81

25

197

39

14

32

10

1472

0

0

0

0

355

36

36

0

0

0

0

0

0

0

98

28

15

0

44

0

11

0

221

41

0

166

14

0

0

1089

0

0

684

366

7

2

309

54

46

0

8

287

73

0

214

26

0

26

9

9

29

19

10

28

28

28

389

149

149

149

149

240

240

58

58

182

182

0

0

0

0

34

0

0

0

0

34

34

34

34

0

0

0

0

0

0

0

0

227

227

0

0

0

0

0

0

227

227

227

14

0

0

0

0

14

14

14

14

166

0

0

0

0

0

0

0

0

0

0

0

0

0

0

0

0

0

0

0

0

0

0

0

1

1

1

1

0

0

0

0

165

54

54

54

0

0

0

0

0

0

0

0

0

0

0

0

0

0

0

0

0

0

35

35

35

0

0

0

76

76

76

0

0

0

0

71

0

0

0

0

0

0

0

71

71

0

0

71

71

0

0

0

0

0

0

0

0

0

0

0

0

0

0

0

59

59

59

59

59

0

0

0

0

0

89

89

89

89

89

0

0

0

179

179

179

179

179

43

43

0

0

0

43

43

43

0

10

10

10

10

10

14

0

0

0

0

3

3

3

3

11

11

11

11

25108

5876

0

0

0

844

844

83

680

37

30

14

0

0

0

0

0

0

20

20

20

0

0

0

20

0

0

20

20

0

0

3096

89

89

3007

0

0

0

0

3007

0

0

0

0

0

0

51

0

0

51

51

0

0

0

1739

1114

191

923

625

500

36

21

68

106

106

106

1496

1496

8

0

8

1488

1470

18

6384

0

0

0

2

2

2

53

53

53

702

702

0

0

0

290

17

395

44

44

44

44

44

44

0

0

0

0

0

0

5539

1206

1

0

1

0

21

572

138

473

0

3481

3441

0

40

19

19

0

0

833

25

257

0

0

0

551

0

0

0

0

806

292

81

81

195

0

195

16

16

0

0

0

0

0

0

53

53

53

0

0

0

0

0

0

0

0

0

0

24

24

24

37

37

37

317

317

317

0

0

46

46

46

0

0

0

0

0

37

26

26

11

11

0

0

0

10546

741

741

710

0

19

12

0

44

1

1

43

43

0

0

0

15

15

15

1214

49

49

37

37

1128

33

31

1047

0

17

0

0

0

8377

221

221

0

0

951

179

772

121

0

121

10

10

36

14

22

3

3

0

6846

6846

60

0

0

7

0

53

129

129

103

0

0

53

53

0

0

50

0

50

52

52

31

0

7

14

0

0

41

41

41

41

41

3

3

3

0

0

3

3

28

28

28

28

28

0

0

0

0

0

2959

0

0

0

0

0

0

0

0

4

0

0

0

0

0

4

0

0

4

4

2706

0

0

0

2706

91

1

0

88

2

0

0

0

45

45

1

1

388

388

307

0

268

39

0

0

106

34

0

1

71

0

0

35

35

256

5

1

78

73

99

100

100

247

247

0

0

168

168

0

0

23

23

0

0

2

2

0

0

0

0

0

0

29

29

0

898

0

0

898

10

0

0

9

0

1

0

0

0

113

113

113

113

136

136

136

0

86

0

50

38

0

0

0

0

0

0

0

0

38

0

0

0

0

0

0

0

0

0

0

38

38

38

124

0

0

0

0

112

112

0

0

0

0

0

0

112

112

0

0

0

0

0

0

0

0

0

0

0

0

10

10

0

0

10

10

0

0

0

0

0

0

0

0

0

0

0

0

2

2

2

2

0

0

0

0

0

0

0

3723

1856

1856

12

12

0

0

193

19

174

0

0

0

23

23

331

231

0

33

67

426

426

39

0

0

39

0

8

8

108

51

57

21

21

695

665

30

0

0

0

5

5

1

1

0

4

4

1828

1828

1828

0

1

1827

0

0

24

24

0

0

0

0

0

0

24

23

0

1

10

10

0

0

10

10

0

0

2005

2005

2005

740

740

1265

1103

162

0
