# Supplementary material for: The effects of genital myiasis on the diversity of the vaginal microbiota in female Bactrian camels
Source: BMC Vet Res. 2022 Mar 5;18:87. doi: 10.1186/s12917-022-03189-5 (PMC8897907; doi:10.1186/s12917-022-03189-5)
Supplement: Supplementary file 5 — Additional file 5. [file 12917_2022_3189_MOESM5_ESM.zip › MPL201709200_16s_yy/Treat1/B10_krona/B04.html]

Javascript must be enabled to view this page.

members
magnitude
magnitudeUnassigned

B04

44408

44408

49

49

49

49

49

5

0

0

0

0

5

5

5

5

0

0

0

0

0

0

0

0

0

0

0

0

0

13

0

0

0

0

0

0

0

0

0

0

0

0

0

0

0

0

0

0

0

0

0

0

0

0

0

13

13

13

13

3854

10

10

0

0

10

10

0

0

1606

1606

0

0

1606

0

1606

0

30

30

16

10

6

14

14

2194

2194

2080

2080

0

0

0

0

0

3

0

3

0

15

15

0

0

0

0

0

0

43

43

0

0

0

0

13

0

2

0

11

40

0

0

33

7

0

0

14

14

0

0

9

0

9

0

5

5

0

0

0

0

0

0

0

0

0

0

0

0

0

0

0

0

0

0

0

0

0

0

16

0

0

0

0

16

16

16

16

0

0

0

0

2

2

2

2

2

0

0

0

0

0

24

2

2

2

2

7

7

7

7

0

0

0

0

0

0

0

10

10

10

10

0

0

0

0

0

0

0

0

0

0

0

0

0

0

0

0

5

0

0

0

0

0

0

0

0

0

0

0

0

0

0

0

5

5

5

0

0

0

0

0

0

0

0

0

0

0

0

55

55

0

0

0

55

55

55

0

0

0

0

0

2

2

2

2

2

0

0

2035

0

0

0

0

0

0

0

0

0

0

0

34

34

34

19

7

8

0

0

0

0

0

0

0

0

0

2001

0

0

0

2001

19

15

4

252

0

211

4

29

8

0

0

82

82

0

0

66

12

5

39

10

68

68

6

6

0

2

2

0

0

0

0

522

522

352

0

352

0

3

3

3

0

3

0

0

0

0

0

0

0

0

0

2

2

21

21

0

355

23

222

110

0

0

0

0

215

215

33

33

0

0

0

0

0

0

0

0

0

5065

1997

0

0

0

1658

6

0

6

1239

618

0

621

0

210

8

202

0

45

0

10

35

0

0

157

157

0

1

1

331

53

53

0

107

0

0

14

69

16

0

8

0

0

0

0

2

2

0

0

169

0

15

18

136

8

8

6

2

14

14

14

0

0

0

7

7

0

0

3054

3054

1

1

0

0

2069

468

55

26

277

450

0

0

512

0

17

264

78

31

7

40

0

0

0

0

14

14

62

0

3

9

0

24

0

0

0

26

0

0

109

109

0

0

0

328

328

184

0

0

171

0

13

0

209

0

209

0

0

0

0

0

0

0

0

0

0

0

0

0

0

7

7

7

7

7

103

10

10

10

10

0

0

0

93

93

85

85

8

8

0

0

0

0

0

0

0

0

0

0

17706

59

0

0

0

0

0

0

11

11

11

0

0

0

0

0

0

10

10

10

13

13

13

0

0

0

0

0

0

0

0

16

0

0

13

13

0

0

0

3

3

0

0

0

0

0

0

0

9

9

9

0

0

0

0

0

7630

789

31

31

90

90

668

618

0

8

29

13

27

27

0

0

18

9

0

0

520

520

0

0

64

0

456

6202

93

0

93

87

35

52

164

164

5286

5286

0

0

4

0

0

4

0

0

341

148

193

220

220

4

4

3

0

3

11

11

11

10

10

10

8

8

8

0

0

0

0

0

63

28

0

0

28

0

0

35

35

0

2918

2918

2918

2876

42

0

0

0

3122

1

1

0

1

0

0

0

0

0

0

238

238

145

0

0

0

0

93

0

0

0

9

9

9

0

0

0

0

0

0

2874

608

2

0

0

2

206

32

366

0

0

0

0

0

0

1883

0

0

1883

383

4

57

0

322

0

0

0

0

0

3977

1237

436

30

404

0

2

801

97

704

0

0

0

0

0

0

52

52

52

0

0

0

2

2

2

0

0

0

0

0

0

0

12

12

12

9

9

5

0

4

4

4

4

0

0

16

16

0

7

9

0

0

2636

2563

26

0

10

2

0

2525

73

73

9

9

9

408

0

0

0

0

51

51

51

51

0

0

0

0

0

0

0

357

342

342

342

15

15

15

11

11

11

11

11

0

0

0

0

0

0

0

0

0

0

0

12

12

12

12

12

13

0

0

0

0

0

0

0

0

0

0

0

0

0

13

13

0

0

13

13

0

0

0

0

0

0

0

0

0

0

0

0

0

0

0

0

0

0

0

0

0

0

0

0

0

0

0

0

0

0

0

0

0

0

0

0

0

0

0

0

0

0

0

0

0

0

0

0

0

0

0

0

0

0

0

0

0

0

0

0

0

0

0

0

0

0

0

0

0

0

0

0

0

0

0

0

0

0

0

0

0

0

0

0

15028

15028

15028

9417

27

0

9390

5611

5611
