# Supplementary material for: The effects of genital myiasis on the diversity of the vaginal microbiota in female Bactrian camels
Source: BMC Vet Res. 2022 Mar 5;18:87. doi: 10.1186/s12917-022-03189-5 (PMC8897907; doi:10.1186/s12917-022-03189-5)
Supplement: Supplementary file 5 — Additional file 5. [file 12917_2022_3189_MOESM5_ESM.zip › MPL201709200_16s_yy/Treat1/B10_krona/B05.html]

Javascript must be enabled to view this page.

members
magnitude
magnitudeUnassigned

B05

45961

45961

34

34

34

34

0

34

0

0

0

13842

3687

0

0

0

1570

1570

1562

8

0

0

0

5

5

5

0

0

0

9

9

9

0

0

0

165

165

0

67

2

3

90

3

5

5

5

1933

301

111

156

27

0

3

0

4

0

0

0

0

213

9

0

129

70

5

0

1419

1415

4

0

0

0

4180

4180

4180

4154

26

0

0

0

233

11

11

11

88

88

88

0

0

0

0

0

0

0

0

0

0

0

80

3

3

44

44

0

0

0

0

0

33

33

0

0

0

0

0

0

0

0

0

0

0

0

0

0

0

0

0

54

54

54

0

0

0

0

0

4002

6

6

6

0

0

0

0

0

243

243

0

240

0

3

0

360

38

38

321

0

20

268

25

8

1

1

4

4

4

3333

0

0

22

0

15

0

7

0

2848

2848

0

0

57

56

1

85

85

199

94

105

81

81

0

0

0

41

10

31

0

0

0

54

22

0

0

22

32

0

32

0

0

0

0

0

2

2

2

0

0

1740

0

0

0

0

0

0

787

677

45

625

7

0

110

11

99

0

0

0

86

86

6

0

0

0

80

54

54

54

12

8

8

4

4

0

0

0

0

0

0

0

0

0

0

801

786

0

759

3

8

12

4

15

15

0

0

0

0

0

0

0

0

0

0

0

9048

2348

0

0

0

2296

0

0

0

0

0

11

1

10

0

0

198

198

0

0

2087

18

554

0

1515

0

0

0

0

0

0

0

0

52

0

0

0

0

13

0

6

0

7

8

0

8

0

0

16

0

0

0

0

0

16

0

15

15

68

68

68

0

0

0

68

0

0

0

6632

6632

0

0

55

0

4

34

0

0

0

0

0

17

1707

1703

4

0

0

0

124

0

124

168

128

4

0

36

0

0

0

702

702

0

0

12

4

0

0

0

8

0

4

0

4

0

2741

1883

76

172

65

0

50

0

42

433

0

20

1119

1119

0

0

0

6830

764

764

764

764

0

0

0

0

14

14

2

2

0

0

12

0

6

6

0

0

9

9

9

9

0

0

0

6043

6043

659

0

0

659

5262

0

5262

0

78

78

0

0

16

16

0

0

0

0

0

0

0

0

0

28

0

17

11

0

0

0

0

0

0

0

0

0

0

0

0

0

0

0

0

0

0

3124

8

0

0

0

0

0

8

0

0

8

8

20

20

0

0

18

18

2

2

3070

20

20

20

3050

0

0

0

0

0

11

11

140

140

0

0

25

25

2254

72

1922

260

0

0

164

7

4

151

2

1

1

59

59

52

6

8

38

5

5

0

0

6

6

0

16

0

0

0

16

18

18

105

93

12

0

0

0

0

0

0

0

0

0

0

0

0

0

0

0

194

0

0

194

0

0

0

0

0

5

5

5

5

21

21

21

0

6

0

15

54

20

20

4

4

16

16

34

34

34

34

0

0

0

0

0

0

0

0

0

0

0

0

0

11829

11829

11829

3805

33

0

3772

8024

8024

15

13

13

13

13

0

0

0

0

2

2

0

0

2

2

0

0

0

0

0

12

12

12

0

0

0

0

0

12

12

27

27

27

27

27

0

0

0

0

0

0

0

0

0

0

0

0

0

10

10

10

0

0

10

10

0

0

0

0

0

4

0

0

0

0

4

4

4

4

285

243

243

243

243

0

0

0

0

0

0

0

0

0

0

0

42

0

0

0

42

42

42

31

0

0

0

0

0

27

27

20

20

7

7

0

0

0

0

0

0

0

0

0

0

0

0

0

0

0

0

0

0

0

0

0

0

0

0

0

0

0

0

0

0

0

0

0

0

0

4

4

4

4

0

0

0

0

0

15

15

3

3

3

12

12

12

15

15

0

0

0

0

0

0

0

0

0

0

0

0

15

0

0

0

0

0

0

15

15

0

0

0

0

0

0

0

0

0

0

0

0

0

0

0

0

0

0

0

0

0

0

0

0

0

0

0

0

0

0

0

0

0

0

0

0

0

0

0

0

0

0

0

0

0

0

0

0

0

0

0

0

0

0

0

0

2

0

0

0

0

0

0

0

0

2

2

2

2

724

5

5

5

5

718

0

0

0

574

574

574

0

144

144

144

0

0

0

1

1

1

1

0

0

0

0

0

0

0

0

0

0

0

60

60

60

60

60

0

0

0

0

0

0

0

0

0

0

0

0

0

0

0

0

0

0

0

0

0

0

0

0

0

0

0

0

0

0

0

0

0

0

0

0

0

0

0

0

0

0

0

0

0

0

0

0

0

0

0

0
