# Supplementary material for: The effects of genital myiasis on the diversity of the vaginal microbiota in female Bactrian camels
Source: BMC Vet Res. 2022 Mar 5;18:87. doi: 10.1186/s12917-022-03189-5 (PMC8897907; doi:10.1186/s12917-022-03189-5)
Supplement: Supplementary file 5 — Additional file 5. [file 12917_2022_3189_MOESM5_ESM.zip › MPL201709200_16s_yy/Treat1/B10_krona/B06.html]

Javascript must be enabled to view this page.

members
magnitude
magnitudeUnassigned

B06

46344

46344

0

0

0

0

0

0

0

0

0

0

0

0

0

0

0

0

0

0

0

0

0

0

0

0

0

0

0

0

0

0

59

59

59

58

0

19

39

1

1

3

3

3

3

3

0

0

0

0

0

0

0

0

12

12

12

12

12

0

0

0

0

0

0

0

0

0

0

0

0

0

0

0

0

0

0

0

0

0

0

0

0

0

0

0

0

0

0

0

0

0

0

0

0

0

14

0

0

0

0

0

0

0

0

0

0

0

0

0

0

0

0

0

0

0

0

8

8

0

0

7

7

0

0

1

1

0

0

0

0

0

0

0

0

0

0

0

0

6

6

6

6

0

0

0

0

0

0

0

0

0

32882

7344

0

0

0

0

0

0

0

7307

0

0

0

0

7269

19

974

6276

0

10

10

0

4

0

4

0

0

0

0

24

0

6

18

37

21

0

0

21

0

0

0

0

0

0

0

0

14

0

0

9

0

0

0

5

2

2

0

0

25535

0

0

0

25535

46

0

8

0

0

25

13

0

0

0

3

3

0

0

0

0

11

0

0

0

10

1

7

4

0

0

0

0

3

1

1

0

0

0

0

0

0

0

0

0

0

0

0

0

0

0

25467

1206

0

0

18087

0

299

2787

1408

1680

0

0

3

3

3

0

0

0

0

0

0

3

161

134

134

134

134

0

0

0

0

0

0

0

27

27

27

27

0

0

0

0

0

0

0

6011

0

0

0

0

0

0

0

0

0

0

0

0

0

0

0

5618

5618

0

0

0

0

0

0

0

0

0

0

0

71

8

0

63

0

0

0

0

0

0

5539

0

0

5539

0

0

0

0

0

0

0

0

0

0

8

8

393

393

0

0

393

0

0

393

0

0

0

0

0

0

0

0

0

0

0

0

0

0

0

0

0

0

0

0

0

0

0

0

0

0

0

0

0

0

0

0

0

0

0

0

0

0

0

28

0

0

0

0

0

0

0

0

0

0

0

0

28

0

0

0

0

0

0

0

0

0

0

0

0

0

0

0

28

0

0

0

0

15

15

13

13

0

0

0

0

0

0

0

0

0

0

0

0

0

0

0

0

0

0

0

0

0

0

0

0

0

0

0

0

0

0

0

0

0

0

0

0

0

0

0

0

0

0

0

0

0

0

0

0

0

0

0

0

0

0

7

7

7

7

7

0

0

0

70

0

0

0

0

70

0

0

0

70

70

70

18

0

0

0

0

0

0

0

0

0

0

0

0

0

0

0

0

0

18

18

18

18

0

0

0

0

0

0

0

0

0

0

0

0

0

0

0

0

0

0

0

0

0

0

0

0

0

36

36

36

36

36

0

0

0

0

0

0

0

0

0

0

0

0

0

0

0

0

0

5165

36

36

36

29

7

0

0

0

189

0

0

0

0

0

0

0

0

0

0

0

0

0

0

0

0

0

0

0

0

0

0

0

0

0

0

0

0

0

0

159

159

0

0

159

30

0

0

0

0

19

19

0

0

0

0

0

11

11

0

0

0

0

0

1066

30

30

30

0

0

242

70

40

30

172

2

13

154

3

0

0

0

0

0

0

0

0

0

217

217

0

0

217

0

0

0

0

0

20

20

20

0

0

0

553

467

0

0

464

0

0

3

86

86

4

0

0

4

4

0

0

0

0

0

0

0

0

0

0

1461

0

0

0

0

0

0

1

1

1

0

0

0

966

535

0

535

0

157

0

0

14

70

73

0

0

0

274

57

7

0

0

0

199

11

0

0

0

0

0

0

0

0

0

0

111

111

0

0

7

49

55

0

383

383

383

0

0

0

0

2413

8

5

5

3

0

0

3

0

47

10

10

0

37

34

0

3

0

0

153

153

0

0

0

0

153

0

0

0

0

0

1942

37

21

16

43

43

0

0

0

0

0

0

0

53

53

1613

1613

0

0

0

0

0

0

0

155

69

86

41

41

0

0

0

8

8

8

0

0

0

255

248

0

3

16

2

227

7

7

0

0

1878

1859

1854

16

0

16

11

11

0

0

0

0

0

0

0

0

0

0

0

0

0

0

0

0

3

3

0

0

0

4

0

0

0

0

4

19

13

0

6

0

6

6

7

7

0

0

0

975

975

0

0

0

0

98

98

6

6

0

70

15

0

0

0

55

0

0

508

3

505

0

0

0

131

0

0

131

5

5

5

17

17

3

3

14

14

0

0

0

0

0

0

0

0

0

0

0

0

0

0

0

0

0

0

0

0

2

2

2

2
