# Supplementary material for: The effects of genital myiasis on the diversity of the vaginal microbiota in female Bactrian camels
Source: BMC Vet Res. 2022 Mar 5;18:87. doi: 10.1186/s12917-022-03189-5 (PMC8897907; doi:10.1186/s12917-022-03189-5)
Supplement: Supplementary file 5 — Additional file 5. [file 12917_2022_3189_MOESM5_ESM.zip › MPL201709200_16s_yy/Treat1/B10_krona/B07.html]

Javascript must be enabled to view this page.

members
magnitude
magnitudeUnassigned

B07

42129

42129

2

2

2

2

2

4

4

4

4

4

0

0

0

0

0

0

0

0

0

0

0

0

1

1

1

1

1

2

0

0

0

0

0

0

0

0

2

2

2

2

6

6

4

4

4

2

2

2

30

24

24

24

24

4

0

0

0

4

4

4

0

0

0

0

0

0

0

2

2

2

2

3490

7

7

2

2

5

0

5

12

12

0

0

8

8

0

0

4

2

0

2

3

3

3

3

0

0

0

0

124

124

124

0

12

112

0

0

3344

3344

13

0

13

102

0

2

100

19

19

0

0

390

193

24

173

0

149

149

2473

2

4

2467

0

0

0

0

39

12

2

0

25

39

39

15

15

105

105

4

4

4

4

4

0

0

0

0

0

0

0

0

1

1

1

1

1

0

0

0

0

0

0

0

0

0

0

0

0

0

0

0

0

0

0

0

0

0

0

0

0

0

0

0

0

0

0

0

0

0

0

0

0

0

0

0

0

0

0

0

0

0

0

0

0

0

0

0

0

0

0

0

0

0

0

0

0

0

0

0

0

0

0

0

0

0

0

0

192

136

136

136

136

0

0

0

0

56

56

54

54

2

2

10677

7441

7441

0

0

0

7441

7439

2

394

0

0

0

0

0

0

0

0

0

39

39

10

3

26

0

0

0

0

0

0

0

0

0

352

165

88

0

2

2

24

0

49

0

0

1

1

14

0

0

4

0

10

0

172

0

7

165

0

0

0

0

0

3

3

3

0

0

0

0

224

0

0

0

207

207

4

0

203

0

0

0

0

0

0

0

0

0

0

0

0

0

0

1

1

1

0

0

0

0

0

0

0

0

0

0

0

0

0

0

0

16

13

2

11

0

0

0

0

3

3

0

0

0

0

832

11

0

0

11

1

10

0

0

0

0

0

4

4

4

0

0

0

0

0

578

38

2

36

12

0

12

7

0

0

0

2

5

0

0

31

31

0

442

442

24

24

24

24

0

0

0

0

0

13

5

0

5

0

0

0

8

0

8

57

57

0

16

0

1

40

130

106

93

6

5

1

1

9

9

15

15

39

39

39

1786

1229

881

839

42

348

218

114

3

13

0

0

0

0

0

0

56

56

0

0

10

38

8

3

3

3

0

0

0

0

0

0

0

0

0

0

204

204

204

0

0

0

0

0

252

243

75

0

20

148

0

0

9

9

35

35

35

0

0

0

0

0

7

7

7

0

0

0

0

0

0

0

2

0

0

0

0

0

0

0

0

0

2

2

0

0

2

2

0

0

0

0

0

0

0

0

0

0

0

0

0

0

0

0

0

0

0

0

0

0

0

0

0

0

0

0

0

0

0

0

0

0

0

0

0

0

0

0

6

0

0

0

0

6

6

6

6

0

0

0

0

0

0

0

0

0

0

0

0

0

0

0

14594

14594

14594

9209

9209

5385

0

9

5376

1749

2

2

2

2

0

0

0

0

1705

1683

24

0

18

0

6

0

0

0

33

0

12

14

7

0

0

0

7

7

14

14

4

4

1

0

1

16

1

6

9

0

7

7

0

4

0

4

2

0

2

505

240

1

47

95

122

0

0

0

0

0

41

41

0

0

8

8

5

5

969

969

15

15

6

6

0

0

0

19

0

4

15

3

3

22

22

22

27

27

27

0

5

22

0

4

4

4

4

11

0

0

0

0

0

11

8

8

3

3

0

0

0

0

0

0

0

0

0

0

0

0

0

0

0

0

0

0

0

0

0

0

0

0

0

0

0

0

0

0

0

0

0

0

0

0

0

0

0

0

16

16

0

0

0

16

16

3

13

105

50

47

47

47

3

3

3

0

0

0

0

0

0

0

0

55

0

0

0

55

55

55

0

0

0

0

0

0

0

0

0

0

0

0

10840

91

91

91

36

0

54

0

0

0

1

8911

8911

259

156

103

506

474

0

32

376

10

32

7

88

4

27

24

14

170

1

1

4936

223

933

0

1492

278

3

587

1016

395

9

0

1039

1039

239

17

4

0

214

4

0

119

119

252

205

0

47

39

39

1097

18

36

55

988

0

48

48

0

0

0

0

0

1838

1791

0

0

52

1

51

4

2

2

1664

2

0

1039

623

23

19

4

0

0

0

48

4

44

0

0

0

0

0

9

9

9

38

0

0

2

1

1

20

4

0

0

16

0

0

0

0

16

0

2

1

0

11

2

0

0

0

0

0

0

0

0

153

153

83

83

83

5

5

5

65

65

65

248

248

248

248

248

7

7

7

7

7
