# Supplementary material for: The effects of genital myiasis on the diversity of the vaginal microbiota in female Bactrian camels
Source: BMC Vet Res. 2022 Mar 5;18:87. doi: 10.1186/s12917-022-03189-5 (PMC8897907; doi:10.1186/s12917-022-03189-5)
Supplement: Supplementary file 5 — Additional file 5. [file 12917_2022_3189_MOESM5_ESM.zip › MPL201709200_16s_yy/Treat1/B10_krona/B08.html]

Javascript must be enabled to view this page.

members
magnitude
magnitudeUnassigned

B08

46294

46294

11

11

11

11

11

9

9

9

9

9

743

10

10

10

10

369

369

369

369

364

13

13

13

10

10

10

111

33

33

78

78

95

95

95

33

33

33

102

102

102

29

29

29

3

3

0

0

0

26

26

1591

51

51

51

18

0

1

32

24

2

0

0

2

2

22

0

0

22

22

5

5

5

5

1426

42

42

42

1384

0

0

0

9

9

415

0

0

41

374

0

4

4

32

32

9

9

0

0

9

1

0

8

0

79

18

61

0

18

1

17

187

10

93

27

3

54

26

26

23

23

74

37

9

20

8

0

0

0

35

9

26

36

36

0

0

0

11

11

0

0

226

0

209

17

154

154

37

29

0

8

0

0

85

85

9

9

30

30

46

46

6

0

0

0

0

6

6

6

6

26

26

26

26

26

2

2

2

2

2

621

81

81

0

0

0

0

32

32

49

49

59

59

59

59

0

15

15

15

15

354

318

11

11

293

293

14

14

36

36

36

11

11

11

11

74

14

14

14

60

10

10

50

50

0

0

0

0

10

10

10

10

17

17

17

17

4563

4563

4563

2307

2307

2256

2211

0

45

25

25

25

25

25

0

0

0

0

0

0

0

0

0

0

0

0

0

0

0

0

19

0

0

0

0

19

19

19

19

0

0

0

20

20

20

20

20

10

10

10

9

9

1

1

384

0

0

0

0

315

190

190

190

125

125

125

0

0

0

0

69

0

0

0

69

69

69

103

48

48

48

48

55

0

0

0

11

11

11

0

0

0

0

44

44

44

0

0

0

0

17413

967

36

36

0

36

9

9

9

0

0

0

0

0

0

14

14

14

0

0

0

0

0

11

11

11

0

0

291

21

21

11

11

174

174

0

0

38

0

38

47

47

0

0

0

0

0

0

0

0

0

606

606

10

494

102

1463

1463

9

0

9

1454

1410

44

6808

15

15

15

0

0

0

1563

723

617

106

840

74

123

640

3

0

0

0

4

0

0

0

0

4

4

2864

2864

2860

4

0

0

0

0

0

0

43

43

43

1406

1406

0

1406

0

0

0

0

0

0

0

897

808

0

0

808

0

0

0

89

89

16

0

0

16

16

3938

223

223

223

0

0

0

0

0

0

0

3272

0

0

1728

0

356

0

94

0

11

11

1253

3

26

26

380

0

9

118

231

7

15

1138

27

0

1111

56

56

56

79

79

79

17

17

17

3

3

3

7

7

7

281

281

42

0

0

77

88

74

4237

15

15

15

516

471

19

329

65

28

30

3

3

42

42

30

13

0

5

4

4

17

17

323

323

0

252

6

57

8

47

47

47

101

27

0

27

65

0

0

65

9

9

7

7

7

0

0

3198

78

78

0

311

213

98

0

0

2459

2459

58

35

14

0

2

7

0

0

14

0

14

51

51

162

0

162

65

65

0

0

0

200

0

0

0

0

25

25

25

25

45

45

45

45

9

9

9

9

52

0

0

0

35

14

14

21

21

0

0

0

0

9

9

9

8

8

8

0

0

0

0

0

0

0

0

0

0

0

0

13

13

13

13

18

18

18

18

0

0

0

12

12

12

12

26

26

26

26

75

39

39

39

39

12

12

12

12

0

0

8

8

0

0

8

8

16

0

0

0

16

16

16

678

449

449

449

449

13

13

13

13

216

216

1

1

215

215

10

0

0

0

0

10

10

10

10

0

0

0

0

115

115

115

115

18

97

0

0

0

4591

3676

3676

8

8

1349

5

1335

9

892

892

0

0

639

0

0

639

74

74

0

68

0

50

7

11

0

0

0

0

495

85

0

379

31

17

17

35

35

99

99

0

0

0

0

0

0

0

0

239

239

20

0

20

0

0

0

219

0

39

180

640

640

0

0

640

13

0

627

36

36

36

5

31

0

0

0

0

0

0

0

14805

139

139

139

0

0

16

100

0

0

23

11337

21

21

21

11316

1947

1167

75

193

0

465

18

8

16

0

5

0

190

123

0

67

132

0

132

0

1805

1805

2

2

80

0

63

5

11

1

0

418

4

414

160

160

360

360

13

13

5269

4755

211

286

2

15

934

6

88

1

18

571

0

38

197

15

6

6

3329

32

32

32

633

0

0

0

0

0

15

15

51

51

0

0

0

0

0

0

2

2

561

11

61

348

141

4

4

2648

27

17

10

2

2

0

0

508

136

6

364

2

10

10

55

0

47

8

2035

2027

8

11

11

16

16

9

7

1

1

1

1

1

0

0

0

0

0

0

0

244

244

6

6

6

24

24

24

214

214

214
