# Supplementary material for: The effects of genital myiasis on the diversity of the vaginal microbiota in female Bactrian camels
Source: BMC Vet Res. 2022 Mar 5;18:87. doi: 10.1186/s12917-022-03189-5 (PMC8897907; doi:10.1186/s12917-022-03189-5)
Supplement: Supplementary file 5 — Additional file 5. [file 12917_2022_3189_MOESM5_ESM.zip › MPL201709200_16s_yy/Treat1/B10_krona/B09.html]

Javascript must be enabled to view this page.

members
magnitude
magnitudeUnassigned

B09

46095

46095

77

2

2

2

2

2

2

2

0

2

1

1

1

1

0

0

0

0

0

0

0

0

2

2

2

2

40

40

4

4

22

22

0

0

14

14

21

5

5

5

16

0

0

15

15

1

1

9

7

0

0

7

7

2

2

2

4

4

4

0

0

4

4

0

0

0

2

2

2

0

0

2

2

0

0

0

0

0

0

0

0

0

0

106

0

0

0

0

7

7

7

7

0

0

0

99

10

10

10

89

89

89

0

0

0

0

17

17

17

17

17

0

0

0

3

0

0

0

0

3

3

3

3

0

0

0

0

0

39

3

3

3

3

0

0

0

0

0

0

0

20

20

1

1

19

19

16

16

3

3

13

13

23

1

1

1

1

0

0

0

0

0

0

0

1

1

1

1

0

0

0

0

0

0

0

0

0

0

0

0

1

1

1

1

2

2

2

2

18

0

0

0

0

0

0

0

0

0

0

0

0

14

14

14

4

4

4

0

0

0

0

0

0

0

0

0

0

0

0

39

39

39

39

39

10930

1929

4

4

4

0

0

0

15

15

15

1775

234

0

177

0

45

6

6

895

0

0

895

0

0

18

18

628

0

0

162

11

0

34

0

2

419

0

0

0

0

0

0

0

0

0

0

0

0

0

5

5

5

130

130

62

0

4

13

44

7

244

244

244

229

15

0

0

0

3051

2

2

2

418

41

41

377

15

14

19

322

7

0

0

263

263

1

30

0

232

0

12

8

8

4

4

0

0

0

1

1

1

32

2

2

23

23

0

0

7

0

7

2323

31

31

74

14

60

0

0

0

0

0

60

0

60

194

70

124

1892

1892

69

69

3

0

1

0

0

2

0

0

0

0

0

0

0

0

0

0

112

58

2

2

36

24

12

15

15

3

3

0

0

2

2

0

0

0

0

0

0

0

0

2

2

2

0

0

0

0

0

0

0

0

0

0

0

0

0

34

34

0

5

29

1

1

1

0

0

8

8

8

9

9

9

5594

693

35

35

658

0

658

0

0

0

0

0

0

0

2

2

2

0

0

1

0

0

1

1

0

0

41

41

41

0

0

0

801

801

0

0

788

0

13

4

4

4

0

0

0

3909

3798

299

3462

0

37

111

19

92

143

143

143

0

0

0

0

0

0

0

0

0

0

0

0

0

0

0

0

0

0

0

0

0

0

0

0

0

0

0

0

2198

1489

1489

72

72

0

0

51

12

5

34

0

2

2

0

0

0

0

0

0

6

4

0

2

0

1312

1312

0

0

0

0

36

36

0

0

6

6

4

4

0

156

156

141

1

16

124

15

9

6

0

0

0

521

521

521

0

519

2

0

0

17

17

0

0

17

17

0

15

15

15

15

0

0

0

0

3441

3441

3441

798

798

2643

1339

0

1304

13

13

13

13

13

55

41

41

41

41

0

0

0

0

14

14

14

14

0

0

7940

15

0

0

0

0

0

15

0

0

15

15

2

2

2

2

6024

3177

8

0

3

0

0

5

4

4

817

0

0

0

9

808

2

2

0

1651

0

1637

9

5

420

420

0

0

28

28

5

2

3

149

0

135

14

0

0

0

0

0

0

0

0

0

0

0

0

7

7

1

1

0

0

0

0

7

7

10

0

8

2

0

0

0

0

0

0

68

1

22

0

4

41

2847

2847

2847

4

4

2

2

2

2

0

0

1895

1895

1895

1890

0

5

0

0

0

0

0

0

0

0

0

21055

112

112

112

0

0

0

6

86

0

20

16884

2

2

2

16882

137

0

5

130

0

0

2

9800

0

87

2685

6221

196

211

387

13

0

0

0

654

654

0

0

3

3

3065

821

1211

22

0

661

0

17

61

272

1378

1280

98

0

8

8

1314

114

585

36

3

576

0

0

245

245

24

24

20

20

234

34

200

4059

0

0

0

0

32

32

32

3247

194

2

192

1558

2

23

1533

0

0

0

0

5

5

26

0

26

7

7

1457

1049

84

0

324

780

0

0

0

0

0

0

40

9

25

0

6

736

0

0

0

0

736

0

0

0

0

0

4

4

4

4

4

4

4

15

12

0

0

0

12

12

12

0

0

0

0

0

0

0

3

3

3

3

0

0

0

0

0

0

0

0

0

7

7

7

7

7

3

0

0

0

0

3

1

1

1

2

2

2

8

8

8

8

6

2

0

0

0

103

22

22

22

22

47

47

47

47

34

14

14

14

14

14

14

0

0

0

0

0

0

4

1

1

3

3

2

2

2

13

13

11

11

11

2

2

2

0

0

0
