# Supplementary material for: The effects of genital myiasis on the diversity of the vaginal microbiota in female Bactrian camels
Source: BMC Vet Res. 2022 Mar 5;18:87. doi: 10.1186/s12917-022-03189-5 (PMC8897907; doi:10.1186/s12917-022-03189-5)
Supplement: Supplementary file 5 — Additional file 5. [file 12917_2022_3189_MOESM5_ESM.zip › MPL201709200_16s_yy/Treat1/B10_krona/B10.html]

Javascript must be enabled to view this page.

members
magnitude
magnitudeUnassigned

B10

46328

46328

0

0

0

0

0

6

6

0

0

0

6

6

6

3

3

3

3

3

0

0

0

0

0

0

0

0

0

0

5176

0

0

0

0

0

0

0

0

0

0

2012

9

0

0

0

0

5

0

1

0

0

4

0

0

4

3

0

1

0

0

0

0

0

0

0

0

2003

0

0

0

0

0

0

1981

3

1623

10

345

19

0

0

19

0

0

3

0

3

0

0

0

0

0

0

0

0

0

0

3164

3164

0

0

5

0

5

0

0

7

7

365

0

0

365

1186

1186

0

0

537

537

9

0

9

0

0

0

0

0

0

0

0

0

0

0

0

0

0

3

0

0

0

3

0

1052

0

382

34

0

323

224

8

1

79

0

1

0

0

0

0

0

0

0

0

0

0

0

0

0

0

0

0

0

0

0

0

0

0

0

0

0

0

0

0

0

0

0

0

0

0

0

0

0

1

1

1

1

1

5

0

0

0

0

0

0

0

0

0

0

0

0

0

0

0

0

0

0

0

0

0

0

0

0

0

0

5

5

5

5

0

0

0

0

0

0

0

0

0

0

0

0

0

0

0

0

0

0

0

0

0

0

0

0

0

17428

17428

17428

14359

14359

3069

3065

4

0

0

0

0

0

0

18648

43

0

0

0

0

0

0

0

0

0

0

0

3

3

3

0

0

0

0

0

0

0

0

0

0

0

0

0

0

0

13

13

13

0

0

27

27

27

0

0

0

0

0

0

0

0

0

0

0

0

0

0

0

0

0

874

0

0

0

78

78

78

0

0

0

0

0

0

0

0

0

0

0

0

0

0

0

0

1

1

1

55

55

31

1

0

23

0

0

740

0

0

1

1

281

0

238

43

114

0

0

0

21

0

93

344

26

0

0

214

0

72

0

14

18

15813

15813

15813

15813

0

0

0

0

1082

1

0

0

0

0

1

1

1

1

0

1

0

0

0

0

0

0

0

0

0

5

5

5

0

0

0

0

0

0

0

0

0

0

0

0

0

0

0

0

0

905

597

10

587

308

0

285

22

1

170

27

27

143

0

0

0

0

140

3

0

0

0

0

0

836

0

0

0

102

6

6

0

0

96

9

77

10

0

0

2

0

0

0

0

0

2

2

0

0

584

0

0

15

15

0

0

9

0

9

31

1

0

21

0

9

450

450

49

15

34

0

0

0

14

14

16

13

3

83

17

0

0

0

17

66

66

64

64

0

57

7

0

0

1

1

1

0

0

0

0

0

0

0

0

0

0

0

0

0

0

0

0

0

0

0

0

2

2

2

2

2

0

0

0

0

0

0

0

0

0

0

0

0

0

0

0

0

0

0

0

0

0

0

0

0

0

0

0

0

0

0

35

32

0

0

0

0

0

0

32

32

32

0

0

0

0

0

0

0

0

0

0

0

0

0

0

0

0

0

0

0

0

0

0

0

0

0

0

0

0

0

0

0

0

0

0

0

0

3

3

3

3

0

0

0

0

0

0

0

0

0

0

0

0

0

0

0

0

0

0

0

0

0

0

0

0

52

11

0

0

0

11

11

11

0

0

0

0

0

0

0

0

41

0

0

0

41

41

41

0

0

0

0

0

0

0

3703

0

0

0

0

6

6

6

4

0

0

2

0

0

0

0

0

0

0

0

3695

3

3

3

3692

1626

2

2

0

1622

3

0

3

2

2

0

0

0

0

0

0

5

5

47

0

44

3

16

0

0

0

16

0

0

0

0

1606

532

8

79

987

0

0

0

0

33

0

33

0

0

0

0

0

0

0

12

12

0

0

0

0

0

2

2

0

0

0

0

314

314

0

0

26

0

0

16

6

4

2

0

0

0

0

0

2

2

2

0

0

8

8

8

8

8

1139

3

3

3

3

0

0

0

936

936

0

0

0

0

1

1

3

3

0

0

0

0

0

0

0

0

0

1

0

1

0

0

0

0

0

5

5

0

0

926

0

0

926

0

0

0

0

146

146

0

0

146

146

0

0

0

0

0

0

0

0

0

0

54

54

27

27

0

0

0

0

27

0

0

27

2

0

0

0

0

0

0

0

0

2

2

2

2

3

0

0

0

0

0

0

0

0

0

0

0

3

3

3

3

0

0

0

0

0

0

0

0

32

32

0

0

0

32

32

0

32

6

6

6

6

6

0

0

0

0

0

0

0

0

0

0

2

2

2

2

2

0

0

77

77

12

12

8

4

0

0

0

65

65

65

0

0

0

0

0

0

0

0

0

0

0
