# Supplementary figures and images for: The effects of genital myiasis on the diversity of the vaginal microbiota in female Bactrian camels
Source: BMC Vet Res. 2022 Mar 5;18:87. doi: 10.1186/s12917-022-03189-5 (PMC8897907; doi:10.1186/s12917-022-03189-5)

Length Distribution

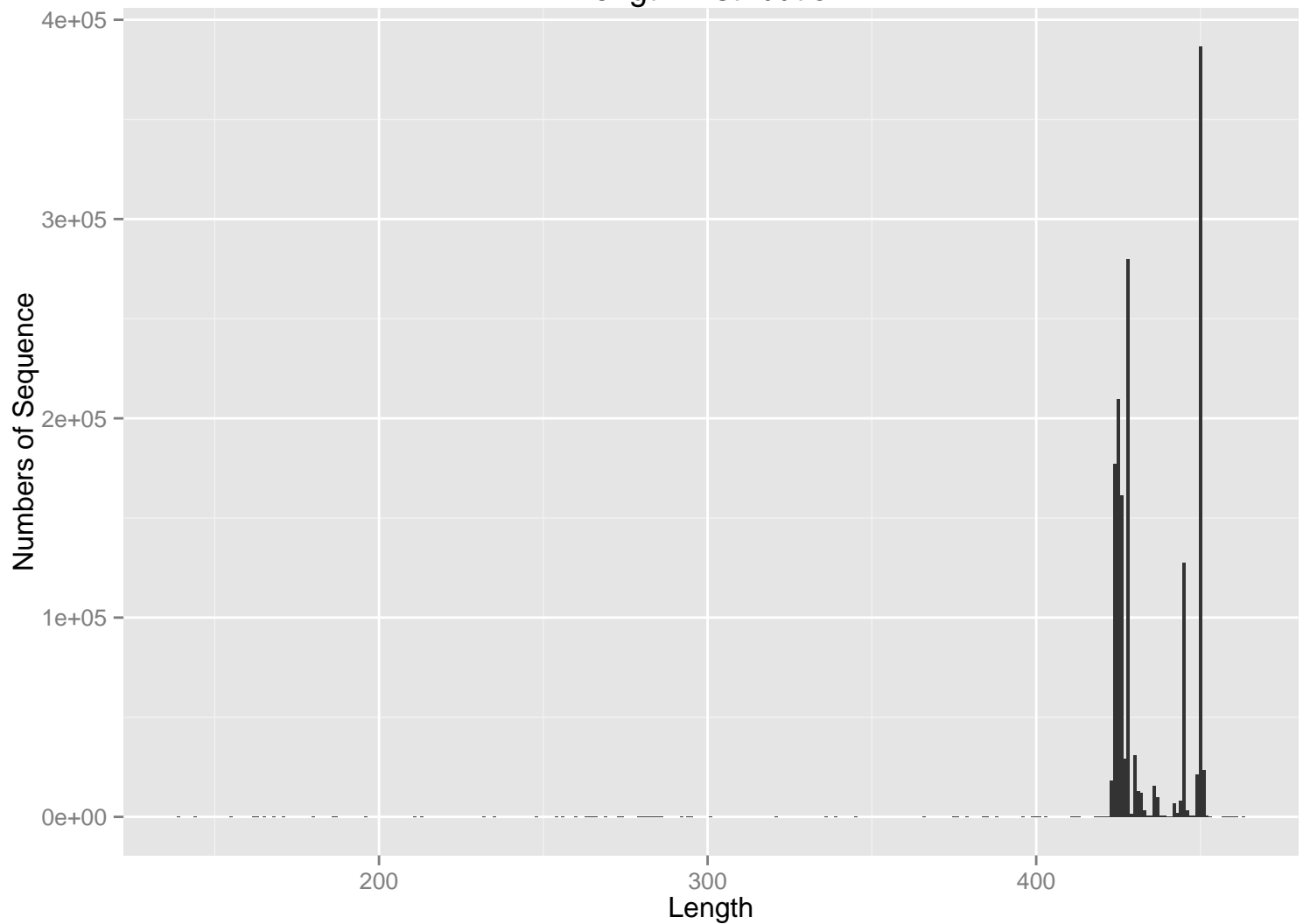

Supplement: Supplementary file 5 — Additional file 5. [file 12917_2022_3189_MOESM5_ESM.zip › MPL201709200_16s_yy/A02_sequences/Length_Distribution.pdf]

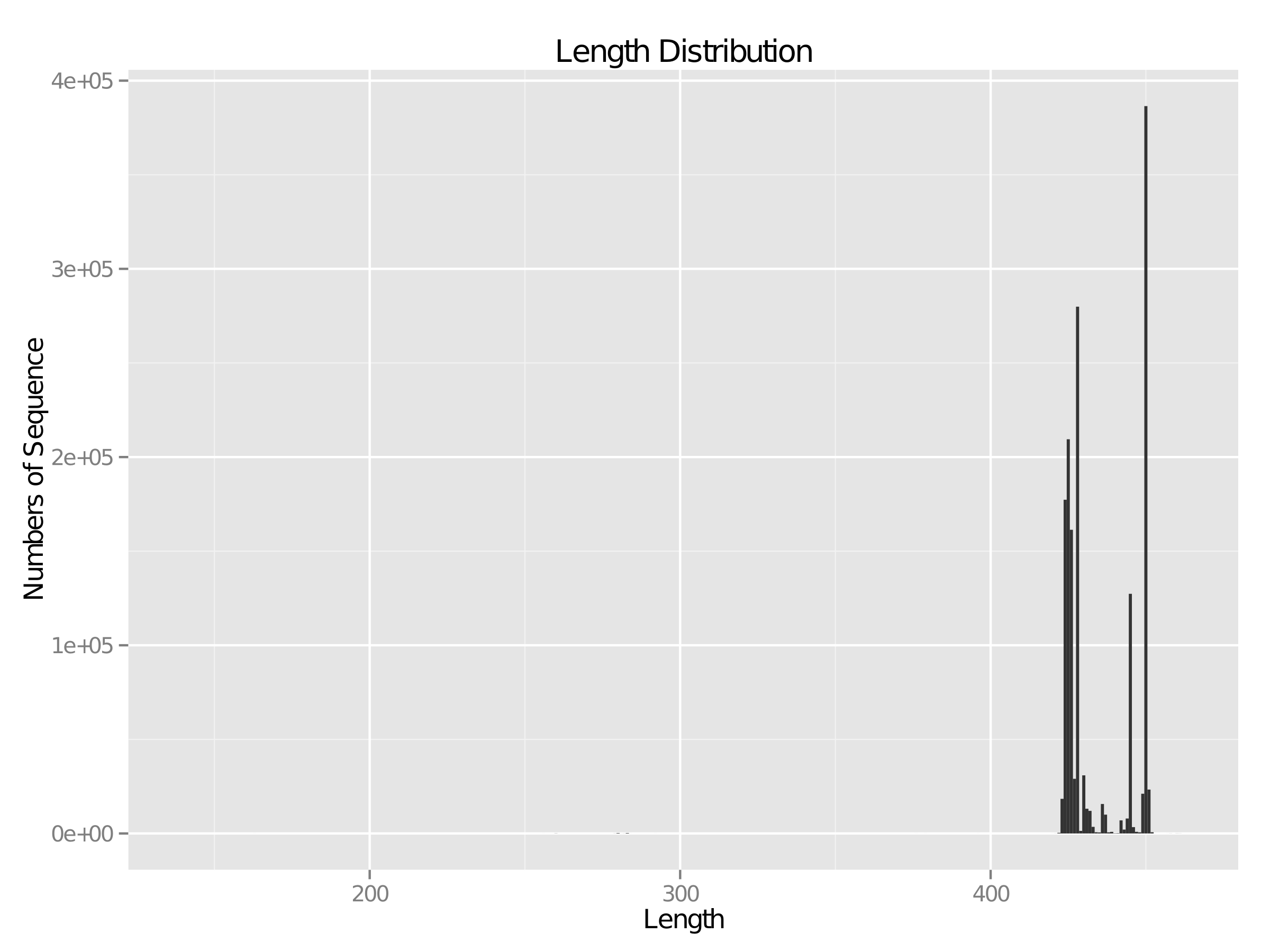

Supplement: Supplementary file 5 — Additional file 5. [file 12917_2022_3189_MOESM5_ESM.zip › MPL201709200_16s_yy/A02_sequences/length_distribution.png]

The Number of OTUs

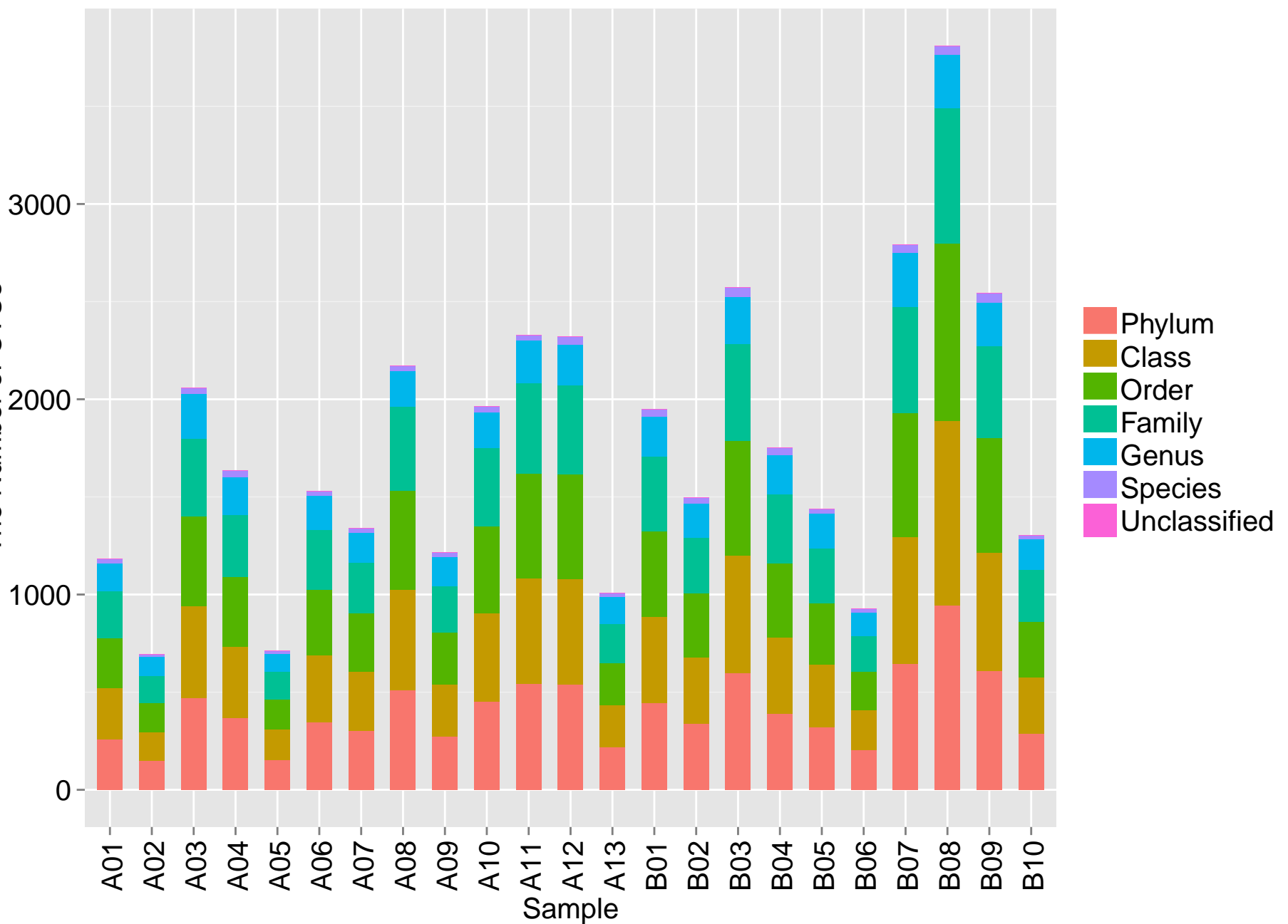

Supplement: Supplementary file 5 — Additional file 5. [file 12917_2022_3189_MOESM5_ESM.zip › MPL201709200_16s_yy/Treat1/B01_OTU/OTU_count.pdf]

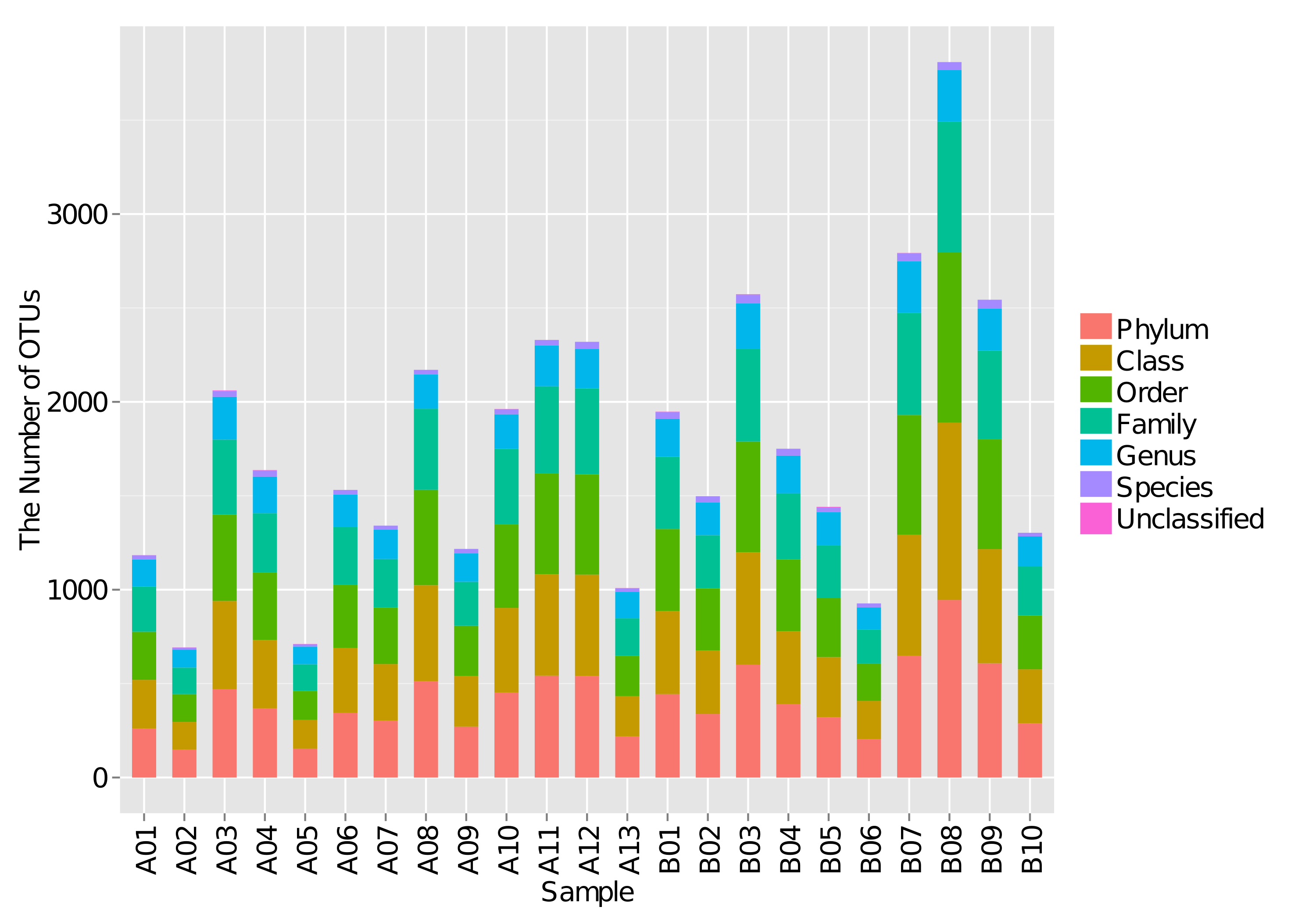

Supplement: Supplementary file 5 — Additional file 5. [file 12917_2022_3189_MOESM5_ESM.zip › MPL201709200_16s_yy/Treat1/B01_OTU/OTU_count.png]

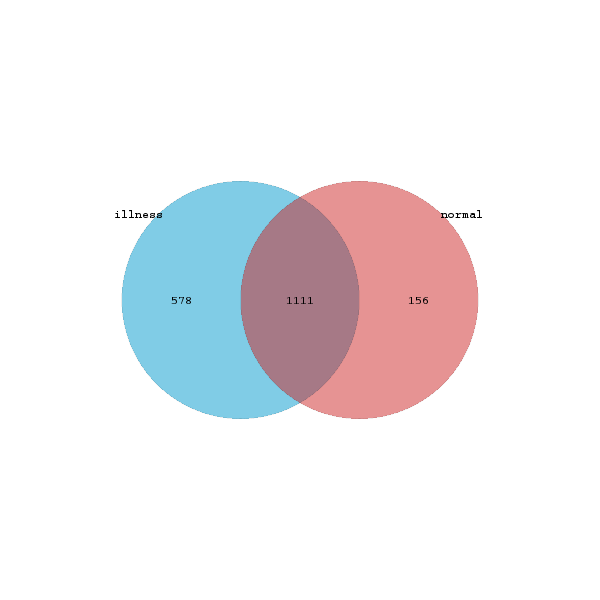

Supplement: Supplementary file 5 — Additional file 5. [file 12917_2022_3189_MOESM5_ESM.zip › MPL201709200_16s_yy/Treat1/B01_OTU/venn/venn.illness-normal.png]

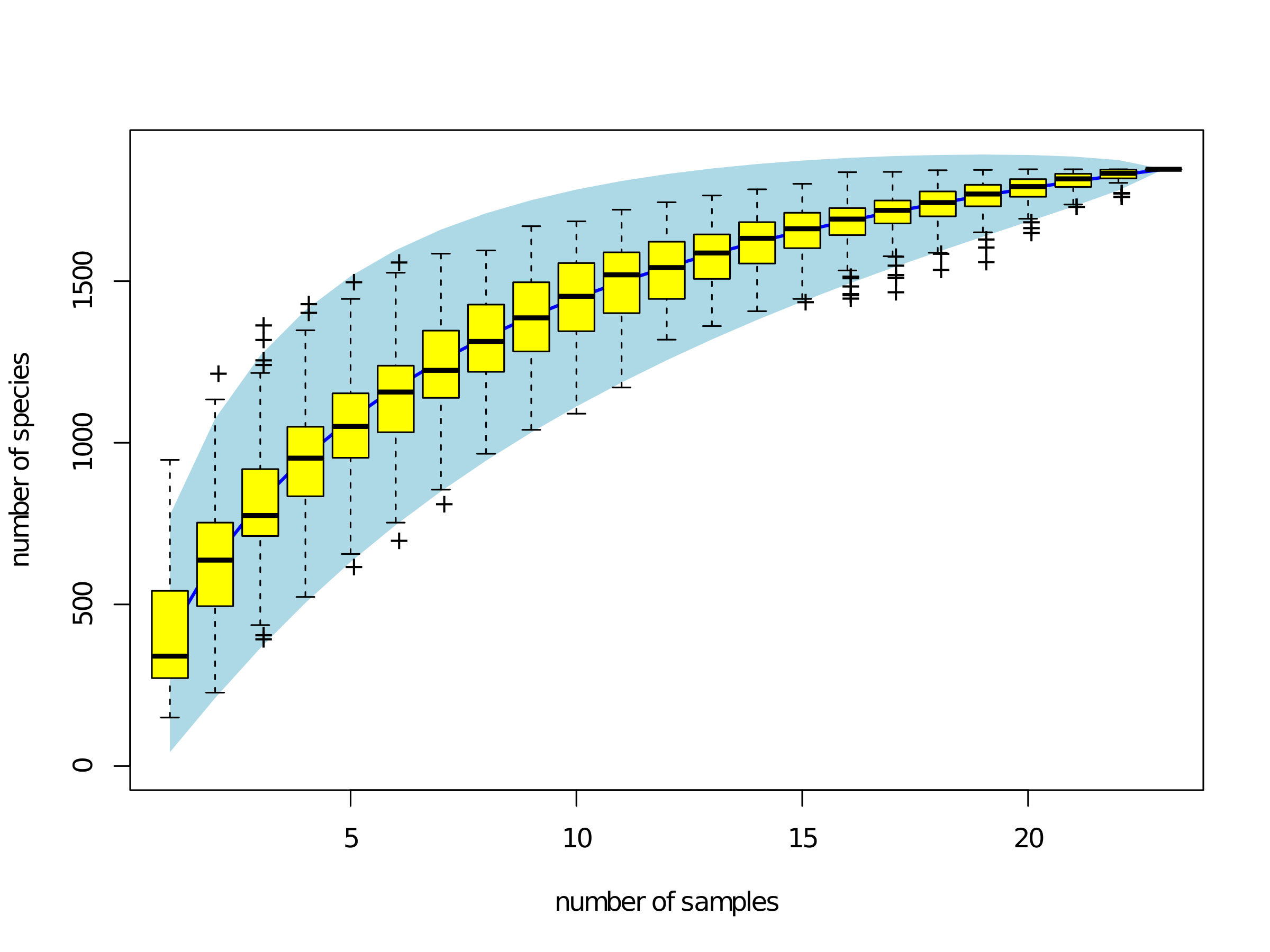

Supplement: Supplementary file 5 — Additional file 5. [file 12917_2022_3189_MOESM5_ESM.zip › MPL201709200_16s_yy/Treat1/B03_specaccum/specaccum.png]

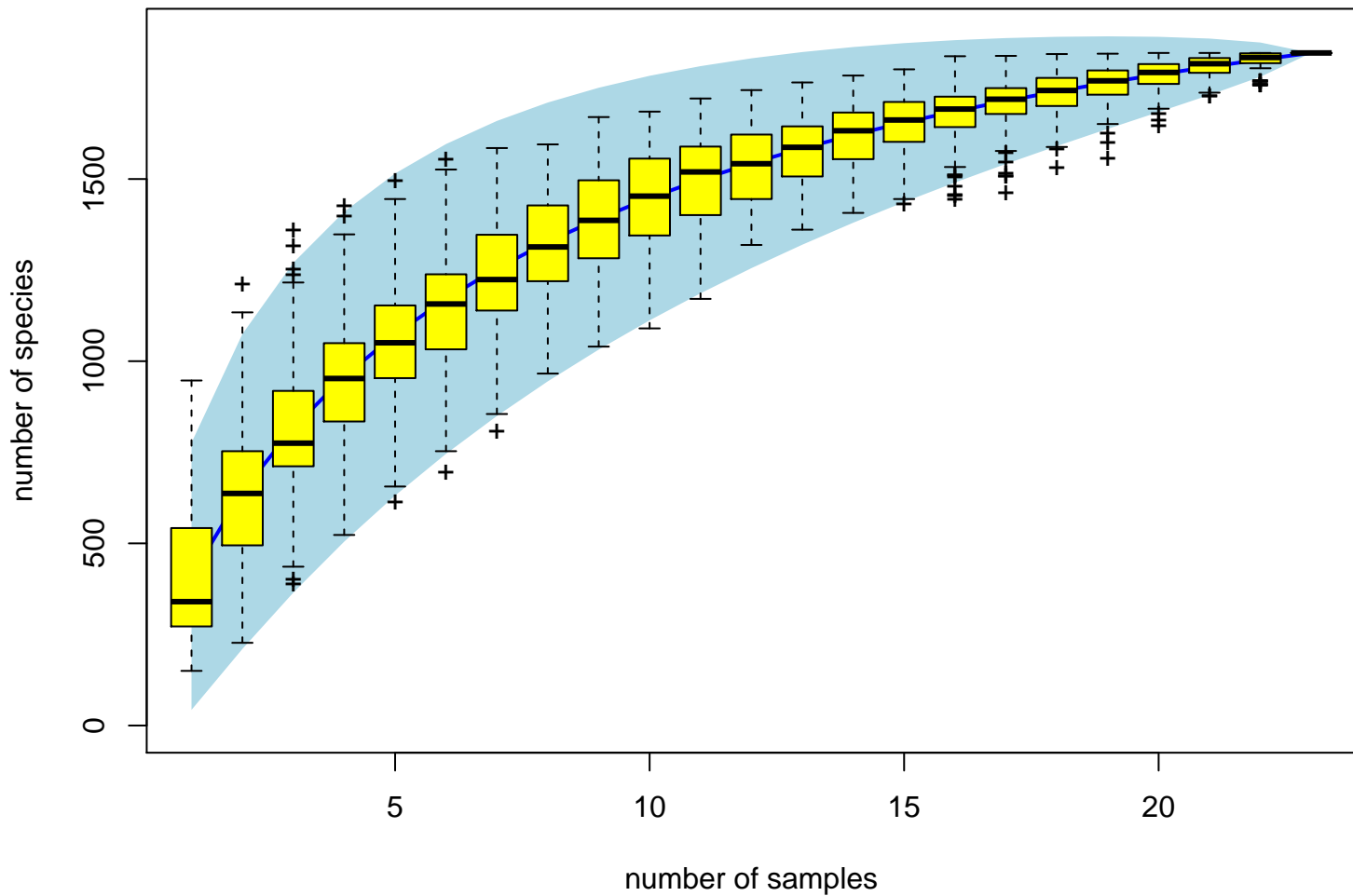

Supplement: Supplementary file 5 — Additional file 5. [file 12917_2022_3189_MOESM5_ESM.zip › MPL201709200_16s_yy/Treat1/B03_specaccum/species_curve.pdf]

Rank Abundance Curve

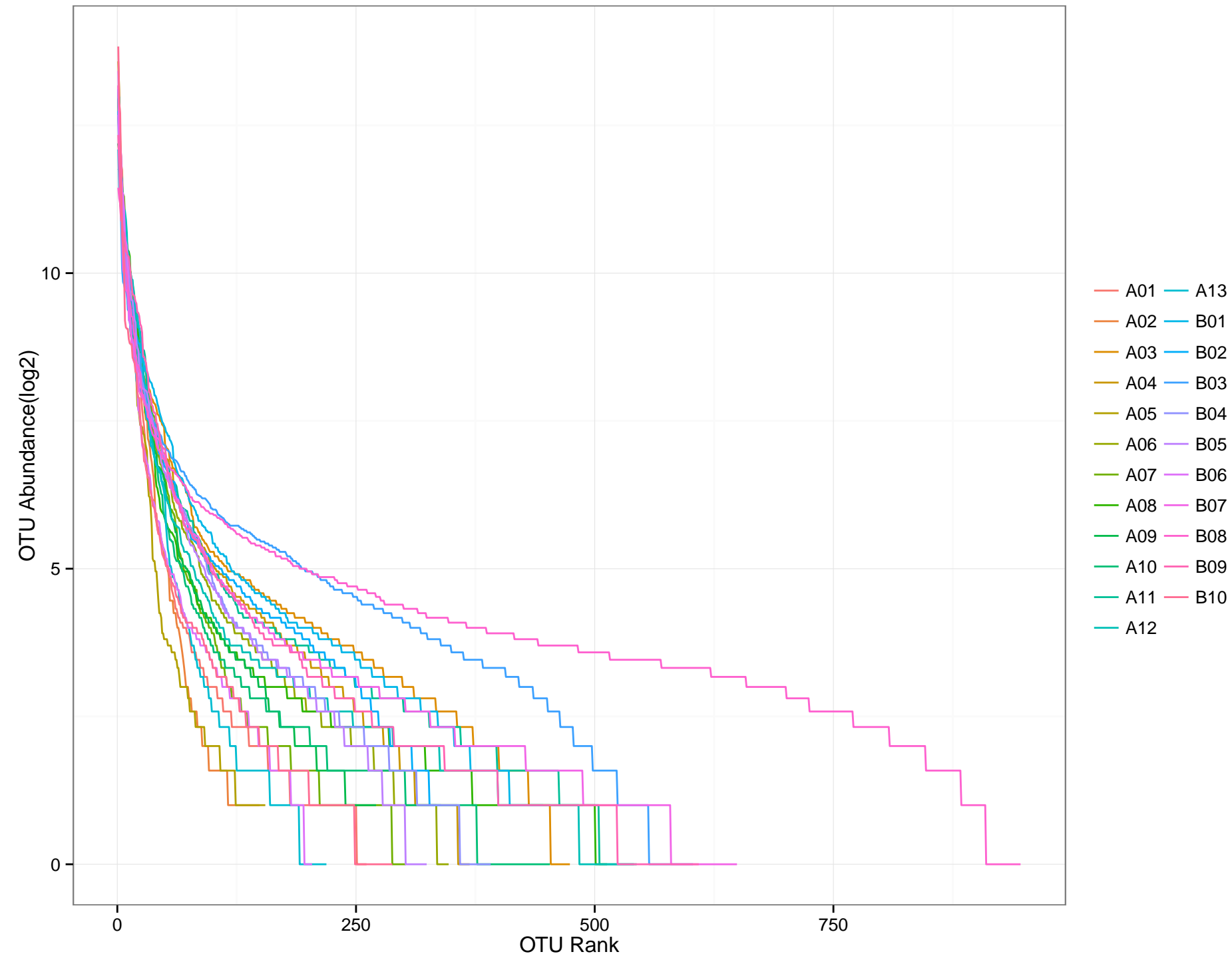

Supplement: Supplementary file 5 — Additional file 5. [file 12917_2022_3189_MOESM5_ESM.zip › MPL201709200_16s_yy/Treat1/B04_rabund/rabund.pdf]

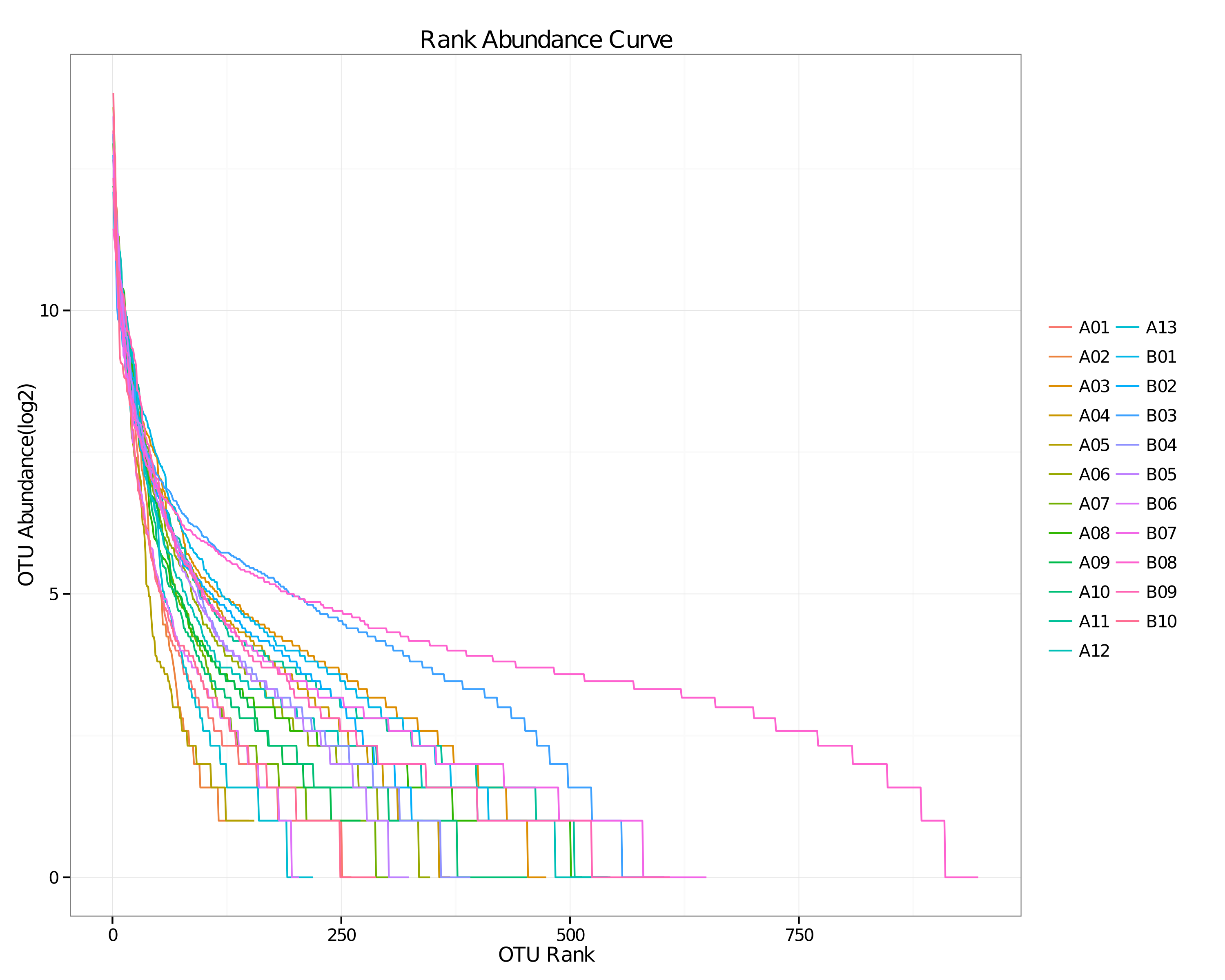

Supplement: Supplementary file 5 — Additional file 5. [file 12917_2022_3189_MOESM5_ESM.zip › MPL201709200_16s_yy/Treat1/B04_rabund/rabund.png]

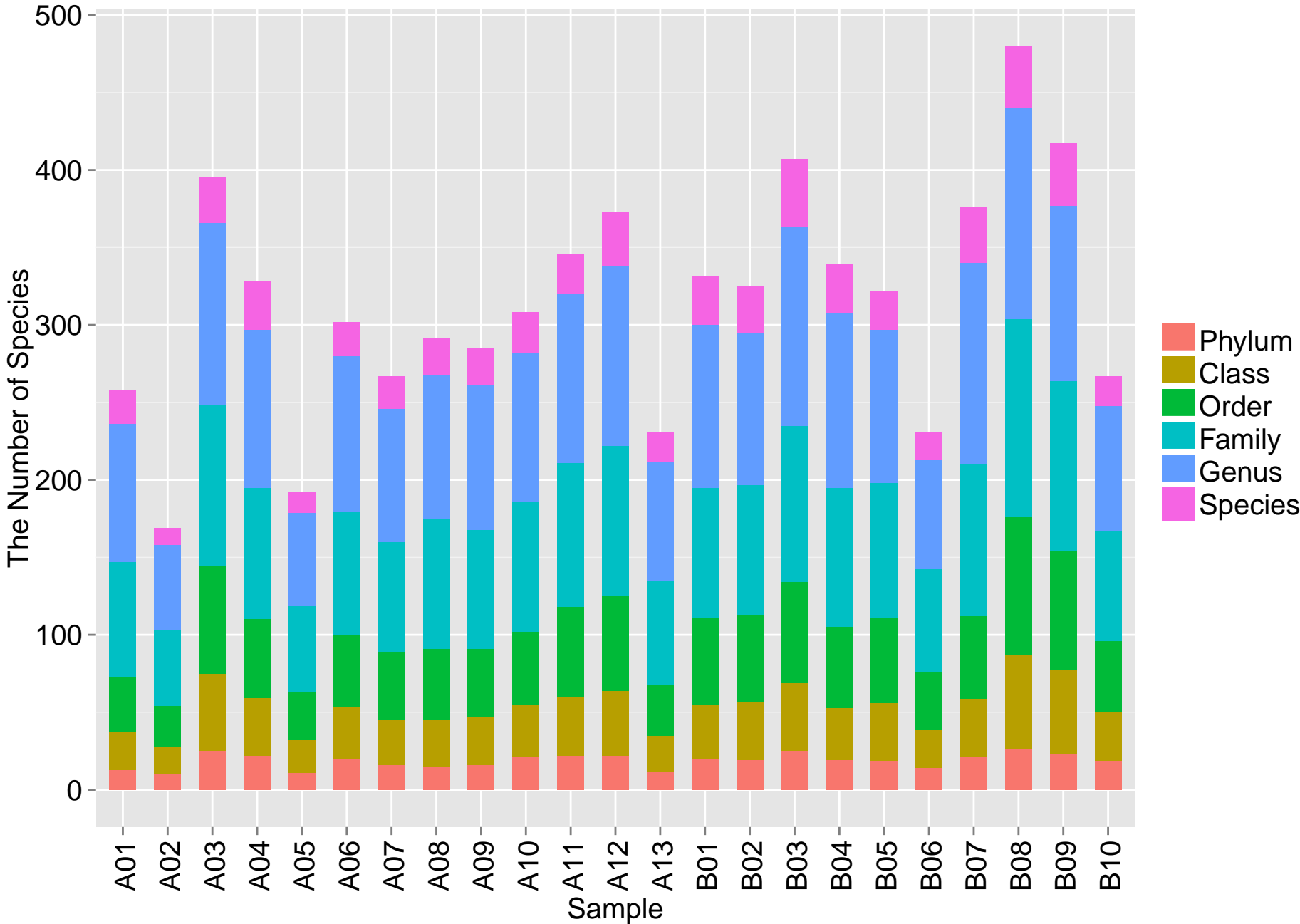

Supplement: Supplementary file 5 — Additional file 5. [file 12917_2022_3189_MOESM5_ESM.zip › MPL201709200_16s_yy/Treat1/B06_taxa/species_count.pdf]

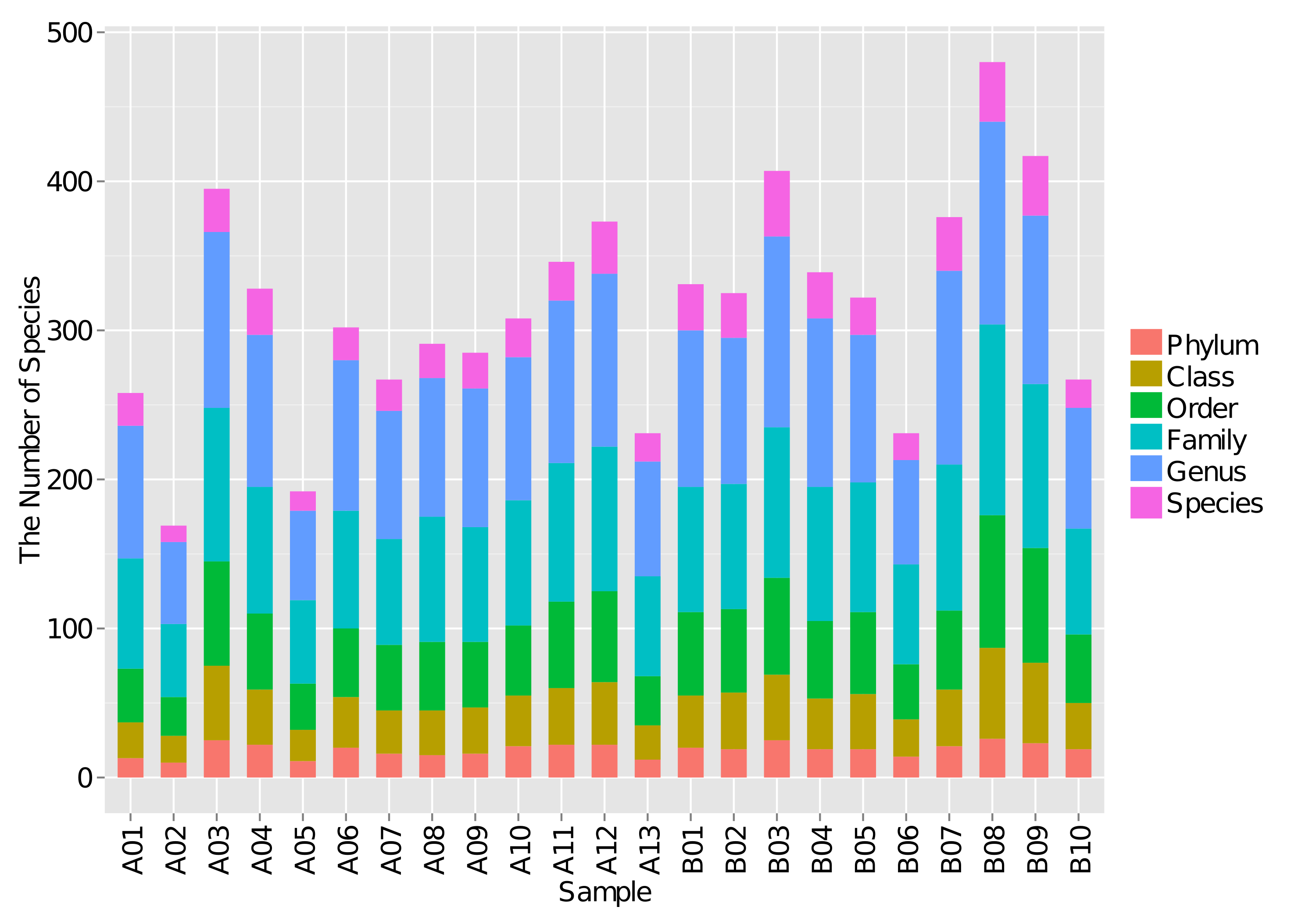

Supplement: Supplementary file 5 — Additional file 5. [file 12917_2022_3189_MOESM5_ESM.zip › MPL201709200_16s_yy/Treat1/B06_taxa/species_count.png]

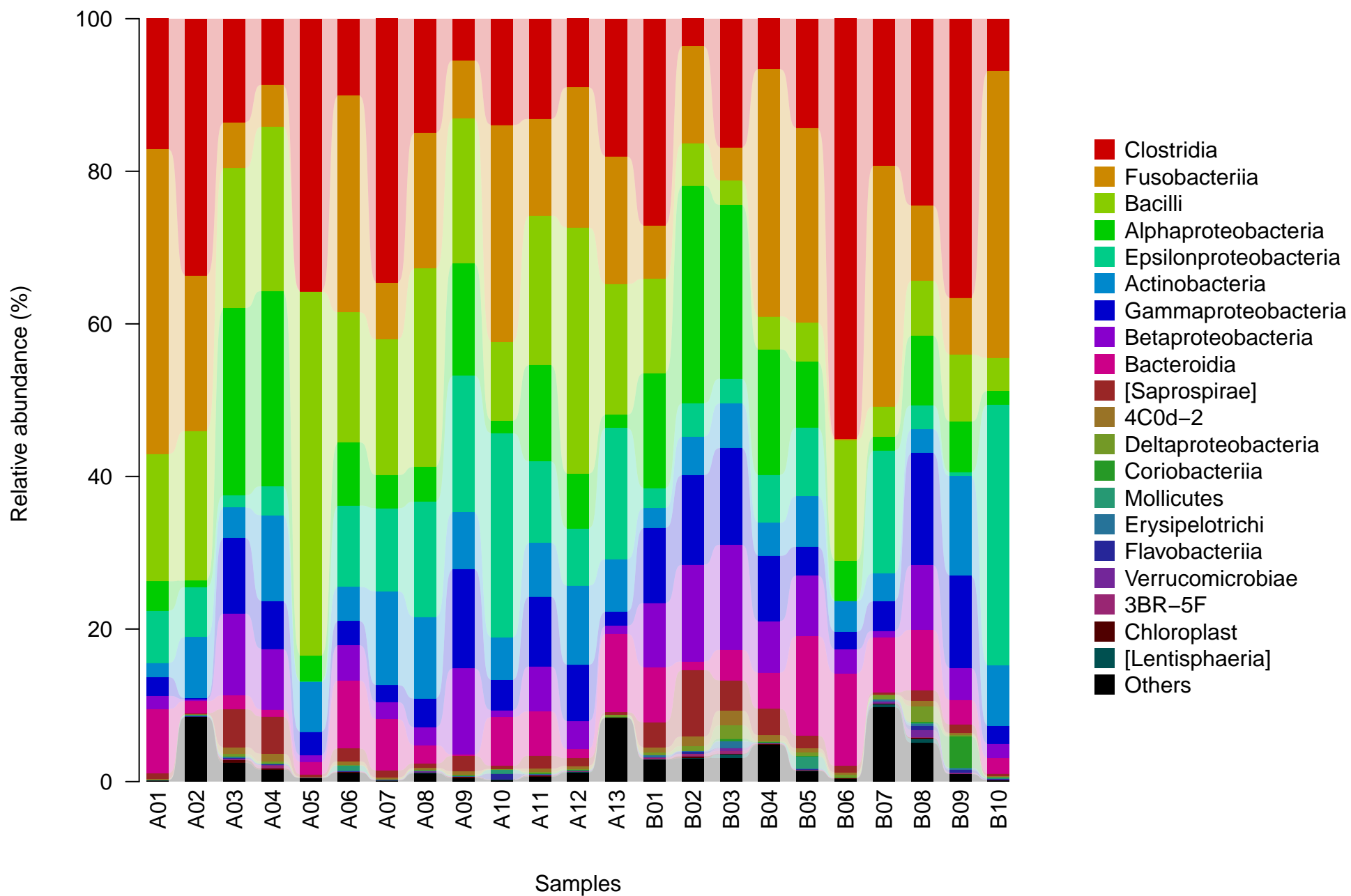

Supplement: Supplementary file 5 — Additional file 5. [file 12917_2022_3189_MOESM5_ESM.zip › MPL201709200_16s_yy/Treat1/B07_taxa_summary/bar_class.pdf]

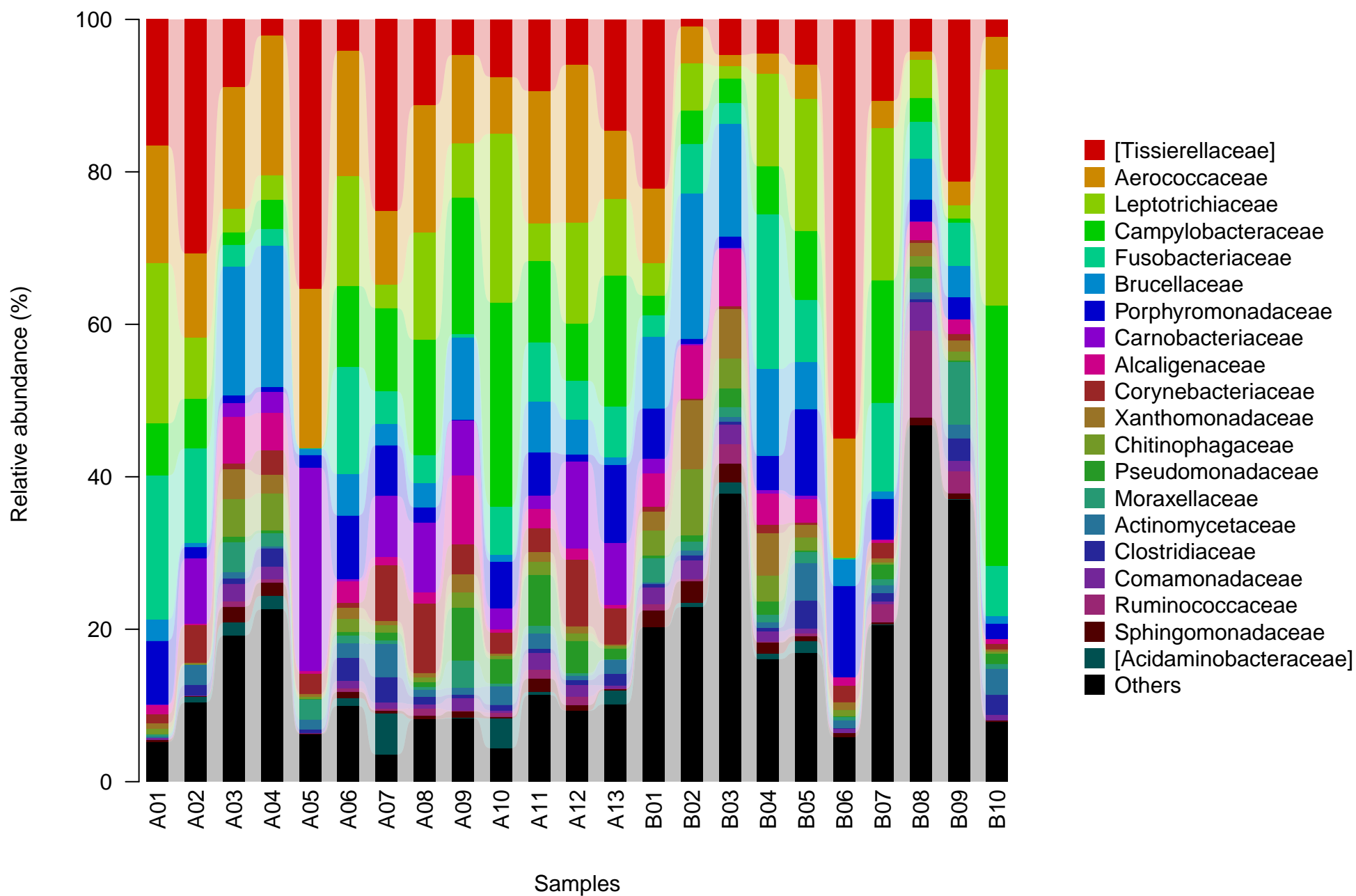

Supplement: Supplementary file 5 — Additional file 5. [file 12917_2022_3189_MOESM5_ESM.zip › MPL201709200_16s_yy/Treat1/B07_taxa_summary/bar_family.pdf]

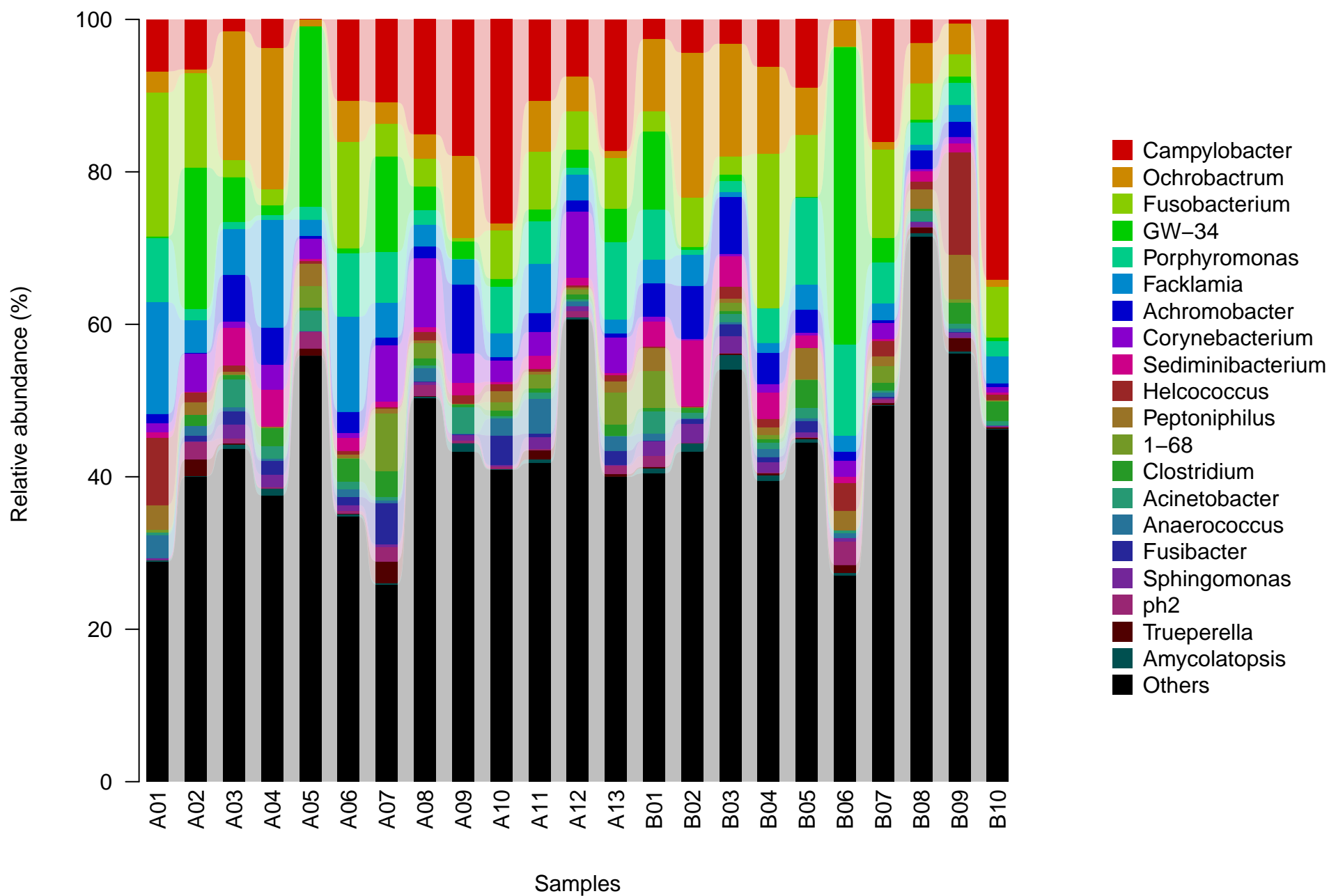

Supplement: Supplementary file 5 — Additional file 5. [file 12917_2022_3189_MOESM5_ESM.zip › MPL201709200_16s_yy/Treat1/B07_taxa_summary/bar_genus.pdf]

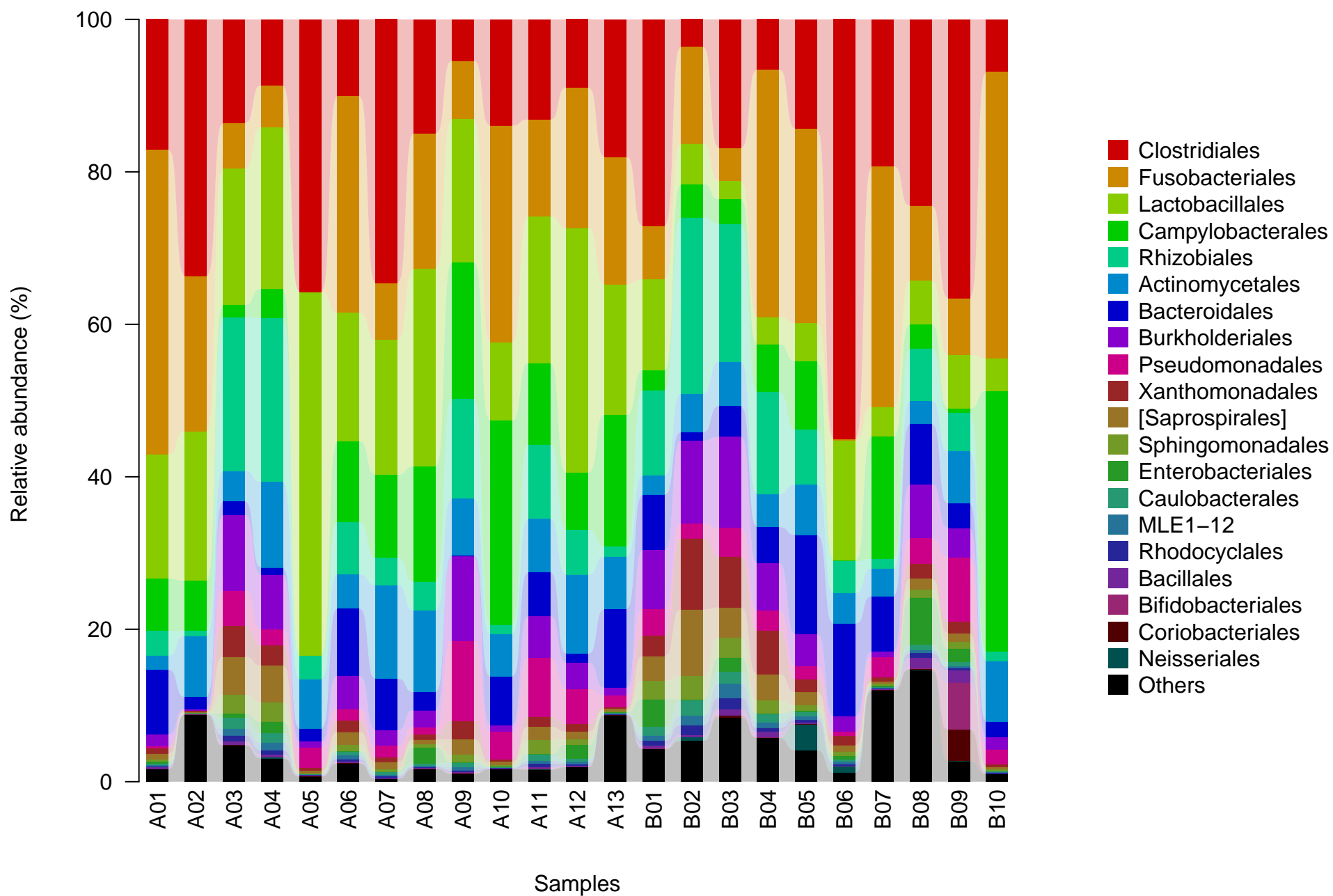

Supplement: Supplementary file 5 — Additional file 5. [file 12917_2022_3189_MOESM5_ESM.zip › MPL201709200_16s_yy/Treat1/B07_taxa_summary/bar_order.pdf]

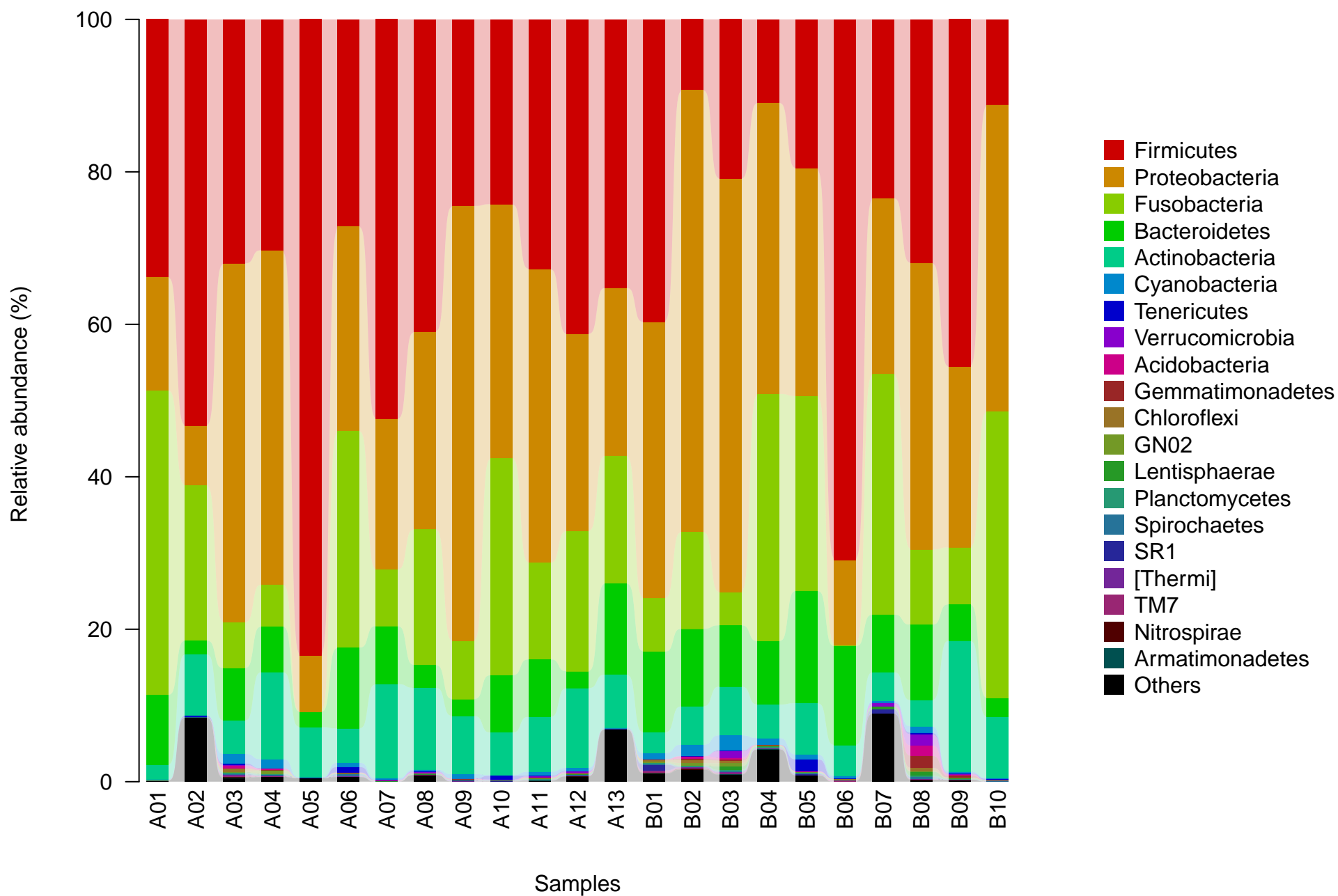

Supplement: Supplementary file 5 — Additional file 5. [file 12917_2022_3189_MOESM5_ESM.zip › MPL201709200_16s_yy/Treat1/B07_taxa_summary/bar_phylum.pdf]

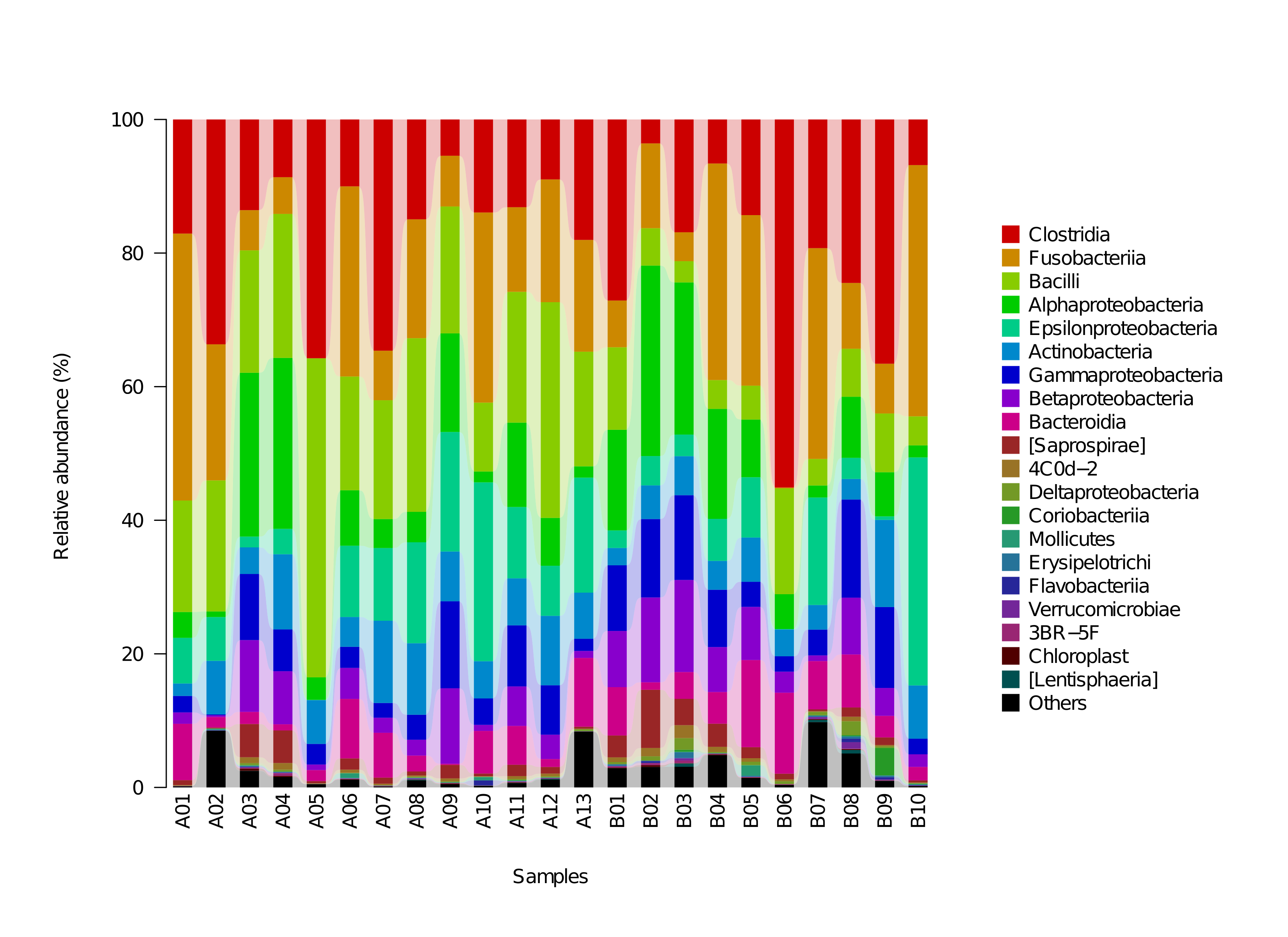

Supplement: Supplementary file 5 — Additional file 5. [file 12917_2022_3189_MOESM5_ESM.zip › MPL201709200_16s_yy/Treat1/B07_taxa_summary/class.png]

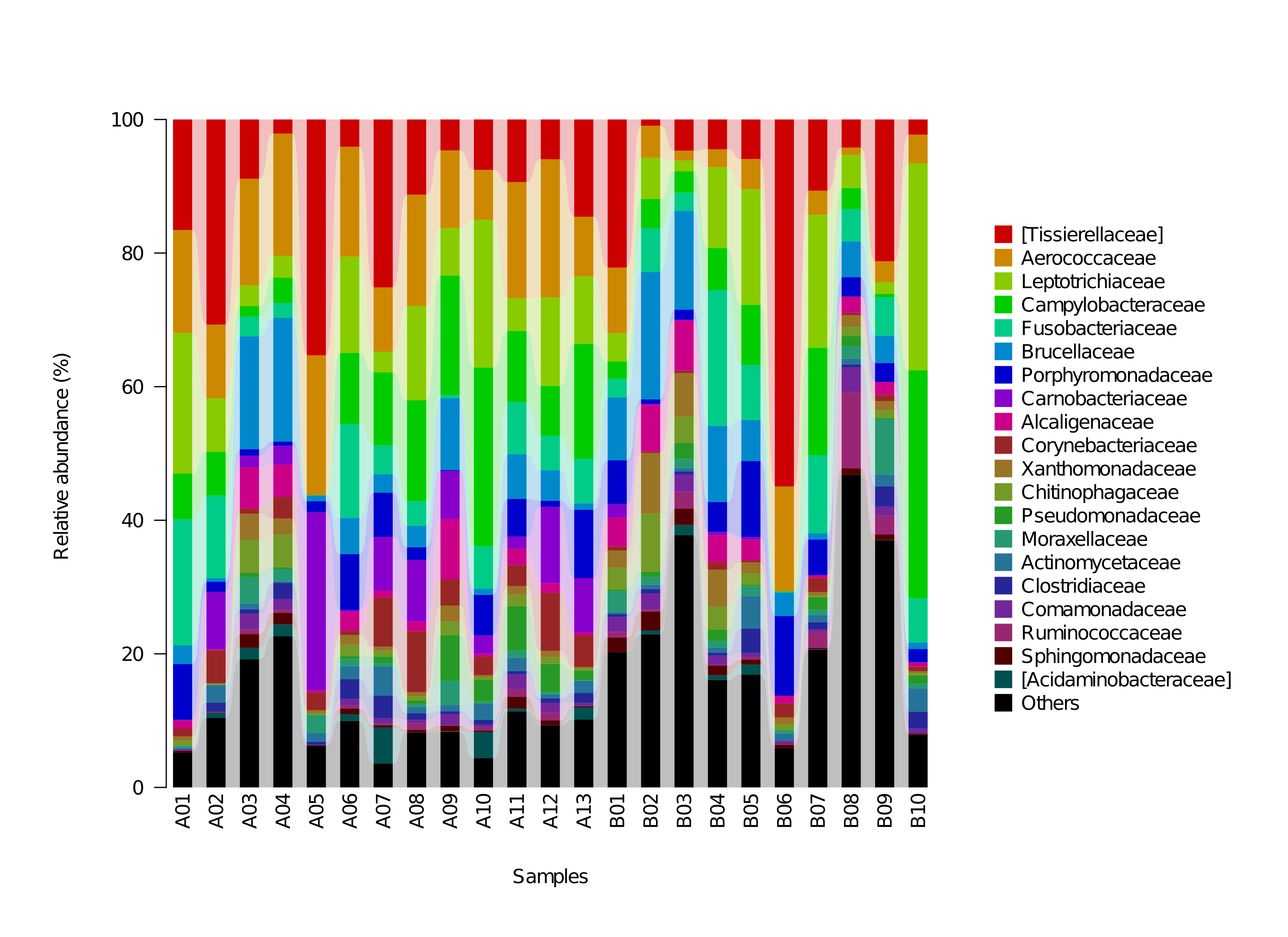

Supplement: Supplementary file 5 — Additional file 5. [file 12917_2022_3189_MOESM5_ESM.zip › MPL201709200_16s_yy/Treat1/B07_taxa_summary/family.png]

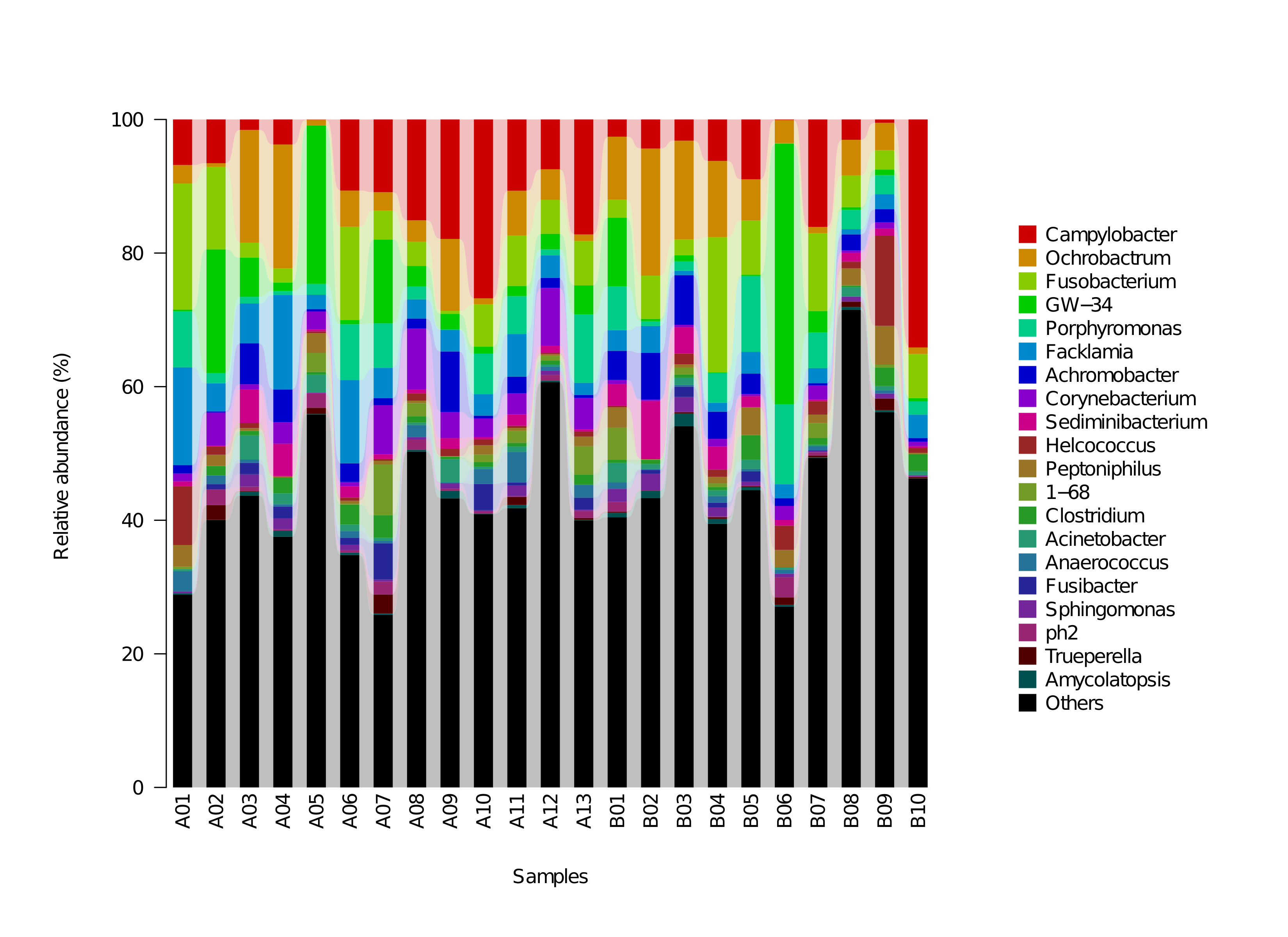

Supplement: Supplementary file 5 — Additional file 5. [file 12917_2022_3189_MOESM5_ESM.zip › MPL201709200_16s_yy/Treat1/B07_taxa_summary/genus.png]

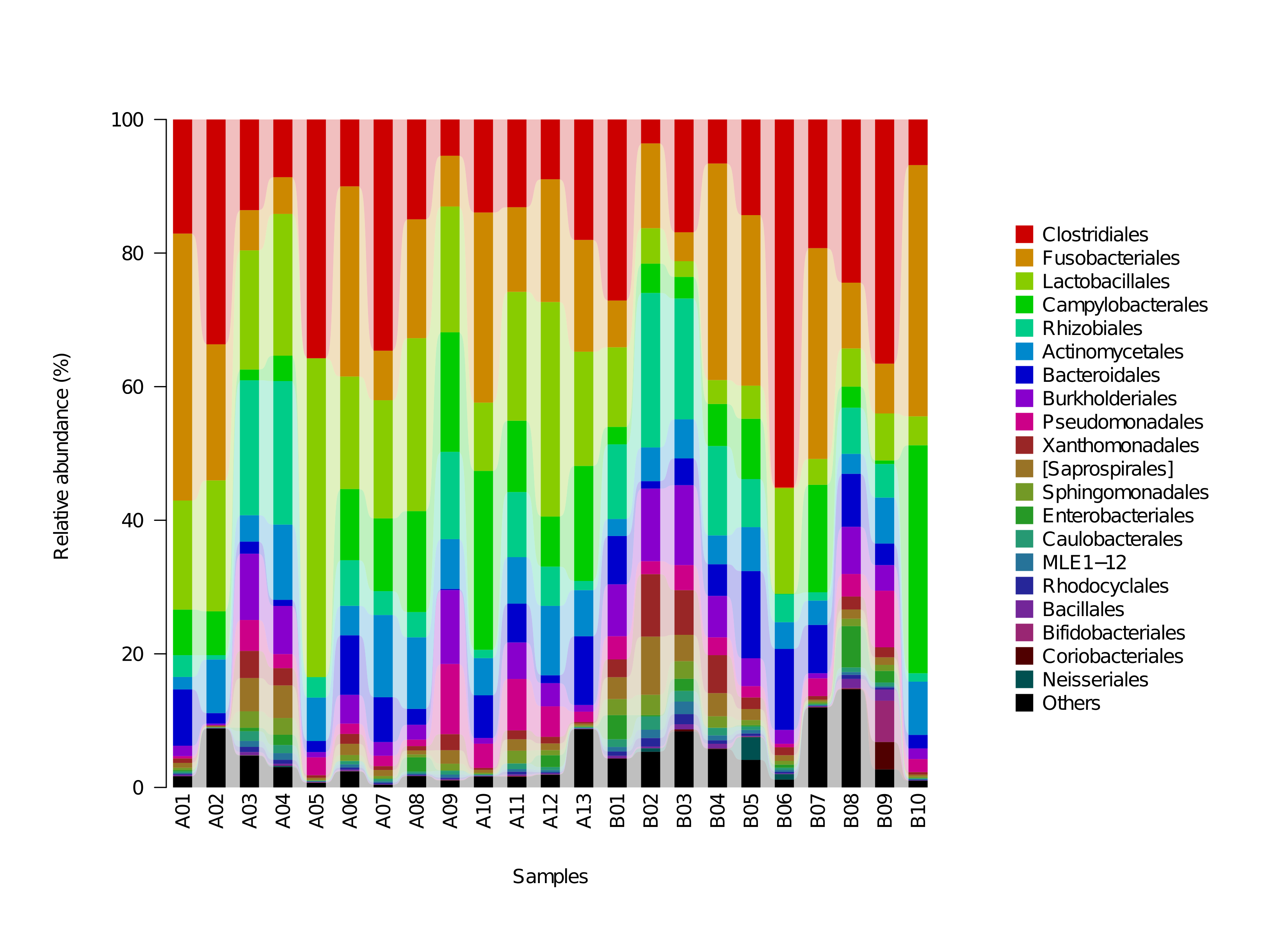

Supplement: Supplementary file 5 — Additional file 5. [file 12917_2022_3189_MOESM5_ESM.zip › MPL201709200_16s_yy/Treat1/B07_taxa_summary/order.png]

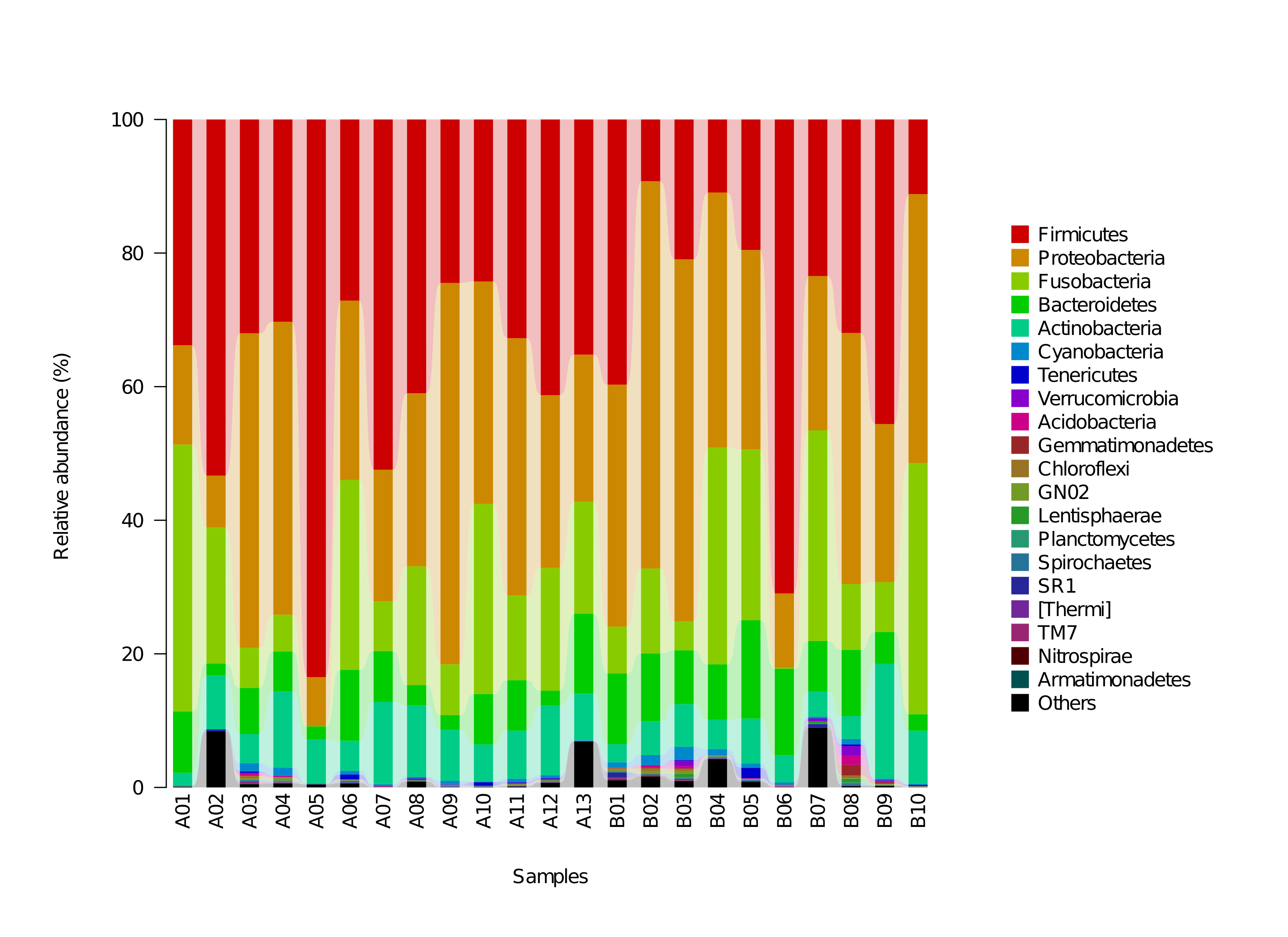

Supplement: Supplementary file 5 — Additional file 5. [file 12917_2022_3189_MOESM5_ESM.zip › MPL201709200_16s_yy/Treat1/B07_taxa_summary/phylum.png]

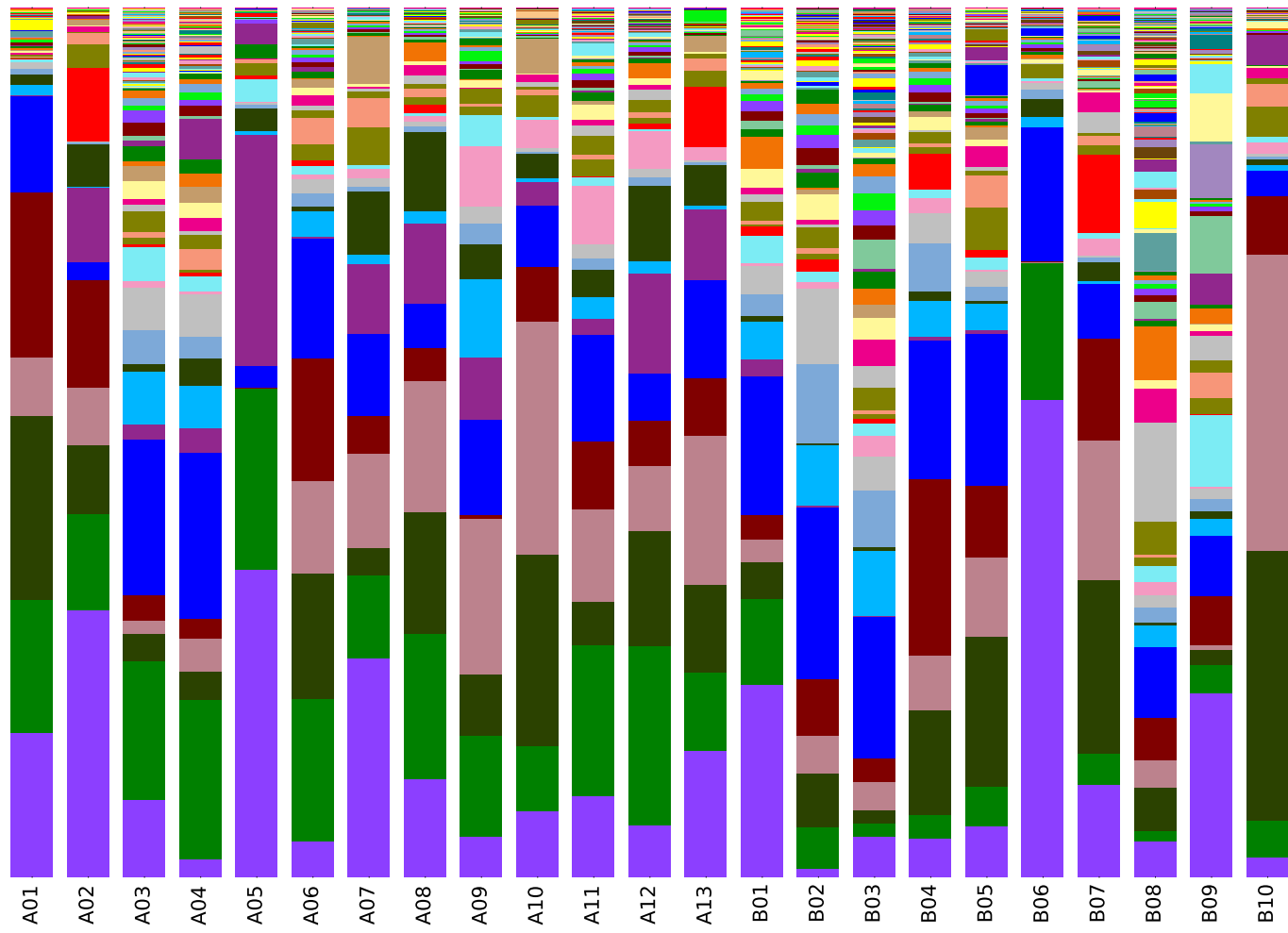

Supplement: Supplementary file 5 — Additional file 5. [file 12917_2022_3189_MOESM5_ESM.zip › MPL201709200_16s_yy/Treat1/B07_taxa_summary/taxa_summary_plots/charts/3N2Aikasq8U05aiXhXqGg6kB5rQR0m.pdf]

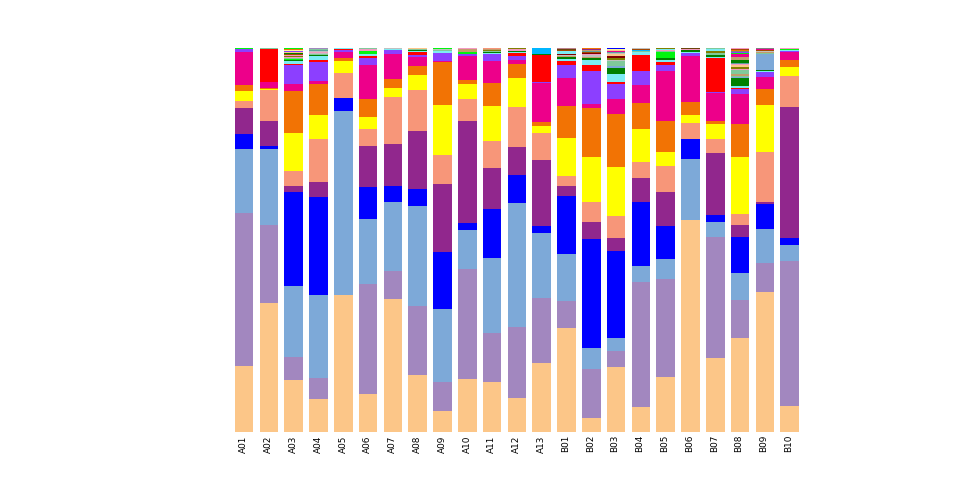

Supplement: Supplementary file 5 — Additional file 5. [file 12917_2022_3189_MOESM5_ESM.zip › MPL201709200_16s_yy/Treat1/B07_taxa_summary/taxa_summary_plots/charts/7YZLmUz9uzS7iDCWJ44LOX8015DagN.png]

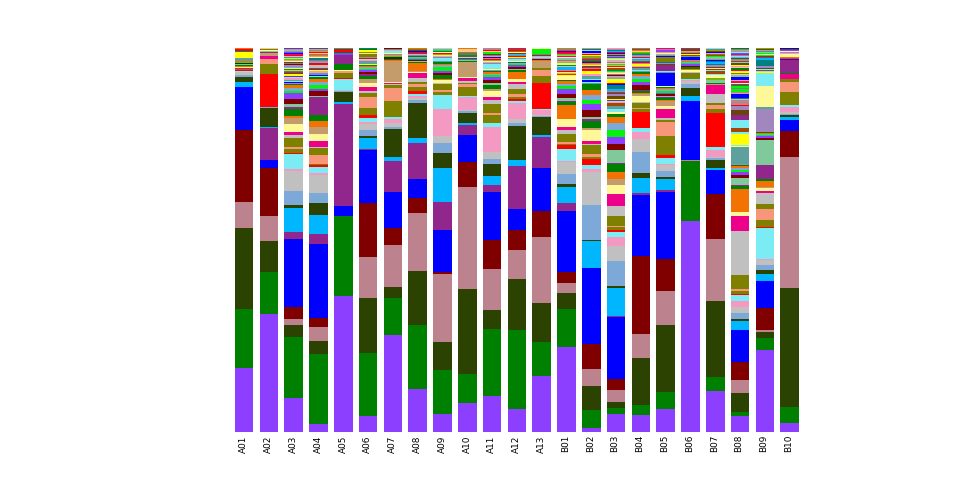

Supplement: Supplementary file 5 — Additional file 5. [file 12917_2022_3189_MOESM5_ESM.zip › MPL201709200_16s_yy/Treat1/B07_taxa_summary/taxa_summary_plots/charts/8hQEXhNGekhgERC58I9KYZz5xy59py.png]

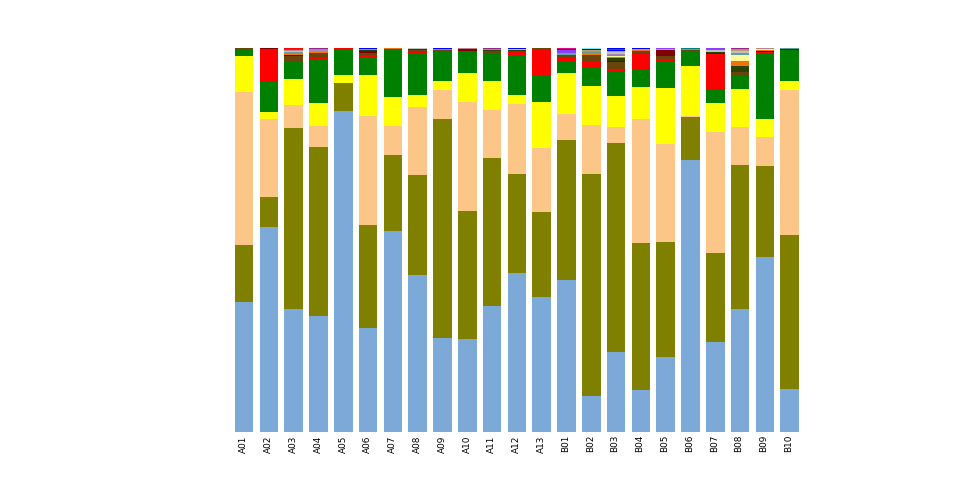

Supplement: Supplementary file 5 — Additional file 5. [file 12917_2022_3189_MOESM5_ESM.zip › MPL201709200_16s_yy/Treat1/B07_taxa_summary/taxa_summary_plots/charts/DN3P2ZniBnDQMENglxB6f7d9gOB2je.png]

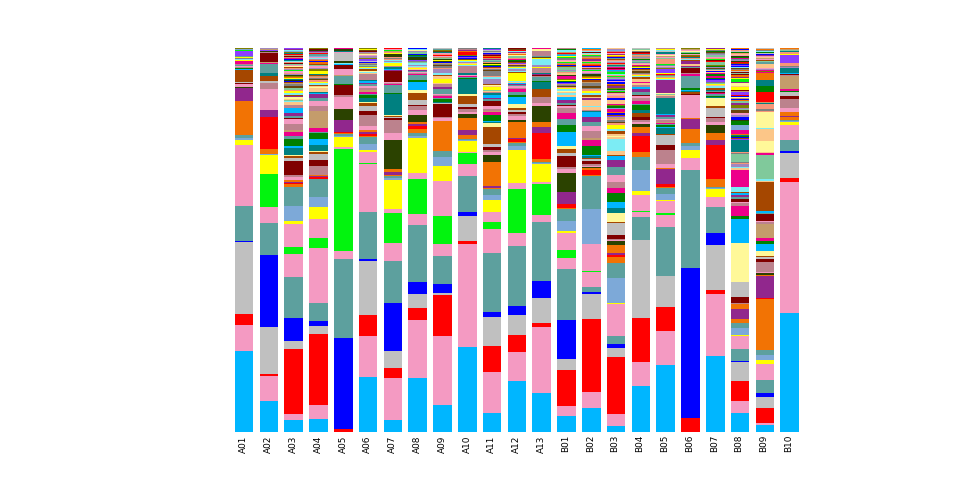

Supplement: Supplementary file 5 — Additional file 5. [file 12917_2022_3189_MOESM5_ESM.zip › MPL201709200_16s_yy/Treat1/B07_taxa_summary/taxa_summary_plots/charts/LZM8GGeezP06dAOwPUzZNEZnKp2tkz.png]

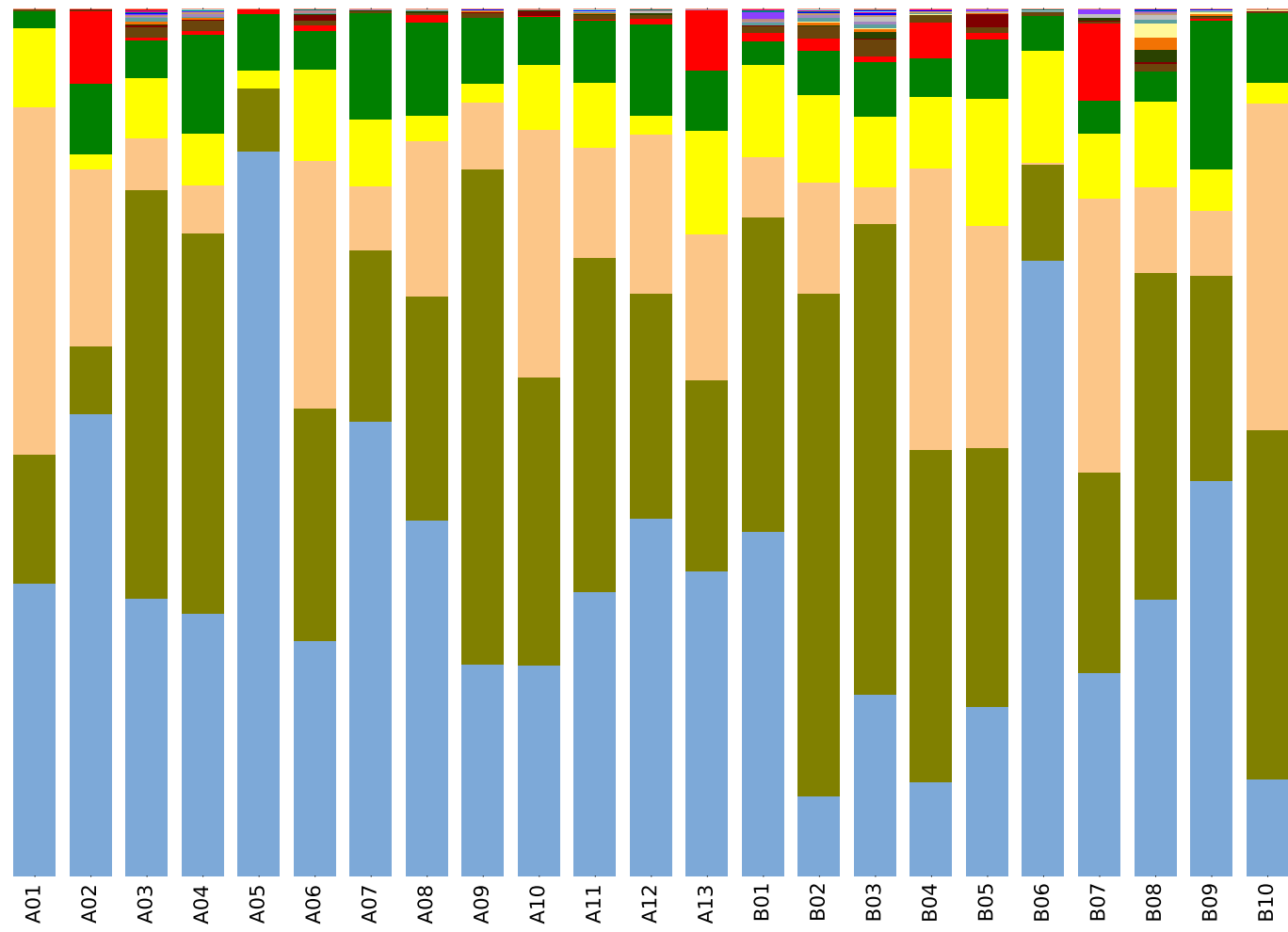

Supplement: Supplementary file 5 — Additional file 5. [file 12917_2022_3189_MOESM5_ESM.zip › MPL201709200_16s_yy/Treat1/B07_taxa_summary/taxa_summary_plots/charts/MHx86YGNGlWyjdrEeX8ioDzHQhD2Td.pdf]

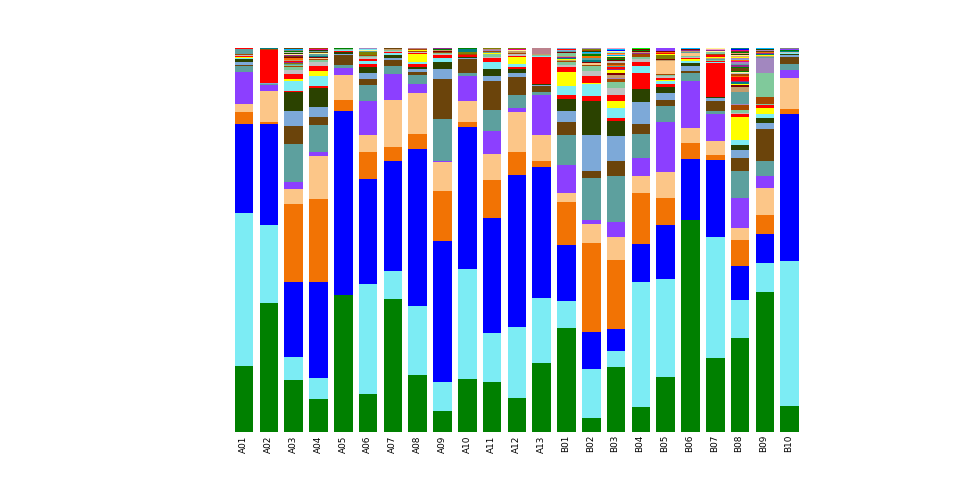

Supplement: Supplementary file 5 — Additional file 5. [file 12917_2022_3189_MOESM5_ESM.zip › MPL201709200_16s_yy/Treat1/B07_taxa_summary/taxa_summary_plots/charts/SBew7Rxbnw2pU9JFC6Ob7hzG1u4afX.png]

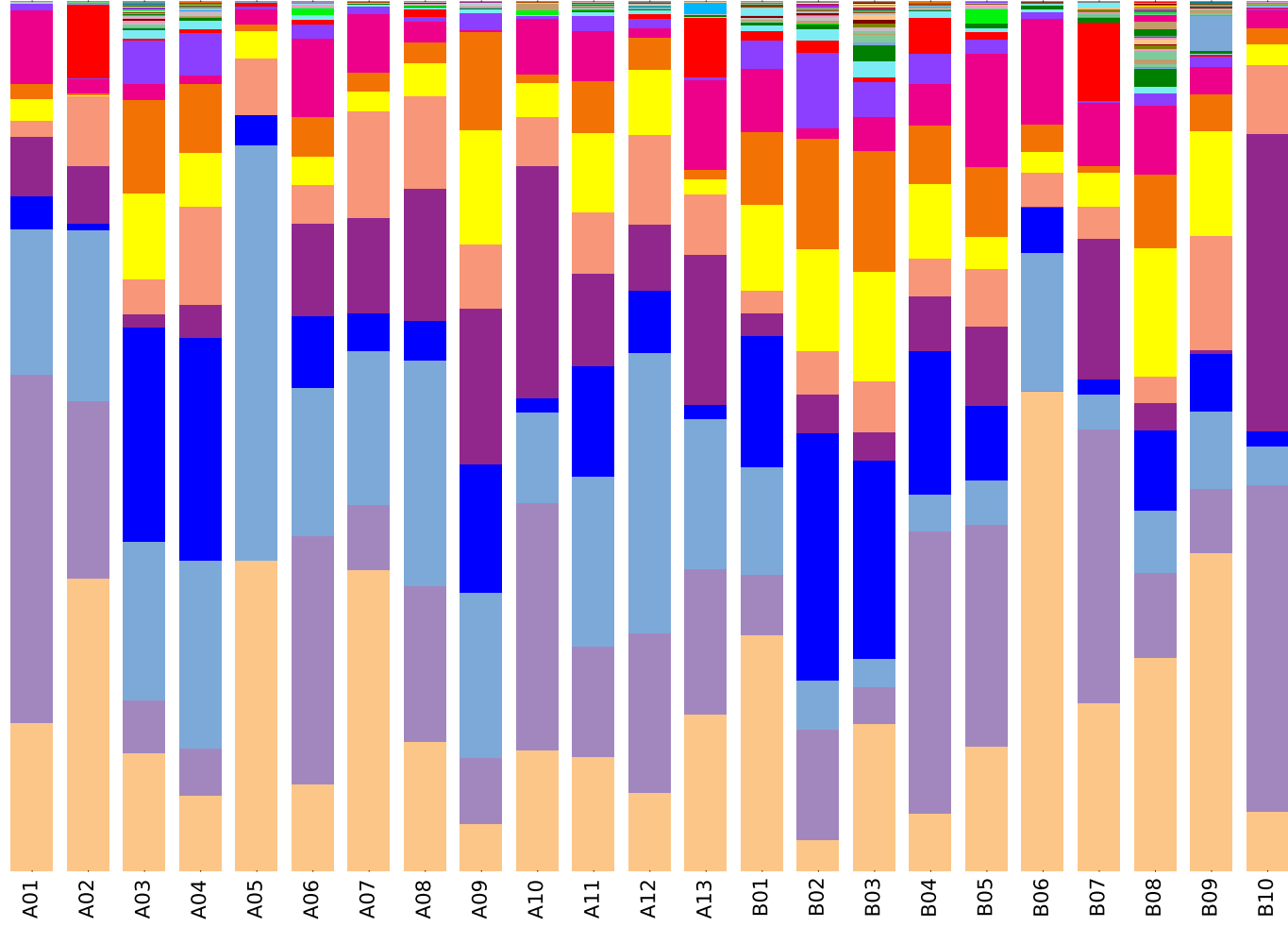

Supplement: Supplementary file 5 — Additional file 5. [file 12917_2022_3189_MOESM5_ESM.zip › MPL201709200_16s_yy/Treat1/B07_taxa_summary/taxa_summary_plots/charts/aDMMpaccmR6TZSqb30bZJFYDGHh5L7.pdf]

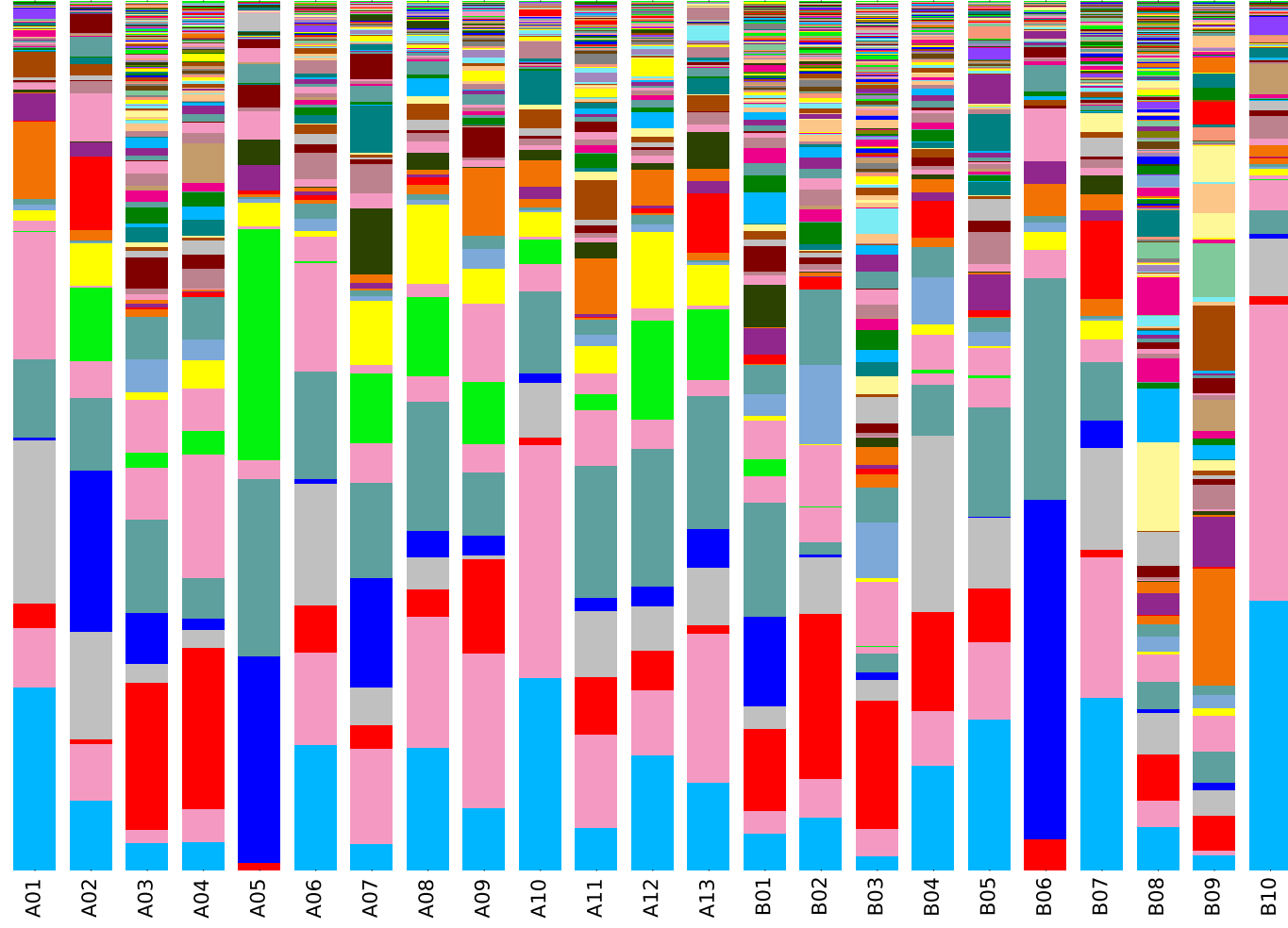

Supplement: Supplementary file 5 — Additional file 5. [file 12917_2022_3189_MOESM5_ESM.zip › MPL201709200_16s_yy/Treat1/B07_taxa_summary/taxa_summary_plots/charts/rX6sZXWsM2gJ15zAs70FYUSMlFxET3.pdf]

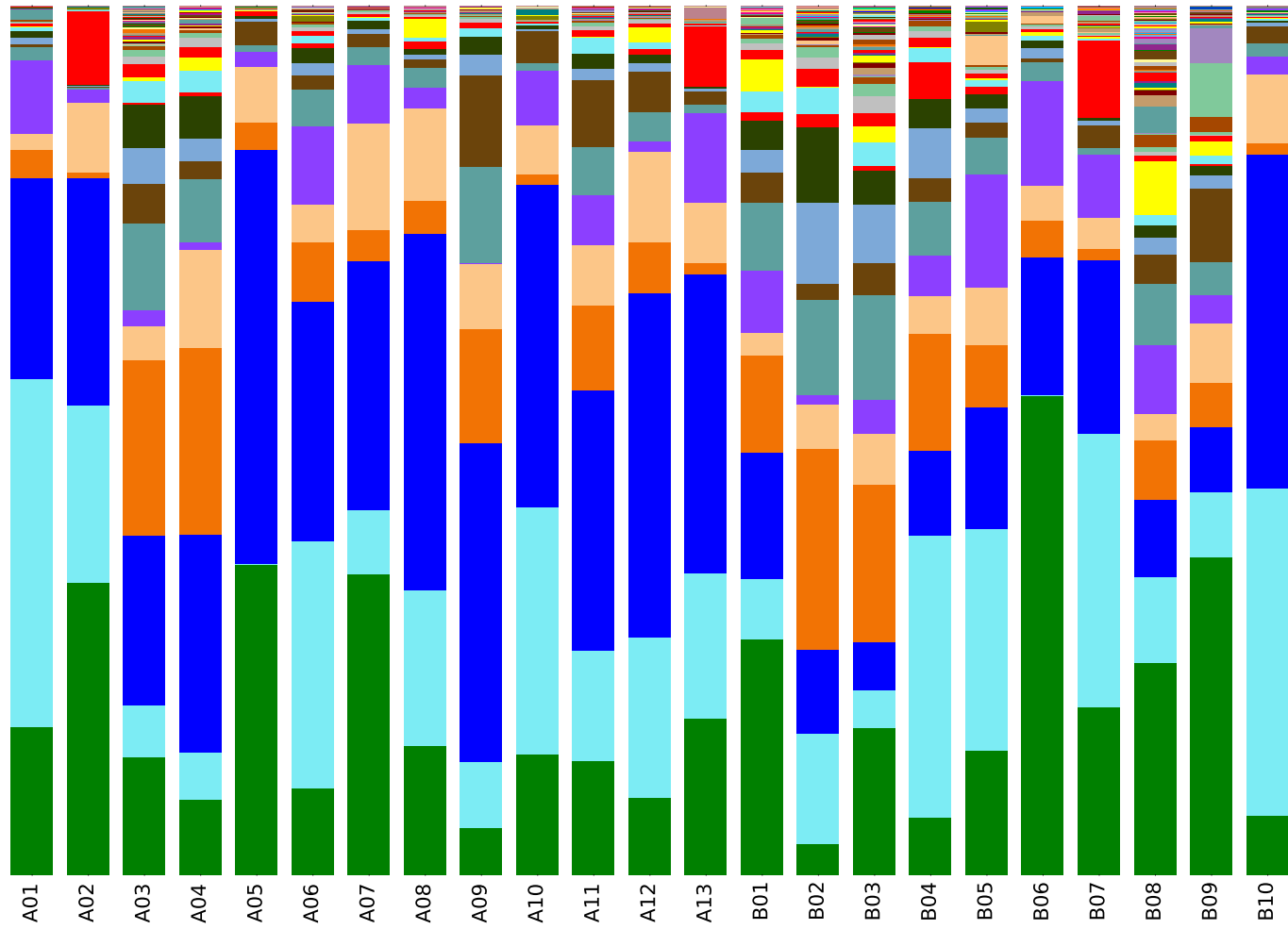

Supplement: Supplementary file 5 — Additional file 5. [file 12917_2022_3189_MOESM5_ESM.zip › MPL201709200_16s_yy/Treat1/B07_taxa_summary/taxa_summary_plots/charts/zmGZZnfBHEtCK9aBTbUATrGiKKqNGa.pdf]

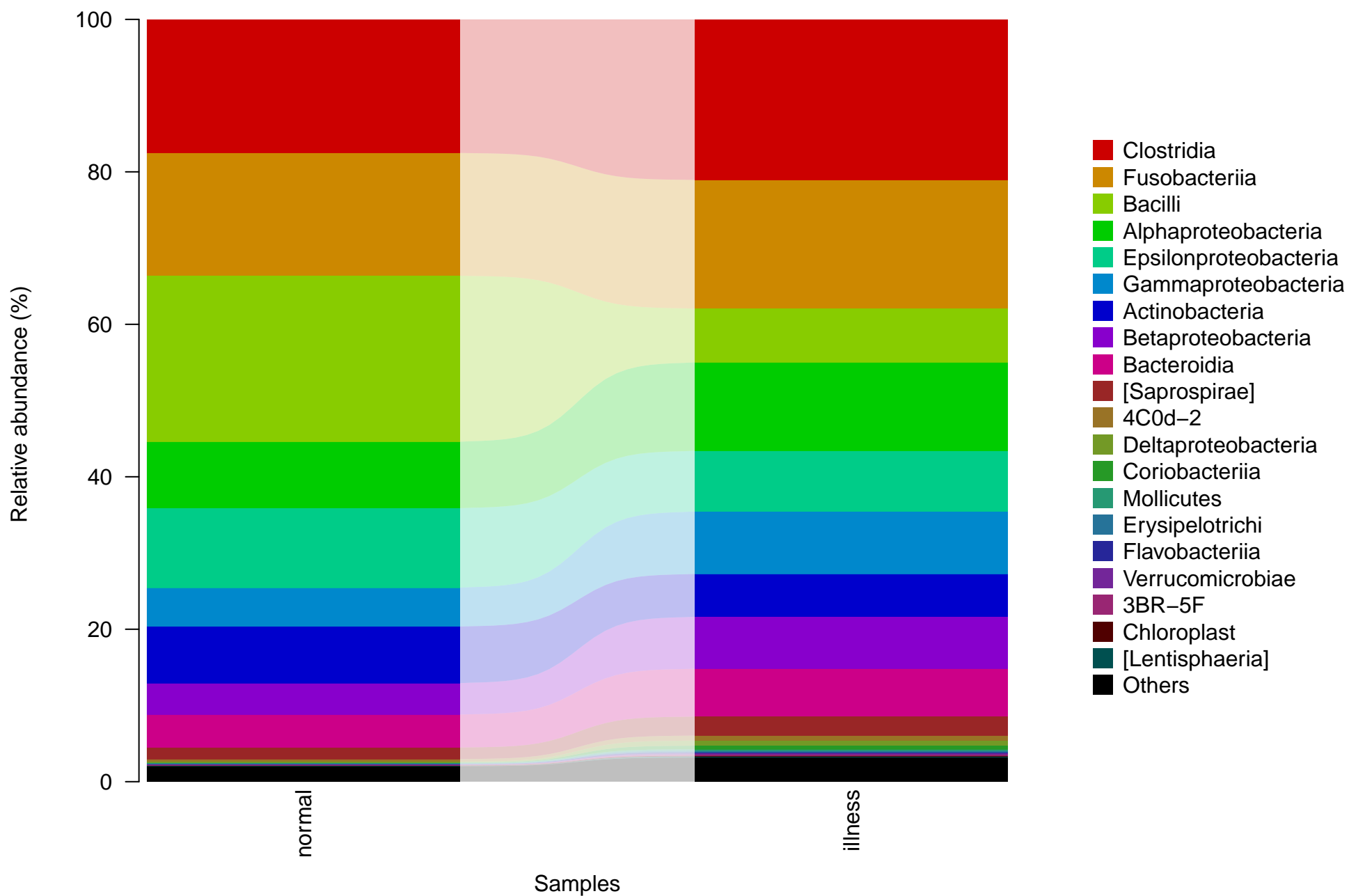

Supplement: Supplementary file 5 — Additional file 5. [file 12917_2022_3189_MOESM5_ESM.zip › MPL201709200_16s_yy/Treat1/B07_taxa_summary_group/bar_class_group.pdf]

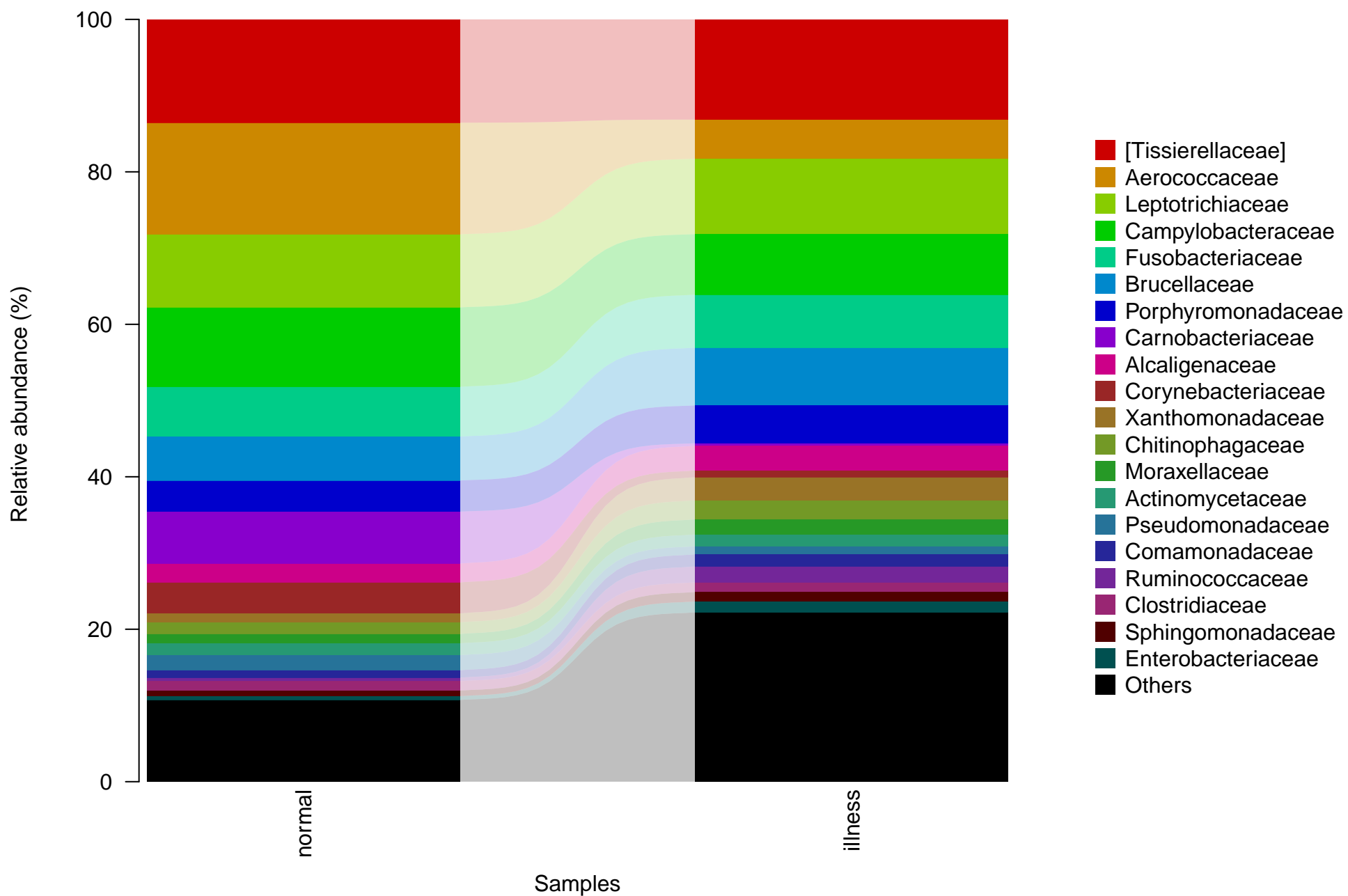

Supplement: Supplementary file 5 — Additional file 5. [file 12917_2022_3189_MOESM5_ESM.zip › MPL201709200_16s_yy/Treat1/B07_taxa_summary_group/bar_family_group.pdf]

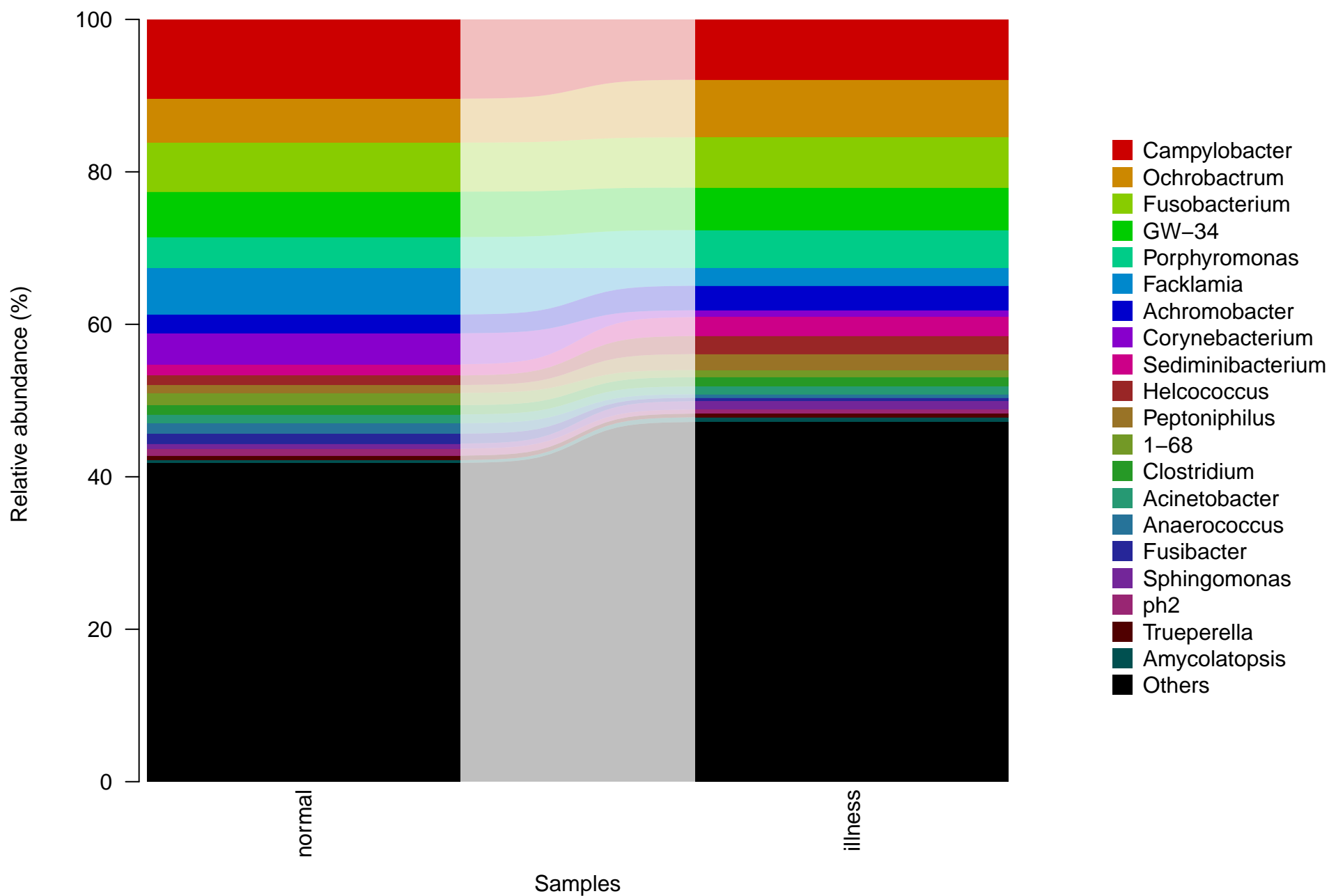

Supplement: Supplementary file 5 — Additional file 5. [file 12917_2022_3189_MOESM5_ESM.zip › MPL201709200_16s_yy/Treat1/B07_taxa_summary_group/bar_genus_group.pdf]

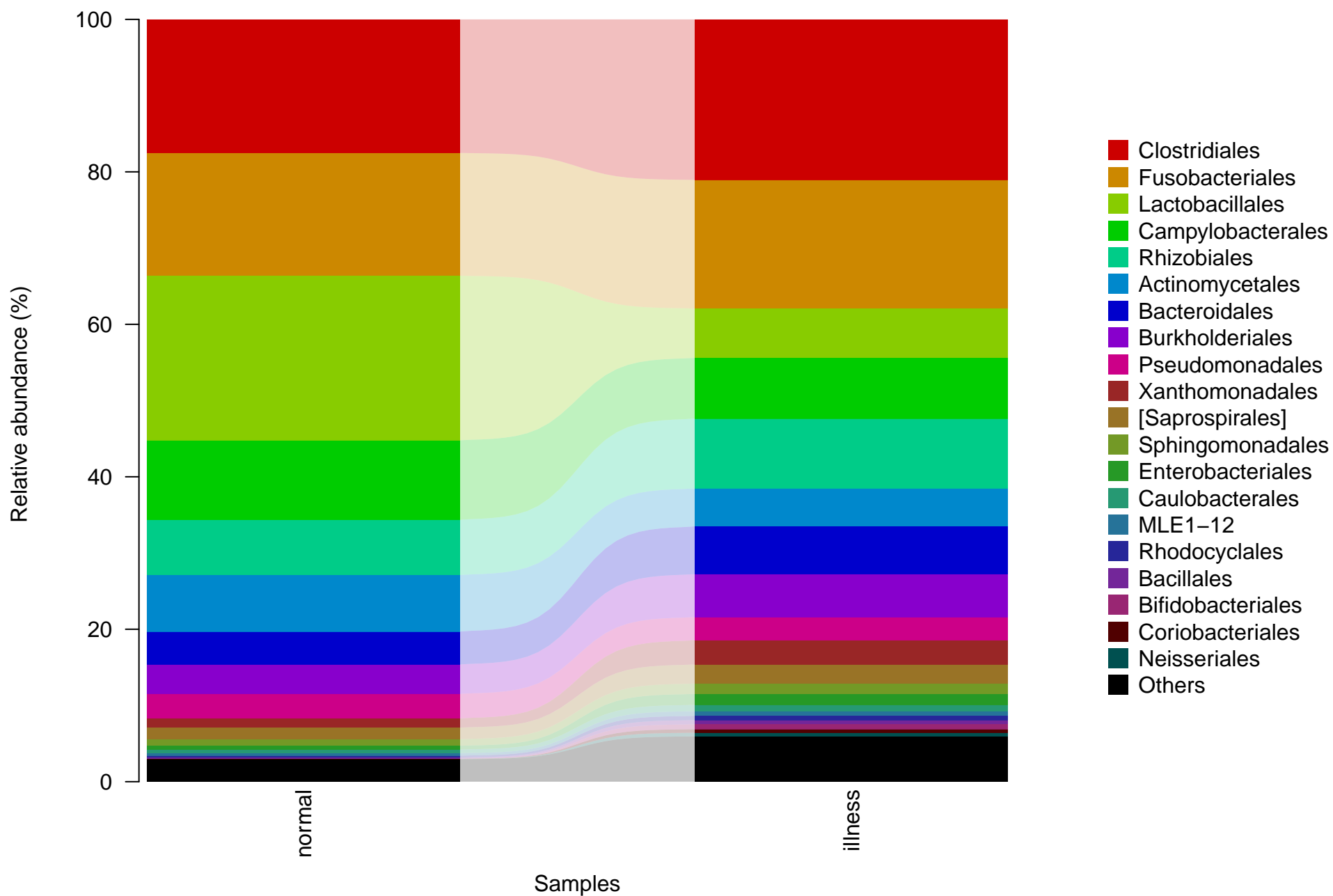

Supplement: Supplementary file 5 — Additional file 5. [file 12917_2022_3189_MOESM5_ESM.zip › MPL201709200_16s_yy/Treat1/B07_taxa_summary_group/bar_order_group.pdf]

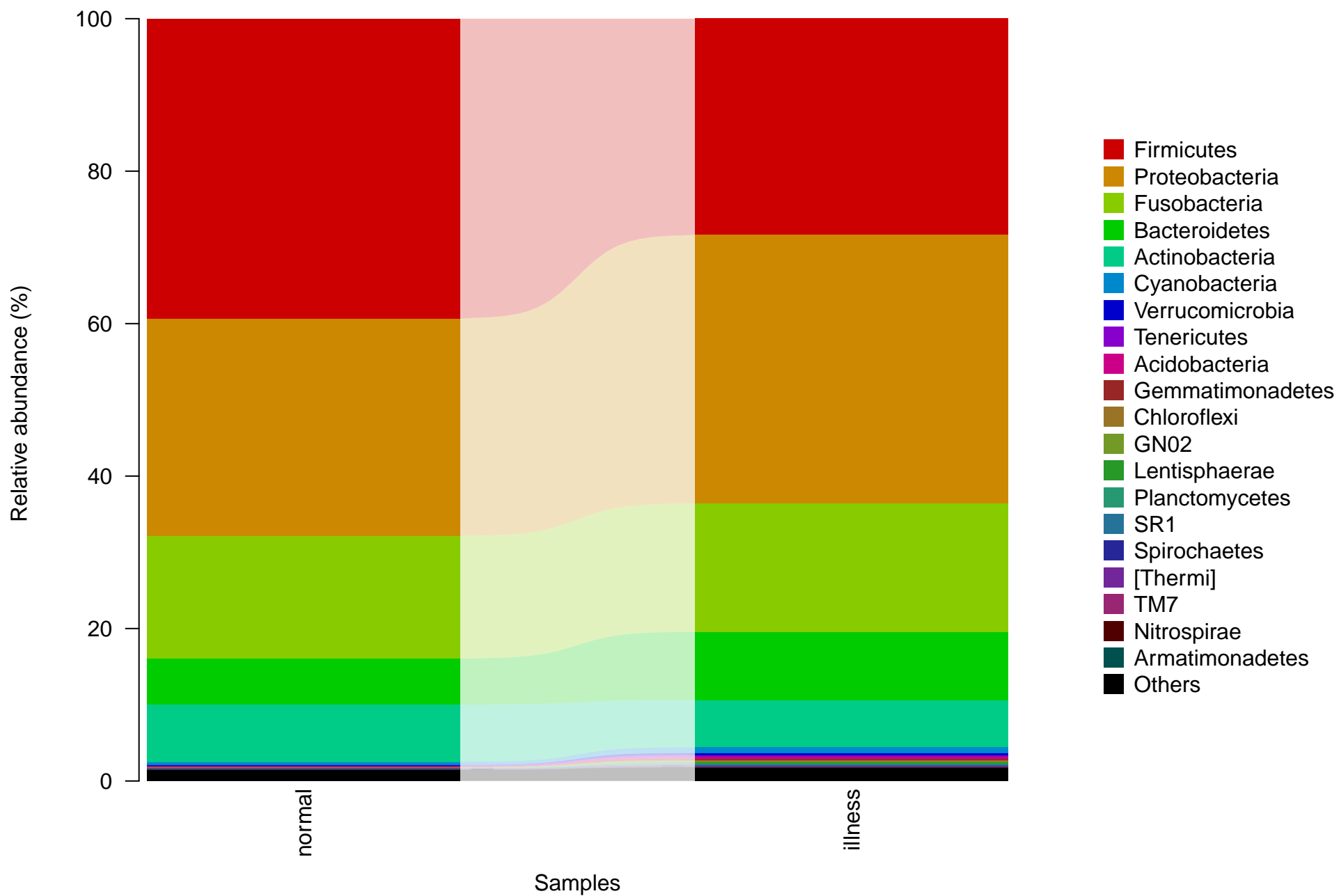

Supplement: Supplementary file 5 — Additional file 5. [file 12917_2022_3189_MOESM5_ESM.zip › MPL201709200_16s_yy/Treat1/B07_taxa_summary_group/bar_phylum_group.pdf]

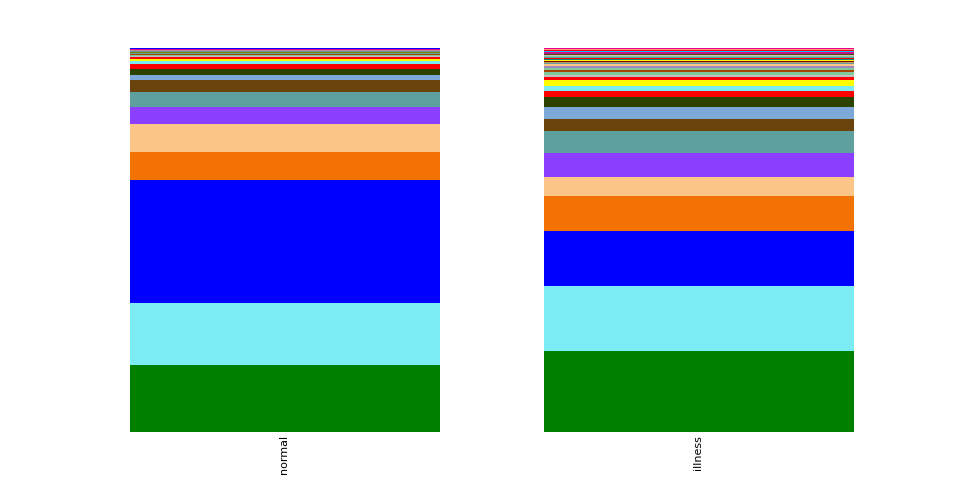

Supplement: Supplementary file 5 — Additional file 5. [file 12917_2022_3189_MOESM5_ESM.zip › MPL201709200_16s_yy/Treat1/B07_taxa_summary_group/taxa_summary_plots/charts/1FLerSsyfw3FSrsP8ZIDwa4lzBiZwR.png]

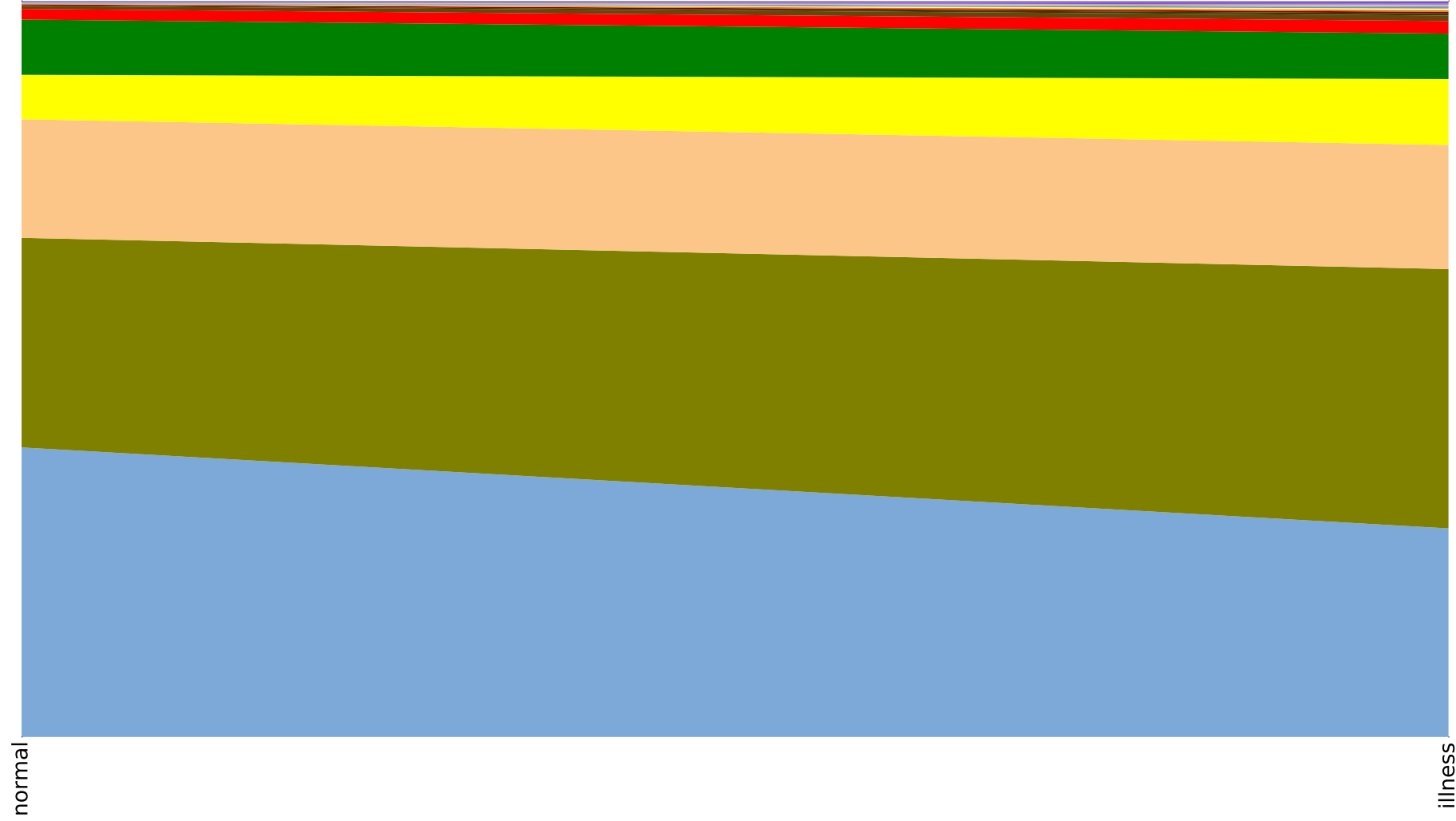

Supplement: Supplementary file 5 — Additional file 5. [file 12917_2022_3189_MOESM5_ESM.zip › MPL201709200_16s_yy/Treat1/B07_taxa_summary_group/taxa_summary_plots/charts/1nQOuq6gsh32ZoXYfKTeOT8Z0jpDju.pdf]

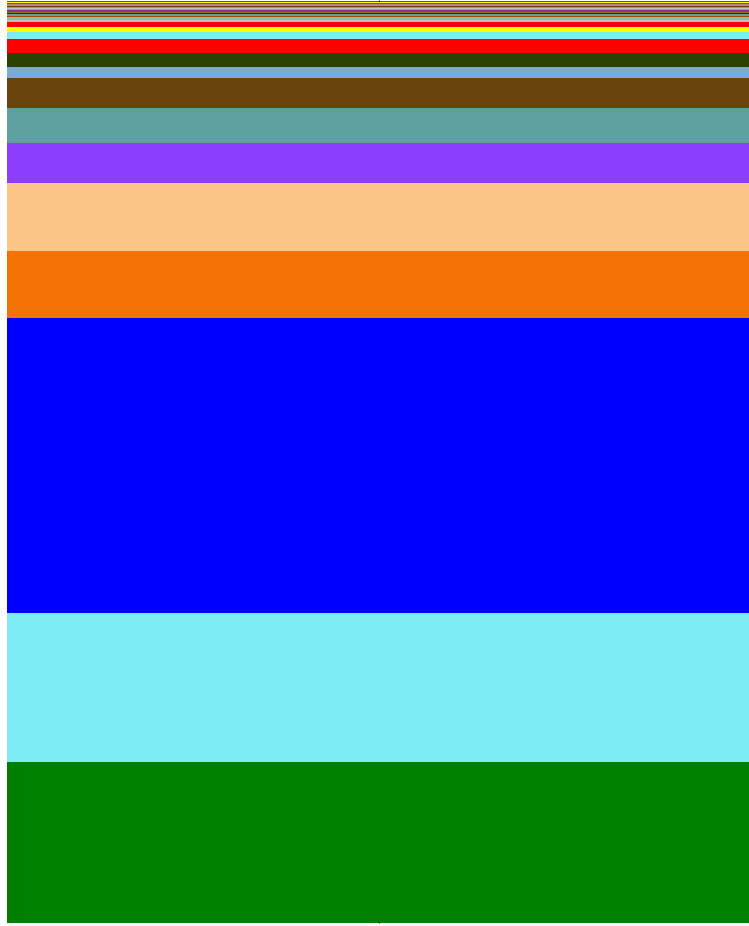

normal

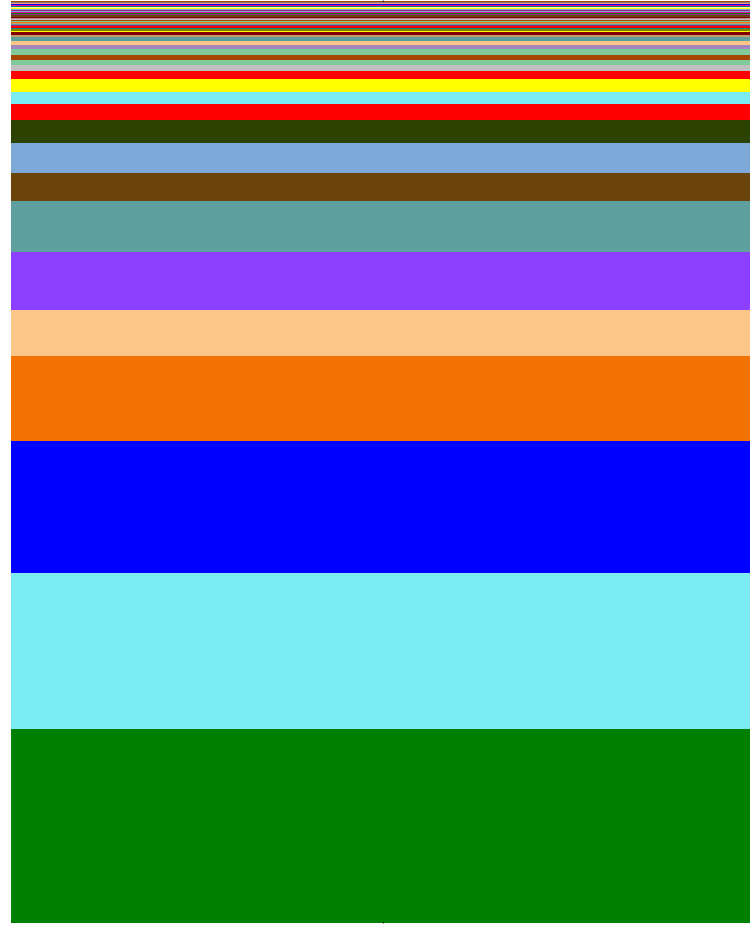

illness

Supplement: Supplementary file 5 — Additional file 5. [file 12917_2022_3189_MOESM5_ESM.zip › MPL201709200_16s_yy/Treat1/B07_taxa_summary_group/taxa_summary_plots/charts/4f53kl0yX5CZTMOy92mWtYUzckHAcE.pdf]

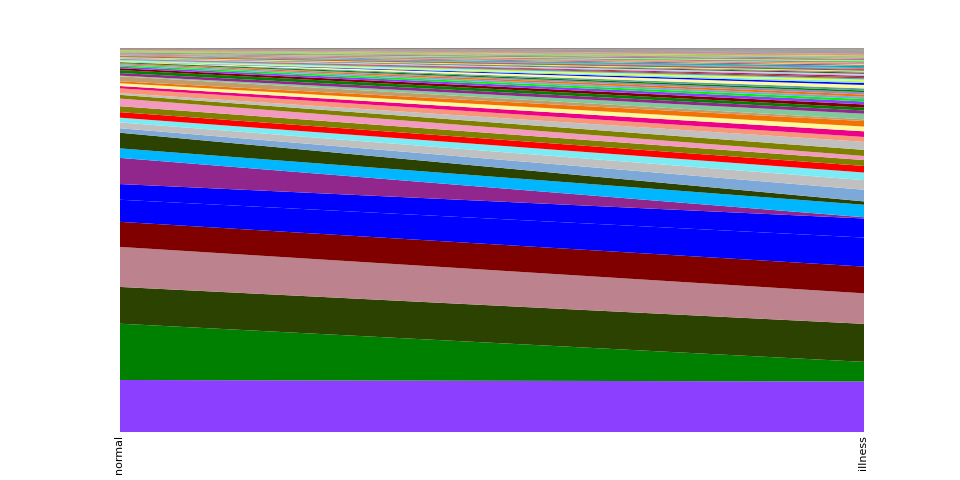

Supplement: Supplementary file 5 — Additional file 5. [file 12917_2022_3189_MOESM5_ESM.zip › MPL201709200_16s_yy/Treat1/B07_taxa_summary_group/taxa_summary_plots/charts/7kLbh317B4KunwfpmjOMPnqzqy0kXb.png]

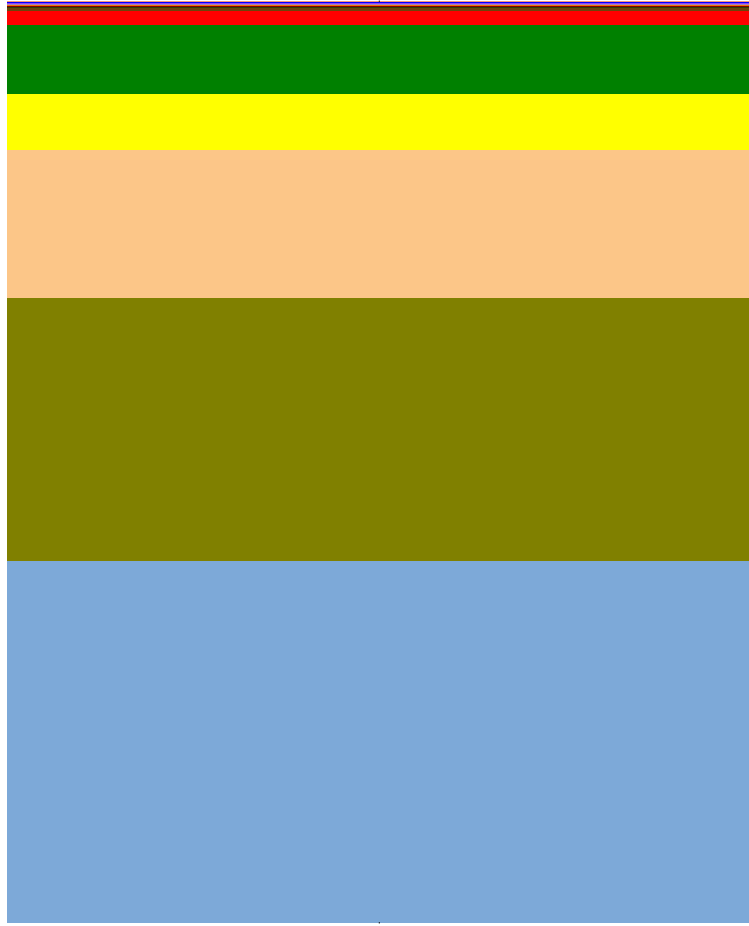

normal

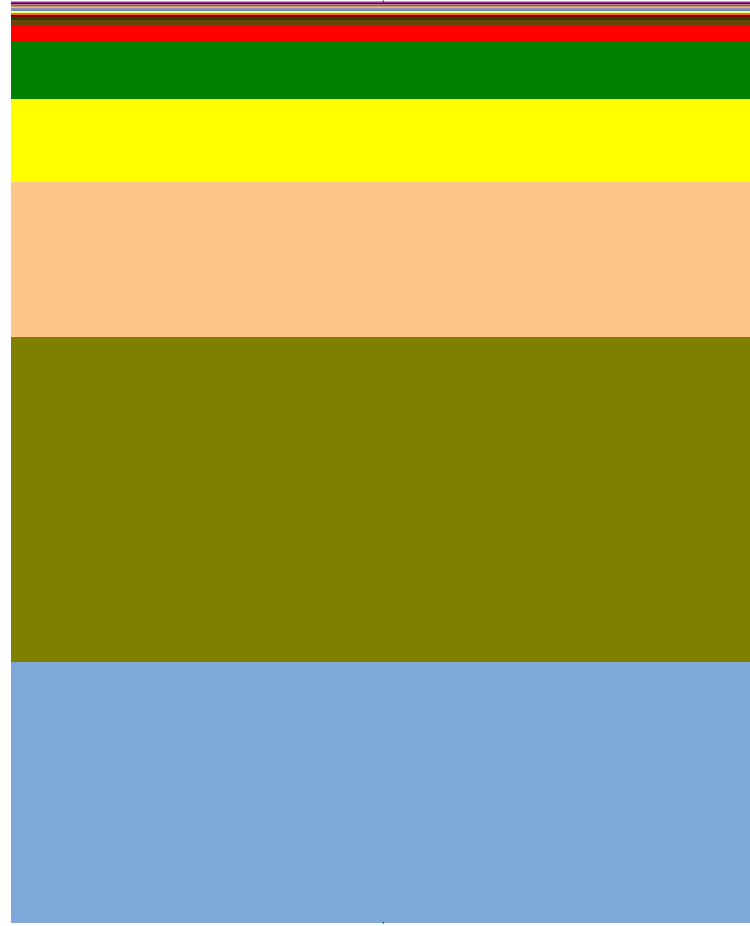

illness

Supplement: Supplementary file 5 — Additional file 5. [file 12917_2022_3189_MOESM5_ESM.zip › MPL201709200_16s_yy/Treat1/B07_taxa_summary_group/taxa_summary_plots/charts/8fI6JzHTugF3HnFR1Bjf58S9YCL5J6.pdf]

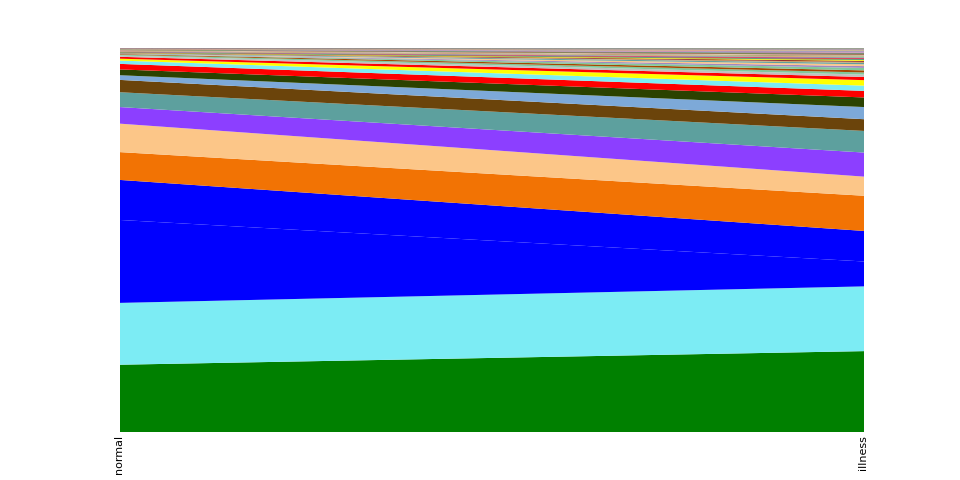

Supplement: Supplementary file 5 — Additional file 5. [file 12917_2022_3189_MOESM5_ESM.zip › MPL201709200_16s_yy/Treat1/B07_taxa_summary_group/taxa_summary_plots/charts/DbtzqzZQa5UQSuDzxnOXZRJCqrtL0Y.png]

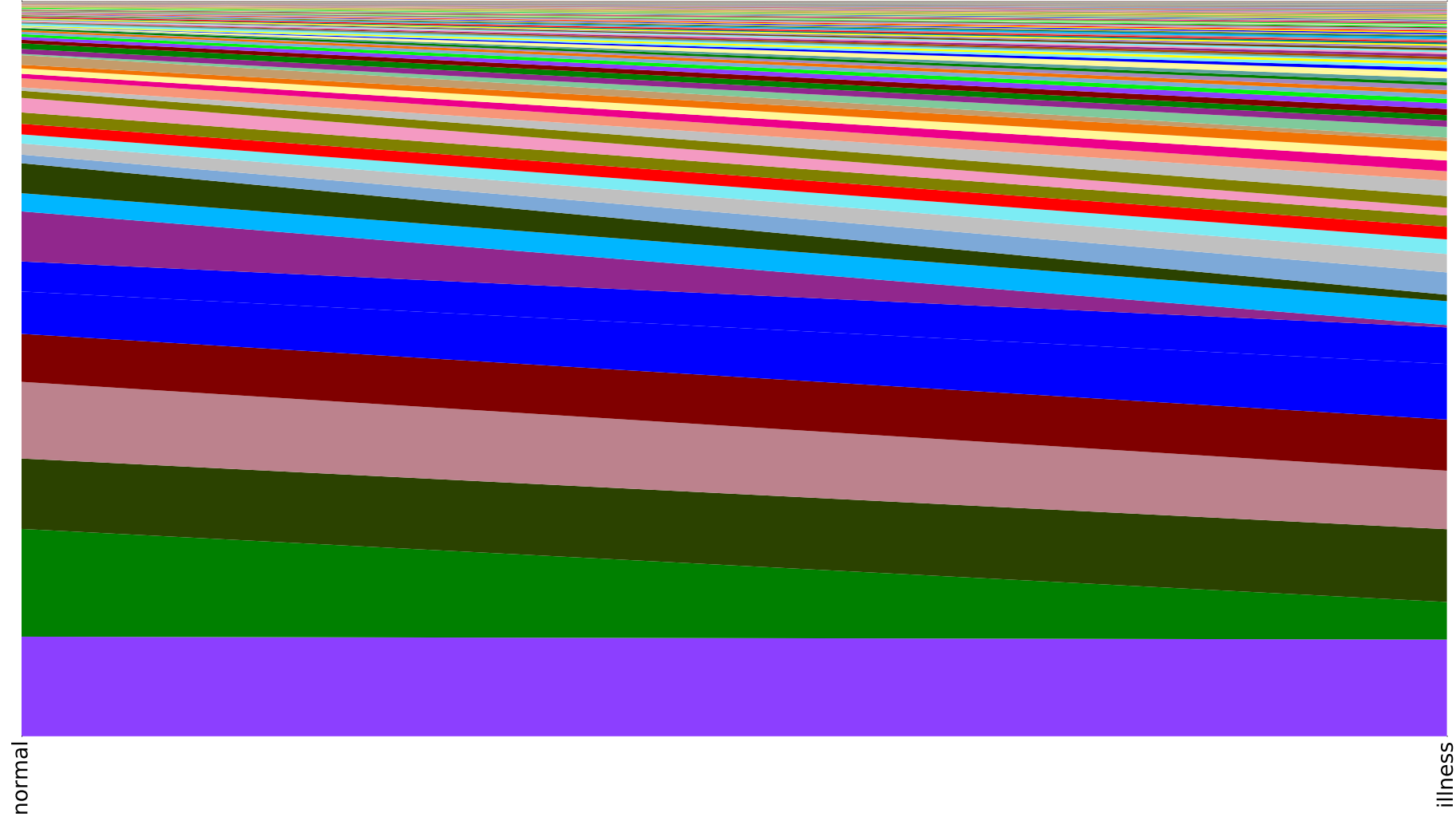

Supplement: Supplementary file 5 — Additional file 5. [file 12917_2022_3189_MOESM5_ESM.zip › MPL201709200_16s_yy/Treat1/B07_taxa_summary_group/taxa_summary_plots/charts/Eeb7zWN65yFaZzT8F6nD80iOJI6t0G.pdf]

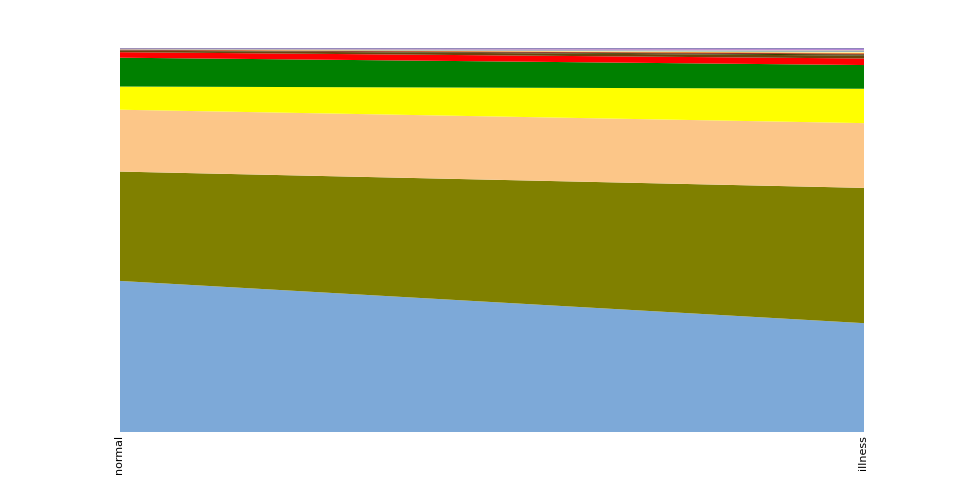

Supplement: Supplementary file 5 — Additional file 5. [file 12917_2022_3189_MOESM5_ESM.zip › MPL201709200_16s_yy/Treat1/B07_taxa_summary_group/taxa_summary_plots/charts/KriMcTu5Hl7hszkXJ0WgLraurHuhS6.png]

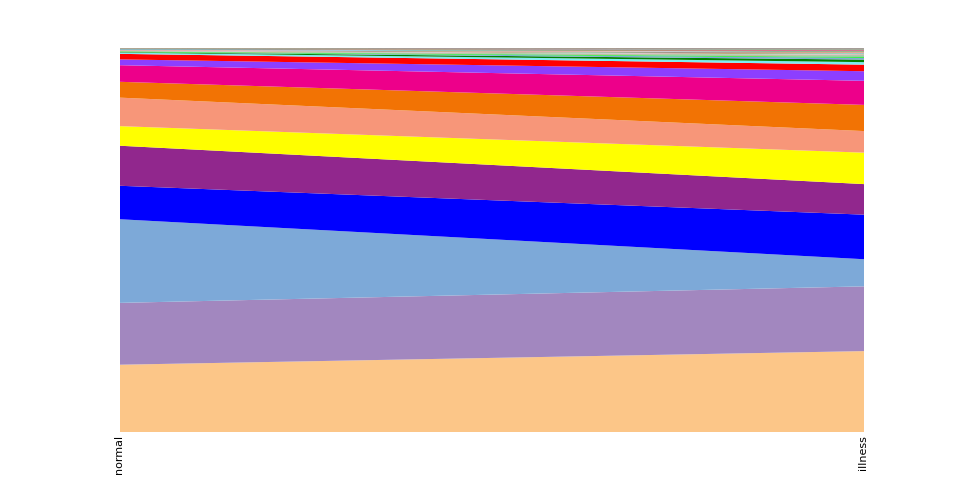

Supplement: Supplementary file 5 — Additional file 5. [file 12917_2022_3189_MOESM5_ESM.zip › MPL201709200_16s_yy/Treat1/B07_taxa_summary_group/taxa_summary_plots/charts/WqeQyjQiPz074miwdKPr5YLE92h7kI.png]

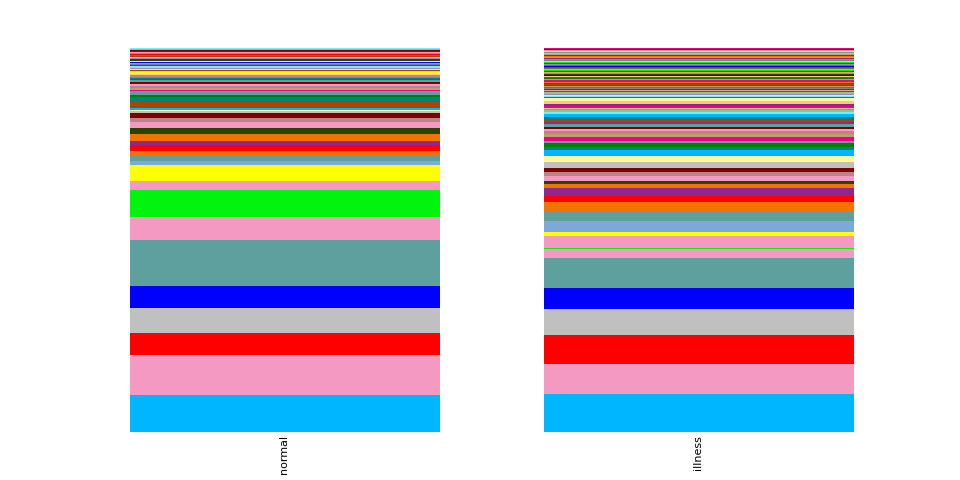

Supplement: Supplementary file 5 — Additional file 5. [file 12917_2022_3189_MOESM5_ESM.zip › MPL201709200_16s_yy/Treat1/B07_taxa_summary_group/taxa_summary_plots/charts/aUC8Y1lJE6YYnIkNazdZm0lUsZQJYm.png]

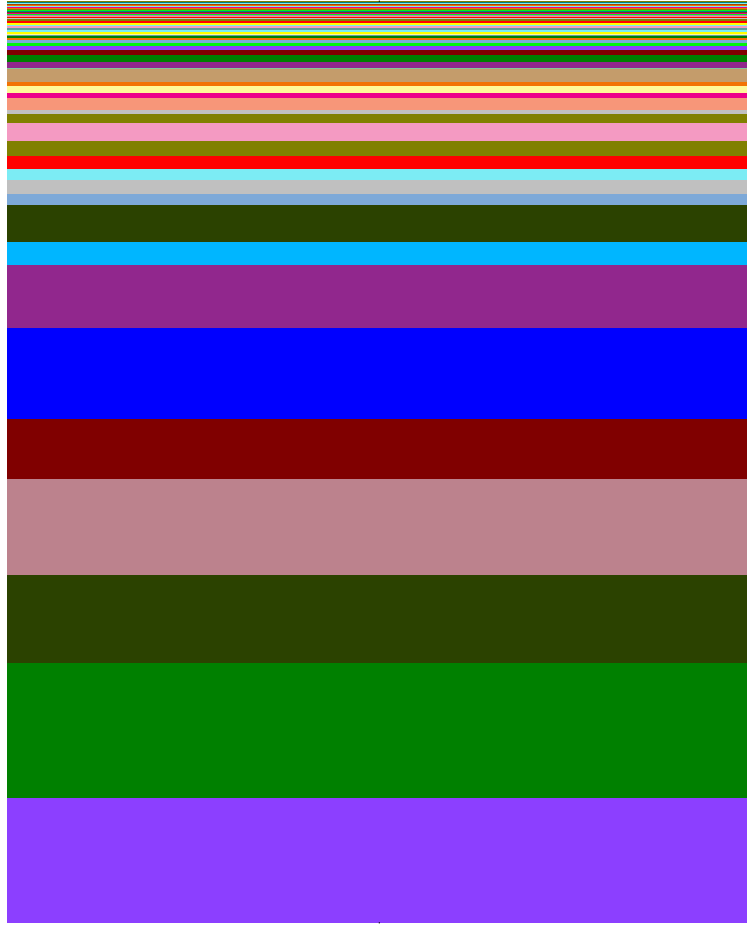

normal

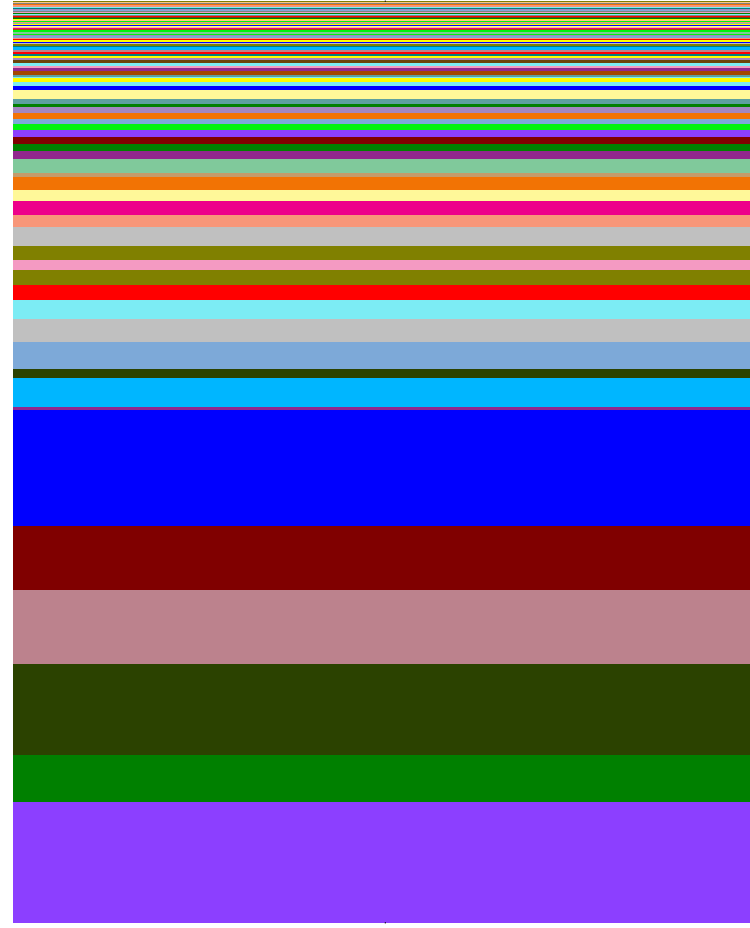

illness

Supplement: Supplementary file 5 — Additional file 5. [file 12917_2022_3189_MOESM5_ESM.zip › MPL201709200_16s_yy/Treat1/B07_taxa_summary_group/taxa_summary_plots/charts/czCQrYrBQZgFdAL2ru46Gt93AzQzRA.pdf]

normal

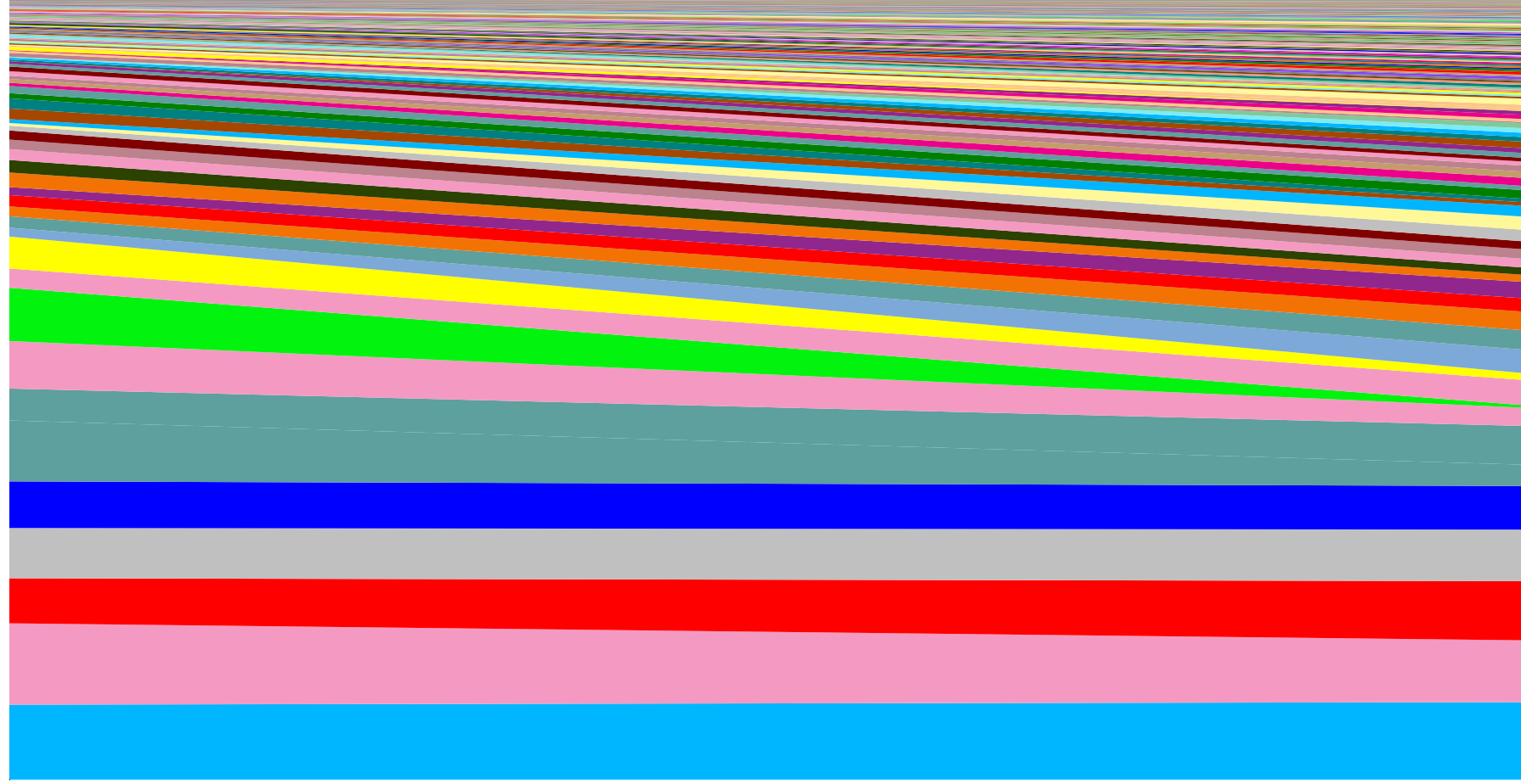

illness

Supplement: Supplementary file 5 — Additional file 5. [file 12917_2022_3189_MOESM5_ESM.zip › MPL201709200_16s_yy/Treat1/B07_taxa_summary_group/taxa_summary_plots/charts/eepQZ0ElilcFZzYn24xtAcnJCGJMch.pdf]

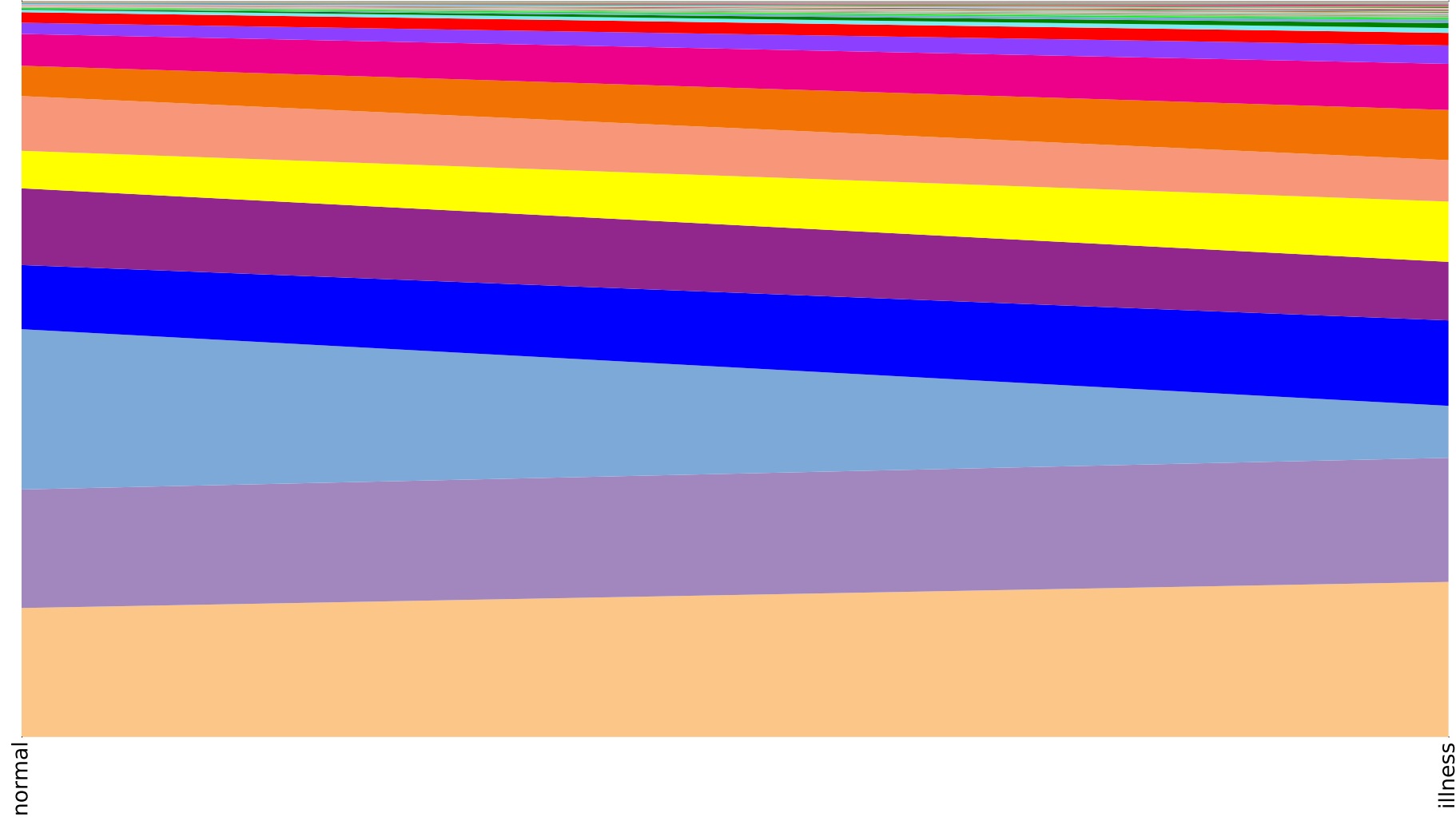

Supplement: Supplementary file 5 — Additional file 5. [file 12917_2022_3189_MOESM5_ESM.zip › MPL201709200_16s_yy/Treat1/B07_taxa_summary_group/taxa_summary_plots/charts/gzIimXWcJXbXbdjQ6yniyC4bJrzDyR.pdf]

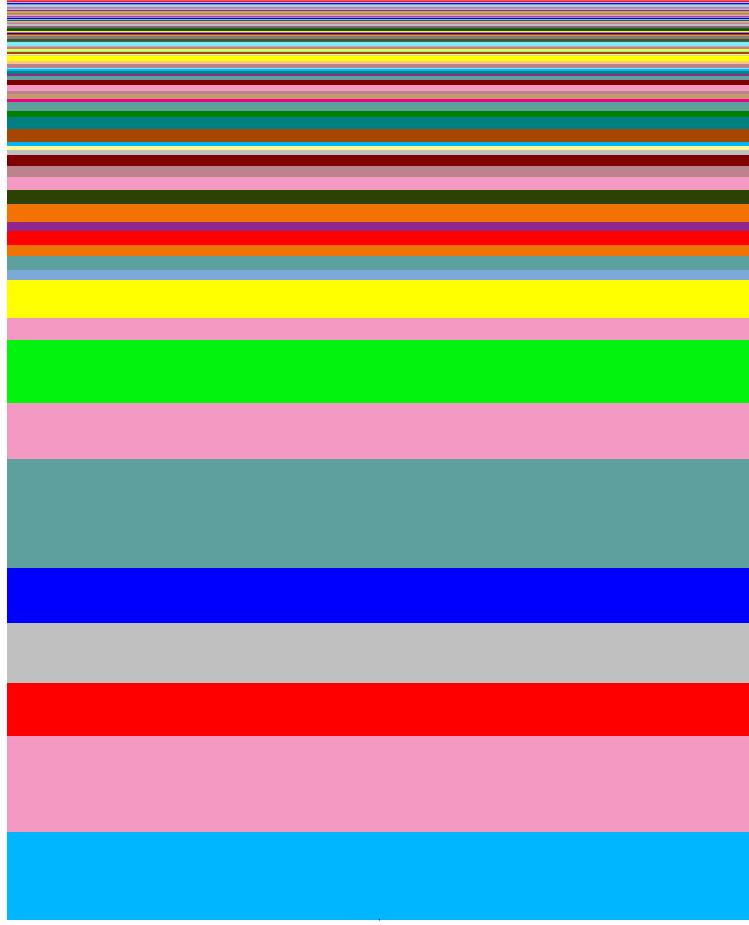

normal

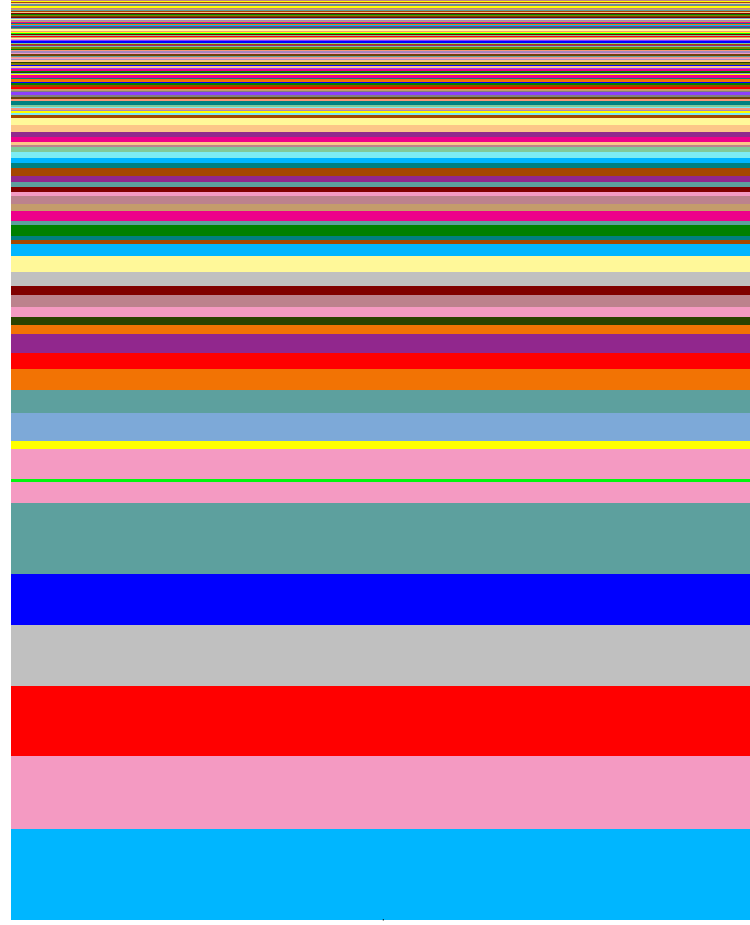

illness

Supplement: Supplementary file 5 — Additional file 5. [file 12917_2022_3189_MOESM5_ESM.zip › MPL201709200_16s_yy/Treat1/B07_taxa_summary_group/taxa_summary_plots/charts/j0lAZf3JfZT1X0kBhKxJUSdY18BycA.pdf]

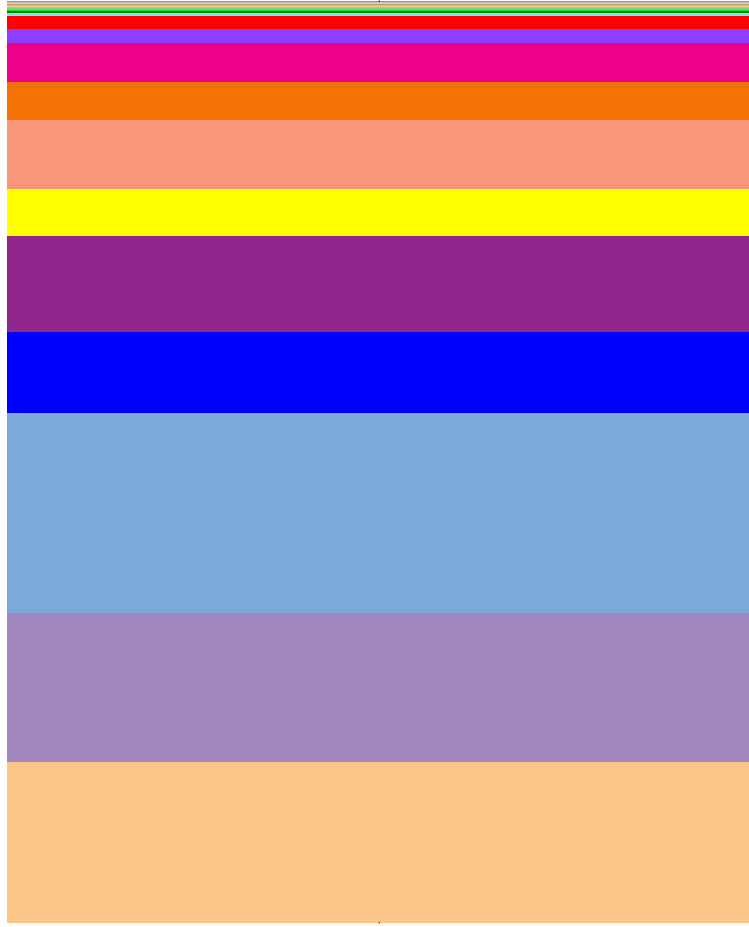

normal

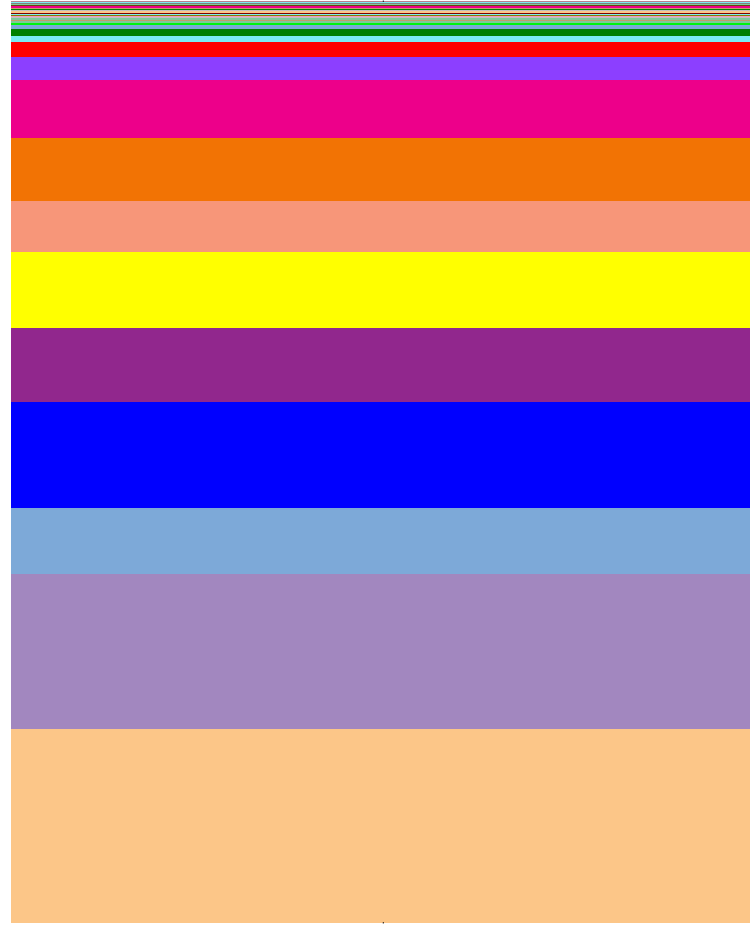

illness

Supplement: Supplementary file 5 — Additional file 5. [file 12917_2022_3189_MOESM5_ESM.zip › MPL201709200_16s_yy/Treat1/B07_taxa_summary_group/taxa_summary_plots/charts/j6Slql3l4ZnulPrpQ0Y92zNZ3gwdrC.pdf]

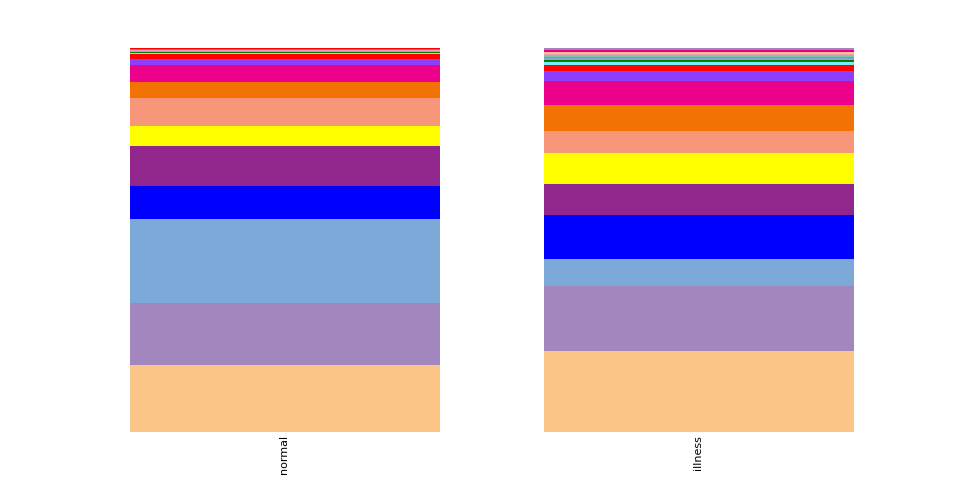

Supplement: Supplementary file 5 — Additional file 5. [file 12917_2022_3189_MOESM5_ESM.zip › MPL201709200_16s_yy/Treat1/B07_taxa_summary_group/taxa_summary_plots/charts/mJ6Bruk5AF7HEz6BcqIiukoq4TKlsj.png]

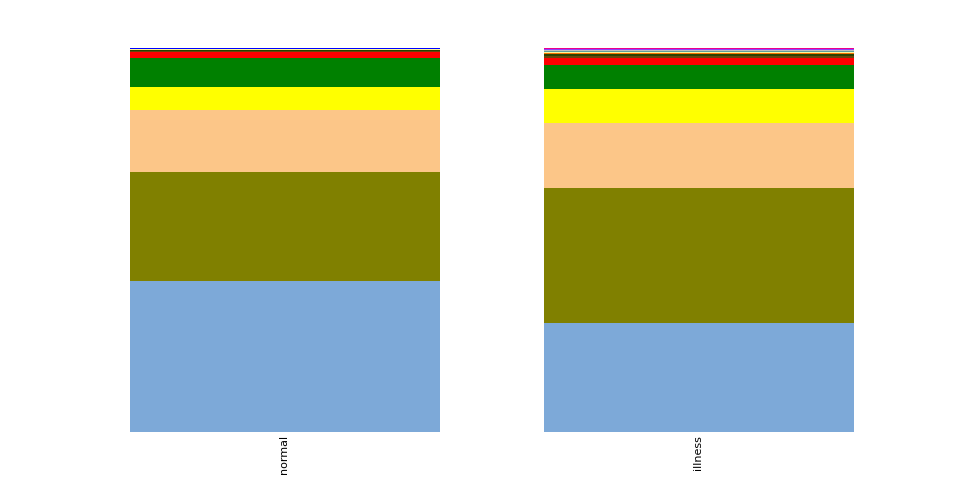

Supplement: Supplementary file 5 — Additional file 5. [file 12917_2022_3189_MOESM5_ESM.zip › MPL201709200_16s_yy/Treat1/B07_taxa_summary_group/taxa_summary_plots/charts/nZdBlfzPZIu39wJtxbZFYuofi2wXyQ.png]

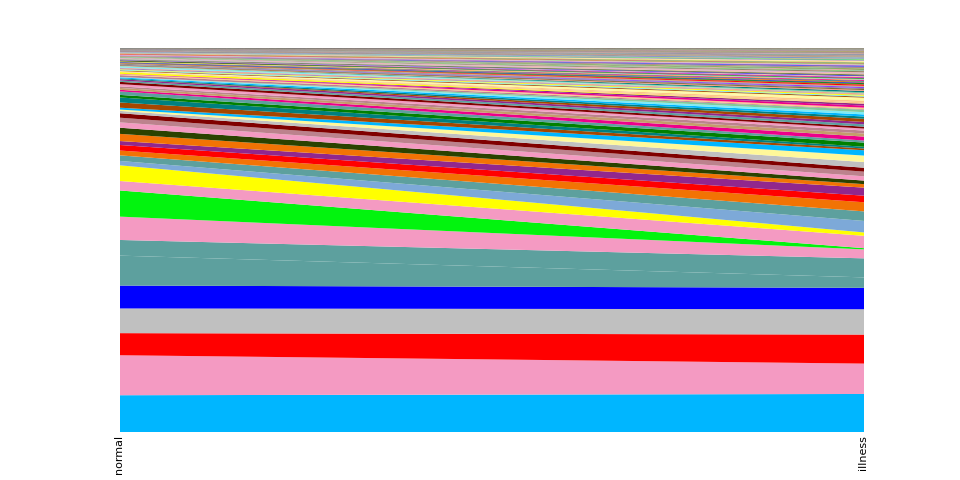

Supplement: Supplementary file 5 — Additional file 5. [file 12917_2022_3189_MOESM5_ESM.zip › MPL201709200_16s_yy/Treat1/B07_taxa_summary_group/taxa_summary_plots/charts/sfcUzUfyJZhB6rreRnr095gtWGznci.png]

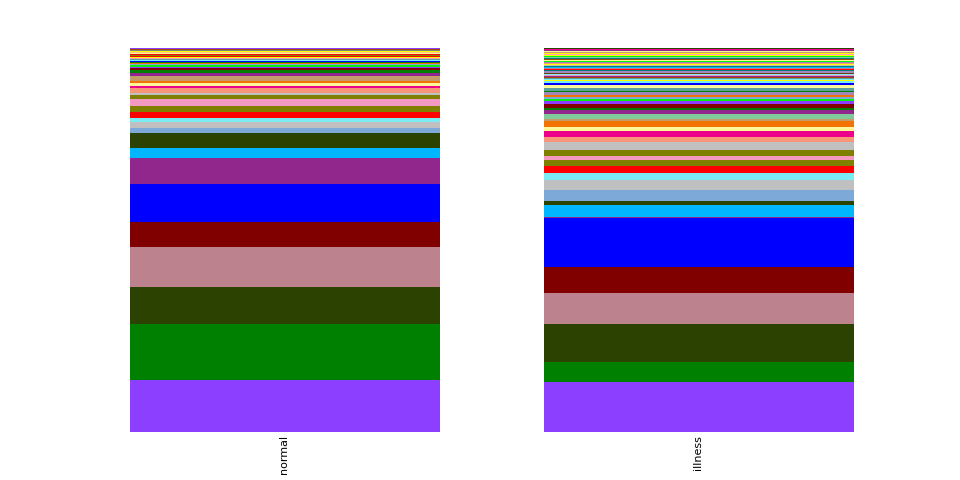

Supplement: Supplementary file 5 — Additional file 5. [file 12917_2022_3189_MOESM5_ESM.zip › MPL201709200_16s_yy/Treat1/B07_taxa_summary_group/taxa_summary_plots/charts/uZyDXQ0O9HrAMAPkbI39GDAd1SUjpg.png]

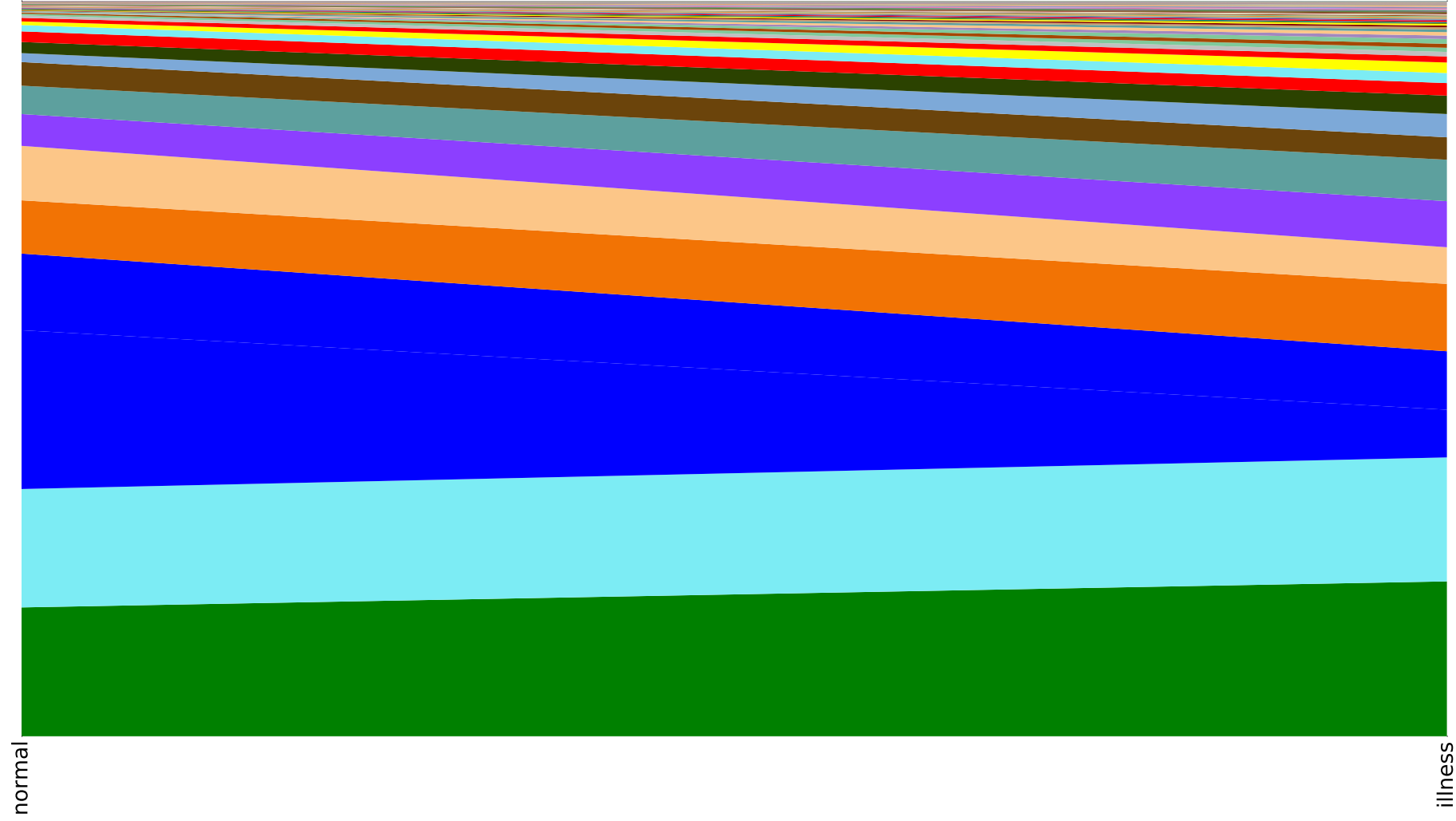

Supplement: Supplementary file 5 — Additional file 5. [file 12917_2022_3189_MOESM5_ESM.zip › MPL201709200_16s_yy/Treat1/B07_taxa_summary_group/taxa_summary_plots/charts/y4WWM2OzL8obqeYfzBp97gwKccdgzE.pdf]

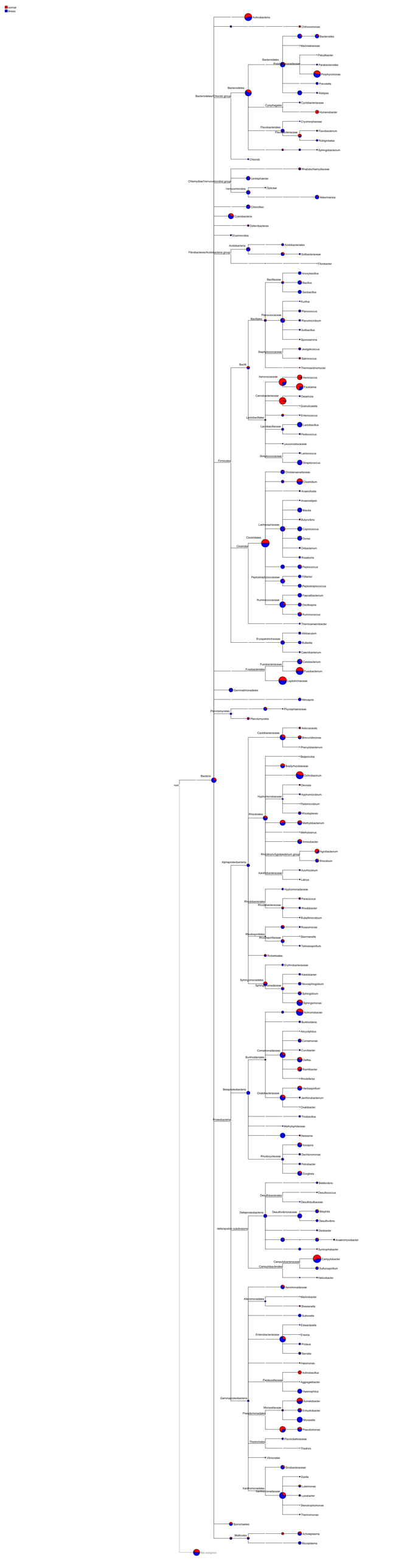

Supplement: Supplementary file 5 — Additional file 5. [file 12917_2022_3189_MOESM5_ESM.zip › MPL201709200_16s_yy/Treat1/B08_megan/megan.pdf]

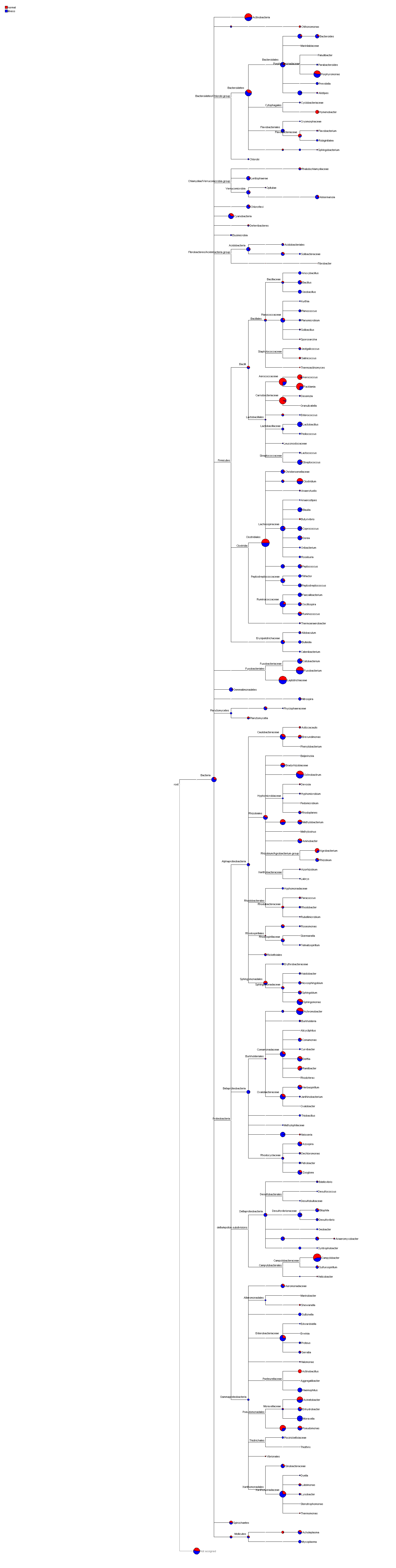

Supplement: Supplementary file 5 — Additional file 5. [file 12917_2022_3189_MOESM5_ESM.zip › MPL201709200_16s_yy/Treat1/B08_megan/megan.png]

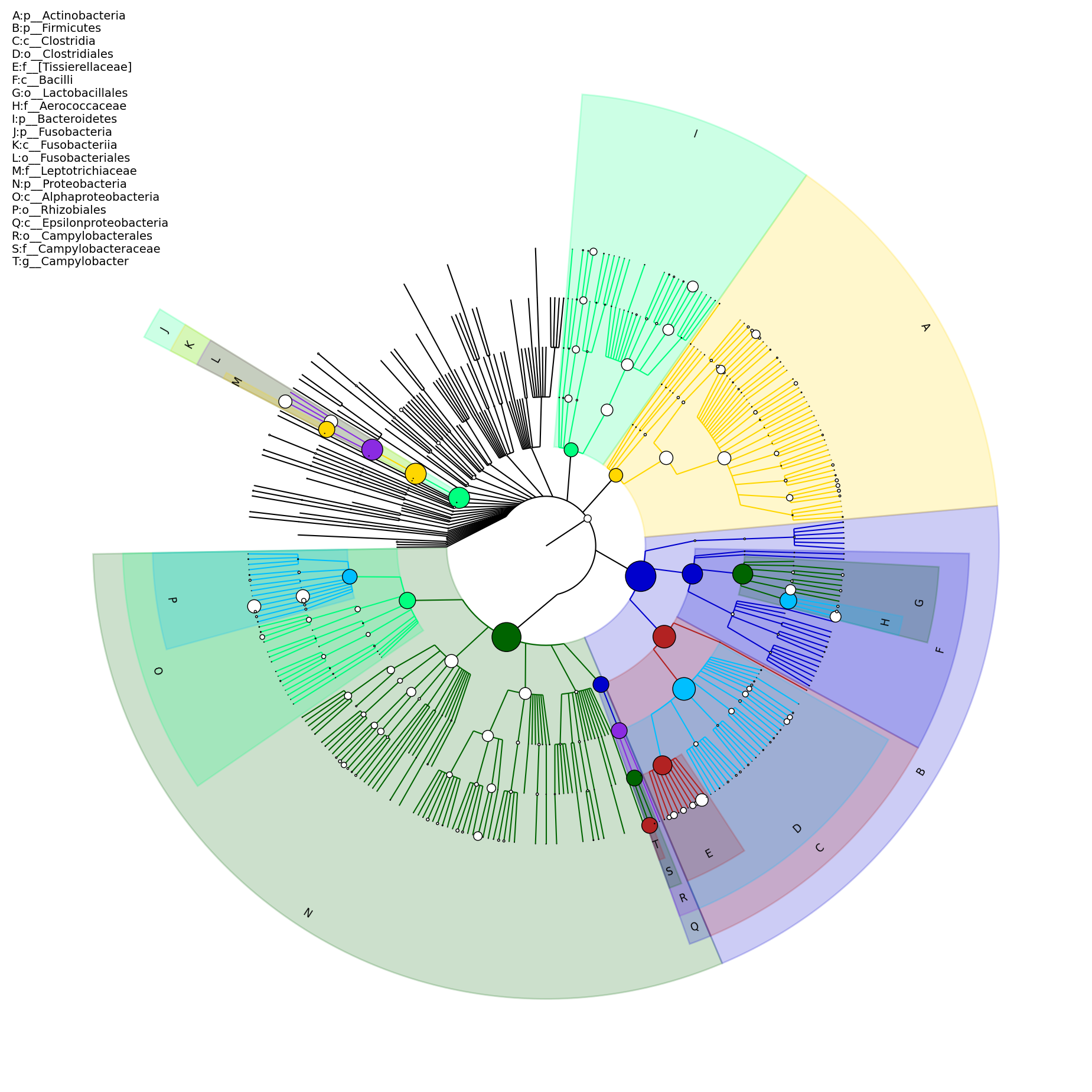

Supplement: Supplementary file 5 — Additional file 5. [file 12917_2022_3189_MOESM5_ESM.zip › MPL201709200_16s_yy/Treat1/B09_graphlan/tree.png]

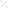

Supplement: Supplementary file 5 — Additional file 5. [file 12917_2022_3189_MOESM5_ESM.zip › MPL201709200_16s_yy/Treat1/B10_krona/src/hidden.png]

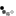

Supplement: Supplementary file 5 — Additional file 5. [file 12917_2022_3189_MOESM5_ESM.zip › MPL201709200_16s_yy/Treat1/B10_krona/src/loading.gif]

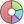

Supplement: Supplementary file 5 — Additional file 5. [file 12917_2022_3189_MOESM5_ESM.zip › MPL201709200_16s_yy/Treat1/B10_krona/src/logo.png]

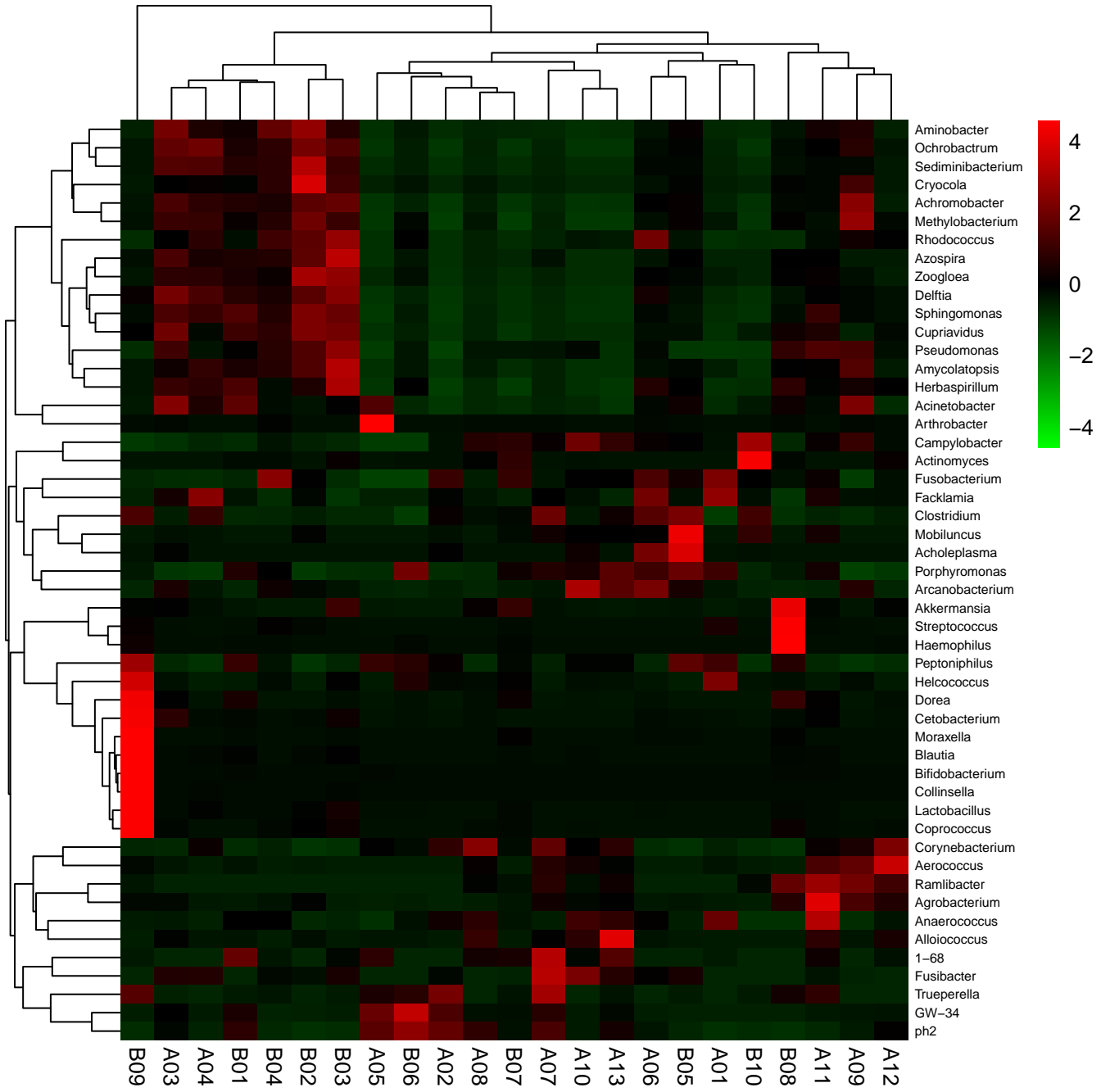

Supplement: Supplementary file 5 — Additional file 5. [file 12917_2022_3189_MOESM5_ESM.zip › MPL201709200_16s_yy/Treat1/B11_heatmap/heatmap.pdf]

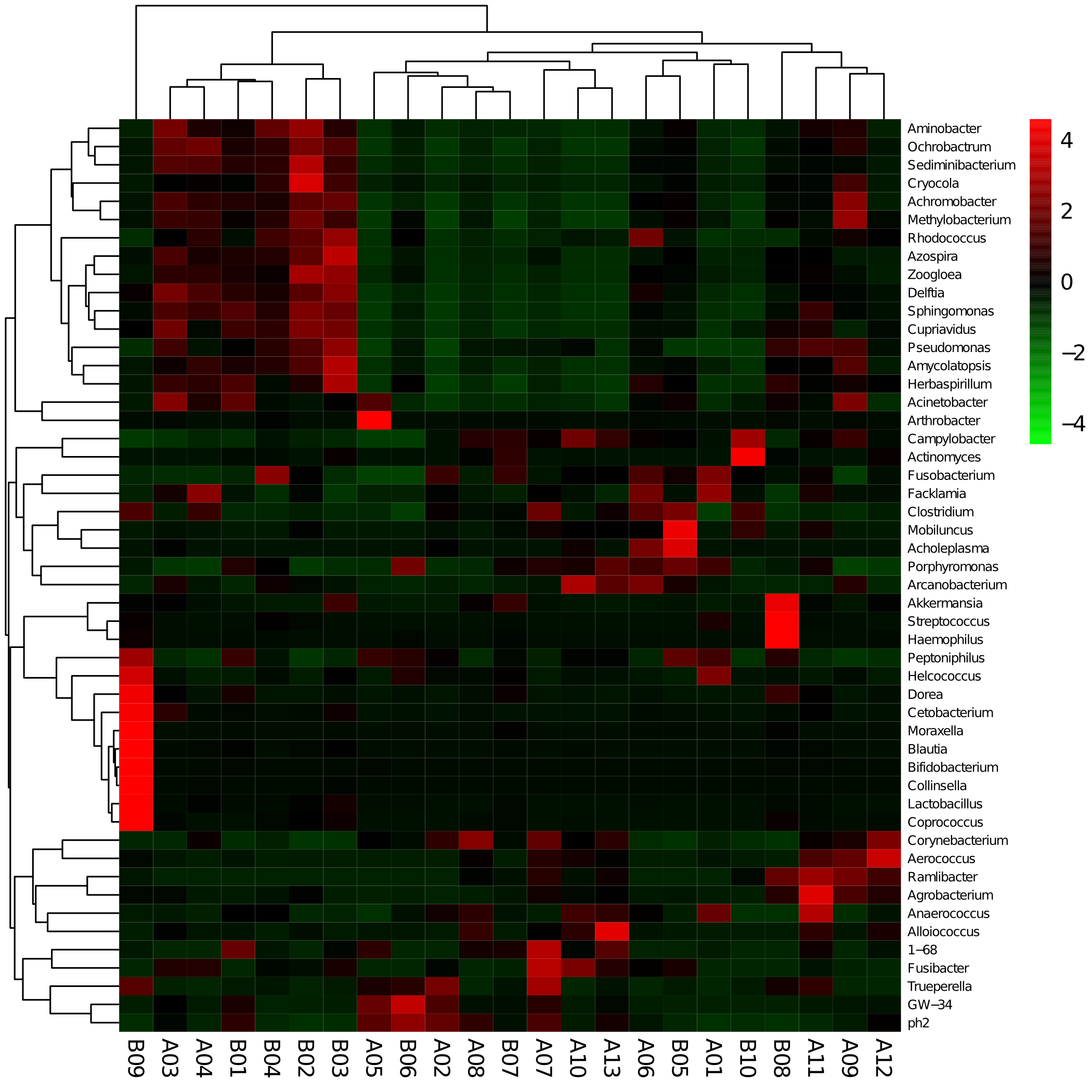

Supplement: Supplementary file 5 — Additional file 5. [file 12917_2022_3189_MOESM5_ESM.zip › MPL201709200_16s_yy/Treat1/B11_heatmap/heatmap.png]

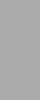

Supplement: Supplementary file 5 — Additional file 5. [file 12917_2022_3189_MOESM5_ESM.zip › MPL201709200_16s_yy/Treat1/B12_pca/3D-PCA/euclidean_emperor_pcoa_plot/emperor_required_resources/css/images/ui-bg_flat_0_aaaaaa_40x100.png]

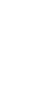

Supplement: Supplementary file 5 — Additional file 5. [file 12917_2022_3189_MOESM5_ESM.zip › MPL201709200_16s_yy/Treat1/B12_pca/3D-PCA/euclidean_emperor_pcoa_plot/emperor_required_resources/css/images/ui-bg_flat_75_ffffff_40x100.png]

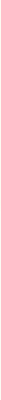

Supplement: Supplementary file 5 — Additional file 5. [file 12917_2022_3189_MOESM5_ESM.zip › MPL201709200_16s_yy/Treat1/B12_pca/3D-PCA/euclidean_emperor_pcoa_plot/emperor_required_resources/css/images/ui-bg_glass_55_fbf9ee_1x400.png]

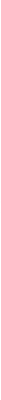

Supplement: Supplementary file 5 — Additional file 5. [file 12917_2022_3189_MOESM5_ESM.zip › MPL201709200_16s_yy/Treat1/B12_pca/3D-PCA/euclidean_emperor_pcoa_plot/emperor_required_resources/css/images/ui-bg_glass_65_ffffff_1x400.png]
